# Spectrum of Genetic Variants in the Most Common Genes Causing Inherited Retinal Disease in a Large Molecularly Characterized United Kingdom Cohort

**DOI:** 10.1016/j.oret.2024.01.012

**Published:** 2024-07

**Authors:** Siying Lin, Sandra Vermeirsch, Nikolas Pontikos, Maria Pilar Martin-Gutierrez, Malena Daich Varela, Samantha Malka, Elena Schiff, Hannah Knight, Genevieve Wright, Neringa Jurkute, Mark J. Simcoe, Patrick Yu-Wai-Man, Mariya Moosajee, Michel Michaelides, Omar A. Mahroo, Andrew R. Webster, Gavin Arno

**Affiliations:** 1 National Institute of Health Research Biomedical Research Centre at Moorfields Eye Hospital and the UCL Institute of Ophthalmology, London, United Kingdom; 2 UCL Institute of Ophthalmology, University College London, United Kingdom; 3 Department of Neuro-Ophhalmology, The National Hospital for Neurology and Neurosurgery, University College London Hospitals NHS Foundation Trust, London, United Kingdom; 4 Department of Ophthalmology, St Thomas’ Hospital, London, United Kingdom

**Keywords:** Genotypes, Inherited retinal disease, Pathogenic alleles, Variant classification, Variants

## Abstract

**Purpose:**

Inherited retinal disease (IRD) is a leading cause of blindness. Recent advances in gene-directed therapies highlight the importance of understanding the genetic basis of these disorders. This study details the molecular spectrum in a large United Kingdom (UK) IRD patient cohort.

**Design:**

Retrospective study of electronic patient records.

**Participants:**

Patients with IRD who attended the Genetics Service at Moorfields Eye Hospital between 2003 and July 2020, in whom a molecular diagnosis was identified.

**Methods:**

Genetic testing was undertaken via a combination of single-gene testing, gene panel testing, whole exome sequencing, and more recently, whole genome sequencing. Likely disease-causing variants were identified from entries within the genetics module of the hospital electronic patient record (OpenEyes Electronic Medical Record). Analysis was restricted to only genes listed in the Genomics England PanelApp R32 Retinal Disorders panel (version 3.24), which includes 412 genes associated with IRD. Manual curation ensured consistent variant annotation and included only plausible disease-associated variants.

**Main Outcome Measures:**

Detailed analysis was performed for variants in the 5 most frequent genes (*ABCA4*, *USH2A*, *RPGR*, *PRPH2*, and *BEST1*), as well as for the most common variants encountered in the IRD study cohort.

**Results:**

We identified 4415 individuals from 3953 families with molecularly diagnosed IRD (variants in 166 genes). Of the families, 42.7% had variants in 1 of the 5 most common IRD genes. Complex disease alleles contributed to disease in 16.9% of affected families with *ABCA4*-associated retinopathy. *USH2A* exon 13 variants were identified in 43% of affected individuals with *USH2A*-associated IRD. Of the *RPGR* variants, 71% were clustered in the ORF15 region. *PRPH2* and *BEST1* variants were associated with a range of dominant and recessive IRD phenotypes. Of the 20 most prevalent variants identified, 5 were not in the most common genes; these included founder variants in *CNGB3*, *BBS1*, *TIMP3*, *EFEMP1*, and *RP1*.

**Conclusions:**

We describe the most common pathogenic IRD alleles in a large single-center multiethnic UK cohort and the burden of disease, in terms of families affected, attributable to these variants. Our findings will inform IRD diagnoses in future patients and help delineate the cohort of patients eligible for gene-directed therapies under development.

**Financial Disclosure(s):**

Proprietary or commercial disclosure may be found in the Footnotes and Disclosures at the end of this article.

Inherited retinal disease (IRD) is the leading cause of blindness certification in the working-age population in several countries.^1^^,^^2^ Affected individuals often develop visual impairment at a young age with a normal life expectancy; as such, these visually disabling conditions are associated with a significant impact on quality of life with emotional and economic burden on affected individuals and their families.

Establishing a precise clinical and molecular diagnosis for IRD can provide useful prognostic information and enables informed genetic counseling and risk analysis for affected individuals and members of the wider family. However, determining the underlying genetic cause in IRD is often challenging due to the broad phenotypic and genetic heterogeneity observed. Advances in next-generation sequencing technologies facilitating the sequencing of whole exomes or genomes of large cohorts of individuals with IRD, alongside functional assays evaluating the impact of implicated variants, have driven significant improvements in molecular diagnostic rates.^3^^,^^4^ Together with developments in multimodal imaging and functional assessments permitting deep phenotypic characterization of retina structure and function,^5^^,^^6^ this has led to a better understanding of the spectrum, frequency, and disease burden of IRD-associated genes and gene variants within a population cohort.

Our group has previously reported on the burden of IRD attributable to different genes in a large molecularly characterized United Kingdom (UK) patient cohort, with the molecular diagnosis in > 70% of affected families consequent upon pathogenic variants in the 20 most common genes within the cohort.^7^ This study aims to present the spectrum of genetic variants in the same cohort and to specifically describe the molecular spectrum in the 5 most encountered genes, namely, *ABCA4*, *USH2A*, *RPGR*, *PRPH2*, and *BEST1*.

## Methods

### Genetic Testing Pathway at Moorfields Eye Hospital

The Genetics Service of Moorfields Eye Hospital (London, UK) receives secondary, tertiary, and quaternary referrals from across the UK for patients with a suspected IRD. A detailed clinical history is obtained from each patient, as well as full medical and family history. An ophthalmic examination is performed including assessment of visual acuity, intraocular pressure, slit-lamp biomicroscopy and fundoscopy, spectral-domain OCT and fundus autofluorescence, and color fundus photos when relevant. Electrodiagnostic testing is requested at the discretion of the treating physician.

If, after a full history and clinical assessment, IRD is suspected, genetic testing is then discussed with the patient. Genetic testing was initially performed via Sanger sequencing of single genes or small gene panels, or through arrayed primer extension-based microarray assays (Asper Biotech Ltd) for retinitis pigmentosa (RP), Leber congenital amaurosis, and Stargardt disease. In recent years, this has been superseded by next-generation sequencing methods including targeted gene panels, whole exome sequencing, and whole genome sequencing (WGS) techniques. The costs of genetic testing are typically covered within a clinical or research setting by the UK’s National Health Service or by research funding, including from the National Institute of Health Research. A full description of the genetic testing pathways has been published previously by Pontikos et al^7^ in 2020.

### Interrogation of the Genetic Database

For each patient, variants identified through genetic testing are assessed by clinical scientists to evaluate likely pathogenicity and contribution to the disease phenotype. Where the genetic etiology remains uncertain or undetermined, results are discussed within a multidisciplinary setting including IRD clinical specialists, genetic counselors, and clinical and research scientists. The discussion includes a review of medical history, family history, retinal imaging, and electrophysiological studies where available, which then directs further interrogation of the genomic data targeted at specific candidate genes. Likely disease-causing variants are entered within the genetics module of the hospital electronic patient record (OpenEyes Electronic Medical Record, Apperta Foundation). Each patient and family pedigree has a unique identifier. In this study, the backend database was interrogated retrospectively to identify all individuals with a molecularly diagnosed IRD. The search date was July 1, 2020, and all patients with a patient encounter since 2003 were identified.

### Manual Curation of Genetic Database

The Moorfields Genetic Database includes all affected individuals with a molecularly diagnosed inherited ocular disease. To identify only affected individuals with IRD as pertinent to this study, the database was filtered only for genes listed in the Genomics England PanelApp (https://panelapp.genomicsengland.co.uk/) R32 Retinal Disorders (version 3.24) panel, which includes 412 genes. Further analysis of variants identified in the 5 most frequently implicated IRD genes in the Moorfields cohort (*ABCA4*, *USH2A*, *RPGR*, *PRPH2*, and *BEST1*) was subsequently performed. Variants classified by the American College of Medical Genetics & Genomics or UK-based Association of Clinical Genomic Science guidelines as pathogenic or likely pathogenic, as well as variants of uncertain significance that were consistent with the clinical phenotype, were included in the analysis. In specific instances, variants classified as benign or likely benign were also included following multidisciplinary team discussion where existing evidence was felt to support causal association or possible contribution to disease phenotype. All variants were analyzed via Mutalyzer (https://mutalyzer.nl/) and VarSome (https://varsome.com/) to ensure sequence variant nomenclature according to Human Genome Variation Society recommendations. Variants are described according to the most clinically relevant transcript for each gene; namely NM_000350.3 (*ABCA4*), NM_206933.4 (*USH2A*), NM_001034853.2 (*RPGR*), NM_000322.5 (*PRPH2*), and NM_004183.4 (*BEST1*).

### Consent and Ethical Approval

This study received relevant local research ethics approval (Moorfields Eye Hospital and the Northwest London research ethics committee) and was performed in accordance with the tenets of the Declaration of Helsinki. Written informed consent for genetic testing was obtained from all participating individuals (or, for children, from their parent or legal guardian).

## Results

The total study cohort included 4415 individuals from 3953 families with a molecular diagnosis thought to account for their IRD, with variants in 166 genes identified (full data set provided in Table S1, available at www.ophthalmologyretina.org). In the cohort, 1468 individuals (33.3%) were of White ethnicity, 378 (8.6%) were Asian (largely South Asian), 109 (2.5%) were Black (African, Caribbean, and other Black background), 43 (1.0%) were of mixed race or ethnicity, 367 (8.3%) were of other ethnicities, and in 2050 individuals (46.4%), their ethnicities were unknown or undisclosed.

In addition, 1840 individuals from 1688 families had disease-associated variants in 1 of the 5 most frequently implicated genes (by numbers of affected families), accounting for 42.7% of all molecularly diagnosed families. The key cohort characteristics of these 5 genes are summarized in Table 2.

**Table 2:** Key Demographic Characteristics of the 5 Most Implicated Genes within the IRD Cohort

Gene	Chromosomal Location	Modes of Inheritance	Affected Families (%)	Affected Individuals (%) (M/F Ratio)
*ABCA4*	1p22.1	Recessive	858 (21.7%)	915 (20.7%) (452M/463F)
*USH2A*	1q41	Recessive	337 (8.5%)	355 (8.0%) (187M/168F)
*RPGR*	Xp11.4	X-linked	190 (4.8%)	228 (5.2%) (193M/35F∗)
*PRPH2*	6p21.1	Dominant & recessive	164 (4.1%)	189 (4.3%) (94M/95F)
*BEST1*	11q12.3	Dominant & recessive	140 (3.5%)	153 (3.7%) (91M/62F)

F = female; IRD = inherited retinal disease; M = male.

∗ Denotes female carriers with retinopathy consistent with an *RPGR* carrier phenotype.

### *ABCA4* (MIM ∗601691)

Variants in *ABCA4* have been associated with a range of phenotypes including macular, cone, and cone-rod dystrophy. This was the most prevalent gene implicated in IRD within this cohort, accounting for the molecular diagnosis in 915 individuals from 858 families. Among these individuals, 108 (11.8%) were homozygous for disease-causing alleles, whereas 807 individuals (88.2%) were compound heterozygous or presumed compound heterozygous.

Four hundred eleven distinct disease-associated variants in *ABCA4* were identified (Table S1); of these, 250 (60.8%) were missense variants, 85 (20.7%) were protein-truncating stopgain and frameshifting variants, 69 (16.8%) variants were predicted to affect splicing (including both canonical and noncanonical splice site variants as well as synonymous variants), 5 (1.2%) were inframe indels, and 1 downstream variant and 1 variant affecting the start codon were also identified. One hundred sixty-three patients with ABCA4-associated retinopathy underwent WGS via the 100KGP or National Institute for Health Research Bioresource rare disease study with all introns covered. Altogether, the 5 most encountered *ABCA4* variants contributed to disease in 50.2% of all families with *ABCA4*-associated retinopathy (Table 3).

**Table 3:** Frequency of the Most Common *ABCA4* Variants within the Study Cohort

*ABCA4* Variant	Number of Alleles	Affected Individuals	Affected Families
c.5882G>A; p.(Gly1961Glu)	213 (11.6%)	196 (21.4%)	190 (22.1%)
c.5461-10T>C	119 (6.5%)	114 (12.5%)	108 (12.6%)
c.2588G>C; p.(Gly863Ala)	98 (5.4%)	98 (10.7%)	94 (11.0%)
c.4139C>T; p.(Pro1380Leu)	57 (3.1%)	57 (6.2%)	54 (6.3%)
c.5714+5G>A	51 (2.8%)	49 (5.4%)	47 (5.5%)

More than 2 *ABCA4* variants were identified in 154 individuals from 145 families, indicating the presence of complex *ABCA4* disease alleles in these individuals. There were 136 individuals with 3 *ABCA4* variants, 17 individuals with 4 *ABCA4* variants, and 1 individual with 5 *ABCA4* variants identified. The c.1622T>C; p.(Leu541Pro) and c.3113C>T; p.(Ala1038Val) variants were likely to occur in *cis* on the same haplotype as a well-established *ABCA4* disease-associated allele^8^ and accounted for 39 alleles in 36 individuals in our study cohort.

The c.5603A>T; p.(Asn1868Ile) variant contributed to known complex alleles in 22 individuals (23 alleles); this included the c.[5603A>T;5461-10T>C]; p.[(Asn1868Ile;?)] allele (16 alleles identified), the c.[2588G>C;5603A>T]; p.[(Gly863Ala;Asn1868Ile)] allele (5 alleles identified), and the c.[4469G>A;5603A>T]; p.[(Cys1490Tyr;Asn1868Ile)] allele (2 alleles identified). The c.5603A>T; p.(Asn1868Ile) variant was also identified as an independently causal variant segregating in *trans* with a severe allele in 33 individuals.

Nine families were identified in which affected individuals within the same family had different *ABCA4* genotypes. Among these families, 8 demonstrated a pseudodominant inheritance pattern with an affected parent or parents and ≥ 1 affected children, and in 1 family, there was an affected sibling pair and an affected cousin. Additionally, we noted an instance where unrelated affected partners shared the same pedigree identification. The most common *ABCA4* variants identified in Table 3 were found to contribute to disease in 8 of the 9 families segregating multiple variants; this included c.5461-10T>C in 4 families, c.5882G>A; p.(Gly1961Glu) in 3 families, and c.2588G>C; p.(Gly863Ala) in 1 family.

Two individuals were found to segregate disease-associated variants in both *ABCA4* and an additional IRD gene, with both genes contributing to the clinical phenotype. In 1 individual, homozygosity for *ABCA4* c.5882G>A; p.(Gly1961Glu) as well as *ABCC6* (NM_001171.6) c.708_709dup^9^; p.(Trp237SerfsTer22) variants resulted in a macular dystrophy phenotype as well as pseudoxanthoma elasticum, with both ocular and dermatological features noted. Another individual was identified as having *ABCA4* c.5196+1056A>G and c.5882G>A; p.(Gly1961Glu) variants in *trans*, as well as homozygosity for *RPE65* (NM_000329.3) c.304G>T; p.(Glu102Ter). This individual was diagnosed with Leber congenital amaurosis at age 3.5 years, and at their last visit at age 8 years, fundus examination showed mild retinal pigmentary mottling and attenuated vessels, consistent with *RPE65*-associated retinopathy, with no signs of *ABCA4*-associated maculopathy.

### *USH2A* (MIM ∗608400)

Biallelic variants in *USH2A* are associated with autosomal recessive Usher syndrome type 2 and nonsyndromic RP. Variants in *USH2A* were the second most frequent cause of IRD within the study cohort, with disease-associated variants identified in 355 individuals from 337 families. Among these individuals, 62 (17.4%) were homozygous for disease-causing alleles, whereas 293 individuals (82.5%) were compound heterozygous or presumed compound heterozygous. Two hundred seven individuals had a diagnosis of Usher syndrome type 2, whereas 148 individuals had a diagnosis of nonsyndromic RP. Of the disease-associated variants in *USH2A*, 257 distinct were identified; of these, 108 (42.0%) were missense variants, 101 (39.3%) were protein-truncating stopgain and frameshifting variants, 30 (11.7%) variants were predicted to affect splicing, 4 (1.6%) were inframe indels, and 14 (5.4%) were deletions and duplications involving ≥ 1 exons of the *USH2A* gene. Missense variants were more commonly identified in individuals with nonsyndromic RP than Usher syndrome, accounting for 73.3% and 22.0% of disease alleles, respectively, whereas stopgain and frameshift variants were more commonly encountered in individuals with Usher syndrome (65.0% of disease alleles) than nonsyndromic RP (20.9% of disease alleles).

Table 4 summarizes the most encountered *USH2A* variants within the study cohort. Together, these 5 variants contributed to disease in 50.1% of all families with *USH2A*-associated IRD.

**Table 4:** Frequency of the Most Common *USH2A* Variants within the Study Cohort

*USH2A* Variant	Number of alleles	Affected individuals	Affected families
c.2299del; p.(Glu767SerfsTer21)	130 (18.3%)	114 (32.1%)	111 (32.9%)
c.2276G>T; p.(Cys759Phe)	37 (5.2%)	36 (10.1%)	35 (10.4%)
c.10073G>A; p.(Cys3358Tyr)	28 (3.9%)	28 (7.9%)	26 (7.7%)
c.5012G>A; p.(Gly1671Asp)	19 (2.7%)	10 (2.8%)	7 (2.1%)
c.920_923dup; p.(His308GlnfsTer16)	16 (2.3%)	15 (4.2%)	13 (3.9%)

Nine distinct variants located within exon 13, including the common c.2299del and c.2276G>T variants, were identified in 153 affected individuals, which represents 43% of all affected individuals with *USH2A*-associated retinal dystrophy within our study cohort.

More than 2 *USH2A* variants were identified in 15 individuals from 15 families, indicating the presence of complex *USH2A* disease alleles in these individuals. There were 13 individuals harboring 3 *USH2A* variants, and 2 individuals with 4 *USH2A* variants were identified.

### *RPGR* (MIM ∗312610)

*RPGR* was the third most encountered gene within the study cohort, accounting for the molecular diagnosis in 228 individuals from 190 families. Variants in *RPGR* are associated with X-linked IRD with a range of phenotypes including RP (most common), cone-rod dystrophy, and isolated cone dystrophy. Female *RPGR* carriers can present with a disease phenotype of variable severity, often with significant asymmetry between eyes. This study cohort, therefore, includes both affected male individuals as well as female carriers with clinical or electrophysiological features of retinopathy or retinal dysfunction in keeping with *RPGR*-associated IRD.

One hundred seventeen distinct disease-associated variants in *RPGR* were identified (Table S1); of these, 13 (11.1%) were missense variants, 88 (75.2%) were protein-truncating stopgain and frameshifting variants, 10 (8.5%) variants were predicted to affect splicing, and 6 (5.1%) were deletions involving ≥ 1 exons of the *RPGR* gene.

Table 5 summarizes the most frequently encountered *RPGR* variants within the study cohort. Together, these 5 variants contributed to disease in 27.9% of all families with *RPGR*-associated IRD.

**Table 5:** Frequency of the Most Common *RPGR* Variants within the Study Cohort

*RPGR* Variant	Number of Alleles	Affected Individuals	Affected Families
c.2405_2406del; p.(Glu802GlyfsTer32)	23 (10.1%)	23 (10.1%) (18M/5F)	15 (7.9%)
c.2426_2427del; p.(Glu809GlyfsTer25)	21 (9.2%)	21 (9.2%) (17M/4F)	16 (8.4%)
c.2236_2237del; p.(Glu746ArgfsTer23)	12 (5.3%)	12 (5.3%) (10M/2F)	11 (5.8%)
c.1234C>T; p.(Arg412Ter)	8 (3.5%)	8 (3.5%) (7M/1F)	6 (3.2%)
c.2384del; p.(Glu795GlyfsTer20)	6 (2.6%)	6 (2.6%) (5M/1F)	5 (2.6%)

F = female; M = male.

Approximately 71% of all *RPGR* disease-associated alleles (comprised largely of small deletions or duplications resulting in frameshifts) were clustered in the terminal exon (open reading frame 15 [ORF15]; c.1754-3459) of the RPGR^ORF15^ isoform, including 60% in the central highly repetitive 1 kb purine-rich region (c.2184-3162) that is particularly difficult to sequence using standard next-generation sequencing-based technologies.^10^

### *PRPH2* (MIM ∗179605)

*PRPH2* variants are associated with a range of disease phenotypes including macular dystrophy, RP, and Leber congenital amaurosis. Variants in this gene are generally associated with an autosomal dominant inheritance pattern; autosomal recessive inheritance has, however, also been reported.^11^

Sixty-eight distinct disease-associated variants in *PRPH2* were identified in 189 individuals from 164 families; of these, 39 (57.4%) were missense variants, 21 (30.9%) were protein-truncating stopgain and frameshifting variants, 3 (4.4%) variants were predicted to affect splicing, 4 (5.9%) were inframe indel variants, and there was 1 deletion (1.5%) involving ≥ 1 exons of the *PRPH2* gene. Two individuals had homozygous *PRPH2* variants associated with autosomal recessive early-onset retinal dystrophy, whereas the remaining 187 individuals had monoallelic *PRPH2* variants associated with autosomal dominant IRD.

Table 6 summarizes the most encountered *PRPH2* variants within the study cohort; 3 variants had allele counts of 6 and tied for the fifth most encountered *PRPH2* variant. Together, these variants contributed to disease in 47.7% of all families with *PRPH2*-associated IRD.

**Table 6:** Frequency of the Most Common *PRPH2* Variants within the Study Cohort

*PRPH2* Variant	Number of Alleles	Affected Individuals	Affected Families
c.514C>T; p.(Arg172Trp)	40 (20.9%)	40 (21.2%)	34 (20.7%)
c.394del; p.(Gln132LysfsTer7)	15 (7.9%)	15 (7.9%)	15 (9.1%)
c.259_266del; p.(Asp87GlnfsTer87)	9 (4.7%)	9 (4.8%)	8 (4.9%)
c.515G>A; p.(Arg172Gln)	7 (3.7%)	7 (3.7%)	6 (3.7%)
c.634A>G; p.(Ser212Gly)	6 (3.1%)	6 (3.2%)	6 (3.7%)
c.136C>T; p.(Arg46Ter)	6 (3.1%)	6 (3.2%)	5 (3.0%)
c.612C>G; p.(Tyr204Ter)	6 (3.1%)	6 (3.2%)	4 (2.4%)

One individual was found to carry a pathogenic c.136C>T; p.(Arg46Ter) stopgain variant in *PRPH2* as well as biallelic pathogenic *ABCC6* variants (NM_001171.6) c.3389C>T; p.(Thr1130Met) and c.4104del; p.(Asp1368GlufsTer35) that are likely in *trans.*^12^ This individual displayed ocular features including angioid streaks and a peau d’orange appearance of the peripheral retina, in keeping with *ABCC6*-associated pseudoxanthoma elasticum, and also had a vitelliform macular dystrophy likely due to a *PRPH2*-associated retinal dystrophy.

### *BEST1* (MIM ∗607854)

Variants in *BEST1* cause a range clinically heterogeneous retinal dystrophies, including autosomal dominant macular dystrophy, autosomal recessive bestrophinopathy (ARB), and the overlapping phenotypes of autosomal dominant vitreoretinochoroidopathy and RP. *BEST1* variants were the fifth most common cause of IRD within our study cohort.

Ninety-two distinct disease-associated variants in *BEST1* were identified in 153 affected individuals from 140 families; of these, 74 (80.4%) were missense variants, 9 (9.8%) were protein-truncating stopgain and frameshifting variants, 5 (5.4%) variants were predicted to affect splicing, 3 (3.3%) were inframe indel variants, and there was 1 deletion (1.1%) involving exons 1−2 of the *BEST1* gene. Biallelic variants were reported in 37 individuals (manifesting ARB); 17 individuals were homozygous for disease-causing variants, whereas 20 individuals were compound heterozygous or presumed compound heterozygous. Monoallelic variants were reported in 116 individuals: 115 individuals with autosomal dominant macular dystrophy, and 1 individual with RP associated with a heterozygous c.682G>A; p.(Asp228Asn) variant that has previously been reported in association with this phenotype.^13^

Table 7 summarizes the most encountered *BEST1* variants within our study cohort. Together, these 5 variants contributed to disease in 22.1% of all families with *BEST1*-associated IRD.

**Table 7:** Frequency of the Most Common *BEST1* Variants within the Study Cohort

*BEST1* Variant	Number of Alleles	Affected Individuals	Affected Families
c.652C>T; p.(Arg218Cys)	11 (5.8%)	11 (7.2%)	10 (7.1%)
c.728C>T; p.(Ala243Val)	11 (5.8%)	11 (7.2%)	8 (5.7%)
c.653G>A; p.(Arg218His)	10 (5.3%)	10 (6.5%)	9 (6.3%)
c.418C>G; p.(Leu140Val)	8 (4.2%)	4 (2.6%)	4 (2.9%)

Subsequent variants have an allele count of < 5.

The *BEST1* c.418C>G; p.(Leu140Val) variant was identified in homozygous form in 4 individuals with unaffected parents from 4 separate families with ARB and was not associated with disease in monoallelic form in our study cohort, consistent with prior publications describing it exclusively in association with ARB.^13^^,^^14^

*BEST1* variants associated with both dominant and recessive disease were identified in our study cohort: c.73C>T; p.(Arg25Trp) and c.74G>A; p.(Arg25Gln). Both variants were identified in a single individual each in monoallelic form, with a phenotype consistent with autosomal dominant vitelliform macular dystrophy. The p.(Arg25Trp) variant was also identified in a single individual in homozygous form, with a clinical diagnosis of ARB and whose parents denied any visual symptoms. The p.(Arg25Gln) variant was identified in compound heterozygous form with c.278G>A; p.(Trp93Ter) in an individual with ARB. Segregation analysis indicated that this variant was inherited from the patient’s father, who had been noted to have bilateral retinal changes that were thought to be in keeping with central serous chorioretinopathy, whereas the p.(Trp93Ter) variant was inherited from a clinically unaffected mother. Both *BEST1* p.(Arg25Trp) and p.(Arg25Gln) variants have been reported in association with both autosomal dominant macular dystrophy^15^^,^^16^ and ARB,^17^^,^^18^ and it may well be that both variants act in a semidominant manner.

One individual homozygous for a c.-37+1G>T variant in *BEST1* affecting the intron 1 splice donor site was initially diagnosed with ARB but was also noted to have peripheral retinal degenerative changes as well as a profoundly subnormal rod-specific electroretinogram recording, features that are not typical of the condition. Reinterrogation of the genomic data subsequently identified an additional homozygous pathogenic *RAX2* missense variant (NM_032753.4) c.247C>T; p.(Arg83Cys). Biallelic *RAX2* variants are associated with autosomal recessive RP,^19^ and dysfunction of both are thought to contribute to the clinical phenotype in this individual.

### Most Frequent Variants in the Cohort

**Figure 1:**
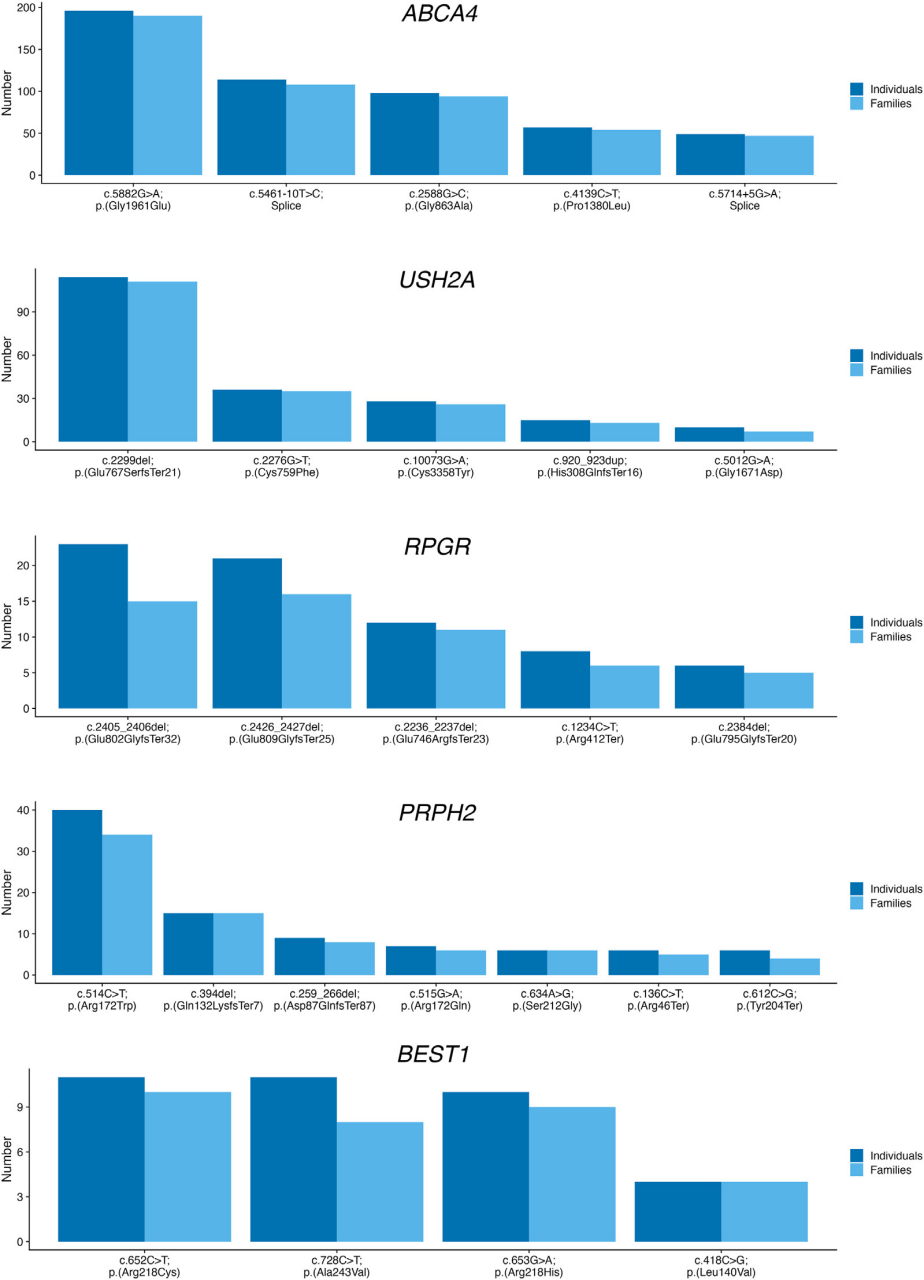
Bar graphs showing the numbers of affected individuals and families with the most common variants in *ABCA4*, *USH2A*, *RPGR*, *PRPH2*, and *BEST1*.

Figure 1 illustrates the most common variants in the 5 most encountered genes (by number of affected individuals and families, respectively).

Table 8 summarizes the most frequent variants identified in the study cohort and includes all variants that have been identified in ≥ 20 alleles within the study cohort.

**Table 8:** Frequently Encountered Variants within the IRD Study Cohort

Gene	Transcript	Nucleotide Change	Protein Change	Number of Alleles
*ABCA4*	NM_000350.3	c.5882G>A	p.(Gly1961Glu)	213 (2.9%)
*USH2A*	NM_206933.4	c.2299del	p.(Glu767SerfsTer21)	130 (1.8%)
*ABCA4*	NM_000350.3	c.5461-10T>C	Affects splicing	119 (1.6%)
*CNGB3*	NM_019098.5	c.1148del	p.(Thr383IlefsTer13)	116 (1.6%)
*ABCA4*	NM_000350.3	c.2588G>C	p.(Gly863Ala)	98 (1.3%)
*ABCA4*	NM_000350.3	c.4139C>T	p.(Pro1380Leu)	57 (0.8%)
*ABCA4*	NM_000350.3	c.5714+5G>A	Affects splicing	51 (0.7%)
*BBS1*	NM_024649.5	c.1169T>G	p.(Met390Arg)	50 (0.7%)
*ABCA4*	NM_000350.3	c.3113C>T	p.(Ala1038Val)	46 (0.6%)
*PRPH2*	NM_000322.5	c.514C>T	p.(Arg172Trp)	44 (0.6%)
*ABCA4*	NM_000350.3	c.1622T>C	p.(Leu541Pro)	41 (0.6%)
*ABCA4*	NM_000350.3	c.4469G>A	p.(Cys1490Tyr)	41 (0.6%)
*TIMP3*	NM_000362.5	c.610A>T	p.(Ser204Cys)	39 (0.5%)
*USH2A*	NM_206933.4	c.2276G>T	p.(Cys759Phe)	37 (0.5%)
*ABCA4*	NM_000350.3	c.6079C>T	p.(Leu2027Phe)	36 (0.5%)
*EFEMP1*	NM_001039348.3	c.1033C>T	p.(Arg345Trp)	35 (0.5%)
*RP1*	NM_006269.2	c.2029C>T	p.(Arg677Ter)	33 (0.5%)
*ABCA4*	NM_000350.3	c.5603A>T	p.(Asn1868Ile)	33 (0.5%)
*ABCA4*	NM_000350.3	c.3322C>T	p.(Arg1108Cys)	32 (0.4%)
*ABCA4*	NM_000350.3	c.6729+5_6729+19del	Affects splicing	32 (0.4%)
*ABCA4*	NM_000350.3	c.6658C>T	p.(Gln2220Ter)	29 (0.4%)
*USH2A*	NM_206933.4	c.10073G>A	p.(Cys3358Tyr)	28 (0.4%)
*ABCA4*	NM_000350.3	c.4577C>T	p.(Thr1526Met)	27 (0.4%)
*CYP4V2*	NM_207352.4	c.197T>G	p.(Met66Arg)	26 (0.4%)
*PROM1*	NM_006017.3	c.1117C>T	p.(Arg373Cys)	26 (0.4%)
*CERKL*	NM_001030311.3	c.847C>T	p.(Arg283Ter)	24 (0.3%)
*ABCA4*	NM_000350.3	c.3064G>A	p.(Glu1022Lys)	23 (0.3%)
*ABCA4*	NM_000350.3	c.6089G>A	p.(Arg2030Gln)	23 (0.3%)
*CNGA3*	NM_001298.3	c.1641C>A	p.(Phe547Leu)	23 (0.3%)
*RPGR*	NM_001034853.2	c.2405_2406del	p.(Glu802GlyfsTer32)	23 (0.3%)
*RP1*	NM_006269.2	c.2596_2597del	p.(Leu866LysfsTer7)	23 (0.3%)
*ABCA4*	NM_000350.3	c.634C>T	p.(Arg212Cys)	22 (0.3%)
*MFSD8*	NM_152778.3	c.1361T>C	p.(Met454Thr)	22 (0.3%)
*NR2E3*	NM_014249.4	c.119-2A>C	Affects splicing	21 (0.3%)
*RPGR*	NM_001034853.2	c.2426_2427del	p.(Glu809GlyfsTer25)	21 (0.3%)
*ABCA4*	NM_000350.3	c.6320G>A	p.(Arg2107His)	21 (0.3%)
*RP1*	NM_006269.2	c.2172_2185del	p.(Ile725ArgfsTer6)	20 (0.3%)
*ABCA4*	NM_000350.3	c.3259G>A	p.(Glu1087Lys)	20 (0.3%)
*PRPF31*	NM_015629.4	c.527+3A>G	Affects splicing	20 (0.3%)
*RHO*	NM_000539.3	c.1040C>T	p.(Pro347Leu)	20 (0.3%)

IRD = inherited retinal disease.

The 5 most frequently encountered variants: *ABCA4* c.5882G>A; p.(Gly1961Glu), c.2588G>C; p.(Gly863Ala), and c.5461-10T>C, together with *USH2A* c.2299del; p.(Glu767SerfsTer21) and *CNGB3* c.1148del; p.(Thr383IlefsTer13), account for almost 9.3% of all disease-associated variants within the IRD study cohort.

### Quantifying the Burden of Private Mutations in the IRD Cohort

Table 9 summarizes the proportion of variants that were private (identified in only a single family) or recurrent (identified in ≥ 2 unrelated families) in the 5 most encountered genes in the IRD cohort.

**Table 9:** Proportion of Private and Recurrent Variants in Each Gene

Gene	Number of Distinct Variants	Private Mutations	Recurrent Mutations
*ABCA4*	411	248 (60.3%)	163 (39.7%)
*USH2A*	256	168 (65.6%)	88 (34.4%)
*RPGR*	117	93 (79.5%)	24 (20.5%)
*PRPH2*	68	46 (67.6%)	22 (32.3%)
*BEST1*	92	59 (64.1%)	33 (35.9%)

## Discussion

We present a survey of the most frequent disease-associated alleles in 4415 individuals with IRD from 3953 families in a large single-center multiethnic UK cohort and explore the molecular spectrum of the 5 genes most associated with IRD in our study cohort.

The most encountered gene within our study cohort was *ABCA4*, with the contribution of frequent variants broadly consistent with findings described by Cornelis et al^20^ in a large meta-analysis of published *ABCA4*-associated retinal dystrophy cases. The hypomorphic c.5882G>A; p.(Gly1961Glu) variant was the most prevalent *ABCA4* disease-associated allele (n = 213 [11.6%]) and also the most frequent IRD-associated variant overall in the study cohort (Table 8). This variant originates from East Africa, with a population frequency of up to 10% in the Somali population.^21^ In contrast, the most prevalent *ABCA4* variant in the Spanish Stargardt population is a founder variant c.3386G>T; p.(Arg1129Leu), accounting for almost 25% of deleterious alleles in this population but only identified in a single affected individual in our cohort.^22^

Complex alleles contributed to *ABCA4*-associated disease pathogenesis in > 16% of affected individuals and families. It is increasingly recognized that the molecular landscape of *ABCA4*-retinopathy is complex, where disease penetrance and severity is dependent on the specific combination of variants acting in *cis* and in *trans*.^23^ This is typified by the hypomorphic p.(Asn1868Ile) variant, which was the fourth most encountered *ABCA4* variant (Table 3) and the sixth most frequent IRD-associated variant overall in our study cohort (Table 8). This variant has a minor allele frequency of almost 7% in populations of non-Finnish European descent and is significantly enriched in patients with Stargardt disease compared with population controls.^8^^,^^24^ It resides in *cis* with known *ABCA4*-disease variants including c.5601-10T>C or p.(Cys1409Tyr) and contributes to disease as a complex allele with the hypomorphic p.(Gly863Ala) *ABCA4* variant. This variant has only recently been recognized and reported as disease-causing in the absence of *cis* variants but only when inherited in *trans* with a severe or moderately severe *ABCA4* allele.^24^ In view of this relatively recent discovery, we believe that the p.(Asn1868Ile) variant is likely underrepresented as an independent disease within our study cohort; future retrospective analyses of our unsolved monoallelic *ABCA4* patients, particularly in mildly affected individuals, may yet lead to the additional identification of p.(Asn1868Ile) contributing to further molecular diagnoses.

*USH2A* was the second most encountered disease-associated gene in this cohort; the most commonly encountered variant was c.2299del; p.(Glu767SerfsTer21), which was present in 32% of all individuals with *USH2A*-associated retinal dystrophy and is the second most encountered variant overall in our IRD study cohort (Table 8). This variant is thought to have arisen on an ancestral European haplotype^25^ and is particularly prevalent in *USH2A* patients of European descent, with allele frequencies ranging from 15% to 45% depending on the population studied.^26^, ^27^, ^28^ Another common European founder variant is c.2276G>T; p.(Cys759Phe),^29^^,^^30^ which was the second most frequent *USH2A* variant in our study cohort. Overall, disease variants located within exon 13 of the *USH2A* gene were identified in > 40% of all affected individuals in our *USH2A* IRD cohort. These variants are targeted by antisense oligonucleotide therapies currently under development,^31^ which if successful have the potential to benefit a significant proportion of patients with *USH2A*-associated retinal dystrophy.

Variants in *RPGR* are associated with X-linked retinal dystrophy, and it is well recognized that female carriers can show a disease phenotype of variable severity.^32^ Indeed, within our study cohort, approximately 15% of all individuals with *RPGR*-associated retinal dystrophy were carrier females manifesting a disease phenotype. Approximately 71% of all *RPGR* disease-associated alleles were clustered in the terminal exon (ORF15) of the RPGR^ORF15^ isoform. This highly repetitive purine-rich region is especially difficult to sequence using standard paired-end short-read sequencing technology, which includes the WGS platform currently used as the standard diagnostic test for investigating retinal disorders in the UK. As such, it is important for clinicians to have an awareness and consideration for *RPGR*-associated disease, especially in individuals or families where affected females may demonstrate a mild or asymmetrical phenotype, allowing for targeted analysis of the *RPGR* ORF15 region to be specified.

*PRPH2* and *BEST1* have a relatively high presence in IRD study cohorts^28^^,^^33^^,^^34^ and were the fourth and fifth most encountered genes, respectively, in this study cohort. Clinical diagnosis is often challenging as both can be associated with a range of distinct clinical phenotypes inherited in both autosomal dominant (more commonly) or recessive forms.^11^^,^^35^^,^^36^ The c.514C>T; p.(Arg172Trp) variant in *PRPH2* has previously been identified through haplotype analysis to be a founder mutation in the British population^37^ and, in keeping with this, was also the most commonly reported *PRPH2* variant in our study cohort, accounting for > 20% of all disease alleles. Another founder mutation in *PRPH2*, the c.828+3A>T splice site variant, has been reported as the most frequently occurring variant in a large *PRPH2*-related retinopathy cohort of 187 affected individuals in the United States^38^; however, this variant was only identified in a single individual in our cohort.

Founder and recurrent variants were among the most encountered variants within the study cohort (Table 8), with the most common variants contributing to disease in approximately 20% to 30% of families with IRD due to variants in *ABCA4*, *RPGR*, and *BEST1* and up to 50% for families with *USH2A*- and *PRPH2*-associated IRD. Despite this, the majority of variants within these genes remain private mutations identified in only a single family within the cohort, where pathogenicity, especially for previously unreported variants, can be difficult to ascertain. This is particularly the case for larger genes such as *ABCA4* and *USH2A*, which are prone to greater variation by virtue of increased transcript length, contributing to the not infrequent occurrence of complex alleles in these genes. Within our study cohort, there were several *ABCA4* families identified where affected individuals within the same family segregate different pathogenic variants in *ABCA4*, highlighting the possibility of high frequency alleles being independently causative within families in a pseudodominant inheritance pattern. Additionally, there were several individuals in our study cohort with variants in 2 different IRD-associated genes contributing to the clinical presentation, emphasizing the importance of full survey of the genomic data. The Genetics Service at Moorfields Eye Hospital serves London’s ethnically diverse and multicultural population characterized by inward migration and a significant proportion of foreign-born communities; it also receives referrals from throughout the UK. As such, where family members reside at a distance or overseas, familial samples for segregation studies can sometimes be difficult to obtain; even where this is available, the often-high population allele frequencies of the more commonly encountered hypomorphic *ABCA4* variants can preclude informative segregation. These frequently encountered difficulties in variant interpretation can often complicate disease diagnosis and prognostication.

Our service benefits from an expert multidisciplinary team approach, where variants can be assessed in the context of an individual’s clinical phenotype, and a targeted interrogation of genomic regions linked to disease presentation can be performed; this approach has been beneficial in improving molecular diagnostic rates and improving understanding of genotype-phenotype correlation.^39^ Consideration should also be given to the coexistent use of emerging long-read sequencing technologies that will allow haplotype phasing, better detection and characterization of structural variation, and analysis of regions of the genome intractable to short-read next-generation sequencing such as the *OPN1LW/OPN1MW* opsin gene array.^40^

There are several limitations to this study, largely inherent to its retrospective nature, which we have previously highlighted.^7^ One significant issue is the reliance on historical data entry, which has been disadvantaged by inconsistent sequence nomenclature and inadvertent error introduction. We have addressed this with a thorough manual curation of all sequence variants identified (presented in Table S1), which now allows a more accurate understanding of the molecular epidemiology of our IRD study cohort. The time limited analysis of our patient data between 2003 and 2020 precludes the inclusion of newer disease genes that have since been identified in association with IRD pathogenesis; in addition, the relatively recent recognition of hypomorphic variants previously thought benign that are now considered disease-causing suggests these variants are likely underrepresented in our analysis. For individuals with likely complex alleles, haplotype phasing was often unavailable or not documented, although there are ongoing attempts to address this deficiency through interrogation of WGS data where available or through additional long-read sequencing research studies. Phenotypic subgroups were not readily extracted in an automatic fashion from the electronic data record due to historical variability in data entry and diagnostic labeling; this was manually curated for affected individuals and families with *USH2A* and *BEST1*-associated IRD disease due to the associated phenotypic variability but was not practicable for the entire IRD cohort. Furthermore, given the diverse and multifaceted testing strategies used in our cohort, the method(s) of genetic testing for individual patients was not consistently available in their electronic patient records, posing a challenge in comprehensively describing the individual testing strategies employed in our patient population. In recent years, there has been a large drive toward the integration of genomics within mainstream ophthalmology practice within the UK, with centralized funding facilitating wide availability of genetic testing through the newly created National Genomic Medicine Service. In parallel with this, there is an increasing acknowledgment of the molecular complexity of inherited eye diseases and challenges with disease variant interpretation. This study quantifies the burden attributable to genes and gene variants contributing to IRD disease in a large UK-based patient cohort, which to our knowledge represents the largest molecularly diagnosed IRD cohort to date. We have described the most common variants in the most frequently implicated genes (and also the most frequently encountered variants overall) and have additionally (in Table S1) provided data relating to all variants in the entire cohort as a useful reference for other clinicians and investigators. This knowledge will provide a framework for efficient interpretation and analysis of putative disease variants that will be identified through increasingly available genetic testing for IRD patients, resulting in improvement in diagnosis and management of affected individuals and their families. We also quantify the disease burden attributable to particular IRD subtypes and thereby delineate the cohort of patients eligible for novel gene-directed therapeutic strategies under development, allowing research and health care resources to be directed toward areas of greatest need and potential benefit.
