# Supplementary material for: Spectrum of Genetic Variants in the Most Common Genes Causing Inherited Retinal Disease in a Large Molecularly Characterized United Kingdom Cohort
Source: Ophthalmol Retina. 2024 Jul;8(7):699–709. doi: 10.1016/j.oret.2024.01.012 (PMC11932969; doi:10.1016/j.oret.2024.01.012)
Supplement: Table S1 [file mmc1.pdf]

Sample ID	Pedigree ID	Gene	Transcript	Variant (nucleotide)	Variant (protein)	Variant Type	ACMG Classification	Sex	Age	Ethnicity
350454	1	ABCA4	NM_000350.3	c.1037A>C	p.(Lys346Thr)	Missense	Variant of Uncertain Significance	M	61	Not stated
350454	1	ABCA4	NM_000350.3	c.4139C>T	p.(Pro1380Leu)	Missense	Pathogenic	M	61	Not stated
3824484	2	ABCA4	NM_000350.3	c.6088C>T	p.(Arg2030Ter)	Stopgain	Pathogenic	F	65	Mixed - White and Asian
3824484	2	ABCA4	NM_000350.3	c.6229C>T	p.(Arg2077Trp)	Missense	Pathogenic	F	65	Mixed - White and Asian
3824484	2	ABCA4	NM_000350.3	c.769-3C>T	Splice	Splice	Variant of Uncertain Significance	F	65	Mixed - White and Asian
382472	3	ABCA4	NM_000350.3	c.3392_3393delinsG	p.(Ala1131GlyfsTer17)	Frameshift	Likely Pathogenic	M	84	Asian - Indian
382472	3	ABCA4	NM_000350.3	c.3392_3393delinsG	p.(Ala1131GlyfsTer17)	Frameshift	Likely Pathogenic	M	84	Asian - Indian
2929877	4	ABCA4	NM_000350.3	c.2588G>C	p.(Gly863Ala)	Missense	Pathogenic	F	73	White - British
2929877	4	ABCA4	NM_000350.3	c.5461-10T>C	Splice	Splice	Pathogenic	F	73	White - British
3500433	5	ABCA4	NM_000350.3	c.1317G>A	p.(Trp439Ter)	Stopgain	Pathogenic	F	40	Not stated
3500433	5	ABCA4	NM_000350.3	c.454C>T	p.(Arg152Ter)	Stopgain	Pathogenic	F	40	Not stated
3096939	6	ABCA4	NM_000350.3	c.1622T>C	p.(Leu541Pro)	Missense	Pathogenic	M	42	Not stated
3096939	6	ABCA4	NM_000350.3	c.1850_1851del	p.(Gln617ArgfsTer148)	Frameshift	Likely Pathogenic	M	42	Not stated
3096939	6	ABCA4	NM_000350.3	c.3113C>T	p.(Ala1038Val)	Missense	Pathogenic	M	42	Not stated
384152	7	ABCA4	NM_000350.3	c.2588G>C	p.(Gly863Ala)	Missense	Pathogenic	F	63	Not stated
384152	7	ABCA4	NM_000350.3	c.5461-10T>C	Splice	Splice	Pathogenic	F	63	Not stated
414294	8	ABCA4	NM_000350.3	c.1622T>C	p.(Leu541Pro)	Missense	Pathogenic	M	59	Not stated
414294	8	ABCA4	NM_000350.3	c.1622T>C	p.(Leu541Pro)	Missense	Pathogenic	M	59	Not stated
414294	8	ABCA4	NM_000350.3	c.3113C>T	p.(Ala1038Val)	Missense	Pathogenic	M	59	Not stated
414294	8	ABCA4	NM_000350.3	c.3113C>T	p.(Ala1038Val)	Missense	Pathogenic	M	59	Not stated
2615570	9	ABCA4	NM_000350.3	c.2041C>T	p.(Arg681Ter)	Stopgain	Pathogenic	M	60	Not stated
2615570	9	ABCA4	NM_000350.3	c.4918C>T	p.(Arg1640Trp)	Missense	Pathogenic	M	60	Not stated
6301651	10	ABCA4	NM_000350.3	c.1922G>C	p.(Cys641Ser)	Missense	Likely Pathogenic	M	36	Not stated
6301651	10	ABCA4	NM_000350.3	c.6079C>T	p.(Leu2027Phe)	Missense	Pathogenic	M	36	Not stated
1729349	11	ABCA4	NM_000350.3	c.5461-10T>C	Splice	Splice	Pathogenic	F	56	Not stated
1729349	11	ABCA4	NM_000350.3	c.5714+5G>A	Splice	Splice	Pathogenic	F	56	Not stated
3790058	12	ABCA4	NM_000350.3	c.2894A>G	p.(Asn965Ser)	Missense	Pathogenic	F	55	Asian - Indian
3790058	12	ABCA4	NM_000350.3	c.6158G>A	p.(Trp2053Ter)	Stopgain	Likely Pathogenic	F	55	Asian - Indian
259972	13	ABCA4	NM_000350.3	c.3210_3211dup	p.(Ser1071CysfsTer14)	Frameshift	Pathogenic	F	80	White - British
259972	13	ABCA4	NM_000350.3	c.4577C>T	p.(Thr1526Met)	Missense	Pathogenic	F	80	White - British
327781	13	ABCA4	NM_000350.3	c.3210_3211dup	p.(Ser1071CysfsTer14)	Frameshift	Pathogenic	F	74	White - British
327781	13	ABCA4	NM_000350.3	c.4577C>T	p.(Thr1526Met)	Missense	Pathogenic	F	74	White - British
4165776	14	ABCA4	NM_000350.3	c.3178C>T	p.(Gln1060Ter)	Stopgain	Likely Pathogenic	F	61	Any other ethnic group
4165776	14	ABCA4	NM_000350.3	c.5882G>A	p.(Gly1961Glu)	Missense	Pathogenic	F	61	Any other ethnic group
707195	15	ABCA4	NM_000350.3	c.1805G>A	p.(Arg602Gln)	Missense	Pathogenic	M	82	White - British
707195	15	ABCA4	NM_000350.3	c.4139C>T	p.(Pro1380Leu)	Missense	Pathogenic	M	82	White - British
17409580	15	ABCA4	NM_000350.3	c.1805G>A	p.(Arg602Gln)	Missense	Pathogenic	M	77	Not stated
17409580	15	ABCA4	NM_000350.3	c.4139C>T	p.(Pro1380Leu)	Missense	Pathogenic	M	77	Not stated
4318558	16	ABCA4	NM_000350.3	c.3210_3211dup	p.(Ser1071CysfsTer14)	Frameshift	Pathogenic	M	35	White - British
4318558	16	ABCA4	NM_000350.3	c.4139C>T	p.(Pro1380Leu)	Missense	Pathogenic	M	35	White - British
4283544	17	ABCA4	NM_000350.3	c.5461-10T>C	Splice	Splice	Pathogenic	F	37	White - British
4283544	17	ABCA4	NM_000350.3	c.6449G>A	p.(Cys2150Tyr)	Missense	Pathogenic	F	37	White - British
2668455	18	ABCA4	NM_000350.3	c.2345G>A	p.(Trp782Ter)	Stopgain	Pathogenic	M	40	White - British
2668455	18	ABCA4	NM_000350.3	c.4469G>A	p.(Cys1490Tyr)	Missense	Pathogenic	M	40	White - British
1649059	19	ABCA4	NM_000350.3	c.161G>A	p.(Cys54Tyr)	Missense	Pathogenic	F	68	Not stated
1649059	19	ABCA4	NM_000350.3	c.2588G>C	p.(Gly863Ala)	Missense	Pathogenic	F	68	Not stated
3487826	20	ABCA4	NM_000350.3	c.161G>A	p.(Cys54Tyr)	Missense	Pathogenic	F	60	Any other ethnic group
3487826	20	ABCA4	NM_000350.3	c.5461-10T>C	Splice	Splice	Pathogenic	F	60	Any other ethnic group
4948523	21	ABCA4	NM_000350.3	c.1760+1G>A	Splice	Splice	Likely Pathogenic	F	67	Not stated
4948523	21	ABCA4	NM_000350.3	c.4594G>T	p.(Asp1532Tyr)	Missense	Likely Pathogenic	F	67	Not stated

2614786	22	ABCA4	NM_000350.3	c.5882G>A	p.(Gly1961Glu)	Missense	Pathogenic	F	64	Any other ethnic group
2614786	22	ABCA4	NM_000350.3	c.6658C>T	p.(Gln2220Ter)	Stopgain	Pathogenic	F	64	Any other ethnic group
720299	23	ABCA4	NM_000350.3	c.4685T>C	p.(Ile1562Thr)	Missense	Pathogenic	M	73	Any other ethnic group
720299	23	ABCA4	NM_000350.3	c.6213C>G	p.(Tyr2071Ter)	Stopgain	Pathogenic	M	73	Any other ethnic group
5130292	23	ABCA4	NM_000350.3	c.4685T>C	p.(Ile1562Thr)	Missense	Pathogenic	F	65	Not stated
5130292	23	ABCA4	NM_000350.3	c.6213C>G	p.(Tyr2071Ter)	Stopgain	Pathogenic	F	65	Not stated
720313	23	ABCA4	NM_000350.3	c.4685T>C	p.(Ile1562Thr)	Missense	Pathogenic	M	74	Asian - Bangladeshi
720313	23	ABCA4	NM_000350.3	c.6213C>G	p.(Tyr2071Ter)	Stopgain	Pathogenic	M	74	Asian - Bangladeshi
6786555	24	ABCA4	NM_000350.3	c.4139C>T	p.(Pro1380Leu)	Missense	Pathogenic	M	66	Not stated
6786555	24	ABCA4	NM_000350.3	c.5196+1137G>A	Splice	Splice	Pathogenic	M	66	Not stated
4828739	25	ABCA4	NM_000350.3	c.4469G>A	p.(Cys1490Tyr)	Missense	Pathogenic	F	54	Not stated
4828739	25	ABCA4	NM_000350.3	c.4919G>A	p.(Arg1640Gln)	Missense	Pathogenic	F	54	Not stated
5129732	26	ABCA4	NM_000350.3	c.4363T>C	p.(Cys1455Arg)	Missense	Pathogenic	F	83	Not stated
5129732	26	ABCA4	NM_000350.3	c.5516T>C	p.(Phe1839Ser)	Missense	Likely Pathogenic	F	83	Not stated
5129732	26	ABCA4	NM_000350.3	c.466A>G	p.(Ile156Val)	Missense	Variant of Uncertain Significance	F	83	Not stated
274322	26	ABCA4	NM_000350.3	c.4363T>C	p.(Cys1455Arg)	Missense	Pathogenic	M	79	White - British
274322	26	ABCA4	NM_000350.3	c.5516T>C	p.(Phe1839Ser)	Missense	Likely Pathogenic	M	79	White - British
274322	26	ABCA4	NM_000350.3	c.466A>G	p.(Ile156Val)	Missense	Variant of Uncertain Significance	M	79	White - British
9046617	27	ABCA4	NM_000350.3	c.4129-1G>A	Splice	Splice	Pathogenic	M	61	White - British
9046617	27	ABCA4	NM_000350.3	c.5461-10T>C	Splice	Splice	Pathogenic	M	61	White - British
5245134	28	ABCA4	NM_000350.3	c.1230A>G	p.(Ile410Met)	Missense	Variant of Uncertain Significance	F	56	Not stated
5245134	28	ABCA4	NM_000350.3	c.6148G>C	p.(Val2050Leu)	Missense	Variant of Uncertain Significance	F	56	Not stated
6661038	29	ABCA4	NM_000350.3	c.4139C>T	p.(Pro1380Leu)	Missense	Pathogenic	F	34	Not stated
6661038	29	ABCA4	NM_000350.3	c.4577C>T	p.(Thr1526Met)	Missense	Pathogenic	F	34	Not stated
3553577	30	ABCA4	NM_000350.3	c.2588G>C	p.(Gly863Ala)	Missense	Pathogenic	F	65	Not stated
3553577	30	ABCA4	NM_000350.3	c.3259G>A	p.(Glu1087Lys)	Missense	Pathogenic	F	65	Not stated
4117581	31	ABCA4	NM_000350.3	c.4535_4537delinsAAA	p.(Pro1512_Gln1513delinsGlnLys	Inframe indel	Variant of Uncertain Significance	F	56	Asian - Other
4117581	31	ABCA4	NM_000350.3	c.5882G>A	p.(Gly1961Glu)	Missense	Pathogenic	F	56	Asian - Other
1564226	31	ABCA4	NM_000350.3	c.4535_4537delinsAAA	p.(Pro1512_Gln1513delinsGlnLys	Inframe indel	Variant of Uncertain Significance	F	63	Asian - Other
1564226	31	ABCA4	NM_000350.3	c.5882G>A	p.(Gly1961Glu)	Missense	Pathogenic	F	63	Asian - Other
4378450	32	ABCA4	NM_000350.3	c.5932A>G	p.(Lys1978Glu)	Missense	Pathogenic	M	53	Asian - Pakistani
4378450	32	ABCA4	NM_000350.3	c.5932A>G	p.(Lys1978Glu)	Missense	Pathogenic	M	53	Asian - Pakistani
5223595	33	ABCA4	NM_000350.3	c.3208_3209insGT	p.(Ser1071fsTer15)	Frameshift	Likely Pathogenic	F	78	Not stated
5223595	33	ABCA4	NM_000350.3	c.3386G>A	p.(Arg1129His)	Missense	Pathogenic	F	78	Not stated
5223595	33	ABCA4	NM_000350.3	c.4634G>A	p.(Ser1545Asn)	Missense	Variant of Uncertain Significance	F	78	Not stated
2939873	34	ABCA4	NM_000350.3	c.2588G>C	p.(Gly863Ala)	Missense	Pathogenic	M	52	Not stated
2939873	34	ABCA4	NM_000350.3	c.3322C>T	p.(Arg1108Cys)	Missense	Pathogenic	M	52	Not stated
2939873	34	ABCA4	NM_000350.3	c.2828G>A	p.(Arg943Gln)	Missense	Variant of Uncertain Significance	M	52	Not stated
5277334	35	ABCA4	NM_000350.3	c.2894A>G	p.(Asn965Ser)	Missense	Pathogenic	M	81	White - British
5277334	35	ABCA4	NM_000350.3	c.6089G>A	p.(Arg2030Gln)	Missense	Pathogenic	M	81	White - British
5277607	36	ABCA4	NM_000350.3	c.5461-10T>C	Splice	Splice	Pathogenic	M	51	Not stated
5277607	36	ABCA4	NM_000350.3	c.5714+5G>A	Splice	Splice	Pathogenic	M	51	Not stated
1944214	37	ABCA4	NM_000350.3	c.1253T>C	p.(Phe418Ser)	Missense	Pathogenic	F	72	Not stated
1944214	37	ABCA4	NM_000350.3	c.5714+5G>A	Splice	Splice	Pathogenic	F	72	Not stated
4812114	38	ABCA4	NM_000350.3	c.5461-10T>C	Splice	Splice	Pathogenic	F	48	Not stated
4812114	38	ABCA4	NM_000350.3	c.6079C>T	p.(Leu2027Phe)	Missense	Pathogenic	F	48	Not stated
788066	39	ABCA4	NM_000350.3	c.1622T>C	p.(Leu541Pro)	Missense	Pathogenic	M	59	White - British
788066	39	ABCA4	NM_000350.3	c.3113C>T	p.(Ala1038Val)	Missense	Pathogenic	M	59	White - British
788066	39	ABCA4	NM_000350.3	c.768G>T	p.(Val256Val)	Synonymous	Pathogenic	M	59	White - British
3690924	40	ABCA4	NM_000350.3	c.4469G>A	p.(Cys1490Tyr)	Missense	Pathogenic	F	57	White - British
3690924	40	ABCA4	NM_000350.3	c.6089G>A	p.(Arg2030Gln)	Missense	Pathogenic	F	57	White - British

5404902	41	ABCA4	NM_000350.3	c.1222C>T	p.(Arg408Ter)	Stopgain	Pathogenic	M	39	Unknown
5404902	41	ABCA4	NM_000350.3	c.2023G>A	p.(Val675Ile)	Missense	Pathogenic	M	39	Unknown
659770	42	ABCA4	NM_000350.3	c.4918C>T	p.(Arg1640Trp)	Missense	Pathogenic	M	56	White - British
659770	42	ABCA4	NM_000350.3	c.6079C>T	p.(Leu2027Phe)	Missense	Pathogenic	M	56	White - British
5248837	43	ABCA4	NM_000350.3	c.5461-10T>C	Splice	Splice	Pathogenic	F	52	White - British
5248837	43	ABCA4	NM_000350.3	c.6342G>A	p.(Val2114Val)	Missense	Likely Pathogenic	F	52	White - British
5595561	44	ABCA4	NM_000350.3	c.4539+2T>G	Splice	Splice	Likely Pathogenic	M	51	Not stated
5595561	44	ABCA4	NM_000350.3	c.5882G>A	p.(Gly1961Glu)	Missense	Pathogenic	M	51	Not stated
5519177	45	ABCA4	NM_000350.3	c.4139C>T	p.(Pro1380Leu)	Missense	Pathogenic	M	55	White - British
5519177	45	ABCA4	NM_000350.3	c.634C>T	p.(Arg212Cys)	Missense	Pathogenic	M	55	White - British
149729	46	ABCA4	NM_000350.3	c.2239del	p.(Leu747CysfsTer40)	Frameshift	Pathogenic	F	67	Not stated
149729	46	ABCA4	NM_000350.3	c.5196+1137G>A	Splice	Splice	Pathogenic	F	67	Not stated
3493188	47	ABCA4	NM_000350.3	c.293A>G	p.(Asn98Ser)	Missense	Likely Pathogenic	F	48	White - British
3493188	47	ABCA4	NM_000350.3	c.5196+1137G>A	Splice	Splice	Pathogenic	F	48	White - British
5634047	48	ABCA4	NM_000350.3	c.4539+2028C>T	Splice	Splice	Likely Pathogenic	F	44	White - British
5634047	48	ABCA4	NM_000350.3	c.5461-10T>C	Splice	Splice	Pathogenic	F	44	White - British
5667689	49	ABCA4	NM_000350.3	c.2588G>C	p.(Gly863Ala)	Missense	Pathogenic	F	56	White - British
5667689	49	ABCA4	NM_000350.3	c.6449G>A	p.(Cys2150Tyr)	Missense	Pathogenic	F	56	White - British
5828675	49	ABCA4	NM_000350.3	c.2588G>C	p.(Gly863Ala)	Missense	Pathogenic	F	59	White - British
5828675	49	ABCA4	NM_000350.3	c.6449G>A	p.(Cys2150Tyr)	Missense	Pathogenic	F	59	White - British
3416468	50	ABCA4	NM_000350.3	c.4537dup	p.(Gln1513ProfsTer42)	Frameshift	Pathogenic	F	57	Not stated
3416468	50	ABCA4	NM_000350.3	c.5578C>T	p.(Arg1860Trp)	Missense	Likely Pathogenic	F	57	Not stated
1695917	51	ABCA4	NM_000350.3	c.161G>A	p.(Cys54Tyr)	Missense	Pathogenic	F	70	White - British
1695917	51	ABCA4	NM_000350.3	c.2297G>T	p.(Gly766Val)	Missense	Likely Pathogenic	F	70	White - British
5666681	52	ABCA4	NM_000350.3	c.3056C>T	p.(Thr1019Met)	Missense	Pathogenic	F	60	Not stated
5666681	52	ABCA4	NM_000350.3	c.5381C>A	p.(Ala1794Asp)	Missense	Pathogenic	F	60	Not stated
663683	52	ABCA4	NM_000350.3	c.3056C>T	p.(Thr1019Met)	Missense	Pathogenic	M	58	Not stated
663683	52	ABCA4	NM_000350.3	c.5381C>A	p.(Ala1794Asp)	Missense	Pathogenic	M	58	Not stated
5812918	53	ABCA4	NM_000350.3	c.4469G>A	p.(Cys1490Tyr)	Missense	Pathogenic	F	49	Not stated
5812918	53	ABCA4	NM_000350.3	c.4469G>A	p.(Cys1490Tyr)	Missense	Pathogenic	F	49	Not stated
5745025	54	ABCA4	NM_000350.3	c.5461-10T>C	Splice	Splice	Pathogenic	M	49	Not stated
5745025	54	ABCA4	NM_000350.3	c.5714+5G>A	Splice	Splice	Pathogenic	M	49	Not stated
5803244	55	ABCA4	NM_000350.3	c.1760G>A	p.(Arg587Lys)	Missense	Variant of Uncertain Significance	F	34	White - British
5803244	55	ABCA4	NM_000350.3	c.2564G>A	p.(Trp855Ter)	Stopgain	Pathogenic	F	34	White - British
2232502	56	ABCA4	NM_000350.3	c.2588G>C	p.(Gly863Ala)	Missense	Pathogenic	M	71	White - British
2232502	56	ABCA4	NM_000350.3	c.4139C>T	p.(Pro1380Leu)	Missense	Pathogenic	M	71	White - British
2232502	56	ABCA4	NM_000350.3	c.5693G>A	p.(Arg1898His)	Missense	Likely Pathogenic	M	71	White - British
5780739	57	ABCA4	NM_000350.3	c.634C>T	p.(Arg212Cys)	Missense	Pathogenic	M	46	Asian - Other
5780739	57	ABCA4	NM_000350.3	c.634C>T	p.(Arg212Cys)	Missense	Pathogenic	M	46	Asian - Other
973545	58	ABCA4	NM_000350.3	c.1622T>C	p.(Leu541Pro)	Missense	Pathogenic	M	65	Not stated
973545	58	ABCA4	NM_000350.3	c.2069G>T	p.(Gly690Val)	Missense	Likely Pathogenic	M	65	Not stated
973545	58	ABCA4	NM_000350.3	c.3113C>T	p.(Ala1038Val)	Missense	Pathogenic	M	65	Not stated
1189635	59	ABCA4	NM_000350.3	c.2894A>G	p.(Asn965Ser)	Missense	Pathogenic	F	65	Not stated
1189635	59	ABCA4	NM_000350.3	c.3322C>T	p.(Arg1108Cys)	Missense	Pathogenic	F	65	Not stated
5952729	60	ABCA4	NM_000350.3	c.161G>A	p.(Cys54Tyr)	Missense	Pathogenic	F	50	Any other ethnic group
5952729	60	ABCA4	NM_000350.3	c.2588G>C	p.(Gly863Ala)	Missense	Pathogenic	F	50	Any other ethnic group
1015230	61	ABCA4	NM_000350.3	c.4195G>A	p.(Glu1399Lys)	Missense	Pathogenic	M	74	Asian - Indian
1015230	61	ABCA4	NM_000350.3	c.5882G>A	p.(Gly1961Glu)	Missense	Pathogenic	M	74	Asian - Indian
5496014	62	ABCA4	NM_000350.3	c.4469G>A	p.(Cys1490Tyr)	Missense	Pathogenic	M	43	Not stated
5496014	62	ABCA4	NM_000350.3	c.5196+1137G>A	Splice	Splice	Pathogenic	M	43	Not stated
8401924	63	ABCA4	NM_000350.3	c.3292C>T	p.(Arg1098Cys)	Missense	Pathogenic	M	37	Not stated

8401924	63	ABCA4	NM_000350.3	c.4139C>T	p.(Pro1380Leu)	Missense	Pathogenic	M	37	Not stated
5952533	64	ABCA4	NM_000350.3	c.4577C>T	p.(Thr1526Met)	Missense	Pathogenic	F	35	Asian - Indian
5952533	64	ABCA4	NM_000350.3	c.4577C>T	p.(Thr1526Met)	Missense	Pathogenic	F	35	Asian - Indian
41544	65	ABCA4	NM_000350.3	c.634C>T	p.(Arg212Cys)	Missense	Pathogenic	F	57	Not stated
41544	65	ABCA4	NM_000350.3	c.666_678del	p.(Lys223MetfsTer14)	Frameshift	Pathogenic	F	57	Not stated
638980	65	ABCA4	NM_000350.3	c.634C>T	p.(Arg212Cys)	Missense	Pathogenic	M	49	White - British
638980	65	ABCA4	NM_000350.3	c.666_678del	p.(Lys223MetfsTer14)	Frameshift	Pathogenic	M	49	White - British
1478882	66	ABCA4	NM_000350.3	c.1957C>T	p.(Arg653Cys)	Missense	Pathogenic	F	59	Unknown
1478882	66	ABCA4	NM_000350.3	c.6320G>A	p.(Arg2107His)	Missense	Pathogenic	F	59	Unknown
724023	67	ABCA4	NM_000350.3	c.6729+5_6729+19del	Splice	Splice	Likely Pathogenic	F	77	Asian - Indian
724023	67	ABCA4	NM_000350.3	c.6729+5_6729+19del	Splice	Splice	Likely Pathogenic	F	77	Asian - Indian
4901070	68	ABCA4	NM_000350.3	c.5461-10T>C	Splice	Splice	Pathogenic	M	39	Not stated
4901070	68	ABCA4	NM_000350.3	c.6079C>T	p.(Leu2027Phe)	Missense	Pathogenic	M	39	Not stated
4010544	69	ABCA4	NM_000350.3	c.4775G>A	p.(Gly1592Asp)	Missense	Variant of Uncertain Significance	F	44	White - British
4010544	69	ABCA4	NM_000350.3	c.6207C>T	p.(Gly2069Gly)	Synonymous	Variant of Uncertain Significance	F	44	White - British
3258212	69	ABCA4	NM_000350.3	c.4775G>A	p.(Gly1592Asp)	Missense	Variant of Uncertain Significance	F	45	White - British
3258212	69	ABCA4	NM_000350.3	c.6207C>T	p.(Gly2069Gly)	Synonymous	Variant of Uncertain Significance	F	45	White - British
3783688	70	ABCA4	NM_000350.3	c.5461-10T>C	Splice	Splice	Pathogenic	M	78	White - British
3783688	70	ABCA4	NM_000350.3	c.6089G>A	p.(Arg2030Gln)	Missense	Pathogenic	M	78	White - British
4777590	71	ABCA4	NM_000350.3	c.5461-10T>C	Splice	Splice	Pathogenic	M	43	Not stated
4777590	71	ABCA4	NM_000350.3	c.6079C>T	p.(Leu2027Phe)	Missense	Pathogenic	M	43	Not stated
3243260	72	ABCA4	NM_000350.3	c.4139C>T	p.(Pro1380Leu)	Missense	Pathogenic	F	66	White - British
3243260	72	ABCA4	NM_000350.3	c.5051T>A	p.(Ile1684Asn)	Missense	Variant of Uncertain Significance	F	66	White - British
6116837	73	ABCA4	NM_000350.3	c.2791G>A	p.(Val931Met)	Missense	Pathogenic	M	38	Unknown
6116837	73	ABCA4	NM_000350.3	c.5114G>A	p.(Arg1705Gln)	Missense	Pathogenic	M	38	Unknown
4374922	74	ABCA4	NM_000350.3	c.3191-1G>T	Splice	Splice	Likely Pathogenic	F	37	Not stated
4374922	74	ABCA4	NM_000350.3	c.5377G>A	p.(Val1793Met)	Missense	Pathogenic	F	37	Not stated
5982626	75	ABCA4	NM_000350.3	c.6320G>A	p.(Arg2107His)	Missense	Pathogenic	M	42	Black - African
5982626	75	ABCA4	NM_000350.3	c.6455G>A	p.(Gly2152Asp)	Missense	Likely Pathogenic	M	42	Black - African
5761706	76	ABCA4	NM_000350.3	c.2588G>C	p.(Gly863Ala)	Missense	Pathogenic	F	46	White - British
5761706	76	ABCA4	NM_000350.3	c.5461-10T>C	Splice	Splice	Pathogenic	F	46	White - British
5761706	76	ABCA4	NM_000350.3	c.2828G>A	p.(Arg943Gln)	Missense	Variant of Uncertain Significance	F	46	White - British
6185150	77	ABCA4	NM_000350.3	c.185C>T	p.(Pro62Leu)	Missense	Pathogenic	F	38	White - British
6185150	77	ABCA4	NM_000350.3	c.4253+5G>A	Splice	Splice	Likely Pathogenic	F	38	White - British
6185150	77	ABCA4	NM_000350.3	c.5603A>T	p.(Asn1868Ile)	Missense	Variant of Uncertain Significance	F	38	White - British
5974072	78	ABCA4	NM_000350.3	c.3322C>T	p.(Arg1108Cys)	Missense	Pathogenic	M	59	Not stated
5974072	78	ABCA4	NM_000350.3	c.6316C>T	p.(Arg2106Cys)	Missense	Pathogenic	M	59	Not stated
6369516	79	ABCA4	NM_000350.3	c.1906C>T	p.(Gln636Ter)	Stopgain	Pathogenic	M	52	Not stated
6369516	79	ABCA4	NM_000350.3	c.2588G>C	p.(Gly863Ala)	Missense	Pathogenic	M	52	Not stated
6225414	80	ABCA4	NM_000350.3	c.5461-10T>C	Splice	Splice	Pathogenic	F	35	White - British
6225414	80	ABCA4	NM_000350.3	c.5461-10T>C	Splice	Splice	Pathogenic	F	35	White - British
6199654	81	ABCA4	NM_000350.3	c.5882G>A	p.(Gly1961Glu)	Missense	Pathogenic	M	33	White - British
6199654	81	ABCA4	NM_000350.3	c.6118C>T	p.(Arg2040Ter)	Stopgain	Pathogenic	M	33	White - British
4430957	82	ABCA4	NM_000350.3	c.2894A>G	p.(Asn965Ser)	Missense	Pathogenic	F	55	Asian - Indian
4430957	82	ABCA4	NM_000350.3	c.5882G>A	p.(Gly1961Glu)	Missense	Pathogenic	F	55	Asian - Indian
6675906	83	ABCA4	NM_000350.3	c.3289A>T	p.(Arg1097Ter)	Stopgain	Pathogenic	M	66	White - British
6675906	83	ABCA4	NM_000350.3	c.4685T>C	p.(Ile1562Thr)	Missense	Pathogenic	M	66	White - British
3865819	84	ABCA4	NM_000350.3	c.4469G>A	p.(Cys1490Tyr)	Missense	Pathogenic	F	42	White - British
3865819	84	ABCA4	NM_000350.3	c.5882G>A	p.(Gly1961Glu)	Missense	Pathogenic	F	42	White - British
6589008	85	ABCA4	NM_000350.3	c.5461-10T>C	Splice	Splice	Pathogenic	M	33	White - British
6589008	85	ABCA4	NM_000350.3	c.6079C>T	p.(Leu2027Phe)	Missense	Pathogenic	M	33	White - British

6551964	86	ABCA4	NM_000350.3	c.4139C>T	p.(Pro1380Leu)	Missense	Pathogenic	F	33	White - British
6551964	86	ABCA4	NM_000350.3	c.3191-1G>T	Splice	Splice	Likely Pathogenic	F	33	White - British
4378093	87	ABCA4	NM_000350.3	c.5585-1G>A	Splice	Splice	Likely Pathogenic	F	66	White - Other
4378093	87	ABCA4	NM_000350.3	c.5603A>T	p.(Asn1868Ile)	Missense	Variant of Uncertain Significance	F	66	White - Other
6628971	88	ABCA4	NM_000350.3	c.2588G>C	p.(Gly863Ala)	Missense	Pathogenic	M	37	Not stated
6628971	88	ABCA4	NM_000350.3	c.655A>T	p.(Arg219Ter)	Stopgain	Pathogenic	M	37	Not stated
6628971	88	ABCA4	NM_000350.3	c.2828G>A	p.(Arg943Gln)	Missense	Variant of Uncertain Significance	M	37	Not stated
6250243	89	ABCA4	NM_000350.3	c.4469G>A	p.(Cys1490Tyr)	Missense	Pathogenic	M	42	Not stated
6250243	89	ABCA4	NM_000350.3	c.4577C>T	p.(Thr1526Met)	Missense	Pathogenic	M	42	Not stated
6250236	89	ABCA4	NM_000350.3	c.4469G>A	p.(Cys1490Tyr)	Missense	Pathogenic	F	45	Not stated
6250236	89	ABCA4	NM_000350.3	c.4577C>T	p.(Thr1526Met)	Missense	Pathogenic	F	45	Not stated
5136795	90	ABCA4	NM_000350.3	c.2588G>C	p.(Gly863Ala)	Missense	Pathogenic	M	48	White - British
5136795	90	ABCA4	NM_000350.3	c.6098T>C	p.(Leu2033Pro)	Missense	Likely Pathogenic	M	48	White - British
5136795	90	ABCA4	NM_000350.3	c.2828G>A	p.(Arg943Gln)	Missense	Variant of Uncertain Significance	M	48	White - British
6550235	91	ABCA4	NM_000350.3	c.3322C>T	p.(Arg1108Cys)	Missense	Pathogenic	F	71	Any other ethnic group
6550235	91	ABCA4	NM_000350.3	c.5714+5G>A	Splice	Splice	Pathogenic	F	71	Any other ethnic group
6145369	92	ABCA4	NM_000350.3	c.1819G>A	p.(Gly607Arg)	Missense	Pathogenic	F	51	Not stated
6145369	92	ABCA4	NM_000350.3	c.634C>T	p.(Arg212Cys)	Missense	Pathogenic	F	51	Not stated
1013116	93	ABCA4	NM_000350.3	c.2861A>C	p.(Tyr954Ser)	Missense	Pathogenic	F	47	Not stated
1013116	93	ABCA4	NM_000350.3	c.3191-1G>T	Splice	Splice	Likely Pathogenic	F	47	Not stated
6288652	94	ABCA4	NM_000350.3	c.1317G>A	p.(Trp439Ter)	Stopgain	Pathogenic	F	38	Not stated
6288652	94	ABCA4	NM_000350.3	c.2588G>C	p.(Gly863Ala)	Missense	Pathogenic	F	38	Not stated
1243304	95	ABCA4	NM_000350.3	c.1715G>A	p.(Arg572Gln)	Missense	Likely Pathogenic	M	62	White - British
1243304	95	ABCA4	NM_000350.3	c.2588G>C	p.(Gly863Ala)	Missense	Pathogenic	M	62	White - British
1243304	95	ABCA4	NM_000350.3	c.454C>T	p.(Arg152Ter)	Stopgain	Pathogenic	M	62	White - British
1243304	95	ABCA4	NM_000350.3	c.6148G>C	p.(Val2050Leu)	Missense	Variant of Uncertain Significance	M	62	White - British
212946	96	ABCA4	NM_000350.3	c.5461-10T>C	Splice	Splice	Pathogenic	F	73	Not stated
212946	96	ABCA4	NM_000350.3	c.6079C>T	p.(Leu2027Phe)	Missense	Pathogenic	F	73	Not stated
998990	97	ABCA4	NM_000350.3	c.4069G>A	p.(Ala1357Thr)	Missense	Pathogenic	M	57	Not stated
998990	97	ABCA4	NM_000350.3	c.4539+2028C>T	Splice	Splice	Likely Pathogenic	M	57	Not stated
46731	97	ABCA4	NM_000350.3	c.4069G>A	p.(Ala1357Thr)	Missense	Pathogenic	M	58	Not stated
46731	97	ABCA4	NM_000350.3	c.4539+2028C>T	Splice	Splice	Likely Pathogenic	M	58	Not stated
400497	98	ABCA4	NM_000350.3	c.5313-2A>T	Splice	Splice	Likely Pathogenic	M	70	White - British
400497	98	ABCA4	NM_000350.3	c.5882G>A	p.(Gly1961Glu)	Missense	Pathogenic	M	70	White - British
4840709	99	ABCA4	NM_000350.3	c.3259G>A	p.(Glu1087Lys)	Missense	Pathogenic	F	82	Any other ethnic group
4840709	99	ABCA4	NM_000350.3	c.5882G>A	p.(Gly1961Glu)	Missense	Pathogenic	F	82	Any other ethnic group
12171935	100	ABCA4	NM_000350.3	c.1335C>G	p.(Ser445Arg)	Missense	Pathogenic	F	59	White - British
12171935	100	ABCA4	NM_000350.3	c.3322C>T	p.(Arg1108Cys)	Missense	Pathogenic	F	59	White - British
253812	101	ABCA4	NM_000350.3	c.1715G>A	p.(Arg572Gln)	Missense	Likely Pathogenic	F	61	White - British
253812	101	ABCA4	NM_000350.3	c.2588G>C	p.(Gly863Ala)	Missense	Pathogenic	F	61	White - British
253812	101	ABCA4	NM_000350.3	c.5461-10T>C	Splice	Splice	Pathogenic	F	61	White - British
1772231	102	ABCA4	NM_000350.3	c.4253+5G>T	Splice	Splice	Likely Pathogenic	F	83	White - British
1772231	102	ABCA4	NM_000350.3	c.5603A>T	p.(Asn1868Ile)	Missense	Variant of Uncertain Significance	F	83	White - British
565298	103	ABCA4	NM_000350.3	c.2041C>T	p.(Arg681Ter)	Stopgain	Pathogenic	F	76	Any other ethnic group
565298	103	ABCA4	NM_000350.3	c.5714+5G>A	Splice	Splice	Pathogenic	F	76	Any other ethnic group
5754888	104	ABCA4	NM_000350.3	c.4577C>T	p.(Thr1526Met)	Missense	Pathogenic	F	84	Asian - Indian
5754888	104	ABCA4	NM_000350.3	c.6376A>G	p.(Thr2126Ala)	Missense	Likely Pathogenic	F	84	Asian - Indian
135582	105	ABCA4	NM_000350.3	c.3322C>T	p.(Arg1108Cys)	Missense	Pathogenic	F	64	White - British
135582	105	ABCA4	NM_000350.3	c.3322C>T	p.(Arg1108Cys)	Missense	Pathogenic	F	64	White - British
9114818	106	ABCA4	NM_000350.3	c.1433T>C	p.(Ile478Thr)	Missense	Variant of Uncertain Significance	F	59	ed - White and Black Caribbean
9114818	106	ABCA4	NM_000350.3	c.2345G>A	p.(Trp782Ter)	Stopgain	Pathogenic	F	59	ed - White and Black Caribbean

9114818	106	ABCA4	NM_000350.3	c.740A>C	p.(Asn247Thr)	Missense	Likely Pathogenic	F	59	ed - White and Black Caribbean
4024467	106	ABCA4	NM_000350.3	c.2345G>A	p.(Trp782Ter)	Stopgain	Pathogenic	F	79	Black - Caribbean
4024467	106	ABCA4	NM_000350.3	c.6415C>T	p.(Arg2139Trp)	Missense	Pathogenic	F	79	Black - Caribbean
639806	107	ABCA4	NM_000350.3	c.4139C>T	p.(Pro1380Leu)	Missense	Pathogenic	M	63	White - British
639806	107	ABCA4	NM_000350.3	c.5882G>A	p.(Gly1961Glu)	Missense	Pathogenic	M	63	White - British
1822239	108	ABCA4	NM_000350.3	c.6079C>T	p.(Leu2027Phe)	Missense	Pathogenic	F	61	Not stated
1822239	109	ABCA4	NM_000350.3	c.4537dup	p.(Gln1513ProfsTer42)	Frameshift	Pathogenic	F	61	Not stated
545901	110	ABCA4	NM_000350.3	c.161G>A	p.(Cys54Tyr)	Missense	Pathogenic	F	51	White - British
545901	110	ABCA4	NM_000350.3	c.2239del	p.(Leu747CysfsTer40)	Frameshift	Pathogenic	F	51	White - British
3107803	111	ABCA4	NM_000350.3	c.4139C>T	p.(Pro1380Leu)	Missense	Pathogenic	M	62	White - British
3107803	111	ABCA4	NM_000350.3	c.5461-10T>C	Splice	Splice	Pathogenic	M	62	White - British
3028066	112	ABCA4	NM_000350.3	c.3323G>A	p.(Arg1108His)	Missense	Pathogenic	F	50	Not stated
3028066	112	ABCA4	NM_000350.3	c.5461-10T>C	Splice	Splice	Pathogenic	F	50	Not stated
4060755	113	ABCA4	NM_000350.3	c.4537dup	p.(Gln1513ProfsTer42)	Frameshift	Pathogenic	M	42	White - British
4060755	113	ABCA4	NM_000350.3	c.5018+5G>A	Splice	Splice	Pathogenic	M	42	White - British
4106255	114	ABCA4	NM_000350.3	c.1622T>C	p.(Leu541Pro)	Missense	Pathogenic	F	58	White - British
4106255	114	ABCA4	NM_000350.3	c.3113C>T	p.(Ala1038Val)	Missense	Pathogenic	F	58	White - British
4106255	114	ABCA4	NM_000350.3	c.5603A>T	p.(Asn1868Ile)	Missense	Variant of Uncertain Significance	F	58	White - British
5185935	115	ABCA4	NM_000350.3	c.5882G>A	p.(Gly1961Glu)	Missense	Pathogenic	M	43	Black - Other
5185935	115	ABCA4	NM_000350.3	c.5882G>A	p.(Gly1961Glu)	Missense	Pathogenic	M	43	Black - Other
5306993	116	ABCA4	NM_000350.3	c.3210_3211dup	p.(Ser1071CysfsTer14)	Frameshift	Pathogenic	F	74	Not stated
5306993	116	ABCA4	NM_000350.3	c.5714+5G>A	Splice	Splice	Pathogenic	F	74	Not stated
2384983	117	ABCA4	NM_000350.3	c.1335C>G	p.(Ser445Arg)	Missense	Pathogenic	M	46	Not stated
2384983	117	ABCA4	NM_000350.3	c.5161_5162del	p.(Thr1721HisfsTer65)	Frameshift	Pathogenic	M	46	Not stated
4671001	118	ABCA4	NM_000350.3	c.4793C>A	p.(Ala1598Asp)	Missense	Pathogenic	M	62	Not stated
4671001	118	ABCA4	NM_000350.3	c.5714+5G>A	Splice	Splice	Pathogenic	M	62	Not stated
4659178	119	ABCA4	NM_000350.3	c.2966T>C	p.(Val989Ala)	Missense	Pathogenic	F	68	Not stated
4659178	119	ABCA4	NM_000350.3	c.3091A>T	p.(Lys1031Ter)	Stopgain	Pathogenic	F	68	Not stated
5458235	120	ABCA4	NM_000350.3	c.6089G>A	p.(Arg2030Gln)	Missense	Pathogenic	F	76	Not stated
5458235	120	ABCA4	NM_000350.3	c.6118C>T	p.(Arg2040Ter)	Stopgain	Pathogenic	F	76	Not stated
10000584	121	ABCA4	NM_000350.3	c.5461-10T>C	Splice	Splice	Pathogenic	F	53	Not stated
10000584	121	ABCA4	NM_000350.3	c.6320G>A	p.(Arg2107His)	Missense	Pathogenic	F	53	Not stated
10000584	121	ABCA4	NM_000350.3	c.5603A>T	p.(Asn1868Ile)	Missense	Variant of Uncertain Significance	F	53	Not stated
2494715	121	ABCA4	NM_000350.3	c.2791G>A	p.(Val931Met)	Missense	Pathogenic	M	83	Black - Caribbean
2494715	121	ABCA4	NM_000350.3	c.5461-10T>C	Splice	Splice	Pathogenic	M	83	Black - Caribbean
2494715	121	ABCA4	NM_000350.3	c.5603A>T	p.(Asn1868Ile)	Missense	Variant of Uncertain Significance	M	83	Black - Caribbean
5443514	122	ABCA4	NM_000350.3	c.1317G>A	p.(Trp439Ter)	Stopgain	Pathogenic	M	80	White - British
5443514	122	ABCA4	NM_000350.3	c.2588G>C	p.(Gly863Ala)	Missense	Pathogenic	M	80	White - British
4152413	123	ABCA4	NM_000350.3	c.5714+5G>A	Splice	Splice	Pathogenic	F	46	White - British
4152413	123	ABCA4	NM_000350.3	c.5018+2T>C	Splice	Splice	Pathogenic	F	46	White - British
5794718	124	ABCA4	NM_000350.3	c.5714+5G>A	Splice	Splice	Pathogenic	F	40	White - British
5794718	124	ABCA4	NM_000350.3	c.6209C>G	p.(Thr2070Arg)	Missense	Likely Pathogenic	F	40	White - British
730148	124	ABCA4	NM_000350.3	c.5461-10T>C	Splice	Splice	Pathogenic	M	51	Not stated
730148	124	ABCA4	NM_000350.3	c.666_678del	p.(Lys223MetfsTer14)	Frameshift	Pathogenic	M	51	Not stated
3716586	125	ABCA4	NM_000350.3	c.4462T>C	p.(Cys1488Arg)	Missense	Pathogenic	F	41	Asian - Bangladeshi
3716586	125	ABCA4	NM_000350.3	c.4462T>C	p.(Cys1488Arg)	Missense	Pathogenic	F	41	Asian - Bangladeshi
4192635	126	ABCA4	NM_000350.3	c.885del	p.(Leu296CysfsTer4)	Frameshift	Pathogenic	F	38	Asian - Indian
4192635	126	ABCA4	NM_000350.3	c.885del	p.(Leu296CysfsTer4)	Frameshift	Pathogenic	F	38	Asian - Indian
4446000	127	ABCA4	NM_000350.3	c.3322C>T	p.(Arg1108Cys)	Missense	Pathogenic	F	53	White - British
4446000	127	ABCA4	NM_000350.3	c.455G>A	p.(Arg152Gln)	Missense	Variant of Uncertain Significance	F	53	White - British
4446000	127	ABCA4	NM_000350.3	c.5714+5G>A	Splice	Splice	Pathogenic	F	53	White - British

4446000	127	ABCA4	NM_000350.3	c.6320G>A	p.(Arg2107His)	Missense	Pathogenic	F	53	White - British
9101560	128	ABCA4	NM_000350.3	c.5461-10T>C	Splice	Splice	Pathogenic	F	54	Not stated
9101560	128	ABCA4	NM_000350.3	c.4469G>A	p.(Cys1490Tyr)	Missense	Pathogenic	F	54	Not stated
6563423	129	ABCA4	NM_000350.3	c.5882G>A	p.(Gly1961Glu)	Missense	Pathogenic	M	35	White - British
6563423	129	ABCA4	NM_000350.3	c.885del	p.(Leu296CysfsTer4)	Frameshift	Pathogenic	M	35	White - British
1688315	130	ABCA4	NM_000350.3	c.5461-10T>C	Splice	Splice	Pathogenic	M	46	White - British
1688315	130	ABCA4	NM_000350.3	c.768G>T	p.(Val256Val)	Synonymous	Pathogenic	M	46	White - British
6593292	131	ABCA4	NM_000350.3	c.5461-10T>C	Splice	Splice	Pathogenic	F	56	White - British
6593292	131	ABCA4	NM_000350.3	c.5882G>A	p.(Gly1961Glu)	Missense	Pathogenic	F	56	White - British
6187803	132	ABCA4	NM_000350.3	c.5882G>A	p.(Gly1961Glu)	Missense	Pathogenic	F	65	White - British
6187803	132	ABCA4	NM_000350.3	c.5882G>A	p.(Gly1961Glu)	Missense	Pathogenic	F	65	White - British
6805448	133	ABCA4	NM_000350.3	c.2971G>C	p.(Gly991Arg)	Missense	Pathogenic	M	51	Any other ethnic group
6805448	133	ABCA4	NM_000350.3	c.4538A>G	p.(Gln1513Arg)	Missense	Pathogenic	M	51	Any other ethnic group
6826182	134	ABCA4	NM_000350.3	c.2588G>C	p.(Gly863Ala)	Missense	Pathogenic	F	75	White - British
6826182	134	ABCA4	NM_000350.3	c.5461-10T>C	Splice	Splice	Pathogenic	F	75	White - British
1535575	135	ABCA4	NM_000350.3	c.5899-3_5899-2del	Splice	Splice	Likely Pathogenic	F	66	White - British
1535575	135	ABCA4	NM_000350.3	c.6709A>C	p.(Thr2237Pro)	Missense	Variant of Uncertain Significance	F	66	White - British
1535575	135	ABCA4	NM_000350.3	c.5603A>T	p.(Asn1868Ile)	Missense	Variant of Uncertain Significance	F	66	White - British
6805532	136	ABCA4	NM_000350.3	c.1957C>T	p.(Arg653Cys)	Missense	Pathogenic	M	67	Any other ethnic group
6805532	136	ABCA4	NM_000350.3	c.5693G>A	p.(Arg1898His)	Missense	Likely Pathogenic	M	67	Any other ethnic group
6736358	137	ABCA4	NM_000350.3	c.4685T>C	p.(Ile1562Thr)	Missense	Pathogenic	M	85	Not stated
6736358	137	ABCA4	NM_000350.3	c.5461-10T>C	Splice	Splice	Pathogenic	M	85	Not stated
6668213	138	ABCA4	NM_000350.3	c.1906C>T	p.(Gln636Ter)	Stopgain	Pathogenic	F	40	White - British
6668213	138	ABCA4	NM_000350.3	c.5196+1137G>A	Splice	Splice	Pathogenic	F	40	White - British
3265212	139	ABCA4	NM_000350.3	c.1622T>C	p.(Leu541Pro)	Missense	Pathogenic	M	82	Any other ethnic group
3265212	139	ABCA4	NM_000350.3	c.3113C>T	p.(Ala1038Val)	Missense	Pathogenic	M	82	Any other ethnic group
3265212	139	ABCA4	NM_000350.3	c.3758C>T	p.(Thr1253Met)	Missense	Variant of Uncertain Significance	M	82	Any other ethnic group
3265212	139	ABCA4	NM_000350.3	c.5882G>A	p.(Gly1961Glu)	Missense	Pathogenic	M	82	Any other ethnic group
2230780	140	ABCA4	NM_000350.3	c.4462T>C	p.(Cys1488Arg)	Missense	Pathogenic	M	47	Asian - Bangladeshi
2230780	140	ABCA4	NM_000350.3	c.4462T>C	p.(Cys1488Arg)	Missense	Pathogenic	M	47	Asian - Bangladeshi
6973847	141	ABCA4	NM_000350.3	c.1648G>A	p.(Gly550Arg)	Missense	Pathogenic	M	33	White - British
6973847	141	ABCA4	NM_000350.3	c.6449G>A	p.(Cys2150Tyr)	Missense	Pathogenic	M	33	White - British
696163	142	ABCA4	NM_000350.3	c.6658C>T	p.(Gln2220Ter)	Stopgain	Pathogenic	M	59	Not stated
696163	142	ABCA4	NM_000350.3	c.6658C>T	p.(Gln2220Ter)	Stopgain	Pathogenic	M	59	Not stated
3207707	143	ABCA4	NM_000350.3	c.4577C>T	p.(Thr1526Met)	Missense	Pathogenic	M	41	White - British
3207707	143	ABCA4	NM_000350.3	c.5461-10T>C	Splice	Splice	Pathogenic	M	41	White - British
960322	144	ABCA4	NM_000350.3	c.4253+43G>A	Splice	Splice	Likely Pathogenic	F	90	White - British
960322	144	ABCA4	NM_000350.3	c.6005+1G>T	Splice	Splice	Pathogenic	F	90	White - British
6668192	145	ABCA4	NM_000350.3	c.5196+1G>A	Splice	Splice	Pathogenic	F	79	White - British
6668192	145	ABCA4	NM_000350.3	c.5461-10T>C	Splice	Splice	Pathogenic	F	79	White - British
6672077	146	ABCA4	NM_000350.3	c.1957C>T	p.(Arg653Cys)	Missense	Pathogenic	F	54	Not stated
6672077	146	ABCA4	NM_000350.3	c.6089G>A	p.(Arg2030Gln)	Missense	Pathogenic	F	54	Not stated
6969556	146	ABCA4	NM_000350.3	c.1957C>T	p.(Arg653Cys)	Missense	Pathogenic	F	50	Any other ethnic group
6969556	146	ABCA4	NM_000350.3	c.6089G>A	p.(Arg2030Gln)	Missense	Pathogenic	F	50	Any other ethnic group
7041439	147	ABCA4	NM_000350.3	c.1622T>C	p.(Leu541Pro)	Missense	Pathogenic	M	39	White - British
7041439	147	ABCA4	NM_000350.3	c.3113C>T	p.(Ala1038Val)	Missense	Pathogenic	M	39	White - British
7041439	147	ABCA4	NM_000350.3	c.3322C>T	p.(Arg1108Cys)	Missense	Pathogenic	M	39	White - British
1293081	148	ABCA4	NM_000350.3	c.2384G>A	p.(Ser795Asn)	Missense	Likely Pathogenic	F	48	White - British
1293081	148	ABCA4	NM_000350.3	c.5461-10T>C	Splice	Splice	Pathogenic	F	48	White - British
11366410	149	ABCA4	NM_000350.3	c.223T>G	p.(Cys75Gly)	Missense	Pathogenic	F	48	Unknown
11366410	149	ABCA4	NM_000350.3	c.5088C>G	p.(Ser1696Arg)	Missense	Likely Pathogenic	F	48	Unknown

7127882	149	ABCA4	NM_000350.3	c.223T>G	p.(Cys75Gly)	Missense	Pathogenic	F	55	White - British
7127882	149	ABCA4	NM_000350.3	c.5088C>G	p.(Ser1696Arg)	Missense	Likely Pathogenic	F	55	White - British
6955773	150	ABCA4	NM_000350.3	c.4981del	p.(Leu1661Ter)	Stopgain	Pathogenic	F	59	White - Other
6955773	150	ABCA4	NM_000350.3	c.5603A>T	p.(Asn1868Ile)	Missense	Variant of Uncertain Significance	F	59	White - Other
1020221	151	ABCA4	NM_000350.3	c.4685T>C	p.(Ile1562Thr)	Missense	Pathogenic	M	69	Unknown
1020221	151	ABCA4	NM_000350.3	c.5461-10T>C	Splice	Splice	Pathogenic	M	69	Unknown
1020221	151	ABCA4	NM_000350.3	c.5603A>T	p.(Asn1868Ile)	Missense	Variant of Uncertain Significance	M	69	Unknown
6919639	151	ABCA4	NM_000350.3	c.1938-1G>A	Splice	Splice	Pathogenic	F	50	White - British
6919639	151	ABCA4	NM_000350.3	c.5461-10T>C	Splice	Splice	Pathogenic	F	50	White - British
6919639	151	ABCA4	NM_000350.3	c.5603A>T	p.(Asn1868Ile)	Missense	Variant of Uncertain Significance	F	50	White - British
6819140	152	ABCA4	NM_000350.3	c.1805G>A	p.(Arg602Gln)	Missense	Pathogenic	F	76	White - British
6819140	152	ABCA4	NM_000350.3	c.3898C>T	p.(Arg1300Ter)	Stopgain	Pathogenic	F	76	White - British
7187221	153	ABCA4	NM_000350.3	c.4685T>C	p.(Ile1562Thr)	Missense	Pathogenic	M	77	White - British
7187221	153	ABCA4	NM_000350.3	c.4685T>C	p.(Ile1562Thr)	Missense	Pathogenic	M	77	White - British
7201844	154	ABCA4	NM_000350.3	c.5917del	p.(Val1973Ter)	Stopgain	Pathogenic	M	36	Asian - Other
7201844	154	ABCA4	NM_000350.3	c.6658C>T	p.(Gln2220Ter)	Stopgain	Pathogenic	M	36	Asian - Other
4454687	155	ABCA4	NM_000350.3	c.3210_3211dup	p.(Ser1071CysfsTer14)	Frameshift	Pathogenic	F	44	White - British
4454687	155	ABCA4	NM_000350.3	c.4070C>T	p.(Ala1357Val)	Missense	Pathogenic	F	44	White - British
534183	156	ABCA4	NM_000350.3	c.1557C>A	p.(Cys519Ter)	Stopgain	Pathogenic	M	61	White - British
534183	156	ABCA4	NM_000350.3	c.2915C>A	p.(Thr972Asn)	Missense	Pathogenic	M	61	White - British
2545654	157	ABCA4	NM_000350.3	c.3292C>T	p.(Arg1098Cys)	Missense	Pathogenic	F	44	White - British
2545654	157	ABCA4	NM_000350.3	c.3299T>A	p.(Ile1100Asn)	Missense	Pathogenic	F	44	White - British
7362802	158	ABCA4	NM_000350.3	c.3050+1G>C	Splice	Splice	Likely Pathogenic	M	44	Not stated
7362802	158	ABCA4	NM_000350.3	c.5882G>A	p.(Gly1961Glu)	Missense	Pathogenic	M	44	Not stated
3131442	159	ABCA4	NM_000350.3	c.1519G>T	p.(Asp507Tyr)	Missense	Likely Pathogenic	F	46	White - British
3131442	159	ABCA4	NM_000350.3	c.3210_3211dup	p.(Ser1071CysfsTer14)	Frameshift	Pathogenic	F	46	White - British
9598931	160	ABCA4	NM_000350.3	c.4319T>C	p.(Phe1440Ser)	Missense	Likely Pathogenic	M	62	Not stated
9598931	160	ABCA4	NM_000350.3	c.5196+1137G>A	Splice	Splice	Pathogenic	M	62	Not stated
7106042	161	ABCA4	NM_000350.3	c.2588G>C	p.(Gly863Ala)	Missense	Pathogenic	F	42	White - British
7106042	161	ABCA4	NM_000350.3	c.5177C>A	p.(Thr1726Asn)	Missense	Variant of Uncertain Significance	F	42	White - British
2786503	162	ABCA4	NM_000350.3	c.5461-10T>C	Splice	Splice	Pathogenic	F	66	Not stated
2786503	162	ABCA4	NM_000350.3	c.5603A>T	p.(Asn1868Ile)	Missense	Variant of Uncertain Significance	F	66	Not stated
2786503	162	ABCA4	NM_000350.3	c.5603A>T	p.(Asn1868Ile)	Missense	Variant of Uncertain Significance	F	66	Not stated
4884578	163	ABCA4	NM_000350.3	c.2588G>C	p.(Gly863Ala)	Missense	Pathogenic	F	46	White - British
4884578	163	ABCA4	NM_000350.3	c.768G>T	p.(Val256Val)	Synonymous	Pathogenic	F	46	White - British
4884578	163	ABCA4	NM_000350.3	c.2828G>A	p.(Arg943Gln)	Missense	Variant of Uncertain Significance	F	46	White - British
5020350	163	ABCA4	NM_000350.3	c.2588G>C	p.(Gly863Ala)	Missense	Pathogenic	M	42	Not stated
5020350	163	ABCA4	NM_000350.3	c.768G>T	p.(Val256Val)	Synonymous	Pathogenic	M	42	Not stated
5020350	163	ABCA4	NM_000350.3	c.2828G>A	p.(Arg943Gln)	Missense	Variant of Uncertain Significance	M	42	Not stated
5179495	164	ABCA4	NM_000350.3	c.1037A>C	p.(Lys346Thr)	Missense	Variant of Uncertain Significance	M	42	Not stated
5179495	164	ABCA4	NM_000350.3	c.4139C>T	p.(Pro1380Leu)	Missense	Pathogenic	M	42	Not stated
7396451	165	ABCA4	NM_000350.3	c.1817G>A	p.(Gly606Asp)	Missense	Pathogenic	M	53	Not stated
7396451	165	ABCA4	NM_000350.3	c.5714+5G>A	Splice	Splice	Pathogenic	M	53	Not stated
7402709	166	ABCA4	NM_000350.3	c.3398T>C	p.(Ile1133Thr)	Missense	Likely Pathogenic	M	46	Not stated
7402709	166	ABCA4	NM_000350.3	c.4139C>T	p.(Pro1380Leu)	Missense	Pathogenic	M	46	Not stated
7115303	167	ABCA4	NM_000350.3	c.1726G>C	p.(Asp576His)	Missense	Pathogenic	F	44	White - British
7115303	167	ABCA4	NM_000350.3	c.4601del	p.(Leu1534TrpfsTer2)	Frameshift	Likely Pathogenic	F	44	White - British
7769425	168	ABCA4	NM_000350.3	c.5882G>A	p.(Gly1961Glu)	Missense	Pathogenic	F	39	Asian - Pakistani
7769425	168	ABCA4	NM_000350.3	c.5882G>A	p.(Gly1961Glu)	Missense	Pathogenic	F	39	Asian - Pakistani
6264166	169	ABCA4	NM_000350.3	c.2522A>C	p.(Gln841Pro)	Missense	Likely Pathogenic	M	57	Not stated
6264166	169	ABCA4	NM_000350.3	c.2522A>C	p.(Gln841Pro)	Missense	Likely Pathogenic	M	57	Not stated

7578724	170	ABCA4	NM_000350.3	c.3322C>T	p.(Arg1108Cys)	Missense	Pathogenic	M	30	White - British
7578724	170	ABCA4	NM_000350.3	c.6079C>T	p.(Leu2027Phe)	Missense	Pathogenic	M	30	White - British
7820721	171	ABCA4	NM_000350.3	c.3522G>A	p.(Glu1174Glu)	Synonymous	Variant of Uncertain Significance	M	64	White - British
7820721	171	ABCA4	NM_000350.3	c.5527C>G	p.(Arg1843Gly)	Missense	Likely Pathogenic	M	64	White - British
7667869	172	ABCA4	NM_000350.3	c.1622T>C	p.(Leu541Pro)	Missense	Pathogenic	F	52	White - Other
7667869	172	ABCA4	NM_000350.3	c.3113C>T	p.(Ala1038Val)	Missense	Pathogenic	F	52	White - Other
7667869	172	ABCA4	NM_000350.3	c.6089G>A	p.(Arg2030Gln)	Missense	Pathogenic	F	52	White - Other
7804159	173	ABCA4	NM_000350.3	c.6479+1G>A	Splice	Splice	Likely Pathogenic	F	29	Asian - Bangladeshi
7804159	173	ABCA4	NM_000350.3	c.6479+1G>A	Splice	Splice	Likely Pathogenic	F	29	Asian - Bangladeshi
11777128	173	ABCA4	NM_000350.3	c.6479+1G>A	Splice	Splice	Likely Pathogenic	M	21	Any other ethnic group
11777128	173	ABCA4	NM_000350.3	c.6479+1G>A	Splice	Splice	Likely Pathogenic	M	21	Any other ethnic group
1313010	174	ABCA4	NM_000350.3	c.3056C>T	p.(Thr1019Met)	Missense	Pathogenic	F	51	Not stated
1313010	174	ABCA4	NM_000350.3	c.4139C>T	p.(Pro1380Leu)	Missense	Pathogenic	F	51	Not stated
1276680	174	ABCA4	NM_000350.3	c.3056C>T	p.(Thr1019Met)	Missense	Pathogenic	M	54	White - British
1276680	174	ABCA4	NM_000350.3	c.4139C>T	p.(Pro1380Leu)	Missense	Pathogenic	M	54	White - British
7996946	175	ABCA4	NM_000350.3	c.5461-10T>C	Splice	Splice	Pathogenic	F	43	White - British
7996946	175	ABCA4	NM_000350.3	c.5882G>A	p.(Gly1961Glu)	Missense	Pathogenic	F	43	White - British
7770104	176	ABCA4	NM_000350.3	c.4222T>C	p.(Trp1408Arg)	Missense	Pathogenic	M	35	White - British
7770104	176	ABCA4	NM_000350.3	c.4918C>T	p.(Arg1640Trp)	Missense	Pathogenic	M	35	White - British
7770104	176	ABCA4	NM_000350.3	c.6089G>A	p.(Arg2030Gln)	Missense	Pathogenic	M	35	White - British
9210935	176	ABCA4	NM_000350.3	c.4222T>C	p.(Trp1408Arg)	Missense	Pathogenic	M	42	Not stated
9210935	176	ABCA4	NM_000350.3	c.4918C>T	p.(Arg1640Trp)	Missense	Pathogenic	M	42	Not stated
9210935	176	ABCA4	NM_000350.3	c.6089G>A	p.(Arg2030Gln)	Missense	Pathogenic	M	42	Not stated
7252643	177	ABCA4	NM_000350.3	c.2966T>C	p.(Val989Ala)	Missense	Pathogenic	F	37	White - British
7252643	177	ABCA4	NM_000350.3	c.5461-10T>C	Splice	Splice	Pathogenic	F	37	White - British
7408799	178	ABCA4	NM_000350.3	c.1317G>A	p.(Trp439Ter)	Stopgain	Pathogenic	M	83	White - British
7408799	178	ABCA4	NM_000350.3	c.2588G>C	p.(Gly863Ala)	Missense	Pathogenic	M	83	White - British
8117836	179	ABCA4	NM_000350.3	c.4727T>G	p.(Leu1576Arg)	Missense	Likely Pathogenic	M	49	White - British
8117836	179	ABCA4	NM_000350.3	c.768G>T	p.(Val256Val)	Synonymous	Pathogenic	M	49	White - British
4549194	180	ABCA4	NM_000350.3	c.4469G>A	p.(Cys1490Tyr)	Missense	Pathogenic	F	55	White - British
4549194	180	ABCA4	NM_000350.3	c.6449G>A	p.(Cys2150Tyr)	Missense	Pathogenic	F	55	White - British
6989835	181	ABCA4	NM_000350.3	c.2023G>A	p.(Val675Ile)	Missense	Pathogenic	F	61	White - Other
6989835	181	ABCA4	NM_000350.3	c.6329G>A	p.(Trp2110Ter)	Stopgain	Pathogenic	F	61	White - Other
6915684	182	ABCA4	NM_000350.3	c.1019A>C	p.(Tyr340Ser)	Missense	Pathogenic	M	52	Black - Caribbean
6915684	182	ABCA4	NM_000350.3	c.5396A>G	p.(Asn1799Ser)	Missense	Likely Pathogenic	M	52	Black - Caribbean
853852	183	ABCA4	NM_000350.3	c.4139C>T	p.(Pro1380Leu)	Missense	Pathogenic	F	71	Not stated
853852	183	ABCA4	NM_000350.3	c.4462T>C	p.(Cys1488Arg)	Missense	Pathogenic	F	71	Not stated
117977	183	ABCA4	NM_000350.3	c.4139C>T	p.(Pro1380Leu)	Missense	Pathogenic	M	69	Unknown
117977	183	ABCA4	NM_000350.3	c.4462T>C	p.(Cys1488Arg)	Missense	Pathogenic	M	69	Unknown
7177057	184	ABCA4	NM_000350.3	c.6658C>T	p.(Gln2220Ter)	Stopgain	Pathogenic	M	42	Asian - Pakistani
7177057	184	ABCA4	NM_000350.3	c.6658C>T	p.(Gln2220Ter)	Stopgain	Pathogenic	M	42	Asian - Pakistani
2941637	185	ABCA4	NM_000350.3	c.3370G>T	p.(Asp1124Tyr)	Missense	Likely Pathogenic	M	65	White - Irish
2941637	185	ABCA4	NM_000350.3	c.6079C>T	p.(Leu2027Phe)	Missense	Pathogenic	M	65	White - Irish
8447032	186	ABCA4	NM_000350.3	c.2588G>C	p.(Gly863Ala)	Missense	Pathogenic	F	45	Not stated
8447032	186	ABCA4	NM_000350.3	c.4469G>A	p.(Cys1490Tyr)	Missense	Pathogenic	F	45	Not stated
8447032	186	ABCA4	NM_000350.3	c.656G>C	p.(Arg219Thr)	Missense	Likely Pathogenic	F	45	Not stated
8415518	187	ABCA4	NM_000350.3	c.3113C>T	p.(Ala1038Val)	Missense	Pathogenic	F	82	White - British
8415518	187	ABCA4	NM_000350.3	c.6449G>A	p.(Cys2150Tyr)	Missense	Pathogenic	F	82	White - British
8326100	188	ABCA4	NM_000350.3	c.2966T>C	p.(Val989Ala)	Missense	Pathogenic	F	35	Any other ethnic group
8326100	188	ABCA4	NM_000350.3	c.5281_5289del	p.(Pro1761_Leu1763del)	Inframe indel	Variant of Uncertain Significance	F	35	Any other ethnic group
3816539	189	ABCA4	NM_000350.3	c.2971G>C	p.(Gly991Arg)	Missense	Pathogenic	F	67	Not stated

3816539	189	ABCA4	NM_000350.3	c.4537dup	p.(Gln1513ProfsTer42)	Frameshift	Pathogenic	F	67	Not stated
7970192	190	ABCA4	NM_000350.3	c.5018+2T>C	Splice	Splice	Pathogenic	M	35	Any other ethnic group
7970192	190	ABCA4	NM_000350.3	c.5882G>A	p.(Gly1961Glu)	Missense	Pathogenic	M	35	Any other ethnic group
8166052	191	ABCA4	NM_000350.3	c.5461-10T>C	Splice	Splice	Pathogenic	M	40	White - Other
8166052	191	ABCA4	NM_000350.3	c.5882G>A	p.(Gly1961Glu)	Missense	Pathogenic	M	40	White - Other
8390227	192	ABCA4	NM_000350.3	c.2588G>C	p.(Gly863Ala)	Missense	Pathogenic	F	43	White - British
8390227	192	ABCA4	NM_000350.3	c.6148G>C	p.(Val2050Leu)	Missense	Variant of Uncertain Significance	F	43	White - British
3217829	193	ABCA4	NM_000350.3	c.4539+2028C>T	Splice	Splice	Likely Pathogenic	F	69	Not stated
3217829	193	ABCA4	NM_000350.3	c.5461-10T>C	Splice	Splice	Pathogenic	F	69	Not stated
8529275	194	ABCA4	NM_000350.3	c.6729+5_6729+19del	Splice	Splice	Likely Pathogenic	F	44	Mixed - Other
8529275	194	ABCA4	NM_000350.3	c.859-9T>C	Splice	Splice	Likely Pathogenic	F	44	Mixed - Other
8468886	195	ABCA4	NM_000350.3	c.1317G>A	p.(Trp439Ter)	Stopgain	Pathogenic	F	37	White - British
8468886	195	ABCA4	NM_000350.3	c.5882G>A	p.(Gly1961Glu)	Missense	Pathogenic	F	37	White - British
8468886	195	ABCA4	NM_000350.3	c.5908C>T	p.(Leu1970Phe)	Missense	Variant of Uncertain Significance	F	37	White - British
8513329	196	ABCA4	NM_000350.3	c.4070C>A	p.(Ala1357Glu)	Missense	Pathogenic	F	52	Not stated
8513329	196	ABCA4	NM_000350.3	c.4139C>T	p.(Pro1380Leu)	Missense	Pathogenic	F	52	Not stated
3327008	197	ABCA4	NM_000350.3	c.1906C>T	p.(Gln636Ter)	Stopgain	Pathogenic	F	62	White - British
3327008	197	ABCA4	NM_000350.3	c.2588G>C	p.(Gly863Ala)	Missense	Pathogenic	F	62	White - British
8343495	198	ABCA4	NM_000350.3	c.4577C>T	p.(Thr1526Met)	Missense	Pathogenic	M	57	Not stated
8343495	198	ABCA4	NM_000350.3	c.71G>A	p.(Arg24His)	Missense	Pathogenic	M	57	Not stated
7520519	199	ABCA4	NM_000350.3	c.2588G>C	p.(Gly863Ala)	Missense	Pathogenic	M	37	White - British
7520519	199	ABCA4	NM_000350.3	c.5018+2T>C	Splice	Splice	Pathogenic	M	37	White - British
8734431	200	ABCA4	NM_000350.3	c.5461-10T>C	Splice	Splice	Pathogenic	M	28	White - British
8734431	200	ABCA4	NM_000350.3	c.6385A>G	p.(Ser2129Gly)	Missense	Likely Pathogenic	M	28	White - British
8678914	201	ABCA4	NM_000350.3	c.4469G>A	p.(Cys1490Tyr)	Missense	Pathogenic	F	60	Any other ethnic group
8678914	201	ABCA4	NM_000350.3	c.6089G>A	p.(Arg2030Gln)	Missense	Pathogenic	F	60	Any other ethnic group
8322600	202	ABCA4	NM_000350.3	c.3364G>A	p.(Glu1122Lys)	Missense	Pathogenic	F	47	Not stated
8322600	202	ABCA4	NM_000350.3	c.5882G>A	p.(Gly1961Glu)	Missense	Pathogenic	F	47	Not stated
6992229	203	ABCA4	NM_000350.3	c.5882G>A	p.(Gly1961Glu)	Missense	Pathogenic	F	45	White - British
6992229	203	ABCA4	NM_000350.3	c.5899-3_5899-2del	Splice	Splice	Likely Pathogenic	F	45	White - British
4236973	204	ABCA4	NM_000350.3	c.3259G>A	p.(Glu1087Lys)	Missense	Pathogenic	F	71	Not stated
4236973	204	ABCA4	NM_000350.3	c.5882G>A	p.(Gly1961Glu)	Missense	Pathogenic	F	71	Not stated
8521512	205	ABCA4	NM_000350.3	c.4537del	p.(Gln1513ArgfsTer13)	Frameshift	Pathogenic	M	41	White - British
8521512	205	ABCA4	NM_000350.3	c.5882G>A	p.(Gly1961Glu)	Missense	Pathogenic	M	41	White - British
8807000	206	ABCA4	NM_000350.3	c.2588G>C	p.(Gly863Ala)	Missense	Pathogenic	F	42	Not stated
8807000	206	ABCA4	NM_000350.3	c.3210_3211dup	p.(Ser1071CysfsTer14)	Frameshift	Pathogenic	F	42	Not stated
7962625	207	ABCA4	NM_000350.3	c.1222C>T	p.(Arg408Ter)	Stopgain	Pathogenic	M	55	Not stated
7962625	207	ABCA4	NM_000350.3	c.2568C>A	p.(Tyr856Ter)	Stopgain	Likely Pathogenic	M	55	Not stated
8848440	208	ABCA4	NM_000350.3	c.3322C>T	p.(Arg1108Cys)	Missense	Pathogenic	M	27	White - British
8848440	208	ABCA4	NM_000350.3	c.4469G>A	p.(Cys1490Tyr)	Missense	Pathogenic	M	27	White - British
8848440	208	ABCA4	NM_000350.3	c.6320G>A	p.(Arg2107His)	Missense	Pathogenic	M	27	White - British
7782137	209	ABCA4	NM_000350.3	c.1726G>C	p.(Asp576His)	Missense	Pathogenic	M	64	Not stated
7782137	209	ABCA4	NM_000350.3	c.4469G>A	p.(Cys1490Tyr)	Missense	Pathogenic	M	64	Not stated
8761108	210	ABCA4	NM_000350.3	c.4326C>A	p.(Asn1442Lys)	Missense	Likely Pathogenic	F	41	Not stated
8761108	210	ABCA4	NM_000350.3	c.6089G>A	p.(Arg2030Gln)	Missense	Pathogenic	F	41	Not stated
5702479	211	ABCA4	NM_000350.3	c.4352+1G>A	Splice	Splice	Pathogenic	F	56	Not stated
5702479	211	ABCA4	NM_000350.3	c.6320G>A	p.(Arg2107His)	Missense	Pathogenic	F	56	Not stated
4116349	212	ABCA4	NM_000350.3	c.4981del	p.(Leu1661Ter)	Stopgain	Pathogenic	F	52	White - British
4116349	212	ABCA4	NM_000350.3	c.5603A>T	p.(Asn1868Ile)	Missense	Variant of Uncertain Significance	F	52	White - British
7892961	213	ABCA4	NM_000350.3	c.4253+4C>T	Splice	Splice	Likely Pathogenic	M	30	Asian - Indian
7892961	213	ABCA4	NM_000350.3	c.4253+4C>T	Splice	Splice	Likely Pathogenic	M	30	Asian - Indian

8731183	214	ABCA4	NM_000350.3	c.1622T>C	p.(Leu541Pro)	Missense	Pathogenic	F	32	White - Other
8731183	214	ABCA4	NM_000350.3	c.3113C>T	p.(Ala1038Val)	Missense	Pathogenic	F	32	White - Other
8731183	214	ABCA4	NM_000350.3	c.5917del	p.(Val1973Ter)	Stopgain	Pathogenic	F	32	White - Other
4371702	215	ABCA4	NM_000350.3	c.5882G>A	p.(Gly1961Glu)	Missense	Pathogenic	M	59	Asian - Indian
4371702	215	ABCA4	NM_000350.3	c.6712C>T	p.(Gln2238Ter)	Stopgain	Likely Pathogenic	M	59	Asian - Indian
4828788	216	ABCA4	NM_000350.3	c.2588G>C	p.(Gly863Ala)	Missense	Pathogenic	F	64	White - British
4828788	216	ABCA4	NM_000350.3	c.5461-10T>C	Splice	Splice	Pathogenic	F	64	White - British
7975967	217	ABCA4	NM_000350.3	c.1906C>T	p.(Gln636Ter)	Stopgain	Pathogenic	M	48	Not stated
7975967	217	ABCA4	NM_000350.3	c.6079C>T	p.(Leu2027Phe)	Missense	Pathogenic	M	48	Not stated
12415668	218	ABCA4	NM_000350.3	c.3438C>G	p.(Phe1146Leu)	Missense	Variant of Uncertain Significance	M	41	Asian - Other
12415668	218	ABCA4	NM_000350.3	c.93G>A	p.(Trp31Ter)	Stopgain	Pathogenic	M	41	Asian - Other
7953896	219	ABCA4	NM_000350.3	c.1757A>G	p.(Asp586Gly)	Missense	Likely Pathogenic	M	34	Asian - Other
7953896	219	ABCA4	NM_000350.3	c.5882G>A	p.(Gly1961Glu)	Missense	Pathogenic	M	34	Asian - Other
8806937	220	ABCA4	NM_000350.3	c.1648G>T	p.(Gly550Ter)	Stopgain	Likely Pathogenic	M	49	Not stated
8806937	220	ABCA4	NM_000350.3	c.5461-10T>C	Splice	Splice	Pathogenic	M	49	Not stated
7777111	221	ABCA4	NM_000350.3	c.2588G>C	p.(Gly863Ala)	Missense	Pathogenic	F	33	White - British
7777111	221	ABCA4	NM_000350.3	c.4469G>A	p.(Cys1490Tyr)	Missense	Pathogenic	F	33	White - British
8081009	222	ABCA4	NM_000350.3	c.6079C>T	p.(Leu2027Phe)	Missense	Pathogenic	M	35	Not stated
8081009	222	ABCA4	NM_000350.3	c.6148G>C	p.(Val2050Leu)	Missense	Variant of Uncertain Significance	M	35	Not stated
5902105	223	ABCA4	NM_000350.3	c.161G>A	p.(Cys54Tyr)	Missense	Pathogenic	M	36	Not stated
5902105	223	ABCA4	NM_000350.3	c.4222T>C	p.(Trp1408Arg)	Missense	Pathogenic	M	36	Not stated
5902105	223	ABCA4	NM_000350.3	c.4918C>T	p.(Arg1640Trp)	Missense	Pathogenic	M	36	Not stated
8823338	224	ABCA4	NM_000350.3	c.6320G>A	p.(Arg2107His)	Missense	Pathogenic	M	83	Any other ethnic group
8823338	224	ABCA4	NM_000350.3	c.768G>T	p.(Val256Val)	Synonymous	Pathogenic	M	83	Any other ethnic group
2079006	225	ABCA4	NM_000350.3	c.5196+1G>A	Splice	Splice	Pathogenic	M	54	Not stated
2079006	225	ABCA4	NM_000350.3	c.6079C>T	p.(Leu2027Phe)	Missense	Pathogenic	M	54	Not stated
126804	226	ABCA4	NM_000350.3	c.1819G>A	p.(Gly607Arg)	Missense	Pathogenic	M	85	xed - White and Black African
126804	226	ABCA4	NM_000350.3	c.2966T>C	p.(Val989Ala)	Missense	Pathogenic	M	85	xed - White and Black African
6254793	227	ABCA4	NM_000350.3	c.1648G>T	p.(Gly550Ter)	Stopgain	Likely Pathogenic	F	47	Not stated
6254793	227	ABCA4	NM_000350.3	c.3608G>A	p.(Gly1203Glu)	Missense	Likely Pathogenic	F	47	Not stated
5585411	228	ABCA4	NM_000350.3	c.4577C>T	p.(Thr1526Met)	Missense	Pathogenic	F	78	White - British
5585411	228	ABCA4	NM_000350.3	c.5463G>A	p.(Thr1821Thr)	Synonymous	Likely Pathogenic	F	78	White - British
8798019	229	ABCA4	NM_000350.3	c.5882G>A	p.(Gly1961Glu)	Missense	Pathogenic	M	43	Not stated
8798019	229	ABCA4	NM_000350.3	c.6449G>A	p.(Cys2150Tyr)	Missense	Pathogenic	M	43	Not stated
4032727	230	ABCA4	NM_000350.3	c.4222T>C	p.(Trp1408Arg)	Missense	Pathogenic	M	40	White - British
4032727	230	ABCA4	NM_000350.3	c.4918C>T	p.(Arg1640Trp)	Missense	Pathogenic	M	40	White - British
4032727	230	ABCA4	NM_000350.3	c.4957G>A	p.(Gly1653Arg)	Missense	Likely Pathogenic	M	40	White - British
9049795	231	ABCA4	NM_000350.3	c.3305A>T	p.(Asp1102Val)	Missense	Likely Pathogenic	M	39	White - British
9049795	231	ABCA4	NM_000350.3	c.5714+5G>A	Splice	Splice	Pathogenic	M	39	White - British
8785860	232	ABCA4	NM_000350.3	c.2588G>C	p.(Gly863Ala)	Missense	Pathogenic	M	42	White - British
8785860	232	ABCA4	NM_000350.3	c.4577C>T	p.(Thr1526Met)	Missense	Pathogenic	M	42	White - British
8956779	233	ABCA4	NM_000350.3	c.3056C>T	p.(Thr1019Met)	Missense	Pathogenic	F	31	White - Other
8956779	233	ABCA4	NM_000350.3	c.5882G>A	p.(Gly1961Glu)	Missense	Pathogenic	F	31	White - Other
9818962	234	ABCA4	NM_000350.3	c.180del	p.(Met61CysfsTer17)	Frameshift	Likely Pathogenic	M	35	White - British
9818962	234	ABCA4	NM_000350.3	c.4328G>A	p.(Arg1443His)	Missense	Pathogenic	M	35	White - British
7150114	234	ABCA4	NM_000350.3	c.180del	p.(Met61CysfsTer17)	Frameshift	Likely Pathogenic	M	38	White - British
7150114	234	ABCA4	NM_000350.3	c.4328G>A	p.(Arg1443His)	Missense	Pathogenic	M	38	White - British
9127614	235	ABCA4	NM_000350.3	c.4216C>T	p.(His1406Tyr)	Missense	Pathogenic	M	27	White - British
9127614	235	ABCA4	NM_000350.3	c.4222T>C	p.(Trp1408Arg)	Missense	Pathogenic	M	27	White - British
9127614	235	ABCA4	NM_000350.3	c.4918C>T	p.(Arg1640Trp)	Missense	Pathogenic	M	27	White - British
8884931	236	ABCA4	NM_000350.3	c.1140T>A	p.(Asn380Lys)	Missense	Likely Pathogenic	F	50	Not stated

8884931	236	ABCA4	NM_000350.3	c.5882G>A	p.(Gly1961Glu)	Missense	Pathogenic	F	50	Not stated
6525392	237	ABCA4	NM_000350.3	c.1015T>G	p.(Trp339Gly)	Missense	Pathogenic	F	62	White - British
6525392	237	ABCA4	NM_000350.3	c.6089G>A	p.(Arg2030Gln)	Missense	Pathogenic	F	62	White - British
8972319	238	ABCA4	NM_000350.3	c.3259G>A	p.(Glu1087Lys)	Missense	Pathogenic	M	24	White - British
8972319	238	ABCA4	NM_000350.3	c.5461-10T>C	Splice	Splice	Pathogenic	M	24	White - British
1108512	239	ABCA4	NM_000350.3	c.1140T>A	p.(Asn380Lys)	Missense	Likely Pathogenic	M	68	White - Other
1108512	239	ABCA4	NM_000350.3	c.4771G>A	p.(Gly1591Arg)	Missense	Variant of Uncertain Significance	M	68	White - Other
1108512	239	ABCA4	NM_000350.3	c.1643G>A	p.(Trp548Ter)	Stopgain	Pathogenic	M	68	White - Other
5640578	240	ABCA4	NM_000350.3	c.3081T>G	p.(Tyr1027Ter)	Stopgain	Pathogenic	F	35	Asian - Pakistani
5640578	240	ABCA4	NM_000350.3	c.3081T>G	p.(Tyr1027Ter)	Stopgain	Pathogenic	F	35	Asian - Pakistani
3330865	241	ABCA4	NM_000350.3	c.2588G>C	p.(Gly863Ala)	Missense	Pathogenic	M	67	White - Irish
3330865	241	ABCA4	NM_000350.3	c.5461-10T>C	Splice	Splice	Pathogenic	M	67	White - Irish
7989211	242	ABCA4	NM_000350.3	c.1018T>C	p.(Tyr340His)	Missense	Pathogenic	M	55	White - British
7989211	242	ABCA4	NM_000350.3	c.5315G>A	p.(Trp1772Ter)	Stopgain	Pathogenic	M	55	White - British
2668665	243	ABCA4	NM_000350.3	c.5461-10T>C	Splice	Splice	Pathogenic	M	77	Not stated
2668665	243	ABCA4	NM_000350.3	c.5461-10T>C	Splice	Splice	Pathogenic	M	77	Not stated
7921885	244	ABCA4	NM_000350.3	c.4552A>C	p.(Ser1518Arg)	Missense	Variant of Uncertain Significance	M	33	Not stated
7921885	244	ABCA4	NM_000350.3	c.5882G>A	p.(Gly1961Glu)	Missense	Pathogenic	M	33	Not stated
9102456	245	ABCA4	NM_000350.3	c.1622T>C	p.(Leu541Pro)	Missense	Pathogenic	F	48	White - Other
9102456	245	ABCA4	NM_000350.3	c.3113C>T	p.(Ala1038Val)	Missense	Pathogenic	F	48	White - Other
9102456	245	ABCA4	NM_000350.3	c.5882G>A	p.(Gly1961Glu)	Missense	Pathogenic	F	48	White - Other
4964742	246	ABCA4	NM_000350.3	c.4139C>T	p.(Pro1380Leu)	Missense	Pathogenic	F	75	White - British
4964742	246	ABCA4	NM_000350.3	c.5882G>A	p.(Gly1961Glu)	Missense	Pathogenic	F	75	White - British
8460080	247	ABCA4	NM_000350.3	c.5222_5232del	p.(Leu1738GlyfsTer45)	Frameshift	Pathogenic	F	58	Not stated
8460080	247	ABCA4	NM_000350.3	c.5882G>A	p.(Gly1961Glu)	Missense	Pathogenic	F	58	Not stated
5630519	248	ABCA4	NM_000350.3	c.859-9T>C	Splice	Splice	Likely Pathogenic	F	67	Asian - Bangladeshi
5630519	248	ABCA4	NM_000350.3	c.859-9T>C	Splice	Splice	Likely Pathogenic	F	67	Asian - Bangladeshi
7906814	249	ABCA4	NM_000350.3	c.3299T>A	p.(Ile1100Asn)	Missense	Pathogenic	M	33	Not stated
7906814	249	ABCA4	NM_000350.3	c.5461-10T>C	Splice	Splice	Pathogenic	M	33	Not stated
9191426	250	ABCA4	NM_000350.3	c.3064G>A	p.(Glu1022Lys)	Missense	Pathogenic	M	46	Not stated
9191426	250	ABCA4	NM_000350.3	c.6729+5_6729+19del	Splice	Splice	Likely Pathogenic	M	46	Not stated
9241476	251	ABCA4	NM_000350.3	c.5461-10T>C	Splice	Splice	Pathogenic	M	75	Not stated
9241476	251	ABCA4	NM_000350.3	c.6079C>T	p.(Leu2027Phe)	Missense	Pathogenic	M	75	Not stated
9384136	252	ABCA4	NM_000350.3	c.5461-10T>C	Splice	Splice	Pathogenic	F	25	White - British
9384136	252	ABCA4	NM_000350.3	c.634C>T	p.(Arg212Cys)	Missense	Pathogenic	F	25	White - British
9420907	253	ABCA4	NM_000350.3	c.3329-1G>A	Splice	Splice	Pathogenic	F	55	White - British
9420907	253	ABCA4	NM_000350.3	c.5196+1137G>A	Splice	Splice	Pathogenic	F	55	White - British
8806839	254	ABCA4	NM_000350.3	c.2942C>T	p.(Pro981Leu)	Missense	Pathogenic	F	31	White - British
8806839	254	ABCA4	NM_000350.3	c.3208_3209insGT	p.(Ser1071fsTer15)	Frameshift	Likely Pathogenic	F	31	White - British
8806839	254	ABCA4	NM_000350.3	c.6529G>A	p.(Asp2177Asn)	Missense	Variant of Uncertain Significance	F	31	White - British
9459799	255	ABCA4	NM_000350.3	c.4139C>T	p.(Pro1380Leu)	Missense	Pathogenic	M	23	White - British
9459799	255	ABCA4	NM_000350.3	c.768G>T	p.(Val256Val)	Synonymous	Pathogenic	M	23	White - British
9343781	256	ABCA4	NM_000350.3	c.2713del	p.(Glu905ArgfsTer27)	Frameshift	Pathogenic	M	24	Not stated
9343781	256	ABCA4	NM_000350.3	c.2713del	p.(Glu905ArgfsTer27)	Frameshift	Pathogenic	M	24	Not stated
9209934	257	ABCA4	NM_000350.3	c.2588G>C	p.(Gly863Ala)	Missense	Pathogenic	F	47	Not stated
9209934	257	ABCA4	NM_000350.3	c.658C>T	p.(Arg220Cys)	Missense	Likely Pathogenic	F	47	Not stated
9351971	258	ABCA4	NM_000350.3	c.5461-10T>C	Splice	Splice	Pathogenic	M	41	Not stated
9351971	258	ABCA4	NM_000350.3	c.5882G>A	p.(Gly1961Glu)	Missense	Pathogenic	M	41	Not stated
8389821	259	ABCA4	NM_000350.3	c.4222T>C	p.(Trp1408Arg)	Missense	Pathogenic	M	81	White - British
8389821	259	ABCA4	NM_000350.3	c.4918C>T	p.(Arg1640Trp)	Missense	Pathogenic	M	81	White - British
8389821	259	ABCA4	NM_000350.3	c.5603A>T	p.(Asn1868Ile)	Missense	Variant of Uncertain Significance	M	81	White - British

6199696	260	ABCA4	NM_000350.3	c.2510T>C	p.(Leu837Pro)	Missense	Likely Pathogenic	M	47	Not stated
6199696	260	ABCA4	NM_000350.3	c.6320G>A	p.(Arg2107His)	Missense	Pathogenic	M	47	Not stated
9578666	261	ABCA4	NM_000350.3	c.3259G>A	p.(Glu1087Lys)	Missense	Pathogenic	M	72	Not stated
9578666	261	ABCA4	NM_000350.3	c.5882G>A	p.(Gly1961Glu)	Missense	Pathogenic	M	72	Not stated
18209876	262	ABCA4	NM_000350.3	c.2588G>C	p.(Gly863Ala)	Missense	Pathogenic	M	20	Not stated
18209876	262	ABCA4	NM_000350.3	c.2894A>G	p.(Asn965Ser)	Missense	Pathogenic	M	20	Not stated
9505992	263	ABCA4	NM_000350.3	c.5461-10T>C	Splice	Splice	Pathogenic	F	56	White - British
9505992	263	ABCA4	NM_000350.3	c.5603A>T	p.(Asn1868Ile)	Missense	Variant of Uncertain Significance	F	56	White - British
9505992	263	ABCA4	NM_000350.3	c.5603A>T	p.(Asn1868Ile)	Missense	Variant of Uncertain Significance	F	56	White - British
9089863	264	ABCA4	NM_000350.3	c.2588G>C	p.(Gly863Ala)	Missense	Pathogenic	F	39	White - British
9089863	264	ABCA4	NM_000350.3	c.5461-10T>C	Splice	Splice	Pathogenic	F	39	White - British
1051994	265	ABCA4	NM_000350.3	c.1335C>G	p.(Ser445Arg)	Missense	Pathogenic	F	64	Not stated
1051994	265	ABCA4	NM_000350.3	c.1356+1G>A	Splice	Splice	Likely Pathogenic	F	64	Not stated
2109246	266	ABCA4	NM_000350.3	c.2858T>C	p.(Phe953Ser)	Missense	Likely Pathogenic	M	67	White - British
2109246	266	ABCA4	NM_000350.3	c.5714+5G>A	Splice	Splice	Pathogenic	M	67	White - British
9594528	267	ABCA4	NM_000350.3	c.5882G>A	p.(Gly1961Glu)	Missense	Pathogenic	M	60	Not stated
9594528	267	ABCA4	NM_000350.3	c.5882G>A	p.(Gly1961Glu)	Missense	Pathogenic	M	60	Not stated
9594528	267	ABCA4	NM_000350.3	c.634C>T	p.(Arg212Cys)	Missense	Pathogenic	M	60	Not stated
7657425	268	ABCA4	NM_000350.3	c.3081T>G	p.(Tyr1027Ter)	Stopgain	Pathogenic	M	26	Not stated
7657425	268	ABCA4	NM_000350.3	c.3081T>G	p.(Tyr1027Ter)	Stopgain	Pathogenic	M	26	Not stated
9449390	269	ABCA4	NM_000350.3	c.2588G>C	p.(Gly863Ala)	Missense	Pathogenic	F	28	White - British
9449390	269	ABCA4	NM_000350.3	c.3364G>A	p.(Glu1122Lys)	Missense	Pathogenic	F	28	White - British
9449390	269	ABCA4	NM_000350.3	c.6088C>T	p.(Arg2030Ter)	Stopgain	Pathogenic	F	28	White - British
5915251	270	ABCA4	NM_000350.3	c.2791G>A	p.(Val931Met)	Missense	Pathogenic	F	51	Not stated
5915251	270	ABCA4	NM_000350.3	c.5882G>A	p.(Gly1961Glu)	Missense	Pathogenic	F	51	Not stated
5915251	270	ABCA4	NM_000350.3	c.5114G>A	p.(Arg1705Gln)	Missense	Pathogenic	F	51	Not stated
10137455	271	ABCA4	NM_000350.3	c.4363T>C	p.(Cys1455Arg)	Missense	Pathogenic	F	25	White - British
10137455	271	ABCA4	NM_000350.3	c.768G>T	p.(Val256Val)	Synonymous	Pathogenic	F	25	White - British
10080734	272	ABCA4	NM_000350.3	c.5461-10T>C	Splice	Splice	Pathogenic	M	40	White - British
10080734	272	ABCA4	NM_000350.3	c.6523A>T	p.(Lys2175Ter)	Stopgain	Likely Pathogenic	M	40	White - British
9475479	273	ABCA4	NM_000350.3	c.1834C>T	p.(Gln612Ter)	Stopgain	Pathogenic	F	78	Not stated
9475479	273	ABCA4	NM_000350.3	c.4216C>T	p.(His1406Tyr)	Missense	Pathogenic	F	78	Not stated
10208015	274	ABCA4	NM_000350.3	c.4469G>A	p.(Cys1490Tyr)	Missense	Pathogenic	F	24	White - British
10208015	274	ABCA4	NM_000350.3	c.5461-10T>C	Splice	Splice	Pathogenic	F	24	White - British
13631498	274	ABCA4	NM_000350.3	c.4469G>A	p.(Cys1490Tyr)	Missense	Pathogenic	F	13	Not stated
13631498	274	ABCA4	NM_000350.3	c.5461-10T>C	Splice	Splice	Pathogenic	F	13	Not stated
18461981	274	ABCA4	NM_000350.3	c.1538T>A	p.(Val513Asp)	Missense	Variant of Uncertain Significance	F	15	Not stated
18461981	274	ABCA4	NM_000350.3	c.5461-10T>C	Splice	Splice	Pathogenic	F	15	Not stated
18461981	274	ABCA4	NM_000350.3	c.5603A>T	p.(Asn1868Ile)	Missense	Variant of Uncertain Significance	F	15	Not stated
18461981	274	ABCA4	NM_000350.3	c.5603A>T	p.(Asn1868Ile)	Missense	Variant of Uncertain Significance	F	15	Not stated
9923157	275	ABCA4	NM_000350.3	c.2588G>C	p.(Gly863Ala)	Missense	Pathogenic	M	37	Not stated
9923157	275	ABCA4	NM_000350.3	c.655A>T	p.(Arg219Ter)	Stopgain	Pathogenic	M	37	Not stated
10224521	276	ABCA4	NM_000350.3	c.2861A>C	p.(Tyr954Ser)	Missense	Pathogenic	F	26	White - British
10224521	276	ABCA4	NM_000350.3	c.5461-10T>C	Splice	Splice	Pathogenic	F	26	White - British
10230737	277	ABCA4	NM_000350.3	c.4363T>C	p.(Cys1455Arg)	Missense	Pathogenic	M	70	Not stated
10230737	277	ABCA4	NM_000350.3	c.5461-10T>C	Splice	Splice	Pathogenic	M	70	Not stated
1809772	278	ABCA4	NM_000350.3	c.1906C>T	p.(Gln636Ter)	Stopgain	Pathogenic	F	68	White - Other
1809772	278	ABCA4	NM_000350.3	c.3364G>A	p.(Glu1122Lys)	Missense	Pathogenic	F	68	White - Other
9579940	279	ABCA4	NM_000350.3	c.1906C>T	p.(Gln636Ter)	Stopgain	Pathogenic	M	29	Not stated
9579940	279	ABCA4	NM_000350.3	c.2588G>C	p.(Gly863Ala)	Missense	Pathogenic	M	29	Not stated
10219999	280	ABCA4	NM_000350.3	c.214G>A	p.(Gly72Arg)	Missense	Pathogenic	M	23	Asian - Other

10219999	280	ABCA4	NM_000350.3	c.214G>A	p.(Gly72Arg)	Missense	Pathogenic	M	23	Asian - Other
9965283	281	ABCA4	NM_000350.3	c.1804C>T	p.(Arg602Trp)	Missense	Pathogenic	F	60	Not stated
9965283	281	ABCA4	NM_000350.3	c.6227A>C	p.(Lys2076Thr)	Missense	Likely Pathogenic	F	60	Not stated
10331341	282	ABCA4	NM_000350.3	c.2894A>G	p.(Asn965Ser)	Missense	Pathogenic	F	33	Black - African
10331341	282	ABCA4	NM_000350.3	c.6286G>A	p.(Glu2096Lys)	Missense	Pathogenic	F	33	Black - African
2862754	283	ABCA4	NM_000350.3	c.2588G>C	p.(Gly863Ala)	Missense	Pathogenic	M	43	Unknown
2862754	283	ABCA4	NM_000350.3	c.3233G>A	p.(Gly1078Glu)	Missense	Likely Pathogenic	M	43	Unknown
2862754	283	ABCA4	NM_000350.3	c.6286G>A	p.(Glu2096Lys)	Missense	Pathogenic	M	43	Unknown
10274627	284	ABCA4	NM_000350.3	c.4577C>T	p.(Thr1526Met)	Missense	Pathogenic	M	38	Not stated
10274627	284	ABCA4	NM_000350.3	c.5714+5G>A	Splice	Splice	Pathogenic	M	38	Not stated
12902336	284	ABCA4	NM_000350.3	c.4577C>T	p.(Thr1526Met)	Missense	Pathogenic	M	29	Not stated
12902336	284	ABCA4	NM_000350.3	c.5714+5G>A	Splice	Splice	Pathogenic	M	29	Not stated
10455584	285	ABCA4	NM_000350.3	c.4253+5G>A	Splice	Splice	Likely Pathogenic	M	21	Not stated
10455584	285	ABCA4	NM_000350.3	c.6098T>G	p.(Leu2033Arg)	Missense	Likely Pathogenic	M	21	Not stated
17187820	286	ABCA4	NM_000350.3	c.1222C>T	p.(Arg408Ter)	Stopgain	Pathogenic	M	43	Not stated
17187820	286	ABCA4	NM_000350.3	c.2588G>C	p.(Gly863Ala)	Missense	Pathogenic	M	43	Not stated
18559015	287	ABCA4	NM_000350.3	c.4139C>T	p.(Pro1380Leu)	Missense	Pathogenic	M	23	Not stated
18559015	287	ABCA4	NM_000350.3	c.4594G>A	p.(Asp1532Asn)	Missense	Pathogenic	M	23	Not stated
9623599	288	ABCA4	NM_000350.3	c.4793C>A	p.(Ala1598Asp)	Missense	Pathogenic	M	25	Asian - Pakistani
9623599	288	ABCA4	NM_000350.3	c.4793C>A	p.(Ala1598Asp)	Missense	Pathogenic	M	25	Asian - Pakistani
505196	289	ABCA4	NM_000350.3	c.4139C>T	p.(Pro1380Leu)	Missense	Pathogenic	M	80	Not stated
505196	289	ABCA4	NM_000350.3	c.4363T>C	p.(Cys1455Arg)	Missense	Pathogenic	M	80	Not stated
9080392	290	ABCA4	NM_000350.3	c.4139C>T	p.(Pro1380Leu)	Missense	Pathogenic	F	61	White - British
9080392	290	ABCA4	NM_000350.3	c.4773G>A	p.(Gly1591Gly)	Synonymous	Variant of Uncertain Significance	F	61	White - British
4588926	291	ABCA4	NM_000350.3	c.161G>A	p.(Cys54Tyr)	Missense	Pathogenic	M	60	Not stated
4588926	291	ABCA4	NM_000350.3	c.2588G>C	p.(Gly863Ala)	Missense	Pathogenic	M	60	Not stated
2657955	292	ABCA4	NM_000350.3	c.5461-10T>C	Splice	Splice	Pathogenic	F	57	White - British
2657955	292	ABCA4	NM_000350.3	c.6449G>A	p.(Cys2150Tyr)	Missense	Pathogenic	F	57	White - British
5220410	292	ABCA4	NM_000350.3	c.5461-10T>C	Splice	Splice	Pathogenic	F	57	White - British
5220410	292	ABCA4	NM_000350.3	c.6449G>A	p.(Cys2150Tyr)	Missense	Pathogenic	F	57	White - British
1598575	292	ABCA4	NM_000350.3	c.5461-10T>C	Splice	Splice	Pathogenic	M	60	Unknown
1598575	292	ABCA4	NM_000350.3	c.6449G>A	p.(Cys2150Tyr)	Missense	Pathogenic	M	60	Unknown
5775867	292	ABCA4	NM_000350.3	c.4319T>C	p.(Phe1440Ser)	Missense	Likely Pathogenic	F	58	Not stated
5775867	292	ABCA4	NM_000350.3	c.5281_5289del	p.(Pro1761_Leu1763del)	Inframe indel	Variant of Uncertain Significance	F	58	Not stated
8794239	293	ABCA4	NM_000350.3	c.4926C>G	p.(Ser1642Arg)	Missense	Pathogenic	F	46	White - Other
8794239	293	ABCA4	NM_000350.3	c.5044_5058del	p.(Val1682_Val1686del)	Inframe indel	Pathogenic	F	46	White - Other
8794239	293	ABCA4	NM_000350.3	c.5603A>T	p.(Asn1868Ile)	Missense	Variant of Uncertain Significance	F	46	White - Other
8729741	294	ABCA4	NM_000350.3	c.3322C>T	p.(Arg1108Cys)	Missense	Pathogenic	F	27	White - British
8729741	294	ABCA4	NM_000350.3	c.4577C>T	p.(Thr1526Met)	Missense	Pathogenic	F	27	White - British
5824244	295	ABCA4	NM_000350.3	c.1381A>T	p.(Lys461Ter)	Stopgain	Likely Pathogenic	M	34	Not stated
5824244	295	ABCA4	NM_000350.3	c.4537dup	p.(Gln1513ProfsTer42)	Frameshift	Pathogenic	M	34	Not stated
10709901	296	ABCA4	NM_000350.3	c.3259G>A	p.(Glu1087Lys)	Missense	Pathogenic	M	39	Not stated
10709901	296	ABCA4	NM_000350.3	c.3259G>A	p.(Glu1087Lys)	Missense	Pathogenic	M	39	Not stated
8310889	297	ABCA4	NM_000350.3	c.3210_3211dup	p.(Ser1071CysfsTer14)	Frameshift	Pathogenic	M	61	White - British
8310889	297	ABCA4	NM_000350.3	c.5461-10T>C	Splice	Splice	Pathogenic	M	61	White - British
17782785	298	ABCA4	NM_000350.3	c.3260A>G	p.(Glu1087Gly)	Missense	Pathogenic	F	71	Unknown
17782785	298	ABCA4	NM_000350.3	c.5693G>A	p.(Arg1898His)	Missense	Likely Pathogenic	F	71	Unknown
17782785	298	ABCA4	NM_000350.3	c.5882G>A	p.(Gly1961Glu)	Missense	Pathogenic	F	71	Unknown
8141972	299	ABCA4	NM_000350.3	c.1891G>A	p.(Gly631Arg)	Missense	Pathogenic	F	65	Any other ethnic group
8141972	299	ABCA4	NM_000350.3	c.5882G>A	p.(Gly1961Glu)	Missense	Pathogenic	F	65	Any other ethnic group
10604124	300	ABCA4	NM_000350.3	c.3259G>A	p.(Glu1087Lys)	Missense	Pathogenic	F	50	Any other ethnic group

10604124	300	ABCA4	NM_000350.3	c.3840_3845del	p.(Asp1281_Ser1282del)	Inframe indel	Variant of Uncertain Significance	F	50	Any other ethnic group
10604124	300	ABCA4	NM_000350.3	c.5560G>T	p.(Val1854Leu)	Missense	Likely Pathogenic	F	50	Any other ethnic group
10604124	300	ABCA4	NM_000350.3	c.5882G>A	p.(Gly1961Glu)	Missense	Pathogenic	F	50	Any other ethnic group
9922198	301	ABCA4	NM_000350.3	c.3385C>T	p.(Arg1129Cys)	Missense	Pathogenic	F	33	Not stated
9922198	301	ABCA4	NM_000350.3	c.4469G>A	p.(Cys1490Tyr)	Missense	Pathogenic	F	33	Not stated
10659424	302	ABCA4	NM_000350.3	c.1957C>T	p.(Arg653Cys)	Missense	Pathogenic	M	21	Asian - Pakistani
10659424	302	ABCA4	NM_000350.3	c.6088C>T	p.(Arg2030Ter)	Stopgain	Pathogenic	M	21	Asian - Pakistani
10908785	303	ABCA4	NM_000350.3	c.4363T>C	p.(Cys1455Arg)	Missense	Pathogenic	F	26	Not stated
10908785	303	ABCA4	NM_000350.3	c.5714+5G>A	Splice	Splice	Pathogenic	F	26	Not stated
7018409	304	ABCA4	NM_000350.3	c.3392_3393delinsG	p.(Ala1131GlyfsTer17)	Frameshift	Likely Pathogenic	M	64	Not stated
7018409	304	ABCA4	NM_000350.3	c.5882G>A	p.(Gly1961Glu)	Missense	Pathogenic	M	64	Not stated
10763948	305	ABCA4	NM_000350.3	c.5603A>T	p.(Asn1868Ile)	Missense	Variant of Uncertain Significance	F	60	White - British
10763948	305	ABCA4	NM_000350.3	c.2588G>C	p.(Gly863Ala)	Missense	Pathogenic	F	60	White - British
10763948	305	ABCA4	NM_000350.3	c.4594G>A	p.(Asp1532Asn)	Missense	Pathogenic	F	60	White - British
10763948	305	ABCA4	NM_000350.3	c.5693G>A	p.(Arg1898His)	Missense	Likely Pathogenic	F	60	White - British
10763948	305	ABCA4	NM_000350.3	c.1411G>A	p.(Glu471Lys)	Missense	Likely Pathogenic	F	60	White - British
8747857	306	ABCA4	NM_000350.3	c.5714+5G>A	Splice	Splice	Pathogenic	M	52	Not stated
8747857	306	ABCA4	NM_000350.3	c.5761G>A	p.(Val1921Met)	Missense	Likely Pathogenic	M	52	Not stated
9330425	307	ABCA4	NM_000350.3	c.2588G>C	p.(Gly863Ala)	Missense	Pathogenic	F	47	Not stated
9330425	307	ABCA4	NM_000350.3	c.5281_5289del	p.(Pro1761_Leu1763del)	Inframe indel	Variant of Uncertain Significance	F	47	Not stated
10927349	307	ABCA4	NM_000350.3	c.2588G>C	p.(Gly863Ala)	Missense	Pathogenic	F	53	Unknown
10927349	307	ABCA4	NM_000350.3	c.5281_5289del	p.(Pro1761_Leu1763del)	Inframe indel	Variant of Uncertain Significance	F	53	Unknown
11059376	308	ABCA4	NM_000350.3	c.5318C>T	p.(Ala1773Val)	Missense	Pathogenic	F	25	White - British
11059376	308	ABCA4	NM_000350.3	c.5461-10T>C	Splice	Splice	Pathogenic	F	25	White - British
10718609	309	ABCA4	NM_000350.3	c.454C>T	p.(Arg152Ter)	Stopgain	Pathogenic	F	43	White - Other
10718609	309	ABCA4	NM_000350.3	c.768G>T	p.(Val256Val)	Synonymous	Pathogenic	F	43	White - Other
10980598	310	ABCA4	NM_000350.3	c.5917del	p.(Val1973Ter)	Stopgain	Pathogenic	M	31	White - Other
10980598	310	ABCA4	NM_000350.3	c.5917del	p.(Val1973Ter)	Stopgain	Pathogenic	M	31	White - Other
8373847	311	ABCA4	NM_000350.3	c.6079C>T	p.(Leu2027Phe)	Missense	Pathogenic	F	60	Not stated
8373847	311	ABCA4	NM_000350.3	c.6079C>T	p.(Leu2027Phe)	Missense	Pathogenic	F	60	Not stated
11032678	312	ABCA4	NM_000350.3	c.1317G>A	p.(Trp439Ter)	Stopgain	Pathogenic	F	53	Any other ethnic group
11032678	312	ABCA4	NM_000350.3	c.3758C>T	p.(Thr1253Met)	Missense	Variant of Uncertain Significance	F	53	Any other ethnic group
11032678	312	ABCA4	NM_000350.3	c.5882G>A	p.(Gly1961Glu)	Missense	Pathogenic	F	53	Any other ethnic group
10932725	313	ABCA4	NM_000350.3	c.3808G>T	p.(Glu1270Ter)	Stopgain	Pathogenic	F	30	Not stated
10932725	313	ABCA4	NM_000350.3	c.5882G>A	p.(Gly1961Glu)	Missense	Pathogenic	F	30	Not stated
15699130	313	ABCA4	NM_000350.3	c.3808G>T	p.(Glu1270Ter)	Stopgain	Pathogenic	M	29	White - Other
15699130	313	ABCA4	NM_000350.3	c.5882G>A	p.(Gly1961Glu)	Missense	Pathogenic	M	29	White - Other
11193174	314	ABCA4	NM_000350.3	c.3259G>A	p.(Glu1087Lys)	Missense	Pathogenic	F	37	Unknown
11193174	314	ABCA4	NM_000350.3	c.6089G>A	p.(Arg2030Gln)	Missense	Pathogenic	F	37	Unknown
11258484	315	ABCA4	NM_000350.3	c.2813T>C	p.(Phe938Ser)	Missense	Pathogenic	M	48	xed - White and Black African
11258484	315	ABCA4	NM_000350.3	c.712C>T	p.(Gln238Ter)	Stopgain	Pathogenic	M	48	xed - White and Black African
11091688	316	ABCA4	NM_000350.3	c.2588G>C	p.(Gly863Ala)	Missense	Pathogenic	F	63	Unknown
11091688	316	ABCA4	NM_000350.3	c.5461-10T>C	Splice	Splice	Pathogenic	F	63	Unknown
5404489	317	ABCA4	NM_000350.3	c.2522A>C	p.(Gln841Pro)	Missense	Likely Pathogenic	F	44	Not stated
5404489	317	ABCA4	NM_000350.3	c.93G>A	p.(Trp31Ter)	Stopgain	Pathogenic	F	44	Not stated
7328600	317	ABCA4	NM_000350.3	c.2522A>C	p.(Gln841Pro)	Missense	Likely Pathogenic	M	31	Not stated
7328600	317	ABCA4	NM_000350.3	c.93G>A	p.(Trp31Ter)	Stopgain	Pathogenic	M	31	Not stated
10645900	318	ABCA4	NM_000350.3	c.2588G>C	p.(Gly863Ala)	Missense	Pathogenic	M	24	White - British
10645900	318	ABCA4	NM_000350.3	c.4469G>A	p.(Cys1490Tyr)	Missense	Pathogenic	M	24	White - British
10645900	318	ABCA4	NM_000350.3	c.4685T>C	p.(Ile1562Thr)	Missense	Pathogenic	M	24	White - British
10067161	319	ABCA4	NM_000350.3	c.4139C>T	p.(Pro1380Leu)	Missense	Pathogenic	F	49	White - British

10067161	319	ABCA4	NM_000350.3	c.5196+1137G>A	Splice	Splice	Pathogenic	F	49	White - British
10632684	320	ABCA4	NM_000350.3	c.4139C>T	p.(Pro1380Leu)	Missense	Pathogenic	F	51	White - British
10632684	320	ABCA4	NM_000350.3	c.4594G>A	p.(Asp1532Asn)	Missense	Pathogenic	F	51	White - British
11188323	321	ABCA4	NM_000350.3	c.6658C>T	p.(Gln2220Ter)	Stopgain	Pathogenic	M	23	Not stated
11188323	321	ABCA4	NM_000350.3	c.6658C>T	p.(Gln2220Ter)	Stopgain	Pathogenic	M	23	Not stated
9662141	322	ABCA4	NM_000350.3	c.1957C>T	p.(Arg653Cys)	Missense	Pathogenic	F	67	Not stated
9662141	322	ABCA4	NM_000350.3	c.5882G>A	p.(Gly1961Glu)	Missense	Pathogenic	F	67	Not stated
11141906	323	ABCA4	NM_000350.3	c.3322C>T	p.(Arg1108Cys)	Missense	Pathogenic	F	48	Not stated
11141906	323	ABCA4	NM_000350.3	c.5882G>A	p.(Gly1961Glu)	Missense	Pathogenic	F	48	Not stated
11355777	324	ABCA4	NM_000350.3	c.5018+5G>A	Splice	Splice	Pathogenic	F	34	White - British
11355777	324	ABCA4	NM_000350.3	c.5196+1217C>T	Splice	Splice	Variant of Uncertain Significance	F	34	White - British
11355777	324	ABCA4	NM_000350.3	c.5882G>A	p.(Gly1961Glu)	Missense	Pathogenic	F	34	White - British
11453441	325	ABCA4	NM_000350.3	c.4139C>T	p.(Pro1380Leu)	Missense	Pathogenic	M	66	White - British
11453441	325	ABCA4	NM_000350.3	c.4469G>A	p.(Cys1490Tyr)	Missense	Pathogenic	M	66	White - British
5828787	326	ABCA4	NM_000350.3	c.2588G>C	p.(Gly863Ala)	Missense	Pathogenic	M	48	White - British
5828787	326	ABCA4	NM_000350.3	c.735T>G	p.(Tyr245Ter)	Stopgain	Pathogenic	M	48	White - British
11460854	327	ABCA4	NM_000350.3	c.3481C>T	p.(Arg1161Cys)	Missense	Pathogenic	M	45	Not stated
11460854	327	ABCA4	NM_000350.3	c.3481C>T	p.(Arg1161Cys)	Missense	Pathogenic	M	45	Not stated
11233921	328	ABCA4	NM_000350.3	c.2588G>C	p.(Gly863Ala)	Missense	Pathogenic	M	64	Not stated
11233921	328	ABCA4	NM_000350.3	c.4139C>T	p.(Pro1380Leu)	Missense	Pathogenic	M	64	Not stated
11550377	329	ABCA4	NM_000350.3	c.1957C>T	p.(Arg653Cys)	Missense	Pathogenic	M	28	Not stated
11550377	329	ABCA4	NM_000350.3	c.4139C>T	p.(Pro1380Leu)	Missense	Pathogenic	M	28	Not stated
11654957	330	ABCA4	NM_000350.3	c.1715G>A	p.(Arg572Gln)	Missense	Likely Pathogenic	M	38	Not stated
11654957	330	ABCA4	NM_000350.3	c.2588G>C	p.(Gly863Ala)	Missense	Pathogenic	M	38	Not stated
11654957	330	ABCA4	NM_000350.3	c.5603A>T	p.(Asn1868Ile)	Missense	Variant of Uncertain Significance	M	38	Not stated
11654957	330	ABCA4	NM_000350.3	c.5603A>T	p.(Asn1868Ile)	Missense	Variant of Uncertain Significance	M	38	Not stated
11548676	331	ABCA4	NM_000350.3	c.6729+5_6729+19del	Splice	Splice	Likely Pathogenic	F	21	Not stated
11548676	331	ABCA4	NM_000350.3	c.6729+5_6729+19del	Splice	Splice	Likely Pathogenic	F	21	Not stated
3557665	332	ABCA4	NM_000350.3	c.4253+5G>T	Splice	Splice	Likely Pathogenic	F	56	White - British
3557665	332	ABCA4	NM_000350.3	c.4519G>A	p.(Gly1507Arg)	Missense	Pathogenic	F	56	White - British
7573362	333	ABCA4	NM_000350.3	c.3386G>T	p.(Arg1129Leu)	Missense	Pathogenic	F	43	Not stated
7573362	333	ABCA4	NM_000350.3	c.4722del	p.(Ala1575HisfsTer6)	Frameshift	Likely Pathogenic	F	43	Not stated
4807795	334	ABCA4	NM_000350.3	c.6658C>T	p.(Gln2220Ter)	Stopgain	Pathogenic	F	59	Black - Caribbean
4807795	334	ABCA4	NM_000350.3	c.71G>A	p.(Arg24His)	Missense	Pathogenic	F	59	Black - Caribbean
2750572	335	ABCA4	NM_000350.3	c.5882G>A	p.(Gly1961Glu)	Missense	Pathogenic	F	49	Any other ethnic group
2750572	335	ABCA4	NM_000350.3	c.6658C>T	p.(Gln2220Ter)	Stopgain	Pathogenic	F	49	Any other ethnic group
10275306	336	ABCA4	NM_000350.3	c.4316G>A	p.(Gly1439Asp)	Missense	Pathogenic	F	54	Any other ethnic group
10275306	336	ABCA4	NM_000350.3	c.6089G>A	p.(Arg2030Gln)	Missense	Pathogenic	F	54	Any other ethnic group
11736220	337	ABCA4	NM_000350.3	c.2588G>C	p.(Gly863Ala)	Missense	Pathogenic	M	60	White - British
11736220	337	ABCA4	NM_000350.3	c.6079C>T	p.(Leu2027Phe)	Missense	Pathogenic	M	60	White - British
11736598	338	ABCA4	NM_000350.3	c.4469G>A	p.(Cys1490Tyr)	Missense	Pathogenic	M	47	White - British
11736598	338	ABCA4	NM_000350.3	c.5882G>A	p.(Gly1961Glu)	Missense	Pathogenic	M	47	White - British
11722192	339	ABCA4	NM_000350.3	c.4469G>A	p.(Cys1490Tyr)	Missense	Pathogenic	M	20	Any other ethnic group
11722192	339	ABCA4	NM_000350.3	c.5318C>A	p.(Ala1773Glu)	Missense	Pathogenic	M	20	Any other ethnic group
11815880	340	ABCA4	NM_000350.3	c.2686A>G	p.(Lys896Glu)	Missense	Variant of Uncertain Significance	M	29	Unknown
11815880	340	ABCA4	NM_000350.3	c.5882G>A	p.(Gly1961Glu)	Missense	Pathogenic	M	29	Unknown
11761889	341	ABCA4	NM_000350.3	c.4139C>T	p.(Pro1380Leu)	Missense	Pathogenic	M	37	Any other ethnic group
11761889	341	ABCA4	NM_000350.3	c.5714+5G>A	Splice	Splice	Pathogenic	M	37	Any other ethnic group
11756492	342	ABCA4	NM_000350.3	c.21dup	p.(Gln8ThrfsTer46)	Frameshift	Likely Pathogenic	F	23	White - British
11756492	342	ABCA4	NM_000350.3	c.5461-10T>C	Splice	Splice	Pathogenic	F	23	White - British
3201330	343	ABCA4	NM_000350.3	c.6119G>A	p.(Arg2040Gln)	Missense	Pathogenic	F	46	Any other ethnic group

3201330	343	ABCA4	NM_000350.3	c.834del	p.(Asp279IlefsTer21)	Frameshift	Pathogenic	F	46	Any other ethnic group
3694438	343	ABCA4	NM_000350.3	c.6119G>A	p.(Arg2040Gln)	Missense	Pathogenic	F	45	Not stated
3694438	343	ABCA4	NM_000350.3	c.834del	p.(Asp279IlefsTer21)	Frameshift	Pathogenic	F	45	Not stated
8370956	344	ABCA4	NM_000350.3	c.2966T>C	p.(Val989Ala)	Missense	Pathogenic	F	48	Black - Caribbean
8370956	344	ABCA4	NM_000350.3	c.6286G>A	p.(Glu2096Lys)	Missense	Pathogenic	F	48	Black - Caribbean
11970384	345	ABCA4	NM_000350.3	c.5087G>A	p.(Ser1696Asn)	Missense	Pathogenic	F	45	Not stated
11970384	345	ABCA4	NM_000350.3	c.571-1G>T	Splice	Splice	Pathogenic	F	45	Not stated
12730689	346	ABCA4	NM_000350.3	c.4532C>G	p.(Pro1511Arg)	Missense	Pathogenic	F	41	Not stated
12730689	346	ABCA4	NM_000350.3	c.5578C>T	p.(Arg1860Trp)	Missense	Likely Pathogenic	F	41	Not stated
10385521	346	ABCA4	NM_000350.3	c.4532C>G	p.(Pro1511Arg)	Missense	Pathogenic	M	43	White - British
10385521	346	ABCA4	NM_000350.3	c.5578C>T	p.(Arg1860Trp)	Missense	Likely Pathogenic	M	43	White - British
11202687	347	ABCA4	NM_000350.3	c.1903C>A	p.(Gln635Lys)	Missense	Pathogenic	F	38	White - Other
11202687	347	ABCA4	NM_000350.3	c.5882G>A	p.(Gly1961Glu)	Missense	Pathogenic	F	38	White - Other
12036912	348	ABCA4	NM_000350.3	c.3898C>T	p.(Arg1300Ter)	Stopgain	Pathogenic	M	18	Unknown
12036912	348	ABCA4	NM_000350.3	c.3898C>T	p.(Arg1300Ter)	Stopgain	Pathogenic	M	18	Unknown
10512977	349	ABCA4	NM_000350.3	c.1805G>A	p.(Arg602Gln)	Missense	Pathogenic	M	59	Not stated
10512977	349	ABCA4	NM_000350.3	c.5113C>T	p.(Arg1705Trp)	Missense	Pathogenic	M	59	Not stated
10604467	350	ABCA4	NM_000350.3	c.2588G>C	p.(Gly863Ala)	Missense	Pathogenic	F	50	White - British
10604467	350	ABCA4	NM_000350.3	c.768G>T	p.(Val256Val)	Synonymous	Pathogenic	F	50	White - British
11989501	351	ABCA4	NM_000350.3	c.3322C>T	p.(Arg1108Cys)	Missense	Pathogenic	F	62	Unknown
11989501	351	ABCA4	NM_000350.3	c.6079C>T	p.(Leu2027Phe)	Missense	Pathogenic	F	62	Unknown
11840275	352	ABCA4	NM_000350.3	c.2588G>C	p.(Gly863Ala)	Missense	Pathogenic	F	25	Not stated
11840275	352	ABCA4	NM_000350.3	c.5161_5162del	p.(Thr1721HisfsTer65)	Frameshift	Pathogenic	F	25	Not stated
13491400	352	ABCA4	NM_000350.3	c.2588G>C	p.(Gly863Ala)	Missense	Pathogenic	F	18	Not stated
13491400	352	ABCA4	NM_000350.3	c.5161_5162del	p.(Thr1721HisfsTer65)	Frameshift	Pathogenic	F	18	Not stated
8204034	353	ABCA4	NM_000350.3	c.1196del	p.(Leu399ArgfsTer13)	Frameshift	Likely Pathogenic	F	38	Not stated
8204034	353	ABCA4	NM_000350.3	c.2966T>C	p.(Val989Ala)	Missense	Pathogenic	F	38	Not stated
11878600	354	ABCA4	NM_000350.3	c.454C>T	p.(Arg152Ter)	Stopgain	Pathogenic	M	36	White - Other
11878600	354	ABCA4	NM_000350.3	c.5882G>A	p.(Gly1961Glu)	Missense	Pathogenic	M	36	White - Other
9116694	355	ABCA4	NM_000350.3	c.3064G>A	p.(Glu1022Lys)	Missense	Pathogenic	F	27	Not stated
9116694	355	ABCA4	NM_000350.3	c.3064G>A	p.(Glu1022Lys)	Missense	Pathogenic	F	27	Not stated
6647101	356	ABCA4	NM_000350.3	c.1292G>A	p.(Trp431Ter)	Stopgain	Likely Pathogenic	M	32	Any other ethnic group
6647101	356	ABCA4	NM_000350.3	c.5461-10T>C	Splice	Splice	Pathogenic	M	32	Any other ethnic group
11820311	357	ABCA4	NM_000350.3	c.443-453T>C	Splice	Splice	Variant of Uncertain Significance	M	61	Any other ethnic group
11820311	357	ABCA4	NM_000350.3	c.5603A>T	p.(Asn1868Ile)	Missense	Variant of Uncertain Significance	M	61	Any other ethnic group
11820311	357	ABCA4	NM_000350.3	c.768G>T	p.(Val256Val)	Synonymous	Pathogenic	M	61	Any other ethnic group
12817937	357	ABCA4	NM_000350.3	c.443-453T>C	Splice	Splice	Variant of Uncertain Significance	F	59	Any other ethnic group
12817937	357	ABCA4	NM_000350.3	c.5603A>T	p.(Asn1868Ile)	Missense	Variant of Uncertain Significance	F	59	Any other ethnic group
12817937	357	ABCA4	NM_000350.3	c.768G>T	p.(Val256Val)	Synonymous	Pathogenic	F	59	Any other ethnic group
7661163	358	ABCA4	NM_000350.3	c.3064G>A	p.(Glu1022Lys)	Missense	Pathogenic	F	26	Asian - Indian
7661163	358	ABCA4	NM_000350.3	c.3064G>A	p.(Glu1022Lys)	Missense	Pathogenic	F	26	Asian - Indian
11679996	359	ABCA4	NM_000350.3	c.1804C>T	p.(Arg602Trp)	Missense	Pathogenic	M	29	White - British
11679996	359	ABCA4	NM_000350.3	c.2588G>C	p.(Gly863Ala)	Missense	Pathogenic	M	29	White - British
9004204	360	ABCA4	NM_000350.3	c.3364G>A	p.(Glu1122Lys)	Missense	Pathogenic	F	59	White - Other
9004204	360	ABCA4	NM_000350.3	c.5603A>T	p.(Asn1868Ile)	Missense	Variant of Uncertain Significance	F	59	White - Other
6047362	361	ABCA4	NM_000350.3	c.1253T>C	p.(Phe418Ser)	Missense	Pathogenic	M	54	White - British
6047362	361	ABCA4	NM_000350.3	c.5603A>T	p.(Asn1868Ile)	Missense	Variant of Uncertain Significance	M	54	White - British
12241207	362	ABCA4	NM_000350.3	c.5461-10T>C	Splice	Splice	Pathogenic	F	73	Any other ethnic group
12241207	362	ABCA4	NM_000350.3	c.5603A>T	p.(Asn1868Ile)	Missense	Variant of Uncertain Significance	F	73	Any other ethnic group
12241207	362	ABCA4	NM_000350.3	c.5603A>T	p.(Asn1868Ile)	Missense	Variant of Uncertain Significance	F	73	Any other ethnic group
12166720	363	ABCA4	NM_000350.3	c.4469G>A	p.(Cys1490Tyr)	Missense	Pathogenic	M	34	White - British

12166720	363	ABCA4	NM_000350.3	c.5714+5G>A	Splice	Splice	Pathogenic	M	34	White - British
7909915	364	ABCA4	NM_000350.3	c.5882G>A	p.(Gly1961Glu)	Missense	Pathogenic	F	28	Asian - Indian
7909915	364	ABCA4	NM_000350.3	c.6729+5_6729+19del	Splice	Splice	Likely Pathogenic	F	28	Asian - Indian
11549628	365	ABCA4	NM_000350.3	c.3994C>T	p.(Gln1332Ter)	Stopgain	Pathogenic	F	53	Unknown
11549628	365	ABCA4	NM_000350.3	c.5882G>A	p.(Gly1961Glu)	Missense	Pathogenic	F	53	Unknown
12185725	366	ABCA4	NM_000350.3	c.2588G>C	p.(Gly863Ala)	Missense	Pathogenic	F	32	Not stated
12185725	366	ABCA4	NM_000350.3	c.3210_3211dup	p.(Ser1071CysfsTer14)	Frameshift	Pathogenic	F	32	Not stated
8788478	367	ABCA4	NM_000350.3	c.3113C>T	p.(Ala1038Val)	Missense	Pathogenic	M	55	White - British
8788478	367	ABCA4	NM_000350.3	c.4469G>A	p.(Cys1490Tyr)	Missense	Pathogenic	M	55	White - British
11812849	368	ABCA4	NM_000350.3	c.2813T>C	p.(Phe938Ser)	Missense	Pathogenic	M	68	Black - Caribbean
11812849	368	ABCA4	NM_000350.3	c.286A>G	p.(Asn96Asp)	Missense	Pathogenic	M	68	Black - Caribbean
12223812	369	ABCA4	NM_000350.3	c.1906C>T	p.(Gln636Ter)	Stopgain	Pathogenic	F	76	Unknown
12223812	369	ABCA4	NM_000350.3	c.2588G>C	p.(Gly863Ala)	Missense	Pathogenic	F	76	Unknown
12042393	370	ABCA4	NM_000350.3	c.1069_1075del	p.(Asp357SerfsTer15)	Frameshift	Pathogenic	F	68	Asian - Bangladeshi
12042393	370	ABCA4	NM_000350.3	c.859-9T>C	Splice	Splice	Likely Pathogenic	F	68	Asian - Bangladeshi
12257055	371	ABCA4	NM_000350.3	c.1906C>T	p.(Gln636Ter)	Stopgain	Pathogenic	F	26	Not stated
12257055	371	ABCA4	NM_000350.3	c.6817-2A>C	Splice	Splice	Pathogenic	F	26	Not stated
12329008	372	ABCA4	NM_000350.3	c.1906C>T	p.(Gln636Ter)	Stopgain	Pathogenic	F	22	Unknown
12329008	372	ABCA4	NM_000350.3	c.5461-10T>C	Splice	Splice	Pathogenic	F	22	Unknown
8961063	373	ABCA4	NM_000350.3	c.1804C>T	p.(Arg602Trp)	Missense	Pathogenic	M	49	White - Other
8961063	373	ABCA4	NM_000350.3	c.5196+1137G>A	Splice	Splice	Pathogenic	M	49	White - Other
10506320	374	ABCA4	NM_000350.3	c.4139C>T	p.(Pro1380Leu)	Missense	Pathogenic	F	52	Asian - Indian
10506320	374	ABCA4	NM_000350.3	c.5882G>A	p.(Gly1961Glu)	Missense	Pathogenic	F	52	Asian - Indian
12216525	375	ABCA4	NM_000350.3	c.1609C>T	p.(Arg537Cys)	Missense	Pathogenic	M	53	Black - Caribbean
12216525	375	ABCA4	NM_000350.3	c.6320G>A	p.(Arg2107His)	Missense	Pathogenic	M	53	Black - Caribbean
12216525	375	ABCA4	NM_000350.3	c.926C>G	p.(Pro309Arg)	Missense	Pathogenic	M	53	Black - Caribbean
653974	375	ABCA4	NM_000350.3	c.1609C>T	p.(Arg537Cys)	Missense	Pathogenic	F	88	Black - Caribbean
653974	375	ABCA4	NM_000350.3	c.6320G>A	p.(Arg2107His)	Missense	Pathogenic	F	88	Black - Caribbean
653974	375	ABCA4	NM_000350.3	c.926C>G	p.(Pro309Arg)	Missense	Pathogenic	F	88	Black - Caribbean
9133270	376	ABCA4	NM_000350.3	c.4139C>T	p.(Pro1380Leu)	Missense	Pathogenic	M	60	White - British
9133270	376	ABCA4	NM_000350.3	c.6079C>T	p.(Leu2027Phe)	Missense	Pathogenic	M	60	White - British
11947494	377	ABCA4	NM_000350.3	c.5882G>A	p.(Gly1961Glu)	Missense	Pathogenic	F	68	Any other ethnic group
11947494	377	ABCA4	NM_000350.3	c.93G>A	p.(Trp31Ter)	Stopgain	Pathogenic	F	68	Any other ethnic group
11964763	378	ABCA4	NM_000350.3	c.2560G>A	p.(Ala854Thr)	Missense	Likely Pathogenic	F	50	Not stated
11964763	378	ABCA4	NM_000350.3	c.4469G>A	p.(Cys1490Tyr)	Missense	Pathogenic	F	50	Not stated
12332977	379	ABCA4	NM_000350.3	c.1317G>A	p.(Trp439Ter)	Stopgain	Pathogenic	F	24	White - British
12332977	379	ABCA4	NM_000350.3	c.4139C>T	p.(Pro1380Leu)	Missense	Pathogenic	F	24	White - British
11339516	380	ABCA4	NM_000350.3	c.1317G>A	p.(Trp439Ter)	Stopgain	Pathogenic	M	65	Unknown
11339516	380	ABCA4	NM_000350.3	c.5056G>A	p.(Val1686Met)	Missense	Likely Pathogenic	M	65	Unknown
12182925	381	ABCA4	NM_000350.3	c.1622T>C	p.(Leu541Pro)	Missense	Pathogenic	M	34	Any other ethnic group
12182925	381	ABCA4	NM_000350.3	c.3113C>T	p.(Ala1038Val)	Missense	Pathogenic	M	34	Any other ethnic group
12182925	381	ABCA4	NM_000350.3	c.5714+5G>A	Splice	Splice	Pathogenic	M	34	Any other ethnic group
16266970	381	ABCA4	NM_000350.3	c.1622T>C	p.(Leu541Pro)	Missense	Pathogenic	M	32	Any other ethnic group
16266970	381	ABCA4	NM_000350.3	c.3113C>T	p.(Ala1038Val)	Missense	Pathogenic	M	32	Any other ethnic group
16266970	381	ABCA4	NM_000350.3	c.5714+5G>A	Splice	Splice	Pathogenic	M	32	Any other ethnic group
12365800	382	ABCA4	NM_000350.3	c.1819G>A	p.(Gly607Arg)	Missense	Pathogenic	M	39	White - British
12365800	382	ABCA4	NM_000350.3	c.4253+43G>A	Splice	Splice	Likely Pathogenic	M	39	White - British
12365800	382	ABCA4	NM_000350.3	c.6148G>C	p.(Val2050Leu)	Missense	Variant of Uncertain Significance	M	39	White - British
12008870	383	ABCA4	NM_000350.3	c.4139C>T	p.(Pro1380Leu)	Missense	Pathogenic	M	27	Unknown
12008870	383	ABCA4	NM_000350.3	c.5308T>G	p.(Tyr1770Asp)	Missense	Pathogenic	M	27	Unknown
12335973	384	ABCA4	NM_000350.3	c.1622T>C	p.(Leu541Pro)	Missense	Pathogenic	F	45	Any other ethnic group

12335973	384	ABCA4	NM_000350.3	c.67-2A>G	Splice	Splice	Pathogenic	F	45	Any other ethnic group
12381550	385	ABCA4	NM_000350.3	c.2588G>C	p.(Gly863Ala)	Missense	Pathogenic	F	56	Not stated
12381550	385	ABCA4	NM_000350.3	c.4222T>C	p.(Trp1408Arg)	Missense	Pathogenic	F	56	Not stated
12381550	385	ABCA4	NM_000350.3	c.4918C>T	p.(Arg1640Trp)	Missense	Pathogenic	F	56	Not stated
10032392	386	ABCA4	NM_000350.3	c.4364G>T	p.(Cys1455Phe)	Missense	Likely Pathogenic	F	38	White - British
10032392	386	ABCA4	NM_000350.3	c.5461-10T>C	Splice	Splice	Pathogenic	F	38	White - British
10220797	387	ABCA4	NM_000350.3	c.4537dup	p.(Gln1513ProfsTer42)	Frameshift	Pathogenic	F	54	Unknown
10220797	387	ABCA4	NM_000350.3	c.4539+2028C>T	Splice	Splice	Likely Pathogenic	F	54	Unknown
4486558	388	ABCA4	NM_000350.3	c.4637T>G	p.(Leu1546Ter)	Stopgain	Pathogenic	M	61	Not stated
4486558	388	ABCA4	NM_000350.3	c.5882G>A	p.(Gly1961Glu)	Missense	Pathogenic	M	61	Not stated
18416488	388	ABCA4	NM_000350.3	c.4637T>G	p.(Leu1546Ter)	Stopgain	Pathogenic	M	59	Not stated
18416488	388	ABCA4	NM_000350.3	c.5882G>A	p.(Gly1961Glu)	Missense	Pathogenic	M	59	Not stated
2025589	389	ABCA4	NM_000350.3	c.1906C>T	p.(Gln636Ter)	Stopgain	Pathogenic	M	57	Unknown
2025589	389	ABCA4	NM_000350.3	c.2588G>C	p.(Gly863Ala)	Missense	Pathogenic	M	57	Unknown
12341139	389	ABCA4	NM_000350.3	c.1906C>T	p.(Gln636Ter)	Stopgain	Pathogenic	F	34	White - British
12341139	389	ABCA4	NM_000350.3	c.5882G>A	p.(Gly1961Glu)	Missense	Pathogenic	F	34	White - British
9828223	390	ABCA4	NM_000350.3	c.4463G>T	p.(Cys1488Phe)	Missense	Pathogenic	M	46	Not stated
9828223	390	ABCA4	NM_000350.3	c.6709dup	p.(Thr2237AsnfsTer14)	Frameshift	Likely Pathogenic	M	46	Not stated
11033833	391	ABCA4	NM_000350.3	c.5196+1G>A	Splice	Splice	Pathogenic	F	56	Not stated
11033833	391	ABCA4	NM_000350.3	c.6079C>T	p.(Leu2027Phe)	Missense	Pathogenic	F	56	Not stated
12334293	392	ABCA4	NM_000350.3	c.454C>T	p.(Arg152Ter)	Stopgain	Pathogenic	M	26	Not stated
12334293	392	ABCA4	NM_000350.3	c.5882G>A	p.(Gly1961Glu)	Missense	Pathogenic	M	26	Not stated
15926630	393	ABCA4	NM_000350.3	c.5584G>A	p.(Gly1862Ser)	Missense	Likely Pathogenic	M	57	Not stated
15926630	393	ABCA4	NM_000350.3	c.6320G>A	p.(Arg2107His)	Missense	Pathogenic	M	57	Not stated
12292811	394	ABCA4	NM_000350.3	c.3322C>T	p.(Arg1108Cys)	Missense	Pathogenic	M	21	White - British
12292811	394	ABCA4	NM_000350.3	c.5461-10T>C	Splice	Splice	Pathogenic	M	21	White - British
10242301	395	ABCA4	NM_000350.3	c.3259G>A	p.(Glu1087Lys)	Missense	Pathogenic	F	24	Unknown
10242301	395	ABCA4	NM_000350.3	c.5461-10T>C	Splice	Splice	Pathogenic	F	24	Unknown
12532918	396	ABCA4	NM_000350.3	c.3289A>T	p.(Arg1097Ter)	Stopgain	Pathogenic	M	22	Unknown
12532918	396	ABCA4	NM_000350.3	c.5196+1G>A	Splice	Splice	Pathogenic	M	22	Unknown
8066477	397	ABCA4	NM_000350.3	c.1253T>C	p.(Phe418Ser)	Missense	Pathogenic	F	48	Not stated
8066477	397	ABCA4	NM_000350.3	c.5882G>A	p.(Gly1961Glu)	Missense	Pathogenic	F	48	Not stated
4459622	398	ABCA4	NM_000350.3	c.859-9T>C	Splice	Splice	Likely Pathogenic	F	40	Asian - Bangladeshi
4459622	398	ABCA4	NM_000350.3	c.93G>A	p.(Trp31Ter)	Stopgain	Pathogenic	F	40	Asian - Bangladeshi
12607734	399	ABCA4	NM_000350.3	c.3380G>A	p.(Gly1127Glu)	Missense	Pathogenic	F	26	Unknown
12607734	399	ABCA4	NM_000350.3	c.3380G>A	p.(Gly1127Glu)	Missense	Pathogenic	F	26	Unknown
12607741	399	ABCA4	NM_000350.3	c.3380G>A	p.(Gly1127Glu)	Missense	Pathogenic	F	32	Unknown
12607741	399	ABCA4	NM_000350.3	c.3380G>A	p.(Gly1127Glu)	Missense	Pathogenic	F	32	Unknown
12424138	400	ABCA4	NM_000350.3	c.5461-10T>C	Splice	Splice	Pathogenic	M	79	White - British
12424138	400	ABCA4	NM_000350.3	c.5882G>A	p.(Gly1961Glu)	Missense	Pathogenic	M	79	White - British
11721877	401	ABCA4	NM_000350.3	c.1531C>T	p.(Arg511Cys)	Missense	Pathogenic	F	27	Asian - Indian
11721877	401	ABCA4	NM_000350.3	c.5882G>A	p.(Gly1961Glu)	Missense	Pathogenic	F	27	Asian - Indian
11721884	401	ABCA4	NM_000350.3	c.1531C>T	p.(Arg511Cys)	Missense	Pathogenic	F	27	Asian - Indian
11721884	401	ABCA4	NM_000350.3	c.5882G>A	p.(Gly1961Glu)	Missense	Pathogenic	F	27	Asian - Indian
12002185	402	ABCA4	NM_000350.3	c.4577C>T	p.(Thr1526Met)	Missense	Pathogenic	F	25	White - British
12002185	402	ABCA4	NM_000350.3	c.4577C>T	p.(Thr1526Met)	Missense	Pathogenic	F	25	White - British
11775126	403	ABCA4	NM_000350.3	c.1874_1912del	p.(Gln625_Met637del)	Inframe indel	Likely Pathogenic	F	38	Not stated
11775126	403	ABCA4	NM_000350.3	c.5882G>A	p.(Gly1961Glu)	Missense	Pathogenic	F	38	Not stated
8468949	404	ABCA4	NM_000350.3	c.3210_3211dup	p.(Ser1071CysfsTer14)	Frameshift	Pathogenic	F	44	Not stated
8468949	404	ABCA4	NM_000350.3	c.5714+5G>A	Splice	Splice	Pathogenic	F	44	Not stated
12577340	405	ABCA4	NM_000350.3	c.302+5G>C	Splice	Splice	Variant of Uncertain Significance	M	48	Any other ethnic group

12577340	405	ABCA4	NM_000350.3	c.618C>G	p.(Ser206Arg)	Missense	Variant of Uncertain Significance	M	48	Any other ethnic group
12577340	405	ABCA4	NM_000350.3	c.6320G>A	p.(Arg2107His)	Missense	Pathogenic	M	48	Any other ethnic group
12000883	406	ABCA4	NM_000350.3	c.3259G>T	p.(Glu1087Ter)	Stopgain	Pathogenic	F	28	Not stated
12000883	406	ABCA4	NM_000350.3	c.5882G>A	p.(Gly1961Glu)	Missense	Pathogenic	F	28	Not stated
12637085	407	ABCA4	NM_000350.3	c.2041C>T	p.(Arg681Ter)	Stopgain	Pathogenic	F	29	White - British
12637085	407	ABCA4	NM_000350.3	c.2588G>C	p.(Gly863Ala)	Missense	Pathogenic	F	29	White - British
12618696	408	ABCA4	NM_000350.3	c.2160+2T>G	Splice	Splice	Likely Pathogenic	F	35	Not stated
12618696	408	ABCA4	NM_000350.3	c.5196+1137G>A	Splice	Splice	Pathogenic	F	35	Not stated
9328787	409	ABCA4	NM_000350.3	c.3322C>T	p.(Arg1108Cys)	Missense	Pathogenic	F	42	White - British
9328787	409	ABCA4	NM_000350.3	c.5882G>A	p.(Gly1961Glu)	Missense	Pathogenic	F	42	White - British
12615406	410	ABCA4	NM_000350.3	c.5297T>C	p.(Leu1766Pro)	Missense	Likely Pathogenic	M	20	Black - African
12615406	410	ABCA4	NM_000350.3	c.6119G>A	p.(Arg2040Gln)	Missense	Pathogenic	M	20	Black - African
1769732	411	ABCA4	NM_000350.3	c.5413A>G	p.(Asn1805Asp)	Missense	Pathogenic	F	59	Not stated
1769732	411	ABCA4	NM_000350.3	c.6079C>T	p.(Leu2027Phe)	Missense	Pathogenic	F	59	Not stated
12722604	412	ABCA4	NM_000350.3	c.1622T>C	p.(Leu541Pro)	Missense	Pathogenic	F	23	White - Other
12722604	412	ABCA4	NM_000350.3	c.3113C>T	p.(Ala1038Val)	Missense	Pathogenic	F	23	White - Other
12722604	412	ABCA4	NM_000350.3	c.5714+5G>A	Splice	Splice	Pathogenic	F	23	White - Other
12747328	413	ABCA4	NM_000350.3	c.6658C>T	p.(Gln2220Ter)	Stopgain	Pathogenic	M	30	Not stated
12747328	413	ABCA4	NM_000350.3	c.6658C>T	p.(Gln2220Ter)	Stopgain	Pathogenic	M	30	Not stated
12956999	413	ABCA4	NM_000350.3	c.6658C>T	p.(Gln2220Ter)	Stopgain	Pathogenic	M	20	Asian - Pakistani
12956999	413	ABCA4	NM_000350.3	c.6658C>T	p.(Gln2220Ter)	Stopgain	Pathogenic	M	20	Asian - Pakistani
11521012	414	ABCA4	NM_000350.3	c.1964T>G	p.(Phe655Cys)	Missense	Pathogenic	M	30	Not stated
11521012	414	ABCA4	NM_000350.3	c.5882G>A	p.(Gly1961Glu)	Missense	Pathogenic	M	30	Not stated
11786676	415	ABCA4	NM_000350.3	c.3329-17T>G	Splice	Splice	Variant of Uncertain Significance	F	29	Not stated
11786676	415	ABCA4	NM_000350.3	c.3329-17T>G	Splice	Splice	Variant of Uncertain Significance	F	29	Not stated
11786676	415	ABCA4	NM_000350.3	c.5882G>A	p.(Gly1961Glu)	Missense	Pathogenic	F	29	Not stated
11786676	415	ABCA4	NM_000350.3	c.5882G>A	p.(Gly1961Glu)	Missense	Pathogenic	F	29	Not stated
12646409	416	ABCA4	NM_000350.3	c.6317G>A	p.(Arg2106His)	Missense	Pathogenic	M	44	Asian - Indian
12646409	416	ABCA4	NM_000350.3	c.6317G>A	p.(Arg2106His)	Missense	Pathogenic	M	44	Asian - Indian
12747776	417	ABCA4	NM_000350.3	c.1622T>C	p.(Leu541Pro)	Missense	Pathogenic	M	19	Unknown
12747776	417	ABCA4	NM_000350.3	c.1622T>C	p.(Leu541Pro)	Missense	Pathogenic	M	19	Unknown
12747776	417	ABCA4	NM_000350.3	c.3113C>T	p.(Ala1038Val)	Missense	Pathogenic	M	19	Unknown
12747776	417	ABCA4	NM_000350.3	c.3113C>T	p.(Ala1038Val)	Missense	Pathogenic	M	19	Unknown
11253808	418	ABCA4	NM_000350.3	c.1937+1G>A	Splice	Splice	Pathogenic	M	40	Not stated
11253808	418	ABCA4	NM_000350.3	c.4715C>T	p.(Thr1572Met)	Missense	Variant of Uncertain Significance	M	40	Not stated
11362462	419	ABCA4	NM_000350.3	c.2588G>C	p.(Gly863Ala)	Missense	Pathogenic	F	80	White - British
11362462	419	ABCA4	NM_000350.3	c.5603A>T	p.(Asn1868Ile)	Missense	Variant of Uncertain Significance	F	80	White - British
11362462	419	ABCA4	NM_000350.3	c.5603A>T	p.(Asn1868Ile)	Missense	Variant of Uncertain Significance	F	80	White - British
8960909	420	ABCA4	NM_000350.3	c.719T>G	p.(Ile240Arg)	Missense	Likely Pathogenic	M	26	Not stated
8960909	420	ABCA4	NM_000350.3	c.719T>G	p.(Ile240Arg)	Missense	Likely Pathogenic	M	26	Not stated
10960431	421	ABCA4	NM_000350.3	c.1622T>C	p.(Leu541Pro)	Missense	Pathogenic	M	91	Any other ethnic group
10960431	421	ABCA4	NM_000350.3	c.3364G>A	p.(Glu1122Lys)	Missense	Pathogenic	M	91	Any other ethnic group
7594628	422	ABCA4	NM_000350.3	c.4139C>T	p.(Pro1380Leu)	Missense	Pathogenic	M	49	Not stated
7594628	422	ABCA4	NM_000350.3	c.4469G>A	p.(Cys1490Tyr)	Missense	Pathogenic	M	49	Not stated
12906613	423	ABCA4	NM_000350.3	c.3482G>A	p.(Arg1161His)	Missense	Pathogenic	F	59	Not stated
12906613	423	ABCA4	NM_000350.3	c.4139C>T	p.(Pro1380Leu)	Missense	Pathogenic	F	59	Not stated
12438999	424	ABCA4	NM_000350.3	c.5882G>A	p.(Gly1961Glu)	Missense	Pathogenic	M	76	Any other ethnic group
12438999	424	ABCA4	NM_000350.3	c.6729+5_6729+19del	Splice	Splice	Likely Pathogenic	M	76	Any other ethnic group
12934018	425	ABCA4	NM_000350.3	c.2300T>A	p.(Val767Asp)	Missense	Pathogenic	F	23	White - British
12934018	425	ABCA4	NM_000350.3	c.4793C>A	p.(Ala1598Asp)	Missense	Pathogenic	F	23	White - British
12871998	426	ABCA4	NM_000350.3	c.6658C>T	p.(Gln2220Ter)	Stopgain	Pathogenic	M	17	Not stated

12871998	426	ABCA4	NM_000350.3	c.6658C>T	p.(Gln2220Ter)	Stopgain	Pathogenic	M	17	Not stated
12883107	427	ABCA4	NM_000350.3	c.3113C>T	p.(Ala1038Val)	Missense	Pathogenic	F	52	Not stated
12883107	427	ABCA4	NM_000350.3	c.5645T>C	p.(Met1882Thr)	Missense	Likely Pathogenic	F	52	Not stated
11314218	428	ABCA4	NM_000350.3	c.5714+5G>A	Splice	Splice	Pathogenic	M	37	Any other ethnic group
11314218	428	ABCA4	NM_000350.3	c.6658C>T	p.(Gln2220Ter)	Stopgain	Pathogenic	M	37	Any other ethnic group
8053702	429	ABCA4	NM_000350.3	c.1253T>C	p.(Phe418Ser)	Missense	Pathogenic	F	81	White - British
8053702	429	ABCA4	NM_000350.3	c.5882G>A	p.(Gly1961Glu)	Missense	Pathogenic	F	81	White - British
8974384	430	ABCA4	NM_000350.3	c.5882G>A	p.(Gly1961Glu)	Missense	Pathogenic	M	25	White - British
8974384	430	ABCA4	NM_000350.3	c.6729+5_6729+19del	Splice	Splice	Likely Pathogenic	M	25	White - British
12826582	431	ABCA4	NM_000350.3	c.3364G>A	p.(Glu1122Lys)	Missense	Pathogenic	F	26	Any other ethnic group
12826582	431	ABCA4	NM_000350.3	c.5196+1137G>A	Splice	Splice	Pathogenic	F	26	Any other ethnic group
12957720	432	ABCA4	NM_000350.3	c.4537del	p.(Gln1513ArgfsTer13)	Frameshift	Pathogenic	F	56	White - British
12957720	432	ABCA4	NM_000350.3	c.5714+5G>A	Splice	Splice	Pathogenic	F	56	White - British
9349269	433	ABCA4	NM_000350.3	c.1715G>A	p.(Arg572Gln)	Missense	Likely Pathogenic	F	56	White - British
9349269	433	ABCA4	NM_000350.3	c.2588G>A	p.(Gly863Ala)	Missense	Pathogenic	F	56	White - British
9349269	433	ABCA4	NM_000350.3	c.5196+1137G>A	Splice	Splice	Pathogenic	F	56	White - British
12497253	434	ABCA4	NM_000350.3	c.4469G>A	p.(Cys1490Tyr)	Missense	Pathogenic	F	21	White - British
12497253	434	ABCA4	NM_000350.3	c.5549T>C	p.(Leu1850Pro)	Missense	Likely Pathogenic	F	21	White - British
12566560	435	ABCA4	NM_000350.3	c.4793C>A	p.(Ala1598Asp)	Missense	Pathogenic	M	25	Not stated
12566560	435	ABCA4	NM_000350.3	c.5882G>A	p.(Gly1961Glu)	Missense	Pathogenic	M	25	Not stated
11485795	436	ABCA4	NM_000350.3	c.52C>T	p.(Arg18Trp)	Missense	Pathogenic	M	62	White - Other
11485795	436	ABCA4	NM_000350.3	c.5882G>A	p.(Gly1961Glu)	Missense	Pathogenic	M	62	White - Other
12816838	437	ABCA4	NM_000350.3	c.4468T>C	p.(Cys1490Arg)	Missense	Likely Pathogenic	F	31	Not stated
12816838	437	ABCA4	NM_000350.3	c.5196+1137G>A	Splice	Splice	Pathogenic	F	31	Not stated
18040343	437	ABCA4	NM_000350.3	c.4468T>C	p.(Cys1490Arg)	Missense	Likely Pathogenic	M	25	White - Other
18040343	437	ABCA4	NM_000350.3	c.5196+1137G>A	Splice	Splice	Pathogenic	M	25	White - Other
13013923	438	ABCA4	NM_000350.3	c.2617T>C	p.(Phe873Leu)	Missense	Pathogenic	F	41	White - Other
13013923	438	ABCA4	NM_000350.3	c.4739T>C	p.(Leu1580Ser)	Missense	Pathogenic	F	41	White - Other
10170040	439	ABCA4	NM_000350.3	c.2966T>C	p.(Val989Ala)	Missense	Pathogenic	M	46	Not stated
10170040	439	ABCA4	NM_000350.3	c.4537dup	p.(Gln1513ProfsTer42)	Frameshift	Pathogenic	M	46	Not stated
11581877	440	ABCA4	NM_000350.3	c.5461-10T>C	Splice	Splice	Pathogenic	F	51	Not stated
11581877	440	ABCA4	NM_000350.3	c.6658C>T	p.(Gln2220Ter)	Stopgain	Pathogenic	F	51	Not stated
10996712	441	ABCA4	NM_000350.3	c.2588G>C	p.(Gly863Ala)	Missense	Pathogenic	M	50	White - British
10996712	441	ABCA4	NM_000350.3	c.4139C>T	p.(Pro1380Leu)	Missense	Pathogenic	M	50	White - British
7901816	442	ABCA4	NM_000350.3	c.1317G>A	p.(Trp439Ter)	Stopgain	Pathogenic	M	52	White - British
7901816	442	ABCA4	NM_000350.3	c.6721C>G	p.(Leu2241Val)	Missense	Pathogenic	M	52	White - British
13105756	443	ABCA4	NM_000350.3	c.4469G>A	p.(Cys1490Tyr)	Missense	Pathogenic	M	34	White - Other
13105756	443	ABCA4	NM_000350.3	c.5714+5G>A	Splice	Splice	Pathogenic	M	34	White - Other
12726034	444	ABCA4	NM_000350.3	c.5461-10T>C	Splice	Splice	Pathogenic	F	20	Not stated
12726034	444	ABCA4	NM_000350.3	c.5929G>A	p.(Gly1977Ser)	Missense	Pathogenic	F	20	Not stated
13094458	445	ABCA4	NM_000350.3	c.1648G>T	p.(Gly550Ter)	Stopgain	Likely Pathogenic	F	43	White - Other
13094458	445	ABCA4	NM_000350.3	c.5882G>A	p.(Gly1961Glu)	Missense	Pathogenic	F	43	White - Other
13111111	446	ABCA4	NM_000350.3	c.5461-10T>C	Splice	Splice	Pathogenic	F	59	Not stated
13111111	446	ABCA4	NM_000350.3	c.5882G>A	p.(Gly1961Glu)	Missense	Pathogenic	F	59	Not stated
9569391	447	ABCA4	NM_000350.3	c.3322C>T	p.(Arg1108Cys)	Missense	Pathogenic	M	47	Not stated
9569391	447	ABCA4	NM_000350.3	c.5714+5G>A	Splice	Splice	Pathogenic	M	47	Not stated
13254576	448	ABCA4	NM_000350.3	c.247_248dup	p.(Gln83ProfsTer17)	Frameshift	Likely Pathogenic	F	18	White - British
13254576	448	ABCA4	NM_000350.3	c.4222T>C	p.(Trp1408Arg)	Missense	Pathogenic	F	18	White - British
13254576	448	ABCA4	NM_000350.3	c.4918C>T	p.(Arg1640Trp)	Missense	Pathogenic	F	18	White - British
13053417	449	ABCA4	NM_000350.3	c.3322C>T	p.(Arg1108Cys)	Missense	Pathogenic	M	65	White - British
13053417	449	ABCA4	NM_000350.3	c.5714+5G>A	Splice	Splice	Pathogenic	M	65	White - British

13116746	450	ABCA4	NM_000350.3	c.3259G>A	p.(Glu1087Lys)	Missense	Pathogenic	F	58	Unknown
13116746	450	ABCA4	NM_000350.3	c.3840_3845del	p.(Asp1281_Ser1282del)	Inframe indel	Variant of Uncertain Significance	F	58	Unknown
13116746	450	ABCA4	NM_000350.3	c.1519G>T	p.(Asp507Tyr)	Missense	Likely Pathogenic	F	58	Unknown
13167692	451	ABCA4	NM_000350.3	c.4139C>T	p.(Pro1380Leu)	Missense	Pathogenic	F	23	White - British
13167692	451	ABCA4	NM_000350.3	c.5461-10T>C	Splice	Splice	Pathogenic	F	23	White - British
12822711	452	ABCA4	NM_000350.3	c.5312+1G>A	Splice	Splice	Pathogenic	M	42	White - Other
12822711	452	ABCA4	NM_000350.3	c.5882G>A	p.(Gly1961Glu)	Missense	Pathogenic	M	42	White - Other
12061055	453	ABCA4	NM_000350.3	c.6320G>A	p.(Arg2107His)	Missense	Pathogenic	M	48	Not stated
12061055	453	ABCA4	NM_000350.3	c.6449G>A	p.(Cys2150Tyr)	Missense	Pathogenic	M	48	Not stated
12998530	454	ABCA4	NM_000350.3	c.1804C>T	p.(Arg602Trp)	Missense	Pathogenic	F	21	Not stated
12998530	454	ABCA4	NM_000350.3	c.3113C>T	p.(Ala1038Val)	Missense	Pathogenic	F	21	Not stated
12998530	454	ABCA4	NM_000350.3	c.5461-10T>C	Splice	Splice	Pathogenic	F	21	Not stated
13102851	455	ABCA4	NM_000350.3	c.2588G>C	p.(Gly863Ala)	Missense	Pathogenic	M	58	Not stated
13102851	455	ABCA4	NM_000350.3	c.658C>T	p.(Arg220Cys)	Missense	Likely Pathogenic	M	58	Not stated
8323895	456	ABCA4	NM_000350.3	c.1819G>A	p.(Gly607Arg)	Missense	Pathogenic	F	42	Not stated
8323895	456	ABCA4	NM_000350.3	c.2128A>G	p.(Met710Val)	Missense	Variant of Uncertain Significance	F	42	Not stated
2217963	457	ABCA4	NM_000350.3	c.1715G>A	p.(Arg572Gln)	Missense	Likely Pathogenic	M	58	Not stated
2217963	457	ABCA4	NM_000350.3	c.2588G>C	p.(Gly863Ala)	Missense	Pathogenic	M	58	Not stated
2217963	457	ABCA4	NM_000350.3	c.4918C>T	p.(Arg1640Trp)	Missense	Pathogenic	M	58	Not stated
7264494	458	ABCA4	NM_000350.3	c.1957C>T	p.(Arg653Cys)	Missense	Pathogenic	M	54	White - Other
7264494	458	ABCA4	NM_000350.3	c.5714+5G>A	Splice	Splice	Pathogenic	M	54	White - Other
13209678	459	ABCA4	NM_000350.3	c.5882G>A	p.(Gly1961Glu)	Missense	Pathogenic	F	42	White - Other
13209678	459	ABCA4	NM_000350.3	c.5917del	p.(Val1973Ter)	Stopgain	Pathogenic	F	42	White - Other
3713464	460	ABCA4	NM_000350.3	c.5381C>A	p.(Ala1794Asp)	Missense	Pathogenic	M	61	Not stated
3713464	460	ABCA4	NM_000350.3	c.5461-10T>C	Splice	Splice	Pathogenic	M	61	Not stated
12154939	461	ABCA4	NM_000350.3	c.4739T>C	p.(Leu1580Ser)	Missense	Pathogenic	M	35	Asian - Indian
12154939	461	ABCA4	NM_000350.3	c.6729+5_6729+19del	Splice	Splice	Likely Pathogenic	M	35	Asian - Indian
12720476	462	ABCA4	NM_000350.3	c.1913C>T	p.(Pro638Leu)	Missense	Pathogenic	M	38	Not stated
12720476	462	ABCA4	NM_000350.3	c.5882G>A	p.(Gly1961Glu)	Missense	Pathogenic	M	38	Not stated
12482462	463	ABCA4	NM_000350.3	c.2522A>C	p.(Gln841Pro)	Missense	Likely Pathogenic	F	48	Any other ethnic group
12482462	463	ABCA4	NM_000350.3	c.5882G>A	p.(Gly1961Glu)	Missense	Pathogenic	F	48	Any other ethnic group
13254723	464	ABCA4	NM_000350.3	c.1957C>T	p.(Arg653Cys)	Missense	Pathogenic	F	22	Any other ethnic group
13254723	464	ABCA4	NM_000350.3	c.6391G>A	p.(Glu2131Lys)	Missense	Pathogenic	F	22	Any other ethnic group
13289408	465	ABCA4	NM_000350.3	c.5461-10T>C	Splice	Splice	Pathogenic	M	26	White - British
13289408	465	ABCA4	NM_000350.3	c.6088C>T	p.(Arg2030Ter)	Stopgain	Pathogenic	M	26	White - British
12713567	466	ABCA4	NM_000350.3	c.4253+5G>A	Splice	Splice	Likely Pathogenic	M	34	Asian - Pakistani
12713567	466	ABCA4	NM_000350.3	c.5882G>A	p.(Gly1961Glu)	Missense	Pathogenic	M	34	Asian - Pakistani
13311577	467	ABCA4	NM_000350.3	c.6079C>T	p.(Leu2027Phe)	Missense	Pathogenic	F	58	Not stated
13311577	467	ABCA4	NM_000350.3	c.6079C>T	p.(Leu2027Phe)	Missense	Pathogenic	F	58	Not stated
12481965	468	ABCA4	NM_000350.3	c.5196+1G>T	Splice	Splice	Likely Pathogenic	F	37	White - British
12481965	468	ABCA4	NM_000350.3	c.6079C>T	p.(Leu2027Phe)	Missense	Pathogenic	F	37	White - British
2555573	469	ABCA4	NM_000350.3	c.1317G>A	p.(Trp439Ter)	Stopgain	Pathogenic	F	49	White - British
2555573	469	ABCA4	NM_000350.3	c.5714+5G>A	Splice	Splice	Pathogenic	F	49	White - British
12626039	470	ABCA4	NM_000350.3	c.6729+5_6729+19del	Splice	Splice	Likely Pathogenic	M	18	Mixed - Other
12626039	470	ABCA4	NM_000350.3	c.6729+5_6729+19del	Splice	Splice	Likely Pathogenic	M	18	Mixed - Other
11685554	471	ABCA4	NM_000350.3	c.2560G>A	p.(Ala854Thr)	Missense	Likely Pathogenic	M	32	Not stated
11685554	471	ABCA4	NM_000350.3	c.3113C>T	p.(Ala1038Val)	Missense	Pathogenic	M	32	Not stated
11685554	471	ABCA4	NM_000350.3	c.6320G>A	p.(Arg2107His)	Missense	Pathogenic	M	32	Not stated
10563573	472	ABCA4	NM_000350.3	c.1253T>C	p.(Phe418Ser)	Missense	Pathogenic	F	21	Black - Caribbean
10563573	472	ABCA4	NM_000350.3	c.6320G>A	p.(Arg2107His)	Missense	Pathogenic	F	21	Black - Caribbean
11749128	473	ABCA4	NM_000350.3	c.3364G>A	p.(Glu1122Lys)	Missense	Pathogenic	F	26	Unknown

11749128	473	ABCA4	NM_000350.3	c.3758C>T	p.(Thr1253Met)	Missense	Variant of Uncertain Significance	F	26	Unknown
11749128	473	ABCA4	NM_000350.3	c.5882G>A	p.(Gly1961Glu)	Missense	Pathogenic	F	26	Unknown
13059136	474	ABCA4	NM_000350.3	c.1804C>T	p.(Arg602Trp)	Missense	Pathogenic	F	35	White - British
13059136	474	ABCA4	NM_000350.3	c.5196+1137G>A	Splice	Splice	Pathogenic	F	35	White - British
13259595	475	ABCA4	NM_000350.3	c.3210_3211dup	p.(Ser1071CysfsTer14)	Frameshift	Pathogenic	F	24	White - British
13259595	475	ABCA4	NM_000350.3	c.3322C>T	p.(Arg1108Cys)	Missense	Pathogenic	F	24	White - British
12066249	476	ABCA4	NM_000350.3	c.6119G>A	p.(Arg2040Gln)	Missense	Pathogenic	M	34	Not stated
12066249	476	ABCA4	NM_000350.3	c.618C>G	p.(Ser206Arg)	Missense	Variant of Uncertain Significance	M	34	Not stated
10015606	477	ABCA4	NM_000350.3	c.5461-10T>C	Splice	Splice	Pathogenic	F	39	White - British
10015606	477	ABCA4	NM_000350.3	c.5882G>A	p.(Gly1961Glu)	Missense	Pathogenic	F	39	White - British
9605763	478	ABCA4	NM_000350.3	c.71G>A	p.(Arg24His)	Missense	Pathogenic	F	46	Not stated
9605763	478	ABCA4	NM_000350.3	c.93G>A	p.(Trp31Ter)	Stopgain	Pathogenic	F	46	Not stated
8476572	479	ABCA4	NM_000350.3	c.5882G>A	p.(Gly1961Glu)	Missense	Pathogenic	F	62	Asian - Other
8476572	479	ABCA4	NM_000350.3	c.6729+5_6729+19del	Splice	Splice	Likely Pathogenic	F	62	Asian - Other
8476544	479	ABCA4	NM_000350.3	c.6729+5_6729+19del	Splice	Splice	Likely Pathogenic	F	28	Asian - Other
8476544	479	ABCA4	NM_000350.3	c.6729+5_6729+19del	Splice	Splice	Likely Pathogenic	F	28	Asian - Other
13557648	480	ABCA4	NM_000350.3	c.3322C>T	p.(Arg1108Cys)	Missense	Pathogenic	M	73	Unknown
13557648	480	ABCA4	NM_000350.3	c.5882G>A	p.(Gly1961Glu)	Missense	Pathogenic	M	73	Unknown
13426503	481	ABCA4	NM_000350.3	c.1937+1G>A	Splice	Splice	Pathogenic	F	42	Any other ethnic group
13426503	481	ABCA4	NM_000350.3	c.1937+1G>A	Splice	Splice	Pathogenic	F	42	Any other ethnic group
13209895	482	ABCA4	NM_000350.3	c.1804C>T	p.(Arg602Trp)	Missense	Pathogenic	F	37	Not stated
13209895	482	ABCA4	NM_000350.3	c.868C>T	p.(Arg290Trp)	Missense	Pathogenic	F	37	Not stated
13653387	483	ABCA4	NM_000350.3	c.2588G>C	p.(Gly863Ala)	Missense	Pathogenic	M	56	White - British
13653387	483	ABCA4	NM_000350.3	c.5461-10T>C	Splice	Splice	Pathogenic	M	56	White - British
12558748	484	ABCA4	NM_000350.3	c.1253T>C	p.(Phe418Ser)	Missense	Pathogenic	F	62	White - British
12558748	484	ABCA4	NM_000350.3	c.1927G>A	p.(Val643Met)	Missense	Variant of Uncertain Significance	F	62	White - British
13371987	485	ABCA4	NM_000350.3	c.4253+43G>A	Splice	Splice	Likely Pathogenic	F	61	Not stated
13371987	485	ABCA4	NM_000350.3	c.4577C>T	p.(Thr1526Met)	Missense	Pathogenic	F	61	Not stated
12227025	486	ABCA4	NM_000350.3	c.4463G>T	p.(Cys1488Phe)	Missense	Pathogenic	M	53	Unknown
12227025	486	ABCA4	NM_000350.3	c.5461-10T>C	Splice	Splice	Pathogenic	M	53	Unknown
9138261	487	ABCA4	NM_000350.3	c.4594G>A	p.(Asp1532Asn)	Missense	Pathogenic	M	76	Asian - Pakistani
9138261	487	ABCA4	NM_000350.3	c.4594G>A	p.(Asp1532Asn)	Missense	Pathogenic	M	76	Asian - Pakistani
13598297	488	ABCA4	NM_000350.3	c.3064G>A	p.(Glu1022Lys)	Missense	Pathogenic	F	46	Asian - Indian
13598297	488	ABCA4	NM_000350.3	c.5882G>A	p.(Gly1961Glu)	Missense	Pathogenic	F	46	Asian - Indian
12789503	489	ABCA4	NM_000350.3	c.1622T>C	p.(Leu541Pro)	Missense	Pathogenic	F	39	Not stated
12789503	489	ABCA4	NM_000350.3	c.3113C>T	p.(Ala1038Val)	Missense	Pathogenic	F	39	Not stated
12789503	489	ABCA4	NM_000350.3	c.5882G>A	p.(Gly1961Glu)	Missense	Pathogenic	F	39	Not stated
13587027	490	ABCA4	NM_000350.3	c.5461-10T>C	Splice	Splice	Pathogenic	F	55	Not stated
13587027	490	ABCA4	NM_000350.3	c.5721C>T	p.(Ala1907Ala)	Synonymous	Likely Benign	F	55	Not stated
13587027	490	ABCA4	NM_000350.3	c.5603A>T	p.(Asn1868Ile)	Missense	Variant of Uncertain Significance	F	55	Not stated
13587027	490	ABCA4	NM_000350.3	c.5603A>T	p.(Asn1868Ile)	Missense	Variant of Uncertain Significance	F	55	Not stated
13414729	491	ABCA4	NM_000350.3	c.4139C>T	p.(Pro1380Leu)	Missense	Pathogenic	F	36	Not stated
13414729	491	ABCA4	NM_000350.3	c.4363del	p.(Cys1455ValfsTer71)	Frameshift	Pathogenic	F	36	Not stated
13414729	491	ABCA4	NM_000350.3	c.4685T>C	p.(Ile1562Thr)	Missense	Pathogenic	F	36	Not stated
13634578	492	ABCA4	NM_000350.3	c.5170T>C	p.(Trp1724Arg)	Missense	Likely Pathogenic	M	17	Asian - Indian
13634578	492	ABCA4	NM_000350.3	c.5170T>C	p.(Trp1724Arg)	Missense	Likely Pathogenic	M	17	Asian - Indian
13626171	493	ABCA4	NM_000350.3	c.5882G>A	p.(Gly1961Glu)	Missense	Pathogenic	M	44	Not stated
13626171	493	ABCA4	NM_000350.3	c.5882G>A	p.(Gly1961Glu)	Missense	Pathogenic	M	44	Not stated
13802242	494	ABCA4	NM_000350.3	c.2588G>C	p.(Gly863Ala)	Missense	Pathogenic	M	41	Not stated
13802242	494	ABCA4	NM_000350.3	c.5908C>T	p.(Leu1970Phe)	Missense	Variant of Uncertain Significance	M	41	Not stated
13610981	495	ABCA4	NM_000350.3	c.2588G>C	p.(Gly863Ala)	Missense	Pathogenic	M	27	Not stated

13610981	495	ABCA4	NM_000350.3	c.4139C>T	p.(Pro1380Leu)	Missense	Pathogenic	M	27	Not stated
10208925	496	ABCA4	NM_000350.3	c.3224C>A	p.(Ala1075Asp)	Missense	Likely Pathogenic	M	60	Unknown
10208925	496	ABCA4	NM_000350.3	c.5882G>A	p.(Gly1961Glu)	Missense	Pathogenic	M	60	Unknown
11451243	497	ABCA4	NM_000350.3	c.6729+5_6729+19del	Splice	Splice	Likely Pathogenic	F	18	Any other ethnic group
11451243	497	ABCA4	NM_000350.3	c.6729+5_6729+19del	Splice	Splice	Likely Pathogenic	F	18	Any other ethnic group
10532948	497	ABCA4	NM_000350.3	c.6729+5_6729+19del	Splice	Splice	Likely Pathogenic	F	58	Any other ethnic group
10532948	497	ABCA4	NM_000350.3	c.6729+5_6729+19del	Splice	Splice	Likely Pathogenic	F	58	Any other ethnic group
10273801	498	ABCA4	NM_000350.3	c.2912C>A	p.(Thr971Asn)	Missense	Pathogenic	M	24	Asian - Bangladeshi
10273801	498	ABCA4	NM_000350.3	c.2912C>A	p.(Thr971Asn)	Missense	Pathogenic	M	24	Asian - Bangladeshi
13297206	499	ABCA4	NM_000350.3	c.2588G>C	p.(Gly863Ala)	Missense	Pathogenic	M	52	White - British
13297206	499	ABCA4	NM_000350.3	c.5196+1216C>A	Splice	Splice	Variant of Uncertain Significance	M	52	White - British
3749220	500	ABCA4	NM_000350.3	c.4139C>T	p.(Pro1380Leu)	Missense	Pathogenic	M	39	Not stated
3749220	500	ABCA4	NM_000350.3	c.4326C>A	p.(Asn1442Lys)	Missense	Likely Pathogenic	M	39	Not stated
6773024	501	ABCA4	NM_000350.3	c.6658C>T	p.(Gln2220Ter)	Stopgain	Pathogenic	M	36	Asian - Pakistani
6773024	501	ABCA4	NM_000350.3	c.859-9T>C	Splice	Splice	Likely Pathogenic	M	36	Asian - Pakistani
13863723	502	ABCA4	NM_000350.3	c.286A>T	p.(Asn96Tyr)	Missense	Likely Pathogenic	M	17	Not stated
13863723	502	ABCA4	NM_000350.3	c.5898+2T>C	Splice	Splice	Pathogenic	M	17	Not stated
13716765	503	ABCA4	NM_000350.3	c.5461-10T>C	Splice	Splice	Pathogenic	M	74	Unknown
13716765	503	ABCA4	NM_000350.3	c.5461-10T>C	Splice	Splice	Pathogenic	M	74	Unknown
13833105	504	ABCA4	NM_000350.3	c.1253T>C	p.(Phe418Ser)	Missense	Pathogenic	M	20	Not stated
13833105	504	ABCA4	NM_000350.3	c.1253T>C	p.(Phe418Ser)	Missense	Pathogenic	M	20	Not stated
5633830	505	ABCA4	NM_000350.3	c.4216C>T	p.(His1406Tyr)	Missense	Pathogenic	M	77	White - British
5633830	505	ABCA4	NM_000350.3	c.5196+1137G>C	Splice	Splice	Variant of Uncertain Significance	M	77	White - British
5633830	505	ABCA4	NM_000350.3	c.6148G>C	p.(Val2050Leu)	Missense	Variant of Uncertain Significance	M	77	White - British
13470148	506	ABCA4	NM_000350.3	c.2843G>C	p.(Arg948Pro)	Missense	Variant of Uncertain Significance	M	60	Any other ethnic group
13470148	506	ABCA4	NM_000350.3	c.5882G>A	p.(Gly1961Glu)	Missense	Pathogenic	M	60	Any other ethnic group
13872487	507	ABCA4	NM_000350.3	c.634C>T	p.(Arg212Cys)	Missense	Pathogenic	M	17	White - British
13872487	507	ABCA4	NM_000350.3	c.768G>T	p.(Val256Val)	Synonymous	Pathogenic	M	17	White - British
13907795	508	ABCA4	NM_000350.3	c.6088C>T	p.(Arg2030Ter)	Stopgain	Pathogenic	M	28	Unknown
13907795	508	ABCA4	NM_000350.3	c.6088C>T	p.(Arg2030Ter)	Stopgain	Pathogenic	M	28	Unknown
13758730	509	ABCA4	NM_000350.3	c.4253+43G>A	Splice	Splice	Likely Pathogenic	M	37	Any other ethnic group
13758730	509	ABCA4	NM_000350.3	c.5898+1G>A	Splice	Splice	Pathogenic	M	37	Any other ethnic group
13785813	510	ABCA4	NM_000350.3	c.2588G>C	p.(Gly863Ala)	Missense	Pathogenic	F	24	Any other ethnic group
13785813	510	ABCA4	NM_000350.3	c.4139C>T	p.(Pro1380Leu)	Missense	Pathogenic	F	24	Any other ethnic group
11634965	511	ABCA4	NM_000350.3	c.3292C>T	p.(Arg1098Cys)	Missense	Pathogenic	M	65	Not stated
11634965	511	ABCA4	NM_000350.3	c.5461-10T>C	Splice	Splice	Pathogenic	M	65	Not stated
13724353	512	ABCA4	NM_000350.3	c.2453G>A	p.(Gly818Glu)	Missense	Pathogenic	M	41	Not stated
13724353	512	ABCA4	NM_000350.3	c.6817-2A>C	Splice	Splice	Pathogenic	M	41	Not stated
13814352	513	ABCA4	NM_000350.3	c.4070C>T	p.(Ala1357Val)	Missense	Pathogenic	F	37	White - British
13814352	513	ABCA4	NM_000350.3	c.6079C>T	p.(Leu2027Phe)	Missense	Pathogenic	F	37	White - British
647870	514	ABCA4	NM_000350.3	c.4469G>A	p.(Cys1490Tyr)	Missense	Pathogenic	M	61	Not stated
647870	514	ABCA4	NM_000350.3	c.6118C>T	p.(Arg2040Ter)	Stopgain	Pathogenic	M	61	Not stated
2851225	514	ABCA4	NM_000350.3	c.5714+5G>A	Splice	Splice	Pathogenic	F	83	White - British
2851225	514	ABCA4	NM_000350.3	c.6118C>T	p.(Arg2040Ter)	Stopgain	Pathogenic	F	83	White - British
13661108	515	ABCA4	NM_000350.3	c.214G>A	p.(Gly72Arg)	Missense	Pathogenic	F	17	Not stated
13661108	515	ABCA4	NM_000350.3	c.214G>A	p.(Gly72Arg)	Missense	Pathogenic	F	17	Not stated
13717955	516	ABCA4	NM_000350.3	c.3191-1G>T	Splice	Splice	Likely Pathogenic	F	17	Any other ethnic group
13717955	516	ABCA4	NM_000350.3	c.4469G>A	p.(Cys1490Tyr)	Missense	Pathogenic	F	17	Any other ethnic group
13951692	517	ABCA4	NM_000350.3	c.4253+43G>A	Splice	Splice	Likely Pathogenic	M	65	Not stated
13951692	517	ABCA4	NM_000350.3	c.4469G>A	p.(Cys1490Tyr)	Missense	Pathogenic	M	65	Not stated
11578727	518	ABCA4	NM_000350.3	c.3303G>A	p.(Trp1101Ter)	Stopgain	Pathogenic	M	42	Not stated

11578727	518	ABCA4	NM_000350.3	c.3303G>A	p.(Trp1101Ter)	Stopgain	Pathogenic	M	42	Not stated
10032238	519	ABCA4	NM_000350.3	c.5932A>G	p.(Lys1978Glu)	Missense	Pathogenic	M	16	Asian - Pakistani
10032238	519	ABCA4	NM_000350.3	c.5932A>G	p.(Lys1978Glu)	Missense	Pathogenic	M	16	Asian - Pakistani
15506175	520	ABCA4	NM_000350.3	c.5461-10T>C	Splice	Splice	Pathogenic	F	21	Not stated
15506175	520	ABCA4	NM_000350.3	c.6319C>T	p.(Arg2107Cys)	Missense	Pathogenic	F	21	Not stated
13981141	521	ABCA4	NM_000350.3	c.2894A>G	p.(Asn965Ser)	Missense	Pathogenic	F	41	Not stated
13981141	521	ABCA4	NM_000350.3	c.5882G>A	p.(Gly1961Glu)	Missense	Pathogenic	F	41	Not stated
13978614	522	ABCA4	NM_000350.3	c.2912C>A	p.(Thr971Asn)	Missense	Pathogenic	M	33	Unknown
13978614	522	ABCA4	NM_000350.3	c.5882G>A	p.(Gly1961Glu)	Missense	Pathogenic	M	33	Unknown
2369205	523	ABCA4	NM_000350.3	c.4139C>T	p.(Pro1380Leu)	Missense	Pathogenic	M	60	Unknown
2369205	523	ABCA4	NM_000350.3	c.634C>T	p.(Arg212Cys)	Missense	Pathogenic	M	60	Unknown
13984998	524	ABCA4	NM_000350.3	c.4326C>A	p.(Asn1442Lys)	Missense	Likely Pathogenic	M	20	Not stated
13984998	524	ABCA4	NM_000350.3	c.5461-10T>C	Splice	Splice	Pathogenic	M	20	Not stated
10517058	525	ABCA4	NM_000350.3	c.1804C>T	p.(Arg602Trp)	Missense	Pathogenic	M	41	White - Other
10517058	525	ABCA4	NM_000350.3	c.3259G>A	p.(Glu1087Lys)	Missense	Pathogenic	M	41	White - Other
13984746	526	ABCA4	NM_000350.3	c.1760+2T>C	Splice	Splice	Likely Pathogenic	F	25	Not stated
13984746	526	ABCA4	NM_000350.3	c.3322C>T	p.(Arg1108Cys)	Missense	Pathogenic	F	25	Not stated
13448539	527	ABCA4	NM_000350.3	c.6729+5_6729+19del	Splice	Splice	Likely Pathogenic	M	24	Not stated
13448539	527	ABCA4	NM_000350.3	c.6729+5_6729+19del	Splice	Splice	Likely Pathogenic	M	24	Not stated
14712823	528	ABCA4	NM_000350.3	c.161G>A	p.(Cys54Tyr)	Missense	Pathogenic	F	26	Not stated
14712823	528	ABCA4	NM_000350.3	c.3259G>A	p.(Glu1087Lys)	Missense	Pathogenic	F	26	Not stated
11329114	529	ABCA4	NM_000350.3	c.6729+5_6729+19del	Splice	Splice	Likely Pathogenic	F	68	Asian - Other
11329114	529	ABCA4	NM_000350.3	c.6729+5_6729+19del	Splice	Splice	Likely Pathogenic	F	68	Asian - Other
12532960	530	ABCA4	NM_000350.3	c.29dup	p.(Leu10PhefsTer44)	Frameshift	Likely Pathogenic	M	18	Not stated
12532960	530	ABCA4	NM_000350.3	c.4539+2028C>T	Splice	Splice	Likely Pathogenic	M	18	Not stated
9641953	531	ABCA4	NM_000350.3	c.161G>A	p.(Cys54Tyr)	Missense	Pathogenic	M	36	Not stated
9641953	531	ABCA4	NM_000350.3	c.5882G>A	p.(Gly1961Glu)	Missense	Pathogenic	M	36	Not stated
13744002	532	ABCA4	NM_000350.3	c.5603A>T	p.(Asn1868Ile)	Missense	Variant of Uncertain Significance	F	58	White - British
13744002	532	ABCA4	NM_000350.3	c.6118C>T	p.(Arg2040Ter)	Stopgain	Pathogenic	F	58	White - British
13994784	532	ABCA4	NM_000350.3	c.5603A>T	p.(Asn1868Ile)	Missense	Variant of Uncertain Significance	F	60	White - British
13994784	532	ABCA4	NM_000350.3	c.6118C>T	p.(Arg2040Ter)	Stopgain	Pathogenic	F	60	White - British
14828211	533	ABCA4	NM_000350.3	c.1622T>C	p.(Leu541Pro)	Missense	Pathogenic	M	43	Any other ethnic group
14828211	533	ABCA4	NM_000350.3	c.3113C>T	p.(Ala1038Val)	Missense	Pathogenic	M	43	Any other ethnic group
14828211	533	ABCA4	NM_000350.3	c.5882G>A	p.(Gly1961Glu)	Missense	Pathogenic	M	43	Any other ethnic group
13692118	534	ABCA4	NM_000350.3	c.1339C>T	p.(Gln447Ter)	Stopgain	Pathogenic	M	37	Not stated
13692118	534	ABCA4	NM_000350.3	c.5714+5G>A	Splice	Splice	Pathogenic	M	37	Not stated
13749490	535	ABCA4	NM_000350.3	c.5882G>A	p.(Gly1961Glu)	Missense	Pathogenic	M	37	Not stated
13749490	535	ABCA4	NM_000350.3	c.834del	p.(Asp279IlefsTer21)	Frameshift	Pathogenic	M	37	Not stated
12921747	536	ABCA4	NM_000350.3	c.3329-1G>A	Splice	Splice	Pathogenic	M	70	Not stated
12921747	536	ABCA4	NM_000350.3	c.6089G>A	p.(Arg2030Gln)	Missense	Pathogenic	M	70	Not stated
10799599	537	ABCA4	NM_000350.3	c.5882G>A	p.(Gly1961Glu)	Missense	Pathogenic	F	42	Any other ethnic group
10799599	537	ABCA4	NM_000350.3	c.5882G>A	p.(Gly1961Glu)	Missense	Pathogenic	F	42	Any other ethnic group
10799599	537	ABCA4	NM_000350.3	c.634C>T	p.(Arg212Cys)	Missense	Pathogenic	F	42	Any other ethnic group
14828981	538	ABCA4	NM_000350.3	c.3113C>T	p.(Ala1038Val)	Missense	Pathogenic	M	74	Unknown
14828981	538	ABCA4	NM_000350.3	c.5714+5G>A	Splice	Splice	Pathogenic	M	74	Unknown
11217268	539	ABCA4	NM_000350.3	c.4253+4C>T	Splice	Splice	Likely Pathogenic	M	36	Mixed - Other
11217268	539	ABCA4	NM_000350.3	c.5714+5G>A	Splice	Splice	Pathogenic	M	36	Mixed - Other
11217268	539	ABCA4	NM_000350.3	c.5908C>T	p.(Leu1970Phe)	Missense	Variant of Uncertain Significance	M	36	Mixed - Other
14764266	540	ABCA4	NM_000350.3	c.4216C>T	p.(His1406Tyr)	Missense	Pathogenic	M	49	White - British
14764266	540	ABCA4	NM_000350.3	c.5196+1137G>A	Splice	Splice	Pathogenic	M	49	White - British
14764266	540	ABCA4	NM_000350.3	c.6148G>C	p.(Val2050Leu)	Missense	Variant of Uncertain Significance	M	49	White - British

7530907	541	ABCA4	NM_000350.3	c.5929G>A	p.(Gly1977Ser)	Missense	Pathogenic	M	41	Any other ethnic group
7530907	541	ABCA4	NM_000350.3	c.5929G>A	p.(Gly1977Ser)	Missense	Pathogenic	M	41	Any other ethnic group
10254523	542	ABCA4	NM_000350.3	c.108del	p.(Leu37TrpfsTer3)	Frameshift	Pathogenic	M	43	White - British
10254523	542	ABCA4	NM_000350.3	c.6449G>A	p.(Cys2150Tyr)	Missense	Pathogenic	M	43	White - British
16267285	543	ABCA4	NM_000350.3	c.2588G>C	p.(Gly863Ala)	Missense	Pathogenic	F	30	Not stated
16267285	543	ABCA4	NM_000350.3	c.768G>T	p.(Val256Val)	Synonymous	Pathogenic	F	30	Not stated
3838218	544	ABCA4	NM_000350.3	c.3056C>T	p.(Thr1019Met)	Missense	Pathogenic	F	47	White - British
3838218	544	ABCA4	NM_000350.3	c.3322C>T	p.(Arg1108Cys)	Missense	Pathogenic	F	47	White - British
13905044	545	ABCA4	NM_000350.3	c.2813T>C	p.(Phe938Ser)	Missense	Pathogenic	M	26	Not stated
13905044	545	ABCA4	NM_000350.3	c.3056C>T	p.(Thr1019Met)	Missense	Pathogenic	M	26	Not stated
14883084	546	ABCA4	NM_000350.3	c.3064G>A	p.(Glu1022Lys)	Missense	Pathogenic	M	22	Not stated
14883084	546	ABCA4	NM_000350.3	c.3064G>A	p.(Glu1022Lys)	Missense	Pathogenic	M	22	Not stated
14839943	547	ABCA4	NM_000350.3	c.161G>A	p.(Cys54Tyr)	Missense	Pathogenic	M	15	Not stated
14839943	547	ABCA4	NM_000350.3	c.655A>T	p.(Arg219Ter)	Stopgain	Pathogenic	M	15	Not stated
14956416	548	ABCA4	NM_000350.3	c.1906C>T	p.(Gln636Ter)	Stopgain	Pathogenic	F	35	Not stated
14956416	548	ABCA4	NM_000350.3	c.6079C>T	p.(Leu2027Phe)	Missense	Pathogenic	F	35	Not stated
14947610	549	ABCA4	NM_000350.3	c.5714+5G>A	Splice	Splice	Pathogenic	M	47	Unknown
14947610	549	ABCA4	NM_000350.3	c.5714+5G>A	Splice	Splice	Pathogenic	M	47	Unknown
9332728	550	ABCA4	NM_000350.3	c.4537dup	p.(Gln1513ProfsTer42)	Frameshift	Pathogenic	M	40	Any other ethnic group
9332728	550	ABCA4	NM_000350.3	c.5882G>A	p.(Gly1961Glu)	Missense	Pathogenic	M	40	Any other ethnic group
13933625	551	ABCA4	NM_000350.3	c.1222C>T	p.(Arg408Ter)	Stopgain	Pathogenic	M	39	Not stated
13933625	551	ABCA4	NM_000350.3	c.5327C>T	p.(Pro1776Leu)	Missense	Pathogenic	M	39	Not stated
10881576	552	ABCA4	NM_000350.3	c.5882G>A	p.(Gly1961Glu)	Missense	Pathogenic	M	49	Any other ethnic group
10881576	552	ABCA4	NM_000350.3	c.6729+5_6729+19del	Splice	Splice	Likely Pathogenic	M	49	Any other ethnic group
14871835	553	ABCA4	NM_000350.3	c.4577C>T	p.(Thr1526Met)	Missense	Pathogenic	M	27	Not stated
14871835	553	ABCA4	NM_000350.3	c.5461-10T>C	Splice	Splice	Pathogenic	M	27	Not stated
14833923	554	ABCA4	NM_000350.3	c.3322C>T	p.(Arg1108Cys)	Missense	Pathogenic	M	35	Not stated
14833923	554	ABCA4	NM_000350.3	c.6319C>T	p.(Arg2107Cys)	Missense	Pathogenic	M	35	Not stated
13180047	555	ABCA4	NM_000350.3	c.2588G>C	p.(Gly863Ala)	Missense	Pathogenic	F	42	White - Other
13180047	555	ABCA4	NM_000350.3	c.3210_3211dup	p.(Ser1071CysfsTer14)	Frameshift	Pathogenic	F	42	White - Other
14939889	556	ABCA4	NM_000350.3	c.1335C>G	p.(Ser445Arg)	Missense	Pathogenic	F	65	White - British
14939889	556	ABCA4	NM_000350.3	c.454C>T	p.(Arg152Ter)	Stopgain	Pathogenic	F	65	White - British
2729047	557	ABCA4	NM_000350.3	c.3322C>T	p.(Arg1108Cys)	Missense	Pathogenic	M	55	Any other ethnic group
2729047	557	ABCA4	NM_000350.3	c.4981del	p.(Leu1661Ter)	Stopgain	Pathogenic	M	55	Any other ethnic group
14872997	558	ABCA4	NM_000350.3	c.5714+5G>A	Splice	Splice	Pathogenic	F	27	Not stated
14872997	558	ABCA4	NM_000350.3	c.6449G>A	p.(Cys2150Tyr)	Missense	Pathogenic	F	27	Not stated
13300559	559	ABCA4	NM_000350.3	c.1335C>G	p.(Ser445Arg)	Missense	Pathogenic	M	35	Unknown
13300559	559	ABCA4	NM_000350.3	c.1715G>A	p.(Arg572Gln)	Missense	Likely Pathogenic	M	35	Unknown
13300559	559	ABCA4	NM_000350.3	c.2588G>C	p.(Gly863Ala)	Missense	Pathogenic	M	35	Unknown
3986394	560	ABCA4	NM_000350.3	c.1715G>A	p.(Arg572Gln)	Missense	Likely Pathogenic	F	69	Any other ethnic group
3986394	560	ABCA4	NM_000350.3	c.2041C>T	p.(Arg681Ter)	Stopgain	Pathogenic	F	69	Any other ethnic group
3986394	560	ABCA4	NM_000350.3	c.2588G>C	p.(Gly863Ala)	Missense	Pathogenic	F	69	Any other ethnic group
11509259	561	ABCA4	NM_000350.3	c.2827C>T	p.(Arg943Trp)	Missense	Pathogenic	M	43	Not stated
11509259	561	ABCA4	NM_000350.3	c.4577C>T	p.(Thr1526Met)	Missense	Pathogenic	M	43	Not stated
7862637	562	ABCA4	NM_000350.3	c.4139C>T	p.(Pro1380Leu)	Missense	Pathogenic	M	32	White - British
7862637	562	ABCA4	NM_000350.3	c.5461-10T>C	Splice	Splice	Pathogenic	M	32	White - British
7102255	563	ABCA4	NM_000350.3	c.2588G>C	p.(Gly863Ala)	Missense	Pathogenic	F	56	White - British
7102255	563	ABCA4	NM_000350.3	c.3322C>T	p.(Arg1108Cys)	Missense	Pathogenic	F	56	White - British
13421477	564	ABCA4	NM_000350.3	c.5882G>A	p.(Gly1961Glu)	Missense	Pathogenic	F	38	Not stated
13421477	564	ABCA4	NM_000350.3	c.5882G>A	p.(Gly1961Glu)	Missense	Pathogenic	F	38	Not stated
8253272	565	ABCA4	NM_000350.3	c.2588G>C	p.(Gly863Ala)	Missense	Pathogenic	M	58	White - British

8253272	565	ABCA4	NM_000350.3	c.5461-10T>C	Splice	Splice	Pathogenic	M	58	White - British
14961407	566	ABCA4	NM_000350.3	c.4139C>T	p.(Pro1380Leu)	Missense	Pathogenic	M	17	Unknown
14961407	566	ABCA4	NM_000350.3	c.4577C>T	p.(Thr1526Met)	Missense	Pathogenic	M	17	Unknown
14974854	567	ABCA4	NM_000350.3	c.4363T>C	p.(Cys1455Arg)	Missense	Pathogenic	M	54	Not stated
14974854	567	ABCA4	NM_000350.3	c.6088C>T	p.(Arg2030Ter)	Stopgain	Pathogenic	M	54	Not stated
1502024	568	ABCA4	NM_000350.3	c.3392_3393delinsG	p.(Ala1131GlyfsTer17)	Frameshift	Likely Pathogenic	F	73	Asian - Indian
1502024	568	ABCA4	NM_000350.3	c.859-9T>C	Splice	Splice	Likely Pathogenic	F	73	Asian - Indian
1508982	568	ABCA4	NM_000350.3	c.3392_3393delinsG	p.(Ala1131GlyfsTer17)	Frameshift	Likely Pathogenic	M	71	Asian - Indian
1508982	568	ABCA4	NM_000350.3	c.859-9T>C	Splice	Splice	Likely Pathogenic	M	71	Asian - Indian
13113666	569	ABCA4	NM_000350.3	c.5461-10T>C	Splice	Splice	Pathogenic	F	34	White - Other
13113666	569	ABCA4	NM_000350.3	c.5882G>A	p.(Gly1961Glu)	Missense	Pathogenic	F	34	White - Other
15209228	570	ABCA4	NM_000350.3	c.4469G>A	p.(Cys1490Tyr)	Missense	Pathogenic	M	15	Not stated
15209228	570	ABCA4	NM_000350.3	c.6449G>A	p.(Cys2150Tyr)	Missense	Pathogenic	M	15	Not stated
14791342	571	ABCA4	NM_000350.3	c.3197T>G	p.(Met1066Arg)	Missense	Likely Pathogenic	F	44	Not stated
14791342	571	ABCA4	NM_000350.3	c.5882G>A	p.(Gly1961Glu)	Missense	Pathogenic	F	44	Not stated
3025098	572	ABCA4	NM_000350.3	c.2588G>C	p.(Gly863Ala)	Missense	Pathogenic	F	58	Not stated
3025098	572	ABCA4	NM_000350.3	c.4685T>C	p.(Ile1562Thr)	Missense	Pathogenic	F	58	Not stated
3025098	572	ABCA4	NM_000350.3	c.5461-10T>C	Splice	Splice	Pathogenic	F	58	Not stated
15081751	573	ABCA4	NM_000350.3	c.3262C>A	p.(Pro1088Thr)	Missense	Likely Pathogenic	F	33	Mixed - Other
15081751	573	ABCA4	NM_000350.3	c.4539+2066C>T	Splice	Splice	Variant of Uncertain Significance	F	33	Mixed - Other
15081751	573	ABCA4	NM_000350.3	c.5882G>A	p.(Gly1961Glu)	Missense	Pathogenic	F	33	Mixed - Other
14721762	574	ABCA4	NM_000350.3	c.5904del	p.(Phe1968LeufsTer6)	Frameshift	Likely Pathogenic	F	19	Asian - Indian
14721762	574	ABCA4	NM_000350.3	c.5904del	p.(Phe1968LeufsTer6)	Frameshift	Likely Pathogenic	F	19	Asian - Indian
6830823	575	ABCA4	NM_000350.3	c.1906C>T	p.(Gln636Ter)	Stopgain	Pathogenic	F	38	Any other ethnic group
6830823	575	ABCA4	NM_000350.3	c.6079C>T	p.(Leu2027Phe)	Missense	Pathogenic	F	38	Any other ethnic group
13539231	576	ABCA4	NM_000350.3	c.4571A>G	p.(Asp1524Gly)	Missense	Variant of Uncertain Significance	F	20	Asian - Bangladeshi
13539231	576	ABCA4	NM_000350.3	c.4571A>G	p.(Asp1524Gly)	Missense	Variant of Uncertain Significance	F	20	Asian - Bangladeshi
15184980	577	ABCA4	NM_000350.3	c.377G>A	p.(Trp126Ter)	Stopgain	Likely Pathogenic	M	29	Unknown
15184980	577	ABCA4	NM_000350.3	c.6119G>A	p.(Arg2040Gln)	Missense	Pathogenic	M	29	Unknown
13831187	577	ABCA4	NM_000350.3	c.377G>A	p.(Trp126Ter)	Stopgain	Likely Pathogenic	M	25	Not stated
13831187	577	ABCA4	NM_000350.3	c.6119G>A	p.(Arg2040Gln)	Missense	Pathogenic	M	25	Not stated
13501753	578	ABCA4	NM_000350.3	c.4240_4253del	p.(Tyr1414HisfsTer3)	Frameshift	Likely Pathogenic	F	41	Not stated
13501753	578	ABCA4	NM_000350.3	c.4253+43G>A	Splice	Splice	Likely Pathogenic	F	41	Not stated
15195466	579	ABCA4	NM_000350.3	c.1654G>A	p.(Val552Ile)	Missense	Variant of Uncertain Significance	F	52	Not stated
15195466	579	ABCA4	NM_000350.3	c.4234C>T	p.(Gln1412Ter)	Stopgain	Pathogenic	F	52	Not stated
15195466	579	ABCA4	NM_000350.3	c.5882G>A	p.(Gly1961Glu)	Missense	Pathogenic	F	52	Not stated
8164120	580	ABCA4	NM_000350.3	c.1099+5G>C	Splice	Splice	Variant of Uncertain Significance	F	40	Black - African
8164120	580	ABCA4	NM_000350.3	c.5882G>A	p.(Gly1961Glu)	Missense	Pathogenic	F	40	Black - African
15254854	581	ABCA4	NM_000350.3	c.1253T>C	p.(Phe418Ser)	Missense	Pathogenic	M	17	White - British
15254854	581	ABCA4	NM_000350.3	c.3322C>T	p.(Arg1108Cys)	Missense	Pathogenic	M	17	White - British
14757028	582	ABCA4	NM_000350.3	c.1222C>T	p.(Arg408Ter)	Stopgain	Pathogenic	F	31	Any other ethnic group
14757028	582	ABCA4	NM_000350.3	c.5882G>A	p.(Gly1961Glu)	Missense	Pathogenic	F	31	Any other ethnic group
15409400	583	ABCA4	NM_000350.3	c.4462T>C	p.(Cys1488Arg)	Missense	Pathogenic	M	23	Not stated
15409400	583	ABCA4	NM_000350.3	c.5882G>A	p.(Gly1961Glu)	Missense	Pathogenic	M	23	Not stated
15388995	584	ABCA4	NM_000350.3	c.1804C>T	p.(Arg602Trp)	Missense	Pathogenic	M	16	Not stated
15388995	584	ABCA4	NM_000350.3	c.885del	p.(Leu296CysfsTer4)	Frameshift	Pathogenic	M	16	Not stated
15350138	585	ABCA4	NM_000350.3	c.2971G>C	p.(Gly991Arg)	Missense	Pathogenic	M	40	Not stated
15350138	585	ABCA4	NM_000350.3	c.4519G>A	p.(Gly1507Arg)	Missense	Pathogenic	M	40	Not stated
15350138	585	ABCA4	NM_000350.3	c.5882G>A	p.(Gly1961Glu)	Missense	Pathogenic	M	40	Not stated
13102298	586	ABCA4	NM_000350.3	c.4469G>A	p.(Cys1490Tyr)	Missense	Pathogenic	M	61	Not stated
13102298	586	ABCA4	NM_000350.3	c.5882G>A	p.(Gly1961Glu)	Missense	Pathogenic	M	61	Not stated

14881138	587	ABCA4	NM_000350.3	c.5882G>A	p.(Gly1961Glu)	Missense	Pathogenic	F	58	Any other ethnic group
14881138	587	ABCA4	NM_000350.3	c.6729+5_6729+19del	Splice	Splice	Likely Pathogenic	F	58	Any other ethnic group
15255827	588	ABCA4	NM_000350.3	c.2588G>C	p.(Gly863Ala)	Missense	Pathogenic	F	65	Unknown
15255827	588	ABCA4	NM_000350.3	c.5714+5G>A	Splice	Splice	Pathogenic	F	65	Unknown
15435650	589	ABCA4	NM_000350.3	c.1648G>A	p.(Gly550Arg)	Missense	Pathogenic	M	20	White - British
15435650	589	ABCA4	NM_000350.3	c.4918C>T	p.(Arg1640Trp)	Missense	Pathogenic	M	20	White - British
15309083	590	ABCA4	NM_000350.3	c.1817G>A	p.(Gly606Asp)	Missense	Pathogenic	M	54	Unknown
15309083	590	ABCA4	NM_000350.3	c.5461-10T>C	Splice	Splice	Pathogenic	M	54	Unknown
11415564	591	ABCA4	NM_000350.3	c.3305A>T	p.(Asp1102Val)	Missense	Likely Pathogenic	M	45	Unknown
11415564	591	ABCA4	NM_000350.3	c.6438C>T	p.(Gly2146Gly)	Synonymous	Likely Benign	M	45	Unknown
11415564	591	ABCA4	NM_000350.3	c.785A>G	p.(Asp262Gly)	Missense	Variant of Uncertain Significance	M	45	Unknown
11415564	591	ABCA4	NM_000350.3	c.5603A>T	p.(Asn1868Ile)	Missense	Variant of Uncertain Significance	M	45	Unknown
11664911	592	ABCA4	NM_000350.3	c.5882G>A	p.(Gly1961Glu)	Missense	Pathogenic	M	41	Asian - Other
11664911	592	ABCA4	NM_000350.3	c.6658C>T	p.(Gln2220Ter)	Stopgain	Pathogenic	M	41	Asian - Other
14957914	593	ABCA4	NM_000350.3	c.5882G>A	p.(Gly1961Glu)	Missense	Pathogenic	M	55	Asian - Other
14957914	593	ABCA4	NM_000350.3	c.6445C>T	p.(Arg2149Ter)	Stopgain	Pathogenic	M	55	Asian - Other
15463839	594	ABCA4	NM_000350.3	c.*55G>T	Downstream	Downstream	Likely Pathogenic	F	17	Asian - Indian
15463839	594	ABCA4	NM_000350.3	c.5018+5G>A	Splice	Splice	Pathogenic	F	17	Asian - Indian
15463839	594	ABCA4	NM_000350.3	c.6445C>T	p.(Arg2149Ter)	Stopgain	Pathogenic	F	17	Asian - Indian
12048364	595	ABCA4	NM_000350.3	c.4363T>C	p.(Cys1455Arg)	Missense	Pathogenic	F	19	White - British
12048364	595	ABCA4	NM_000350.3	c.5461-10T>C	Splice	Splice	Pathogenic	F	19	White - British
15020214	596	ABCA4	NM_000350.3	c.1622T>C	p.(Leu541Pro)	Missense	Pathogenic	F	41	White - Other
15020214	596	ABCA4	NM_000350.3	c.3113C>T	p.(Ala1038Val)	Missense	Pathogenic	F	41	White - Other
15020214	596	ABCA4	NM_000350.3	c.5882G>A	p.(Gly1961Glu)	Missense	Pathogenic	F	41	White - Other
15213988	597	ABCA4	NM_000350.3	c.3322C>T	p.(Arg1108Cys)	Missense	Pathogenic	F	18	Not stated
15213988	597	ABCA4	NM_000350.3	c.454C>T	p.(Arg152Ter)	Stopgain	Pathogenic	F	18	Not stated
15485112	598	ABCA4	NM_000350.3	c.5603A>T	p.(Asn1868Ile)	Missense	Variant of Uncertain Significance	M	62	White - British
15485112	598	ABCA4	NM_000350.3	c.731T>C	p.(Leu244Pro)	Missense	Likely Pathogenic	M	62	White - British
15555392	599	ABCA4	NM_000350.3	c.161G>A	p.(Cys54Tyr)	Missense	Pathogenic	F	22	Not stated
15555392	599	ABCA4	NM_000350.3	c.5882G>A	p.(Gly1961Glu)	Missense	Pathogenic	F	22	Not stated
9555734	600	ABCA4	NM_000350.3	c.3210_3211dup	p.(Ser1071CysfsTer14)	Frameshift	Pathogenic	F	34	White - British
9555734	600	ABCA4	NM_000350.3	c.4328G>A	p.(Arg1443His)	Missense	Pathogenic	F	34	White - British
15478462	601	ABCA4	NM_000350.3	c.1726G>C	p.(Asp576His)	Missense	Pathogenic	M	32	White - British
15478462	601	ABCA4	NM_000350.3	c.5461-10T>C	Splice	Splice	Pathogenic	M	32	White - British
1057594	602	ABCA4	NM_000350.3	c.4222T>C	p.(Trp1408Arg)	Missense	Pathogenic	M	68	White - British
1057594	602	ABCA4	NM_000350.3	c.4918C>T	p.(Arg1640Trp)	Missense	Pathogenic	M	68	White - British
1057594	602	ABCA4	NM_000350.3	c.4326C>A	p.(Asn1442Lys)	Missense	Likely Pathogenic	M	68	White - British
6519694	603	ABCA4	NM_000350.3	c.4793C>A	p.(Ala1598Asp)	Missense	Pathogenic	M	30	Any other ethnic group
6519694	603	ABCA4	NM_000350.3	c.4793C>A	p.(Ala1598Asp)	Missense	Pathogenic	M	30	Any other ethnic group
12718180	604	ABCA4	NM_000350.3	c.4253+43G>A	Splice	Splice	Likely Pathogenic	F	47	Not stated
12718180	604	ABCA4	NM_000350.3	c.5603A>T	p.(Asn1868Ile)	Missense	Variant of Uncertain Significance	F	47	Not stated
12718180	604	ABCA4	NM_000350.3	c.6079C>T	p.(Leu2027Phe)	Missense	Pathogenic	F	47	Not stated
8732023	605	ABCA4	NM_000350.3	c.5882G>A	p.(Gly1961Glu)	Missense	Pathogenic	M	31	Any other ethnic group
8732023	605	ABCA4	NM_000350.3	c.634C>T	p.(Arg212Cys)	Missense	Pathogenic	M	31	Any other ethnic group
15407748	606	ABCA4	NM_000350.3	c.4873C>A	p.(His1625Asn)	Missense	Likely Pathogenic	F	32	Asian - Pakistani
15407748	606	ABCA4	NM_000350.3	c.5882G>A	p.(Gly1961Glu)	Missense	Pathogenic	F	32	Asian - Pakistani
17250477	606	ABCA4	NM_000350.3	c.4873C>A	p.(His1625Asn)	Missense	Likely Pathogenic	F	19	Not stated
17250477	606	ABCA4	NM_000350.3	c.5882G>A	p.(Gly1961Glu)	Missense	Pathogenic	F	19	Not stated
5580343	607	ABCA4	NM_000350.3	c.1253T>C	p.(Phe418Ser)	Missense	Pathogenic	F	54	White - British
5580343	607	ABCA4	NM_000350.3	c.1253T>C	p.(Phe418Ser)	Missense	Pathogenic	F	54	White - British
5580343	607	ABCA4	NM_000350.3	c.6079C>T	p.(Leu2027Phe)	Missense	Pathogenic	F	54	White - British

15431842	608	ABCA4	NM_000350.3	c.1903C>A	p.(Gln635Lys)	Missense	Pathogenic	M	39	Not stated
15431842	608	ABCA4	NM_000350.3	c.5882G>A	p.(Gly1961Glu)	Missense	Pathogenic	M	39	Not stated
983408	609	ABCA4	NM_000350.3	c.4067A>C	p.(Gln1356Pro)	Missense	Variant of Uncertain Significance	M	72	White - British
983408	609	ABCA4	NM_000350.3	c.4577C>T	p.(Thr1526Met)	Missense	Pathogenic	M	72	White - British
15600010	610	ABCA4	NM_000350.3	c.5714+5G>A	Splice	Splice	Pathogenic	F	59	White - British
15600010	610	ABCA4	NM_000350.3	c.5882G>A	p.(Gly1961Glu)	Missense	Pathogenic	F	59	White - British
15715594	611	ABCA4	NM_000350.3	c.1928T>G	p.(Val643Gly)	Missense	Likely Pathogenic	M	16	Not stated
15715594	611	ABCA4	NM_000350.3	c.3840_3845del	p.(Asp1281_Ser1282del)	Inframe indel	Variant of Uncertain Significance	M	16	Not stated
10346622	612	ABCA4	NM_000350.3	c.6079C>T	p.(Leu2027Phe)	Missense	Pathogenic	F	32	White - British
10346622	612	ABCA4	NM_000350.3	c.6079C>T	p.(Leu2027Phe)	Missense	Pathogenic	F	32	White - British
15549624	613	ABCA4	NM_000350.3	c.1856T>A	p.(Ile619Asn)	Missense	Variant of Uncertain Significance	M	31	Not stated
15549624	613	ABCA4	NM_000350.3	c.3210_3211dup	p.(Ser1071CysfsTer14)	Frameshift	Pathogenic	M	31	Not stated
3037131	614	ABCA4	NM_000350.3	c.5882G>A	p.(Gly1961Glu)	Missense	Pathogenic	M	56	Not stated
3037131	614	ABCA4	NM_000350.3	c.93G>A	p.(Trp31Ter)	Stopgain	Pathogenic	M	56	Not stated
15359749	615	ABCA4	NM_000350.3	c.4463G>T	p.(Cys1488Phe)	Missense	Pathogenic	M	30	Any other ethnic group
15359749	615	ABCA4	NM_000350.3	c.5461-10T>C	Splice	Splice	Pathogenic	M	30	Any other ethnic group
15386069	616	ABCA4	NM_000350.3	c.1253T>C	p.(Phe418Ser)	Missense	Pathogenic	M	28	White - British
15386069	616	ABCA4	NM_000350.3	c.1253T>C	p.(Phe418Ser)	Missense	Pathogenic	M	28	White - British
15386069	616	ABCA4	NM_000350.3	c.2588G>C	p.(Gly863Ala)	Missense	Pathogenic	M	28	White - British
7548337	617	ABCA4	NM_000350.3	c.2587+2T>C	Splice	Splice	Pathogenic	M	50	Mixed - White and Asian
7548337	617	ABCA4	NM_000350.3	c.5882G>A	p.(Gly1961Glu)	Missense	Pathogenic	M	50	Mixed - White and Asian
7548337	617	ABCA4	NM_000350.3	c.5882G>A	p.(Gly1961Glu)	Missense	Pathogenic	M	50	Mixed - White and Asian
13984438	618	ABCA4	NM_000350.3	c.4577C>T	p.(Thr1526Met)	Missense	Pathogenic	F	29	White - British
13984438	618	ABCA4	NM_000350.3	c.5882G>A	p.(Gly1961Glu)	Missense	Pathogenic	F	29	White - British
13688541	619	ABCA4	NM_000350.3	c.3352C>T	p.(His1118Tyr)	Missense	Pathogenic	M	60	Any other ethnic group
13688541	619	ABCA4	NM_000350.3	c.3352C>T	p.(His1118Tyr)	Missense	Pathogenic	M	60	Any other ethnic group
15763768	620	ABCA4	NM_000350.3	c.4577C>T	p.(Thr1526Met)	Missense	Pathogenic	M	19	Not stated
15763768	620	ABCA4	NM_000350.3	c.4670A>G	p.(Tyr1557Cys)	Missense	Likely Pathogenic	M	19	Not stated
15763768	620	ABCA4	NM_000350.3	c.6148G>C	p.(Val2050Leu)	Missense	Variant of Uncertain Significance	M	19	Not stated
8634884	621	ABCA4	NM_000350.3	c.5882G>A	p.(Gly1961Glu)	Missense	Pathogenic	M	36	Any other ethnic group
8634884	621	ABCA4	NM_000350.3	c.5882G>A	p.(Gly1961Glu)	Missense	Pathogenic	M	36	Any other ethnic group
8634884	621	ABCA4	NM_000350.3	c.634C>T	p.(Arg212Cys)	Missense	Pathogenic	M	36	Any other ethnic group
13529620	622	ABCA4	NM_000350.3	c.1317G>A	p.(Trp439Ter)	Stopgain	Pathogenic	M	75	White - British
13529620	622	ABCA4	NM_000350.3	c.5908C>T	p.(Leu1970Phe)	Missense	Variant of Uncertain Significance	M	75	White - British
15810913	623	ABCA4	NM_000350.3	c.1906C>T	p.(Gln636Ter)	Stopgain	Pathogenic	F	28	White - British
15810913	623	ABCA4	NM_000350.3	c.4328G>A	p.(Arg1443His)	Missense	Pathogenic	F	28	White - British
15760695	624	ABCA4	NM_000350.3	c.2396C>T	p.(Pro799Leu)	Missense	Likely Pathogenic	M	71	Not stated
15760695	624	ABCA4	NM_000350.3	c.6658C>T	p.(Gln2220Ter)	Stopgain	Pathogenic	M	71	Not stated
15727116	625	ABCA4	NM_000350.3	c.1928T>G	p.(Val643Gly)	Missense	Likely Pathogenic	F	57	Not stated
15727116	625	ABCA4	NM_000350.3	c.2560G>T	p.(Ala854Ser)	Missense	Variant of Uncertain Significance	F	57	Not stated
15727116	625	ABCA4	NM_000350.3	c.4222T>C	p.(Trp1408Arg)	Missense	Pathogenic	F	57	Not stated
15727116	625	ABCA4	NM_000350.3	c.4918C>T	p.(Arg1640Trp)	Missense	Pathogenic	F	57	Not stated
15794953	626	ABCA4	NM_000350.3	c.1648G>A	p.(Gly550Arg)	Missense	Pathogenic	M	29	White - British
15794953	626	ABCA4	NM_000350.3	c.6416G>C	p.(Arg2139Pro)	Missense	Pathogenic	M	29	White - British
15477706	627	ABCA4	NM_000350.3	c.2588G>C	p.(Gly863Ala)	Missense	Pathogenic	M	38	White - British
15477706	627	ABCA4	NM_000350.3	c.6352del	p.(Arg2118GlufsTer27)	Frameshift	Likely Pathogenic	M	38	White - British
15592555	628	ABCA4	NM_000350.3	c.3386G>T	p.(Arg1129Leu)	Missense	Pathogenic	M	54	Not stated
15592555	628	ABCA4	NM_000350.3	c.5044_5058del	p.(Val1682_Val1686del)	Inframe indel	Pathogenic	M	54	Not stated
15539572	629	ABCA4	NM_000350.3	c.5882G>A	p.(Gly1961Glu)	Missense	Pathogenic	F	31	Any other ethnic group
15539572	629	ABCA4	NM_000350.3	c.634C>T	p.(Arg212Cys)	Missense	Pathogenic	F	31	Any other ethnic group
15801799	630	ABCA4	NM_000350.3	c.1622T>C	p.(Leu541Pro)	Missense	Pathogenic	F	43	White - Other

15801799	630	ABCA4	NM_000350.3	c.3113C>T	p.(Ala1038Val)	Missense	Pathogenic	F	43	White - Other
15801799	630	ABCA4	NM_000350.3	c.5882G>A	p.(Gly1961Glu)	Missense	Pathogenic	F	43	White - Other
15825074	631	ABCA4	NM_000350.3	c.1253T>C	p.(Phe418Ser)	Missense	Pathogenic	M	17	Mixed - Other
15825074	631	ABCA4	NM_000350.3	c.4773+1G>T	Splice	Splice	Pathogenic	M	17	Mixed - Other
15804837	632	ABCA4	NM_000350.3	c.3113C>T	p.(Ala1038Val)	Missense	Pathogenic	M	31	Not stated
15804837	632	ABCA4	NM_000350.3	c.4978C>T	p.(Pro1660Ser)	Missense	Pathogenic	M	31	Not stated
15718037	633	ABCA4	NM_000350.3	c.5882G>A	p.(Gly1961Glu)	Missense	Pathogenic	F	55	Not stated
15718037	633	ABCA4	NM_000350.3	c.6088C>T	p.(Arg2030Ter)	Stopgain	Pathogenic	F	55	Not stated
7606367	634	ABCA4	NM_000350.3	c.4538A>G	p.(Gln1513Arg)	Missense	Pathogenic	F	59	Black - Caribbean
7606367	634	ABCA4	NM_000350.3	c.6320G>A	p.(Arg2107His)	Missense	Pathogenic	F	59	Black - Caribbean
15877609	635	ABCA4	NM_000350.3	c.4469G>A	p.(Cys1490Tyr)	Missense	Pathogenic	F	25	Not stated
15877609	635	ABCA4	NM_000350.3	c.5051T>A	p.(Ile1684Asn)	Missense	Variant of Uncertain Significance	F	25	Not stated
11001871	636	ABCA4	NM_000350.3	c.4234C>T	p.(Gln1412Ter)	Stopgain	Pathogenic	M	35	Not stated
11001871	636	ABCA4	NM_000350.3	c.4919G>A	p.(Arg1640Gln)	Missense	Pathogenic	M	35	Not stated
15886583	637	ABCA4	NM_000350.3	c.6098T>G	p.(Leu2033Arg)	Missense	Likely Pathogenic	M	38	Any other ethnic group
15886583	637	ABCA4	NM_000350.3	c.763C>T	p.(Arg255Cys)	Missense	Pathogenic	M	38	Any other ethnic group
12191318	638	ABCA4	NM_000350.3	c.619G>A	p.(Glu207Lys)	Missense	Variant of Uncertain Significance	M	62	Black - African
12191318	638	ABCA4	NM_000350.3	c.6320G>A	p.(Arg2107His)	Missense	Pathogenic	M	62	Black - African
15599870	639	ABCA4	NM_000350.3	c.5196+1G>A	Splice	Splice	Pathogenic	F	57	Not stated
15599870	639	ABCA4	NM_000350.3	c.5882G>A	p.(Gly1961Glu)	Missense	Pathogenic	F	57	Not stated
13973210	640	ABCA4	NM_000350.3	c.331_332del	p.(Glu111ThrfsTer49)	Frameshift	Pathogenic	F	18	Not stated
13973210	640	ABCA4	NM_000350.3	c.5461-10T>C	Splice	Splice	Pathogenic	F	18	Not stated
9180737	641	ABCA4	NM_000350.3	c.4098dup	p.(Ile1367HisfsTer55)	Frameshift	Likely Pathogenic	M	31	Any other ethnic group
9180737	641	ABCA4	NM_000350.3	c.5882G>A	p.(Gly1961Glu)	Missense	Pathogenic	M	31	Any other ethnic group
12124125	642	ABCA4	NM_000350.3	c.5882G>A	p.(Gly1961Glu)	Missense	Pathogenic	M	53	White - British
12124125	642	ABCA4	NM_000350.3	c.5882G>A	p.(Gly1961Glu)	Missense	Pathogenic	M	53	White - British
12124125	642	ABCA4	NM_000350.3	c.634C>T	p.(Arg212Cys)	Missense	Pathogenic	M	53	White - British
10604985	643	ABCA4	NM_000350.3	c.2588G>C	p.(Gly863Ala)	Missense	Pathogenic	F	34	White - British
10604985	643	ABCA4	NM_000350.3	c.6449G>A	p.(Cys2150Tyr)	Missense	Pathogenic	F	34	White - British
14839544	644	ABCA4	NM_000350.3	c.1817G>A	p.(Gly606Asp)	Missense	Pathogenic	F	24	White - British
14839544	644	ABCA4	NM_000350.3	c.5714+5G>A	Splice	Splice	Pathogenic	F	24	White - British
15323538	645	ABCA4	NM_000350.3	c.2588G>C	p.(Gly863Ala)	Missense	Pathogenic	F	60	Unknown
15323538	645	ABCA4	NM_000350.3	c.2827C>T	p.(Arg943Trp)	Missense	Pathogenic	F	60	Unknown
15582951	646	ABCA4	NM_000350.3	c.5603A>T	p.(Asn1868Ile)	Missense	Variant of Uncertain Significance	M	42	White - Other
15582951	646	ABCA4	NM_000350.3	c.768G>T	p.(Val256Val)	Synonymous	Pathogenic	M	42	White - Other
15847691	647	ABCA4	NM_000350.3	c.6729+5_6729+19del	Splice	Splice	Likely Pathogenic	M	13	Asian - Pakistani
15847691	647	ABCA4	NM_000350.3	c.6729+5_6729+19del	Splice	Splice	Likely Pathogenic	M	13	Asian - Pakistani
15873808	648	ABCA4	NM_000350.3	c.2041C>T	p.(Arg681Ter)	Stopgain	Pathogenic	M	53	Not stated
15873808	648	ABCA4	NM_000350.3	c.3220A>G	p.(Ile1074Val)	Missense	Variant of Uncertain Significance	M	53	Not stated
16083647	649	ABCA4	NM_000350.3	c.1957C>T	p.(Arg653Cys)	Missense	Pathogenic	F	16	White - British
16083647	649	ABCA4	NM_000350.3	c.1957C>T	p.(Arg653Cys)	Missense	Pathogenic	F	16	White - British
15966278	650	ABCA4	NM_000350.3	c.1804C>T	p.(Arg602Trp)	Missense	Pathogenic	F	34	White - Other
15966278	650	ABCA4	NM_000350.3	c.6112C>T	p.(Arg2038Trp)	Missense	Pathogenic	F	34	White - Other
15300172	651	ABCA4	NM_000350.3	c.3064G>A	p.(Glu1022Lys)	Missense	Pathogenic	M	36	Mixed - White and Asian
15300172	651	ABCA4	NM_000350.3	c.3064G>A	p.(Glu1022Lys)	Missense	Pathogenic	M	36	Mixed - White and Asian
17799662	651	ABCA4	NM_000350.3	c.3064G>A	p.(Glu1022Lys)	Missense	Pathogenic	M	41	Not stated
17799662	651	ABCA4	NM_000350.3	c.3064G>A	p.(Glu1022Lys)	Missense	Pathogenic	M	41	Not stated
3664667	652	ABCA4	NM_000350.3	c.4577C>T	p.(Thr1526Met)	Missense	Pathogenic	F	42	Not stated
3664667	652	ABCA4	NM_000350.3	c.4577C>T	p.(Thr1526Met)	Missense	Pathogenic	F	42	Not stated
15500617	653	ABCA4	NM_000350.3	c.5882G>A	p.(Gly1961Glu)	Missense	Pathogenic	F	56	Not stated
15500617	653	ABCA4	NM_000350.3	c.5917del	p.(Val1973Ter)	Stopgain	Pathogenic	F	56	Not stated

15378859	654	ABCA4	NM_000350.3	c.1856T>A	p.(Ile619Asn)	Missense	Variant of Uncertain Significance	F	29	White - British
15378859	654	ABCA4	NM_000350.3	c.2588G>C	p.(Gly863Ala)	Missense	Pathogenic	F	29	White - British
11778794	655	ABCA4	NM_000350.3	c.5693G>A	p.(Arg1898His)	Missense	Likely Pathogenic	F	47	White - British
11778794	655	ABCA4	NM_000350.3	c.5882G>A	p.(Gly1961Glu)	Missense	Pathogenic	F	47	White - British
1530171	656	ABCA4	NM_000350.3	c.2588G>C	p.(Gly863Ala)	Missense	Pathogenic	M	69	White - British
1530171	656	ABCA4	NM_000350.3	c.5461-10T>C	Splice	Splice	Pathogenic	M	69	White - British
155833	657	ABCA4	NM_000350.3	c.161G>A	p.(Cys54Tyr)	Missense	Pathogenic	F	63	White - British
155833	657	ABCA4	NM_000350.3	c.2588G>C	p.(Gly863Ala)	Missense	Pathogenic	F	63	White - British
16009447	658	ABCA4	NM_000350.3	c.5882G>A	p.(Gly1961Glu)	Missense	Pathogenic	F	41	Not stated
16009447	658	ABCA4	NM_000350.3	c.6729+5_6729+19del	Splice	Splice	Likely Pathogenic	F	41	Not stated
16043768	659	ABCA4	NM_000350.3	c.3322C>T	p.(Arg1108Cys)	Missense	Pathogenic	F	23	Not stated
16043768	659	ABCA4	NM_000350.3	c.6286G>A	p.(Glu2096Lys)	Missense	Pathogenic	F	23	Not stated
16070207	660	ABCA4	NM_000350.3	c.1760+3G>C	Splice	Splice	Variant of Uncertain Significance	F	26	Not stated
16070207	660	ABCA4	NM_000350.3	c.6352del	p.(Arg2118GlufsTer27)	Frameshift	Likely Pathogenic	F	26	Not stated
13466018	661	ABCA4	NM_000350.3	c.2023G>A	p.(Val675Ile)	Missense	Pathogenic	M	46	Not stated
13466018	661	ABCA4	NM_000350.3	c.5882G>A	p.(Gly1961Glu)	Missense	Pathogenic	M	46	Not stated
13166516	662	ABCA4	NM_000350.3	c.5882G>A	p.(Gly1961Glu)	Missense	Pathogenic	F	31	Any other ethnic group
13166516	662	ABCA4	NM_000350.3	c.6229C>T	p.(Arg2077Trp)	Missense	Pathogenic	F	31	Any other ethnic group
2355961	663	ABCA4	NM_000350.3	c.5413A>G	p.(Asn1805Asp)	Missense	Pathogenic	M	82	White - Other
2355961	663	ABCA4	NM_000350.3	c.5714+5G>A	Splice	Splice	Pathogenic	M	82	White - Other
16065412	664	ABCA4	NM_000350.3	c.53G>A	p.(Arg18Gln)	Missense	Pathogenic	M	41	Not stated
16065412	664	ABCA4	NM_000350.3	c.5882G>A	p.(Gly1961Glu)	Missense	Pathogenic	M	41	Not stated
16065356	665	ABCA4	NM_000350.3	c.4363T>C	p.(Cys1455Arg)	Missense	Pathogenic	M	37	White - British
16065356	665	ABCA4	NM_000350.3	c.5313-3C>G	Splice	Splice	Variant of Uncertain Significance	M	37	White - British
16168116	666	ABCA4	NM_000350.3	c.5196+1056A>G	Splice	Splice	Likely Pathogenic	M	10	Not stated
16168116	666	ABCA4	NM_000350.3	c.5882G>A	p.(Gly1961Glu)	Missense	Pathogenic	M	10	Not stated
8550681	667	ABCA4	NM_000350.3	c.2161-6T>C	Splice	Splice	Variant of Uncertain Significance	M	69	Black - Other
8550681	667	ABCA4	NM_000350.3	c.6320G>A	p.(Arg2107His)	Missense	Pathogenic	M	69	Black - Other
16227287	668	ABCA4	NM_000350.3	c.3064G>A	p.(Glu1022Lys)	Missense	Pathogenic	F	54	Asian - Indian
16227287	668	ABCA4	NM_000350.3	c.5882G>A	p.(Gly1961Glu)	Missense	Pathogenic	F	54	Asian - Indian
16135825	669	ABCA4	NM_000350.3	c.5882G>A	p.(Gly1961Glu)	Missense	Pathogenic	F	30	Not stated
16135825	669	ABCA4	NM_000350.3	c.5908C>T	p.(Leu1970Phe)	Missense	Variant of Uncertain Significance	F	30	Not stated
16135825	669	ABCA4	NM_000350.3	c.6598del	p.(Glu2200ArgfsTer47)	Frameshift	Likely Pathogenic	F	30	Not stated
14827357	670	ABCA4	NM_000350.3	c.3292C>T	p.(Arg1098Cys)	Missense	Pathogenic	M	48	White - Other
14827357	670	ABCA4	NM_000350.3	c.5714+5G>A	Splice	Splice	Pathogenic	M	48	White - Other
16117450	671	ABCA4	NM_000350.3	c.4139C>T	p.(Pro1380Leu)	Missense	Pathogenic	M	45	Any other ethnic group
16117450	671	ABCA4	NM_000350.3	c.5882G>A	p.(Gly1961Glu)	Missense	Pathogenic	M	45	Any other ethnic group
16117450	671	ABCA4	NM_000350.3	c.6647C>T	p.(Ala2216Val)	Missense	Likely Pathogenic	M	45	Any other ethnic group
13303268	672	ABCA4	NM_000350.3	c.4064T>A	p.(Val1355Glu)	Missense	Likely Pathogenic	M	36	Any other ethnic group
13303268	672	ABCA4	NM_000350.3	c.5318C>T	p.(Ala1773Val)	Missense	Pathogenic	M	36	Any other ethnic group
16203494	673	ABCA4	NM_000350.3	c.4469G>A	p.(Cys1490Tyr)	Missense	Pathogenic	M	62	White - British
16203494	673	ABCA4	NM_000350.3	c.5603A>T	p.(Asn1868Ile)	Missense	Variant of Uncertain Significance	M	62	White - British
16203494	673	ABCA4	NM_000350.3	c.5603A>T	p.(Asn1868Ile)	Missense	Variant of Uncertain Significance	M	62	White - British
16147186	674	ABCA4	NM_000350.3	c.4462T>C	p.(Cys1488Arg)	Missense	Pathogenic	M	50	Asian - Bangladeshi
16147186	674	ABCA4	NM_000350.3	c.6112C>T	p.(Arg2038Trp)	Missense	Pathogenic	M	50	Asian - Bangladeshi
16208933	675	ABCA4	NM_000350.3	c.3322C>T	p.(Arg1108Cys)	Missense	Pathogenic	M	32	White - British
16208933	675	ABCA4	NM_000350.3	c.3482G>A	p.(Arg1161His)	Missense	Pathogenic	M	32	White - British
12329428	676	ABCA4	NM_000350.3	c.1293G>T	p.(Trp431Cys)	Missense	Likely Pathogenic	M	57	White - British
12329428	676	ABCA4	NM_000350.3	c.4253+43G>A	Splice	Splice	Likely Pathogenic	M	57	White - British
12329428	676	ABCA4	NM_000350.3	c.5603A>T	p.(Asn1868Ile)	Missense	Variant of Uncertain Significance	M	57	White - British
16278597	677	ABCA4	NM_000350.3	c.5714+5G>A	Splice	Splice	Pathogenic	F	56	White - British

16278597	677	ABCA4	NM_000350.3	c.5714+5G>A	Splice	Splice	Pathogenic	F	56	White - British
17783464	678	ABCA4	NM_000350.3	c.3064G>A	p.(Glu1022Lys)	Missense	Pathogenic	M	14	White - Other
17783464	678	ABCA4	NM_000350.3	c.3064G>A	p.(Glu1022Lys)	Missense	Pathogenic	M	14	White - Other
14919029	679	ABCA4	NM_000350.3	c.2971G>C	p.(Gly991Arg)	Missense	Pathogenic	M	53	Black - African
14919029	679	ABCA4	NM_000350.3	c.4537dup	p.(Gln1513ProfsTer42)	Frameshift	Pathogenic	M	53	Black - African
7000734	680	ABCA4	NM_000350.3	c.4139C>T	p.(Pro1380Leu)	Missense	Pathogenic	F	51	Asian - Indian
7000734	680	ABCA4	NM_000350.3	c.5882G>A	p.(Gly1961Glu)	Missense	Pathogenic	F	51	Asian - Indian
13314482	681	ABCA4	NM_000350.3	c.3212C>T	p.(Ser1071Leu)	Missense	Pathogenic	F	39	Not stated
13314482	681	ABCA4	NM_000350.3	c.4663C>T	p.(Gln1555Ter)	Stopgain	Likely Pathogenic	F	39	Not stated
13314482	681	ABCA4	NM_000350.3	c.6694G>A	p.(Glu2232Lys)	Missense	Variant of Uncertain Significance	F	39	Not stated
15682659	682	ABCA4	NM_000350.3	c.5461-10T>C	Splice	Splice	Pathogenic	M	62	White - British
15682659	682	ABCA4	NM_000350.3	c.5603A>T	p.(Asn1868Ile)	Missense	Variant of Uncertain Significance	M	62	White - British
15682659	682	ABCA4	NM_000350.3	c.5603A>T	p.(Asn1868Ile)	Missense	Variant of Uncertain Significance	M	62	White - British
6813778	683	ABCA4	NM_000350.3	c.5882G>A	p.(Gly1961Glu)	Missense	Pathogenic	M	46	Asian - Other
6813778	683	ABCA4	NM_000350.3	c.858+1G>T	Splice	Splice	Likely Pathogenic	M	46	Asian - Other
16398934	684	ABCA4	NM_000350.3	c.4948G>T	p.(Glu1650Ter)	Stopgain	Likely Pathogenic	F	42	Not stated
16398934	684	ABCA4	NM_000350.3	c.5882G>A	p.(Gly1961Glu)	Missense	Pathogenic	F	42	Not stated
6667142	685	ABCA4	NM_000350.3	c.5882G>A	p.(Gly1961Glu)	Missense	Pathogenic	F	58	White - Other
6667142	685	ABCA4	NM_000350.3	c.5018+2T>C	Splice	Splice	Pathogenic	F	58	White - Other
16409378	686	ABCA4	NM_000350.3	c.1715G>A	p.(Arg572Gln)	Missense	Likely Pathogenic	F	27	White - British
16409378	686	ABCA4	NM_000350.3	c.2588G>C	p.(Gly863Ala)	Missense	Pathogenic	F	27	White - British
16409378	686	ABCA4	NM_000350.3	c.4139C>T	p.(Pro1380Leu)	Missense	Pathogenic	F	27	White - British
6092645	687	ABCA4	NM_000350.3	c.1906C>T	p.(Gln636Ter)	Stopgain	Pathogenic	M	63	White - British
6092645	687	ABCA4	NM_000350.3	c.67-2023T>G	Splice	Splice	Variant of Uncertain Significance	M	63	White - British
16393306	688	ABCA4	NM_000350.3	c.5882G>A	p.(Gly1961Glu)	Missense	Pathogenic	M	49	Not stated
16393306	688	ABCA4	NM_000350.3	c.6449G>A	p.(Cys2150Tyr)	Missense	Pathogenic	M	49	Not stated
17915400	688	ABCA4	NM_000350.3	c.5882G>A	p.(Gly1961Glu)	Missense	Pathogenic	F	52	Unknown
17915400	688	ABCA4	NM_000350.3	c.6449G>A	p.(Cys2150Tyr)	Missense	Pathogenic	F	52	Unknown
16166373	689	ABCA4	NM_000350.3	c.5882G>A	p.(Gly1961Glu)	Missense	Pathogenic	F	34	Asian - Bangladeshi
16166373	689	ABCA4	NM_000350.3	c.6479+1G>A	Splice	Splice	Likely Pathogenic	F	34	Asian - Bangladeshi
10330767	690	ABCA4	NM_000350.3	c.4139C>T	p.(Pro1380Leu)	Missense	Pathogenic	F	70	White - Other
10330767	690	ABCA4	NM_000350.3	c.5882G>A	p.(Gly1961Glu)	Missense	Pathogenic	F	70	White - Other
15573263	691	ABCA4	NM_000350.3	c.1622T>C	p.(Leu541Pro)	Missense	Pathogenic	M	71	White - Other
15573263	691	ABCA4	NM_000350.3	c.3113C>T	p.(Ala1038Val)	Missense	Pathogenic	M	71	White - Other
15573263	691	ABCA4	NM_000350.3	c.5882G>A	p.(Gly1961Glu)	Missense	Pathogenic	M	71	White - Other
13261611	692	ABCA4	NM_000350.3	c.3064G>A	p.(Glu1022Lys)	Missense	Pathogenic	M	37	Not stated
13261611	692	ABCA4	NM_000350.3	c.3064G>A	p.(Glu1022Lys)	Missense	Pathogenic	M	37	Not stated
16125171	693	ABCA4	NM_000350.3	c.4253+5G>T	Splice	Splice	Likely Pathogenic	F	36	Any other ethnic group
16125171	693	ABCA4	NM_000350.3	c.5882G>A	p.(Gly1961Glu)	Missense	Pathogenic	F	36	Any other ethnic group
16303342	694	ABCA4	NM_000350.3	c.1804C>T	p.(Arg602Trp)	Missense	Pathogenic	M	12	Not stated
16303342	694	ABCA4	NM_000350.3	c.5196+1G>A	Splice	Splice	Pathogenic	M	12	Not stated
16758342	695	ABCA4	NM_000350.3	c.5882G>A	p.(Gly1961Glu)	Missense	Pathogenic	F	31	White - British
16758342	695	ABCA4	NM_000350.3	c.5933_5943dup	p.(Phe1982LysfsTer14)	Frameshift	Likely Pathogenic	F	31	White - British
16586765	696	ABCA4	NM_000350.3	c.1622T>C	p.(Leu541Pro)	Missense	Pathogenic	F	36	Not stated
16586765	696	ABCA4	NM_000350.3	c.2588G>C	p.(Gly863Ala)	Missense	Pathogenic	F	36	Not stated
16586765	696	ABCA4	NM_000350.3	c.3113C>T	p.(Ala1038Val)	Missense	Pathogenic	F	36	Not stated
16762003	697	ABCA4	NM_000350.3	c.3056C>T	p.(Thr1019Met)	Missense	Pathogenic	M	19	Any other ethnic group
16762003	697	ABCA4	NM_000350.3	c.4326C>A	p.(Asn1442Lys)	Missense	Likely Pathogenic	M	19	Any other ethnic group
15848622	698	ABCA4	NM_000350.3	c.3210_3211dup	p.(Ser1071CysfsTer14)	Frameshift	Pathogenic	F	32	White - British
15848622	698	ABCA4	NM_000350.3	c.6320G>A	p.(Arg2107His)	Missense	Pathogenic	F	32	White - British
16741073	699	ABCA4	NM_000350.3	c.4577C>T	p.(Thr1526Met)	Missense	Pathogenic	F	45	White - Other

16741073	699	ABCA4	NM_000350.3	c.5882G>A	p.(Gly1961Glu)	Missense	Pathogenic	F	45	White - Other
16861074	700	ABCA4	NM_000350.3	c.4222T>C	p.(Trp1408Arg)	Missense	Pathogenic	F	28	Not stated
16861074	700	ABCA4	NM_000350.3	c.4539+2028C>T	Splice	Splice	Likely Pathogenic	F	28	Not stated
16861074	700	ABCA4	NM_000350.3	c.4918C>T	p.(Arg1640Trp)	Missense	Pathogenic	F	28	Not stated
11735793	701	ABCA4	NM_000350.3	c.5882G>A	p.(Gly1961Glu)	Missense	Pathogenic	F	46	Black - African
11735793	701	ABCA4	NM_000350.3	c.5882G>A	p.(Gly1961Glu)	Missense	Pathogenic	F	46	Black - African
11735793	701	ABCA4	NM_000350.3	c.634C>T	p.(Arg212Cys)	Missense	Pathogenic	F	46	Black - African
16767603	702	ABCA4	NM_000350.3	c.2297G>A	p.(Gly766Asp)	Missense	Likely Pathogenic	F	47	White - British
16767603	702	ABCA4	NM_000350.3	c.5882G>A	p.(Gly1961Glu)	Missense	Pathogenic	F	47	White - British
7848252	703	ABCA4	NM_000350.3	c.5313-1_5313del	Splice	Splice	Likely Pathogenic	F	46	Asian - Pakistani
7848252	703	ABCA4	NM_000350.3	c.5882G>A	p.(Gly1961Glu)	Missense	Pathogenic	F	46	Asian - Pakistani
3317082	704	ABCA4	NM_000350.3	c.2063A>T	p.(Asn688Ile)	Missense	Variant of Uncertain Significance	M	52	Any other ethnic group
3317082	704	ABCA4	NM_000350.3	c.5461-10T>C	Splice	Splice	Pathogenic	M	52	Any other ethnic group
3317082	704	ABCA4	NM_000350.3	c.5461-10T>C	Splice	Splice	Pathogenic	M	52	Any other ethnic group
16738763	705	ABCA4	NM_000350.3	c.5461-10T>C	Splice	Splice	Pathogenic	F	76	Not stated
16738763	705	ABCA4	NM_000350.3	c.5516T>C	p.(Phe1839Ser)	Missense	Likely Pathogenic	F	76	Not stated
16738763	705	ABCA4	NM_000350.3	c.466A>G	p.(Ile156Val)	Missense	Variant of Uncertain Significance	F	76	Not stated
16738763	705	ABCA4	NM_000350.3	c.5603A>T	p.(Asn1868Ile)	Missense	Variant of Uncertain Significance	F	76	Not stated
10515378	706	ABCA4	NM_000350.3	c.2654G>A	p.(Gly885Glu)	Missense	Variant of Uncertain Significance	M	26	Any other ethnic group
10515378	706	ABCA4	NM_000350.3	c.2897G>A	p.(Gly966Glu)	Missense	Pathogenic	M	26	Any other ethnic group
16759266	707	ABCA4	NM_000350.3	c.5312+1G>A	Splice	Splice	Pathogenic	M	38	White - Other
16759266	707	ABCA4	NM_000350.3	c.5312+1G>A	Splice	Splice	Pathogenic	M	38	White - Other
16767911	708	ABCA4	NM_000350.3	c.1239+1G>A	Splice	Splice	Likely Pathogenic	F	20	Not stated
16767911	708	ABCA4	NM_000350.3	c.6317G>A	p.(Arg2106His)	Missense	Pathogenic	F	20	Not stated
16287410	709	ABCA4	NM_000350.3	c.1460G>A	p.(Arg487Gln)	Missense	Variant of Uncertain Significance	M	54	Not stated
16287410	709	ABCA4	NM_000350.3	c.3064G>A	p.(Glu1022Lys)	Missense	Pathogenic	M	54	Not stated
16287410	709	ABCA4	NM_000350.3	c.6694G>A	p.(Glu2232Lys)	Missense	Variant of Uncertain Significance	M	54	Not stated
13580580	710	ABCA4	NM_000350.3	c.1622T>C	p.(Leu541Pro)	Missense	Pathogenic	F	44	White - Other
13580580	710	ABCA4	NM_000350.3	c.3113C>T	p.(Ala1038Val)	Missense	Pathogenic	F	44	White - Other
13580580	710	ABCA4	NM_000350.3	c.5603A>T	p.(Asn1868Ile)	Missense	Variant of Uncertain Significance	F	44	White - Other
16909948	711	ABCA4	NM_000350.3	c.3364G>A	p.(Glu1122Lys)	Missense	Pathogenic	F	41	Asian - Other
16909948	711	ABCA4	NM_000350.3	c.5882G>A	p.(Gly1961Glu)	Missense	Pathogenic	F	41	Asian - Other
16810695	712	ABCA4	NM_000350.3	c.1622T>C	p.(Leu541Pro)	Missense	Pathogenic	F	45	Any other ethnic group
16810695	712	ABCA4	NM_000350.3	c.3113C>T	p.(Ala1038Val)	Missense	Pathogenic	F	45	Any other ethnic group
16810695	712	ABCA4	NM_000350.3	c.5882G>A	p.(Gly1961Glu)	Missense	Pathogenic	F	45	Any other ethnic group
12605627	713	ABCA4	NM_000350.3	c.5882G>A	p.(Gly1961Glu)	Missense	Pathogenic	F	40	Asian - Indian
12605627	713	ABCA4	NM_000350.3	c.5905G>A	p.(Gly1969Ser)	Missense	Likely Pathogenic	F	40	Asian - Indian
15255477	714	ABCA4	NM_000350.3	c.2930C>T	p.(Thr977Met)	Missense	Pathogenic	M	54	Not stated
15255477	714	ABCA4	NM_000350.3	c.2966T>C	p.(Val989Ala)	Missense	Pathogenic	M	54	Not stated
11505857	715	ABCA4	NM_000350.3	c.2588G>C	p.(Gly863Ala)	Missense	Pathogenic	M	30	White - British
11505857	715	ABCA4	NM_000350.3	c.4234C>T	p.(Gln1412Ter)	Stopgain	Pathogenic	M	30	White - British
15237676	716	ABCA4	NM_000350.3	c.5316G>A	p.(Trp1772Ter)	Stopgain	Pathogenic	M	54	White - Other
15237676	716	ABCA4	NM_000350.3	c.5603A>T	p.(Asn1868Ile)	Missense	Variant of Uncertain Significance	M	54	White - Other
16601633	717	ABCA4	NM_000350.3	c.3482G>A	p.(Arg1161His)	Missense	Pathogenic	F	26	White - British
16601633	717	ABCA4	NM_000350.3	c.4469G>A	p.(Cys1490Tyr)	Missense	Pathogenic	F	26	White - British
16698135	718	ABCA4	NM_000350.3	c.1343T>A	p.(Met448Lys)	Missense	Pathogenic	M	61	Asian - Indian
16698135	718	ABCA4	NM_000350.3	c.6305A>G	p.(Asp2102Gly)	Missense	Likely Pathogenic	M	61	Asian - Indian
8248414	719	ABCA4	NM_000350.3	c.4139C>T	p.(Pro1380Leu)	Missense	Pathogenic	F	55	Not stated
8248414	719	ABCA4	NM_000350.3	c.5603A>T	p.(Asn1868Ile)	Missense	Variant of Uncertain Significance	F	55	Not stated
15921149	720	ABCA4	NM_000350.3	c.3050+34C>T	Splice	Splice	Variant of Uncertain Significance	M	52	Black - Other
15921149	720	ABCA4	NM_000350.3	c.3821T>C	p.(Leu1274Pro)	Missense	Likely Pathogenic	M	52	Black - Other

13150150	721	ABCA4	NM_000350.3	c.2971G>C	p.(Gly991Arg)	Missense	Pathogenic	M	37	Black - Other
13150150	721	ABCA4	NM_000350.3	c.5519G>T	p.(Cys1840Phe)	Missense	Likely Pathogenic	M	37	Black - Other
16904691	722	ABCA4	NM_000350.3	c.1957C>T	p.(Arg653Cys)	Missense	Pathogenic	M	51	White - British
16904691	722	ABCA4	NM_000350.3	c.5603A>T	p.(Asn1868Ile)	Missense	Variant of Uncertain Significance	M	51	White - British
17013835	723	ABCA4	NM_000350.3	c.4457C>T	p.(Pro1486Leu)	Missense	Pathogenic	F	31	White - Other
17013835	723	ABCA4	NM_000350.3	c.5316G>A	p.(Trp1772Ter)	Stopgain	Pathogenic	F	31	White - Other
17046035	724	ABCA4	NM_000350.3	c.1622T>C	p.(Leu541Pro)	Missense	Pathogenic	F	56	Not stated
17046035	724	ABCA4	NM_000350.3	c.3113C>T	p.(Ala1038Val)	Missense	Pathogenic	F	56	Not stated
17046035	724	ABCA4	NM_000350.3	c.5018+5G>A	Splice	Splice	Pathogenic	F	56	Not stated
17077710	725	ABCA4	NM_000350.3	c.2588G>C	p.(Gly863Ala)	Missense	Pathogenic	F	52	White - British
17077710	725	ABCA4	NM_000350.3	c.3814-3C>A	Splice	Splice	Variant of Uncertain Significance	F	52	White - British
17051292	726	ABCA4	NM_000350.3	c.1381A>T	p.(Lys461Ter)	Stopgain	Likely Pathogenic	F	27	Not stated
17051292	726	ABCA4	NM_000350.3	c.2588G>C	p.(Gly863Ala)	Missense	Pathogenic	F	27	Not stated
17001501	727	ABCA4	NM_000350.3	c.5461-10T>C	Splice	Splice	Pathogenic	F	63	Not stated
17001501	727	ABCA4	NM_000350.3	c.5603A>T	p.(Asn1868Ile)	Missense	Variant of Uncertain Significance	F	63	Not stated
17001501	727	ABCA4	NM_000350.3	c.5603A>T	p.(Asn1868Ile)	Missense	Variant of Uncertain Significance	F	63	Not stated
6477078	728	ABCA4	NM_000350.3	c.2915C>A	p.(Thr972Asn)	Missense	Pathogenic	M	32	White - British
6477078	728	ABCA4	NM_000350.3	c.4918C>T	p.(Arg1640Trp)	Missense	Pathogenic	M	32	White - British
17023383	729	ABCA4	NM_000350.3	c.3289A>T	p.(Arg1097Ter)	Stopgain	Pathogenic	M	66	Any other ethnic group
17023383	729	ABCA4	NM_000350.3	c.4793C>A	p.(Ala1598Asp)	Missense	Pathogenic	M	66	Any other ethnic group
17066433	730	ABCA4	NM_000350.3	c.5882G>A	p.(Gly1961Glu)	Missense	Pathogenic	F	39	White - Other
17066433	730	ABCA4	NM_000350.3	c.5917del	p.(Val1973Ter)	Stopgain	Pathogenic	F	39	White - Other
17193805	731	ABCA4	NM_000350.3	c.4124C>A	p.(Ala1375Glu)	Missense	Likely Pathogenic	F	41	Asian - Indian
17193805	731	ABCA4	NM_000350.3	c.5882G>A	p.(Gly1961Glu)	Missense	Pathogenic	F	41	Asian - Indian
17232648	732	ABCA4	NM_000350.3	c.2564G>A	p.(Trp855Ter)	Stopgain	Pathogenic	M	18	Not stated
17232648	732	ABCA4	NM_000350.3	c.5196+1137G>A	Splice	Splice	Pathogenic	M	18	Not stated
17125520	733	ABCA4	NM_000350.3	c.6089G>A	p.(Arg2030Gln)	Missense	Pathogenic	F	35	White - British
17125520	733	ABCA4	NM_000350.3	c.5018+2T>C	Splice	Splice	Pathogenic	F	35	White - British
11314211	734	ABCA4	NM_000350.3	c.2966T>C	p.(Val989Ala)	Missense	Pathogenic	F	27	Black - African
11314211	734	ABCA4	NM_000350.3	c.3259G>A	p.(Glu1087Lys)	Missense	Pathogenic	F	27	Black - African
11236651	735	ABCA4	NM_000350.3	c.1622T>C	p.(Leu541Pro)	Missense	Pathogenic	F	80	White - Irish
11236651	735	ABCA4	NM_000350.3	c.3113C>T	p.(Ala1038Val)	Missense	Pathogenic	F	80	White - Irish
11236651	735	ABCA4	NM_000350.3	c.4253+43G>A	Splice	Splice	Likely Pathogenic	F	80	White - Irish
16641015	736	ABCA4	NM_000350.3	c.1622T>C	p.(Leu541Pro)	Missense	Pathogenic	M	32	Any other ethnic group
16641015	736	ABCA4	NM_000350.3	c.3113C>T	p.(Ala1038Val)	Missense	Pathogenic	M	32	Any other ethnic group
16641015	736	ABCA4	NM_000350.3	c.5882G>A	p.(Gly1961Glu)	Missense	Pathogenic	M	32	Any other ethnic group
17158798	737	ABCA4	NM_000350.3	c.1622T>C	p.(Leu541Pro)	Missense	Pathogenic	F	15	Not stated
17158798	737	ABCA4	NM_000350.3	c.3113C>T	p.(Ala1038Val)	Missense	Pathogenic	F	15	Not stated
17158798	737	ABCA4	NM_000350.3	c.4469G>A	p.(Cys1490Tyr)	Missense	Pathogenic	F	15	Not stated
18163172	737	ABCA4	NM_000350.3	c.1622T>C	p.(Leu541Pro)	Missense	Pathogenic	M	13	White - British
18163172	737	ABCA4	NM_000350.3	c.3113C>T	p.(Ala1038Val)	Missense	Pathogenic	M	13	White - British
18163172	737	ABCA4	NM_000350.3	c.4469G>A	p.(Cys1490Tyr)	Missense	Pathogenic	M	13	White - British
17068855	738	ABCA4	NM_000350.3	c.5461-10T>C	Splice	Splice	Pathogenic	M	64	White - British
17068855	738	ABCA4	NM_000350.3	c.5603A>T	p.(Asn1868Ile)	Missense	Variant of Uncertain Significance	M	64	White - British
17068855	738	ABCA4	NM_000350.3	c.5603A>T	p.(Asn1868Ile)	Missense	Variant of Uncertain Significance	M	64	White - British
10913678	739	ABCA4	NM_000350.3	c.2265C>G	p.(Phe755Leu)	Missense	Variant of Uncertain Significance	M	46	White - Other
10913678	739	ABCA4	NM_000350.3	c.4139C>T	p.(Pro1380Leu)	Missense	Pathogenic	M	46	White - Other
3919957	740	ABCA4	NM_000350.3	c.3323G>A	p.(Arg1108His)	Missense	Pathogenic	F	75	White - British
3919957	740	ABCA4	NM_000350.3	c.4253+5G>T	Splice	Splice	Likely Pathogenic	F	75	White - British
17024888	741	ABCA4	NM_000350.3	c.93G>A	p.(Trp31Ter)	Stopgain	Pathogenic	F	12	Not stated
17024888	741	ABCA4	NM_000350.3	c.93G>A	p.(Trp31Ter)	Stopgain	Pathogenic	F	12	Not stated

17243568	742	ABCA4	NM_000350.3	c.3050+5G>A	Splice	Splice	Likely Pathogenic	M	29	Not stated
17243568	742	ABCA4	NM_000350.3	c.5882G>A	p.(Gly1961Glu)	Missense	Pathogenic	M	29	Not stated
14843548	743	ABCA4	NM_000350.3	c.3064G>A	p.(Glu1022Lys)	Missense	Pathogenic	F	51	Asian - Indian
14843548	743	ABCA4	NM_000350.3	c.3064G>A	p.(Glu1022Lys)	Missense	Pathogenic	F	51	Asian - Indian
17108097	744	ABCA4	NM_000350.3	c.5917del	p.(Val1973Ter)	Stopgain	Pathogenic	F	59	White - Other
17108097	744	ABCA4	NM_000350.3	c.5917del	p.(Val1973Ter)	Stopgain	Pathogenic	F	59	White - Other
17269356	745	ABCA4	NM_000350.3	c.2588G>C	p.(Gly863Ala)	Missense	Pathogenic	F	41	White - British
17269356	745	ABCA4	NM_000350.3	c.331_332del	p.(Glu111ThrfsTer49)	Frameshift	Pathogenic	F	41	White - British
17334694	746	ABCA4	NM_000350.3	c.5461-10T>C	Splice	Splice	Pathogenic	M	51	White - British
17334694	746	ABCA4	NM_000350.3	c.5461-10T>C	Splice	Splice	Pathogenic	M	51	White - British
13439110	747	ABCA4	NM_000350.3	c.4253+4C>T	Splice	Splice	Likely Pathogenic	M	20	White - British
13439110	747	ABCA4	NM_000350.3	c.4849G>A	p.(Val1617Met)	Missense	Variant of Uncertain Significance	M	20	White - British
17344221	748	ABCA4	NM_000350.3	c.2519T>A	p.(Met840Lys)	Missense	Likely Pathogenic	F	61	Not stated
17344221	748	ABCA4	NM_000350.3	c.2588G>C	p.(Gly863Ala)	Missense	Pathogenic	F	61	Not stated
17344221	748	ABCA4	NM_000350.3	c.5603A>T	p.(Asn1868Ile)	Missense	Variant of Uncertain Significance	F	61	Not stated
15881074	749	ABCA4	NM_000350.3	c.5846del	p.(Gly1949AlafsTer25)	Frameshift	Pathogenic	M	12	Any other ethnic group
15881074	749	ABCA4	NM_000350.3	c.5846del	p.(Gly1949AlafsTer25)	Frameshift	Pathogenic	M	12	Any other ethnic group
17402258	750	ABCA4	NM_000350.3	c.5917del	p.(Val1973Ter)	Stopgain	Pathogenic	F	15	Not stated
17402258	750	ABCA4	NM_000350.3	c.5917del	p.(Val1973Ter)	Stopgain	Pathogenic	F	15	Not stated
17357472	751	ABCA4	NM_000350.3	c.5917del	p.(Val1973Ter)	Stopgain	Pathogenic	M	56	Not stated
17357472	751	ABCA4	NM_000350.3	c.5917del	p.(Val1973Ter)	Stopgain	Pathogenic	M	56	Not stated
17085634	752	ABCA4	NM_000350.3	c.1622T>C	p.(Leu541Pro)	Missense	Pathogenic	F	43	White - Other
17085634	752	ABCA4	NM_000350.3	c.3113C>T	p.(Ala1038Val)	Missense	Pathogenic	F	43	White - Other
17085634	752	ABCA4	NM_000350.3	c.4685T>C	p.(Ile1562Thr)	Missense	Pathogenic	F	43	White - Other
15547804	753	ABCA4	NM_000350.3	c.5196+1G>A	Splice	Splice	Pathogenic	M	37	White - British
15547804	753	ABCA4	NM_000350.3	c.5882G>A	p.(Gly1961Glu)	Missense	Pathogenic	M	37	White - British
17536133	753	ABCA4	NM_000350.3	c.5196+1G>A	Splice	Splice	Pathogenic	F	72	Not stated
17536133	753	ABCA4	NM_000350.3	c.6089G>A	p.(Arg2030Gln)	Missense	Pathogenic	F	72	Not stated
16521476	754	ABCA4	NM_000350.3	c.5461-10T>C	Splice	Splice	Pathogenic	M	27	White - Other
16521476	754	ABCA4	NM_000350.3	c.5882G>A	p.(Gly1961Glu)	Missense	Pathogenic	M	27	White - Other
17465125	755	ABCA4	NM_000350.3	c.1253T>C	p.(Phe418Ser)	Missense	Pathogenic	F	64	Not stated
17465125	755	ABCA4	NM_000350.3	c.6079C>T	p.(Leu2027Phe)	Missense	Pathogenic	F	64	Not stated
17430076	756	ABCA4	NM_000350.3	c.4918C>T	p.(Arg1640Trp)	Missense	Pathogenic	M	21	White - British
17430076	756	ABCA4	NM_000350.3	c.6079C>T	p.(Leu2027Phe)	Missense	Pathogenic	M	21	White - British
16542896	757	ABCA4	NM_000350.3	c.5603A>T	p.(Asn1868Ile)	Missense	Variant of Uncertain Significance	F	56	Not stated
16542896	757	ABCA4	NM_000350.3	c.6658C>T	p.(Gln2220Ter)	Stopgain	Pathogenic	F	56	Not stated
13274967	758	ABCA4	NM_000350.3	c.1622T>C	p.(Leu541Pro)	Missense	Pathogenic	M	17	Not stated
13274967	758	ABCA4	NM_000350.3	c.1622T>C	p.(Leu541Pro)	Missense	Pathogenic	M	17	Not stated
13274967	758	ABCA4	NM_000350.3	c.3113C>T	p.(Ala1038Val)	Missense	Pathogenic	M	17	Not stated
13274967	758	ABCA4	NM_000350.3	c.3113C>T	p.(Ala1038Val)	Missense	Pathogenic	M	17	Not stated
8252985	759	ABCA4	NM_000350.3	c.3322C>T	p.(Arg1108Cys)	Missense	Pathogenic	F	67	White - Other
8252985	759	ABCA4	NM_000350.3	c.5882G>A	p.(Gly1961Glu)	Missense	Pathogenic	F	67	White - Other
17589172	760	ABCA4	NM_000350.3	c.1292G>A	p.(Trp431Ter)	Stopgain	Likely Pathogenic	F	17	White - British
17589172	760	ABCA4	NM_000350.3	c.5714+5G>A	Splice	Splice	Pathogenic	F	17	White - British
7014769	761	ABCA4	NM_000350.3	c.2023G>A	p.(Val675Ile)	Missense	Pathogenic	F	36	Not stated
7014769	761	ABCA4	NM_000350.3	c.6658C>T	p.(Gln2220Ter)	Stopgain	Pathogenic	F	36	Not stated
17513628	762	ABCA4	NM_000350.3	c.2588G>C	p.(Gly863Ala)	Missense	Pathogenic	M	29	White - British
17513628	762	ABCA4	NM_000350.3	c.5461-10T>C	Splice	Splice	Pathogenic	M	29	White - British
17624697	763	ABCA4	NM_000350.3	c.3259G>A	p.(Glu1087Lys)	Missense	Pathogenic	M	14	White - Other
17624697	763	ABCA4	NM_000350.3	c.3259G>A	p.(Glu1087Lys)	Missense	Pathogenic	M	14	White - Other
12369412	764	ABCA4	NM_000350.3	c.4734del	p.(Leu1580Ter)	Stopgain	Likely Pathogenic	F	63	Asian - Other

12369412	764	ABCA4	NM_000350.3	c.5882G>A	p.(Gly1961Glu)	Missense	Pathogenic	F	63	Asian - Other
17369267	765	ABCA4	NM_000350.3	c.5882G>A	p.(Gly1961Glu)	Missense	Pathogenic	F	35	Not stated
17369267	765	ABCA4	NM_000350.3	c.6088C>T	p.(Arg2030Ter)	Stopgain	Pathogenic	F	35	Not stated
17290755	766	ABCA4	NM_000350.3	c.4793C>A	p.(Ala1598Asp)	Missense	Pathogenic	M	32	Not stated
17290755	766	ABCA4	NM_000350.3	c.4793C>A	p.(Ala1598Asp)	Missense	Pathogenic	M	32	Not stated
17608338	767	ABCA4	NM_000350.3	c.4539+2028C>T	Splice	Splice	Likely Pathogenic	F	70	Not stated
17608338	767	ABCA4	NM_000350.3	c.5882G>A	p.(Gly1961Glu)	Missense	Pathogenic	F	70	Not stated
17577930	768	ABCA4	NM_000350.3	c.5461-10T>C	Splice	Splice	Pathogenic	M	38	Not stated
17577930	768	ABCA4	NM_000350.3	c.5714+5G>A	Splice	Splice	Pathogenic	M	38	Not stated
17628673	769	ABCA4	NM_000350.3	c.1317G>A	p.(Trp439Ter)	Stopgain	Pathogenic	F	61	Not stated
17628673	769	ABCA4	NM_000350.3	c.4139C>T	p.(Pro1380Leu)	Missense	Pathogenic	F	61	Not stated
15833005	770	ABCA4	NM_000350.3	c.3289A>T	p.(Arg1097Ter)	Stopgain	Pathogenic	F	76	Not stated
15833005	770	ABCA4	NM_000350.3	c.6089G>A	p.(Arg2030Gln)	Missense	Pathogenic	F	76	Not stated
17657093	770	ABCA4	NM_000350.3	c.3289A>T	p.(Arg1097Ter)	Stopgain	Pathogenic	M	79	Not stated
17657093	770	ABCA4	NM_000350.3	c.6089G>A	p.(Arg2030Gln)	Missense	Pathogenic	M	79	Not stated
10268432	771	ABCA4	NM_000350.3	c.2588G>C	p.(Gly863Ala)	Missense	Pathogenic	M	44	Not stated
10268432	771	ABCA4	NM_000350.3	c.5281_5289del	p.(Pro1761_Leu1763del)	Inframe indel	Variant of Uncertain Significance	M	44	Not stated
17347602	772	ABCA4	NM_000350.3	c.5882G>A	p.(Gly1961Glu)	Missense	Pathogenic	F	45	Black - African
17347602	772	ABCA4	NM_000350.3	c.5882G>A	p.(Gly1961Glu)	Missense	Pathogenic	F	45	Black - African
17347602	772	ABCA4	NM_000350.3	c.634C>T	p.(Arg212Cys)	Missense	Pathogenic	F	45	Black - African
16940538	773	ABCA4	NM_000350.3	c.2588G>C	p.(Gly863Ala)	Missense	Pathogenic	F	33	Not stated
16940538	773	ABCA4	NM_000350.3	c.5461-10T>C	Splice	Splice	Pathogenic	F	33	Not stated
10576523	774	ABCA4	NM_000350.3	c.3210_3211dup	p.(Ser1071CysfsTer14)	Frameshift	Pathogenic	M	78	White - British
10576523	774	ABCA4	NM_000350.3	c.5603A>T	p.(Asn1868Ile)	Missense	Variant of Uncertain Significance	M	78	White - British
10576523	774	ABCA4	NM_000350.3	c.5603A>T	p.(Asn1868Ile)	Missense	Variant of Uncertain Significance	M	78	White - British
17619972	775	ABCA4	NM_000350.3	c.2382G>A	p.(Val794Val)	Synonymous	Variant of Uncertain Significance	M	17	Not stated
17619972	775	ABCA4	NM_000350.3	c.768G>T	p.(Val256Val)	Synonymous	Pathogenic	M	17	Not stated
15772336	776	ABCA4	NM_000350.3	c.859-9T>C	Splice	Splice	Likely Pathogenic	M	76	Asian - Indian
15772336	776	ABCA4	NM_000350.3	c.859-9T>C	Splice	Splice	Likely Pathogenic	M	76	Asian - Indian
17408810	777	ABCA4	NM_000350.3	c.1917C>A	p.(Tyr639Ter)	Stopgain	Pathogenic	M	29	Not stated
17408810	777	ABCA4	NM_000350.3	c.6079C>T	p.(Leu2027Phe)	Missense	Pathogenic	M	29	Not stated
6241507	778	ABCA4	NM_000350.3	c.1222C>T	p.(Arg408Ter)	Stopgain	Pathogenic	M	39	Not stated
6241507	778	ABCA4	NM_000350.3	c.3322C>T	p.(Arg1108Cys)	Missense	Pathogenic	M	39	Not stated
17610151	779	ABCA4	NM_000350.3	c.6658C>T	p.(Gln2220Ter)	Stopgain	Pathogenic	F	31	Not stated
17610151	779	ABCA4	NM_000350.3	c.859-9T>C	Splice	Splice	Likely Pathogenic	F	31	Not stated
17868990	780	ABCA4	NM_000350.3	c.1622T>C	p.(Leu541Pro)	Missense	Pathogenic	M	17	Any other ethnic group
17868990	780	ABCA4	NM_000350.3	c.3113C>T	p.(Ala1038Val)	Missense	Pathogenic	M	17	Any other ethnic group
17868990	780	ABCA4	NM_000350.3	c.4537dup	p.(Gln1513ProfsTer42)	Frameshift	Pathogenic	M	17	Any other ethnic group
17799291	781	ABCA4	NM_000350.3	c.3259G>A	p.(Glu1087Lys)	Missense	Pathogenic	M	45	White - Other
17799291	781	ABCA4	NM_000350.3	c.4139C>T	p.(Pro1380Leu)	Missense	Pathogenic	M	45	White - Other
17547032	782	ABCA4	NM_000350.3	c.859-9T>C	Splice	Splice	Likely Pathogenic	F	42	Asian - Pakistani
17547032	782	ABCA4	NM_000350.3	c.859-9T>C	Splice	Splice	Likely Pathogenic	F	42	Asian - Pakistani
17760231	783	ABCA4	NM_000350.3	c.6088C>T	p.(Arg2030Ter)	Stopgain	Pathogenic	F	44	Not stated
17760231	783	ABCA4	NM_000350.3	c.6088C>T	p.(Arg2030Ter)	Stopgain	Pathogenic	F	44	Not stated
10735934	784	ABCA4	NM_000350.3	c.1317G>A	p.(Trp439Ter)	Stopgain	Pathogenic	M	61	Not stated
10735934	784	ABCA4	NM_000350.3	c.191C>T	p.(Ala64Val)	Missense	Likely Pathogenic	M	61	Not stated
690808	785	ABCA4	NM_000350.3	c.2042G>A	p.(Arg681Gln)	Missense	Likely Pathogenic	M	84	White - British
690808	785	ABCA4	NM_000350.3	c.2588G>C	p.(Gly863Ala)	Missense	Pathogenic	M	84	White - British
690808	785	ABCA4	NM_000350.3	c.6209C>G	p.(Thr2070Arg)	Missense	Likely Pathogenic	M	84	White - British
17692898	786	ABCA4	NM_000350.3	c.5882G>A	p.(Gly1961Glu)	Missense	Pathogenic	F	40	Not stated
17692898	786	ABCA4	NM_000350.3	c.5882G>A	p.(Gly1961Glu)	Missense	Pathogenic	F	40	Not stated

17602115	787	ABCA4	NM_000350.3	c.3814-2A>T	Splice	Splice	Pathogenic	M	25	Not stated
17602115	787	ABCA4	NM_000350.3	c.5882G>A	p.(Gly1961Glu)	Missense	Pathogenic	M	25	Not stated
17586946	788	ABCA4	NM_000350.3	c.4539+2028C>T	Splice	Splice	Likely Pathogenic	M	28	Not stated
17586946	788	ABCA4	NM_000350.3	c.5461-10T>C	Splice	Splice	Pathogenic	M	28	Not stated
16484698	789	ABCA4	NM_000350.3	c.1A>G	p.(Met1?)	Start codon	Likely Pathogenic	M	40	White - Other
16484698	789	ABCA4	NM_000350.3	c.2609C>T	p.(Pro870Leu)	Missense	Pathogenic	M	40	White - Other
16484698	789	ABCA4	NM_000350.3	c.6089G>A	p.(Arg2030Gln)	Missense	Pathogenic	M	40	White - Other
17855494	790	ABCA4	NM_000350.3	c.1622T>C	p.(Leu541Pro)	Missense	Pathogenic	M	47	Not stated
17855494	790	ABCA4	NM_000350.3	c.3113C>T	p.(Ala1038Val)	Missense	Pathogenic	M	47	Not stated
17855494	790	ABCA4	NM_000350.3	c.4529C>T	p.(Pro1510Leu)	Missense	Likely Pathogenic	M	47	Not stated
17855494	790	ABCA4	NM_000350.3	c.6040A>G	p.(Met2014Val)	Missense	Variant of Uncertain Significance	M	47	Not stated
13521486	791	ABCA4	NM_000350.3	c.2791G>A	p.(Val931Met)	Missense	Pathogenic	M	70	Black - Other
13521486	791	ABCA4	NM_000350.3	c.3004C>T	p.(Arg1002Trp)	Missense	Pathogenic	M	70	Black - Other
13521486	791	ABCA4	NM_000350.3	c.5056G>A	p.(Val1686Met)	Missense	Likely Pathogenic	M	70	Black - Other
10323928	792	ABCA4	NM_000350.3	c.5882G>A	p.(Gly1961Glu)	Missense	Pathogenic	M	51	Any other ethnic group
10323928	792	ABCA4	NM_000350.3	c.5917del	p.(Val1973Ter)	Stopgain	Pathogenic	M	51	Any other ethnic group
11829292	793	ABCA4	NM_000350.3	c.2588G>C	p.(Gly863Ala)	Missense	Pathogenic	F	58	Not stated
11829292	793	ABCA4	NM_000350.3	c.5461-10T>C	Splice	Splice	Pathogenic	F	58	Not stated
11773047	794	ABCA4	NM_000350.3	c.2588G>C	p.(Gly863Ala)	Missense	Pathogenic	F	67	White - Other
11773047	794	ABCA4	NM_000350.3	c.4773+3A>G	Splice	Splice	Pathogenic	F	67	White - Other
11773047	794	ABCA4	NM_000350.3	c.656G>C	p.(Arg219Thr)	Missense	Likely Pathogenic	F	67	White - Other
14902838	795	ABCA4	NM_000350.3	c.5882G>A	p.(Gly1961Glu)	Missense	Pathogenic	F	49	Unknown
14902838	795	ABCA4	NM_000350.3	c.5882G>A	p.(Gly1961Glu)	Missense	Pathogenic	F	49	Unknown
14902838	795	ABCA4	NM_000350.3	c.634C>T	p.(Arg212Cys)	Missense	Pathogenic	F	49	Unknown
17922295	796	ABCA4	NM_000350.3	c.5714+5G>A	Splice	Splice	Pathogenic	F	19	Not stated
17922295	796	ABCA4	NM_000350.3	c.629del	p.(Leu210ArgfsTer31)	Frameshift	Pathogenic	F	19	Not stated
17985890	797	ABCA4	NM_000350.3	c.2588G>C	p.(Gly863Ala)	Missense	Pathogenic	M	57	Not stated
17985890	797	ABCA4	NM_000350.3	c.5461-10T>C	Splice	Splice	Pathogenic	M	57	Not stated
17985890	797	ABCA4	NM_000350.3	c.5603A>T	p.(Asn1868Ile)	Missense	Variant of Uncertain Significance	M	57	Not stated
17936897	798	ABCA4	NM_000350.3	c.5882G>A	p.(Gly1961Glu)	Missense	Pathogenic	F	28	Any other ethnic group
17936897	798	ABCA4	NM_000350.3	c.5882G>A	p.(Gly1961Glu)	Missense	Pathogenic	F	28	Any other ethnic group
17936897	798	ABCA4	NM_000350.3	c.634C>T	p.(Arg212Cys)	Missense	Pathogenic	F	28	Any other ethnic group
13945154	799	ABCA4	NM_000350.3	c.1957C>T	p.(Arg653Cys)	Missense	Pathogenic	F	53	White - Other
13945154	799	ABCA4	NM_000350.3	c.71G>A	p.(Arg24His)	Missense	Pathogenic	F	53	White - Other
17813851	800	ABCA4	NM_000350.3	c.2588G>C	p.(Gly863Ala)	Missense	Pathogenic	M	91	Not stated
17813851	800	ABCA4	NM_000350.3	c.5461-10T>C	Splice	Splice	Pathogenic	M	91	Not stated
17813851	800	ABCA4	NM_000350.3	c.5908C>T	p.(Leu1970Phe)	Missense	Variant of Uncertain Significance	M	91	Not stated
18003061	801	ABCA4	NM_000350.3	c.1906C>T	p.(Gln636Ter)	Stopgain	Pathogenic	F	26	Not stated
18003061	801	ABCA4	NM_000350.3	c.6242C>T	p.(Thr2081Ile)	Missense	Likely Pathogenic	F	26	Not stated
17905411	802	ABCA4	NM_000350.3	c.3064G>A	p.(Glu1022Lys)	Missense	Pathogenic	M	39	Any other ethnic group
17905411	802	ABCA4	NM_000350.3	c.5882G>A	p.(Gly1961Glu)	Missense	Pathogenic	M	39	Any other ethnic group
17890200	803	ABCA4	NM_000350.3	c.6658C>T	p.(Gln2220Ter)	Stopgain	Pathogenic	M	38	Not stated
17890200	803	ABCA4	NM_000350.3	c.6658C>T	p.(Gln2220Ter)	Stopgain	Pathogenic	M	38	Not stated
2049116	803	ABCA4	NM_000350.3	c.6658C>T	p.(Gln2220Ter)	Stopgain	Pathogenic	M	43	Unknown
2049116	803	ABCA4	NM_000350.3	c.6658C>T	p.(Gln2220Ter)	Stopgain	Pathogenic	M	43	Unknown
17841746	804	ABCA4	NM_000350.3	c.2861A>C	p.(Tyr954Ser)	Missense	Pathogenic	F	30	Not stated
17841746	804	ABCA4	NM_000350.3	c.5603A>T	p.(Asn1868Ile)	Missense	Variant of Uncertain Significance	F	30	Not stated
18093333	805	ABCA4	NM_000350.3	c.1995C>A	p.(Tyr665Ter)	Stopgain	Pathogenic	M	46	Not stated
18093333	805	ABCA4	NM_000350.3	c.5882G>A	p.(Gly1961Glu)	Missense	Pathogenic	M	46	Not stated
3532423	806	ABCA4	NM_000350.3	c.4253+43G>A	Splice	Splice	Likely Pathogenic	F	57	White - British
3532423	806	ABCA4	NM_000350.3	c.4537del	p.(Gln1513ArgfsTer13)	Frameshift	Pathogenic	F	57	White - British

17941209	807	ABCA4	NM_000350.3	c.1222C>T	p.(Arg408Ter)	Stopgain	Pathogenic	F	62	Not stated
17941209	807	ABCA4	NM_000350.3	c.4685T>C	p.(Ile1562Thr)	Missense	Pathogenic	F	62	Not stated
18496274	808	ABCA4	NM_000350.3	c.2588G>C	p.(Gly863Ala)	Missense	Pathogenic	M	14	White - British
18496274	808	ABCA4	NM_000350.3	c.5461-10T>C	Splice	Splice	Pathogenic	M	14	White - British
18496274	808	ABCA4	NM_000350.3	c.5603A>T	p.(Asn1868Ile)	Missense	Variant of Uncertain Significance	M	14	White - British
18496274	808	ABCA4	NM_000350.3	c.5603A>T	p.(Asn1868Ile)	Missense	Variant of Uncertain Significance	M	14	White - British
17832506	809	ABCA4	NM_000350.3	c.1622T>C	p.(Leu541Pro)	Missense	Pathogenic	F	28	Not stated
17832506	809	ABCA4	NM_000350.3	c.3113C>T	p.(Ala1038Val)	Missense	Pathogenic	F	28	Not stated
17832506	809	ABCA4	NM_000350.3	c.5882G>A	p.(Gly1961Glu)	Missense	Pathogenic	F	28	Not stated
14368710	810	ABCA4	NM_000350.3	c.2813T>C	p.(Phe938Ser)	Missense	Pathogenic	F	35	Black - Other
14368710	810	ABCA4	NM_000350.3	c.834del	p.(Asp279IlefsTer21)	Frameshift	Pathogenic	F	35	Black - Other
17985862	811	ABCA4	NM_000350.3	c.498del	p.(Leu167SerfsTer15)	Frameshift	Likely Pathogenic	M	66	Not stated
17985862	811	ABCA4	NM_000350.3	c.5603A>T	p.(Asn1868Ile)	Missense	Variant of Uncertain Significance	M	66	Not stated
13203336	812	ABCA4	NM_000350.3	c.1622T>C	p.(Leu541Pro)	Missense	Pathogenic	M	66	Any other ethnic group
13203336	812	ABCA4	NM_000350.3	c.3113C>T	p.(Ala1038Val)	Missense	Pathogenic	M	66	Any other ethnic group
13203336	812	ABCA4	NM_000350.3	c.5882G>A	p.(Gly1961Glu)	Missense	Pathogenic	M	66	Any other ethnic group
9965143	813	ABCA4	NM_000350.3	c.3350C>A	p.(Thr1117Asn)	Missense	Likely Pathogenic	M	44	Not stated
9965143	813	ABCA4	NM_000350.3	c.5882G>A	p.(Gly1961Glu)	Missense	Pathogenic	M	44	Not stated
18144608	814	ABCA4	NM_000350.3	c.1519G>T	p.(Asp507Tyr)	Missense	Likely Pathogenic	F	33	Not stated
18144608	814	ABCA4	NM_000350.3	c.6079C>T	p.(Leu2027Phe)	Missense	Pathogenic	F	33	Not stated
17397554	815	ABCA4	NM_000350.3	c.1805G>A	p.(Arg602Gln)	Missense	Pathogenic	F	54	Not stated
17397554	815	ABCA4	NM_000350.3	c.5761G>A	p.(Val1921Met)	Missense	Likely Pathogenic	F	54	Not stated
9617362	816	ABCA4	NM_000350.3	c.194G>A	p.(Gly65Glu)	Missense	Pathogenic	F	41	White - Other
9617362	816	ABCA4	NM_000350.3	c.5882G>A	p.(Gly1961Glu)	Missense	Pathogenic	F	41	White - Other
18150747	817	ABCA4	NM_000350.3	c.3259G>A	p.(Glu1087Lys)	Missense	Pathogenic	M	63	Not stated
18150747	817	ABCA4	NM_000350.3	c.5882G>A	p.(Gly1961Glu)	Missense	Pathogenic	M	63	Not stated
18152574	818	ABCA4	NM_000350.3	c.6290C>T	p.(Pro2097Leu)	Missense	Pathogenic	M	68	Any other ethnic group
18152574	818	ABCA4	NM_000350.3	c.6320G>A	p.(Arg2107His)	Missense	Pathogenic	M	68	Any other ethnic group
3249364	819	ABCA4	NM_000350.3	c.5714+5G>A	Splice	Splice	Pathogenic	F	68	Not stated
3249364	819	ABCA4	NM_000350.3	c.6079C>T	p.(Leu2027Phe)	Missense	Pathogenic	F	68	Not stated
17084199	820	ABCA4	NM_000350.3	c.42_48delinsAG	p.(Asn14LysfsTer38)	Frameshift	Pathogenic	M	62	Black - Caribbean
17084199	820	ABCA4	NM_000350.3	c.6320G>A	p.(Arg2107His)	Missense	Pathogenic	M	62	Black - Caribbean
16691527	821	ABCA4	NM_000350.3	c.1846G>T	p.(Glu616Ter)	Stopgain	Likely Pathogenic	F	57	Not stated
16691527	821	ABCA4	NM_000350.3	c.5882G>A	p.(Gly1961Glu)	Missense	Pathogenic	F	57	Not stated
16661861	822	ABCA4	NM_000350.3	c.4462T>C	p.(Cys1488Arg)	Missense	Pathogenic	M	46	Not stated
16661861	822	ABCA4	NM_000350.3	c.4462T>C	p.(Cys1488Arg)	Missense	Pathogenic	M	46	Not stated
10478712	823	ABCA4	NM_000350.3	c.4139C>T	p.(Pro1380Leu)	Missense	Pathogenic	F	44	White - British
10478712	823	ABCA4	NM_000350.3	c.6089G>A	p.(Arg2030Gln)	Missense	Pathogenic	F	44	White - British
8547587	824	ABCA4	NM_000350.3	c.2092T>C	p.(Cys698Arg)	Missense	Variant of Uncertain Significance	M	26	Any other ethnic group
8547587	824	ABCA4	NM_000350.3	c.2092T>C	p.(Cys698Arg)	Missense	Variant of Uncertain Significance	M	26	Any other ethnic group
18263510	825	ABCA4	NM_000350.3	c.2041C>T	p.(Arg681Ter)	Stopgain	Pathogenic	F	55	Not stated
18263510	825	ABCA4	NM_000350.3	c.2971G>C	p.(Gly991Arg)	Missense	Pathogenic	F	55	Not stated
18227600	826	ABCA4	NM_000350.3	c.5087G>T	p.(Ser1696Ile)	Missense	Likely Pathogenic	F	18	Not stated
18227600	826	ABCA4	NM_000350.3	c.5882G>A	p.(Gly1961Glu)	Missense	Pathogenic	F	18	Not stated
18217002	827	ABCA4	NM_000350.3	c.4139C>T	p.(Pro1380Leu)	Missense	Pathogenic	M	53	Not stated
18217002	827	ABCA4	NM_000350.3	c.5882G>A	p.(Gly1961Glu)	Missense	Pathogenic	M	53	Not stated
13458220	828	ABCA4	NM_000350.3	c.3210_3211dup	p.(Ser1071CysfsTer14)	Frameshift	Pathogenic	F	60	Not stated
13458220	828	ABCA4	NM_000350.3	c.5603A>T	p.(Asn1868Ile)	Missense	Variant of Uncertain Significance	F	60	Not stated
17973157	829	ABCA4	NM_000350.3	c.5882G>A	p.(Gly1961Glu)	Missense	Pathogenic	F	46	Any other ethnic group
17973157	829	ABCA4	NM_000350.3	c.634C>T	p.(Arg212Cys)	Missense	Pathogenic	F	46	Any other ethnic group
18290971	830	ABCA4	NM_000350.3	c.1906C>T	p.(Gln636Ter)	Stopgain	Pathogenic	M	13	Not stated

18290971	830	ABCA4	NM_000350.3	c.3210_3211dup	p.(Ser1071CysfsTer14)	Frameshift	Pathogenic	M	13	Not stated
18359977	831	ABCA4	NM_000350.3	c.2971G>C	p.(Gly991Arg)	Missense	Pathogenic	M	15	Black - African
18359977	831	ABCA4	NM_000350.3	c.5077G>A	p.(Val1693Ile)	Missense	Likely Pathogenic	M	15	Black - African
18378856	832	ABCA4	NM_000350.3	c.4918C>T	p.(Arg1640Trp)	Missense	Pathogenic	F	46	Not stated
18378856	832	ABCA4	NM_000350.3	c.5603A>T	p.(Asn1868Ile)	Missense	Variant of Uncertain Significance	F	46	Not stated
10020030	833	ABCA4	NM_000350.3	c.6089G>A	p.(Arg2030Gln)	Missense	Pathogenic	F	33	Not stated
10020030	833	ABCA4	NM_000350.3	c.690C>G	p.(Cys230Trp)	Missense	Likely Pathogenic	F	33	Not stated
18306378	834	ABCA4	NM_000350.3	c.6729+5_6729+19del	Splice	Splice	Likely Pathogenic	M	11	Not stated
18306378	834	ABCA4	NM_000350.3	c.6729+5_6729+19del	Splice	Splice	Likely Pathogenic	M	11	Not stated
18409474	835	ABCA4	NM_000350.3	c.1522C>T	p.(Arg508Cys)	Missense	Pathogenic	M	57	Not stated
18409474	835	ABCA4	NM_000350.3	c.1964T>G	p.(Phe655Cys)	Missense	Pathogenic	M	57	Not stated
18409474	835	ABCA4	NM_000350.3	c.2023G>A	p.(Val675Ile)	Missense	Pathogenic	M	57	Not stated
18118330	836	ABCA4	NM_000350.3	c.3259G>A	p.(Glu1087Lys)	Missense	Pathogenic	F	32	Not stated
18118330	836	ABCA4	NM_000350.3	c.5882G>A	p.(Gly1961Glu)	Missense	Pathogenic	F	32	Not stated
18498248	837	ABCA4	NM_000350.3	c.1622T>C	p.(Leu541Pro)	Missense	Pathogenic	M	30	Not stated
18498248	837	ABCA4	NM_000350.3	c.3113C>T	p.(Ala1038Val)	Missense	Pathogenic	M	30	Not stated
18498248	837	ABCA4	NM_000350.3	c.5882G>A	p.(Gly1961Glu)	Missense	Pathogenic	M	30	Not stated
18616443	838	ABCA4	NM_000350.3	c.3064G>A	p.(Glu1022Lys)	Missense	Pathogenic	M	48	Not stated
18616443	838	ABCA4	NM_000350.3	c.5882G>A	p.(Gly1961Glu)	Missense	Pathogenic	M	48	Not stated
18547906	839	ABCA4	NM_000350.3	c.4234C>T	p.(Gln1412Ter)	Stopgain	Pathogenic	M	46	Not stated
18547906	839	ABCA4	NM_000350.3	c.5603A>T	p.(Asn1868Ile)	Missense	Variant of Uncertain Significance	M	46	Not stated
9997833	840	ABCA4	NM_000350.3	c.93G>A	p.(Trp31Ter)	Stopgain	Pathogenic	M	21	Not stated
9997833	840	ABCA4	NM_000350.3	c.93G>A	p.(Trp31Ter)	Stopgain	Pathogenic	M	21	Not stated
18547598	841	ABCA4	NM_000350.3	c.3064G>A	p.(Glu1022Lys)	Missense	Pathogenic	F	16	Unknown
18547598	841	ABCA4	NM_000350.3	c.5882G>A	p.(Gly1961Glu)	Missense	Pathogenic	F	16	Unknown
18684098	842	ABCA4	NM_000350.3	c.5882G>A	p.(Gly1961Glu)	Missense	Pathogenic	M	14	Not stated
18684098	842	ABCA4	NM_000350.3	c.6658C>T	p.(Gln2220Ter)	Stopgain	Pathogenic	M	14	Not stated
18471130	843	ABCA4	NM_000350.3	c.5461-10T>C	Splice	Splice	Pathogenic	F	52	Not stated
18471130	843	ABCA4	NM_000350.3	c.5603A>T	p.(Asn1868Ile)	Missense	Variant of Uncertain Significance	F	52	Not stated
18471130	843	ABCA4	NM_000350.3	c.5603A>T	p.(Asn1868Ile)	Missense	Variant of Uncertain Significance	F	52	Not stated
18604837	844	ABCA4	NM_000350.3	c.1622T>C	p.(Leu541Pro)	Missense	Pathogenic	F	36	White - British
18604837	844	ABCA4	NM_000350.3	c.3113C>T	p.(Ala1038Val)	Missense	Pathogenic	F	36	White - British
18604837	844	ABCA4	NM_000350.3	c.5714+5G>A	Splice	Splice	Pathogenic	F	36	White - British
6838348	845	ABCA4	NM_000350.3	c.4462T>C	p.(Cys1488Arg)	Missense	Pathogenic	M	54	Asian - Indian
6838348	845	ABCA4	NM_000350.3	c.5882G>A	p.(Gly1961Glu)	Missense	Pathogenic	M	54	Asian - Indian
18632620	846	ABCA4	NM_000350.3	c.1622T>C	p.(Leu541Pro)	Missense	Pathogenic	F	31	Any other ethnic group
18632620	846	ABCA4	NM_000350.3	c.3113C>T	p.(Ala1038Val)	Missense	Pathogenic	F	31	Any other ethnic group
18632620	846	ABCA4	NM_000350.3	c.5882G>A	p.(Gly1961Glu)	Missense	Pathogenic	F	31	Any other ethnic group
9861221	847	ABCA4	NM_000350.3	c.3259G>A	p.(Glu1087Lys)	Missense	Pathogenic	F	24	Asian - Other
9861221	847	ABCA4	NM_000350.3	c.3322C>T	p.(Arg1108Cys)	Missense	Pathogenic	F	24	Asian - Other
18590249	848	ABCA4	NM_000350.3	c.859-9T>C	Splice	Splice	Likely Pathogenic	F	64	Not stated
18590249	848	ABCA4	NM_000350.3	c.859-9T>C	Splice	Splice	Likely Pathogenic	F	64	Not stated
18709676	849	ABCA4	NM_000350.3	c.4139C>T	p.(Pro1380Leu)	Missense	Pathogenic	F	20	Not stated
18709676	849	ABCA4	NM_000350.3	c.4469G>A	p.(Cys1490Tyr)	Missense	Pathogenic	F	20	Not stated
18739237	850	ABCA4	NM_000350.3	c.6729+5_6729+19del	Splice	Splice	Likely Pathogenic	M	41	Not stated
18739237	850	ABCA4	NM_000350.3	c.6729+5_6729+19del	Splice	Splice	Likely Pathogenic	M	41	Not stated
18511548	851	ABCA4	NM_000350.3	c.5882G>A	p.(Gly1961Glu)	Missense	Pathogenic	F	26	Any other ethnic group
18511548	851	ABCA4	NM_000350.3	c.5882G>A	p.(Gly1961Glu)	Missense	Pathogenic	F	26	Any other ethnic group
18650939	852	ABCA4	NM_000350.3	c.1906C>T	p.(Gln636Ter)	Stopgain	Pathogenic	M	13	Not stated
18650939	852	ABCA4	NM_000350.3	c.5018+5G>A	Splice	Splice	Pathogenic	M	13	Not stated
18626607	853	ABCA4	NM_000350.3	c.4469G>A	p.(Cys1490Tyr)	Missense	Pathogenic	M	54	Not stated

18626607	853	ABCA4	NM_000350.3	c.5603A>T	p.(Asn1868Ile)	Missense	Variant of Uncertain Significance	M	54	Not stated
18626607	853	ABCA4	NM_000350.3	c.6089G>A	p.(Arg2030Gln)	Missense	Pathogenic	M	54	Not stated
8281881	854	ABCA4	NM_000350.3	c.1069_1075del	p.(Asp357SerfsTer15)	Frameshift	Pathogenic	M	48	Asian - Bangladeshi
8281881	854	ABCA4	NM_000350.3	c.5882G>A	p.(Gly1961Glu)	Missense	Pathogenic	M	48	Asian - Bangladeshi
18481602	855	ABCA4	NM_000350.3	c.5882G>A	p.(Gly1961Glu)	Missense	Pathogenic	F	33	Not stated
18481602	855	ABCA4	NM_000350.3	c.634C>T	p.(Arg212Cys)	Missense	Pathogenic	F	33	Not stated
18481602	855	ABCA4	NM_000350.3	c.634C>T	p.(Arg212Cys)	Missense	Pathogenic	F	33	Not stated
18455450	856	ABCA4	NM_000350.3	c.1937+1G>A	Splice	Splice	Pathogenic	M	11	Black - African
18455450	856	ABCA4	NM_000350.3	c.1937+1G>A	Splice	Splice	Pathogenic	M	11	Black - African
18653648	857	ABCA4	NM_000350.3	c.1609C>T	p.(Arg537Cys)	Missense	Pathogenic	M	14	Not stated
18653648	857	ABCA4	NM_000350.3	c.1609C>T	p.(Arg537Cys)	Missense	Pathogenic	M	14	Not stated
18653648	857	ABCA4	NM_000350.3	c.5881G>A	p.(Gly1961Arg)	Missense	Pathogenic	M	14	Not stated
18653648	857	ABCA4	NM_000350.3	c.5881G>A	p.(Gly1961Arg)	Missense	Pathogenic	M	14	Not stated
17896108	858	ABCA4	NM_000350.3	c.5461-10T>C	Splice	Splice	Pathogenic	F	16	Not stated
17896108	858	ABCA4	NM_000350.3	c.5714+5G>A	Splice	Splice	Pathogenic	F	16	Not stated
5185935	115	ABCC6	NM_001171.6	c.708_709dup	p.(Trp237SerfsTer22)	Frameshift	Pathogenic	M	43	Black - Other
5185935	115	ABCC6	NM_001171.6	c.708_709dup	p.(Trp237SerfsTer22)	Frameshift	Pathogenic	M	43	Black - Other
10355736	859	ABCC6	NM_001171.6	c.3421C>T	p.(Arg1141Ter)	Stopgain	Pathogenic	F	74	Not stated
10355736	859	ABCC6	NM_001171.6	c.3421C>T	p.(Arg1141Ter)	Stopgain	Pathogenic	F	74	Not stated
794247	860	ABCC6	NM_001171.6	c.3413G>A	p.(Arg1138Gln)	Missense	Pathogenic	M	72	Not stated
794247	860	ABCC6	NM_001171.6	Exon 23-28 deletion	Deletion	Deletion	Likely Pathogenic	M	72	Not stated
11514299	861	ABCC6	NM_001171.6	Exon 23-28 deletion	Deletion	Deletion	Likely Pathogenic	F	71	Unknown
11514299	861	ABCC6	NM_001171.6	c.4222C>T	p.(Gln1408Ter)	Stopgain	Likely Pathogenic	F	71	Unknown
659609	862	ABCC6	NM_001171.6	c.3907G>C	p.(Ala1303Pro)	Missense	Pathogenic	M	86	Not stated
659609	862	ABCC6	NM_001171.6	c.3907G>C	p.(Ala1303Pro)	Missense	Pathogenic	M	86	Not stated
8581467	863	ABCC6	NM_001171.6	c.4016G>A	p.(Arg1339His)	Missense	Pathogenic	M	27	Not stated
8581467	863	ABCC6	NM_001171.6	c.4016G>A	p.(Arg1339His)	Missense	Pathogenic	M	27	Not stated
6736904	864	ABCC6	NM_001171.6	Exon 23-28 deletion	Deletion	Deletion	Likely Pathogenic	F	77	White - British
6736904	864	ABCC6	NM_001171.6	Exon 23-28 deletion	Deletion	Deletion	Likely Pathogenic	F	77	White - British
6667954	865	ABCC6	NM_001171.6	c.3389C>T	p.(Thr1130Met)	Missense	Pathogenic	M	55	White - British
6667954	865	ABCC6	NM_001171.6	c.4104del	p.(Asp1368GlufsTer35)	Frameshift	Pathogenic	M	55	White - British
896594	866	ABCC6	NM_001171.6	c.1944_1965del	p.(Ile649AlafsTer32)	Frameshift	Pathogenic	F	63	White - British
896594	866	ABCC6	NM_001171.6	c.2278C>T	p.(Arg760Trp)	Missense	Pathogenic	F	63	White - British
11620916	867	ABCC6	NM_001171.6	c.3421C>T	p.(Arg1141Ter)	Stopgain	Pathogenic	F	60	White - British
11620916	867	ABCC6	NM_001171.6	Exon 23-28 deletion	Deletion	Deletion	Likely Pathogenic	F	60	White - British
401603	868	ABCC6	NM_001171.6	c.3506+5del	Splice	Splice	Variant of Uncertain Significance	F	81	White - Other
401603	868	ABCC6	NM_001171.6	c.3506+5del	Splice	Splice	Variant of Uncertain Significance	F	81	White - Other
12351975	869	ABCC6	NM_001171.6	c.3421C>T	p.(Arg1141Ter)	Stopgain	Pathogenic	M	69	Any other ethnic group
12351975	869	ABCC6	NM_001171.6	c.3421C>T	p.(Arg1141Ter)	Stopgain	Pathogenic	M	69	Any other ethnic group
15984282	870	ABCC6	NM_001171.6	c.1091C>G	p.(Thr364Arg)	Missense	Likely Pathogenic	F	56	Not stated
15984282	870	ABCC6	NM_001171.6	c.1519G>C	p.(Glu507Gln)	Missense	Variant of Uncertain Significance	F	56	Not stated
17026596	871	ABCC6	NM_001171.6	c.1081T>C	p.(Cys361Arg)	Missense	Variant of Uncertain Significance	M	59	White - British
17026596	871	ABCC6	NM_001171.6	c.3775del	p.(Trp1259GlyfsTer14)	Frameshift	Pathogenic	M	59	White - British
17916233	872	ABCC6	NM_001171.6	Exon 23-29 deletion	Deletion	Deletion	Likely Pathogenic	M	58	Not stated
17916233	872	ABCC6	NM_001171.6	Exon 23-29 deletion	Deletion	Deletion	Likely Pathogenic	M	58	Not stated
11061987	873	ABHD12	NM_001042472.3	c.193C>T	p.(Arg65Ter)	Stopgain	Pathogenic	M	31	Asian - Pakistani
11061987	873	ABHD12	NM_001042472.3	c.193C>T	p.(Arg65Ter)	Stopgain	Pathogenic	M	31	Asian - Pakistani
12648047	874	ABHD12	NM_001042472.3	c.620-2A>G	Splice	Splice	Pathogenic	M	38	Any other ethnic group
12648047	874	ABHD12	NM_001042472.3	c.620-2A>G	Splice	Splice	Pathogenic	M	38	Any other ethnic group
7069838	875	ABHD12	NM_001042472.3	c.1154T>C	p.(Leu385Pro)	Missense	Variant of Uncertain Significance	M	57	Asian - Other
7069838	875	ABHD12	NM_001042472.3	c.374C>T	p.(Thr125Met)	Missense	Variant of Uncertain Significance	M	57	Asian - Other

1535302	876	ABHD12	NM_001042472.3	c.784C>T	p.(Arg262Ter)	Stopgain	Pathogenic	M	59	Not stated
1535302	876	ABHD12	NM_001042472.3	c.867+5G>A	Splice	Splice	Variant of Uncertain Significance	M	59	Not stated
12282535	877	ADAMTS18	NM_199355.4	c.1952G>A	p.(Arg651Gln)	Missense	Variant of Uncertain Significance	M	26	Asian - Indian
12282535	877	ADAMTS18	NM_199355.4	c.1952G>A	p.(Arg651Gln)	Missense	Variant of Uncertain Significance	M	26	Asian - Indian
1381253	878	ADGRV1	NM_032119.4	c.4123G>C	p.(Asp1375His)	Missense	Variant of Uncertain Significance	M	64	White - British
1381253	878	ADGRV1	NM_032119.4	c.6458_6466delins8	p.(Lys2153ArgfsTer7)	Frameshift	Likely Pathogenic	M	64	White - British
3418652	879	ADGRV1	NM_032119.4	c.15144del	p.(Ser5048ArgfsTer29)	Frameshift	Likely Pathogenic	F	60	White - British
3418652	879	ADGRV1	NM_032119.4	c.6962_6963del	p.(Val2321AlafsTer4)	Frameshift	Pathogenic	F	60	White - British
6995050	880	ADGRV1	NM_032119.4	c.16111del	p.(Ser5371ValfsTer8)	Frameshift	Likely Pathogenic	F	56	Any other ethnic group
6995050	880	ADGRV1	NM_032119.4	Exon 3-43 deletion	Deletion	Deletion	Likely Pathogenic	F	56	Any other ethnic group
3691442	881	ADGRV1	NM_032119.4	c.6307G>T	p.(Glu2103Ter)	Stopgain	Pathogenic	F	77	Unknown
3691442	881	ADGRV1	NM_032119.4	c.6901C>T	p.(Gln2301Ter)	Stopgain	Pathogenic	F	77	Unknown
4406114	882	ADGRV1	NM_032119.4	c.10016G>A	p.(Ser3339Asn)	Missense	Variant of Uncertain Significance	M	70	Not stated
4406114	882	ADGRV1	NM_032119.4	c.6856C>T	p.(Arg2286Ter)	Stopgain	Pathogenic	M	70	Not stated
7611358	883	ADGRV1	NM_032119.4	Exon 83 deletion	Deletion	Deletion	Likely Pathogenic	M	50	Not stated
7611358	883	ADGRV1	NM_032119.4	Exon 83 deletion	Deletion	Deletion	Likely Pathogenic	M	50	Not stated
11077093	884	ADGRV1	NM_032119.4	c.14517G>C	p.(Gln4839His)	Missense	Likely Pathogenic	M	44	Not stated
11077093	884	ADGRV1	NM_032119.4	c.17314C>T	p.(Arg5772Ter)	Stopgain	Pathogenic	M	44	Not stated
9044363	885	ADGRV1	NM_032119.4	c.10088_10091del	p.(Val3363AspfsTer11)	Frameshift	Pathogenic	F	54	White - British
9044363	885	ADGRV1	NM_032119.4	c.10736_10737del	p.(Ala3579ValfsTer7)	Frameshift	Pathogenic	F	54	White - British
12614748	886	ADGRV1	NM_032119.4	c.12798T>A	p.(Tyr4266Ter)	Stopgain	Pathogenic	M	55	Asian - Indian
12614748	886	ADGRV1	NM_032119.4	c.12798T>A	p.(Tyr4266Ter)	Stopgain	Pathogenic	M	55	Asian - Indian
12614748	886	ADGRV1	NM_032119.4	c.7087G>A	p.(Glu2363Lys)	Missense	Variant of Uncertain Significance	M	55	Asian - Indian
13579733	887	ADGRV1	NM_032119.4	c.2758C>T	p.(Arg920Ter)	Stopgain	Pathogenic	M	49	Asian - Pakistani
13579733	887	ADGRV1	NM_032119.4	c.9749-2del	Splice	Splice	Pathogenic	M	49	Asian - Pakistani
14862623	888	ADGRV1	NM_032119.4	c.2070G>A	p.(Trp690Ter)	Stopgain	Likely Pathogenic	M	41	White - Other
14862623	888	ADGRV1	NM_032119.4	c.2070G>A	p.(Trp690Ter)	Stopgain	Likely Pathogenic	M	41	White - Other
17730656	889	ADGRV1	NM_032119.4	c.6466del	p.(Ala2156LeufsTer4)	Frameshift	Likely Pathogenic	M	41	Not stated
17730656	889	ADGRV1	NM_032119.4	Exon 21 deletion	Deletion	Deletion	Likely Pathogenic	M	41	Not stated
17922316	890	ADGRV1	NM_032119.4	c.16453_16454del	p.(Gln5485AspfsTer2)	Frameshift	Likely Pathogenic	M	31	Not stated
17922316	890	ADGRV1	NM_032119.4	c.2239A>G	p.(Arg747Gly)	Missense	Variant of Uncertain Significance	M	31	Not stated
14317344	891	ADGRV1	NM_032119.4	c.4553_4554del	p.(Gln1518ArgfsTer4)	Frameshift	Likely Pathogenic	F	27	Asian - Indian
14317344	891	ADGRV1	NM_032119.4	c.4553_4554del	p.(Gln1518ArgfsTer4)	Frameshift	Likely Pathogenic	F	27	Asian - Indian
3216121	892	AGBL5	NM_021831.6	c.1504dup	p.(Ala502GlyfsTer15)	Frameshift	Pathogenic	F	73	White - British
3216121	892	AGBL5	NM_021831.6	c.752T>G	p.(Val251Gly)	Missense	Likely Pathogenic	F	73	White - British
3967942	893	AHI1	NM_017651.5	c.2212C>T	p.(Arg738Ter)	Stopgain	Pathogenic	F	65	White - British
3967942	893	AHI1	NM_017651.5	c.703dup	p.(Arg235LysfsTer12)	Frameshift	Pathogenic	F	65	White - British
10136853	894	AHI1	NM_017651.5	c.2569_2570insAG	p.(Phe857Ter)	Stopgain	Pathogenic	M	55	Not stated
10136853	894	AHI1	NM_017651.5	c.2988del	p.(Val997SerfsTer20)	Frameshift	Variant of Uncertain Significance	M	55	Not stated
10185958	895	AHI1	NM_017651.5	c.2087A>G	p.(His696Arg)	Missense	Pathogenic	F	49	Asian - Indian
10185958	895	AHI1	NM_017651.5	c.2429C>T	p.(Pro810Leu)	Missense	Variant of Uncertain Significance	F	49	Asian - Indian
16517619	896	AHI1	NM_017651.5	c.1793C>T	p.(Pro598Leu)	Missense	Variant of Uncertain Significance	F	17	Not stated
16517619	896	AHI1	NM_017651.5	c.2072del	p.(Phe691SerfsTer19)	Frameshift	Likely Pathogenic	F	17	Not stated
17251156	897	AHI1	NM_017651.5	c.2244C>G	p.(Asn748Lys)	Missense	Variant of Uncertain Significance	M	43	White - Other
17251156	897	AHI1	NM_017651.5	c.2244C>G	p.(Asn748Lys)	Missense	Variant of Uncertain Significance	M	43	White - Other
3559926	898	AIPL1	NM_014336.5	c.465G>T	p.(Gln155His)	Missense	Variant of Uncertain Significance	M	34	Not stated
3559926	898	AIPL1	NM_014336.5	c.465G>T	p.(Gln155His)	Missense	Variant of Uncertain Significance	M	34	Not stated
7195761	899	AIPL1	NM_014336.5	c.834G>A	p.(Trp278Ter)	Stopgain	Pathogenic	M	40	White - Other
7195761	899	AIPL1	NM_014336.5	c.834G>A	p.(Trp278Ter)	Stopgain	Pathogenic	M	40	White - Other
9942841	900	AIPL1	NM_014336.5	c.277-2A>G	Splice	Splice	Pathogenic	M	33	Not stated
9942841	900	AIPL1	NM_014336.5	c.784G>A	p.(Gly262Ser)	Missense	Likely Pathogenic	M	33	Not stated

11191991	901	AIPL1	NM_014336.5	c.190G>A	p.(Gly64Arg)	Missense	Pathogenic	F	14	White - British
11191991	901	AIPL1	NM_014336.5	c.834G>A	p.(Trp278Ter)	Stopgain	Pathogenic	F	14	White - British
12507977	902	AIPL1	NM_014336.5	c.834G>A	p.(Trp278Ter)	Stopgain	Pathogenic	M	12	Not stated
12507977	902	AIPL1	NM_014336.5	c.834G>A	p.(Trp278Ter)	Stopgain	Pathogenic	M	12	Not stated
13287931	903	AIPL1	NM_014336.5	c.834G>A	p.(Trp278Ter)	Stopgain	Pathogenic	M	10	Not stated
13287931	903	AIPL1	NM_014336.5	c.929dup	p.(Asn310LysfsTer98)	Frameshift	Likely Pathogenic	M	10	Not stated
13861294	904	AIPL1	NM_014336.5	c.783del	p.(Gly262AlafsTer3)	Frameshift	Likely Pathogenic	F	36	Any other ethnic group
13861294	904	AIPL1	NM_014336.5	c.834G>A	p.(Trp278Ter)	Stopgain	Pathogenic	F	36	Any other ethnic group
15502514	905	AIPL1	NM_014336.5	c.834G>A	p.(Trp278Ter)	Stopgain	Pathogenic	M	43	Asian - Pakistani
15502514	905	AIPL1	NM_014336.5	c.834G>A	p.(Trp278Ter)	Stopgain	Pathogenic	M	43	Asian - Pakistani
16619063	906	AIPL1	NM_014336.5	c.834G>A	p.(Trp278Ter)	Stopgain	Pathogenic	M	9	Not stated
16619063	906	AIPL1	NM_014336.5	c.834G>A	p.(Trp278Ter)	Stopgain	Pathogenic	M	9	Not stated
17730754	907	AIPL1	NM_014336.5	c.834G>A	p.(Trp278Ter)	Stopgain	Pathogenic	M	9	Not stated
17730754	907	AIPL1	NM_014336.5	c.834G>A	p.(Trp278Ter)	Stopgain	Pathogenic	M	9	Not stated
18120283	908	AIPL1	NM_014336.5	c.465+1G>C	Splice	Splice	Likely Pathogenic	F	6	Not stated
18120283	908	AIPL1	NM_014336.5	c.465+1G>C	Splice	Splice	Likely Pathogenic	F	6	Not stated
17903878	909	AIPL1	NM_014336.5	c.834G>A	p.(Trp278Ter)	Stopgain	Pathogenic	F	6	Not stated
17903878	909	AIPL1	NM_014336.5	c.834G>A	p.(Trp278Ter)	Stopgain	Pathogenic	F	6	Not stated
18511023	910	AIPL1	NM_014336.5	c.834G>A	p.(Trp278Ter)	Stopgain	Pathogenic	M	6	Asian - Bangladeshi
18511023	910	AIPL1	NM_014336.5	c.834G>A	p.(Trp278Ter)	Stopgain	Pathogenic	M	6	Asian - Bangladeshi
18653053	911	AIPL1	NM_014336.5	c.834G>A	p.(Trp278Ter)	Stopgain	Pathogenic	M	4	Any other ethnic group
18653053	911	AIPL1	NM_014336.5	c.834G>A	p.(Trp278Ter)	Stopgain	Pathogenic	M	4	Any other ethnic group
411571	912	ALMS1	NM_001378454.1	c.10775del	p.(Thr3592LysfsTer6)	Frameshift	Likely Pathogenic	M	48	White - British
411571	912	ALMS1	NM_001378454.1	c.5753T>A	p.(Leu1918Ter)	Stopgain	Likely Pathogenic	M	48	White - British
12633088	913	ALMS1	NM_001378454.1	c.2961_2962ins13	p.(Thr988LeufsTer6)	Frameshift	Likely Pathogenic	M	16	Not stated
12633088	913	ALMS1	NM_001378454.1	c.2961_2962ins13	p.(Thr988LeufsTer6)	Frameshift	Likely Pathogenic	M	16	Not stated
5511330	914	ALMS1	NM_001378454.1	c.1793del	p.(Glu598GlyfsTer3)	Frameshift	Likely Pathogenic	M	36	Asian - Other
5511330	914	ALMS1	NM_001378454.1	c.284del	p.(Pro95ArgfsTer19)	Frameshift	Likely Pathogenic	M	36	Asian - Other
3563286	915	AMACR	NM_014324.6	c.154T>C	p.(Ser52Pro)	Missense	Pathogenic	F	64	White - British
3563286	915	AMACR	NM_014324.6	c.154T>C	p.(Ser52Pro)	Missense	Pathogenic	F	64	White - British
10667936	915	AMACR	NM_014324.6	c.154T>C	p.(Ser52Pro)	Missense	Pathogenic	F	60	White - British
10667936	915	AMACR	NM_014324.6	c.154T>C	p.(Ser52Pro)	Missense	Pathogenic	F	60	White - British
3934524	916	ARHGEF18	NM_001367823.1	c.2632G>T	p.(Glu878Ter)	Stopgain	Likely Pathogenic	M	59	White - British
3934524	916	ARHGEF18	NM_001367823.1	c.2738_2761del	p.(Arg913_Glu920del)	Inframe indel	Variant of Uncertain Significance	M	59	White - British
9933503	917	ARHGEF18	NM_001367823.1	c.2181+5G>A	Splice	Splice	Likely Pathogenic	F	46	Not stated
9933503	917	ARHGEF18	NM_001367823.1	c.2181+5G>A	Splice	Splice	Likely Pathogenic	F	46	Not stated
10489772	918	ARHGEF18	NM_001367823.1	c.2560C>T	p.(Arg854Ter)	Stopgain	Pathogenic	F	45	White - British
10489772	918	ARHGEF18	NM_001367823.1	c.808A>G	p.(Thr270Ala)	Missense	Variant of Uncertain Significance	F	45	White - British
8308642	919	ATF6	NM_007348.4	c.1018G>C	p.(Ala340Pro)	Missense	Variant of Uncertain Significance	F	31	Asian - Bangladeshi
8308642	919	ATF6	NM_007348.4	c.1018G>C	p.(Ala340Pro)	Missense	Variant of Uncertain Significance	F	31	Asian - Bangladeshi
12922524	920	ATF6	NM_007348.4	c.417dup	p.(Asn140Ter)	Stopgain	Pathogenic	F	34	Not stated
12922524	920	ATF6	NM_007348.4	c.417dup	p.(Asn140Ter)	Stopgain	Pathogenic	F	34	Not stated
3602486	921	BBS1	NM_024649.5	c.1169T>G	p.(Met390Arg)	Missense	Pathogenic	M	60	Not stated
3602486	921	BBS1	NM_024649.5	c.1169T>G	p.(Met390Arg)	Missense	Pathogenic	M	60	Not stated
4589101	922	BBS1	NM_024649.5	c.1169T>G	p.(Met390Arg)	Missense	Pathogenic	M	39	White - British
4589101	922	BBS1	NM_024649.5	c.1570_1572del	p.(Asn524del)	Inframe indel	Variant of Uncertain Significance	M	39	White - British
6016338	923	BBS1	NM_024649.5	c.1169T>G	p.(Met390Arg)	Missense	Pathogenic	M	37	White - British
6016338	923	BBS1	NM_024649.5	c.1169T>G	p.(Met390Arg)	Missense	Pathogenic	M	37	White - British
5836690	924	BBS1	NM_024649.5	c.1169T>G	p.(Met390Arg)	Missense	Pathogenic	M	58	Not stated
5836690	924	BBS1	NM_024649.5	c.1169T>G	p.(Met390Arg)	Missense	Pathogenic	M	58	Not stated
7037967	925	BBS1	NM_024649.5	c.479G>A	p.(Arg160Gln)	Missense	Pathogenic	M	39	White - Other

7037967	925	BBS1	NM_024649.5	c.479G>A	p.(Arg160Gln)	Missense	Pathogenic	M	39	White - Other
827658	926	BBS1	NM_024649.5	c.1169T>G	p.(Met390Arg)	Missense	Pathogenic	F	70	White - British
827658	926	BBS1	NM_024649.5	c.1169T>G	p.(Met390Arg)	Missense	Pathogenic	F	70	White - British
9361190	927	BBS1	NM_024649.5	c.479G>A	p.(Arg160Gln)	Missense	Pathogenic	F	29	White - Other
9361190	927	BBS1	NM_024649.5	c.479G>A	p.(Arg160Gln)	Missense	Pathogenic	F	29	White - Other
9903228	928	BBS1	NM_024649.5	c.1169T>G	p.(Met390Arg)	Missense	Pathogenic	M	36	Unknown
9903228	928	BBS1	NM_024649.5	c.951+58C>T	Splice	Splice	Likely Pathogenic	M	36	Unknown
8069634	929	BBS1	NM_024649.5	c.1169T>G	p.(Met390Arg)	Missense	Pathogenic	M	58	White - British
8069634	929	BBS1	NM_024649.5	c.1169T>G	p.(Met390Arg)	Missense	Pathogenic	M	58	White - British
91699	930	BBS1	NM_024649.5	c.1169T>G	p.(Met390Arg)	Missense	Pathogenic	M	57	White - British
91699	930	BBS1	NM_024649.5	c.1169T>G	p.(Met390Arg)	Missense	Pathogenic	M	57	White - British
10534250	931	BBS1	NM_024649.5	c.1169T>G	p.(Met390Arg)	Missense	Pathogenic	F	61	Not stated
10534250	931	BBS1	NM_024649.5	c.1169T>G	p.(Met390Arg)	Missense	Pathogenic	F	61	Not stated
12806590	932	BBS1	NM_024649.5	c.1169T>G	p.(Met390Arg)	Missense	Pathogenic	F	43	Not stated
12806590	932	BBS1	NM_024649.5	c.1169T>G	p.(Met390Arg)	Missense	Pathogenic	F	43	Not stated
10050095	933	BBS1	NM_024649.5	c.1169T>G	p.(Met390Arg)	Missense	Pathogenic	F	73	Not stated
10050095	933	BBS1	NM_024649.5	c.1169T>G	p.(Met390Arg)	Missense	Pathogenic	F	73	Not stated
12225044	934	BBS1	NM_024649.5	c.1169T>G	p.(Met390Arg)	Missense	Pathogenic	F	26	White - British
12225044	934	BBS1	NM_024649.5	c.1169T>G	p.(Met390Arg)	Missense	Pathogenic	F	26	White - British
7298521	935	BBS1	NM_024649.5	c.1169T>G	p.(Met390Arg)	Missense	Pathogenic	M	38	White - British
7298521	935	BBS1	NM_024649.5	c.1169T>G	p.(Met390Arg)	Missense	Pathogenic	M	38	White - British
7613738	936	BBS1	NM_024649.5	c.1169T>G	p.(Met390Arg)	Missense	Pathogenic	M	46	Not stated
7613738	936	BBS1	NM_024649.5	c.1169T>G	p.(Met390Arg)	Missense	Pathogenic	M	46	Not stated
10086516	937	BBS1	NM_024649.5	c.1169T>G	p.(Met390Arg)	Missense	Pathogenic	F	39	White - British
10086516	937	BBS1	NM_024649.5	c.1169T>G	p.(Met390Arg)	Missense	Pathogenic	F	39	White - British
15004324	938	BBS1	NM_024649.5	c.479G>A	p.(Arg160Gln)	Missense	Pathogenic	M	26	Not stated
15004324	938	BBS1	NM_024649.5	c.479G>A	p.(Arg160Gln)	Missense	Pathogenic	M	26	Not stated
12306692	938	BBS1	NM_024649.5	c.479G>A	p.(Arg160Gln)	Missense	Pathogenic	F	23	Not stated
12306692	938	BBS1	NM_024649.5	c.479G>A	p.(Arg160Gln)	Missense	Pathogenic	F	23	Not stated
15557779	939	BBS1	NM_024649.5	c.1169T>G	p.(Met390Arg)	Missense	Pathogenic	M	48	White - British
15557779	939	BBS1	NM_024649.5	c.1169T>G	p.(Met390Arg)	Missense	Pathogenic	M	48	White - British
13524587	940	BBS1	NM_024649.5	c.1169T>G	p.(Met390Arg)	Missense	Pathogenic	F	22	White - British
13524587	940	BBS1	NM_024649.5	c.1643dup	p.(Glu549GlyfsTer9)	Frameshift	Pathogenic	F	22	White - British
16477187	941	BBS1	NM_024649.5	c.1169T>G	p.(Met390Arg)	Missense	Pathogenic	F	29	White - British
16477187	941	BBS1	NM_024649.5	c.1169T>G	p.(Met390Arg)	Missense	Pathogenic	F	29	White - British
17100082	942	BBS1	NM_024649.5	c.1169T>G	p.(Met390Arg)	Missense	Pathogenic	F	33	White - British
17100082	942	BBS1	NM_024649.5	c.1169T>G	p.(Met390Arg)	Missense	Pathogenic	F	33	White - British
16889305	943	BBS1	NM_024649.5	c.1169T>G	p.(Met390Arg)	Missense	Pathogenic	F	32	Not stated
16889305	943	BBS1	NM_024649.5	c.1169T>G	p.(Met390Arg)	Missense	Pathogenic	F	32	Not stated
8199239	944	BBS1	NM_024649.5	c.1169T>G	p.(Met390Arg)	Missense	Pathogenic	M	67	White - British
8199239	944	BBS1	NM_024649.5	c.1169T>G	p.(Met390Arg)	Missense	Pathogenic	M	67	White - British
15770103	945	BBS1	NM_024649.5	c.1169T>G	p.(Met390Arg)	Missense	Pathogenic	F	35	White - British
15770103	945	BBS1	NM_024649.5	c.1169T>G	p.(Met390Arg)	Missense	Pathogenic	F	35	White - British
16227308	946	BBS1	NM_024649.5	c.1169T>G	p.(Met390Arg)	Missense	Pathogenic	F	30	Not stated
16227308	946	BBS1	NM_024649.5	c.1169T>G	p.(Met390Arg)	Missense	Pathogenic	F	30	Not stated
17565673	947	BBS1	NM_024649.5	c.1169T>G	p.(Met390Arg)	Missense	Pathogenic	F	21	Not stated
17565673	947	BBS1	NM_024649.5	c.1169T>G	p.(Met390Arg)	Missense	Pathogenic	F	21	Not stated
17868353	948	BBS1	NM_024649.5	c.1169T>G	p.(Met390Arg)	Missense	Pathogenic	F	44	Not stated
17868353	948	BBS1	NM_024649.5	c.1169T>G	p.(Met390Arg)	Missense	Pathogenic	F	44	Not stated
8041067	949	BBS1	NM_024649.5	c.1318C>T	p.(Arg440Ter)	Stopgain	Pathogenic	M	35	Asian - Bangladeshi
8041067	949	BBS1	NM_024649.5	c.1339G>A	p.(Ala447Thr)	Missense	Likely Pathogenic	M	35	Asian - Bangladeshi

18450165	950	BBS1	NM_024649.5	c.1169T>G	p.(Met390Arg)	Missense	Pathogenic	F	26	Not stated
18450165	950	BBS1	NM_024649.5	c.1169T>G	p.(Met390Arg)	Missense	Pathogenic	F	26	Not stated
18301541	951	BBS1	NM_024649.5	c.1169T>G	p.(Met390Arg)	Missense	Pathogenic	F	17	Not stated
18301541	951	BBS1	NM_024649.5	c.1570_1572del	p.(Asn524del)	Inframe indel	Variant of Uncertain Significance	F	17	Not stated
7284192	952	BBS10	NM_024685.4	c.2119_2120del	p.(Val707Ter)	Stopgain	Pathogenic	F	36	White - British
7284192	952	BBS10	NM_024685.4	c.285A>T	p.(Arg95Ser)	Missense	Variant of Uncertain Significance	F	36	White - British
8255183	953	BBS10	NM_024685.4	c.226C>T	p.(Leu76Phe)	Missense	Variant of Uncertain Significance	F	38	White - Other
8255183	953	BBS10	NM_024685.4	c.271dup	p.(Cys91LeufsTer5)	Frameshift	Pathogenic	F	38	White - Other
10009817	954	BBS10	NM_024685.4	c.306_309del	p.(Asp102GlufsTer6)	Frameshift	Pathogenic	F	32	Not stated
10009817	954	BBS10	NM_024685.4	c.899A>C	p.(His300Pro)	Missense	Variant of Uncertain Significance	F	32	Not stated
12977544	955	BBS10	NM_024685.4	c.271dup	p.(Cys91LeufsTer5)	Frameshift	Pathogenic	M	18	White - British
12977544	955	BBS10	NM_024685.4	c.590A>G	p.(Tyr197Cys)	Missense	Pathogenic	M	18	White - British
7211525	956	BBS10	NM_024685.4	c.271dup	p.(Cys91LeufsTer5)	Frameshift	Pathogenic	F	40	Not stated
7211525	956	BBS10	NM_024685.4	c.271dup	p.(Cys91LeufsTer5)	Frameshift	Pathogenic	F	40	Not stated
16688776	957	BBS10	NM_024685.4	c.118A>T	p.(Lys40Ter)	Stopgain	Pathogenic	F	40	Not stated
16688776	957	BBS10	NM_024685.4	c.271dup	p.(Cys91LeufsTer5)	Frameshift	Pathogenic	F	40	Not stated
2773049	958	BBS12	NM_152618.3	c.1418_1420del	p.(Phe473del)	Inframe indel	Variant of Uncertain Significance	M	60	Not stated
2773049	958	BBS12	NM_152618.3	c.1418_1420del	p.(Phe473del)	Inframe indel	Variant of Uncertain Significance	M	60	Not stated
3633839	959	BBS12	NM_152618.3	c.1055A>C	p.(Gln352Pro)	Missense	Variant of Uncertain Significance	M	47	Not stated
3633839	959	BBS12	NM_152618.3	c.1055A>C	p.(Gln352Pro)	Missense	Variant of Uncertain Significance	M	47	Not stated
13524587	940	BBS12	NM_152618.3	c.714dup	p.(Arg239Ter)	Stopgain	Likely Pathogenic	F	22	White - British
7934422	960	BBS2	NM_031885.5	c.401C>G	p.(Pro134Arg)	Missense	Pathogenic	F	45	White - British
7934422	960	BBS2	NM_031885.5	c.72C>G	p.(Tyr24Ter)	Stopgain	Pathogenic	F	45	White - British
15812194	961	BBS2	NM_031885.5	c.2115_2116del	p.(Asn705LysfsTer22)	Frameshift	Variant of Uncertain Significance	M	19	Any other ethnic group
15812194	961	BBS2	NM_031885.5	c.534+1G>T	Splice	Splice	Pathogenic	M	19	Any other ethnic group
12917071	962	BBS2	NM_031885.5	c.700C>T	p.(Arg234Ter)	Stopgain	Pathogenic	M	46	Not stated
12917071	962	BBS2	NM_031885.5	c.700C>T	p.(Arg234Ter)	Stopgain	Pathogenic	M	46	Not stated
4664960	963	ARL6	NM_177976.3	c.272T>C	p.(Ile91Thr)	Missense	Likely Pathogenic	M	48	Asian - Indian
4664960	963	ARL6	NM_177976.3	c.272T>C	p.(Ile91Thr)	Missense	Likely Pathogenic	M	48	Asian - Indian
2514875	964	ARL6	NM_177976.3	c.281T>C	p.(Ile94Thr)	Missense	Likely Pathogenic	F	44	Asian - Pakistani
2514875	964	ARL6	NM_177976.3	c.281T>C	p.(Ile94Thr)	Missense	Likely Pathogenic	F	44	Asian - Pakistani
12374550	965	ARL6	NM_177976.3	c.528G>T	p.(Trp176Cys)	Missense	Variant of Uncertain Significance	M	45	White - Other
12374550	965	ARL6	NM_177976.3	c.528G>T	p.(Trp176Cys)	Missense	Variant of Uncertain Significance	M	45	White - Other
60220	966	BBS5	NM_152384.3	c.412C>T	p.(Arg138Cys)	Missense	Likely Pathogenic	F	74	White - British
60220	966	BBS5	NM_152384.3	c.900G>C	p.(Val300Val)	Synonymous	Variant of Uncertain Significance	F	74	White - British
8029433	967	BBS5	NM_152384.3	c.2T>A	p.(Met1?)	Start codon	Pathogenic	F	33	Asian - Pakistani
8029433	967	BBS5	NM_152384.3	c.2T>A	p.(Met1?)	Start codon	Pathogenic	F	33	Asian - Pakistani
18599860	968	TTC8	NM_144596.4	c.1253del	p.(Gln418ArgfsTer43)	Frameshift	Likely Pathogenic	M	12	Not stated
18599860	968	TTC8	NM_144596.4	c.1253del	p.(Gln418ArgfsTer43)	Frameshift	Likely Pathogenic	M	12	Not stated
4024089	969	BEST1	NM_004183.4	c.224T>C	p.(Leu75Pro)	Missense	Pathogenic	M	68	Unknown
4024103	969	BEST1	NM_004183.4	c.224T>C	p.(Leu75Pro)	Missense	Pathogenic	F	37	Unknown
2691821	970	BEST1	NM_004183.4	c.883A>G	p.(Ile295Val)	Missense	Likely Pathogenic	F	76	White - British
5513185	971	BEST1	NM_004183.4	c.692G>C	p.(Ser231Thr)	Missense	Likely Pathogenic	F	71	Not stated
11854940	971	BEST1	NM_004183.4	c.692G>C	p.(Ser231Thr)	Missense	Likely Pathogenic	M	25	White - British
5575940	972	BEST1	NM_004183.4	c.122T>C	p.(Leu41Pro)	Missense	Likely Pathogenic	F	67	White - British
5575940	972	BEST1	NM_004183.4	c.454C>G	p.(Pro152Ala)	Missense	Pathogenic	F	67	White - British
4733630	973	BEST1	NM_004183.4	c.422G>A	p.(Arg141His)	Missense	Pathogenic	F	68	Not stated
4733630	973	BEST1	NM_004183.4	c.949G>A	p.(Val317Met)	Missense	Likely Pathogenic	F	68	Not stated
5897821	974	BEST1	NM_004183.4	c.122T>C	p.(Leu41Pro)	Missense	Likely Pathogenic	F	50	Not stated
5897821	974	BEST1	NM_004183.4	c.422G>A	p.(Arg141His)	Missense	Pathogenic	F	50	Not stated
3922050	975	BEST1	NM_004183.4	c.-37+1G>T	Splice	Splice	Pathogenic	M	70	Any other ethnic group

3922050	975	BEST1	NM_004183.4	c.-37+1G>T	Splice	Splice	Pathogenic	M	70	Any other ethnic group
7169098	976	BEST1	NM_004183.4	c.728C>T	p.(Ala243Val)	Missense	Pathogenic	M	62	White - British
6369222	977	BEST1	NM_004183.4	c.279G>T	p.(Trp93Cys)	Missense	Likely Pathogenic	M	38	White - British
3823448	978	BEST1	NM_004183.4	c.324C>G	p.(Ser108Arg)	Missense	Likely Pathogenic	F	72	Not stated
13879326	978	BEST1	NM_004183.4	c.324C>G	p.(Ser108Arg)	Missense	Likely Pathogenic	M	14	Not stated
223866	979	BEST1	NM_004183.4	c.90G>C	p.(Lys30Asn)	Missense	Likely Pathogenic	F	53	White - British
17894127	980	BEST1	NM_004183.4	c.28G>A	p.(Ala10Thr)	Missense	Pathogenic	F	59	Black - Caribbean
4704461	981	BEST1	NM_004183.4	c.886A>C	p.(Asn296His)	Missense	Pathogenic	F	61	Not stated
796459	982	BEST1	NM_004183.4	c.652C>T	p.(Arg218Cys)	Missense	Pathogenic	M	61	Not stated
6433489	983	BEST1	NM_004183.4	c.703G>T	p.(Val235Leu)	Missense	Pathogenic	F	62	White - British
1014656	984	BEST1	NM_004183.4	c.916G>A	p.(Glu306Lys)	Missense	Likely Pathogenic	M	49	White - British
1970464	985	BEST1	NM_004183.4	c.5C>G	p.(Thr2Ser)	Missense	Pathogenic	F	38	White - British
8727858	986	BEST1	NM_004183.4	c.107_118del	p.(Phe36_Phe39del)	Inframe indel	Likely Pathogenic	F	52	Not stated
8727858	986	BEST1	NM_004183.4	c.107_118del	p.(Phe36_Phe39del)	Inframe indel	Likely Pathogenic	F	52	Not stated
4985798	987	BEST1	NM_004183.4	c.418C>G	p.(Leu140Val)	Missense	Pathogenic	M	39	Asian - Pakistani
4985798	987	BEST1	NM_004183.4	c.418C>G	p.(Leu140Val)	Missense	Pathogenic	M	39	Asian - Pakistani
4073852	988	BEST1	NM_004183.4	c.1014G>A	p.(Trp338Ter)	Stopgain	Likely Pathogenic	M	46	White - Other
4073852	988	BEST1	NM_004183.4	c.-37+1G>T	Splice	Splice	Pathogenic	M	46	White - Other
9009706	989	BEST1	NM_004183.4	c.1038dup	p.(Tyr347LeufsTer54)	Frameshift	Likely Pathogenic	F	34	Not stated
9009706	989	BEST1	NM_004183.4	c.533A>C	p.(His178Pro)	Missense	Variant of Uncertain Significance	F	34	Not stated
10138022	990	BEST1	NM_004183.4	c.914T>C	p.(Phe305Ser)	Missense	Pathogenic	F	49	Unknown
9881444	991	BEST1	NM_004183.4	c.636+1G>C	Splice	Splice	Likely Pathogenic	M	35	Not stated
9881444	991	BEST1	NM_004183.4	c.636+1G>C	Splice	Splice	Likely Pathogenic	M	35	Not stated
10067168	992	BEST1	NM_004183.4	c.914T>C	p.(Phe305Ser)	Missense	Pathogenic	F	21	Not stated
10023971	993	BEST1	NM_004183.4	c.1066C>T	p.(Arg356Ter)	Stopgain	Pathogenic	M	29	White - British
10023971	993	BEST1	NM_004183.4	Exon 1-2 deletion	Deletion	Deletion	Likely Pathogenic	M	29	White - British
10107229	994	BEST1	NM_004183.4	c.288G>C	p.(Gln96His)	Missense	Pathogenic	F	46	Not stated
10225277	995	BEST1	NM_004183.4	c.847T>C	p.(Phe283Leu)	Missense	Likely Pathogenic	F	51	Unknown
1415805	996	BEST1	NM_004183.4	c.418C>G	p.(Leu140Val)	Missense	Pathogenic	M	45	Asian - Pakistani
1415805	996	BEST1	NM_004183.4	c.418C>G	p.(Leu140Val)	Missense	Pathogenic	M	45	Asian - Pakistani
3597523	997	BEST1	NM_004183.4	c.1470_1471del	p.(His490GlnfsTer24)	Frameshift	Pathogenic	M	58	White - British
3597523	997	BEST1	NM_004183.4	c.102C>T	p.(Gly34Gly)	Synonymous	Likely Pathogenic	M	58	White - British
8011429	998	BEST1	NM_004183.4	c.905A>G	p.(Asp302Gly)	Missense	Pathogenic	M	29	Not stated
10318573	999	BEST1	NM_004183.4	c.652C>T	p.(Arg218Cys)	Missense	Pathogenic	M	51	Not stated
8575062	1000	BEST1	NM_004183.4	c.536_538del	p.(Asn179del)	Inframe indel	Likely Pathogenic	M	44	Asian - Bangladeshi
8575062	1000	BEST1	NM_004183.4	c.536_538del	p.(Asn179del)	Inframe indel	Likely Pathogenic	M	44	Asian - Bangladeshi
10340315	1001	BEST1	NM_004183.4	c.986T>C	p.(Leu329Pro)	Missense	Variant of Uncertain Significance	M	20	Unknown
10340315	1001	BEST1	NM_004183.4	c.905A>C	p.(Asp302Ala)	Missense	Likely Pathogenic	M	20	Unknown
5864417	1002	BEST1	NM_004183.4	c.37C>T	p.(Arg13Cys)	Missense	Likely Pathogenic	M	59	White - British
9885994	1003	BEST1	NM_004183.4	c.901G>A	p.(Asp301Asn)	Missense	Likely Pathogenic	F	44	White - Other
10911851	1004	BEST1	NM_004183.4	c.728C>T	p.(Ala243Val)	Missense	Pathogenic	F	27	White - British
10945388	1004	BEST1	NM_004183.4	c.728C>T	p.(Ala243Val)	Missense	Pathogenic	F	70	White - British
10684015	1005	BEST1	NM_004183.4	c.691A>C	p.(Ser231Arg)	Missense	Pathogenic	M	20	Not stated
10812157	1006	BEST1	NM_004183.4	c.1066C>T	p.(Arg356Ter)	Stopgain	Pathogenic	M	26	Any other ethnic group
10812157	1006	BEST1	NM_004183.4	c.602T>C	p.(Ile201Thr)	Missense	Pathogenic	M	26	Any other ethnic group
11327847	1007	BEST1	NM_004183.4	c.47C>T	p.(Ser16Phe)	Missense	Pathogenic	F	33	Black - African
10870670	1008	BEST1	NM_004183.4	c.906T>G	p.(Asp302Glu)	Missense	Pathogenic	F	57	Any other ethnic group
9843623	1009	BEST1	NM_004183.4	c.902A>G	p.(Asp301Gly)	Missense	Pathogenic	M	52	White - British
11334693	1010	BEST1	NM_004183.4	c.877C>A	p.(Gln293Lys)	Missense	Pathogenic	F	47	Unknown
845935	1011	BEST1	NM_004183.4	c.892T>G	p.(Phe298Val)	Missense	Likely Pathogenic	M	76	Unknown
3057683	1012	BEST1	NM_004183.4	c.652C>T	p.(Arg218Cys)	Missense	Pathogenic	F	56	Any other ethnic group

11594750	1013	BEST1	NM_004183.4	c.73C>T	p.(Arg25Trp)	Missense	Pathogenic	M	51	Not stated
7694567	1014	BEST1	NM_004183.4	c.44G>A	p.(Gly15Asp)	Missense	Pathogenic	M	47	Asian - Bangladeshi
12079297	1015	BEST1	NM_004183.4	c.728C>T	p.(Ala243Val)	Missense	Pathogenic	F	44	Not stated
6523495	1016	BEST1	NM_004183.4	c.253T>C	p.(Tyr85His)	Missense	Likely Pathogenic	M	30	Not stated
12037220	1017	BEST1	NM_004183.4	c.16A>C	p.(Thr6Pro)	Missense	Pathogenic	M	44	White - Other
12251000	1018	BEST1	NM_004183.4	c.826T>C	p.(Phe276Leu)	Missense	Variant of Uncertain Significance	M	22	Unknown
12217197	1019	BEST1	NM_004183.4	c.874G>A	p.(Glu292Lys)	Missense	Pathogenic	F	26	Not stated
12437382	1019	BEST1	NM_004183.4	c.874G>A	p.(Glu292Lys)	Missense	Pathogenic	M	55	Not stated
847181	1020	BEST1	NM_004183.4	c.37C>T	p.(Arg13Cys)	Missense	Likely Pathogenic	M	76	White - British
7256577	1021	BEST1	NM_004183.4	c.652C>T	p.(Arg218Cys)	Missense	Pathogenic	M	44	Not stated
13027755	1021	BEST1	NM_004183.4	c.652C>T	p.(Arg218Cys)	Missense	Pathogenic	F	47	Asian - Other
3060196	1022	BEST1	NM_004183.4	c.28G>A	p.(Ala10Thr)	Missense	Pathogenic	F	61	Black - Caribbean
1247777	1023	BEST1	NM_004183.4	c.874G>A	p.(Glu292Lys)	Missense	Pathogenic	M	47	Not stated
12421079	1024	BEST1	NM_004183.4	c.47C>T	p.(Ser16Phe)	Missense	Pathogenic	M	72	White - British
8545781	1024	BEST1	NM_004183.4	c.47C>T	p.(Ser16Phe)	Missense	Pathogenic	F	59	White - British
13744716	1024	BEST1	NM_004183.4	c.47C>T	p.(Ser16Phe)	Missense	Pathogenic	M	26	Not stated
1634135	1025	BEST1	NM_004183.4	c.703G>A	p.(Val235Met)	Missense	Likely Pathogenic	M	82	White - Other
12275514	1026	BEST1	NM_004183.4	c.139C>T	p.(Arg47Cys)	Missense	Pathogenic	M	23	Any other ethnic group
12275514	1026	BEST1	NM_004183.4	c.139C>T	p.(Arg47Cys)	Missense	Pathogenic	M	23	Any other ethnic group
5784477	1027	BEST1	NM_004183.4	c.652C>T	p.(Arg218Cys)	Missense	Pathogenic	F	39	White - British
10212845	1028	BEST1	NM_004183.4	c.728C>T	p.(Ala243Val)	Missense	Pathogenic	F	74	Not stated
12179306	1029	BEST1	NM_004183.4	c.310G>C	p.(Asp104His)	Missense	Likely Pathogenic	M	55	Asian - Pakistani
12868876	1030	BEST1	NM_004183.4	c.652C>T	p.(Arg218Cys)	Missense	Pathogenic	M	58	White - British
13010822	1031	BEST1	NM_004183.4	c.652C>T	p.(Arg218Cys)	Missense	Pathogenic	M	19	White - British
9807846	1032	BEST1	NM_004183.4	c.653G>A	p.(Arg218His)	Missense	Pathogenic	M	33	Unknown
12519744	1033	BEST1	NM_004183.4	c.439G>T	p.(Val147Phe)	Missense	Likely Pathogenic	M	62	White - British
5365478	1034	BEST1	NM_004183.4	c.682G>A	p.(Asp228Asn)	Missense	Pathogenic	F	67	White - Other
12911940	1035	BEST1	NM_004183.4	c.728C>T	p.(Ala243Val)	Missense	Pathogenic	M	65	Not stated
13676865	1035	BEST1	NM_004183.4	c.728C>T	p.(Ala243Val)	Missense	Pathogenic	M	90	Unknown
12791442	1036	BEST1	NM_004183.4	c.74G>T	p.(Arg25Leu)	Missense	Likely Pathogenic	F	44	Unknown
3054169	1037	BEST1	NM_004183.4	c.886A>C	p.(Asn296His)	Missense	Pathogenic	M	68	White - Irish
12679323	1038	BEST1	NM_004183.4	c.974T>C	p.(Met325Thr)	Missense	Variant of Uncertain Significance	M	56	Unknown
12679323	1038	BEST1	NM_004183.4	c.602T>C	p.(Ile201Thr)	Missense	Pathogenic	M	56	Unknown
13370895	1039	BEST1	NM_004183.4	c.868-2A>G	Splice	Splice	Likely Pathogenic	F	62	Unknown
13370895	1039	BEST1	NM_004183.4	c.868-2A>G	Splice	Splice	Likely Pathogenic	F	62	Unknown
337252	1040	BEST1	NM_004183.4	c.5C>G	p.(Thr2Ser)	Missense	Pathogenic	M	82	Not stated
13221459	1041	BEST1	NM_004183.4	c.892T>G	p.(Phe298Val)	Missense	Likely Pathogenic	M	38	Any other ethnic group
13638855	1042	BEST1	NM_004183.4	c.122T>C	p.(Leu41Pro)	Missense	Likely Pathogenic	F	21	Not stated
13638855	1042	BEST1	NM_004183.4	c.291C>G	p.(Tyr97Ter)	Stopgain	Likely Pathogenic	F	21	Not stated
3577419	1043	BEST1	NM_004183.4	c.28G>A	p.(Ala10Thr)	Missense	Pathogenic	M	48	Unknown
13753417	1044	BEST1	NM_004183.4	c.653G>A	p.(Arg218His)	Missense	Pathogenic	M	28	Not stated
13753823	1045	BEST1	NM_004183.4	c.288G>C	p.(Gln96His)	Missense	Pathogenic	M	14	Not stated
13432474	1046	BEST1	NM_004183.4	c.728C>T	p.(Ala243Val)	Missense	Pathogenic	F	71	Unknown
14890728	1047	BEST1	NM_004183.4	c.223C>G	p.(Leu75Val)	Missense	Likely Pathogenic	F	18	White - British
14732423	1048	BEST1	NM_004183.4	c.278G>A	p.(Trp93Ter)	Stopgain	Likely Pathogenic	M	20	Unknown
14732423	1048	BEST1	NM_004183.4	c.74G>A	p.(Arg25Gln)	Missense	Pathogenic	M	20	Unknown
14865052	1049	BEST1	NM_004183.4	c.653G>A	p.(Arg218His)	Missense	Pathogenic	M	15	Unknown
15005381	1050	BEST1	NM_004183.4	c.294G>T	p.(Glu98Asp)	Missense	Pathogenic	M	57	Unknown
15056908	1051	BEST1	NM_004183.4	c.169G>T	p.(Glu57Ter)	Stopgain	Pathogenic	M	39	Any other ethnic group
15056908	1051	BEST1	NM_004183.4	c.530C>T	p.(Pro177Leu)	Missense	Variant of Uncertain Significance	M	39	Any other ethnic group
13391307	1052	BEST1	NM_004183.4	c.29C>T	p.(Ala10Val)	Missense	Likely Pathogenic	F	33	Not stated

13391307	1052	BEST1	NM_004183.4	c.422G>A	p.(Arg141His)	Missense	Pathogenic	F	33	Not stated
13620256	1053	BEST1	NM_004183.4	c.653G>A	p.(Arg218His)	Missense	Pathogenic	M	60	Unknown
15208738	1054	BEST1	NM_004183.4	c.903T>A	p.(Asp301Glu)	Missense	Pathogenic	M	27	White - British
13429702	1054	BEST1	NM_004183.4	c.903T>A	p.(Asp301Glu)	Missense	Pathogenic	M	71	Any other ethnic group
15268280	1055	BEST1	NM_004183.4	c.345del	p.(Gly116AlafsTer48)	Frameshift	Likely Pathogenic	M	15	Unknown
14708294	1056	BEST1	NM_004183.4	c.313C>G	p.(Arg105Gly)	Missense	Likely Pathogenic	M	42	Not stated
15602943	1057	BEST1	NM_004183.4	c.26T>C	p.(Val9Ala)	Missense	Likely Pathogenic	M	15	Not stated
15285836	1058	BEST1	NM_004183.4	c.653G>A	p.(Arg218His)	Missense	Pathogenic	M	50	Any other ethnic group
201753	1059	BEST1	NM_004183.4	c.602T>C	p.(Ile201Thr)	Missense	Pathogenic	M	47	Not stated
201753	1059	BEST1	NM_004183.4	Exon 1-2 deletion	Deletion	Deletion	Likely Pathogenic	M	47	Not stated
15754717	1060	BEST1	NM_004183.4	c.1120dup	p.(Glu374GlyfsTer27)	Frameshift	Pathogenic	M	42	Not stated
15754717	1060	BEST1	NM_004183.4	c.974T>C	p.(Met325Thr)	Missense	Variant of Uncertain Significance	M	42	Not stated
5861876	1061	BEST1	NM_004183.4	c.229C>T	p.(Pro77Ser)	Missense	Likely Pathogenic	M	68	Not stated
15849700	1062	BEST1	NM_004183.4	c.728C>A	p.(Ala243Glu)	Missense	Likely Pathogenic	F	38	White - Other
15849700	1062	BEST1	NM_004183.4	c.728C>A	p.(Ala243Glu)	Missense	Likely Pathogenic	F	38	White - Other
9015180	1063	BEST1	NM_004183.4	c.90G>C	p.(Lys30Asn)	Missense	Likely Pathogenic	F	27	White - British
15696596	1064	BEST1	NM_004183.4	c.1038dup	p.(Tyr347LeufsTer54)	Frameshift	Likely Pathogenic	F	70	White - British
15696596	1064	BEST1	NM_004183.4	c.421C>A	p.(Arg141Ser)	Missense	Pathogenic	F	70	White - British
4615785	1065	BEST1	NM_004183.4	c.703G>A	p.(Val235Met)	Missense	Likely Pathogenic	M	53	White - British
10247327	1066	BEST1	NM_004183.4	c.74G>A	p.(Arg25Gln)	Missense	Pathogenic	M	43	Mixed - Other
15429056	1067	BEST1	NM_004183.4	c.892T>G	p.(Phe298Val)	Missense	Likely Pathogenic	M	11	Not stated
16534608	1068	BEST1	NM_004183.4	c.287A>G	p.(Gln96Arg)	Missense	Likely Pathogenic	F	22	Not stated
15744014	1069	BEST1	NM_004183.4	c.718_720dup	p.(Val240dup)	Inframe indel	Likely Pathogenic	F	41	Not stated
16577518	1070	BEST1	NM_004183.4	c.418C>G	p.(Leu140Val)	Missense	Pathogenic	F	13	Not stated
16577518	1070	BEST1	NM_004183.4	c.418C>G	p.(Leu140Val)	Missense	Pathogenic	F	13	Not stated
3509939	1071	BEST1	NM_004183.4	c.653G>A	p.(Arg218His)	Missense	Pathogenic	F	72	Not stated
16677933	1072	BEST1	NM_004183.4	c.37C>T	p.(Arg13Cys)	Missense	Likely Pathogenic	M	86	White - British
16528049	1073	BEST1	NM_004183.4	c.914T>C	p.(Phe305Ser)	Missense	Pathogenic	M	16	White - British
12475273	1074	BEST1	NM_004183.4	c.728C>T	p.(Ala243Val)	Missense	Pathogenic	F	64	Unknown
16431708	1074	BEST1	NM_004183.4	c.728C>T	p.(Ala243Val)	Missense	Pathogenic	M	34	Not stated
16454346	1075	BEST1	NM_004183.4	c.883A>G	p.(Ile295Val)	Missense	Likely Pathogenic	F	74	Not stated
16411177	1076	BEST1	NM_004183.4	c.653G>A	p.(Arg218His)	Missense	Pathogenic	M	34	Not stated
16688741	1076	BEST1	NM_004183.4	c.653G>A	p.(Arg218His)	Missense	Pathogenic	F	52	Not stated
7688120	1077	BEST1	NM_004183.4	c.652C>T	p.(Arg218Cys)	Missense	Pathogenic	M	64	White - British
16101847	1078	BEST1	NM_004183.4	c.302C>A	p.(Pro101Gln)	Missense	Pathogenic	F	44	Black - African
12888161	1079	BEST1	NM_004183.4	c.889C>T	p.(Pro297Ser)	Missense	Pathogenic	F	37	Not stated
17058845	1080	BEST1	NM_004183.4	c.728C>T	p.(Ala243Val)	Missense	Pathogenic	F	64	White - Other
16885868	1081	BEST1	NM_004183.4	c.310G>T	p.(Asp104Tyr)	Missense	Likely Pathogenic	M	44	Not stated
16504865	1082	BEST1	NM_004183.4	c.44G>A	p.(Gly15Asp)	Missense	Pathogenic	M	50	Asian - Bangladeshi
6972888	1083	BEST1	NM_004183.4	c.74G>T	p.(Arg25Leu)	Missense	Likely Pathogenic	M	29	White - British
3584594	1084	BEST1	NM_004183.4	c.889C>T	p.(Pro297Ser)	Missense	Pathogenic	F	32	Mixed - White and Asian
17122615	1085	BEST1	NM_004183.4	c.883A>G	p.(Ile295Val)	Missense	Likely Pathogenic	M	76	Not stated
16371788	1086	BEST1	NM_004183.4	c.653G>A	p.(Arg218His)	Missense	Pathogenic	M	28	White - British
17088427	1087	BEST1	NM_004183.4	c.887A>G	p.(Asn296Ser)	Missense	Pathogenic	M	70	Any other ethnic group
17055989	1088	BEST1	NM_004183.4	c.422G>A	p.(Arg141His)	Missense	Pathogenic	F	28	White - British
17055989	1088	BEST1	NM_004183.4	c.839A>C	p.(Gln280Pro)	Missense	Likely Pathogenic	F	28	White - British
16948595	1089	BEST1	NM_004183.4	c.1087A>C	p.(Thr363Pro)	Missense	Variant of Uncertain Significance	M	50	Black - African
16948595	1089	BEST1	NM_004183.4	c.1087A>C	p.(Thr363Pro)	Missense	Variant of Uncertain Significance	M	50	Black - African
17315066	1090	BEST1	NM_004183.4	c.310G>C	p.(Asp104His)	Missense	Likely Pathogenic	M	51	Not stated
17453155	1091	BEST1	NM_004183.4	c.653G>A	p.(Arg218His)	Missense	Pathogenic	F	84	Unknown
16900078	1092	BEST1	NM_004183.4	c.241G>A	p.(Val81Met)	Missense	Pathogenic	M	63	Not stated

17068995	1093	BEST1	NM_004183.4	c.584C>T	p.(Ala195Val)	Missense	Pathogenic	M	45	Not stated
17068995	1093	BEST1	NM_004183.4	c.636+1G>A	Splice	Splice	Pathogenic	M	45	Not stated
17806214	1094	BEST1	NM_004183.4	c.826T>C	p.(Phe276Leu)	Missense	Variant of Uncertain Significance	M	53	Not stated
17386270	1095	BEST1	NM_004183.4	c.652C>T	p.(Arg218Cys)	Missense	Pathogenic	F	52	Any other ethnic group
16382743	1096	BEST1	NM_004183.4	c.652C>T	p.(Arg218Cys)	Missense	Pathogenic	M	20	Not stated
16417764	1097	BEST1	NM_004183.4	c.128A>G	p.(Tyr43Cys)	Missense	Likely Pathogenic	M	33	Not stated
16417764	1097	BEST1	NM_004183.4	c.128A>G	p.(Tyr43Cys)	Missense	Likely Pathogenic	M	33	Not stated
10074924	1098	BEST1	NM_004183.4	c.302C>G	p.(Pro101Arg)	Missense	Pathogenic	M	46	Any other ethnic group
17544974	1099	BEST1	NM_004183.4	c.821C>G	p.(Pro274Arg)	Missense	Pathogenic	F	22	Not stated
17544974	1099	BEST1	NM_004183.4	c.821C>G	p.(Pro274Arg)	Missense	Pathogenic	F	22	Not stated
18016354	1100	BEST1	NM_004183.4	c.1087A>C	p.(Thr363Pro)	Missense	Variant of Uncertain Significance	F	14	Not stated
18016354	1100	BEST1	NM_004183.4	c.1087A>C	p.(Thr363Pro)	Missense	Variant of Uncertain Significance	F	14	Not stated
18142613	1101	BEST1	NM_004183.4	c.652C>A	p.(Arg218Ser)	Missense	Pathogenic	M	21	Not stated
18142627	1101	BEST1	NM_004183.4	c.652C>A	p.(Arg218Ser)	Missense	Pathogenic	M	20	Not stated
18115061	1102	BEST1	NM_004183.4	c.253T>C	p.(Tyr85His)	Missense	Likely Pathogenic	M	67	Not stated
18275403	1103	BEST1	NM_004183.4	c.418C>G	p.(Leu140Val)	Missense	Pathogenic	F	40	Not stated
18275403	1103	BEST1	NM_004183.4	c.418C>G	p.(Leu140Val)	Missense	Pathogenic	F	40	Not stated
18268137	1104	BEST1	NM_004183.4	c.73C>T	p.(Arg25Trp)	Missense	Pathogenic	F	14	Not stated
18268137	1104	BEST1	NM_004183.4	c.73C>T	p.(Arg25Trp)	Missense	Pathogenic	F	14	Not stated
18077114	1105	BEST1	NM_004183.4	c.122T>C	p.(Leu41Pro)	Missense	Likely Pathogenic	F	26	Not stated
18077114	1105	BEST1	NM_004183.4	c.829A>T	p.(Thr277Ser)	Missense	Likely Pathogenic	F	26	Not stated
6310359	1106	BEST1	NM_004183.4	c.914T>C	p.(Phe305Ser)	Missense	Pathogenic	F	38	Not stated
18066915	1107	BEST1	NM_004183.4	c.90G>C	p.(Lys30Asn)	Missense	Likely Pathogenic	M	60	Not stated
10769954	1108	BEST1	NM_004183.4	c.468C>G	p.(His156Gln)	Missense	Variant of Uncertain Significance	M	54	Not stated
10769954	1108	BEST1	NM_004183.4	c.468C>G	p.(His156Gln)	Missense	Variant of Uncertain Significance	M	54	Not stated
4556187	1109	C21ORF2	NM_004928.3	c.218G>C	p.(Arg73Pro)	Missense	Pathogenic	F	28	Not stated
4556187	1109	C21ORF2	NM_004928.3	c.33_34insAGCTGCACAGCGTGCA	p.(Ala12SerfsTer60)	Frameshift	Pathogenic	F	28	Not stated
8325715	1110	C21ORF2	NM_004928.3	c.218G>C	p.(Arg73Pro)	Missense	Pathogenic	F	28	White - British
8325715	1110	C21ORF2	NM_004928.3	c.218G>C	p.(Arg73Pro)	Missense	Pathogenic	F	28	White - British
12476127	1111	C21ORF2	NM_004928.3	c.269G>C	p.(Arg90Pro)	Missense	Likely Pathogenic	F	57	White - British
12476127	1111	C21ORF2	NM_004928.3	c.33_34insAGCTGCACAGCGTGCA	p.(Ala12SerfsTer60)	Frameshift	Pathogenic	F	57	White - British
13647696	1112	C21ORF2	NM_004928.3	c.58G>A	p.(Val20Met)	Missense	Variant of Uncertain Significance	M	41	Not stated
13647696	1112	C21ORF2	NM_004928.3	c.58G>A	p.(Val20Met)	Missense	Variant of Uncertain Significance	M	41	Not stated
12956761	1113	C21ORF2	NM_004928.3	c.95_96del	p.(Ile33LeufsTer33)	Frameshift	Likely Pathogenic	M	36	Not stated
12956761	1113	C21ORF2	NM_004928.3	c.95_96del	p.(Ile33LeufsTer33)	Frameshift	Likely Pathogenic	M	36	Not stated
6923265	1114	C21ORF2	NM_004928.3	c.286G>A	p.(Glu96Lys)	Missense	Likely Pathogenic	M	56	White - Other
6923265	1114	C21ORF2	NM_004928.3	c.286G>A	p.(Glu96Lys)	Missense	Likely Pathogenic	M	56	White - Other
7503425	1115	PCARE	NM_001029883.3	c.1541del	p.(Pro514HisfsTer27)	Frameshift	Pathogenic	M	45	White - Other
7503425	1115	PCARE	NM_001029883.3	c.1541del	p.(Pro514HisfsTer27)	Frameshift	Pathogenic	M	45	White - Other
6468118	1116	PCARE	NM_001029883.3	c.1541del	p.(Pro514HisfsTer27)	Frameshift	Pathogenic	M	52	Not stated
6468118	1116	PCARE	NM_001029883.3	c.1541del	p.(Pro514HisfsTer27)	Frameshift	Pathogenic	M	52	Not stated
8167970	1117	PCARE	NM_001029883.3	c.344del	p.(Pro115ArgfsTer67)	Frameshift	Likely Pathogenic	M	47	White - Other
8167970	1117	PCARE	NM_001029883.3	c.344del	p.(Pro115ArgfsTer67)	Frameshift	Likely Pathogenic	M	47	White - Other
8058413	1118	PCARE	NM_001029883.3	c.2315_2321delinsAAG	p.(Thr772LysfsTer24)	Frameshift	Likely Pathogenic	M	49	Any other ethnic group
8058413	1118	PCARE	NM_001029883.3	c.2315_2321delinsAAG	p.(Thr772LysfsTer24)	Frameshift	Likely Pathogenic	M	49	Any other ethnic group
12250076	1119	PCARE	NM_001029883.3	c.3002G>A	p.(Trp1001Ter)	Stopgain	Pathogenic	M	46	Not stated
12250076	1119	PCARE	NM_001029883.3	c.3002G>A	p.(Trp1001Ter)	Stopgain	Pathogenic	M	46	Not stated
12835647	1120	PCARE	NM_001029883.3	c.758G>A	p.(Trp253Ter)	Stopgain	Pathogenic	M	45	Unknown
12835647	1120	PCARE	NM_001029883.3	c.758G>A	p.(Trp253Ter)	Stopgain	Pathogenic	M	45	Unknown
13634942	1121	PCARE	NM_001029883.3	c.1541del	p.(Pro514HisfsTer27)	Frameshift	Pathogenic	M	32	Unknown
13634942	1121	PCARE	NM_001029883.3	c.1541del	p.(Pro514HisfsTer27)	Frameshift	Pathogenic	M	32	Unknown

10572771	1122	PCARE	NM_001029883.3	c.1949G>A	p.(Trp650Ter)	Stopgain	Pathogenic	M	37	Any other ethnic group
10572771	1122	PCARE	NM_001029883.3	c.1949G>A	p.(Trp650Ter)	Stopgain	Pathogenic	M	37	Any other ethnic group
15452380	1122	PCARE	NM_001029883.3	c.1949G>A	p.(Trp650Ter)	Stopgain	Pathogenic	M	41	Any other ethnic group
15452380	1122	PCARE	NM_001029883.3	c.1949G>A	p.(Trp650Ter)	Stopgain	Pathogenic	M	41	Any other ethnic group
16503920	1123	PCARE	NM_001029883.3	c.3002G>A	p.(Trp1001Ter)	Stopgain	Pathogenic	F	40	Any other ethnic group
16503920	1123	PCARE	NM_001029883.3	c.920T>A	p.(Leu307Ter)	Stopgain	Pathogenic	F	40	Any other ethnic group
17017153	1124	PCARE	NM_001029883.3	c.3149dup	p.(Pro1051ThrfsTer56)	Frameshift	Pathogenic	M	69	Any other ethnic group
17017153	1124	PCARE	NM_001029883.3	c.3149dup	p.(Pro1051ThrfsTer56)	Frameshift	Pathogenic	M	69	Any other ethnic group
12949068	1125	PCARE	NM_001029883.3	c.2906_2915dup	p.(Ser973LysfsTer137)	Frameshift	Pathogenic	M	55	Not stated
12949068	1125	PCARE	NM_001029883.3	c.2966del	p.(Pro989LeusfsTer46)	Frameshift	Pathogenic	M	55	Not stated
17744061	1126	PCARE	NM_001029883.3	c.3461del	p.(Gly1154AlafsTer18)	Frameshift	Likely Pathogenic	M	32	Any other ethnic group
17744061	1126	PCARE	NM_001029883.3	c.3461del	p.(Gly1154AlafsTer18)	Frameshift	Likely Pathogenic	M	32	Any other ethnic group
18474308	1127	PCARE	NM_001029883.3	c.682_684del	p.(Glu228del)	Inframe indel	Variant of Uncertain Significance	M	25	Not stated
18474308	1127	PCARE	NM_001029883.3	c.682_684del	p.(Glu228del)	Inframe indel	Variant of Uncertain Significance	M	25	Not stated
10622128	1128	CABP4	NM_145200.5	Partial gene deletion	Partial gene deletion	Partial gene deletion	Likely Pathogenic	F	49	Unknown
10622128	1128	CABP4	NM_145200.5	Partial gene deletion	Partial gene deletion	Partial gene deletion	Likely Pathogenic	F	49	Unknown
17687263	1128	CABP4	NM_145200.5	Partial gene deletion	Partial gene deletion	Partial gene deletion	Likely Pathogenic	F	37	Not stated
17687263	1128	CABP4	NM_145200.5	Partial gene deletion	Partial gene deletion	Partial gene deletion	Likely Pathogenic	F	37	Not stated
11764227	1129	CABP4	NM_145200.5	c.673C>T	p.(Arg225Ter)	Stopgain	Pathogenic	F	26	Asian - Pakistani
11764227	1129	CABP4	NM_145200.5	c.673C>T	p.(Arg225Ter)	Stopgain	Pathogenic	F	26	Asian - Pakistani
13614327	1130	CABP4	NM_145200.5	c.673C>T	p.(Arg225Ter)	Stopgain	Pathogenic	F	48	Unknown
13614327	1130	CABP4	NM_145200.5	c.673C>T	p.(Arg225Ter)	Stopgain	Pathogenic	F	48	Unknown
13800723	1131	CABP4	NM_145200.5	c.61_62delinsA	p.(Pro21ThrfsTer6)	Frameshift	Pathogenic	M	10	Any other ethnic group
13800723	1131	CABP4	NM_145200.5	c.61_62delinsA	p.(Pro21ThrfsTer6)	Frameshift	Pathogenic	M	10	Any other ethnic group
4738523	1132	CACNA1F	NM_005183.4	c.1684+2T>C	Splice	Splice	Pathogenic	M	62	White - British
4956986	1133	CACNA1F	NM_005183.4	c.3269+1G>A	Splice	Splice	Pathogenic	M	29	White - British
4750003	1133	CACNA1F	NM_005183.4	c.3269+1G>A	Splice	Splice	Pathogenic	M	33	White - British
5301162	1134	CACNA1F	NM_005183.4	c.1218del	p.(Trp407GlyfsTer37)	Frameshift	Pathogenic	M	27	White - British
4876353	1134	CACNA1F	NM_005183.4	c.1218del	p.(Trp407GlyfsTer37)	Frameshift	Pathogenic	M	30	White - British
6445095	1135	CACNA1F	NM_005183.4	c.952_954del	p.(Phe318del)	Inframe indel	Pathogenic	M	27	White - British
9909479	1136	CACNA1F	NM_005183.4	c.784C>T	p.(Arg262Ter)	Stopgain	Pathogenic	M	31	White - British
11044865	1137	CACNA1F	NM_005183.4	c.1538_1542del	p.(Arg513GlnfsTer35)	Frameshift	Pathogenic	M	14	Not stated
9261202	1138	CACNA1F	NM_005183.4	c.3628C>T	p.(Gln1210Ter)	Stopgain	Pathogenic	M	19	White - Other
10908820	1139	CACNA1F	NM_005183.4	c.1218del	p.(Trp407GlyfsTer37)	Frameshift	Pathogenic	M	29	White - British
17273038	1140	CACNA1F	NM_005183.4	c.2264C>A	p.(Ala755Asp)	Missense	Likely Pathogenic	M	5	White - Other
10717314	1141	CACNA1F	NM_005183.4	c.3633_3637delinsA	p.(His1211GlnfsTer55)	Frameshift	Likely Pathogenic	M	17	Black - African
12596030	1142	CACNA1F	NM_005183.4	c.3213T>G	p.(Asn1071Lys)	Missense	Likely Pathogenic	M	34	Not stated
11578377	1143	CACNA1F	NM_005183.4	c.1466_1496+7del	p.(Gly490ThrfsTer4)	Frameshift	Pathogenic	M	17	Black - African
12920802	1144	CACNA1F	NM_005183.4	c.2905C>T	p.(Arg969Ter)	Stopgain	Pathogenic	M	33	Not stated
12178753	1145	CACNA1F	NM_005183.4	c.3341_3342del	p.(Ser1114CysfsTer38)	Frameshift	Pathogenic	M	15	Asian - Other
13611079	1146	CACNA1F	NM_005183.4	c.1873C>T	p.(Arg625Ter)	Stopgain	Pathogenic	M	19	Not stated
9065055	1147	CACNA1F	NM_005183.4	c.1910+1G>T	Splice	Splice	Pathogenic	M	27	Not stated
12996731	1148	CACNA1F	NM_005183.4	c.3001G>T	p.(Gly1001Ter)	Stopgain	Likely Pathogenic	M	12	Unknown
12564439	1149	CACNA1F	NM_005183.4	c.244C>T	p.(Arg82Ter)	Stopgain	Pathogenic	M	17	Not stated
9536302	1150	CACNA1F	NM_005183.4	c.4723+1G>T	Splice	Splice	Variant of Uncertain Significance	M	19	Asian - Other
15751196	1151	CACNA1F	NM_005183.4	c.5416C>T	p.(Gln1806Ter)	Stopgain	Likely Pathogenic	M	10	Not stated
13590359	1152	CACNA1F	NM_005183.4	c.3121_3122+2del	Splice	Splice	Likely Pathogenic	M	26	ed - White and Black Caribbean
15279382	1153	CACNA1F	NM_005183.4	c.2387_2388del	p.(Glu796GlyfsTer51)	Frameshift	Likely Pathogenic	M	11	White - British
16141943	1154	CACNA1F	NM_005183.4	c.3887del	p.(Arg1296ProfsTer41)	Frameshift	Likely Pathogenic	M	13	Not stated
16108777	1155	CACNA1F	NM_005183.4	c.1537C>T	p.(Arg513Ter)	Stopgain	Pathogenic	M	12	Not stated
14718535	1156	CACNA1F	NM_005183.4	c.1218del	p.(Trp407GlyfsTer37)	Frameshift	Pathogenic	M	14	White - Other

9256428	1157	CACNA1F	NM_005183.4	c.4472C>T	p.(Pro1491Leu)	Missense	Likely Pathogenic	M	74	Not stated
16397219	1158	CACNA1F	NM_005183.4	c.4084C>T	p.(Arg1362Ter)	Stopgain	Pathogenic	M	14	Not stated
10775141	1159	CACNA1F	NM_005183.4	c.4258T>G	p.(Tyr1420Arg)	Missense	Variant of Uncertain Significance	M	16	White - British
16845219	1160	CACNA1F	NM_005183.4	c.381+1G>A	Splice	Splice	Pathogenic	M	12	Not stated
15486890	1161	CACNA1F	NM_005183.4	c.784C>T	p.(Arg262Ter)	Stopgain	Pathogenic	M	15	Any other ethnic group
17354595	1162	CACNA1F	NM_005183.4	c.3269+1G>A	Splice	Splice	Pathogenic	M	10	Not stated
18206404	1163	CACNA1F	NM_005183.4	c.2767-1G>T	Splice	Splice	Likely Pathogenic	M	4	Not stated
13078183	1164	CACNA1F	NM_005183.4	c.2071C>T	p.(Arg691Ter)	Stopgain	Pathogenic	M	14	White - British
17861815	1165	CACNA1F	NM_005183.4	c.4424G>A	p.(Gly1475Asp)	Missense	Variant of Uncertain Significance	M	5	Black - African
18350030	1166	CACNA1F	NM_005183.4	c.3069+2T>C	Splice	Splice	Likely Pathogenic	M	11	Not stated
9131429	1167	CACNA1F	NM_005183.4	c.4147G>A	p.(Glu1383Lys)	Missense	Likely Pathogenic	M	59	White - British
8191014	1168	CACNA2D4	NM_172364.5	c.1882C>T	p.(Arg628Ter)	Stopgain	Variant of Uncertain Significance	F	41	Asian - Indian
8191014	1168	CACNA2D4	NM_172364.5	c.1882C>T	p.(Arg628Ter)	Stopgain	Variant of Uncertain Significance	F	41	Asian - Indian
5125413	1169	CDH23	NM_022124.6	c.193del	p.(Leu65TrpfsTer49)	Frameshift	Pathogenic	F	69	Not stated
5125413	1169	CDH23	NM_022124.6	c.7908C>G	p.(Tyr2636Ter)	Stopgain	Pathogenic	F	69	Not stated
13698971	1170	CDH23	NM_022124.6	c.6254-3_6254delinsT	Splice	Splice	Pathogenic	F	47	Not stated
13698971	1170	CDH23	NM_022124.6	c.6393del	p.(Ile2132SerfsTer11)	Frameshift	Pathogenic	F	47	Not stated
2223724	1171	CDH23	NM_022124.6	c.2177-2A>G	Splice	Splice	Pathogenic	M	45	Not stated
2223724	1171	CDH23	NM_022124.6	c.9122T>C	p.(Leu3041Pro)	Missense	Variant of Uncertain Significance	M	45	Not stated
5196897	1172	CDH23	NM_022124.6	c.3337G>C	p.(Glu1113Gln)	Missense	Variant of Uncertain Significance	F	36	Asian - Indian
5196897	1172	CDH23	NM_022124.6	c.8722G>A	p.(Gly2908Arg)	Missense	Variant of Uncertain Significance	F	36	Asian - Indian
8956275	1173	CDH23	NM_022124.6	c.6254-3_6254delinsT	Splice	Splice	Pathogenic	F	49	Not stated
8956275	1173	CDH23	NM_022124.6	c.7362G>A	p.(Thr2454Thr)	Synonymous	Likely Pathogenic	F	49	Not stated
1062242	1174	CDH23	NM_022124.6	c.6319C>T	p.(Arg2107Ter)	Stopgain	Pathogenic	M	53	Not stated
1062242	1174	CDH23	NM_022124.6	c.7305dup	p.(Leu2436ThrfsTer3)	Frameshift	Likely Pathogenic	M	53	Not stated
8549141	1175	CDH23	NM_022124.6	c.1369C>T	p.(Arg457Trp)	Missense	Variant of Uncertain Significance	F	46	White - British
8549141	1175	CDH23	NM_022124.6	c.5237G>A	p.(Arg1746Gln)	Missense	Likely Pathogenic	F	46	White - British
11772655	1176	CDH23	NM_022124.6	c.6319C>T	p.(Arg2107Ter)	Stopgain	Pathogenic	F	31	Asian - Pakistani
11772655	1176	CDH23	NM_022124.6	c.6319C>T	p.(Arg2107Ter)	Stopgain	Pathogenic	F	31	Asian - Pakistani
13488257	1177	CDH23	NM_022124.6	c.1986+3A>T	Splice	Splice	Variant of Uncertain Significance	F	18	Not stated
13488257	1177	CDH23	NM_022124.6	c.1986+3A>T	Splice	Splice	Variant of Uncertain Significance	F	18	Not stated
12427554	1178	CDH23	NM_022124.6	c.1411G>A	p.(Glu471Lys)	Missense	Likely Pathogenic	F	17	Unknown
12427554	1178	CDH23	NM_022124.6	c.4759_4766del	p.(Thr158CysfsTer4)	Frameshift	Pathogenic	F	17	Unknown
11035310	1179	CDH23	NM_022124.6	c.4246C>T	p.(Pro1416Ser)	Missense	Variant of Uncertain Significance	M	16	Asian - Pakistani
11035310	1179	CDH23	NM_022124.6	c.4246C>T	p.(Pro1416Ser)	Missense	Variant of Uncertain Significance	M	16	Asian - Pakistani
12793248	1180	CDH23	NM_022124.6	c.5237G>A	p.(Arg1746Gln)	Missense	Likely Pathogenic	M	28	White - British
12793248	1180	CDH23	NM_022124.6	c.9278+2T>G	Splice	Splice	Pathogenic	M	28	White - British
15176979	1181	CDH23	NM_022124.6	c.1986+3A>T	Splice	Splice	Variant of Uncertain Significance	M	21	White - Other
15176979	1181	CDH23	NM_022124.6	c.1986+3A>T	Splice	Splice	Variant of Uncertain Significance	M	21	White - Other
15775164	1182	CDH23	NM_022124.6	c.6050-9G>A	Splice	Splice	Pathogenic	F	16	Asian - Pakistani
15775164	1182	CDH23	NM_022124.6	c.6050-9G>A	Splice	Splice	Pathogenic	F	16	Asian - Pakistani
6627613	1183	CDH23	NM_022124.6	c.7823G>A	p.(Arg2608His)	Missense	Variant of Uncertain Significance	M	37	Not stated
6627613	1183	CDH23	NM_022124.6	c.9122T>C	p.(Leu3041Pro)	Missense	Variant of Uncertain Significance	M	37	Not stated
17701242	1184	CDH23	NM_022124.6	Exon 4-6 deletion	Deletion	Deletion	Likely Pathogenic	F	50	Not stated
17701242	1184	CDH23	NM_022124.6	Exon 4-6 deletion	Deletion	Deletion	Likely Pathogenic	F	50	Not stated
17688712	1185	CDH23	NM_022124.6	c.2398-1G>T	Splice	Splice	Pathogenic	F	49	Not stated
17688712	1185	CDH23	NM_022124.6	c.7908C>G	p.(Tyr2636Ter)	Stopgain	Pathogenic	F	49	Not stated
17887890	1186	CDH23	NM_022124.6	c.6900C>G	p.(Tyr2300Ter)	Stopgain	Likely Pathogenic	F	74	Not stated
17887890	1186	CDH23	NM_022124.6	c.9077+1G>T	Splice	Splice	Likely Pathogenic	F	74	Not stated
18286365	1187	CDH23	NM_022124.6	Exon 67-69 deletion	Deletion	Deletion	Likely Pathogenic	M	20	Not stated
18286365	1187	CDH23	NM_022124.6	Exon 67-69 deletion	Deletion	Deletion	Likely Pathogenic	M	20	Not stated

4389454	1188	CDH3	NM_001793.6	c.830del	p.(Gly277AlafsTer20)	Frameshift	Pathogenic	M	39	Not stated
4389454	1188	CDH3	NM_001793.6	c.830del	p.(Gly277AlafsTer20)	Frameshift	Pathogenic	M	39	Not stated
12399281	1189	CDH3	NM_001793.6	c.2357del	p.(Gly786AlafsTer7)	Frameshift	Variant of Uncertain Significance	M	44	Asian - Pakistani
12399281	1189	CDH3	NM_001793.6	c.2357del	p.(Gly786AlafsTer7)	Frameshift	Variant of Uncertain Significance	M	44	Asian - Pakistani
13884632	1190	CDH3	NM_001793.6	c.325T>G	p.(Trp109Gly)	Missense	Variant of Uncertain Significance	M	38	Asian - Other
13884632	1190	CDH3	NM_001793.6	c.325T>G	p.(Trp109Gly)	Missense	Variant of Uncertain Significance	M	38	Asian - Other
5484632	1191	CDHR1	NM_033100.4	c.1448A>G	p.(Glu483Gly)	Missense	Likely Pathogenic	F	58	Not stated
5484632	1191	CDHR1	NM_033100.4	c.1448A>G	p.(Glu483Gly)	Missense	Likely Pathogenic	F	58	Not stated
4324515	1192	CDHR1	NM_033100.4	c.562G>A	p.(Gly188Ser)	Missense	Likely Pathogenic	F	77	White - British
4324515	1192	CDHR1	NM_033100.4	c.783G>A	p.(Pro261Pro)	Synonymous	Likely Pathogenic	F	77	White - British
9931319	1193	CDHR1	NM_033100.4	c.1463del	p.(Gly488AlafsTer20)	Frameshift	Pathogenic	F	41	Asian - Indian
9931319	1193	CDHR1	NM_033100.4	c.1463del	p.(Gly488AlafsTer20)	Frameshift	Pathogenic	F	41	Asian - Indian
10427150	1194	CDHR1	NM_033100.4	c.2522_2528del	p.(Ile841SerfsTer119)	Frameshift	Pathogenic	M	51	White - Other
10427150	1194	CDHR1	NM_033100.4	c.2522_2528del	p.(Ile841SerfsTer119)	Frameshift	Pathogenic	M	51	White - Other
4080082	1195	CDHR1	NM_033100.4	c.1463del	p.(Gly488AlafsTer20)	Frameshift	Pathogenic	F	35	Not stated
4080082	1195	CDHR1	NM_033100.4	c.1463del	p.(Gly488AlafsTer20)	Frameshift	Pathogenic	F	35	Not stated
10542552	1195	CDHR1	NM_033100.4	c.1463del	p.(Gly488AlafsTer20)	Frameshift	Pathogenic	M	32	Any other ethnic group
10542552	1195	CDHR1	NM_033100.4	c.1463del	p.(Gly488AlafsTer20)	Frameshift	Pathogenic	M	32	Any other ethnic group
10851609	1196	CDHR1	NM_033100.4	c.1527T>G	p.(Tyr509Ter)	Stopgain	Pathogenic	M	38	Any other ethnic group
10851609	1196	CDHR1	NM_033100.4	c.2522_2528del	p.(Ile841SerfsTer119)	Frameshift	Pathogenic	M	38	Any other ethnic group
11138441	1197	CDHR1	NM_033100.4	c.2522_2528del	p.(Ile841SerfsTer119)	Frameshift	Pathogenic	M	43	White - Other
11138441	1197	CDHR1	NM_033100.4	c.2522_2528del	p.(Ile841SerfsTer119)	Frameshift	Pathogenic	M	43	White - Other
12674318	1198	CDHR1	NM_033100.4	c.2522_2528del	p.(Ile841SerfsTer119)	Frameshift	Pathogenic	F	52	Unknown
12674318	1198	CDHR1	NM_033100.4	c.2522_2528del	p.(Ile841SerfsTer119)	Frameshift	Pathogenic	F	52	Unknown
17220293	1198	CDHR1	NM_033100.4	c.2522_2528del	p.(Ile841SerfsTer119)	Frameshift	Pathogenic	M	27	Any other ethnic group
17220293	1198	CDHR1	NM_033100.4	c.2522_2528del	p.(Ile841SerfsTer119)	Frameshift	Pathogenic	M	27	Any other ethnic group
13102004	1199	CDHR1	NM_033100.4	c.1A>G	p.(Met1?)	Start codon	Likely Pathogenic	M	82	Not stated
13102004	1199	CDHR1	NM_033100.4	c.783G>A	p.(Pro261Pro)	Synonymous	Likely Pathogenic	M	82	Not stated
13564221	1200	CDHR1	NM_033100.4	c.2522_2528del	p.(Ile841SerfsTer119)	Frameshift	Pathogenic	F	35	Not stated
13564221	1200	CDHR1	NM_033100.4	c.2522_2528del	p.(Ile841SerfsTer119)	Frameshift	Pathogenic	F	35	Not stated
7073142	1201	CDHR1	NM_033100.4	c.783G>A	p.(Pro261Pro)	Synonymous	Likely Pathogenic	F	50	White - British
7073142	1201	CDHR1	NM_033100.4	c.783G>A	p.(Pro261Pro)	Synonymous	Likely Pathogenic	F	50	White - British
16814146	1202	CDHR1	NM_033100.4	c.2522_2528del	p.(Ile841SerfsTer119)	Frameshift	Pathogenic	M	40	White - Other
16814146	1202	CDHR1	NM_033100.4	c.2522_2528del	p.(Ile841SerfsTer119)	Frameshift	Pathogenic	M	40	White - Other
17886784	1203	CDHR1	NM_033100.4	c.1527T>G	p.(Tyr509Ter)	Stopgain	Pathogenic	M	42	Not stated
17886784	1203	CDHR1	NM_033100.4	c.2522_2528del	p.(Ile841SerfsTer119)	Frameshift	Pathogenic	M	42	Not stated
18002991	1204	CDHR1	NM_033100.4	c.1367G>A	p.(Ala456Glu)	Missense	Variant of Uncertain Significance	F	30	Mixed - White and Asian
18002991	1204	CDHR1	NM_033100.4	c.1485+2T>G	Splice	Splice	Pathogenic	F	30	Mixed - White and Asian
1800735	1205	CEP290	NM_025114.4	c.148C>T	p.(His50Tyr)	Missense	Likely Pathogenic	M	37	Any other ethnic group
1800735	1205	CEP290	NM_025114.4	c.4393C>T	p.(Arg1465Ter)	Stopgain	Pathogenic	M	37	Any other ethnic group
1554776	1205	CEP290	NM_025114.4	c.148C>T	p.(His50Tyr)	Missense	Likely Pathogenic	F	38	Not stated
1554776	1205	CEP290	NM_025114.4	c.4393C>T	p.(Arg1465Ter)	Stopgain	Pathogenic	F	38	Not stated
293831	1205	CEP290	NM_025114.4	c.148C>T	p.(His50Tyr)	Missense	Likely Pathogenic	M	42	Asian - Pakistani
293831	1205	CEP290	NM_025114.4	c.4393C>T	p.(Arg1465Ter)	Stopgain	Pathogenic	M	42	Asian - Pakistani
1865128	1206	CEP290	NM_025114.4	c.2991+1655A>G	Splice	Splice	Likely Pathogenic	F	37	Not stated
1865128	1206	CEP290	NM_025114.4	c.4966G>T	p.(Glu1656Ter)	Stopgain	Pathogenic	F	37	Not stated
381534	1207	CEP290	NM_025114.4	c.2991+1655A>G	Splice	Splice	Likely Pathogenic	M	50	Not stated
381534	1207	CEP290	NM_025114.4	c.2991+1655A>G	Splice	Splice	Likely Pathogenic	M	50	Not stated
1240378	1208	CEP290	NM_025114.4	c.2991+1655A>G	Splice	Splice	Likely Pathogenic	F	39	White - British
1240378	1208	CEP290	NM_025114.4	c.5254C>T	p.(Arg1752Trp)	Missense	Likely Pathogenic	F	39	White - British
6841288	1209	CEP290	NM_025114.4	c.2991+1655A>G	Splice	Splice	Likely Pathogenic	M	24	White - British

6841288	1209	CEP290	NM_025114.4	c.4438-3del	Splice	Splice	Likely Pathogenic	M	24	White - British
12013868	1210	CEP290	NM_025114.4	c.4966_4967del	p.(Glu1656AsnfsTer3)	Frameshift	Pathogenic	M	55	White - Other
12013868	1210	CEP290	NM_025114.4	c.5777G>C	p.(Arg1926Pro)	Missense	Likely Pathogenic	M	55	White - Other
5216546	1210	CEP290	NM_025114.4	c.4966_4967del	p.(Glu1656AsnfsTer3)	Frameshift	Pathogenic	F	57	Any other ethnic group
5216546	1210	CEP290	NM_025114.4	c.5777G>C	p.(Arg1926Pro)	Missense	Likely Pathogenic	F	57	Any other ethnic group
8529261	1211	CEP290	NM_025114.4	c.1984C>T	p.(Gln662Ter)	Stopgain	Pathogenic	M	19	Not stated
8529261	1211	CEP290	NM_025114.4	c.2991+1655A>G	Splice	Splice	Likely Pathogenic	M	19	Not stated
7279334	1212	CEP290	NM_025114.4	c.223A>G	p.(Lys75Glu)	Missense	Likely Pathogenic	M	25	White - British
7279334	1212	CEP290	NM_025114.4	c.2991+1655A>G	Splice	Splice	Likely Pathogenic	M	25	White - British
9986920	1212	CEP290	NM_025114.4	c.223A>G	p.(Lys75Glu)	Missense	Likely Pathogenic	F	31	Unknown
9986920	1212	CEP290	NM_025114.4	c.2991+1655A>G	Splice	Splice	Likely Pathogenic	F	31	Unknown
367856	1213	CEP290	NM_025114.4	c.1163T>A	p.(Leu388Ter)	Stopgain	Likely Pathogenic	F	42	White - British
367856	1213	CEP290	NM_025114.4	c.2991+1655A>G	Splice	Splice	Likely Pathogenic	F	42	White - British
8278808	1214	CEP290	NM_025114.4	c.21G>T	p.(Trp7Cys)	Missense	Likely Pathogenic	F	20	Not stated
8278808	1214	CEP290	NM_025114.4	c.21G>T	p.(Trp7Cys)	Missense	Likely Pathogenic	F	20	Not stated
10017783	1215	CEP290	NM_025114.4	c.2991+1655A>G	Splice	Splice	Likely Pathogenic	M	16	Not stated
10017783	1215	CEP290	NM_025114.4	c.6277del	p.(Val2093SerfsTer4)	Frameshift	Pathogenic	M	16	Not stated
8087736	1216	CEP290	NM_025114.4	c.5932C>T	p.(Arg1978Ter)	Stopgain	Pathogenic	F	40	Mixed - White and Asian
8087736	1216	CEP290	NM_025114.4	c.5941G>T	p.(Glu1981Ter)	Stopgain	Pathogenic	F	40	Mixed - White and Asian
11026301	1217	CEP290	NM_025114.4	c.1066-1G>A	Splice	Splice	Pathogenic	M	14	White - British
11026301	1217	CEP290	NM_025114.4	c.2991+1655A>G	Splice	Splice	Likely Pathogenic	M	14	White - British
4637758	1218	CEP290	NM_025114.4	c.2444T>G	p.(Ile815Ser)	Missense	Variant of Uncertain Significance	F	36	Any other ethnic group
4637758	1218	CEP290	NM_025114.4	c.2444T>G	p.(Ile815Ser)	Missense	Variant of Uncertain Significance	F	36	Any other ethnic group
11465383	1219	CEP290	NM_025114.4	c.2991+1655A>G	Splice	Splice	Likely Pathogenic	M	14	Unknown
11465383	1219	CEP290	NM_025114.4	c.4966G>T	p.(Glu1656Ter)	Stopgain	Pathogenic	M	14	Unknown
12058339	1220	CEP290	NM_025114.4	c.2991+1655A>G	Splice	Splice	Likely Pathogenic	M	12	Not stated
12058339	1220	CEP290	NM_025114.4	c.4801C>T	p.(Gln1601Ter)	Stopgain	Pathogenic	M	12	Not stated
8253839	1221	CEP290	NM_025114.4	c.6869dup	p.(Asn2290LysfsTer6)	Frameshift	Pathogenic	M	57	Asian - Pakistani
8253839	1221	CEP290	NM_025114.4	c.96_97del	p.(Leu32PhefsTer7)	Frameshift	Likely Pathogenic	M	57	Asian - Pakistani
11800081	1222	CEP290	NM_025114.4	c.2991+1655A>G	Splice	Splice	Likely Pathogenic	M	25	White - British
11800081	1222	CEP290	NM_025114.4	c.7048C>T	p.(Gln2350Ter)	Stopgain	Pathogenic	M	25	White - British
16299961	1223	CEP290	NM_025114.4	c.4661_4663del	p.(Glu1554del)	Inframe indel	Likely Pathogenic	M	18	Not stated
16299961	1223	CEP290	NM_025114.4	c.4661_4663del	p.(Glu1554del)	Inframe indel	Likely Pathogenic	M	18	Not stated
16088449	1224	CEP290	NM_025114.4	c.270_274del	p.(Lys90AsnfsTer6)	Frameshift	Likely Pathogenic	M	7	Black - Other
16088449	1224	CEP290	NM_025114.4	c.2991+1655A>G	Splice	Splice	Likely Pathogenic	M	7	Black - Other
16907344	1225	CEP290	NM_025114.4	c.6869dup	p.(Asn2290LysfsTer6)	Frameshift	Pathogenic	F	18	Unknown
16907344	1225	CEP290	NM_025114.4	Exon 39-41 deletion	Deletion	Deletion	Likely Pathogenic	F	18	Unknown
16926825	1225	CEP290	NM_025114.4	c.6869dup	p.(Asn2290LysfsTer6)	Frameshift	Pathogenic	M	24	Unknown
16926825	1225	CEP290	NM_025114.4	Exon 39-41 deletion	Deletion	Deletion	Likely Pathogenic	M	24	Unknown
15613660	1226	CEP290	NM_025114.4	c.1514_1515del	p.(Glu505AlafsTer5)	Frameshift	Pathogenic	F	33	Any other ethnic group
15613660	1226	CEP290	NM_025114.4	c.5587-1G>C	Splice	Splice	Pathogenic	F	33	Any other ethnic group
17380971	1227	CEP290	NM_025114.4	c.1666dup	p.(Ile556AsnfsTer20)	Frameshift	Pathogenic	F	11	Not stated
17380971	1227	CEP290	NM_025114.4	c.6788del	p.(Ser2263IlefsTer17)	Frameshift	Likely Pathogenic	F	11	Not stated
18187623	1228	CEP290	NM_025114.4	c.1066-1G>A	Splice	Splice	Pathogenic	M	6	Not stated
18187623	1228	CEP290	NM_025114.4	c.5434_5435del	p.(Glu1812LysfsTer5)	Frameshift	Pathogenic	M	6	Not stated
16818591	1229	CEP290	NM_025114.4	c.1623+1G>A	Splice	Splice	Pathogenic	F	6	Not stated
16818591	1229	CEP290	NM_025114.4	c.2991+1655A>G	Splice	Splice	Likely Pathogenic	F	6	Not stated
18541767	1230	CEP290	NM_025114.4	c.2632dup	p.(Ile878AsnfsTer6)	Frameshift	Likely Pathogenic	F	50	White - British
18541767	1230	CEP290	NM_025114.4	c.3788T>G	p.(Leu1263Arg)	Missense	Variant of Uncertain Significance	F	50	White - British
5421457	1231	CEP78	NM_001098802.3	Deletion-Inversion	Deletion-Inversion	Deletion-Inversion	Likely Pathogenic	M	72	Not stated
5421457	1231	CEP78	NM_001098802.3	Deletion-Inversion	Deletion-Inversion	Deletion-Inversion	Likely Pathogenic	M	72	Not stated

1151072	1232	CERKL	NM_001030311.3	c.847C>T	p.(Arg283Ter)	Stopgain	Pathogenic	M	77	Asian - Pakistani
1151072	1232	CERKL	NM_001030311.3	c.847C>T	p.(Arg283Ter)	Stopgain	Pathogenic	M	77	Asian - Pakistani
2758846	1233	CERKL	NM_001030311.3	c.847C>T	p.(Arg283Ter)	Stopgain	Pathogenic	M	57	Asian - Indian
2758846	1233	CERKL	NM_001030311.3	c.847C>T	p.(Arg283Ter)	Stopgain	Pathogenic	M	57	Asian - Indian
5149990	1234	CERKL	NM_001030311.3	c.1238-1G>A	Splice	Splice	Likely Pathogenic	M	41	White - British
5149990	1234	CERKL	NM_001030311.3	c.941_942del	p.(His314ArgfsTer55)	Frameshift	Likely Pathogenic	M	41	White - British
4733840	1234	CERKL	NM_001030311.3	c.1238-1G>A	Splice	Splice	Likely Pathogenic	M	50	Not stated
4733840	1234	CERKL	NM_001030311.3	c.941_942del	p.(His314ArgfsTer55)	Frameshift	Likely Pathogenic	M	50	Not stated
5355020	1235	CERKL	NM_001030311.3	c.847C>T	p.(Arg283Ter)	Stopgain	Pathogenic	F	63	Not stated
5355020	1235	CERKL	NM_001030311.3	c.847C>T	p.(Arg283Ter)	Stopgain	Pathogenic	F	63	Not stated
153152	1236	CERKL	NM_001030311.3	c.1347-3C>G	Splice	Splice	Likely Pathogenic	F	80	Not stated
153152	1236	CERKL	NM_001030311.3	c.847C>T	p.(Arg283Ter)	Stopgain	Pathogenic	F	80	Not stated
10295592	1237	CERKL	NM_001030311.3	c.1151+3_1151+6del	Splice	Splice	Pathogenic	F	38	Asian - Pakistani
10295592	1237	CERKL	NM_001030311.3	c.1151+3_1151+6del	Splice	Splice	Pathogenic	F	38	Asian - Pakistani
6791399	1237	CERKL	NM_001030311.3	c.1151+3_1151+6del	Splice	Splice	Pathogenic	F	41	Any other ethnic group
6791399	1237	CERKL	NM_001030311.3	c.1151+3_1151+6del	Splice	Splice	Pathogenic	F	41	Any other ethnic group
7777139	1238	CERKL	NM_001030311.3	c.316C>A	p.(Arg106Ser)	Missense	Pathogenic	M	38	Not stated
7777139	1238	CERKL	NM_001030311.3	c.316C>A	p.(Arg106Ser)	Missense	Pathogenic	M	38	Not stated
8835280	1239	CERKL	NM_001030311.3	c.1090C>T	p.(Arg364Ter)	Stopgain	Pathogenic	M	33	Asian - Other
8835280	1239	CERKL	NM_001030311.3	c.1090C>T	p.(Arg364Ter)	Stopgain	Pathogenic	M	33	Asian - Other
7592976	1240	CERKL	NM_001030311.3	c.847C>T	p.(Arg283Ter)	Stopgain	Pathogenic	M	45	White - Irish
7592976	1240	CERKL	NM_001030311.3	c.847C>T	p.(Arg283Ter)	Stopgain	Pathogenic	M	45	White - Irish
7128708	1241	CERKL	NM_001030311.3	c.847C>T	p.(Arg283Ter)	Stopgain	Pathogenic	F	43	Asian - Indian
7128708	1241	CERKL	NM_001030311.3	c.847C>T	p.(Arg283Ter)	Stopgain	Pathogenic	F	43	Asian - Indian
10622758	1242	CERKL	NM_001030311.3	c.238+2T>C	Splice	Splice	Likely Pathogenic	M	29	Not stated
10622758	1242	CERKL	NM_001030311.3	Exon 1 deletion	Deletion	Deletion	Likely Pathogenic	M	29	Not stated
10742710	1243	CERKL	NM_001030311.3	c.847C>T	p.(Arg283Ter)	Stopgain	Pathogenic	F	50	Asian - Pakistani
10742710	1243	CERKL	NM_001030311.3	c.847C>T	p.(Arg283Ter)	Stopgain	Pathogenic	F	50	Asian - Pakistani
3156117	1244	CERKL	NM_001030311.3	c.316C>A	p.(Arg106Ser)	Missense	Pathogenic	M	72	Asian - Indian
3156117	1244	CERKL	NM_001030311.3	c.316C>T	p.(Arg106Cys)	Missense	Likely Pathogenic	M	72	Asian - Indian
10448220	1245	CERKL	NM_001030311.3	c.847C>T	p.(Arg283Ter)	Stopgain	Pathogenic	F	38	White - British
10448220	1245	CERKL	NM_001030311.3	c.847C>T	p.(Arg283Ter)	Stopgain	Pathogenic	F	38	White - British
12398035	1246	CERKL	NM_001030311.3	c.316C>A	p.(Arg106Ser)	Missense	Pathogenic	M	59	Not stated
12398035	1246	CERKL	NM_001030311.3	c.316C>A	p.(Arg106Ser)	Missense	Pathogenic	M	59	Not stated
15102016	1247	CERKL	NM_001030311.3	c.271G>T	p.(Glu91Ter)	Stopgain	Likely Pathogenic	F	47	White - Other
15102016	1247	CERKL	NM_001030311.3	c.271G>T	p.(Glu91Ter)	Stopgain	Likely Pathogenic	F	47	White - Other
16185014	1248	CERKL	NM_001030311.3	c.847C>T	p.(Arg283Ter)	Stopgain	Pathogenic	F	25	Not stated
16185014	1248	CERKL	NM_001030311.3	c.847C>T	p.(Arg283Ter)	Stopgain	Pathogenic	F	25	Not stated
16931753	1249	CERKL	NM_001030311.3	c.316C>A	p.(Arg106Ser)	Missense	Pathogenic	M	32	Any other ethnic group
16931753	1249	CERKL	NM_001030311.3	c.316C>A	p.(Arg106Ser)	Missense	Pathogenic	M	32	Any other ethnic group
17421599	1250	CERKL	NM_001030311.3	c.316C>A	p.(Arg106Ser)	Missense	Pathogenic	M	27	Not stated
17421599	1250	CERKL	NM_001030311.3	c.847C>T	p.(Arg283Ter)	Stopgain	Pathogenic	M	27	Not stated
8988405	1251	CERKL	NM_001030311.3	c.1090C>T	p.(Arg364Ter)	Stopgain	Pathogenic	F	65	Asian - Indian
8988405	1251	CERKL	NM_001030311.3	c.847C>T	p.(Arg283Ter)	Stopgain	Pathogenic	F	65	Asian - Indian
17276916	1252	CERKL	NM_001030311.3	c.1381C>T	p.(Arg461Ter)	Stopgain	Pathogenic	M	21	Not stated
17276916	1252	CERKL	NM_001030311.3	c.613+2T>C	Splice	Splice	Likely Pathogenic	M	21	Not stated
17561977	1253	CERKL	NM_001030311.3	c.316C>A	p.(Arg106Ser)	Missense	Pathogenic	M	23	Unknown
17561977	1253	CERKL	NM_001030311.3	c.316C>A	p.(Arg106Ser)	Missense	Pathogenic	M	23	Unknown
18402971	1254	CERKL	NM_001030311.3	c.847C>T	p.(Arg283Ter)	Stopgain	Pathogenic	M	33	Unknown
18402971	1254	CERKL	NM_001030311.3	c.847C>T	p.(Arg283Ter)	Stopgain	Pathogenic	M	33	Unknown
12195175	1255	CFH	NM_000186.4	c.1107G>A	p.(Trp369Ter)	Stopgain	Likely Pathogenic	M	76	White - British

2707179	1256	CHM	NM_000390.4	c.698C>G	p.(Ser233Ter)	Stopgain	Likely Pathogenic	M	56	White - British
9446093	1256	CHM	NM_000390.4	c.698C>G	p.(Ser233Ter)	Stopgain	Likely Pathogenic	M	75	Not stated
13250761	1256	CHM	NM_000390.4	c.698C>G	p.(Ser233Ter)	Stopgain	Likely Pathogenic	F	35	Any other ethnic group
566628	1257	CHM	NM_000390.4	Exon 9-15 deletion	Deletion	Deletion	Likely Pathogenic	M	58	Not stated
2631824	1258	CHM	NM_000390.4	c.808C>T	p.(Arg270Ter)	Stopgain	Pathogenic	M	73	Unknown
7654121	1259	CHM	NM_000390.4	c.652_655del	p.(Ser218LysfsTer13)	Frameshift	Pathogenic	M	66	Not stated
3694445	1260	CHM	NM_000390.4	c.-98C>T	Regulatory	Regulatory	Variant of Uncertain Significance	M	44	Not stated
3711119	1260	CHM	NM_000390.4	c.-98C>T	Regulatory	Regulatory	Variant of Uncertain Significance	M	58	Not stated
1288391	1261	CHM	NM_000390.4	c.877C>T	p.(Arg293Ter)	Stopgain	Pathogenic	F	74	Unknown
3271162	1262	CHM	NM_000390.4	c.49+3del	Splice	Splice	Variant of Uncertain Significance	M	62	Asian - Indian
10143209	1263	CHM	NM_000390.4	Exon 3-15 deletion	Deletion	Deletion	Likely Pathogenic	F	43	Not stated
18035184	1264	CHM	NM_000390.4	Exon 1-15 deletion	Deletion	Deletion	Likely Pathogenic	F	41	White - Irish
236004	1265	CHM	NM_000390.4	c.104T>C	p.(Leu35Pro)	Missense	Likely Pathogenic	M	55	Not stated
2142279	1266	CHM	NM_000390.4	Exon 10-11 deletion	Deletion	Deletion	Likely Pathogenic	M	60	White - British
725395	1267	CHM	NM_000390.4	c.49+2dup	Splice	Splice	Likely Pathogenic	M	74	White - British
658566	1268	CHM	NM_000390.4	c.529del	p.(Glu177LysfsTer20)	Frameshift	Likely Pathogenic	M	52	White - British
276926	1269	CHM	NM_000390.4	c.427del	p.(Asp143MetfsTer4)	Frameshift	Likely Pathogenic	M	80	Not stated
16250443	1270	CHM	NM_000390.4	c.1349+1del	Splice	Splice	Likely Pathogenic	F	62	Not stated
551543	1271	CHM	NM_000390.4	c.715C>T	p.(Arg239Ter)	Stopgain	Pathogenic	M	55	White - British
13450814	1272	CHM	NM_000390.4	Exon 1 deletion	Deletion	Deletion	Likely Pathogenic	M	80	Not stated
3232466	1273	CHM	NM_000390.4	c.535_538del	p.(Glu179ThrfsTer17)	Frameshift	Likely Pathogenic	M	66	Not stated
1372559	1274	CHM	NM_000390.4	c.1584_1587del	p.(Val529HisfsTer7)	Frameshift	Pathogenic	M	60	Unknown
9328255	1275	CHM	NM_000390.4	c.930_931insA	p.(Glu311ArgfsTer3)	Frameshift	Likely Pathogenic	F	80	Not stated
6626157	1276	CHM	NM_000390.4	c.877C>T	p.(Arg293Ter)	Stopgain	Pathogenic	M	60	Not stated
3253151	1277	CHM	NM_000390.4	c.189+1G>C	Splice	Splice	Pathogenic	M	59	Not stated
7376004	1278	CHM	NM_000390.4	Exon 1-15 deletion	Deletion	Deletion	Likely Pathogenic	M	26	White - British
2873282	1278	CHM	NM_000390.4	Exon 1-15 deletion	Deletion	Deletion	Likely Pathogenic	F	54	White - Irish
4085374	1279	CHM	NM_000390.4	c.116+1G>A	Splice	Splice	Pathogenic	M	51	White - British
2760666	1280	CHM	NM_000390.4	c.1520A>G	p.(His507Arg)	Missense	Variant of Uncertain Significance	M	79	White - Irish
13027881	1280	CHM	NM_000390.4	c.1520A>G	p.(His507Arg)	Missense	Variant of Uncertain Significance	F	48	Unknown
2760645	1280	CHM	NM_000390.4	c.1520A>G	p.(His507Arg)	Missense	Variant of Uncertain Significance	F	56	White - British
2618580	1281	CHM	NM_000390.4	c.315-1536A>G	Splice	Splice	Variant of Uncertain Significance	M	59	Any other ethnic group
9995124	1281	CHM	NM_000390.4	c.315-1536A>G	Splice	Splice	Variant of Uncertain Significance	M	32	White - British
4203548	1282	CHM	NM_000390.4	c.525_526del	p.(Glu177LysfsTer6)	Frameshift	Pathogenic	M	76	Not stated
4344626	1283	CHM	NM_000390.4	c.649_652del	p.(Tyr217HisfsTer14)	Frameshift	Pathogenic	M	53	White - British
4473615	1284	CHM	NM_000390.4	c.877C>T	p.(Arg293Ter)	Stopgain	Pathogenic	M	60	White - British
4924933	1285	CHM	NM_000390.4	c.649_652del	p.(Tyr217HisfsTer14)	Frameshift	Pathogenic	M	65	White - Irish
5179033	1286	CHM	NM_000390.4	c.126C>G	p.(Tyr42Ter)	Stopgain	Pathogenic	M	34	White - British
5354712	1287	CHM	NM_000390.4	Exon 1-15 deletion	Deletion	Deletion	Likely Pathogenic	M	40	Not stated
5450451	1288	CHM	NM_000390.4	Exon 1-15 deletion	Deletion	Deletion	Likely Pathogenic	M	39	Not stated
5423697	1289	CHM	NM_000390.4	c.116+1G>A	Splice	Splice	Pathogenic	F	77	Not stated
5525365	1290	CHM	NM_000390.4	c.1264C>T	p.(Gln422Ter)	Stopgain	Likely Pathogenic	M	49	Not stated
11037053	1290	CHM	NM_000390.4	c.1264C>T	p.(Gln422Ter)	Stopgain	Likely Pathogenic	F	69	Unknown
6361081	1291	CHM	NM_000390.4	c.877C>T	p.(Arg293Ter)	Stopgain	Pathogenic	M	49	Not stated
6285628	1292	CHM	NM_000390.4	c.1245_1246delins14	p.(Lys415AsnfsTer4)	Frameshift	Variant of Uncertain Significance	M	36	Not stated
14942101	1293	CHM	NM_000390.4	c.1244+1G>A	Splice	Splice	Likely Pathogenic	M	63	Unknown
1051434	1294	CHM	NM_000390.4	c.1408C>T	p.(Gln470Ter)	Stopgain	Likely Pathogenic	M	49	White - Other
6673806	1295	CHM	NM_000390.4	c.525_526del	p.(Glu177LysfsTer6)	Frameshift	Pathogenic	M	31	White - British
3500104	1296	CHM	NM_000390.4	c.941-2A>G	Splice	Splice	Pathogenic	M	41	White - British
6832573	1297	CHM	NM_000390.4	c.1584_1587del	p.(Val529HisfsTer7)	Frameshift	Pathogenic	M	67	Not stated
3557854	1298	CHM	NM_000390.4	c.1584_1587del	p.(Val529HisfsTer7)	Frameshift	Pathogenic	M	38	White - British

1095996	1299	CHM	NM_000390.4	c.1310_1313del	p.(Ser437ThrfsTer20)	Frameshift	Likely Pathogenic	M	53	White - Other
12262865	1300	CHM	NM_000390.4	c.116+1G>A	Splice	Splice	Pathogenic	M	60	Unknown
8458890	1301	CHM	NM_000390.4	c.737_741del	p.(Leu246GlnfsTer3)	Frameshift	Likely Pathogenic	M	64	Not stated
7376585	1301	CHM	NM_000390.4	c.737_741del	p.(Leu246GlnfsTer3)	Frameshift	Likely Pathogenic	F	70	Not stated
15042264	1302	CHM	NM_000390.4	Exon 3 deletion	Deletion	Deletion	Variant of Uncertain Significance	M	48	Unknown
6561421	1303	CHM	NM_000390.4	Exon 1-11 deletion	Deletion	Deletion	Likely Pathogenic	M	29	White - British
7857296	1304	CHM	NM_000390.4	c.1245-1G>A	Splice	Splice	Likely Pathogenic	M	41	White - British
6276556	1305	CHM	NM_000390.4	c.49+2dup	Splice	Splice	Likely Pathogenic	F	74	White - British
6855869	1306	CHM	NM_000390.4	Exon 10-15 deletion	Deletion	Deletion	Likely Pathogenic	M	70	White - British
9409434	1307	CHM	NM_000390.4	c.940G>A	p.(Gly314Arg)	Missense	Likely Pathogenic	M	58	Unknown
9458889	1308	CHM	NM_000390.4	Exon 12-13 deletion	Deletion	Deletion	Likely Pathogenic	M	37	Not stated
3672815	1309	CHM	NM_000390.4	c.715C>T	p.(Arg239Ter)	Stopgain	Pathogenic	M	68	Not stated
10061197	1310	CHM	NM_000390.4	c.1618C>T	p.(Gln540Ter)	Stopgain	Likely Pathogenic	M	88	Not stated
12707743	1311	CHM	NM_000390.4	c.715C>T	p.(Arg239Ter)	Stopgain	Pathogenic	M	32	Not stated
10245864	1312	CHM	NM_000390.4	Exon 9 deletion	Deletion	Deletion	Likely Pathogenic	M	41	Unknown
10370023	1313	CHM	NM_000390.4	c.799C>T	p.(Arg267Ter)	Stopgain	Pathogenic	M	54	Unknown
10589627	1314	CHM	NM_000390.4	c.877C>T	p.(Arg293Ter)	Stopgain	Pathogenic	M	32	White - British
6901894	1315	CHM	NM_000390.4	Exon 9 deletion	Deletion	Deletion	Likely Pathogenic	M	81	White - British
17087671	1315	CHM	NM_000390.4	Exon 9 deletion	Deletion	Deletion	Likely Pathogenic	M	28	Not stated
17098192	1315	CHM	NM_000390.4	Exon 9 deletion	Deletion	Deletion	Likely Pathogenic	M	30	Not stated
9108140	1316	CHM	NM_000390.4	Exon 1-15 deletion	Deletion	Deletion	Likely Pathogenic	M	68	Unknown
3824470	1317	CHM	NM_000390.4	Exon 1-15 deletion	Deletion	Deletion	Likely Pathogenic	M	43	Asian - Indian
10768393	1318	CHM	NM_000390.4	c.1349+1del	Splice	Splice	Likely Pathogenic	M	47	Unknown
10735906	1319	CHM	NM_000390.4	c.49+3_49+10delinsGCTT	Splice	Splice	Variant of Uncertain Significance	M	31	White - British
10544232	1320	CHM	NM_000390.4	c.831del	p.(Arg278GlufsTer13)	Frameshift	Likely Pathogenic	M	28	Any other ethnic group
10934251	1321	CHM	NM_000390.4	c.652_655del	p.(Ser218LysfsTer13)	Frameshift	Pathogenic	M	76	Unknown
10157188	1322	CHM	NM_000390.4	c.703-1_727delinsTTAGA	Splice	Splice	Likely Pathogenic	M	25	White - British
11155108	1323	CHM	NM_000390.4	c.1161_1164delinsTTT	p.(Cys388LeufsTer21)	Frameshift	Likely Pathogenic	M	33	White - British
11133765	1324	CHM	NM_000390.4	c.759del	p.(Tyr254MetfsTer37)	Frameshift	Likely Pathogenic	M	40	Unknown
1724295	1325	CHM	NM_000390.4	c.1347C>G	p.(Tyr449Ter)	Stopgain	Likely Pathogenic	M	64	White - British
11421577	1326	CHM	NM_000390.4	Exon 3-15 deletion	Deletion	Deletion	Likely Pathogenic	M	60	Not stated
11475813	1327	CHM	NM_000390.4	Exon 5-8 deletion	Deletion	Deletion	Likely Pathogenic	M	33	Asian - Other
11346348	1328	CHM	NM_000390.4	c.1079del	p.(Asn360ThrfsTer49)	Frameshift	Likely Pathogenic	M	25	White - British
11620881	1329	CHM	NM_000390.4	c.757C>T	p.(Arg253Ter)	Stopgain	Pathogenic	M	26	Not stated
11307589	1330	CHM	NM_000390.4	c.1153dup	p.(Gln385ProfsTer33)	Frameshift	Pathogenic	F	86	Unknown
18433981	1330	CHM	NM_000390.4	c.1153dup	p.(Gln385ProfsTer33)	Frameshift	Pathogenic	F	56	Not stated
6843885	1331	CHM	NM_000390.4	c.940+3del	Splice	Splice	Variant of Uncertain Significance	M	46	Not stated
12130719	1332	CHM	NM_000390.4	c.495dup	p.(Ala166CysfsTer8)	Frameshift	Likely Pathogenic	M	58	Not stated
11677077	1333	CHM	NM_000390.4	c.315-1536A>G	Splice	Splice	Variant of Uncertain Significance	M	19	Asian - Other
12861113	1334	CHM	NM_000390.4	c.675dup	p.(Arg226GlufsTer3)	Frameshift	Likely Pathogenic	M	16	Unknown
12701541	1335	CHM	NM_000390.4	c.715C>T	p.(Arg239Ter)	Stopgain	Pathogenic	M	49	Not stated
653435	1336	CHM	NM_000390.4	c.940+3del	Splice	Splice	Variant of Uncertain Significance	M	52	White - British
13134645	1337	CHM	NM_000390.4	Exon 3-4 deletion	Deletion	Deletion	Likely Pathogenic	M	27	Unknown
13311353	1338	CHM	NM_000390.4	Exon 1-7 deletion	Deletion	Deletion	Likely Pathogenic	M	20	White - British
11877025	1339	CHM	NM_000390.4	c.757C>T	p.(Arg253Ter)	Stopgain	Pathogenic	M	38	White - British
13514745	1340	CHM	NM_000390.4	c.808C>T	p.(Arg270Ter)	Stopgain	Pathogenic	M	17	Unknown
16183194	1340	CHM	NM_000390.4	c.808C>T	p.(Arg270Ter)	Stopgain	Pathogenic	M	12	Not stated
12835661	1341	CHM	NM_000390.4	c.282del	p.(Ile95PhefsTer31)	Frameshift	Likely Pathogenic	M	25	Mixed - White and Asian
13616763	1342	CHM	NM_000390.4	c.116+1G>A	Splice	Splice	Pathogenic	M	43	White - British
16475514	1343	CHM	NM_000390.4	c.116+1G>A	Splice	Splice	Pathogenic	M	39	Unknown
13727622	1344	CHM	NM_000390.4	Exon 1-15 deletion	Deletion	Deletion	Likely Pathogenic	M	43	Not stated

13999628	1344	CHM	NM_000390.4	Exon 1-15 deletion	Deletion	Deletion	Likely Pathogenic	F	38	Unknown
13589379	1345	CHM	NM_000390.4	c.1349+1del	Splice	Splice	Likely Pathogenic	M	15	Unknown
16490627	1345	CHM	NM_000390.4	c.1349+1del	Splice	Splice	Likely Pathogenic	M	12	Unknown
17536840	1345	CHM	NM_000390.4	c.1349+1del	Splice	Splice	Likely Pathogenic	F	56	Unknown
18016214	1345	CHM	NM_000390.4	c.1349+1del	Splice	Splice	Likely Pathogenic	F	26	Not stated
13801724	1346	CHM	NM_000390.4	c.1342C>T	p.(Gln448Ter)	Stopgain	Pathogenic	F	38	White - British
14819594	1347	CHM	NM_000390.4	c.940+3del	Splice	Splice	Variant of Uncertain Significance	M	41	White - British
6702142	1348	CHM	NM_000390.4	c.1048del	p.(Ser350AlafsTer3)	Frameshift	Likely Pathogenic	M	38	White - British
2483753	1349	CHM	NM_000390.4	c.649_652del	p.(Tyr217HisfsTer14)	Frameshift	Pathogenic	M	69	Any other ethnic group
15382366	1350	CHM	NM_000390.4	c.1277_1278del	p.(Arg426AsnfsTer4)	Frameshift	Likely Pathogenic	M	28	Not stated
15610895	1351	CHM	NM_000390.4	c.1584_1587del	p.(Val529HisfsTer7)	Frameshift	Pathogenic	F	64	Unknown
16324251	1351	CHM	NM_000390.4	c.1584_1587del	p.(Val529HisfsTer7)	Frameshift	Pathogenic	M	35	Not stated
14991031	1352	CHM	NM_000390.4	c.1144G>T	p.(Glu382Ter)	Stopgain	Pathogenic	M	39	Any other ethnic group
6669025	1353	CHM	NM_000390.4	c.1641del	p.(Trp548GlyfsTer7)	Frameshift	Likely Pathogenic	M	63	Not stated
15984954	1354	CHM	NM_000390.4	c.877C>T	p.(Arg293Ter)	Stopgain	Pathogenic	F	34	Any other ethnic group
16338867	1355	CHM	NM_000390.4	c.116+1G>A	Splice	Splice	Pathogenic	M	22	Not stated
10811429	1356	CHM	NM_000390.4	c.1234G>T	p.(Glu412Ter)	Stopgain	Pathogenic	M	43	Asian - Bangladeshi
16531255	1357	CHM	NM_000390.4	c.1764_1770+2del	Splice	Splice	Likely Pathogenic	F	54	White - British
16768289	1358	CHM	NM_000390.4	c.808C>T	p.(Arg270Ter)	Stopgain	Pathogenic	M	40	Not stated
16318805	1359	CHM	NM_000390.4	c.1314C>G	p.(Tyr438Ter)	Stopgain	Pathogenic	M	18	Not stated
7736763	1360	CHM	NM_000390.4	Exon 1-15 deletion	Deletion	Deletion	Likely Pathogenic	M	20	White - British
10073937	1361	CHM	NM_000390.4	c.1300_1303del	p.(Val434ArgfsTer23)	Frameshift	Likely Pathogenic	M	22	White - British
17208407	1362	CHM	NM_000390.4	c.1673C>G	p.(Ser558Ter)	Stopgain	Likely Pathogenic	M	20	Not stated
17409713	1363	CHM	NM_000390.4	c.116+1G>A	Splice	Splice	Pathogenic	M	19	Not stated
17530659	1364	CHM	NM_000390.4	c.886del	p.(Met296Ter)	Stopgain	Likely Pathogenic	M	42	Not stated
17588626	1365	CHM	NM_000390.4	Exon 10 deletion	Deletion	Deletion	Variant of Uncertain Significance	M	43	Any other ethnic group
16543127	1366	CHM	NM_000390.4	c.616dup	p.(Thr206AsnfsTer17)	Frameshift	Pathogenic	M	50	Not stated
17832380	1367	CHM	NM_000390.4	c.799C>T	p.(Arg267Ter)	Stopgain	Pathogenic	M	35	Not stated
14232203	1368	CHM	NM_000390.4	c.1300_1303del	p.(Val434ArgfsTer23)	Frameshift	Likely Pathogenic	F	39	White - British
18075056	1369	CHM	NM_000390.4	c.130G>T	p.(Gly44Ter)	Stopgain	Pathogenic	M	15	Not stated
13052325	1370	CHM	NM_000390.4	c.1573C>T	p.(Gln525Ter)	Stopgain	Pathogenic	M	16	Not stated
18308961	1371	CHM	NM_000390.4	c.315_318del	p.(Ser105ArgfsTer20)	Frameshift	Pathogenic	F	25	Unknown
18554318	1372	CHM	NM_000390.4	c.1762_1765del	p.(Val588AsnfsTer60)	Frameshift	Likely Pathogenic	M	22	Unknown
11519031	1373	CHM	NM_000390.4	c.20C>A	p.(Ser7Ter)	Stopgain	Likely Pathogenic	M	23	White - British
7706698	1374	CLCC1	NM_015127.5	c.75C>A	p.(Asp25Glu)	Missense	Likely Pathogenic	M	26	Asian - Bangladeshi
7706698	1374	CLCC1	NM_015127.5	c.75C>A	p.(Asp25Glu)	Missense	Likely Pathogenic	M	26	Asian - Bangladeshi
2670625	1375	CLN3	NM_001042432.2	c.1213C>T	p.(Arg405Trp)	Missense	Pathogenic	F	58	White - British
2670625	1375	CLN3	NM_001042432.2	Exon 7-8 deletion	Deletion	Deletion	Likely Pathogenic	F	58	White - British
3777633	1376	CLN3	NM_001042432.2	c.1213C>T	p.(Arg405Trp)	Missense	Pathogenic	F	48	White - British
3777633	1376	CLN3	NM_001042432.2	Exon 2 deletion	Deletion	Deletion	Variant of Uncertain Significance	F	48	White - British
10589025	1377	CLN3	NM_001042432.2	c.1213C>T	p.(Arg405Trp)	Missense	Pathogenic	F	44	Asian - Bangladeshi
10589025	1377	CLN3	NM_001042432.2	c.1213C>T	p.(Arg405Trp)	Missense	Pathogenic	F	44	Asian - Bangladeshi
6813638	1378	CLN3	NM_001042432.2	c.1213C>T	p.(Arg405Trp)	Missense	Pathogenic	M	60	White - British
6813638	1378	CLN3	NM_001042432.2	c.1213C>T	p.(Arg405Trp)	Missense	Pathogenic	M	60	White - British
10776149	1379	CLN3	NM_001042432.2	c.1168G>A	p.(Val390Met)	Missense	Variant of Uncertain Significance	M	52	Not stated
10776149	1379	CLN3	NM_001042432.2	Exon 7-8 deletion	Deletion	Deletion	Likely Pathogenic	M	52	Not stated
13063434	1380	CLN3	NM_001042432.2	c.565G>T	p.(Gly189Trp)	Missense	Likely Pathogenic	M	16	Asian - Pakistani
13063434	1380	CLN3	NM_001042432.2	c.565G>T	p.(Gly189Trp)	Missense	Likely Pathogenic	M	16	Asian - Pakistani
14744785	1381	CLN3	NM_001042432.2	c.647T>A	p.(Met216Lys)	Missense	Likely Pathogenic	M	49	White - British
14744785	1381	CLN3	NM_001042432.2	Exon 7-8 deletion	Deletion	Deletion	Likely Pathogenic	M	49	White - British
15476236	1382	CLN3	NM_001042432.2	Exon 7-8 deletion	Deletion	Deletion	Likely Pathogenic	F	14	White - British

15476236	1382	CLN3	NM_001042432.2	Exon 7-8 deletion	Deletion	Deletion	Likely Pathogenic	F	14	White - British
16472497	1383	CLN3	NM_001042432.2	Exon 7-8 deletion	Deletion	Deletion	Likely Pathogenic	M	14	Not stated
16472497	1383	CLN3	NM_001042432.2	Exon 7-8 deletion	Deletion	Deletion	Likely Pathogenic	M	14	Not stated
17415320	1384	CLN3	NM_001042432.2	c.1213C>T	p.(Arg405Trp)	Missense	Pathogenic	F	44	Not stated
17415320	1384	CLN3	NM_001042432.2	c.837+5G>A	Splice	Splice	Likely Pathogenic	F	44	Not stated
17286191	1385	CLN3	NM_001042432.2	Exon 7-8 deletion	Deletion	Deletion	Likely Pathogenic	M	53	Not stated
17286191	1385	CLN3	NM_001042432.2	c.875A>G	p.(Tyr292Cys)	Missense	Likely Pathogenic	M	53	Not stated
427006	1386	CLRN1	NM_174878.3	c.144T>G	p.(Asn48Lys)	Missense	Pathogenic	M	67	White - British
427006	1386	CLRN1	NM_174878.3	c.144T>G	p.(Asn48Lys)	Missense	Pathogenic	M	67	White - British
1190377	1387	CLRN1	NM_174878.3	c.149_152delinsTGTCCAAT	p.(Ser50LeufsTer12)	Frameshift	Pathogenic	M	75	White - British
1190377	1387	CLRN1	NM_174878.3	c.149_152delinsTGTCCAAT	p.(Ser50LeufsTer12)	Frameshift	Pathogenic	M	75	White - British
4121186	1388	CLRN1	NM_174878.3	c.144T>G	p.(Asn48Lys)	Missense	Pathogenic	F	46	Any other ethnic group
4121186	1388	CLRN1	NM_174878.3	c.189C>A	p.(Tyr63Ter)	Stopgain	Pathogenic	F	46	Any other ethnic group
8805040	1389	CLRN1	NM_174878.3	c.149_152delinsTGTCCAAT	p.(Ser50LeufsTer12)	Frameshift	Pathogenic	M	40	White - British
8805040	1389	CLRN1	NM_174878.3	c.149_152delinsTGTCCAAT	p.(Ser50LeufsTer12)	Frameshift	Pathogenic	M	40	White - British
8805005	1389	CLRN1	NM_174878.3	c.149_152delinsTGTCCAAT	p.(Ser50LeufsTer12)	Frameshift	Pathogenic	M	43	Not stated
8805005	1389	CLRN1	NM_174878.3	c.149_152delinsTGTCCAAT	p.(Ser50LeufsTer12)	Frameshift	Pathogenic	M	43	Not stated
16429321	1390	CLRN1	NM_174878.3	c.118T>G	p.(Cys40Gly)	Missense	Likely Pathogenic	F	23	Not stated
16429321	1390	CLRN1	NM_174878.3	c.149_152delinsTGTCCAAT	p.(Ser50LeufsTer12)	Frameshift	Pathogenic	F	23	Not stated
17482723	1391	CLRN1	NM_174878.3	c.118T>G	p.(Cys40Gly)	Missense	Likely Pathogenic	M	59	White - British
17482723	1391	CLRN1	NM_174878.3	c.221G>A	p.(Cys74Tyr)	Missense	Variant of Uncertain Significance	M	59	White - British
17630290	1392	CLRN1	NM_174878.3	c.188A>C	p.(Tyr63Ser)	Missense	Likely Pathogenic	M	44	Not stated
17630290	1392	CLRN1	NM_174878.3	c.188A>C	p.(Tyr63Ser)	Missense	Likely Pathogenic	M	44	Not stated
8711828	1393	CNGA1	NM_000087.5	c.1528C>T	p.(Arg510Ter)	Stopgain	Pathogenic	M	54	White - British
8711828	1393	CNGA1	NM_000087.5	c.1873C>T	p.(Arg625Ter)	Stopgain	Pathogenic	M	54	White - British
12338472	1394	CNGA1	NM_000087.5	c.931G>C	p.(Ala311Pro)	Missense	Likely Pathogenic	M	34	White - British
12338472	1394	CNGA1	NM_000087.5	c.1528C>T	p.(Arg510Ter)	Stopgain	Pathogenic	M	34	White - British
16482976	1395	CNGA1	NM_000087.5	c.1743_1746del	p.(Thr582SerfsTer17)	Frameshift	Pathogenic	M	45	Unknown
16482976	1395	CNGA1	NM_000087.5	c.192_193del	p.(Tyr64Ter)	Stopgain	Likely Pathogenic	M	45	Unknown
5161106	1396	CNGA1	NM_000087.5	c.947C>T	p.(Ser316Phe)	Missense	Pathogenic	M	53	White - British
5161106	1396	CNGA1	NM_000087.5	c.628G>A	p.(Asp210Asn)	Missense	Variant of Uncertain Significance	M	53	White - British
9534482	1397	CNGA1	NM_000087.5	c.817C>T	p.(Arg273Trp)	Missense	Likely Pathogenic	F	45	Black - African
9534482	1397	CNGA1	NM_000087.5	c.817C>T	p.(Arg273Trp)	Missense	Likely Pathogenic	F	45	Black - African
17755835	1398	CNGA1	NM_000087.5	c.947C>T	p.(Ser316Phe)	Missense	Pathogenic	F	55	Not stated
17755835	1398	CNGA1	NM_000087.5	c.640C>T	p.(Arg214Ter)	Stopgain	Pathogenic	F	55	Not stated
5930322	1399	CNGA3	NM_001298.3	c.1641C>A	p.(Phe547Leu)	Missense	Pathogenic	F	44	Asian - Indian
5930322	1399	CNGA3	NM_001298.3	c.1641C>A	p.(Phe547Leu)	Missense	Pathogenic	F	44	Asian - Indian
1758735	1400	CNGA3	NM_001298.3	c.1642G>A	p.(Gly548Arg)	Missense	Pathogenic	F	58	Asian - Indian
1758735	1400	CNGA3	NM_001298.3	c.1642G>A	p.(Gly548Arg)	Missense	Pathogenic	F	58	Asian - Indian
4405659	1401	CNGA3	NM_001298.3	c.1306C>T	p.(Arg436Trp)	Missense	Pathogenic	M	33	Asian - Pakistani
4405659	1401	CNGA3	NM_001298.3	c.1306C>T	p.(Arg436Trp)	Missense	Pathogenic	M	33	Asian - Pakistani
3289628	1401	CNGA3	NM_001298.3	c.1306C>T	p.(Arg436Trp)	Missense	Pathogenic	M	36	Asian - Pakistani
3289628	1401	CNGA3	NM_001298.3	c.1306C>T	p.(Arg436Trp)	Missense	Pathogenic	M	36	Asian - Pakistani
1428916	1402	CNGA3	NM_001298.3	c.586C>T	p.(Gln196Ter)	Stopgain	Likely Pathogenic	F	40	Asian - Indian
1428916	1402	CNGA3	NM_001298.3	c.586C>T	p.(Gln196Ter)	Stopgain	Likely Pathogenic	F	40	Asian - Indian
5467496	1403	CNGA3	NM_001298.3	c.1017C>G	p.(Asn339Lys)	Missense	Variant of Uncertain Significance	F	29	White - British
5467496	1403	CNGA3	NM_001298.3	c.1061A>G	p.(Tyr354Cys)	Missense	Likely Pathogenic	F	29	White - British
7410731	1404	CNGA3	NM_001298.3	c.1315C>T	p.(Arg439Trp)	Missense	Pathogenic	F	37	Not stated
7410731	1404	CNGA3	NM_001298.3	c.1315C>T	p.(Arg439Trp)	Missense	Pathogenic	F	37	Not stated
6482391	1405	CNGA3	NM_001298.3	c.1773_1776del	p.(Tyr591Ter)	Stopgain	Likely Pathogenic	F	24	Any other ethnic group
6482391	1405	CNGA3	NM_001298.3	c.725A>C	p.(His242Pro)	Missense	Variant of Uncertain Significance	F	24	Any other ethnic group

7165794	1406	CNGA3	NM_001298.3	c.1694C>T	p.(Thr565Met)	Missense	Pathogenic	F	31	White - British
7165794	1406	CNGA3	NM_001298.3	c.661C>T	p.(Arg221Ter)	Stopgain	Pathogenic	F	31	White - British
8046415	1407	CNGA3	NM_001298.3	c.107_110del	p.(His36ArgfsTer136)	Frameshift	Pathogenic	F	29	White - British
8046415	1407	CNGA3	NM_001298.3	c.1688G>A	p.(Arg563His)	Missense	Pathogenic	F	29	White - British
6087451	1408	CNGA3	NM_001298.3	c.661C>T	p.(Arg221Ter)	Stopgain	Pathogenic	M	47	Any other ethnic group
6087451	1408	CNGA3	NM_001298.3	c.848G>A	p.(Arg283Gln)	Missense	Pathogenic	M	47	Any other ethnic group
8148622	1408	CNGA3	NM_001298.3	c.661C>T	p.(Arg221Ter)	Stopgain	Pathogenic	F	52	Black - Caribbean
8148622	1408	CNGA3	NM_001298.3	c.848G>A	p.(Arg283Gln)	Missense	Pathogenic	F	52	Black - Caribbean
8660847	1408	CNGA3	NM_001298.3	c.661C>T	p.(Arg221Ter)	Stopgain	Pathogenic	M	55	Black - Other
8660847	1408	CNGA3	NM_001298.3	c.848G>A	p.(Arg283Gln)	Missense	Pathogenic	M	55	Black - Other
8661106	1408	CNGA3	NM_001298.3	c.661C>T	p.(Arg221Ter)	Stopgain	Pathogenic	F	41	Not stated
8661106	1408	CNGA3	NM_001298.3	c.848G>A	p.(Arg283Gln)	Missense	Pathogenic	F	41	Not stated
8321431	1409	CNGA3	NM_001298.3	c.1279C>T	p.(Arg427Cys)	Missense	Pathogenic	F	34	White - British
8321431	1409	CNGA3	NM_001298.3	c.847C>T	p.(Arg283Trp)	Missense	Pathogenic	F	34	White - British
8665852	1410	CNGA3	NM_001298.3	c.1306C>T	p.(Arg436Trp)	Missense	Pathogenic	M	33	White - Other
8665852	1410	CNGA3	NM_001298.3	c.1306C>T	p.(Arg436Trp)	Missense	Pathogenic	M	33	White - Other
7010415	1411	CNGA3	NM_001298.3	c.1710C>A	p.(Ser570Arg)	Missense	Likely Pathogenic	M	22	Asian - Indian
7010415	1411	CNGA3	NM_001298.3	c.1710C>A	p.(Ser570Arg)	Missense	Likely Pathogenic	M	22	Asian - Indian
9977050	1412	CNGA3	NM_001298.3	c.1641C>A	p.(Phe547Leu)	Missense	Pathogenic	M	17	Unknown
9977050	1412	CNGA3	NM_001298.3	c.1641C>A	p.(Phe547Leu)	Missense	Pathogenic	M	17	Unknown
9352013	1413	CNGA3	NM_001298.3	c.1279C>T	p.(Arg427Cys)	Missense	Pathogenic	F	31	White - British
9352013	1413	CNGA3	NM_001298.3	c.513G>A	p.(Trp171Ter)	Stopgain	Pathogenic	F	31	White - British
9938165	1414	CNGA3	NM_001298.3	c.1279C>T	p.(Arg427Cys)	Missense	Pathogenic	F	22	Not stated
9938165	1414	CNGA3	NM_001298.3	c.1279C>T	p.(Arg427Cys)	Missense	Pathogenic	F	22	Not stated
4935776	1415	CNGA3	NM_001298.3	c.1641C>A	p.(Phe547Leu)	Missense	Pathogenic	M	46	Not stated
4935776	1415	CNGA3	NM_001298.3	c.1641C>A	p.(Phe547Leu)	Missense	Pathogenic	M	46	Not stated
10265331	1416	CNGA3	NM_001298.3	c.811C>T	p.(Pro271Ser)	Missense	Likely Pathogenic	F	20	White - Other
10265331	1416	CNGA3	NM_001298.3	c.829C>T	p.(Arg277Cys)	Missense	Pathogenic	F	20	White - Other
10194253	1417	CNGA3	NM_001298.3	c.1642G>A	p.(Gly548Arg)	Missense	Pathogenic	M	22	White - British
10194253	1417	CNGA3	NM_001298.3	c.67C>T	p.(Arg23Ter)	Stopgain	Pathogenic	M	22	White - British
10308962	1418	CNGA3	NM_001298.3	c.67C>T	p.(Arg23Ter)	Stopgain	Pathogenic	F	56	Not stated
10308962	1418	CNGA3	NM_001298.3	c.67C>T	p.(Arg23Ter)	Stopgain	Pathogenic	F	56	Not stated
3570139	1419	CNGA3	NM_001298.3	c.1773_1776del	p.(Tyr591Ter)	Stopgain	Likely Pathogenic	M	65	Any other ethnic group
3570139	1419	CNGA3	NM_001298.3	c.1773_1776del	p.(Tyr591Ter)	Stopgain	Likely Pathogenic	M	65	Any other ethnic group
12654599	1420	CNGA3	NM_001298.3	c.1286T>C	p.(Val429Ala)	Missense	Likely Pathogenic	M	19	White - British
12654599	1420	CNGA3	NM_001298.3	c.608G>A	p.(Trp203Ter)	Stopgain	Likely Pathogenic	M	19	White - British
11040084	1421	CNGA3	NM_001298.3	c.1641C>A	p.(Phe547Leu)	Missense	Pathogenic	M	14	Asian - Indian
11040084	1421	CNGA3	NM_001298.3	c.2071_2077del	p.(Asp691AsnfsTer48)	Frameshift	Variant of Uncertain Significance	M	14	Asian - Indian
10170208	1421	CNGA3	NM_001298.3	c.1641C>A	p.(Phe547Leu)	Missense	Pathogenic	M	16	White - British
10170208	1421	CNGA3	NM_001298.3	c.2071_2077del	p.(Asp691AsnfsTer48)	Frameshift	Variant of Uncertain Significance	M	16	White - British
11118071	1422	CNGA3	NM_001298.3	c.1804G>A	p.(Gly602Arg)	Missense	Variant of Uncertain Significance	M	18	Asian - Indian
11118071	1422	CNGA3	NM_001298.3	c.1813A>G	p.(Ile605Val)	Missense	Variant of Uncertain Significance	M	18	Asian - Indian
11118071	1422	CNGA3	NM_001298.3	c.829C>T	p.(Arg277Cys)	Missense	Pathogenic	M	18	Asian - Indian
11156256	1423	CNGA3	NM_001298.3	c.1580T>G	p.(Leu527Arg)	Missense	Likely Pathogenic	F	28	Unknown
11156256	1423	CNGA3	NM_001298.3	c.1805G>A	p.(Gly602Glu)	Missense	Pathogenic	F	28	Unknown
11156270	1423	CNGA3	NM_001298.3	c.1580T>G	p.(Leu527Arg)	Missense	Likely Pathogenic	M	24	Unknown
11156270	1423	CNGA3	NM_001298.3	c.1805G>A	p.(Gly602Glu)	Missense	Pathogenic	M	24	Unknown
12184402	1424	CNGA3	NM_001298.3	c.667C>T	p.(Arg223Trp)	Missense	Pathogenic	F	41	Not stated
12184402	1424	CNGA3	NM_001298.3	c.848G>A	p.(Arg283Gln)	Missense	Pathogenic	F	41	Not stated
11173882	1424	CNGA3	NM_001298.3	c.667C>T	p.(Arg223Trp)	Missense	Pathogenic	M	43	White - British
11173882	1424	CNGA3	NM_001298.3	c.848G>A	p.(Arg283Gln)	Missense	Pathogenic	M	43	White - British

12184416	1424	CNGA3	NM_001298.3	c.667C>T	p.(Arg223Trp)	Missense	Pathogenic	F	34	Not stated
12184416	1424	CNGA3	NM_001298.3	c.848G>A	p.(Arg283Gln)	Missense	Pathogenic	F	34	Not stated
18313777	1425	CNGA3	NM_001298.3	c.1315C>T	p.(Arg439Trp)	Missense	Pathogenic	M	7	Asian - Pakistani
18313777	1425	CNGA3	NM_001298.3	c.1315C>T	p.(Arg439Trp)	Missense	Pathogenic	M	7	Asian - Pakistani
11707212	1426	CNGA3	NM_001298.3	c.1720T>C	p.(Ser574Pro)	Missense	Likely Pathogenic	F	20	Not stated
11707212	1426	CNGA3	NM_001298.3	c.1720T>C	p.(Ser574Pro)	Missense	Likely Pathogenic	F	20	Not stated
6511889	1427	CNGA3	NM_001298.3	c.1071C>G	p.(Tyr357Ter)	Stopgain	Likely Pathogenic	F	30	Not stated
6511889	1427	CNGA3	NM_001298.3	c.450-1G>A	Splice	Splice	Pathogenic	F	30	Not stated
7020117	1428	CNGA3	NM_001298.3	c.1774C>G	p.(Pro592Ala)	Missense	Likely Pathogenic	F	39	Asian - Pakistani
7020117	1428	CNGA3	NM_001298.3	c.1774C>G	p.(Pro592Ala)	Missense	Likely Pathogenic	F	39	Asian - Pakistani
11780488	1429	CNGA3	NM_001298.3	c.1768G>A	p.(Glu590Lys)	Missense	Pathogenic	M	13	Unknown
11780488	1429	CNGA3	NM_001298.3	c.661C>T	p.(Arg221Ter)	Stopgain	Pathogenic	M	13	Unknown
7691298	1430	CNGA3	NM_001298.3	c.1228C>T	p.(Arg410Trp)	Missense	Pathogenic	M	43	Not stated
7691298	1430	CNGA3	NM_001298.3	c.1228C>T	p.(Arg410Trp)	Missense	Pathogenic	M	43	Not stated
8381456	1431	CNGA3	NM_001298.3	c.1001C>T	p.(Ser334Phe)	Missense	Pathogenic	M	29	White - British
8381456	1431	CNGA3	NM_001298.3	c.1360A>T	p.(Lys454Ter)	Stopgain	Likely Pathogenic	M	29	White - British
11534130	1432	CNGA3	NM_001298.3	c.1228C>T	p.(Arg410Trp)	Missense	Pathogenic	F	13	Mixed - White and Asian
11534130	1432	CNGA3	NM_001298.3	c.955T>C	p.(Cys319Arg)	Missense	Pathogenic	F	13	Mixed - White and Asian
12381452	1433	CNGA3	NM_001298.3	c.1642G>A	p.(Gly548Arg)	Missense	Pathogenic	M	30	Unknown
12381452	1433	CNGA3	NM_001298.3	c.449+9_12del	Splice	Splice	Variant of Uncertain Significance	M	30	Unknown
10440569	1434	CNGA3	NM_001298.3	c.967G>C	p.(Ala323Pro)	Missense	Pathogenic	M	17	Black - Other
10440569	1434	CNGA3	NM_001298.3	c.967G>C	p.(Ala323Pro)	Missense	Pathogenic	M	17	Black - Other
12742932	1435	CNGA3	NM_001298.3	c.1641C>A	p.(Phe547Leu)	Missense	Pathogenic	F	65	Not stated
12742932	1435	CNGA3	NM_001298.3	c.1641C>A	p.(Phe547Leu)	Missense	Pathogenic	F	65	Not stated
2699332	1436	CNGA3	NM_001298.3	c.1810C>T	p.(Gln604Ter)	Stopgain	Pathogenic	M	62	Asian - Indian
2699332	1436	CNGA3	NM_001298.3	c.1810C>T	p.(Gln604Ter)	Stopgain	Pathogenic	M	62	Asian - Indian
13168861	1437	CNGA3	NM_001298.3	c.1557G>A	p.(Met519Ile)	Missense	Pathogenic	F	15	White - British
13168861	1437	CNGA3	NM_001298.3	c.1557G>A	p.(Met519Ile)	Missense	Pathogenic	F	15	White - British
12864011	1438	CNGA3	NM_001298.3	c.1228C>T	p.(Arg410Trp)	Missense	Pathogenic	M	11	Any other ethnic group
12864011	1438	CNGA3	NM_001298.3	c.1706G>A	p.(Arg569His)	Missense	Pathogenic	M	11	Any other ethnic group
13724892	1439	CNGA3	NM_001298.3	c.661C>T	p.(Arg221Ter)	Stopgain	Pathogenic	M	24	Not stated
13724892	1439	CNGA3	NM_001298.3	c.661C>T	p.(Arg221Ter)	Stopgain	Pathogenic	M	24	Not stated
14783803	1440	CNGA3	NM_001298.3	c.485A>T	p.(Asp162Val)	Missense	Likely Pathogenic	M	14	Asian - Pakistani
14783803	1440	CNGA3	NM_001298.3	c.485A>T	p.(Asp162Val)	Missense	Likely Pathogenic	M	14	Asian - Pakistani
15237214	1441	CNGA3	NM_001298.3	c.1585G>A	p.(Val529Met)	Missense	Pathogenic	M	15	Unknown
15237214	1441	CNGA3	NM_001298.3	c.1807C>T	p.(Arg603Trp)	Missense	Variant of Uncertain Significance	M	15	Unknown
15137121	1442	CNGA3	NM_001298.3	c.1768G>A	p.(Glu590Lys)	Missense	Pathogenic	M	10	Not stated
15137121	1442	CNGA3	NM_001298.3	c.830G>A	p.(Arg277His)	Missense	Pathogenic	M	10	Not stated
15288727	1443	CNGA3	NM_001298.3	c.1641C>A	p.(Phe547Leu)	Missense	Pathogenic	M	11	Not stated
15288727	1443	CNGA3	NM_001298.3	c.1773C>A	p.(Tyr591Ter)	Stopgain	Likely Pathogenic	M	11	Not stated
15431037	1444	CNGA3	NM_001298.3	c.1315C>T	p.(Arg439Trp)	Missense	Pathogenic	F	8	Not stated
15431037	1444	CNGA3	NM_001298.3	c.1315C>T	p.(Arg439Trp)	Missense	Pathogenic	F	8	Not stated
15626869	1445	CNGA3	NM_001298.3	c.1694C>T	p.(Thr565Met)	Missense	Pathogenic	F	17	Not stated
15626869	1445	CNGA3	NM_001298.3	c.293_294del	p.(Phe98SerfsTer2)	Frameshift	Likely Pathogenic	F	17	Not stated
6292012	1446	CNGA3	NM_001298.3	c.1641C>A	p.(Phe547Leu)	Missense	Pathogenic	M	24	Not stated
6292012	1446	CNGA3	NM_001298.3	c.1641C>A	p.(Phe547Leu)	Missense	Pathogenic	M	24	Not stated
9179400	1446	CNGA3	NM_001298.3	c.1641C>A	p.(Phe547Leu)	Missense	Pathogenic	F	20	Not stated
9179400	1446	CNGA3	NM_001298.3	c.1641C>A	p.(Phe547Leu)	Missense	Pathogenic	F	20	Not stated
16682042	1447	CNGA3	NM_001298.3	c.1641C>A	p.(Phe547Leu)	Missense	Pathogenic	F	38	Not stated
16682042	1447	CNGA3	NM_001298.3	c.1641C>A	p.(Phe547Leu)	Missense	Pathogenic	F	38	Not stated
17099585	1448	CNGA3	NM_001298.3	c.52del	p.(Val18Ter)	Stopgain	Likely Pathogenic	M	14	Not stated

17099585	1448	CNGA3	NM_001298.3	c.52del	p.(Val18Ter)	Stopgain	Likely Pathogenic	M	14	Not stated
15897475	1449	CNGA3	NM_001298.3	c.955T>C	p.(Cys319Arg)	Missense	Pathogenic	F	41	Asian - Indian
15897475	1449	CNGA3	NM_001298.3	c.955T>C	p.(Cys319Arg)	Missense	Pathogenic	F	41	Asian - Indian
17061659	1450	CNGA3	NM_001298.3	c.1641C>A	p.(Phe547Leu)	Missense	Pathogenic	M	49	White - Other
17061659	1450	CNGA3	NM_001298.3	c.1641C>A	p.(Phe547Leu)	Missense	Pathogenic	M	49	White - Other
17487217	1451	CNGA3	NM_001298.3	c.1279C>T	p.(Arg472Cys)	Missense	Pathogenic	F	41	Not stated
17487217	1451	CNGA3	NM_001298.3	c.450-1G>A	Splice	Splice	Pathogenic	F	41	Not stated
17380978	1452	CNGA3	NM_001298.3	c.1405G>A	p.(Ala469Thr)	Missense	Pathogenic	M	28	Not stated
17380978	1452	CNGA3	NM_001298.3	c.967G>C	p.(Ala323Pro)	Missense	Pathogenic	M	28	Not stated
17662721	1453	CNGA3	NM_001298.3	c.1793T>C	p.(Leu598Pro)	Missense	Likely Pathogenic	F	5	White - Other
17662721	1453	CNGA3	NM_001298.3	c.830G>A	p.(Arg277His)	Missense	Pathogenic	F	5	White - Other
17417448	1454	CNGA3	NM_001298.3	c.1763T>C	p.(Leu588Pro)	Missense	Likely Pathogenic	M	5	White - British
17417448	1454	CNGA3	NM_001298.3	c.667C>T	p.(Arg223Trp)	Missense	Pathogenic	M	5	White - British
17713254	1455	CNGA3	NM_001298.3	c.1306C>T	p.(Arg436Trp)	Missense	Pathogenic	F	5	Asian - Indian
17713254	1455	CNGA3	NM_001298.3	c.1306C>T	p.(Arg436Trp)	Missense	Pathogenic	F	5	Asian - Indian
17944513	1456	CNGA3	NM_001298.3	c.1315C>T	p.(Arg439Trp)	Missense	Pathogenic	F	5	Not stated
17944513	1456	CNGA3	NM_001298.3	c.1315C>T	p.(Arg439Trp)	Missense	Pathogenic	F	5	Not stated
17424147	1457	CNGA3	NM_001298.3	c.1279C>T	p.(Arg427Cys)	Missense	Pathogenic	F	7	Not stated
17424147	1457	CNGA3	NM_001298.3	c.1595A>T	p.(Asp532Val)	Missense	Likely Pathogenic	F	7	Not stated
18377393	1458	CNGA3	NM_001298.3	c.1279C>T	p.(Arg427Cys)	Missense	Pathogenic	M	15	Not stated
18377393	1458	CNGA3	NM_001298.3	c.1306C>T	p.(Arg436Trp)	Missense	Pathogenic	M	15	Not stated
18596885	1459	CNGA3	NM_001298.3	c.661C>T	p.(Arg221Ter)	Stopgain	Pathogenic	F	4	Not stated
18596885	1459	CNGA3	NM_001298.3	c.67C>T	p.(Arg23Ter)	Stopgain	Pathogenic	F	4	Not stated
18571979	1460	CNGA3	NM_001298.3	c.1228C>T	p.(Arg410Trp)	Missense	Pathogenic	M	13	Not stated
18571979	1460	CNGA3	NM_001298.3	c.1669G>A	p.(Gly557Arg)	Missense	Pathogenic	M	13	Not stated
18473321	1461	CNGA3	NM_001298.3	c.1810C>T	p.(Gln604Ter)	Stopgain	Pathogenic	M	17	Any other ethnic group
18473321	1461	CNGA3	NM_001298.3	c.1810C>T	p.(Gln604Ter)	Stopgain	Pathogenic	M	17	Any other ethnic group
18110938	1462	CNGA3	NM_001298.3	c.1641C>A	p.(Phe547Leu)	Missense	Pathogenic	F	12	Not stated
18110938	1462	CNGA3	NM_001298.3	c.1641C>A	p.(Phe547Leu)	Missense	Pathogenic	F	12	Not stated
18319426	1463	CNGA3	NM_001298.3	c.1641C>A	p.(Phe547Leu)	Missense	Pathogenic	F	7	Not stated
18319426	1463	CNGA3	NM_001298.3	c.847C>T	p.(Arg283Trp)	Missense	Pathogenic	F	7	Not stated
18319419	1463	CNGA3	NM_001298.3	c.1641C>A	p.(Phe547Leu)	Missense	Pathogenic	M	10	Not stated
18319419	1463	CNGA3	NM_001298.3	c.847C>T	p.(Arg283Trp)	Missense	Pathogenic	M	10	Not stated
18377911	1464	CNGA3	NM_001298.3	c.1306C>T	p.(Arg436Trp)	Missense	Pathogenic	M	14	Not stated
18377911	1464	CNGA3	NM_001298.3	c.1306C>T	p.(Arg436Trp)	Missense	Pathogenic	M	14	Not stated
18683391	1465	CNGA3	NM_001298.3	c.1306C>T	p.(Arg436Trp)	Missense	Pathogenic	F	4	Not stated
18683391	1465	CNGA3	NM_001298.3	c.1306C>T	p.(Arg436Trp)	Missense	Pathogenic	F	4	Not stated
18663700	1466	CNGA3	NM_001298.3	c.1279C>T	p.(Arg427Cys)	Missense	Pathogenic	F	32	Unknown
18663700	1466	CNGA3	NM_001298.3	c.667C>T	p.(Arg223Trp)	Missense	Pathogenic	F	32	Unknown
18403251	1467	CNGA3	NM_001298.3	c.670A>G	p.(Thr224Ala)	Missense	Likely Pathogenic	M	7	Not stated
18403251	1467	CNGA3	NM_001298.3	c.670A>G	p.(Thr224Ala)	Missense	Likely Pathogenic	M	7	Not stated
2068366	1468	CNGB1	NM_01297.5	c.2957A>T	p.(Asn986Ile)	Missense	Pathogenic	F	74	White - British
2068366	1468	CNGB1	NM_01297.5	c.952C>T	p.(Gln318Ter)	Stopgain	Pathogenic	F	74	White - British
2615451	1469	CNGB1	NM_01297.5	c.262C>T	p.(Gln88Ter)	Stopgain	Pathogenic	F	64	White - British
2615451	1469	CNGB1	NM_01297.5	c.664C>T	p.(Gln222Ter)	Stopgain	Pathogenic	F	64	White - British
3977399	1470	CNGB1	NM_01297.5	c.2676C>A	p.(Tyr892Ter)	Stopgain	Pathogenic	F	76	Asian - Indian
3977399	1470	CNGB1	NM_01297.5	c.2980G>T	p.(Glu994Ter)	Stopgain	Pathogenic	F	76	Asian - Indian
7190224	1471	CNGB1	NM_01297.5	c.1729del	p.(Glu577SerfsTer6)	Frameshift	Likely Pathogenic	M	61	Mixed - Other
7190224	1471	CNGB1	NM_01297.5	c.1729del	p.(Glu577SerfsTer6)	Frameshift	Likely Pathogenic	M	61	Mixed - Other
1584022	1472	CNGB1	NM_01297.5	c.1613G>A	p.(Trp538Ter)	Stopgain	Likely Pathogenic	F	77	Not stated
1584022	1472	CNGB1	NM_01297.5	c.1613G>A	p.(Trp538Ter)	Stopgain	Likely Pathogenic	F	77	Not stated

8294607	1473	CNGB1	NM_01297.5	c.2544dup	p.(Leu849AlafsTer3)	Frameshift	Pathogenic	F	58	White - British
8294607	1473	CNGB1	NM_01297.5	c.2544dup	p.(Leu849AlafsTer3)	Frameshift	Pathogenic	F	58	White - British
7109822	1474	CNGB1	NM_01297.5	c.2285G>A	p.(Arg762His)	Missense	Likely Pathogenic	M	73	Not stated
7109822	1474	CNGB1	NM_01297.5	c.2285G>A	p.(Arg762His)	Missense	Likely Pathogenic	M	73	Not stated
11182513	1475	CNGB1	NM_01297.5	Exon 28-29 deletion	Deletion	Deletion	Likely Pathogenic	M	55	White - Other
11182513	1475	CNGB1	NM_01297.5	Exon 28-29 deletion	Deletion	Deletion	Likely Pathogenic	M	55	White - Other
11570278	1476	CNGB1	NM_01297.5	c.2957A>T	p.(Asn986Ile)	Missense	Pathogenic	F	64	White - British
11570278	1476	CNGB1	NM_01297.5	c.3139_3142dup	p.(Ala1048GlyfsTer13)	Frameshift	Pathogenic	F	64	White - British
11184249	1477	CNGB1	NM_01297.5	c.2957A>T	p.(Asn986Ile)	Missense	Pathogenic	M	63	Any other ethnic group
11184249	1477	CNGB1	NM_01297.5	c.2957A>T	p.(Asn986Ile)	Missense	Pathogenic	M	63	Any other ethnic group
7069845	1478	CNGB1	NM_01297.5	c.761+2T>A	Splice	Splice	Pathogenic	F	59	Asian - Bangladeshi
7069845	1478	CNGB1	NM_01297.5	c.761+2T>A	Splice	Splice	Pathogenic	F	59	Asian - Bangladeshi
9414089	1479	CNGB1	NM_01297.5	c.217+1G>T	Splice	Splice	Likely Pathogenic	F	54	Black - African
9414089	1479	CNGB1	NM_01297.5	c.290G>C	p.(Ser97Thr)	Missense	Variant of Uncertain Significance	F	54	Black - African
12403733	1480	CNGB1	NM_01297.5	c.2544dup	p.(Leu849AlafsTer3)	Frameshift	Pathogenic	M	47	Unknown
12403733	1480	CNGB1	NM_01297.5	c.262C>T	p.(Gln88Ter)	Stopgain	Pathogenic	M	47	Unknown
12639381	1481	CNGB1	NM_01297.5	c.2544dup	p.(Leu849AlafsTer3)	Frameshift	Pathogenic	F	69	White - British
12639381	1481	CNGB1	NM_01297.5	c.2957A>T	p.(Asn986Ile)	Missense	Pathogenic	F	69	White - British
12767152	1482	CNGB1	NM_01297.5	c.2957A>T	p.(Asn986Ile)	Missense	Pathogenic	F	50	White - British
12767152	1482	CNGB1	NM_01297.5	c.2957A>T	p.(Asn986Ile)	Missense	Pathogenic	F	50	White - British
12996521	1483	CNGB1	NM_01297.5	c.2957A>T	p.(Asn986Ile)	Missense	Pathogenic	M	52	White - British
12996521	1483	CNGB1	NM_01297.5	c.2957A>T	p.(Asn986Ile)	Missense	Pathogenic	M	52	White - British
13210567	1484	CNGB1	NM_01297.5	c.2957A>T	p.(Asn986Ile)	Missense	Pathogenic	M	48	Any other ethnic group
13210567	1484	CNGB1	NM_01297.5	c.2957A>T	p.(Asn986Ile)	Missense	Pathogenic	M	48	Any other ethnic group
8072182	1485	CNGB1	NM_01297.5	c.413-1G>A	Splice	Splice	Pathogenic	F	68	White - British
8072182	1485	CNGB1	NM_01297.5	c.413-1G>A	Splice	Splice	Pathogenic	F	68	White - British
5217939	1486	CNGB1	NM_01297.5	c.3139_3142dup	p.(Ala1048GlyfsTer13)	Frameshift	Pathogenic	M	58	White - British
5217939	1486	CNGB1	NM_01297.5	c.534+1G>A	Splice	Splice	Pathogenic	M	58	White - British
16761919	1487	CNGB1	NM_01297.5	c.2540G>A	p.(Gly847Glu)	Missense	Likely Pathogenic	M	87	Not stated
16761919	1487	CNGB1	NM_01297.5	c.346C>T	p.(Gln116Ter)	Stopgain	Pathogenic	M	87	Not stated
636117	1488	CNGB1	NM_01297.5	c.2544dup	p.(Leu849AlafsTer3)	Frameshift	Pathogenic	F	66	White - British
636117	1488	CNGB1	NM_01297.5	c.2957A>T	p.(Asn986Ile)	Missense	Pathogenic	F	66	White - British
17331068	1489	CNGB1	NM_01297.5	c.2544dup	p.(Leu849AlafsTer3)	Frameshift	Pathogenic	F	50	Not stated
17331068	1489	CNGB1	NM_01297.5	c.2957A>T	p.(Asn986Ile)	Missense	Pathogenic	F	50	Not stated
16303174	1490	CNGB1	NM_01297.5	c.2957A>T	p.(Asn986Ile)	Missense	Pathogenic	M	50	Not stated
16303174	1490	CNGB1	NM_01297.5	c.2957A>T	p.(Asn986Ile)	Missense	Pathogenic	M	50	Not stated
16618573	1491	CNGB1	NM_01297.5	c.2897G>A	p.(Cys966Tyr)	Missense	Variant of Uncertain Significance	F	34	White - British
16618573	1491	CNGB1	NM_01297.5	c.2957A>T	p.(Asn986Ile)	Missense	Pathogenic	F	34	White - British
16740100	1492	CNGB1	NM_01297.5	c.1209+1G>A	Splice	Splice	Likely Pathogenic	F	27	Any other ethnic group
16740100	1492	CNGB1	NM_01297.5	c.1209+1G>A	Splice	Splice	Likely Pathogenic	F	27	Any other ethnic group
3190431	1493	CNGB3	NM_019098.5	c.1148del	p.(Thr383IlefsTer13)	Frameshift	Pathogenic	F	55	White - British
3190431	1493	CNGB3	NM_019098.5	c.1148del	p.(Thr383IlefsTer13)	Frameshift	Pathogenic	F	55	White - British
5816824	1494	CNGB3	NM_019098.5	c.1148del	p.(Thr383IlefsTer13)	Frameshift	Pathogenic	F	31	White - British
5816824	1494	CNGB3	NM_019098.5	c.889T>C	p.(Ser297Pro)	Missense	Variant of Uncertain Significance	F	31	White - British
18561591	1494	CNGB3	NM_019098.5	c.1148del	p.(Thr383IlefsTer13)	Frameshift	Pathogenic	F	18	Not stated
18561591	1494	CNGB3	NM_019098.5	c.889T>C	p.(Ser297Pro)	Missense	Variant of Uncertain Significance	F	18	Not stated
5007645	1495	CNGB3	NM_019098.5	c.1148del	p.(Thr383IlefsTer13)	Frameshift	Pathogenic	F	61	Not stated
5007645	1495	CNGB3	NM_019098.5	c.1148del	p.(Thr383IlefsTer13)	Frameshift	Pathogenic	F	61	Not stated
6585837	1496	CNGB3	NM_019098.5	c.1148del	p.(Thr383IlefsTer13)	Frameshift	Pathogenic	M	65	White - British
6585837	1496	CNGB3	NM_019098.5	c.981G>C	p.(Arg327Ser)	Missense	Variant of Uncertain Significance	M	65	White - British
5129368	1497	CNGB3	NM_019098.5	c.1810C>T	p.(Arg604Ter)	Stopgain	Pathogenic	M	37	Black - African

5129368	1497	CNGB3	NM_019098.5	c.1810C>T	p.(Arg604Ter)	Stopgain	Pathogenic	M	37	Black - African
6184884	1498	CNGB3	NM_019098.5	c.1148del	p.(Thr383IlefsTer13)	Frameshift	Pathogenic	F	35	Not stated
6184884	1498	CNGB3	NM_019098.5	c.1148del	p.(Thr383IlefsTer13)	Frameshift	Pathogenic	F	35	Not stated
6579537	1498	CNGB3	NM_019098.5	c.1148del	p.(Thr383IlefsTer13)	Frameshift	Pathogenic	F	41	Not stated
6579537	1498	CNGB3	NM_019098.5	c.1148del	p.(Thr383IlefsTer13)	Frameshift	Pathogenic	F	41	Not stated
4682677	1499	CNGB3	NM_019098.5	c.1148del	p.(Thr383IlefsTer13)	Frameshift	Pathogenic	F	29	Not stated
4682677	1499	CNGB3	NM_019098.5	c.1148del	p.(Thr383IlefsTer13)	Frameshift	Pathogenic	F	29	Not stated
6684159	1500	CNGB3	NM_019098.5	c.1148del	p.(Thr383IlefsTer13)	Frameshift	Pathogenic	M	39	Not stated
6684159	1500	CNGB3	NM_019098.5	c.1853del	p.(Thr618IlefsTer2)	Frameshift	Likely Pathogenic	M	39	Not stated
5767453	1501	CNGB3	NM_019098.5	c.1148del	p.(Thr383IlefsTer13)	Frameshift	Pathogenic	M	63	White - British
5767453	1501	CNGB3	NM_019098.5	c.1148del	p.(Thr383IlefsTer13)	Frameshift	Pathogenic	M	63	White - British
6940443	1502	CNGB3	NM_019098.5	c.1432C>T	p.(Arg478Ter)	Stopgain	Pathogenic	F	30	Not stated
6940443	1502	CNGB3	NM_019098.5	c.412del	p.(Arg138GlufsTer14)	Frameshift	Pathogenic	F	30	Not stated
689779	1503	CNGB3	NM_019098.5	c.1148del	p.(Thr383IlefsTer13)	Frameshift	Pathogenic	F	44	Any other ethnic group
689779	1503	CNGB3	NM_019098.5	c.1148del	p.(Thr383IlefsTer13)	Frameshift	Pathogenic	F	44	Any other ethnic group
6865977	1504	CNGB3	NM_019098.5	c.1148del	p.(Thr383IlefsTer13)	Frameshift	Pathogenic	F	58	White - British
6865977	1504	CNGB3	NM_019098.5	c.2105del	p.(Lys702ArgfsTer127)	Frameshift	Pathogenic	F	58	White - British
7070188	1505	CNGB3	NM_019098.5	c.1148del	p.(Thr383IlefsTer13)	Frameshift	Pathogenic	M	22	White - British
7070188	1505	CNGB3	NM_019098.5	c.1148del	p.(Thr383IlefsTer13)	Frameshift	Pathogenic	M	22	White - British
1373728	1506	CNGB3	NM_019098.5	c.1148del	p.(Thr383IlefsTer13)	Frameshift	Pathogenic	F	54	White - British
1373728	1506	CNGB3	NM_019098.5	c.1148del	p.(Thr383IlefsTer13)	Frameshift	Pathogenic	F	54	White - British
8149854	1507	CNGB3	NM_019098.5	c.1148del	p.(Thr383IlefsTer13)	Frameshift	Pathogenic	F	47	White - British
8149854	1507	CNGB3	NM_019098.5	c.1148del	p.(Thr383IlefsTer13)	Frameshift	Pathogenic	F	47	White - British
2995075	1508	CNGB3	NM_019098.5	c.1214T>C	p.(Leu405Ser)	Missense	Likely Pathogenic	F	63	Asian - Pakistani
2995075	1508	CNGB3	NM_019098.5	c.1214T>C	p.(Leu405Ser)	Missense	Likely Pathogenic	F	63	Asian - Pakistani
9944360	1509	CNGB3	NM_019098.5	c.1148del	p.(Thr383IlefsTer13)	Frameshift	Pathogenic	F	16	White - British
9944360	1509	CNGB3	NM_019098.5	c.1148del	p.(Thr383IlefsTer13)	Frameshift	Pathogenic	F	16	White - British
8360834	1510	CNGB3	NM_019098.5	c.1148del	p.(Thr383IlefsTer13)	Frameshift	Pathogenic	M	19	Not stated
8360834	1510	CNGB3	NM_019098.5	c.1148del	p.(Thr383IlefsTer13)	Frameshift	Pathogenic	M	19	Not stated
10420941	1511	CNGB3	NM_019098.5	c.1148del	p.(Thr383IlefsTer13)	Frameshift	Pathogenic	F	49	Unknown
10420941	1511	CNGB3	NM_019098.5	c.2181_2184del	p.(Glu729MetfsTer99)	Frameshift	Pathogenic	F	49	Unknown
1278689	1512	CNGB3	NM_019098.5	c.1148del	p.(Thr383IlefsTer13)	Frameshift	Pathogenic	F	39	Not stated
1278689	1512	CNGB3	NM_019098.5	c.1148del	p.(Thr383IlefsTer13)	Frameshift	Pathogenic	F	39	Not stated
10606490	1513	CNGB3	NM_019098.5	c.1148del	p.(Thr383IlefsTer13)	Frameshift	Pathogenic	M	18	White - British
10606490	1513	CNGB3	NM_019098.5	c.1148del	p.(Thr383IlefsTer13)	Frameshift	Pathogenic	M	18	White - British
9377409	1514	CNGB3	NM_019098.5	c.1148del	p.(Thr383IlefsTer13)	Frameshift	Pathogenic	F	78	Not stated
9377409	1514	CNGB3	NM_019098.5	c.1148del	p.(Thr383IlefsTer13)	Frameshift	Pathogenic	F	78	Not stated
10632544	1515	CNGB3	NM_019098.5	c.1148del	p.(Thr383IlefsTer13)	Frameshift	Pathogenic	M	17	White - Other
10632544	1515	CNGB3	NM_019098.5	c.1578+1G>A	Splice	Splice	Pathogenic	M	17	White - Other
1641667	1516	CNGB3	NM_019098.5	c.1148del	p.(Thr383IlefsTer13)	Frameshift	Pathogenic	F	45	White - British
1641667	1516	CNGB3	NM_019098.5	c.1148del	p.(Thr383IlefsTer13)	Frameshift	Pathogenic	F	45	White - British
1651901	1516	CNGB3	NM_019098.5	c.1148del	p.(Thr383IlefsTer13)	Frameshift	Pathogenic	M	39	Not stated
1651901	1516	CNGB3	NM_019098.5	c.1148del	p.(Thr383IlefsTer13)	Frameshift	Pathogenic	M	39	Not stated
11053951	1517	CNGB3	NM_019098.5	c.1006G>T	p.(Glu336Ter)	Stopgain	Pathogenic	F	25	Not stated
11053951	1517	CNGB3	NM_019098.5	c.1148del	p.(Thr383IlefsTer13)	Frameshift	Pathogenic	F	25	Not stated
11004825	1518	CNGB3	NM_019098.5	c.1148del	p.(Thr383IlefsTer13)	Frameshift	Pathogenic	M	14	White - British
11004825	1518	CNGB3	NM_019098.5	c.1856T>C	p.(Leu619Pro)	Missense	Likely Pathogenic	M	14	White - British
8340555	1519	CNGB3	NM_019098.5	c.1148del	p.(Thr383IlefsTer13)	Frameshift	Pathogenic	F	30	White - British
8340555	1519	CNGB3	NM_019098.5	c.1148del	p.(Thr383IlefsTer13)	Frameshift	Pathogenic	F	30	White - British
11231310	1520	CNGB3	NM_019098.5	c.1148del	p.(Thr383IlefsTer13)	Frameshift	Pathogenic	F	14	White - British
11231310	1520	CNGB3	NM_019098.5	c.1148del	p.(Thr383IlefsTer13)	Frameshift	Pathogenic	F	14	White - British

6875658	1521	CNGB3	NM_019098.5	c.1148del	p.(Thr383IlefsTer13)	Frameshift	Pathogenic	F	32	Asian - Pakistani
6875658	1521	CNGB3	NM_019098.5	c.1148del	p.(Thr383IlefsTer13)	Frameshift	Pathogenic	F	32	Asian - Pakistani
12068356	1522	CNGB3	NM_019098.5	c.1260del	p.(Ile420MetfsTer6)	Frameshift	Pathogenic	M	19	Asian - Bangladeshi
12068356	1522	CNGB3	NM_019098.5	c.1260del	p.(Ile420MetfsTer6)	Frameshift	Pathogenic	M	19	Asian - Bangladeshi
2657409	1523	CNGB3	NM_019098.5	c.1148del	p.(Thr383IlefsTer13)	Frameshift	Pathogenic	M	35	White - British
2657409	1523	CNGB3	NM_019098.5	c.1148del	p.(Thr383IlefsTer13)	Frameshift	Pathogenic	M	35	White - British
11780537	1524	CNGB3	NM_019098.5	c.1148del	p.(Thr383IlefsTer13)	Frameshift	Pathogenic	M	23	White - British
11780537	1524	CNGB3	NM_019098.5	c.1148del	p.(Thr383IlefsTer13)	Frameshift	Pathogenic	M	23	White - British
11825351	1525	CNGB3	NM_019098.5	c.1148del	p.(Thr383IlefsTer13)	Frameshift	Pathogenic	M	30	Not stated
11825351	1525	CNGB3	NM_019098.5	c.1148del	p.(Thr383IlefsTer13)	Frameshift	Pathogenic	M	30	Not stated
11995381	1526	CNGB3	NM_019098.5	c.1148del	p.(Thr383IlefsTer13)	Frameshift	Pathogenic	M	50	Not stated
11995381	1526	CNGB3	NM_019098.5	c.1148del	p.(Thr383IlefsTer13)	Frameshift	Pathogenic	M	50	Not stated
7317372	1527	CNGB3	NM_019098.5	c.1148del	p.(Thr383IlefsTer13)	Frameshift	Pathogenic	F	24	White - British
7317372	1527	CNGB3	NM_019098.5	c.1148del	p.(Thr383IlefsTer13)	Frameshift	Pathogenic	F	24	White - British
12209252	1528	CNGB3	NM_019098.5	c.1119G>A	p.(Trp373Ter)	Stopgain	Pathogenic	M	56	Not stated
12209252	1528	CNGB3	NM_019098.5	c.991-3T>G	Splice	Splice	Pathogenic	M	56	Not stated
12242684	1529	CNGB3	NM_019098.5	c.1148del	p.(Thr383IlefsTer13)	Frameshift	Pathogenic	M	27	Not stated
12242684	1529	CNGB3	NM_019098.5	c.1148del	p.(Thr383IlefsTer13)	Frameshift	Pathogenic	M	27	Not stated
12405413	1530	CNGB3	NM_019098.5	c.1148del	p.(Thr383IlefsTer13)	Frameshift	Pathogenic	M	17	Unknown
12405413	1530	CNGB3	NM_019098.5	c.1148del	p.(Thr383IlefsTer13)	Frameshift	Pathogenic	M	17	Unknown
12345759	1531	CNGB3	NM_019098.5	c.1148del	p.(Thr383IlefsTer13)	Frameshift	Pathogenic	M	21	Unknown
12345759	1531	CNGB3	NM_019098.5	c.1148del	p.(Thr383IlefsTer13)	Frameshift	Pathogenic	M	21	Unknown
11971098	1532	CNGB3	NM_019098.5	c.1208G>A	p.(Arg403Gln)	Missense	Variant of Uncertain Significance	F	52	Asian - Pakistani
11971098	1532	CNGB3	NM_019098.5	c.643+3A>G	Splice	Splice	Variant of Uncertain Significance	F	52	Asian - Pakistani
12579783	1533	CNGB3	NM_019098.5	c.1148del	p.(Thr383IlefsTer13)	Frameshift	Pathogenic	F	47	Unknown
12579783	1533	CNGB3	NM_019098.5	c.1148del	p.(Thr383IlefsTer13)	Frameshift	Pathogenic	F	47	Unknown
335005	1534	CNGB3	NM_019098.5	c.1148del	p.(Thr383IlefsTer13)	Frameshift	Pathogenic	F	58	White - British
335005	1534	CNGB3	NM_019098.5	c.1285del	p.(Ser429LeufsTer9)	Frameshift	Pathogenic	F	58	White - British
12350337	1535	CNGB3	NM_019098.5	c.1148del	p.(Thr383IlefsTer13)	Frameshift	Pathogenic	M	36	Unknown
12350337	1535	CNGB3	NM_019098.5	c.1148del	p.(Thr383IlefsTer13)	Frameshift	Pathogenic	M	36	Unknown
12350344	1535	CNGB3	NM_019098.5	c.1148del	p.(Thr383IlefsTer13)	Frameshift	Pathogenic	M	41	Unknown
12350344	1535	CNGB3	NM_019098.5	c.1148del	p.(Thr383IlefsTer13)	Frameshift	Pathogenic	M	41	Unknown
9340281	1536	CNGB3	NM_019098.5	c.1148del	p.(Thr383IlefsTer13)	Frameshift	Pathogenic	F	32	White - British
9340281	1536	CNGB3	NM_019098.5	c.1578+1G>A	Splice	Splice	Pathogenic	F	32	White - British
12616309	1537	CNGB3	NM_019098.5	c.1148del	p.(Thr383IlefsTer13)	Frameshift	Pathogenic	F	32	Any other ethnic group
12616309	1537	CNGB3	NM_019098.5	c.1148del	p.(Thr383IlefsTer13)	Frameshift	Pathogenic	F	32	Any other ethnic group
12565153	1538	CNGB3	NM_019098.5	c.1148del	p.(Thr383IlefsTer13)	Frameshift	Pathogenic	F	18	White - British
12565153	1538	CNGB3	NM_019098.5	c.1148del	p.(Thr383IlefsTer13)	Frameshift	Pathogenic	F	18	White - British
12675494	1539	CNGB3	NM_019098.5	c.1148del	p.(Thr383IlefsTer13)	Frameshift	Pathogenic	M	11	Not stated
12675494	1539	CNGB3	NM_019098.5	c.819_826del	p.(Arg274ValfsTer13)	Frameshift	Pathogenic	M	11	Not stated
8323293	1540	CNGB3	NM_019098.5	c.1148del	p.(Thr383IlefsTer13)	Frameshift	Pathogenic	M	19	White - British
8323293	1540	CNGB3	NM_019098.5	c.595del	p.(Glu199SerfsTer3)	Frameshift	Pathogenic	M	19	White - British
13645477	1541	CNGB3	NM_019098.5	c.1148del	p.(Thr383IlefsTer13)	Frameshift	Pathogenic	M	36	Not stated
13645477	1541	CNGB3	NM_019098.5	c.1148del	p.(Thr383IlefsTer13)	Frameshift	Pathogenic	M	36	Not stated
140972	1542	CNGB3	NM_019098.5	c.1447T>G	p.(Tyr483Asp)	Missense	Likely Pathogenic	M	67	White - British
140972	1542	CNGB3	NM_019098.5	c.190del	p.(Glu64SerfsTer19)	Frameshift	Pathogenic	M	67	White - British
16472441	1542	CNGB3	NM_019098.5	c.1447T>G	p.(Tyr483Asp)	Missense	Likely Pathogenic	M	65	Not stated
16472441	1542	CNGB3	NM_019098.5	c.190del	p.(Glu64SerfsTer19)	Frameshift	Pathogenic	M	65	Not stated
13146545	1543	CNGB3	NM_019098.5	c.1148del	p.(Thr383IlefsTer13)	Frameshift	Pathogenic	F	25	White - Other
13146545	1543	CNGB3	NM_019098.5	c.1148del	p.(Thr383IlefsTer13)	Frameshift	Pathogenic	F	25	White - Other
3110981	1544	CNGB3	NM_019098.5	c.1148del	p.(Thr383IlefsTer13)	Frameshift	Pathogenic	F	35	Unknown

3110981	1544	CNGB3	NM_019098.5	c.1426C>T	p.(Gln476Ter)	Stopgain	Pathogenic	F	35	Unknown
7379798	1545	CNGB3	NM_019098.5	c.990+1G>T	Splice	Splice	Pathogenic	M	21	White - British
7379798	1545	CNGB3	NM_019098.5	Exon 9 deletion	Deletion	Deletion	Variant of Uncertain Significance	M	21	White - British
7509039	1546	CNGB3	NM_019098.5	c.1148del	p.(Thr383IlefsTer13)	Frameshift	Pathogenic	F	48	White - Irish
7509039	1546	CNGB3	NM_019098.5	c.412del	p.(Arg138GlufsTer14)	Frameshift	Pathogenic	F	48	White - Irish
11398330	1547	CNGB3	NM_019098.5	c.1148del	p.(Thr383IlefsTer13)	Frameshift	Pathogenic	F	18	Unknown
11398330	1547	CNGB3	NM_019098.5	c.1879del	p.(Ile627PhefsTer2)	Frameshift	Likely Pathogenic	F	18	Unknown
8469054	1548	CNGB3	NM_019098.5	c.1148del	p.(Thr383IlefsTer13)	Frameshift	Pathogenic	F	21	White - British
8469054	1548	CNGB3	NM_019098.5	c.1148del	p.(Thr383IlefsTer13)	Frameshift	Pathogenic	F	21	White - British
1468760	1549	CNGB3	NM_019098.5	c.1148del	p.(Thr383IlefsTer13)	Frameshift	Pathogenic	M	47	Unknown
1468760	1549	CNGB3	NM_019098.5	c.1208G>A	p.(Arg403Gln)	Missense	Variant of Uncertain Significance	M	47	Unknown
13754537	1550	CNGB3	NM_019098.5	c.1148del	p.(Thr383IlefsTer13)	Frameshift	Pathogenic	F	10	Not stated
13754537	1550	CNGB3	NM_019098.5	c.1148del	p.(Thr383IlefsTer13)	Frameshift	Pathogenic	F	10	Not stated
1599898	1551	CNGB3	NM_019098.5	c.2103+1G>A	Splice	Splice	Likely Pathogenic	F	72	Not stated
1599898	1551	CNGB3	NM_019098.5	c.2103+1G>A	Splice	Splice	Likely Pathogenic	F	72	Not stated
15643193	1552	CNGB3	NM_019098.5	c.1578+1G>A	Splice	Splice	Pathogenic	M	8	White - Other
15643193	1552	CNGB3	NM_019098.5	c.819_826del	p.(Arg274ValfsTer13)	Frameshift	Pathogenic	M	8	White - Other
15897797	1553	CNGB3	NM_019098.5	c.1148del	p.(Thr383IlefsTer13)	Frameshift	Pathogenic	M	50	Not stated
15897797	1553	CNGB3	NM_019098.5	c.1148del	p.(Thr383IlefsTer13)	Frameshift	Pathogenic	M	50	Not stated
16331363	1554	CNGB3	NM_019098.5	c.1006G>T	p.(Glu336Ter)	Stopgain	Pathogenic	M	8	White - Other
16331363	1554	CNGB3	NM_019098.5	c.1006G>T	p.(Glu336Ter)	Stopgain	Pathogenic	M	8	White - Other
16595102	1555	CNGB3	NM_019098.5	c.1148del	p.(Thr383IlefsTer13)	Frameshift	Pathogenic	M	80	Any other ethnic group
16595102	1555	CNGB3	NM_019098.5	c.1148del	p.(Thr383IlefsTer13)	Frameshift	Pathogenic	M	80	Any other ethnic group
17017174	1556	CNGB3	NM_019098.5	c.1148del	p.(Thr383IlefsTer13)	Frameshift	Pathogenic	M	9	Any other ethnic group
17017174	1556	CNGB3	NM_019098.5	c.1148del	p.(Thr383IlefsTer13)	Frameshift	Pathogenic	M	9	Any other ethnic group
16552031	1557	CNGB3	NM_019098.5	c.1148del	p.(Thr383IlefsTer13)	Frameshift	Pathogenic	F	22	Not stated
16552031	1557	CNGB3	NM_019098.5	c.1148del	p.(Thr383IlefsTer13)	Frameshift	Pathogenic	F	22	Not stated
17168409	1558	CNGB3	NM_019098.5	c.1148del	p.(Thr383IlefsTer13)	Frameshift	Pathogenic	M	15	Not stated
17168409	1558	CNGB3	NM_019098.5	c.1751T>C	p.(Leu584Pro)	Missense	Likely Pathogenic	M	15	Not stated
17028437	1559	CNGB3	NM_019098.5	c.1148del	p.(Thr383IlefsTer13)	Frameshift	Pathogenic	M	52	White - British
17028437	1559	CNGB3	NM_019098.5	c.1148del	p.(Thr383IlefsTer13)	Frameshift	Pathogenic	M	52	White - British
2246558	1560	CNGB3	NM_019098.5	c.1148del	p.(Thr383IlefsTer13)	Frameshift	Pathogenic	F	74	White - British
2246558	1560	CNGB3	NM_019098.5	c.2103G>C	p.(Gln701His)	Missense	Variant of Uncertain Significance	F	74	White - British
16817674	1561	CNGB3	NM_019098.5	c.1148del	p.(Thr383IlefsTer13)	Frameshift	Pathogenic	F	27	Not stated
16817674	1561	CNGB3	NM_019098.5	c.819_826del	p.(Arg274ValfsTer13)	Frameshift	Pathogenic	F	27	Not stated
12851019	1562	CNGB3	NM_019098.5	c.1148del	p.(Thr383IlefsTer13)	Frameshift	Pathogenic	F	18	Not stated
12851019	1562	CNGB3	NM_019098.5	c.1148del	p.(Thr383IlefsTer13)	Frameshift	Pathogenic	F	18	Not stated
15768577	1563	CNGB3	NM_019098.5	c.1148del	p.(Thr383IlefsTer13)	Frameshift	Pathogenic	F	18	Not stated
15768577	1563	CNGB3	NM_019098.5	c.1148del	p.(Thr383IlefsTer13)	Frameshift	Pathogenic	F	18	Not stated
17536532	1564	CNGB3	NM_019098.5	c.1148del	p.(Thr383IlefsTer13)	Frameshift	Pathogenic	F	13	Not stated
17536532	1564	CNGB3	NM_019098.5	c.1148del	p.(Thr383IlefsTer13)	Frameshift	Pathogenic	F	13	Not stated
17861864	1565	CNGB3	NM_019098.5	c.1148del	p.(Thr383IlefsTer13)	Frameshift	Pathogenic	M	5	Not stated
17861864	1565	CNGB3	NM_019098.5	c.819_826del	p.(Arg274ValfsTer13)	Frameshift	Pathogenic	M	5	Not stated
18108810	1566	CNGB3	NM_019098.5	c.1148del	p.(Thr383IlefsTer13)	Frameshift	Pathogenic	F	6	Not stated
18108810	1566	CNGB3	NM_019098.5	c.1148del	p.(Thr383IlefsTer13)	Frameshift	Pathogenic	F	6	Not stated
10251674	1567	CNGB3	NM_019098.5	c.1148del	p.(Thr383IlefsTer13)	Frameshift	Pathogenic	F	34	Not stated
10251674	1567	CNGB3	NM_019098.5	c.1148del	p.(Thr383IlefsTer13)	Frameshift	Pathogenic	F	34	Not stated
18353418	1568	CNGB3	NM_019098.5	c.1148del	p.(Thr383IlefsTer13)	Frameshift	Pathogenic	F	10	Not stated
18353418	1568	CNGB3	NM_019098.5	c.852+1G>C	Splice	Splice	Pathogenic	F	10	Not stated
5939170	1569	CNNM4	NM_020184.4	c.1312dup	p.(Leu438ProfsTer9)	Frameshift	Pathogenic	M	28	Any other ethnic group
5939170	1569	CNNM4	NM_020184.4	c.1312dup	p.(Leu438ProfsTer9)	Frameshift	Pathogenic	M	28	Any other ethnic group

7956199	1570	VPS13B	NM_017890.5	c.787A>T	p.(Lys263Ter)	Stopgain	Likely Pathogenic	F	35	Not stated
7956199	1570	VPS13B	NM_017890.5	Exon 40 deletion	Deletion	Deletion	Variant of Uncertain Significance	F	35	Not stated
13103901	1571	VPS13B	NM_017890.5	c.11777_11780del	p.(Leu3926GlnfsTer15)	Frameshift	Pathogenic	M	62	Unknown
13103901	1571	VPS13B	NM_017890.5	c.3025_3026insCAATC	p.(Gln1009ProfsTer15)	Frameshift	Likely Pathogenic	M	62	Unknown
13284837	1572	VPS13B	NM_017890.5	c.5024+2T>C	Splice	Splice	Pathogenic	M	36	Asian - Pakistani
13284837	1572	VPS13B	NM_017890.5	c.5024+2T>C	Splice	Splice	Pathogenic	M	36	Asian - Pakistani
3843839	1573	COL11A1	NM_001854.4	c.2755-2A>G	Splice	Splice	Pathogenic	F	38	Not stated
3957743	1574	COL11A1	NM_001854.4	c.2556+2T>A	Splice	Splice	Likely Pathogenic	F	56	Not stated
9664374	1575	COL11A1	NM_001854.4	Exon 60-62 duplication	Duplication	Duplication	Variant of Uncertain Significance	F	31	Not stated
9469648	1576	COL11A1	NM_001854.4	c.2844+5G>A	Splice	Splice	Variant of Uncertain Significance	M	48	White - Irish
934135	1577	COL18A1	NM_001379500.1	c.3523_3524del	p.(Leu1175ValfsTer72)	Frameshift	Pathogenic	M	43	Any other ethnic group
934135	1577	COL18A1	NM_001379500.1	c.3523_3524del	p.(Leu1175ValfsTer72)	Frameshift	Pathogenic	M	43	Any other ethnic group
9008439	1578	COL18A1	NM_001379500.1	c.3523_3524del	p.(Leu1175ValfsTer72)	Frameshift	Pathogenic	F	19	White - Other
9008439	1578	COL18A1	NM_001379500.1	c.3523_3524del	p.(Leu1175ValfsTer72)	Frameshift	Pathogenic	F	19	White - Other
6990822	1579	COL18A1	NM_001379500.1	c.1897-2A>G	Splice	Splice	Pathogenic	M	24	Mixed - Other
6990822	1579	COL18A1	NM_001379500.1	c.2673del	p.(Gly892AspfsTer17)	Frameshift	Pathogenic	M	24	Mixed - Other
3147395	1580	COL18A1	NM_001379500.1	c.2673dup	p.(Gly892ArgfsTer9)	Frameshift	Pathogenic	M	34	Any other ethnic group
3147395	1580	COL18A1	NM_001379500.1	c.3524T>C	p.(Leu1175Pro)	Missense	Variant of Uncertain Significance	M	34	Any other ethnic group
17249231	1581	COL18A1	NM_001379500.1	c.3523_3524del	p.(Leu1175ValfsTer72)	Frameshift	Pathogenic	M	31	White - Other
17249231	1581	COL18A1	NM_001379500.1	c.2620+1G>C	Splice	Splice	Likely Pathogenic	M	31	White - Other
3257309	1582	COL2A1	NM_001844.5	c.3137dup	p.(Gly1047TrpfsTer11)	Frameshift	Likely Pathogenic	M	60	White - British
5444760	1582	COL2A1	NM_001844.5	c.3137dup	p.(Gly1047TrpfsTer11)	Frameshift	Likely Pathogenic	F	71	White - British
11250168	1583	COL2A1	NM_001844.5	c.3235del	p.(Ala1079LeufsTer51)	Frameshift	Likely Pathogenic	F	18	Asian - Pakistani
3515553	1583	COL2A1	NM_001844.5	c.3235del	p.(Ala1079LeufsTer51)	Frameshift	Likely Pathogenic	M	43	Asian - Pakistani
7787170	1584	COL2A1	NM_001844.5	c.2353C>T	p.(Arg785Ter)	Stopgain	Pathogenic	M	24	White - Other
191435	1585	COL2A1	NM_001844.5	c.342+4_342+5del	Splice	Splice	Variant of Uncertain Significance	F	71	White - Other
13378637	1586	COL4A1	NM_001845.5	c.1456G>A	p.(Gly486Arg)	Missense	Likely Pathogenic	F	49	Unknown
1180017	1587	CRB1	NM_201253.3	c.2688T>A	p.(Cys896Ter)	Stopgain	Pathogenic	M	58	Any other ethnic group
1180017	1587	CRB1	NM_201253.3	c.584G>T	p.(Cys195Phe)	Missense	Pathogenic	M	58	Any other ethnic group
678096	1588	CRB1	NM_201253.3	c.2555T>C	p.(Ile852Thr)	Missense	Pathogenic	F	66	White - British
678096	1588	CRB1	NM_201253.3	c.2843G>A	p.(Cys948Tyr)	Missense	Pathogenic	F	66	White - British
6472360	1589	CRB1	NM_201253.3	c.2129A>T	p.(Glu710Val)	Missense	Pathogenic	M	52	White - British
6472360	1589	CRB1	NM_201253.3	c.2843G>A	p.(Cys948Tyr)	Missense	Pathogenic	M	52	White - British
3430622	1590	CRB1	NM_201253.3	c.1576C>T	p.(Arg526Ter)	Stopgain	Pathogenic	M	35	Not stated
3430622	1590	CRB1	NM_201253.3	c.2843G>A	p.(Cys948Tyr)	Missense	Pathogenic	M	35	Not stated
1978388	1591	CRB1	NM_201253.3	c.2129A>T	p.(Glu710Val)	Missense	Pathogenic	M	44	White - British
1978388	1591	CRB1	NM_201253.3	c.2234C>T	p.(Thr745Met)	Missense	Pathogenic	M	44	White - British
3446435	1592	CRB1	NM_201253.3	c.2025G>T	p.(Trp675Cys)	Missense	Variant of Uncertain Significance	M	65	Not stated
3446435	1592	CRB1	NM_201253.3	c.3520T>G	p.(Cys1174Gly)	Missense	Likely Pathogenic	M	65	Not stated
5729016	1593	CRB1	NM_201253.3	c.750T>G	p.(Cys250Trp)	Missense	Variant of Uncertain Significance	M	36	Not stated
5729016	1593	CRB1	NM_201253.3	c.750T>G	p.(Cys250Trp)	Missense	Variant of Uncertain Significance	M	36	Not stated
5783266	1594	CRB1	NM_201253.3	c.2536G>A	p.(Gly846Arg)	Missense	Pathogenic	F	34	Asian - Pakistani
5783266	1594	CRB1	NM_201253.3	c.2536G>A	p.(Gly846Arg)	Missense	Pathogenic	F	34	Asian - Pakistani
4410629	1594	CRB1	NM_201253.3	c.2536G>A	p.(Gly846Arg)	Missense	Pathogenic	M	38	Asian - Pakistani
4410629	1594	CRB1	NM_201253.3	c.2536G>A	p.(Gly846Arg)	Missense	Pathogenic	M	38	Asian - Pakistani
3762996	1595	CRB1	NM_201253.3	c.3074G>A	p.(Ser1025Asn)	Missense	Likely Pathogenic	M	59	xed - White and Black African
3762996	1595	CRB1	NM_201253.3	c.3074G>A	p.(Ser1025Asn)	Missense	Likely Pathogenic	M	59	xed - White and Black African
6790790	1596	CRB1	NM_201253.3	c.3676G>T	p.(Gly1226Ter)	Stopgain	Pathogenic	M	51	Any other ethnic group
6790790	1596	CRB1	NM_201253.3	c.3676G>T	p.(Gly1226Ter)	Stopgain	Pathogenic	M	51	Any other ethnic group
10136118	1597	CRB1	NM_201253.3	c.2639A>G	p.(Asn880Ser)	Missense	Pathogenic	M	62	White - Irish
10136118	1597	CRB1	NM_201253.3	c.2639A>G	p.(Asn880Ser)	Missense	Pathogenic	M	62	White - Irish

7165395	1597	CRB1	NM_201253.3	c.2639A>G	p.(Asn880Ser)	Missense	Pathogenic	M	40	White - British
7165395	1597	CRB1	NM_201253.3	c.2639A>G	p.(Asn880Ser)	Missense	Pathogenic	M	40	White - British
12227445	1597	CRB1	NM_201253.3	c.2639A>G	p.(Asn880Ser)	Missense	Pathogenic	F	47	White - British
12227445	1597	CRB1	NM_201253.3	c.2639A>G	p.(Asn880Ser)	Missense	Pathogenic	F	47	White - British
4654530	1598	CRB1	NM_201253.3	c.2401A>T	p.(Lys801Ter)	Stopgain	Pathogenic	M	35	White - British
4654530	1598	CRB1	NM_201253.3	c.2843G>A	p.(Cys948Tyr)	Missense	Pathogenic	M	35	White - British
567538	1599	CRB1	NM_201253.3	c.253T>C	p.(Cys85Arg)	Missense	Likely Pathogenic	M	76	White - British
567538	1599	CRB1	NM_201253.3	c.4009_4015del	p.(Ala1337ThrfsTer2)	Frameshift	Likely Pathogenic	M	76	White - British
1345315	1600	CRB1	NM_201253.3	c.2230C>T	p.(Arg744Ter)	Stopgain	Pathogenic	F	65	White - British
1345315	1600	CRB1	NM_201253.3	c.3988del	p.(Glu1330SerfsTer11)	Frameshift	Pathogenic	F	65	White - British
6714938	1601	CRB1	NM_201253.3	c.254G>A	p.(Cys85Tyr)	Missense	Likely Pathogenic	F	42	Not stated
6714938	1601	CRB1	NM_201253.3	c.3542dup	p.(Cys1181TrpfsTer13)	Frameshift	Pathogenic	F	42	Not stated
7314194	1602	CRB1	NM_201253.3	Exon 12 deletion	Deletion	Deletion	Variant of Uncertain Significance	M	23	Asian - Pakistani
7314194	1602	CRB1	NM_201253.3	Exon 12 deletion	Deletion	Deletion	Variant of Uncertain Significance	M	23	Asian - Pakistani
6888454	1603	CRB1	NM_201253.3	c.2843G>A	p.(Cys948Tyr)	Missense	Pathogenic	M	26	White - British
6888454	1603	CRB1	NM_201253.3	c.2843G>A	p.(Cys948Tyr)	Missense	Pathogenic	M	26	White - British
7706208	1604	CRB1	NM_201253.3	c.2290C>T	p.(Arg764Cys)	Missense	Pathogenic	M	32	White - British
7706208	1604	CRB1	NM_201253.3	c.2401A>T	p.(Lys801Ter)	Stopgain	Pathogenic	M	32	White - British
7767759	1605	CRB1	NM_201253.3	c.2843G>A	p.(Cys948Tyr)	Missense	Pathogenic	M	33	White - British
7767759	1605	CRB1	NM_201253.3	c.2843G>A	p.(Cys948Tyr)	Missense	Pathogenic	M	33	White - British
3647013	1606	CRB1	NM_201253.3	c.2222T>C	p.(Met741Thr)	Missense	Pathogenic	M	58	White - Other
3647013	1606	CRB1	NM_201253.3	c.2843G>A	p.(Cys948Tyr)	Missense	Pathogenic	M	58	White - Other
8207233	1607	CRB1	NM_201253.3	c.2676+1del	Splice	Splice	Likely Pathogenic	F	25	Not stated
8207233	1607	CRB1	NM_201253.3	c.2676+1del	Splice	Splice	Likely Pathogenic	F	25	Not stated
8382968	1608	CRB1	NM_201253.3	c.2548G>A	p.(Gly850Ser)	Missense	Pathogenic	M	27	Not stated
8382968	1608	CRB1	NM_201253.3	c.3008T>C	p.(Ile1003Thr)	Missense	Likely Pathogenic	M	27	Not stated
2856111	1609	CRB1	NM_201253.3	c.2548G>A	p.(Gly850Ser)	Missense	Pathogenic	F	40	White - British
2856111	1609	CRB1	NM_201253.3	c.3307G>A	p.(Gly1103Arg)	Missense	Pathogenic	F	40	White - British
8325848	1610	CRB1	NM_201253.3	c.2843G>A	p.(Cys948Tyr)	Missense	Pathogenic	M	25	White - British
8325848	1610	CRB1	NM_201253.3	c.3520T>G	p.(Cys1174Gly)	Missense	Likely Pathogenic	M	25	White - British
8650459	1611	CRB1	NM_201253.3	c.2843G>A	p.(Cys948Tyr)	Missense	Pathogenic	M	28	White - British
8650459	1611	CRB1	NM_201253.3	c.717_718insG	p.(Gln240AlafsTer21)	Frameshift	Likely Pathogenic	M	28	White - British
3273024	1612	CRB1	NM_201253.3	c.2290C>T	p.(Arg764Cys)	Missense	Pathogenic	M	48	White - British
3273024	1612	CRB1	NM_201253.3	c.3879-1203C>G	Splice	Splice	Variant of Uncertain Significance	M	48	White - British
3255797	1612	CRB1	NM_201253.3	c.2290C>T	p.(Arg764Cys)	Missense	Pathogenic	M	50	Not stated
3255797	1612	CRB1	NM_201253.3	c.3879-1203C>G	Splice	Splice	Variant of Uncertain Significance	M	50	Not stated
11752271	1612	CRB1	NM_201253.3	c.2290C>T	p.(Arg764Cys)	Missense	Pathogenic	F	53	Unknown
11752271	1612	CRB1	NM_201253.3	c.3879-1203C>G	Splice	Splice	Variant of Uncertain Significance	F	53	Unknown
7496229	1613	CRB1	NM_201253.3	c.1006T>C	p.(Cys336Arg)	Missense	Pathogenic	M	25	Any other ethnic group
7496229	1613	CRB1	NM_201253.3	c.2129A>T	p.(Glu710Val)	Missense	Pathogenic	M	25	Any other ethnic group
8506938	1614	CRB1	NM_201253.3	c.1312T>C	p.(Cys438Arg)	Missense	Likely Pathogenic	M	33	Asian - Other
8506938	1614	CRB1	NM_201253.3	c.3655C>T	p.(Gln1219Ter)	Stopgain	Likely Pathogenic	M	33	Asian - Other
9245634	1615	CRB1	NM_201253.3	c.1431del	p.(Ser478ProfsTer24)	Frameshift	Pathogenic	M	80	White - British
9245634	1615	CRB1	NM_201253.3	c.584G>T	p.(Cys195Phe)	Missense	Pathogenic	M	80	White - British
13285026	1615	CRB1	NM_201253.3	c.1431del	p.(Ser478ProfsTer24)	Frameshift	Pathogenic	M	40	Not stated
13285026	1615	CRB1	NM_201253.3	c.498_506del	p.(Ile167_Gly169del)	Inframe indel	Pathogenic	M	40	Not stated
7216474	1616	CRB1	NM_201253.3	c.2548G>A	p.(Gly850Ser)	Missense	Pathogenic	M	56	Not stated
7216474	1616	CRB1	NM_201253.3	c.4142C>T	p.(Pro1381Leu)	Missense	Pathogenic	M	56	Not stated
9667020	1617	CRB1	NM_201253.3	c.2401A>T	p.(Lys801Ter)	Stopgain	Pathogenic	M	27	Not stated
9667020	1617	CRB1	NM_201253.3	c.4094C>A	p.(Ala1365Asp)	Missense	Likely Pathogenic	M	27	Not stated
9667027	1617	CRB1	NM_201253.3	c.2401A>T	p.(Lys801Ter)	Stopgain	Pathogenic	F	30	Not stated

9667027	1617	CRB1	NM_201253.3	c.4094C>A	p.(Ala1365Asp)	Missense	Likely Pathogenic	F	30	Not stated
9500399	1618	CRB1	NM_201253.3	c.2043T>A	p.(Cys681Ter)	Stopgain	Likely Pathogenic	M	20	Not stated
9500399	1618	CRB1	NM_201253.3	c.2843G>A	p.(Cys948Tyr)	Missense	Pathogenic	M	20	Not stated
9504606	1619	CRB1	NM_201253.3	c.2688T>A	p.(Cys896Ter)	Stopgain	Pathogenic	M	29	White - British
9504606	1619	CRB1	NM_201253.3	c.498_506del	p.(Ile167_Gly169del)	Inframe indel	Pathogenic	M	29	White - British
9567396	1620	CRB1	NM_201253.3	c.2401A>T	p.(Lys801Ter)	Stopgain	Pathogenic	F	39	Not stated
9567396	1620	CRB1	NM_201253.3	c.3035T>C	p.(Leu1012Ser)	Missense	Likely Pathogenic	F	39	Not stated
9448074	1621	CRB1	NM_201253.3	c.2401A>T	p.(Lys801Ter)	Stopgain	Pathogenic	M	45	White - British
9448074	1621	CRB1	NM_201253.3	c.3320T>C	p.(Leu1107Pro)	Missense	Pathogenic	M	45	White - British
10428872	1622	CRB1	NM_201253.3	c.2843G>A	p.(Cys948Tyr)	Missense	Pathogenic	F	18	Not stated
10428872	1622	CRB1	NM_201253.3	c.4006-1G>A	Splice	Splice	Likely Pathogenic	F	18	Not stated
10435991	1622	CRB1	NM_201253.3	c.2843G>A	p.(Cys948Tyr)	Missense	Pathogenic	F	18	Not stated
10435991	1622	CRB1	NM_201253.3	c.4006-1G>A	Splice	Splice	Likely Pathogenic	F	18	Not stated
6239022	1623	CRB1	NM_201253.3	c.2688T>A	p.(Cys896Ter)	Stopgain	Pathogenic	M	55	White - British
6239022	1623	CRB1	NM_201253.3	c.2843G>A	p.(Cys948Tyr)	Missense	Pathogenic	M	55	White - British
11561591	1624	CRB1	NM_201253.3	c.2555T>C	p.(Ile852Thr)	Missense	Pathogenic	F	25	Unknown
11561591	1624	CRB1	NM_201253.3	c.3307G>A	p.(Gly1103Arg)	Missense	Pathogenic	F	25	Unknown
11746104	1625	CRB1	NM_201253.3	c.2555T>C	p.(Ile852Thr)	Missense	Pathogenic	M	25	Unknown
11746104	1625	CRB1	NM_201253.3	c.3247del	p.(Cys1143AlafsTer67)	Frameshift	Likely Pathogenic	M	25	Unknown
11855213	1626	CRB1	NM_201253.3	c.1712A>C	p.(Glu571Ala)	Missense	Likely Pathogenic	F	23	Not stated
11855213	1626	CRB1	NM_201253.3	c.2843G>A	p.(Cys948Tyr)	Missense	Pathogenic	F	23	Not stated
11839911	1627	CRB1	NM_201253.3	c.2308G>A	p.(Gly770Ser)	Missense	Pathogenic	F	40	Not stated
11839911	1627	CRB1	NM_201253.3	c.4006-1G>T	Splice	Splice	Pathogenic	F	40	Not stated
11681165	1628	CRB1	NM_201253.3	c.1183G>T	p.(Glu395Ter)	Stopgain	Pathogenic	F	23	Black - African
11681165	1628	CRB1	NM_201253.3	c.2220dup	p.(Met741HisfsTer49)	Frameshift	Pathogenic	F	23	Black - African
11780446	1629	CRB1	NM_201253.3	c.1831T>C	p.(Ser611Pro)	Missense	Likely Pathogenic	M	45	Unknown
11780446	1629	CRB1	NM_201253.3	c.1831T>C	p.(Ser611Pro)	Missense	Likely Pathogenic	M	45	Unknown
12440210	1630	CRB1	NM_201253.3	c.2401A>T	p.(Lys801Ter)	Stopgain	Pathogenic	F	12	White - British
12440210	1630	CRB1	NM_201253.3	c.2843-1G>A	Splice	Splice	Likely Pathogenic	F	12	White - British
12756141	1631	CRB1	NM_201253.3	c.2843G>A	p.(Cys948Tyr)	Missense	Pathogenic	M	36	Not stated
12756141	1631	CRB1	NM_201253.3	c.584G>T	p.(Cys195Phe)	Missense	Pathogenic	M	36	Not stated
12713616	1632	CRB1	NM_201253.3	c.1673T>C	p.(Ile558Thr)	Missense	Variant of Uncertain Significance	M	47	Any other ethnic group
12713616	1632	CRB1	NM_201253.3	c.1673T>C	p.(Ile558Thr)	Missense	Variant of Uncertain Significance	M	47	Any other ethnic group
12919696	1633	CRB1	NM_201253.3	c.2688T>A	p.(Cys896Ter)	Stopgain	Pathogenic	F	18	Not stated
12919696	1633	CRB1	NM_201253.3	c.498_506del	p.(Ile167_Gly169del)	Inframe indel	Pathogenic	F	18	Not stated
11292490	1634	CRB1	NM_201253.3	c.2548G>A	p.(Gly850Ser)	Missense	Pathogenic	F	16	Unknown
11292490	1634	CRB1	NM_201253.3	c.4006-10A>G	Splice	Splice	Likely Pathogenic	F	16	Unknown
12791267	1635	CRB1	NM_201253.3	c.1183G>T	p.(Glu395Ter)	Stopgain	Pathogenic	M	19	Unknown
12791267	1635	CRB1	NM_201253.3	c.988+1G>T	Splice	Splice	Likely Pathogenic	M	19	Unknown
13523992	1636	CRB1	NM_201253.3	c.2291G>A	p.(Arg764His)	Missense	Pathogenic	F	40	Not stated
13523992	1636	CRB1	NM_201253.3	c.3014A>T	p.(Asp1005Val)	Missense	Likely Pathogenic	F	40	Not stated
13168476	1637	CRB1	NM_201253.3	c.1459T>C	p.(Ser487Pro)	Missense	Likely Pathogenic	F	37	White - Other
13168476	1637	CRB1	NM_201253.3	c.1459T>C	p.(Ser487Pro)	Missense	Likely Pathogenic	F	37	White - Other
10356870	1638	CRB1	NM_201253.3	c.2017A>G	p.(Lys673Glu)	Missense	Likely Pathogenic	M	43	Not stated
10356870	1638	CRB1	NM_201253.3	c.2839G>A	p.(Glu947Lys)	Missense	Likely Pathogenic	M	43	Not stated
15321193	1639	CRB1	NM_201253.3	c.3625G>A	p.(Val1209Met)	Missense	Variant of Uncertain Significance	M	39	White - Other
15321193	1639	CRB1	NM_201253.3	c.4142C>G	p.(Pro1381Leu)	Missense	Likely Pathogenic	M	39	White - Other
15321193	1639	CRB1	NM_201253.3	c.498_506del	p.(Ile167_Gly169del)	Inframe indel	Pathogenic	M	39	White - Other
15508485	1640	CRB1	NM_201253.3	c.2290C>T	p.(Arg764Cys)	Missense	Pathogenic	F	34	Any other ethnic group
15508485	1640	CRB1	NM_201253.3	c.498_506del	p.(Ile167_Gly169del)	Inframe indel	Pathogenic	F	34	Any other ethnic group
15550450	1641	CRB1	NM_201253.3	c.2843G>A	p.(Cys948Tyr)	Missense	Pathogenic	M	11	Unknown

15550450	1641	CRB1	NM_201253.3	c.2843G>A	p.(Cys948Tyr)	Missense	Pathogenic	M	11	Unknown
13881027	1642	CRB1	NM_201253.3	c.4082T>C	p.(Leu1361Pro)	Missense	Likely Pathogenic	M	34	Asian - Other
13881027	1642	CRB1	NM_201253.3	c.4082T>C	p.(Leu1361Pro)	Missense	Likely Pathogenic	M	34	Asian - Other
15881270	1643	CRB1	NM_201253.3	c.2308G>T	p.(Gly770Cys)	Missense	Likely Pathogenic	M	17	Not stated
15881270	1643	CRB1	NM_201253.3	c.498_506del	p.(Ile167_Gly169del)	Inframe indel	Pathogenic	M	17	Not stated
15931404	1644	CRB1	NM_201253.3	c.2234C>T	p.(Thr745Met)	Missense	Pathogenic	M	12	Not stated
15931404	1644	CRB1	NM_201253.3	c.2506C>A	p.(Pro836Thr)	Missense	Pathogenic	M	12	Not stated
3885734	1645	CRB1	NM_201253.3	c.2506C>A	p.(Pro836Thr)	Missense	Pathogenic	M	74	Not stated
3885734	1645	CRB1	NM_201253.3	c.2506C>A	p.(Pro836Thr)	Missense	Pathogenic	M	74	Not stated
16593156	1646	CRB1	NM_201253.3	c.2129A>T	p.(Glu710Val)	Missense	Pathogenic	M	24	White - British
16593156	1646	CRB1	NM_201253.3	c.3988del	p.(Glu1330SerfsTer11)	Frameshift	Pathogenic	M	24	White - British
6374913	1647	CRB1	NM_201253.3	c.498_506del	p.(Ile167_Gly169del)	Inframe indel	Pathogenic	M	47	Not stated
6374913	1647	CRB1	NM_201253.3	c.584G>T	p.(Cys195Phe)	Missense	Pathogenic	M	47	Not stated
15786707	1648	CRB1	NM_201253.3	c.1183G>T	p.(Glu395Ter)	Stopgain	Pathogenic	M	13	Black - African
15786707	1648	CRB1	NM_201253.3	c.2506C>A	p.(Pro836Thr)	Missense	Pathogenic	M	13	Black - African
16817429	1649	CRB1	NM_201253.3	c.2401A>T	p.(Lys801Ter)	Stopgain	Pathogenic	F	34	White - British
16817429	1649	CRB1	NM_201253.3	c.2548G>A	p.(Gly850Ser)	Missense	Pathogenic	F	34	White - British
17099823	1650	CRB1	NM_201253.3	c.2401A>T	p.(Lys801Ter)	Stopgain	Pathogenic	M	17	Not stated
17099823	1650	CRB1	NM_201253.3	c.988+6T>G	Splice	Splice	Variant of Uncertain Significance	M	17	Not stated
17896241	1651	CRB1	NM_201253.3	c.2401A>T	p.(Lys801Ter)	Stopgain	Pathogenic	F	5	White - British
17896241	1651	CRB1	NM_201253.3	c.2688T>A	p.(Cys896Ter)	Stopgain	Pathogenic	F	5	White - British
18088951	1652	CRB1	NM_201253.3	c.2401A>T	p.(Lys801Ter)	Stopgain	Pathogenic	M	52	White - British
18088951	1652	CRB1	NM_201253.3	c.498_506del	p.(Ile167_Gly169del)	Inframe indel	Pathogenic	M	52	White - British
18596871	1653	CRB1	NM_201253.3	c.1339dup	p.(His447ProfsTer7)	Frameshift	Pathogenic	F	4	Not stated
18596871	1653	CRB1	NM_201253.3	c.2401A>T	p.(Lys801Ter)	Stopgain	Pathogenic	F	4	Not stated
2409322	1654	CRX	NM_000554.6	c.821del	p.(Gly274AlafsTer97)	Frameshift	Likely Pathogenic	F	85	White - British
1224341	1655	CRX	NM_000554.6	c.121C>T	p.(Arg41Trp)	Missense	Pathogenic	F	61	Not stated
13423045	1655	CRX	NM_000554.6	c.121C>T	p.(Arg41Trp)	Missense	Pathogenic	M	67	Unknown
1436847	1656	CRX	NM_000554.6	c.568_590del	p.(Pro190GlyfsTer38)	Frameshift	Likely Pathogenic	M	61	Asian - Bangladeshi
6989310	1656	CRX	NM_000554.6	c.568_590del	p.(Pro190GlyfsTer38)	Frameshift	Likely Pathogenic	F	36	Any other ethnic group
5974660	1657	CRX	NM_000554.6	c.268C>T	p.(Arg90Trp)	Missense	Pathogenic	M	55	Not stated
8447060	1658	CRX	NM_000554.6	c.121C>T	p.(Arg41Trp)	Missense	Pathogenic	M	53	White - British
4741855	1659	CRX	NM_000554.6	c.121C>T	p.(Arg41Trp)	Missense	Pathogenic	M	53	Asian - Other
17944639	1659	CRX	NM_000554.6	c.121C>T	p.(Arg41Trp)	Missense	Pathogenic	M	58	Any other ethnic group
6979755	1660	CRX	NM_000554.6	c.522_523dup	p.(Gln175ArgfsTer13)	Frameshift	Likely Pathogenic	M	22	White - British
7765183	1661	CRX	NM_000554.6	c.121C>T	p.(Arg41Trp)	Missense	Pathogenic	F	64	White - British
3383722	1662	CRX	NM_000554.6	c.269G>C	p.(Arg90Pro)	Missense	Likely Pathogenic	M	81	Not stated
6706279	1663	CRX	NM_000554.6	c.272G>A	p.(Arg91Lys)	Missense	Likely Pathogenic	M	73	Black - African
389899	1664	CRX	NM_000554.6	c.119G>A	p.(Arg40Gln)	Missense	Pathogenic	F	66	White - British
11562669	1665	CRX	NM_000554.6	c.570del	p.(Tyr191MetfsTer3)	Frameshift	Likely Pathogenic	F	13	Any other ethnic group
6383691	1666	CRX	NM_000554.6	c.121C>T	p.(Arg41Trp)	Missense	Pathogenic	F	83	White - British
11945926	1667	CRX	NM_000554.6	c.295C>T	p.(Gln99Ter)	Stopgain	Likely Pathogenic	M	78	Not stated
12791078	1668	CRX	NM_000554.6	c.615del	p.(Ser206ProfsTer13)	Frameshift	Likely Pathogenic	M	49	Asian - Other
10384366	1669	CRX	NM_000554.6	c.523C>T	p.(Gln175Ter)	Stopgain	Pathogenic	F	21	Asian - Indian
15519811	1670	CRX	NM_000554.6	c.121C>T	p.(Arg41Trp)	Missense	Pathogenic	M	80	Any other ethnic group
15458855	1671	CRX	NM_000554.6	c.579del	p.(Met193IlefsTer26)	Frameshift	Likely Pathogenic	M	61	White - British
12661319	1672	CRX	NM_000554.6	Exon 2-4 deletion	Deletion	Deletion	Likely Pathogenic	M	61	Black - African
17225256	1673	CRX	NM_000554.6	c.268C>T	p.(Arg90Trp)	Missense	Pathogenic	M	56	Black - African
16973319	1674	CRX	NM_000554.6	c.121C>T	p.(Arg41Trp)	Missense	Pathogenic	F	77	White - British
12923259	1675	CRX	NM_000554.6	c.121C>T	p.(Arg41Trp)	Missense	Pathogenic	F	47	Any other ethnic group
5835024	1676	CTNNA1	NM_001903.5	c.1316C>T	p.(Ser439Phe)	Missense	Variant of Uncertain Significance	F	62	White - British

6101507	1676	CTNNA1	NM_001903.5	c.1316C>T	p.(Ser439Phe)	Missense	Variant of Uncertain Significance	M	65	White - British
15573914	1677	CTNNA1	NM_001903.5	c.1316C>T	p.(Ser439Phe)	Missense	Variant of Uncertain Significance	F	40	White - British
15568790	1678	CTNNA1	NM_001903.5	c.965C>T	p.(Ser322Leu)	Missense	Likely Pathogenic	F	34	Not stated
15271878	1679	CTNNA1	NM_001903.5	c.1294G>A	p.(Glu432Lys)	Missense	Variant of Uncertain Significance	F	39	White - British
5125364	1680	C1QTNF5	NM_015645.5	c.489C>G	p.(Ser163Arg)	Missense	Pathogenic	M	78	Not stated
15480604	1681	C1QTNF5	NM_015645.5	c.489C>G	p.(Ser163Arg)	Missense	Pathogenic	F	51	Unknown
10395685	1682	C1QTNF5	NM_015645.5	c.489C>G	p.(Ser163Arg)	Missense	Pathogenic	F	77	Unknown
15270478	1682	C1QTNF5	NM_015645.5	c.489C>G	p.(Ser163Arg)	Missense	Pathogenic	F	72	Any other ethnic group
15444876	1682	C1QTNF5	NM_015645.5	c.489C>G	p.(Ser163Arg)	Missense	Pathogenic	M	57	Not stated
10501532	1683	C1QTNF5	NM_015645.5	c.489C>G	p.(Ser163Arg)	Missense	Pathogenic	M	86	White - British
11560219	1684	C1QTNF5	NM_015645.5	c.489C>G	p.(Ser163Arg)	Missense	Pathogenic	M	76	Not stated
11602527	1685	C1QTNF5	NM_015645.5	c.556C>T	p.(Pro186Ser)	Missense	Likely Pathogenic	M	78	Unknown
11602527	1685	C1QTNF5	NM_015645.5	c.569C>G	p.(Ser190Trp)	Missense	Likely Pathogenic	M	78	Unknown
11935643	1686	C1QTNF5	NM_015645.5	c.489C>G	p.(Ser163Arg)	Missense	Pathogenic	M	55	Unknown
3301304	1687	C1QTNF5	NM_015645.5	c.489C>G	p.(Ser163Arg)	Missense	Pathogenic	F	70	White - British
18172832	1688	C1QTNF5	NM_015645.5	c.489C>G	p.(Ser163Arg)	Missense	Pathogenic	F	65	White - British
9120999	1689	C1QTNF5	NM_015645.5	c.486C>A	p.(Ser163Arg)	Missense	Variant of Uncertain Significance	F	74	Any other ethnic group
18233179	1690	C1QTNF5	NM_015645.5	c.562C>A	p.(Pro188Thr)	Missense	Pathogenic	F	68	Not stated
10183767	1691	CWC27	NM_0058694	c.1002dup	p.(Val335SerfsTer13)	Frameshift	Pathogenic	M	20	White - British
10183767	1691	CWC27	NM_0058694	c.1002dup	p.(Val335SerfsTer13)	Frameshift	Pathogenic	M	20	White - British
10506383	1692	CYP1B1	NM_000104.4	c.1345del	p.(Asp449MetfsTer8)	Frameshift	Pathogenic	F	19	White - British
10506383	1692	CYP1B1	NM_000104.4	c.1345del	p.(Asp449MetfsTer8)	Frameshift	Pathogenic	F	19	White - British
12231470	1693	CYP1B1	NM_000104.4	c.1103G>A	p.(Arg368His)	Missense	Pathogenic	M	15	White - British
12231470	1693	CYP1B1	NM_000104.4	c.1169G>A	p.(Arg390His)	Missense	Pathogenic	M	15	White - British
12818665	1694	CYP1B1	NM_000104.4	c.862delinsCC	p.(Ala288ProfsTer39)	Frameshift	Likely Pathogenic	F	11	Asian - Pakistani
12818665	1694	CYP1B1	NM_000104.4	c.862delinsCC	p.(Ala288ProfsTer39)	Frameshift	Likely Pathogenic	F	11	Asian - Pakistani
1416540	1695	CYP4V2	NM_207352.4	c.197T>G	p.(Met66Arg)	Missense	Pathogenic	M	75	Asian - Indian
1416540	1695	CYP4V2	NM_207352.4	c.197T>G	p.(Met66Arg)	Missense	Pathogenic	M	75	Asian - Indian
6046298	1696	CYP4V2	NM_207352.4	c.1199G>A	p.(Arg400His)	Missense	Pathogenic	M	51	Any other ethnic group
6046298	1696	CYP4V2	NM_207352.4	c.802-8_810delinsGC	Splice	Splice	Pathogenic	M	51	Any other ethnic group
1700936	1697	CYP4V2	NM_207352.4	c.197T>G	p.(Met66Arg)	Missense	Pathogenic	F	68	Asian - Indian
1700936	1697	CYP4V2	NM_207352.4	c.197T>G	p.(Met66Arg)	Missense	Pathogenic	F	68	Asian - Indian
1864617	1697	CYP4V2	NM_207352.4	c.197T>G	p.(Met66Arg)	Missense	Pathogenic	F	68	Asian - Indian
1864617	1697	CYP4V2	NM_207352.4	c.197T>G	p.(Met66Arg)	Missense	Pathogenic	F	68	Asian - Indian
3227895	1698	CYP4V2	NM_207352.4	c.77G>A	p.(Gly26Asp)	Missense	Variant of Uncertain Significance	F	56	Unknown
3227895	1698	CYP4V2	NM_207352.4	c.987+3A>G	Splice	Splice	Variant of Uncertain Significance	F	56	Unknown
6870317	1699	CYP4V2	NM_207352.4	c.998C>A	p.(Thr333Lys)	Missense	Likely Pathogenic	F	73	White - British
6870317	1699	CYP4V2	NM_207352.4	c.998C>A	p.(Thr333Lys)	Missense	Likely Pathogenic	F	73	White - British
8593647	1700	CYP4V2	NM_207352.4	c.677T>A	p.(Met226Lys)	Missense	Variant of Uncertain Significance	F	43	Not stated
8593647	1700	CYP4V2	NM_207352.4	c.677T>A	p.(Met226Lys)	Missense	Variant of Uncertain Significance	F	43	Not stated
10722361	1701	CYP4V2	NM_207352.4	c.1393A>G	p.(Arg465Gly)	Missense	Pathogenic	F	68	White - Other
10722361	1701	CYP4V2	NM_207352.4	c.1393A>G	p.(Arg465Gly)	Missense	Pathogenic	F	68	White - Other
10107964	1702	CYP4V2	NM_207352.4	c.197T>G	p.(Met66Arg)	Missense	Pathogenic	M	46	Not stated
10107964	1702	CYP4V2	NM_207352.4	c.197T>G	p.(Met66Arg)	Missense	Pathogenic	M	46	Not stated
10087454	1703	CYP4V2	NM_207352.4	c.283G>A	p.(Gly95Arg)	Missense	Likely Pathogenic	M	30	Unknown
10087454	1703	CYP4V2	NM_207352.4	c.637_641del	p.(Ser213Ter)	Stopgain	Likely Pathogenic	M	30	Unknown
11129957	1704	CYP4V2	NM_207352.4	c.802-8_810delinsGC	Splice	Splice	Pathogenic	F	50	Not stated
11129957	1704	CYP4V2	NM_207352.4	c.802-8_810delinsGC	Splice	Splice	Pathogenic	F	50	Not stated
9030461	1705	CYP4V2	NM_207352.4	c.197T>G	p.(Met66Arg)	Missense	Pathogenic	F	46	Asian - Other
9030461	1705	CYP4V2	NM_207352.4	c.197T>G	p.(Met66Arg)	Missense	Pathogenic	F	46	Asian - Other
10505221	1706	CYP4V2	NM_207352.4	c.76G>C	p.(Gly26Arg)	Missense	Variant of Uncertain Significance	M	48	Any other ethnic group

10505221	1706	CYP4V2	NM_207352.4	c.802-8_810delinsGC	Splice	Splice	Pathogenic	M	48	Any other ethnic group
3249553	1707	CYP4V2	NM_207352.4	c.197T>G	p.(Met66Arg)	Missense	Pathogenic	F	72	Asian - Indian
3249553	1707	CYP4V2	NM_207352.4	c.197T>G	p.(Met66Arg)	Missense	Pathogenic	F	72	Asian - Indian
11986372	1708	CYP4V2	NM_207352.4	c.197T>G	p.(Met66Arg)	Missense	Pathogenic	M	51	Asian - Indian
11986372	1708	CYP4V2	NM_207352.4	c.197T>G	p.(Met66Arg)	Missense	Pathogenic	M	51	Asian - Indian
6968303	1708	CYP4V2	NM_207352.4	c.197T>G	p.(Met66Arg)	Missense	Pathogenic	M	85	Asian - Other
6968303	1708	CYP4V2	NM_207352.4	c.197T>G	p.(Met66Arg)	Missense	Pathogenic	M	85	Asian - Other
5846644	1708	CYP4V2	NM_207352.4	c.197T>G	p.(Met66Arg)	Missense	Pathogenic	M	48	Asian - Indian
5846644	1708	CYP4V2	NM_207352.4	c.197T>G	p.(Met66Arg)	Missense	Pathogenic	M	48	Asian - Indian
17775127	1708	CYP4V2	NM_207352.4	c.197T>G	p.(Met66Arg)	Missense	Pathogenic	F	22	Unknown
17775127	1708	CYP4V2	NM_207352.4	c.197T>G	p.(Met66Arg)	Missense	Pathogenic	F	22	Unknown
6967953	1709	CYP4V2	NM_207352.4	c.279G>A	p.(Trp93Ter)	Stopgain	Likely Pathogenic	F	41	Asian - Pakistani
6967953	1709	CYP4V2	NM_207352.4	c.279G>A	p.(Trp93Ter)	Stopgain	Likely Pathogenic	F	41	Asian - Pakistani
12231708	1710	CYP4V2	NM_207352.4	c.802-8_810delinsGC	Splice	Splice	Pathogenic	M	44	Unknown
12231708	1710	CYP4V2	NM_207352.4	c.802-8_810delinsGC	Splice	Splice	Pathogenic	M	44	Unknown
3300212	1711	CYP4V2	NM_207352.4	c.197T>G	p.(Met66Arg)	Missense	Pathogenic	F	65	Asian - Indian
3300212	1711	CYP4V2	NM_207352.4	c.197T>G	p.(Met66Arg)	Missense	Pathogenic	F	65	Asian - Indian
16159800	1711	CYP4V2	NM_207352.4	c.197T>G	p.(Met66Arg)	Missense	Pathogenic	M	63	Asian - Indian
16159800	1711	CYP4V2	NM_207352.4	c.197T>G	p.(Met66Arg)	Missense	Pathogenic	M	63	Asian - Indian
12709955	1712	CYP4V2	NM_207352.4	c.197T>G	p.(Met66Arg)	Missense	Pathogenic	M	45	Asian - Indian
12709955	1712	CYP4V2	NM_207352.4	c.197T>G	p.(Met66Arg)	Missense	Pathogenic	M	45	Asian - Indian
15244641	1713	CYP4V2	NM_207352.4	c.802-8_810delinsGC	Splice	Splice	Pathogenic	M	55	Unknown
15244641	1713	CYP4V2	NM_207352.4	c.992A>C	p.(His331Pro)	Missense	Pathogenic	M	55	Unknown
17482380	1714	CYP4V2	NM_207352.4	c.414-1G>A	Splice	Splice	Pathogenic	F	34	White - Other
17482380	1714	CYP4V2	NM_207352.4	c.414-1G>A	Splice	Splice	Pathogenic	F	34	White - Other
18172496	1715	CYP4V2	NM_207352.4	c.1168C>T	p.(Arg390Cys)	Missense	Pathogenic	F	36	Any other ethnic group
18172496	1715	CYP4V2	NM_207352.4	c.1168C>T	p.(Arg390Cys)	Missense	Pathogenic	F	36	Any other ethnic group
5640396	1716	DRAM2	NM_178454.6	c.362A>T	p.(His121Leu)	Missense	Variant of Uncertain Significance	M	56	Not stated
5640396	1716	DRAM2	NM_178454.6	c.362A>T	p.(His121Leu)	Missense	Variant of Uncertain Significance	M	56	Not stated
8672866	1717	DRAM2	NM_178454.6	c.217_225del	p.(Val73_Tyr75del)	Inframe indel	Variant of Uncertain Significance	F	47	Not stated
8672866	1717	DRAM2	NM_178454.6	c.79C>T	p.(Tyr27His)	Missense	Likely Pathogenic	F	47	Not stated
7500037	1718	DRAM2	NM_178454.6	c.470G>A	p.(Trp157Ter)	Stopgain	Likely Pathogenic	F	53	White - British
7500037	1718	DRAM2	NM_178454.6	c.677dup	p.(Tyr226Ter)	Stopgain	Likely Pathogenic	F	53	White - British
11263286	1719	DRAM2	NM_178454.6	c.104A>G	p.(His35Arg)	Missense	Variant of Uncertain Significance	F	56	Not stated
11263286	1719	DRAM2	NM_178454.6	c.737T>C	p.(Leu246Pro)	Missense	Variant of Uncertain Significance	F	56	Not stated
14798034	1720	DRAM2	NM_178454.6	c.700_706del	p.(Ser234GlyfsTer9)	Frameshift	Likely Pathogenic	M	43	Unknown
14798034	1720	DRAM2	NM_178454.6	c.700_706del	p.(Ser234GlyfsTer9)	Frameshift	Likely Pathogenic	M	43	Unknown
17555236	1721	DRAM2	NM_178454.6	c.131G>A	p.(Ser44Asn)	Missense	Pathogenic	F	70	White - British
17555236	1721	DRAM2	NM_178454.6	c.92dup	p.(Thr32AsnfsTer14)	Frameshift	Pathogenic	F	70	White - British
17706373	1722	DRAM2	NM_178454.6	c.217_225del	p.(Val73_Tyr75del)	Inframe indel	Variant of Uncertain Significance	M	63	Not stated
17706373	1722	DRAM2	NM_178454.6	c.737T>C	p.(Leu246Pro)	Missense	Variant of Uncertain Significance	M	63	Not stated
15457448	1723	DYNC2H1	NM_001080463.2	c.9836C>A	p.(Ser3279Ter)	Stopgain	Likely Pathogenic	M	51	Any other ethnic group
15457448	1723	DYNC2H1	NM_001080463.2	c.9836C>A	p.(Ser3279Ter)	Stopgain	Likely Pathogenic	M	51	Any other ethnic group
2814615	1724	EFEMP1	NM_001039348.3	c.1033C>T	p.(Arg345Trp)	Missense	Likely Pathogenic	F	61	Any other ethnic group
5627131	1725	EFEMP1	NM_001039348.3	c.1033C>T	p.(Arg345Trp)	Missense	Likely Pathogenic	M	66	Not stated
6634445	1726	EFEMP1	NM_001039348.3	c.1033C>T	p.(Arg345Trp)	Missense	Likely Pathogenic	F	48	Not stated
1731015	1727	EFEMP1	NM_001039348.3	c.1033C>T	p.(Arg345Trp)	Missense	Likely Pathogenic	F	69	Not stated
3545296	1728	EFEMP1	NM_001039348.3	c.1033C>T	p.(Arg345Trp)	Missense	Likely Pathogenic	F	75	White - British
9642709	1728	EFEMP1	NM_001039348.3	c.1033C>T	p.(Arg345Trp)	Missense	Likely Pathogenic	M	72	Not stated
16928274	1728	EFEMP1	NM_001039348.3	c.1033C>T	p.(Arg345Trp)	Missense	Likely Pathogenic	F	41	White - British
7077594	1729	EFEMP1	NM_001039348.3	c.1033C>T	p.(Arg345Trp)	Missense	Likely Pathogenic	M	55	Not stated

6969444	1729	EFEMP1	NM_001039348.3	c.1033C>T	p.(Arg345Trp)	Missense	Likely Pathogenic	F	78	White - British
9539368	1730	EFEMP1	NM_001039348.3	c.1033C>T	p.(Arg345Trp)	Missense	Likely Pathogenic	F	56	White - British
10275166	1731	EFEMP1	NM_001039348.3	c.1033C>T	p.(Arg345Trp)	Missense	Likely Pathogenic	F	60	Not stated
7058771	1732	EFEMP1	NM_001039348.3	c.1033C>T	p.(Arg345Trp)	Missense	Likely Pathogenic	F	51	White - British
12152174	1733	EFEMP1	NM_001039348.3	c.1033C>T	p.(Arg345Trp)	Missense	Likely Pathogenic	F	53	Unknown
11855626	1734	EFEMP1	NM_001039348.3	c.1033C>T	p.(Arg345Trp)	Missense	Likely Pathogenic	F	55	Any other ethnic group
2732456	1735	EFEMP1	NM_001039348.3	c.1033C>T	p.(Arg345Trp)	Missense	Likely Pathogenic	F	78	Unknown
12940213	1735	EFEMP1	NM_001039348.3	c.1033C>T	p.(Arg345Trp)	Missense	Likely Pathogenic	F	54	Not stated
13091427	1735	EFEMP1	NM_001039348.3	c.1033C>T	p.(Arg345Trp)	Missense	Likely Pathogenic	F	31	Any other ethnic group
17056171	1735	EFEMP1	NM_001039348.3	c.1033C>T	p.(Arg345Trp)	Missense	Likely Pathogenic	M	52	Not stated
15142497	1736	EFEMP1	NM_001039348.3	c.1033C>T	p.(Arg345Trp)	Missense	Likely Pathogenic	F	70	White - British
13510153	1737	EFEMP1	NM_001039348.3	c.1033C>T	p.(Arg345Trp)	Missense	Likely Pathogenic	F	45	Unknown
15438506	1737	EFEMP1	NM_001039348.3	c.1033C>T	p.(Arg345Trp)	Missense	Likely Pathogenic	F	64	Not stated
16057600	1738	EFEMP1	NM_001039348.3	c.1033C>T	p.(Arg345Trp)	Missense	Likely Pathogenic	F	46	Not stated
16097073	1739	EFEMP1	NM_001039348.3	c.1033C>T	p.(Arg345Trp)	Missense	Likely Pathogenic	F	52	White - British
16081358	1740	EFEMP1	NM_001039348.3	c.1033C>T	p.(Arg345Trp)	Missense	Likely Pathogenic	F	59	Not stated
16330586	1741	EFEMP1	NM_001039348.3	c.1033C>T	p.(Arg345Trp)	Missense	Likely Pathogenic	F	42	Not stated
16421390	1742	EFEMP1	NM_001039348.3	c.1033C>T	p.(Arg345Trp)	Missense	Likely Pathogenic	M	41	Not stated
16778019	1743	EFEMP1	NM_001039348.3	c.1033C>T	p.(Arg345Trp)	Missense	Likely Pathogenic	F	69	White - British
17149754	1744	EFEMP1	NM_001039348.3	c.1033C>T	p.(Arg345Trp)	Missense	Likely Pathogenic	M	36	Not stated
17250092	1745	EFEMP1	NM_001039348.3	c.1033C>T	p.(Arg345Trp)	Missense	Likely Pathogenic	M	65	Not stated
17866533	1746	EFEMP1	NM_001039348.3	c.1033C>T	p.(Arg345Trp)	Missense	Likely Pathogenic	F	59	Not stated
17278750	1747	EFEMP1	NM_001039348.3	c.1033C>T	p.(Arg345Trp)	Missense	Likely Pathogenic	M	66	Not stated
17592525	1748	EFEMP1	NM_001039348.3	c.1033C>T	p.(Arg345Trp)	Missense	Likely Pathogenic	F	35	White - British
18425406	1749	EFEMP1	NM_001039348.3	c.1033C>T	p.(Arg345Trp)	Missense	Likely Pathogenic	F	41	Not stated
18605544	1749	EFEMP1	NM_001039348.3	c.1033C>T	p.(Arg345Trp)	Missense	Likely Pathogenic	F	64	Not stated
18583515	1750	EFEMP1	NM_001039348.3	c.1033C>T	p.(Arg345Trp)	Missense	Likely Pathogenic	F	58	Not stated
626674	1751	EYS	NM_001142800.2	c.892del	p.(Cys298ValfsTer41)	Frameshift	Likely Pathogenic	M	61	Not stated
626674	1751	EYS	NM_001142800.2	Exon 34 deletion	Deletion	Deletion	Likely Pathogenic	M	61	Not stated
1490957	1752	EYS	NM_001142800.2	c.490C>T	p.(Arg164Ter)	Stopgain	Pathogenic	M	61	Asian - Pakistani
1490957	1752	EYS	NM_001142800.2	c.490C>T	p.(Arg164Ter)	Stopgain	Pathogenic	M	61	Asian - Pakistani
2406291	1753	EYS	NM_001142800.2	c.3024C>A	p.(Cys1008Ter)	Stopgain	Pathogenic	F	78	Not stated
2406291	1753	EYS	NM_001142800.2	c.8122_8125delinsG	p.(Ser2708_Phe2709delinsVal)	Inframe indel	Variant of Uncertain Significance	F	78	Not stated
3591146	1754	EYS	NM_001142800.2	c.490C>T	p.(Arg164Ter)	Stopgain	Pathogenic	M	78	Asian - Indian
3591146	1754	EYS	NM_001142800.2	c.8133_8137del	p.(Phe2712CysfsTer33)	Frameshift	Pathogenic	M	78	Asian - Indian
3493069	1755	EYS	NM_001142800.2	c.4609A>T	p.(Arg1537Ter)	Stopgain	Likely Pathogenic	M	73	Not stated
3493069	1755	EYS	NM_001142800.2	c.4609A>T	p.(Arg1537Ter)	Stopgain	Likely Pathogenic	M	73	Not stated
5125637	1756	EYS	NM_001142800.2	c.3380_3381insATTAA	p.(Asn1127LysfsTer38)	Frameshift	Likely Pathogenic	F	62	White - British
5125637	1756	EYS	NM_001142800.2	c.3380_3381insATTAA	p.(Asn1127LysfsTer38)	Frameshift	Likely Pathogenic	F	62	White - British
9192889	1757	EYS	NM_001142800.2	c.3226T>C	p.(Cys1076Arg)	Missense	Variant of Uncertain Significance	M	45	Any other ethnic group
9192889	1757	EYS	NM_001142800.2	c.490C>T	p.(Arg164Ter)	Stopgain	Pathogenic	M	45	Any other ethnic group
5300875	1758	EYS	NM_001142800.2	c.7822C>T	p.(Gln2608Ter)	Stopgain	Pathogenic	F	64	White - British
5300875	1758	EYS	NM_001142800.2	c.9299_9302del	p.(Thr3100LysfsTer26)	Frameshift	Pathogenic	F	64	White - British
5359626	1759	EYS	NM_001142800.2	c.6323G>A	p.(Cys2108Tyr)	Missense	Pathogenic	M	59	White - British
5359626	1759	EYS	NM_001142800.2	c.6545del	p.(Asn2182ThrfsTer3)	Frameshift	Pathogenic	M	59	White - British
7208900	1760	EYS	NM_001142800.2	c.6416G>A	p.(Cys2139Tyr)	Missense	Pathogenic	M	70	Any other ethnic group
7208900	1760	EYS	NM_001142800.2	c.6416G>A	p.(Cys2139Tyr)	Missense	Pathogenic	M	70	Any other ethnic group
5745053	1761	EYS	NM_001142800.2	c.2260-3T>G	Splice	Splice	Variant of Uncertain Significance	F	58	White - British
5745053	1761	EYS	NM_001142800.2	c.6416G>A	p.(Cys2139Tyr)	Missense	Pathogenic	F	58	White - British
10364836	1762	EYS	NM_001142800.2	c.5408C>G	p.(Ser1803Ter)	Stopgain	Pathogenic	M	49	Black - African
10364836	1762	EYS	NM_001142800.2	c.5928-2A>G	Splice	Splice	Pathogenic	M	49	Black - African

6603022	1763	EYS	NM_001142800.2	c.2620C>T	p.(Gln874Ter)	Stopgain	Pathogenic	M	56	White - Irish
6603022	1763	EYS	NM_001142800.2	c.5317_5342delinsTA	p.(Asn1773Ter)	Stopgain	Pathogenic	M	56	White - Irish
7127630	1764	EYS	NM_001142800.2	Exon 32-33 deletion	Deletion	Deletion	Likely Pathogenic	F	68	Any other ethnic group
7127630	1764	EYS	NM_001142800.2	Exon 32-33 deletion	Deletion	Deletion	Likely Pathogenic	F	68	Any other ethnic group
7275358	1765	EYS	NM_001142800.2	c.6137G>A	p.(Trp2046Ter)	Stopgain	Pathogenic	F	58	White - British
7275358	1765	EYS	NM_001142800.2	c.9131G>T	p.(Trp3044Leu)	Missense	Variant of Uncertain Significance	F	58	White - British
6835303	1766	EYS	NM_001142800.2	c.2826_2827del	p.(Val944GlyfsTer9)	Frameshift	Pathogenic	M	47	Asian - Other
6835303	1766	EYS	NM_001142800.2	c.490C>T	p.(Arg164Ter)	Stopgain	Pathogenic	M	47	Asian - Other
7310785	1767	EYS	NM_001142800.2	c.490C>T	p.(Arg164Ter)	Stopgain	Pathogenic	F	50	White - Other
7310785	1767	EYS	NM_001142800.2	c.490C>T	p.(Arg164Ter)	Stopgain	Pathogenic	F	50	White - Other
10857622	1768	EYS	NM_001142800.2	c.490C>T	p.(Arg164Ter)	Stopgain	Pathogenic	F	40	Unknown
10857622	1768	EYS	NM_001142800.2	c.490C>T	p.(Arg164Ter)	Stopgain	Pathogenic	F	40	Unknown
8798082	1769	EYS	NM_001142800.2	c.1765A>G	p.(Arg589Gly)	Missense	Likely Pathogenic	F	41	xed - White and Black African
8798082	1769	EYS	NM_001142800.2	c.7665C>G	p.(Tyr2555Ter)	Stopgain	Likely Pathogenic	F	41	xed - White and Black African
5108417	1770	EYS	NM_001142800.2	c.490C>T	p.(Arg164Ter)	Stopgain	Pathogenic	F	62	Asian - Indian
5108417	1770	EYS	NM_001142800.2	c.7810C>T	p.(Arg2604Cys)	Missense	Variant of Uncertain Significance	F	62	Asian - Indian
9289573	1771	EYS	NM_001142800.2	c.1767-3C>G	Splice	Splice	Variant of Uncertain Significance	M	66	White - British
9289573	1771	EYS	NM_001142800.2	c.-448+5G>A	Splice	Splice	Variant of Uncertain Significance	M	66	White - British
9416259	1772	EYS	NM_001142800.2	c.6794del	p.(Pro2265GlnfsTer46)	Frameshift	Pathogenic	F	34	Not stated
9416259	1772	EYS	NM_001142800.2	c.9344T>A	p.(Val3115Asp)	Missense	Likely Pathogenic	F	34	Not stated
8245215	1772	EYS	NM_001142800.2	c.6794del	p.(Pro2265GlnfsTer46)	Frameshift	Pathogenic	M	35	ed - White and Black Caribbean
8245215	1772	EYS	NM_001142800.2	c.9344T>A	p.(Val3115Asp)	Missense	Likely Pathogenic	M	35	ed - White and Black Caribbean
8522058	1773	EYS	NM_001142800.2	c.1505_1506insGA	p.(Phe502LeufsTer14)	Frameshift	Likely Pathogenic	M	34	Not stated
8522058	1773	EYS	NM_001142800.2	c.9036del	p.(Leu3013SerfsTer6)	Frameshift	Pathogenic	M	34	Not stated
8666006	1774	EYS	NM_001142800.2	c.9354dup	p.(Gln3119SerfsTer7)	Frameshift	Pathogenic	M	60	Asian - Pakistani
8666006	1774	EYS	NM_001142800.2	c.9354dup	p.(Gln3119SerfsTer7)	Frameshift	Pathogenic	M	60	Asian - Pakistani
7278606	1774	EYS	NM_001142800.2	c.9354dup	p.(Gln3119SerfsTer7)	Frameshift	Pathogenic	F	58	Asian - Pakistani
7278606	1774	EYS	NM_001142800.2	c.9354dup	p.(Gln3119SerfsTer7)	Frameshift	Pathogenic	F	58	Asian - Pakistani
9843420	1775	EYS	NM_001142800.2	Exon 37 deletion	Deletion	Deletion	Variant of Uncertain Significance	M	66	Asian - Indian
9843420	1775	EYS	NM_001142800.2	Exon 37 deletion	Deletion	Deletion	Variant of Uncertain Significance	M	66	Asian - Indian
6627473	1776	EYS	NM_001142800.2	c.6794del	p.(Pro2265GlnfsTer46)	Frameshift	Pathogenic	M	71	Black - Caribbean
6627473	1776	EYS	NM_001142800.2	c.6794del	p.(Pro2265GlnfsTer46)	Frameshift	Pathogenic	M	71	Black - Caribbean
12368810	1777	EYS	NM_001142800.2	c.2976T>A	p.(Cys992Ter)	Stopgain	Pathogenic	M	70	Not stated
12368810	1777	EYS	NM_001142800.2	c.7994G>A	p.(Gly2665Glu)	Missense	Variant of Uncertain Significance	M	70	Not stated
8034942	1778	EYS	NM_001142800.2	c.2000G>A	p.(Arg667His)	Missense	Variant of Uncertain Significance	F	68	Black - Caribbean
8034942	1778	EYS	NM_001142800.2	c.2000G>A	p.(Arg667His)	Missense	Variant of Uncertain Significance	F	68	Black - Caribbean
8034942	1778	EYS	NM_001142800.2	c.977G>A	p.(Ser326Asn)	Missense	Benign	F	68	Black - Caribbean
8034942	1778	EYS	NM_001142800.2	c.977G>A	p.(Ser326Asn)	Missense	Benign	F	68	Black - Caribbean
8911090	1779	EYS	NM_001142800.2	c.5928-3_5928-1del	Splice	Splice	Pathogenic	F	62	Not stated
8911090	1779	EYS	NM_001142800.2	c.5928-3_5928-1del	Splice	Splice	Pathogenic	F	62	Not stated
12135234	1780	EYS	NM_001142800.2	c.1211del	p.(Asn404ThrfsTer17)	Frameshift	Pathogenic	F	36	Any other ethnic group
12135234	1780	EYS	NM_001142800.2	c.1211del	p.(Asn404ThrfsTer17)	Frameshift	Pathogenic	F	36	Any other ethnic group
10639810	1781	EYS	NM_001142800.2	c.613C>T	p.(Pro205Ser)	Missense	Variant of Uncertain Significance	M	58	White - British
10639810	1781	EYS	NM_001142800.2	c.6937C>T	p.(Gln2313Ter)	Stopgain	Pathogenic	M	58	White - British
10639810	1781	EYS	NM_001142800.2	c.8618A>G	p.(Asp2873Gly)	Missense	Variant of Uncertain Significance	M	58	White - British
13503832	1782	EYS	NM_001142800.2	c.5928-2A>G	Splice	Splice	Pathogenic	F	60	Not stated
13503832	1782	EYS	NM_001142800.2	c.5928-2A>G	Splice	Splice	Pathogenic	F	60	Not stated
16716461	1782	EYS	NM_001142800.2	c.5928-2A>G	Splice	Splice	Pathogenic	M	62	Any other ethnic group
16716461	1782	EYS	NM_001142800.2	c.5928-2A>G	Splice	Splice	Pathogenic	M	62	Any other ethnic group
13328671	1783	EYS	NM_001142800.2	c.3775C>T	p.(Gln1259Ter)	Stopgain	Pathogenic	M	35	Unknown
13328671	1783	EYS	NM_001142800.2	c.4045C>T	p.(Arg1349Ter)	Stopgain	Pathogenic	M	35	Unknown

14997233	1784	EYS	NM_001142800.2	c.988G>T	p.(Glu330Ter)	Stopgain	Pathogenic	F	56	Asian - Other
14997233	1784	EYS	NM_001142800.2	c.988G>T	p.(Glu330Ter)	Stopgain	Pathogenic	F	56	Asian - Other
14382395	1785	EYS	NM_001142800.2	c.6794del	p.(Pro2265GlnfsTer46)	Frameshift	Pathogenic	F	63	Black - Caribbean
14382395	1785	EYS	NM_001142800.2	c.8278C>T	p.(Arg2760Cys)	Missense	Variant of Uncertain Significance	F	63	Black - Caribbean
11652430	1786	EYS	NM_001142800.2	c.6192-1G>A	Splice	Splice	Likely Pathogenic	M	75	Any other ethnic group
11652430	1786	EYS	NM_001142800.2	c.967G>T	p.(Gly323Ter)	Stopgain	Pathogenic	M	75	Any other ethnic group
15581677	1787	EYS	NM_001142800.2	Exon 14 duplication	Duplication	Duplication	Likely Pathogenic	M	52	Asian - Other
15581677	1787	EYS	NM_001142800.2	c.8805C>A	p.(Tyr2935Ter)	Stopgain	Pathogenic	M	52	Asian - Other
13921200	1788	EYS	NM_001142800.2	c.4451G>A	p.(Trp1484Ter)	Stopgain	Pathogenic	F	52	Any other ethnic group
13921200	1788	EYS	NM_001142800.2	c.6794del	p.(Pro2265GlnfsTer46)	Frameshift	Pathogenic	F	52	Any other ethnic group
15483789	1789	EYS	NM_001142800.2	c.6192-1G>T	Splice	Splice	Pathogenic	M	62	Asian - Indian
15483789	1789	EYS	NM_001142800.2	c.7868G>A	p.(Gly2623Glu)	Missense	Likely Pathogenic	M	62	Asian - Indian
8644145	1790	EYS	NM_001142800.2	c.2620C>T	p.(Gln874Ter)	Stopgain	Pathogenic	M	87	White - Irish
8644145	1790	EYS	NM_001142800.2	c.9362_9365del	p.(Pro3121GlnfsTer5)	Frameshift	Likely Pathogenic	M	87	White - Irish
13016282	1791	EYS	NM_001142800.2	c.8309T>C	p.(Leu2770Pro)	Missense	Variant of Uncertain Significance	M	56	Asian - Other
13016282	1791	EYS	NM_001142800.2	c.8845G>A	p.(Glu2949Lys)	Missense	Variant of Uncertain Significance	M	56	Asian - Other
4401620	1792	EYS	NM_001142800.2	c.9299_9302del	p.(Thr3100LysfsTer26)	Frameshift	Pathogenic	M	68	Not stated
4401620	1792	EYS	NM_001142800.2	Exon 33 deletion	Deletion	Deletion	Likely Pathogenic	M	68	Not stated
16002454	1793	EYS	NM_001142800.2	c.490C>T	p.(Arg164Ter)	Stopgain	Pathogenic	F	44	Asian - Pakistani
16002454	1793	EYS	NM_001142800.2	c.490C>T	p.(Arg164Ter)	Stopgain	Pathogenic	F	44	Asian - Pakistani
15900933	1794	EYS	NM_001142800.2	c.1155T>A	p.(Cys385Ter)	Stopgain	Pathogenic	F	36	White - Other
15900933	1794	EYS	NM_001142800.2	Exon 37 deletion	Deletion	Deletion	Variant of Uncertain Significance	F	36	White - Other
3719666	1795	EYS	NM_001142800.2	c.7868G>A	p.(Gly2623Glu)	Missense	Likely Pathogenic	F	65	Asian - Bangladeshi
3719666	1795	EYS	NM_001142800.2	c.9354dup	p.(Gln3119SerfsTer7)	Frameshift	Pathogenic	F	65	Asian - Bangladeshi
14769530	1796	EYS	NM_001142800.2	c.3881del	p.(Pro1294GlnfsTer20)	Frameshift	Likely Pathogenic	F	53	Not stated
14769530	1796	EYS	NM_001142800.2	Exon 34-35 duplication	Duplication	Duplication	Likely Pathogenic	F	53	Not stated
17208757	1797	EYS	NM_001142800.2	c.5928-2A>G	Splice	Splice	Pathogenic	M	44	White - British
17208757	1797	EYS	NM_001142800.2	Exon 9-11 duplication	Duplication	Duplication	Variant of Uncertain Significance	M	44	White - British
15353603	1798	EYS	NM_001142800.2	c.4045C>T	p.(Arg1349Ter)	Stopgain	Pathogenic	F	64	White - Other
15353603	1798	EYS	NM_001142800.2	c.4045C>T	p.(Arg1349Ter)	Stopgain	Pathogenic	F	64	White - Other
17369813	1799	EYS	NM_001142800.2	c.9354dup	p.(Gln3119SerfsTer7)	Frameshift	Pathogenic	M	45	Asian - Pakistani
17369813	1799	EYS	NM_001142800.2	c.9354dup	p.(Gln3119SerfsTer7)	Frameshift	Pathogenic	M	45	Asian - Pakistani
12280512	1800	EYS	NM_001142800.2	c.533dup	p.(Ser179GlufsTer10)	Frameshift	Pathogenic	M	45	Not stated
12280512	1800	EYS	NM_001142800.2	c.533dup	p.(Ser179GlufsTer10)	Frameshift	Pathogenic	M	45	Not stated
17544288	1801	EYS	NM_001142800.2	Exon 13-15 deletion	Deletion	Deletion	Likely Pathogenic	M	87	Unknown
17544288	1801	EYS	NM_001142800.2	Exon 13-15 deletion	Deletion	Deletion	Likely Pathogenic	M	87	Unknown
16845653	1802	EYS	NM_001142800.2	c.3443+1G>T	Splice	Splice	Pathogenic	M	29	Black - African
16845653	1802	EYS	NM_001142800.2	c.3443+1G>T	Splice	Splice	Pathogenic	M	29	Black - African
17972408	1803	EYS	NM_001142800.2	c.7187G>C	p.(Cys2396Ser)	Missense	Likely Pathogenic	M	58	Any other ethnic group
17972408	1803	EYS	NM_001142800.2	c.7187G>C	p.(Cys2396Ser)	Missense	Likely Pathogenic	M	58	Any other ethnic group
18189898	1804	EYS	NM_001142800.2	c.8107G>T	p.(Glu2703Ter)	Stopgain	Pathogenic	M	37	Not stated
18189898	1804	EYS	NM_001142800.2	Exon 14-22 deletion	Deletion	Deletion	Likely Pathogenic	M	37	Not stated
14817802	1805	EYS	NM_001142800.2	c.9354dup	p.(Gln3119SerfsTer7)	Frameshift	Pathogenic	M	41	Asian - Pakistani
14817802	1805	EYS	NM_001142800.2	c.9354dup	p.(Gln3119SerfsTer7)	Frameshift	Pathogenic	M	41	Asian - Pakistani
4584467	1806	EYS	NM_001142800.2	c.8830del	p.(Val2944TrpfsTer31)	Frameshift	Pathogenic	M	60	Asian - Indian
4584467	1806	EYS	NM_001142800.2	Exon 34 deletion	Deletion	Deletion	Likely Pathogenic	M	60	Asian - Indian
18271385	1807	EYS	NM_001142800.2	c.35T>C	p.(Met12Thr)	Missense	Variant of Uncertain Significance	M	35	Asian - Other
18271385	1807	EYS	NM_001142800.2	c.490C>T	p.(Arg164Ter)	Stopgain	Pathogenic	M	35	Asian - Other
8026031	1808	EYS	NM_001142800.2	c.9380_9383del	p.(Ile3127AsnfsTer2)	Frameshift	Likely Pathogenic	M	62	Not stated
8026031	1808	EYS	NM_001142800.2	c.9383_9387del	p.(Lys3128ArgfsTer7)	Frameshift	Pathogenic	M	62	Not stated
17991546	1809	EYS	NM_001142800.2	c.4350_4356del	p.(Ile1451ProfsTer3)	Frameshift	Pathogenic	M	53	Not stated

17991546	1809	EYS	NM_001142800.2	Exon 16-19 deletion	Deletion	Deletion	Likely Pathogenic	M	53	Not stated
17897585	1810	EYS	NM_001142800.2	c.2461G>T	p.(Gly821Ter)	Stopgain	Pathogenic	M	27	White - Other
17897585	1810	EYS	NM_001142800.2	c.4392_4393del	p.(Ala1465SerfsTer5)	Frameshift	Likely Pathogenic	M	27	White - Other
2792635	1811	EYS	NM_001142800.2	c.4152dup	p.(Pro1385SerfsTer2)	Frameshift	Pathogenic	F	69	Not stated
2792635	1811	EYS	NM_001142800.2	c.9071T>G	p.(Leu3024Trp)	Missense	Variant of Uncertain Significance	F	69	Not stated
6769132	1812	EYS	NM_001142800.2	c.4943C>A	p.(Ser1648Tyr)	Missense	Variant of Uncertain Significance	F	43	Black - African
6769132	1812	EYS	NM_001142800.2	c.746C>G	p.(Thr249Arg)	Missense	Variant of Uncertain Significance	F	43	Black - African
4610542	1813	EYS	NM_001142800.2	c.2992G>A	p.(Gly998Ser)	Missense	Likely Pathogenic	M	64	Any other ethnic group
4610542	1813	EYS	NM_001142800.2	c.704G>A	p.(Trp235Ter)	Stopgain	Likely Pathogenic	M	64	Any other ethnic group
4486621	1814	FAM161A	NM_001201543.2	c.1309A>T	p.(Arg437Ter)	Stopgain	Pathogenic	M	58	Any other ethnic group
4486621	1814	FAM161A	NM_001201543.2	c.1309A>T	p.(Arg437Ter)	Stopgain	Pathogenic	M	58	Any other ethnic group
10508287	1815	FAM161A	NM_001201543.2	c.782del	p.(Asp261ValfsTer39)	Frameshift	Pathogenic	M	41	Not stated
10508287	1815	FAM161A	NM_001201543.2	c.782del	p.(Asp261ValfsTer39)	Frameshift	Pathogenic	M	41	Not stated
3485117	1816	FAM161A	NM_001201543.2	c.1309A>T	p.(Arg437Ter)	Stopgain	Pathogenic	F	63	Unknown
3485117	1816	FAM161A	NM_001201543.2	c.1309A>T	p.(Arg437Ter)	Stopgain	Pathogenic	F	63	Unknown
1798159	1817	FAM161A	NM_001201543.2	c.1309A>T	p.(Arg437Ter)	Stopgain	Pathogenic	F	51	Not stated
1798159	1817	FAM161A	NM_001201543.2	c.1309A>T	p.(Arg437Ter)	Stopgain	Pathogenic	F	51	Not stated
1798145	1817	FAM161A	NM_001201543.2	c.1309A>T	p.(Arg437Ter)	Stopgain	Pathogenic	F	57	Not stated
1798145	1817	FAM161A	NM_001201543.2	c.1309A>T	p.(Arg437Ter)	Stopgain	Pathogenic	F	57	Not stated
11636001	1818	FAM161A	NM_001201543.2	c.678_681del	p.(Lys227AsnfsTer17)	Frameshift	Pathogenic	M	55	Any other ethnic group
11636001	1818	FAM161A	NM_001201543.2	c.678_681del	p.(Lys227AsnfsTer17)	Frameshift	Pathogenic	M	55	Any other ethnic group
13374626	1819	FAM161A	NM_001201543.2	c.1309A>T	p.(Arg437Ter)	Stopgain	Pathogenic	M	27	Not stated
13374626	1819	FAM161A	NM_001201543.2	c.1355_1356del	p.(Thr452SerfsTer3)	Frameshift	Pathogenic	M	27	Not stated
12698671	1820	FAM161A	NM_001201543.2	c.1309A>T	p.(Arg437Ter)	Stopgain	Pathogenic	F	51	Unknown
12698671	1820	FAM161A	NM_001201543.2	c.1830del	p.(Leu611TyrfsTer22)	Frameshift	Pathogenic	F	51	Unknown
9574935	1821	FAM161A	NM_001201543.2	c.1309A>T	p.(Arg437Ter)	Stopgain	Pathogenic	F	47	Not stated
9574935	1821	FAM161A	NM_001201543.2	c.1584-1G>A	Splice	Splice	Likely Pathogenic	F	47	Not stated
5479480	1822	FLVCR1	NM_014053.4	c.1092+5G>A	Splice	Splice	Pathogenic	M	53	White - British
5479480	1822	FLVCR1	NM_014053.4	c.1413G>A	p.(Gln471Gln)	Synonymous	Variant of Uncertain Significance	M	53	White - British
6175623	1823	FLVCR1	NM_014053.4	c.1092+5G>A	Splice	Splice	Pathogenic	F	54	Any other ethnic group
6175623	1823	FLVCR1	NM_014053.4	c.1092+5G>A	Splice	Splice	Pathogenic	F	54	Any other ethnic group
5282962	1824	FLVCR1	NM_014053.4	c.323T>C	p.(Phe108Ser)	Missense	Variant of Uncertain Significance	F	54	Asian - Indian
5282962	1824	FLVCR1	NM_014053.4	c.323T>C	p.(Phe108Ser)	Missense	Variant of Uncertain Significance	F	54	Asian - Indian
15601256	1825	FOXC1	NM_001453.3	c.400G>T	p.(Glu134Ter)	Stopgain	Likely Pathogenic	F	8	Not stated
5192767	1825	FOXC1	NM_001453.3	c.400G>T	p.(Glu134Ter)	Stopgain	Likely Pathogenic	M	38	Not stated
17831883	1826	FOXE3	NM_012186.3	c.763dup	p.(Ala255GlyfsTer30)	Frameshift	Likely Pathogenic	F	5	Not stated
17831883	1826	FOXE3	NM_012186.3	c.763dup	p.(Ala255GlyfsTer30)	Frameshift	Likely Pathogenic	F	5	Not stated
17896591	1826	FOXE3	NM_012186.3	c.763dup	p.(Ala255GlyfsTer30)	Frameshift	Likely Pathogenic	M	5	Asian - Other
17896591	1826	FOXE3	NM_012186.3	c.763dup	p.(Ala255GlyfsTer30)	Frameshift	Likely Pathogenic	M	5	Asian - Other
499876	1827	FRMD7	NM_194277.3	c.206-5T>A	Splice	Splice	Variant of Uncertain Significance	F	50	White - British
13445949	1828	FRMD7	NM_194277.3	c.875T>C	p.(Leu292Pro)	Missense	Likely Pathogenic	F	44	Mixed - Other
17817162	1829	FRMD7	NM_194277.3	c.796G>C	p.(Ala266Pro)	Missense	Variant of Uncertain Significance	M	6	Not stated
17944653	1830	FRMD7	NM_194277.3	c.706_707del	p.(Lys236AlafsTer66)	Frameshift	Pathogenic	M	6	Not stated
11499396	1831	FZD4	NM_012193.4	c.1513C>T	p.(Gln505Ter)	Stopgain	Pathogenic	F	14	White - British
350832	1831	FZD4	NM_012193.4	c.1513C>T	p.(Gln505Ter)	Stopgain	Pathogenic	F	46	White - British
13773983	1831	FZD4	NM_012193.4	c.1513C>T	p.(Gln505Ter)	Stopgain	Pathogenic	F	10	White - British
10770192	1832	FZD4	NM_012193.4	c.1282_1285del	p.(Asp428SerfsTer2)	Frameshift	Pathogenic	M	15	White - British
17655091	1833	FZD4	NM_012193.4	c.169G>T	p.(Gly57Cys)	Missense	Pathogenic	M	5	White - British
11406562	1834	GNAT2	NM_005272.5	c.139A>G	p.(Ser47Gly)	Missense	Variant of Uncertain Significance	M	35	Not stated
11406562	1834	GNAT2	NM_005272.5	c.139A>G	p.(Ser47Gly)	Missense	Variant of Uncertain Significance	M	35	Not stated
9986927	1835	GPR179	NM_001004334.4	c.349G>T	p.(Asp117Tyr)	Missense	Variant of Uncertain Significance	F	16	White - Other

9986927	1835	GPR179	NM_001004334.4	c.349G>T	p.(Asp117Tyr)	Missense	Variant of Uncertain Significance	F	16	White - Other
11710047	1836	GPR179	NM_001004334.4	c.984del	p.(Ser329LeufsTer4)	Frameshift	Pathogenic	M	13	Any other ethnic group
11710047	1836	GPR179	NM_001004334.4	c.984del	p.(Ser329LeufsTer4)	Frameshift	Pathogenic	M	13	Any other ethnic group
15190335	1837	GRK1	NM_002929.3	c.1612G>A	p.(Gly538Ser)	Missense	Variant of Uncertain Significance	F	49	Asian - Pakistani
15190335	1837	GRK1	NM_002929.3	c.55C>T	p.(Arg19Ter)	Stopgain	Pathogenic	F	49	Asian - Pakistani
9394552	1838	GRM6	NM_000843.4	c.577del	p.(Val193TrpfsTer16)	Frameshift	Pathogenic	F	20	Asian - Bangladeshi
9394552	1838	GRM6	NM_000843.4	c.577del	p.(Val193TrpfsTer16)	Frameshift	Pathogenic	F	20	Asian - Bangladeshi
277850	1839	GRM6	NM_000843.4	c.118_132del	p.(Thr40_Leu44del)	Inframe indel	Likely Pathogenic	M	59	White - British
277850	1839	GRM6	NM_000843.4	c.137C>T	p.(Pro46Leu)	Missense	Pathogenic	M	59	White - British
10525871	1840	GRM6	NM_000843.4	c.1861C>T	p.(Arg621Ter)	Stopgain	Pathogenic	F	21	Any other ethnic group
10525871	1840	GRM6	NM_000843.4	c.1861C>T	p.(Arg621Ter)	Stopgain	Pathogenic	F	21	Any other ethnic group
15236346	1841	GRM6	NM_000843.4	c.2030G>A	p.(Arg677His)	Missense	Likely Pathogenic	M	12	White - Other
15236346	1841	GRM6	NM_000843.4	c.2030G>A	p.(Arg677His)	Missense	Likely Pathogenic	M	12	White - Other
16504900	1842	GRM6	NM_000843.4	c.1026C>A	p.(Tyr342Ter)	Stopgain	Likely Pathogenic	M	33	Not stated
16504900	1842	GRM6	NM_000843.4	c.2267G>A	p.(Gly756Asp)	Missense	Variant of Uncertain Significance	M	33	Not stated
17532934	1843	GRM6	NM_000843.4	c.58_72del	p.(Trp20_Ala24del)	Inframe indel	Variant of Uncertain Significance	F	54	Any other ethnic group
12617093	1844	GRM6	NM_000843.4	c.2155del	p.(Arg719GlyfsTer6)	Frameshift	Likely Pathogenic	M	38	Any other ethnic group
12617093	1844	GRM6	NM_000843.4	c.2155del	p.(Arg719GlyfsTer6)	Frameshift	Likely Pathogenic	M	38	Any other ethnic group
15672236	1845	GRM6	NM_000843.4	c.1605del	p.(Cys536AlafsTer27)	Frameshift	Likely Pathogenic	M	10	Any other ethnic group
15672236	1845	GRM6	NM_000843.4	c.1605del	p.(Cys536AlafsTer27)	Frameshift	Likely Pathogenic	M	10	Any other ethnic group
5094088	1846	GUCA1A	NM_000409.5	c.296A>G	p.(Tyr99Cys)	Missense	Pathogenic	F	54	Not stated
11278420	1846	GUCA1A	NM_000409.5	c.296A>G	p.(Tyr99Cys)	Missense	Pathogenic	M	29	White - British
5402928	1847	GUCA1A	NM_000409.5	c.296A>G	p.(Tyr99Cys)	Missense	Pathogenic	F	58	White - British
4903037	1848	GUCA1A	NM_000409.5	c.118C>T	p.(Arg40Cys)	Missense	Likely Benign	M	71	White - British
4903037	1848	GUCA1A	NM_000409.5	c.296A>G	p.(Tyr99Cys)	Missense	Pathogenic	M	71	White - British
7191743	1849	GUCA1A	NM_000409.5	c.296A>G	p.(Tyr99Cys)	Missense	Pathogenic	F	71	White - British
3027555	1850	GUCA1A	NM_000409.5	c.250C>T	p.(Leu84Phe)	Missense	Likely Pathogenic	F	38	Mixed - Other
3027506	1850	GUCA1A	NM_000409.5	c.250C>T	p.(Leu84Phe)	Missense	Likely Pathogenic	M	42	Not stated
732661	1850	GUCA1A	NM_000409.5	c.250C>T	p.(Leu84Phe)	Missense	Likely Pathogenic	M	70	White - Other
7491525	1851	GUCA1A	NM_000409.5	c.296A>G	p.(Tyr99Cys)	Missense	Pathogenic	M	59	White - British
11550118	1852	GUCA1A	NM_000409.5	c.296A>G	p.(Tyr99Cys)	Missense	Pathogenic	M	64	Not stated
3674397	1853	GUCA1A	NM_000409.5	c.332A>C	p.(Glu111Ala)	Missense	Likely Pathogenic	M	56	Unknown
4804834	1853	GUCA1A	NM_000409.5	c.332A>C	p.(Glu111Ala)	Missense	Likely Pathogenic	M	53	Not stated
2698905	1853	GUCA1A	NM_000409.5	c.332A>C	p.(Glu111Ala)	Missense	Likely Pathogenic	M	93	Unknown
13716723	1854	GUCA1A	NM_000409.5	c.296A>G	p.(Tyr99Cys)	Missense	Pathogenic	M	61	Unknown
15227827	1855	GUCA1A	NM_000409.5	c.296A>G	p.(Tyr99Cys)	Missense	Pathogenic	M	41	White - British
15757251	1856	GUCA1A	NM_000409.5	c.296A>G	p.(Tyr99Cys)	Missense	Pathogenic	M	34	White - British
14053423	1857	GUCA1A	NM_000409.5	c.320T>C	p.(Ile107Thr)	Missense	Pathogenic	F	73	White - British
17237506	1858	GUCA1A	NM_000409.5	c.296A>G	p.(Tyr99Cys)	Missense	Pathogenic	F	57	Not stated
10356030	1859	GUCY2D	NM_000180.4	c.652del	p.(Met218TrpfsTer13)	Frameshift	Likely Pathogenic	M	33	Not stated
10356030	1859	GUCY2D	NM_000180.4	c.652del	p.(Met218TrpfsTer13)	Frameshift	Likely Pathogenic	M	33	Not stated
2695629	1860	GUCY2D	NM_000180.4	c.2511_2512delinsCA	p.(Glu837_Arg838delinsAspSer)	Inframe indel	Variant of Uncertain Significance	M	62	Not stated
5441358	1860	GUCY2D	NM_000180.4	c.2511_2512delinsCA	p.(Glu837_Arg838delinsAspSer)	Inframe indel	Variant of Uncertain Significance	M	86	Not stated
17371605	1861	GUCY2D	NM_000180.4	c.2512C>T	p.(Arg838Cys)	Missense	Pathogenic	F	17	Not stated
6337610	1862	GUCY2D	NM_000180.4	c.2513G>A	p.(Arg838His)	Missense	Pathogenic	F	34	Not stated
4196359	1862	GUCY2D	NM_000180.4	c.2513G>A	p.(Arg838His)	Missense	Pathogenic	F	57	Not stated
4290621	1862	GUCY2D	NM_000180.4	c.2513G>A	p.(Arg838His)	Missense	Pathogenic	F	57	Not stated
4447589	1863	GUCY2D	NM_000180.4	c.2513G>A	p.(Arg838His)	Missense	Pathogenic	M	58	Not stated
6782	1864	GUCY2D	NM_000180.4	c.238_252del	p.(Ala80_Leu84del)	Inframe indel	Pathogenic	F	60	Not stated
6782	1864	GUCY2D	NM_000180.4	c.307G>A	p.(Glu103Lys)	Missense	Pathogenic	F	60	Not stated
15317483	1865	GUCY2D	NM_000180.4	c.2512C>T	p.(Arg838Cys)	Missense	Pathogenic	M	41	Any other ethnic group

1248652	1866	GUCY2D	NM_000180.4	c.2348T>C	p.(Leu783Pro)	Missense	Variant of Uncertain Significance	F	46	Not stated
1248652	1866	GUCY2D	NM_000180.4	c.2384G>A	p.(Arg795Gln)	Missense	Likely Pathogenic	F	46	Not stated
7128141	1867	GUCY2D	NM_000180.4	c.2741C>T	p.(Ala914Val)	Missense	Variant of Uncertain Significance	M	72	White - British
6627431	1868	GUCY2D	NM_000180.4	c.2512C>T	p.(Arg838Cys)	Missense	Pathogenic	F	59	White - British
13824992	1869	GUCY2D	NM_000180.4	c.2513G>A	p.(Arg838His)	Missense	Pathogenic	F	14	Not stated
16182018	1869	GUCY2D	NM_000180.4	c.2513G>A	p.(Arg838His)	Missense	Pathogenic	F	74	Not stated
7305388	1870	GUCY2D	NM_000180.4	c.2512C>T	p.(Arg838Cys)	Missense	Pathogenic	F	62	White - British
7334403	1871	GUCY2D	NM_000180.4	c.3098_3099insCGTGCTCT	p.(Gly1034ValfsTer15)	Frameshift	Pathogenic	F	21	Asian - Indian
7334403	1871	GUCY2D	NM_000180.4	c.3098_3099insCGTGCTCT	p.(Gly1034ValfsTer15)	Frameshift	Pathogenic	F	21	Asian - Indian
3248902	1872	GUCY2D	NM_000180.4	c.2513G>A	p.(Arg838His)	Missense	Pathogenic	M	55	Not stated
8800966	1873	GUCY2D	NM_000180.4	c.1978C>T	p.(Arg660Ter)	Stopgain	Pathogenic	F	19	Not stated
8800966	1873	GUCY2D	NM_000180.4	c.2302C>T	p.(Arg768Trp)	Missense	Pathogenic	F	19	Not stated
9447878	1874	GUCY2D	NM_000180.4	c.1343C>A	p.(Ser448Ter)	Stopgain	Pathogenic	M	34	White - British
9447878	1874	GUCY2D	NM_000180.4	c.1958del	p.(Gly653GlufsTer2)	Frameshift	Likely Pathogenic	M	34	White - British
8255645	1875	GUCY2D	NM_000180.4	c.2123G>C	p.(Trp708Ser)	Missense	Variant of Uncertain Significance	F	19	White - British
8255645	1875	GUCY2D	NM_000180.4	c.2123G>C	p.(Trp708Ser)	Missense	Variant of Uncertain Significance	F	19	White - British
11822313	1876	GUCY2D	NM_000180.4	c.2291del	p.(Pro764LeufsTer20)	Frameshift	Likely Pathogenic	M	23	White - Other
11822313	1876	GUCY2D	NM_000180.4	c.2944+1del	Splice	Splice	Pathogenic	M	23	White - Other
10302739	1877	GUCY2D	NM_000180.4	c.1759C>G	p.(Leu587Val)	Missense	Variant of Uncertain Significance	F	32	White - British
10302739	1877	GUCY2D	NM_000180.4	c.2302C>T	p.(Arg768Trp)	Missense	Pathogenic	F	32	White - British
11014590	1878	GUCY2D	NM_000180.4	c.2858C>T	p.(Ser953Leu)	Missense	Likely Pathogenic	M	24	Not stated
11014590	1878	GUCY2D	NM_000180.4	c.2944+1del	Splice	Splice	Pathogenic	M	24	Not stated
11134458	1879	GUCY2D	NM_000180.4	c.2512C>T	p.(Arg838Cys)	Missense	Pathogenic	F	67	Unknown
4514964	1880	GUCY2D	NM_000180.4	c.1762C>T	p.(Arg588Trp)	Missense	Variant of Uncertain Significance	F	68	White - British
4514964	1880	GUCY2D	NM_000180.4	c.307G>A	p.(Glu103Lys)	Missense	Pathogenic	F	68	White - British
9890068	1881	GUCY2D	NM_000180.4	c.3056A>C	p.(His1019Pro)	Missense	Likely Pathogenic	M	16	Asian - Pakistani
9890068	1881	GUCY2D	NM_000180.4	c.3056A>C	p.(His1019Pro)	Missense	Likely Pathogenic	M	16	Asian - Pakistani
12163969	1882	GUCY2D	NM_000180.4	c.2872A>C	p.(Ser958Arg)	Missense	Likely Pathogenic	M	17	Unknown
12163969	1882	GUCY2D	NM_000180.4	c.307G>A	p.(Glu103Lys)	Missense	Pathogenic	M	17	Unknown
10064270	1883	GUCY2D	NM_000180.4	c.2513G>A	p.(Arg838His)	Missense	Pathogenic	M	46	Not stated
13159572	1884	GUCY2D	NM_000180.4	c.2704G>A	p.(Val902Met)	Missense	Likely Pathogenic	M	37	Not stated
15615746	1884	GUCY2D	NM_000180.4	c.2704G>A	p.(Val902Met)	Missense	Likely Pathogenic	M	8	Not stated
11093235	1885	GUCY2D	NM_000180.4	c.2513G>A	p.(Arg838His)	Missense	Pathogenic	M	45	Unknown
1894591	1886	GUCY2D	NM_000180.4	c.2512C>T	p.(Arg838Cys)	Missense	Pathogenic	M	68	Unknown
16632699	1886	GUCY2D	NM_000180.4	c.2512C>T	p.(Arg838Cys)	Missense	Pathogenic	M	30	Not stated
15148489	1887	GUCY2D	NM_000180.4	c.2513G>A	p.(Arg838His)	Missense	Pathogenic	M	44	Unknown
15430988	1888	GUCY2D	NM_000180.4	c.2837C>A	p.(Ala946Glu)	Missense	Likely Pathogenic	M	9	Unknown
15430988	1888	GUCY2D	NM_000180.4	c.2969G>T	p.(Gly990Val)	Missense	Variant of Uncertain Significance	M	9	Unknown
15378250	1889	GUCY2D	NM_000180.4	c.2512C>T	p.(Arg838Cys)	Missense	Pathogenic	M	45	Not stated
15432269	1890	GUCY2D	NM_000180.4	c.2512C>T	p.(Arg838Cys)	Missense	Pathogenic	F	20	Unknown
15980397	1891	GUCY2D	NM_000180.4	c.2512C>T	p.(Arg838Cys)	Missense	Pathogenic	F	42	White - British
3024363	1892	GUCY2D	NM_000180.4	c.2512C>T	p.(Arg838Cys)	Missense	Pathogenic	M	71	Not stated
15721411	1893	GUCY2D	NM_000180.4	c.1694T>C	p.(Phe565Ser)	Missense	Likely Pathogenic	M	8	Any other ethnic group
15721411	1893	GUCY2D	NM_000180.4	c.1694T>C	p.(Phe565Ser)	Missense	Likely Pathogenic	M	8	Any other ethnic group
15721411	1893	GUCY2D	NM_000180.4	c.2633_2636del	p.(Gln878ArgfsTer17)	Frameshift	Likely Pathogenic	M	8	Any other ethnic group
15721411	1893	GUCY2D	NM_000180.4	c.2633_2636del	p.(Gln878ArgfsTer17)	Frameshift	Likely Pathogenic	M	8	Any other ethnic group
16176551	1894	GUCY2D	NM_000180.4	c.3044-2A>G	Splice	Splice	Likely Pathogenic	F	7	Not stated
16176551	1894	GUCY2D	NM_000180.4	c.3044-2A>G	Splice	Splice	Likely Pathogenic	F	7	Not stated
16441851	1895	GUCY2D	NM_000180.4	c.2948C>T	p.(Pro983Leu)	Missense	Variant of Uncertain Significance	F	62	White - British
16441851	1895	GUCY2D	NM_000180.4	c.307G>A	p.(Glu103Lys)	Missense	Pathogenic	F	62	White - British
7142169	1896	GUCY2D	NM_000180.4	c.1664A>C	p.(Tyr555Ser)	Missense	Variant of Uncertain Significance	M	47	White - British

7142169	1896	GUCY2D	NM_000180.4	c.2302C>T	p.(Arg768Trp)	Missense	Pathogenic	M	47	White - British
18308716	1897	GUCY2D	NM_000180.4	c.2512C>A	p.(Arg838Ser)	Missense	Pathogenic	M	30	Unknown
18337038	1898	GUCY2D	NM_000180.4	c.2302C>T	p.(Arg768Trp)	Missense	Pathogenic	F	5	Not stated
18337038	1898	GUCY2D	NM_000180.4	c.2303G>A	p.(Arg768Gln)	Missense	Pathogenic	F	5	Not stated
3789946	1899	HGSNAT	NM_152419.3	c.1283T>G	p.(Phe428Cys)	Missense	Variant of Uncertain Significance	F	85	Asian - Pakistani
3789946	1899	HGSNAT	NM_152419.3	c.1283T>G	p.(Phe428Cys)	Missense	Variant of Uncertain Significance	F	85	Asian - Pakistani
6495600	1900	HGSNAT	NM_152419.3	c.1708del	p.(Thr570ProfsTer8)	Frameshift	Pathogenic	M	56	Not stated
6495600	1900	HGSNAT	NM_152419.3	c.1843G>A	p.(Ala615Thr)	Missense	Variant of Uncertain Significance	M	56	Not stated
12982024	1901	HGSNAT	NM_152419.3	c.1542+4dup	Splice	Splice	Likely Pathogenic	F	62	White - Other
12982024	1901	HGSNAT	NM_152419.3	c.1843G>A	p.(Ala615Thr)	Missense	Variant of Uncertain Significance	F	62	White - Other
4617605	1902	HGSNAT	NM_152419.3	c.1843G>A	p.(Ala615Thr)	Missense	Variant of Uncertain Significance	F	69	White - British
4617605	1902	HGSNAT	NM_152419.3	c.848C>T	p.(Pro283Leu)	Missense	Pathogenic	F	69	White - British
9121580	1903	HGSNAT	NM_152419.3	c.1252G>C	p.(Gly418Arg)	Missense	Likely Pathogenic	M	53	White - British
9121580	1903	HGSNAT	NM_152419.3	c.1843G>A	p.(Ala615Thr)	Missense	Variant of Uncertain Significance	M	53	White - British
3563020	1904	HGSNAT	NM_152419.3	c.1543-2A>C	Splice	Splice	Pathogenic	M	86	White - British
3563020	1904	HGSNAT	NM_152419.3	c.1843G>A	p.(Ala615Thr)	Missense	Variant of Uncertain Significance	M	86	White - British
13726257	1905	HGSNAT	NM_152419.3	c.743G>C	p.(Gly248Ala)	Missense	Likely Pathogenic	M	58	Not stated
13726257	1905	HGSNAT	NM_152419.3	c.887C>T	p.(Ser296Leu)	Missense	Variant of Uncertain Significance	M	58	Not stated
13814135	1906	HGSNAT	NM_152419.3	c.1250+1G>A	Splice	Splice	Pathogenic	M	64	Not stated
13814135	1906	HGSNAT	NM_152419.3	c.1843G>A	p.(Ala615Thr)	Missense	Variant of Uncertain Significance	M	64	Not stated
5768657	1907	HGSNAT	NM_152419.3	c.887C>T	p.(Ser296Leu)	Missense	Variant of Uncertain Significance	M	81	Asian - Pakistani
5768657	1907	HGSNAT	NM_152419.3	c.887C>T	p.(Ser296Leu)	Missense	Variant of Uncertain Significance	M	81	Asian - Pakistani
5633907	1908	PRPF3	NM_004698.4	c.1481C>T	p.(Thr494Met)	Missense	Pathogenic	F	89	White - British
8391718	1909	PRPF3	NM_004698.4	c.1481C>T	p.(Thr494Met)	Missense	Pathogenic	M	55	White - British
12683096	1909	PRPF3	NM_004698.4	c.1481C>T	p.(Thr494Met)	Missense	Pathogenic	M	28	Unknown
6587587	1910	PRPF3	NM_004698.4	c.1499T>G	p.(Val500Gly)	Missense	Likely Pathogenic	F	61	Not stated
12360109	1911	PRPF3	NM_004698.4	c.1481C>T	p.(Thr494Met)	Missense	Pathogenic	M	20	Asian - Other
17572939	1912	PRPF3	NM_004698.4	c.1481C>T	p.(Thr494Met)	Missense	Pathogenic	F	30	Not stated
265	1913	IFT140	NM_014714.4	c.1246C>T	p.(Gln416Ter)	Stopgain	Likely Pathogenic	F	69	Not stated
265	1913	IFT140	NM_014714.4	c.212C>T	p.(Pro71Leu)	Missense	Likely Pathogenic	F	69	Not stated
11618207	1914	IFT140	NM_014714.4	c.998G>A	p.(Cys333Tyr)	Missense	Likely Pathogenic	F	66	Any other ethnic group
11618207	1914	IFT140	NM_014714.4	c.998G>A	p.(Cys333Tyr)	Missense	Likely Pathogenic	F	66	Any other ethnic group
11877081	1914	IFT140	NM_014714.4	c.998G>A	p.(Cys333Tyr)	Missense	Likely Pathogenic	M	50	Unknown
11877081	1914	IFT140	NM_014714.4	c.998G>A	p.(Cys333Tyr)	Missense	Likely Pathogenic	M	50	Unknown
12788516	1915	IFT140	NM_014714.4	c.998G>A	p.(Cys333Tyr)	Missense	Likely Pathogenic	M	35	Not stated
12788516	1915	IFT140	NM_014714.4	c.998G>A	p.(Cys333Tyr)	Missense	Likely Pathogenic	M	35	Not stated
7401302	1916	IFT140	NM_014714.4	c.1319T>C	p.(Leu440Pro)	Missense	Likely Pathogenic	M	93	White - British
7401302	1916	IFT140	NM_014714.4	c.2611C>T	p.(Arg871Cys)	Missense	Likely Pathogenic	M	93	White - British
16981313	1917	IFT140	NM_014714.4	c.2611C>T	p.(Arg871Cys)	Missense	Likely Pathogenic	M	39	White - British
16981313	1917	IFT140	NM_014714.4	c.634G>A	p.(Gly212Arg)	Missense	Likely Pathogenic	M	39	White - British
15738232	1918	IFT140	NM_014714.4	c.2399+1G>T	Splice	Splice	Pathogenic	F	51	Any other ethnic group
15738232	1918	IFT140	NM_014714.4	c.4196T>C	p.(Leu1399Pro)	Missense	Variant of Uncertain Significance	F	51	Any other ethnic group
292543	1919	IMPDH1	NM_000883.4	c.931G>A	p.(Asp311Asn)	Missense	Pathogenic	F	85	Unknown
18135949	1920	IMPDH1	NM_000883.4	c.931G>A	p.(Asp311Asn)	Missense	Pathogenic	F	59	White - British
10089680	1921	IMPDH1	NM_000883.4	c.713A>G	p.(Lys238Arg)	Missense	Variant of Uncertain Significance	M	64	Unknown
5628076	1922	IMPDH1	NM_000883.4	c.713A>G	p.(Lys238Arg)	Missense	Variant of Uncertain Significance	F	34	White - British
4779844	1923	IMPDH1	NM_000883.4	c.779T>C	p.(Leu260Pro)	Missense	Likely Pathogenic	M	73	White - British
4177928	1924	IMPDH1	NM_000883.4	c.772A>C	p.(Thr258Pro)	Missense	Variant of Uncertain Significance	F	48	White - British
10407585	1924	IMPDH1	NM_000883.4	c.928A>C	p.(Thr310Pro)	Missense	Likely Pathogenic	M	22	White - British
10407592	1924	IMPDH1	NM_000883.4	c.928A>C	p.(Thr310Pro)	Missense	Likely Pathogenic	M	19	White - British
6911323	1925	IMPDH1	NM_000883.4	3bp deletion in non-coding exon 2	Regulatory	Regulatory	NA	F	71	Any other ethnic group

15488878	1926	IMPDH1	NM_000883.4	c.1031G>A	p.(Arg344His)	Missense	Variant of Uncertain Significance	F	60	Any other ethnic group
8701895	1927	IMPG1	NM_001563.4	c.1157C>A	p.(Ala386Asp)	Missense	Likely Pathogenic	M	85	Not stated
9142734	1928	IMPG1	NM_001563.4	c.807+2T>A	Splice	Splice	Likely Pathogenic	M	46	Not stated
8055767	1929	IMPG2	NM_016247.4	c.68dup	p.(Asp23GlufsTer29)	Frameshift	Pathogenic	F	32	Not stated
8055767	1929	IMPG2	NM_016247.4	c.68dup	p.(Asp23GlufsTer29)	Frameshift	Pathogenic	F	32	Not stated
9821041	1930	IMPG2	NM_016247.4	c.1875_1879dup	p.(Pro627LeufsTer25Ter)	Frameshift	Likely Pathogenic	M	30	Not stated
9821041	1930	IMPG2	NM_016247.4	c.1875_1879dup	p.(Pro627LeufsTer25Ter)	Frameshift	Likely Pathogenic	M	30	Not stated
11169962	1931	IMPG2	NM_016247.4	c.1680T>A	p.(Tyr560Ter)	Stopgain	Pathogenic	M	31	Asian - Pakistani
11169962	1931	IMPG2	NM_016247.4	c.1680T>A	p.(Tyr560Ter)	Stopgain	Pathogenic	M	31	Asian - Pakistani
10554375	1932	IMPG2	NM_016247.4	c.118G>T	p.(Glu40Ter)	Stopgain	Pathogenic	F	52	Not stated
10554375	1932	IMPG2	NM_016247.4	c.2426G>A	p.(Trp809Ter)	Stopgain	Pathogenic	F	52	Not stated
12504428	1933	IMPG2	NM_016247.4	c.1680T>A	p.(Tyr560Ter)	Stopgain	Pathogenic	F	33	Any other ethnic group
12504428	1933	IMPG2	NM_016247.4	c.1680T>A	p.(Tyr560Ter)	Stopgain	Pathogenic	F	33	Any other ethnic group
4303179	1934	IMPG2	NM_016247.4	c.2426G>A	p.(Trp809Ter)	Stopgain	Pathogenic	M	53	White - British
4303179	1934	IMPG2	NM_016247.4	c.3413_3420delinsATAA	p.(Ser1138AsnfsTer19)	Frameshift	Likely Pathogenic	M	53	White - British
2277407	1935	IMPG2	NM_016247.4	c.2928del	p.(Asn976LysfsTer6)	Frameshift	Pathogenic	M	50	Not stated
2277407	1935	IMPG2	NM_016247.4	c.3023-6_3030dup	p.(Ala10011PhefsTer2)	Frameshift	Pathogenic	M	50	Not stated
2111899	1936	IMPG2	NM_016247.4	c.2716C>T	p.(Arg906Ter)	Stopgain	Pathogenic	M	64	Unknown
2111899	1936	IMPG2	NM_016247.4	c.3056G>A	p.(Cys1019Tyr)	Missense	Variant of Uncertain Significance	M	64	Unknown
4394011	1937	INPP5E	NM_019892.6	c.1456C>T	p.(Arg486Cys)	Missense	Likely Pathogenic	M	48	White - British
4394011	1937	INPP5E	NM_019892.6	c.1577C>T	p.(Pro526Leu)	Missense	Likely Pathogenic	M	48	White - British
11816573	1938	INPP5E	NM_019892.6	c.1402C>T	p.(Arg468Cys)	Missense	Likely Pathogenic	M	29	White - British
11816573	1938	INPP5E	NM_019892.6	c.1861C>T	p.(Arg621Trp)	Missense	Pathogenic	M	29	White - British
11816601	1938	INPP5E	NM_019892.6	c.1402C>T	p.(Arg468Cys)	Missense	Likely Pathogenic	F	33	Any other ethnic group
11816601	1938	INPP5E	NM_019892.6	c.1861C>T	p.(Arg621Trp)	Missense	Pathogenic	F	33	Any other ethnic group
11339509	1939	INPP5E	NM_019892.6	c.1754G>A	p.(Arg585His)	Missense	Pathogenic	F	36	White - British
11339509	1939	INPP5E	NM_019892.6	c.1760del	p.(Val587GlyfsTer7)	Frameshift	Pathogenic	F	36	White - British
6789005	1940	IQCB1	NM_001023570.4	c.1036G>T	p.(Glu346Ter)	Stopgain	Pathogenic	F	23	White - British
6789005	1940	IQCB1	NM_001023570.4	c.260T>G	p.(Leu87Ter)	Stopgain	Pathogenic	F	23	White - British
8806559	1941	IQCB1	NM_001023570.4	c.424_425del	p.(Phe142ProfsTer5)	Frameshift	Pathogenic	M	18	White - British
8806559	1941	IQCB1	NM_001023570.4	c.424_425del	p.(Phe142ProfsTer5)	Frameshift	Pathogenic	M	18	White - British
11381481	1942	IQCB1	NM_001023570.4	c.1278+1G>A	Splice	Splice	Pathogenic	F	14	Mixed - White and Asian
11381481	1942	IQCB1	NM_001023570.4	c.1381C>T	p.(Arg461Ter)	Stopgain	Pathogenic	F	14	Mixed - White and Asian
12419154	1943	IQCB1	NM_001023570.4	c.214C>T	p.(Arg72Ter)	Stopgain	Pathogenic	F	80	Not stated
12419154	1943	IQCB1	NM_001023570.4	c.424_425del	p.(Phe142ProfsTer5)	Frameshift	Pathogenic	F	80	Not stated
13748755	1944	IQCB1	NM_001023570.4	c.1504C>T	p.(Arg502Ter)	Stopgain	Pathogenic	F	14	Not stated
13748755	1944	IQCB1	NM_001023570.4	c.814C>T	p.(Gln272Ter)	Stopgain	Pathogenic	F	14	Not stated
15659272	1945	IQCB1	NM_001023570.4	c.1518_1519del	p.(His506GlnfsTer13)	Frameshift	Pathogenic	M	8	White - British
15659272	1945	IQCB1	NM_001023570.4	c.424_425del	p.(Phe142ProfsTer5)	Frameshift	Pathogenic	M	8	White - British
11923799	1946	IQCB1	NM_001023570.4	c.700_701del	p.(Leu234ThrfsTer5)	Frameshift	Likely Pathogenic	M	66	Unknown
11923799	1946	IQCB1	NM_001023570.4	c.700_701del	p.(Leu234ThrfsTer5)	Frameshift	Likely Pathogenic	M	66	Unknown
15198287	1947	IQCB1	NM_001023570.4	Exon 9 deletion	Deletion	Deletion	Variant of Uncertain Significance	F	45	Any other ethnic group
15198287	1947	IQCB1	NM_001023570.4	Exon 9 deletion	Deletion	Deletion	Variant of Uncertain Significance	F	45	Any other ethnic group
16093643	1948	IQCB1	NM_001023570.4	c.488-1G>A	Splice	Splice	Pathogenic	F	24	Asian - Pakistani
16093643	1948	IQCB1	NM_001023570.4	c.488-1G>A	Splice	Splice	Pathogenic	F	24	Asian - Pakistani
16392732	1949	IQCB1	NM_001023570.4	c.1518_1519del	p.(His506GlnfsTer13)	Frameshift	Pathogenic	M	56	White - British
16392732	1949	IQCB1	NM_001023570.4	c.897_900dup	p.(Ile301LeufsTer42)	Frameshift	Pathogenic	M	56	White - British
17081455	1950	IQCB1	NM_001023570.4	c.1036G>T	p.(Glu346Ter)	Stopgain	Pathogenic	M	7	Not stated
17081455	1950	IQCB1	NM_001023570.4	c.1518_1519del	p.(His506GlnfsTer13)	Frameshift	Pathogenic	M	7	Not stated
11748519	1951	IQCB1	NM_001023570.4	c.745A>T	p.(Arg249Ter)	Stopgain	Likely Pathogenic	F	50	Asian - Bangladeshi
11748519	1951	IQCB1	NM_001023570.4	c.745A>T	p.(Arg249Ter)	Stopgain	Likely Pathogenic	F	50	Asian - Bangladeshi

16802953	1951	IQCB1	NM_001023570.4	c.745A>T	p.(Arg249Ter)	Stopgain	Likely Pathogenic	F	47	Asian - Bangladeshi
16802953	1951	IQCB1	NM_001023570.4	c.745A>T	p.(Arg249Ter)	Stopgain	Likely Pathogenic	F	47	Asian - Bangladeshi
8026885	1952	JAG1	NM_000214.3	c.2122_2125del	p.(Gln708ValfsTer34)	Frameshift	Pathogenic	F	37	White - British
1147019	1953	KCNJ13	NM_002242.4	c.722T>C	p.(Leu241Pro)	Missense	Likely Pathogenic	M	45	White - British
1147019	1953	KCNJ13	NM_002242.4	c.722T>C	p.(Leu241Pro)	Missense	Likely Pathogenic	M	45	White - British
3468919	1954	KCNV2	NM_133497.4	c.916G>T	p.(Glu306Ter)	Stopgain	Pathogenic	M	47	Asian - Pakistani
3468919	1954	KCNV2	NM_133497.4	c.916G>T	p.(Glu306Ter)	Stopgain	Pathogenic	M	47	Asian - Pakistani
10127046	1955	KCNV2	NM_133497.4	c.325C>T	p.(Gln109Ter)	Stopgain	Pathogenic	F	41	Asian - Bangladeshi
10127046	1955	KCNV2	NM_133497.4	c.325C>T	p.(Gln109Ter)	Stopgain	Pathogenic	F	41	Asian - Bangladeshi
8329544	1955	KCNV2	NM_133497.4	c.325C>T	p.(Gln109Ter)	Stopgain	Pathogenic	M	37	Any other ethnic group
8329544	1955	KCNV2	NM_133497.4	c.325C>T	p.(Gln109Ter)	Stopgain	Pathogenic	M	37	Any other ethnic group
8437260	1956	KCNV2	NM_133497.4	c.427G>T	p.(Glu143Ter)	Stopgain	Pathogenic	M	29	Any other ethnic group
8437260	1956	KCNV2	NM_133497.4	c.767C>G	p.(Ser256Trp)	Missense	Likely Pathogenic	M	29	Any other ethnic group
8437288	1956	KCNV2	NM_133497.4	c.427G>T	p.(Glu143Ter)	Stopgain	Pathogenic	F	28	Not stated
8437288	1956	KCNV2	NM_133497.4	c.767C>G	p.(Ser256Trp)	Missense	Likely Pathogenic	F	28	Not stated
3612916	1957	KCNV2	NM_133497.4	c.433C>T	p.(Gln145Ter)	Stopgain	Likely Pathogenic	F	57	White - British
3612916	1957	KCNV2	NM_133497.4	c.776C>T	p.(Ala259Val)	Missense	Likely Pathogenic	F	57	White - British
664453	1958	KCNV2	NM_133497.4	c.7A>T	p.(Lys3Ter)	Stopgain	Likely Pathogenic	M	77	White - British
664453	1958	KCNV2	NM_133497.4	c.7A>T	p.(Lys3Ter)	Stopgain	Likely Pathogenic	M	77	White - British
9940538	1959	KCNV2	NM_133497.4	Exon 2 deletion	Deletion	Deletion	Likely Pathogenic	F	21	Unknown
9940538	1959	KCNV2	NM_133497.4	Exon 2 deletion	Deletion	Deletion	Likely Pathogenic	F	21	Unknown
3672507	1960	KCNV2	NM_133497.4	c.1381G>A	p.(Gly461Arg)	Missense	Pathogenic	F	61	Not stated
3672507	1960	KCNV2	NM_133497.4	c.1381G>A	p.(Gly461Arg)	Missense	Pathogenic	F	61	Not stated
11204038	1961	KCNV2	NM_133497.4	c.562T>A	p.(Trp188Arg)	Missense	Pathogenic	F	26	Any other ethnic group
11204038	1961	KCNV2	NM_133497.4	c.8_11del	p.(Lys3ArgfsTer96)	Frameshift	Pathogenic	F	26	Any other ethnic group
11420667	1962	KCNV2	NM_133497.4	c.1A>G	p.(Met1?)	Start codon	Likely Pathogenic	F	50	Not stated
11420667	1962	KCNV2	NM_133497.4	c.766T>G	p.(Ser256Ala)	Missense	Variant of Uncertain Significance	F	50	Not stated
11981962	1963	KCNV2	NM_133497.4	c.1096del	p.(Val366TrpfsTer88)	Frameshift	Pathogenic	F	45	White - Other
11981962	1963	KCNV2	NM_133497.4	c.339C>A	p.(Cys113Ter)	Stopgain	Pathogenic	F	45	White - Other
11293575	1964	KCNV2	NM_133497.4	Partial exon 1 duplication	Duplication	Duplication	Likely Pathogenic	F	14	White - British
11293575	1964	KCNV2	NM_133497.4	Partial exon 1 duplication	Duplication	Duplication	Likely Pathogenic	F	14	White - British
13316197	1965	KCNV2	NM_133497.4	c.427G>T	p.(Glu143Ter)	Stopgain	Pathogenic	M	26	Unknown
13316197	1965	KCNV2	NM_133497.4	c.427G>T	p.(Glu143Ter)	Stopgain	Pathogenic	M	26	Unknown
3162760	1966	KCNV2	NM_133497.4	c.1381G>A	p.(Gly461Arg)	Missense	Pathogenic	M	49	Unknown
3162760	1966	KCNV2	NM_133497.4	c.433C>T	p.(Gln145Ter)	Stopgain	Likely Pathogenic	M	49	Unknown
1213869	1966	KCNV2	NM_133497.4	c.1381G>A	p.(Gly461Arg)	Missense	Pathogenic	M	52	Not stated
1213869	1966	KCNV2	NM_133497.4	c.433C>T	p.(Gln145Ter)	Stopgain	Likely Pathogenic	M	52	Not stated
13094332	1967	KCNV2	NM_133497.4	c.445_446del	p.(Tyr149LeufsTer222)	Frameshift	Likely Pathogenic	F	26	Not stated
13094332	1967	KCNV2	NM_133497.4	c.445_446del	p.(Tyr149LeufsTer222)	Frameshift	Likely Pathogenic	F	26	Not stated
12726545	1968	KCNV2	NM_133497.4	c.1199del	p.(Phe400SerfsTer54)	Frameshift	Pathogenic	M	15	Unknown
12726545	1968	KCNV2	NM_133497.4	c.417C>A	p.(Cys139Ter)	Stopgain	Pathogenic	M	15	Unknown
13839132	1969	KCNV2	NM_133497.4	c.1381G>A	p.(Gly461Arg)	Missense	Pathogenic	M	30	Unknown
13839132	1969	KCNV2	NM_133497.4	c.494A>G	p.(Tyr165Cys)	Missense	Likely Pathogenic	M	30	Unknown
13816648	1970	KCNV2	NM_133497.4	c.1110_1128del	p.(Lys371AlafsTer77)	Frameshift	Likely Pathogenic	F	32	Asian - Indian
13816648	1970	KCNV2	NM_133497.4	c.1110_1128del	p.(Lys371AlafsTer77)	Frameshift	Likely Pathogenic	F	32	Asian - Indian
11224632	1971	KCNV2	NM_133497.4	c.325C>T	p.(Gln109Ter)	Stopgain	Pathogenic	F	42	Asian - Bangladeshi
11224632	1971	KCNV2	NM_133497.4	c.325C>T	p.(Gln109Ter)	Stopgain	Pathogenic	F	42	Asian - Bangladeshi
12295100	1972	KCNV2	NM_133497.4	c.778A>T	p.(Lys260Ter)	Stopgain	Pathogenic	M	30	Any other ethnic group
12295100	1972	KCNV2	NM_133497.4	c.778A>T	p.(Lys260Ter)	Stopgain	Pathogenic	M	30	Any other ethnic group
16828790	1973	KCNV2	NM_133497.4	c.782C>A	p.(Ala261Asp)	Missense	Variant of Uncertain Significance	M	35	Not stated
16828790	1973	KCNV2	NM_133497.4	c.782C>A	p.(Ala261Asp)	Missense	Variant of Uncertain Significance	M	35	Not stated

17256511	1974	KCNV2	NM_133497.4	c.1381G>A	p.(Gly461Arg)	Missense	Pathogenic	M	10	Not stated
17256511	1974	KCNV2	NM_133497.4	c.874_889dup	p.(Asp297GlyfsTer80)	Frameshift	Likely Pathogenic	M	10	Not stated
17343031	1975	KCNV2	NM_133497.4	c.778A>T	p.(Lys260Ter)	Stopgain	Pathogenic	M	44	Not stated
17343031	1975	KCNV2	NM_133497.4	c.866C>A	p.(Ser289Ter)	Stopgain	Pathogenic	M	44	Not stated
17117267	1976	KCNV2	NM_133497.4	c.1381G>T	p.(Gly461Ter)	Stopgain	Pathogenic	M	36	Any other ethnic group
17117267	1976	KCNV2	NM_133497.4	c.442G>T	p.(Glu148Ter)	Stopgain	Pathogenic	M	36	Any other ethnic group
9122329	1977	KCNV2	NM_133497.4	Whole gene deletion	Deletion	Deletion	Likely Pathogenic	F	24	Not stated
9122329	1977	KCNV2	NM_133497.4	Whole gene deletion	Deletion	Deletion	Likely Pathogenic	F	24	Not stated
11282249	1978	KIF11	NM_004523.4	c.308+88_387+327del	Deletion	Deletion	Likely Pathogenic	M	13	White - British
11581632	1979	KIF11	NM_004523.4	Exon 1 deletion	Deletion	Deletion	Likely Pathogenic	M	19	White - Other
10604677	1980	KIF11	NM_004523.4	c.413G>A	p.(Arg138His)	Missense	Variant of Uncertain Significance	M	29	Not stated
14827861	1981	KIF11	NM_004523.4	c.247C>T	p.(Arg83Ter)	Stopgain	Pathogenic	M	9	White - British
17574059	1982	KIF11	NM_004523.4	c.1431_1434del	p.(Glu478AsnfsTer60)	Frameshift	Likely Pathogenic	M	11	Mixed - White and Asian
1304799	1983	KLHL7	NM_001031710.3	c.433A>G	p.(Asn145Asp)	Missense	Pathogenic	M	53	Not stated
16065209	1983	KLHL7	NM_001031710.3	c.433A>G	p.(Asn145Asp)	Missense	Pathogenic	F	35	Not stated
17652704	1984	KLHL7	NM_001031710.3	c.422T>C	p.(Val141Ala)	Missense	Likely Pathogenic	M	45	Not stated
3009768	1985	KLHL7	NM_001031710.3	c.433A>G	p.(Asn145Asp)	Missense	Pathogenic	M	52	White - British
16991757	1986	KLHL7	NM_001031710.3	c.458C>T	p.(Ala153Val)	Missense	Pathogenic	M	36	Not stated
6424753	1987	KLHL7	NM_001031710.3	c.422T>C	p.(Val141Ala)	Missense	Likely Pathogenic	F	67	Asian - Other
11051599	1987	KLHL7	NM_001031710.3	c.422T>C	p.(Val141Ala)	Missense	Likely Pathogenic	F	32	Asian - Other
10243043	1988	KLHL7	NM_001031710.3	c.458C>T	p.(Ala153Val)	Missense	Pathogenic	M	80	White - British
17348169	1989	KLHL7	NM_001031710.3	c.422T>C	p.(Val141Ala)	Missense	Likely Pathogenic	F	44	White - British
322664	1990	LAMA1	NM_005559.4	c.1492del	p.(Arg498GlyfsTer25)	Frameshift	Likely Pathogenic	M	42	Asian - Indian
322664	1990	LAMA1	NM_005559.4	c.1492del	p.(Arg498GlyfsTer25)	Frameshift	Likely Pathogenic	M	42	Asian - Indian
1214772	1990	LAMA1	NM_005559.4	c.1492del	p.(Arg498GlyfsTer25)	Frameshift	Likely Pathogenic	M	40	Asian - Indian
1214772	1990	LAMA1	NM_005559.4	c.1492del	p.(Arg498GlyfsTer25)	Frameshift	Likely Pathogenic	M	40	Asian - Indian
719767	1990	LAMA1	NM_005559.4	c.1492del	p.(Arg498GlyfsTer25)	Frameshift	Likely Pathogenic	F	45	Asian - Indian
719767	1990	LAMA1	NM_005559.4	c.1492del	p.(Arg498GlyfsTer25)	Frameshift	Likely Pathogenic	F	45	Asian - Indian
10783478	1991	SPATA7	NM_018418.5	c.253C>T	p.(Arg85Ter)	Stopgain	Pathogenic	M	32	Unknown
10783478	1991	SPATA7	NM_018418.5	c.253C>T	p.(Arg85Ter)	Stopgain	Pathogenic	M	32	Unknown
7236102	1992	SPATA7	NM_018418.5	c.253C>T	p.(Arg85Ter)	Stopgain	Pathogenic	M	56	Not stated
7236102	1992	SPATA7	NM_018418.5	c.253C>T	p.(Arg85Ter)	Stopgain	Pathogenic	M	56	Not stated
8322005	1993	SPATA7	NM_018418.5	c.1229_1231del	p.(His410del)	Inframe indel	Variant of Uncertain Significance	M	36	Not stated
8322005	1993	SPATA7	NM_018418.5	c.265_268del	p.(Leu89LysfsTer4)	Frameshift	Likely Pathogenic	M	36	Not stated
8322033	1993	SPATA7	NM_018418.5	c.1229_1231del	p.(His410del)	Inframe indel	Variant of Uncertain Significance	M	33	Not stated
8322033	1993	SPATA7	NM_018418.5	c.265_268del	p.(Leu89LysfsTer4)	Frameshift	Pathogenic	M	33	Not stated
6999691	1994	SPATA7	NM_018418.5	c.253C>T	p.(Arg85Ter)	Stopgain	Pathogenic	F	42	Any other ethnic group
6999691	1994	SPATA7	NM_018418.5	c.253C>T	p.(Arg85Ter)	Stopgain	Pathogenic	F	42	Any other ethnic group
15986543	1995	SPATA7	NM_018418.5	c.253C>T	p.(Arg85Ter)	Stopgain	Pathogenic	M	22	Asian - Pakistani
15986543	1995	SPATA7	NM_018418.5	c.253C>T	p.(Arg85Ter)	Stopgain	Pathogenic	M	22	Asian - Pakistani
9066112	1996	LCA5	NM_181714.4	c.1151del	p.(Pro384GlnfsTer18)	Frameshift	Pathogenic	F	18	Asian - Other
9066112	1996	LCA5	NM_181714.4	c.1151del	p.(Pro384GlnfsTer18)	Frameshift	Pathogenic	F	18	Asian - Other
9645922	1997	LCA5	NM_181714.4	c.1756A>T	p.(Lys586Ter)	Stopgain	Pathogenic	M	28	Not stated
9645922	1997	LCA5	NM_181714.4	c.835C>T	p.(Gln279Ter)	Stopgain	Pathogenic	M	28	Not stated
9865421	1998	LCA5	NM_181714.4	c.633_639del	p.(Glu211AspfsTer13)	Frameshift	Pathogenic	F	21	White - Other
9865421	1998	LCA5	NM_181714.4	c.633_639del	p.(Glu211AspfsTer13)	Frameshift	Pathogenic	F	21	White - Other
10267431	1999	LCA5	NM_181714.4	c.1676C>A	p.(Ser559Ter)	Stopgain	Pathogenic	F	16	Unknown
10267431	1999	LCA5	NM_181714.4	c.1676C>A	p.(Ser559Ter)	Stopgain	Pathogenic	F	16	Unknown
10051768	2000	LCA5	NM_181714.4	c.1151del	p.(Pro384GlylnfsTer18)	Frameshift	Pathogenic	M	32	Any other ethnic group
10051768	2000	LCA5	NM_181714.4	c.1151del	p.(Pro384GlylnfsTer18)	Frameshift	Pathogenic	M	32	Any other ethnic group
12101774	2001	LCA5	NM_181714.4	c.633_639del	p.(Glu211AspfsTer13)	Frameshift	Pathogenic	F	20	Black - Other

12101774	2001	LCA5	NM_181714.4	c.633_639del	p.(Glu211AspfsTer13)	Frameshift	Pathogenic	F	20	Black - Other
1743251	2002	LCA5	NM_181714.4	c.1756A>T	p.(Lys586Ter)	Stopgain	Pathogenic	F	42	White - British
1743251	2002	LCA5	NM_181714.4	c.1756A>T	p.(Lys586Ter)	Stopgain	Pathogenic	F	42	White - British
12641642	2003	LCA5	NM_181714.4	c.838C>T	p.(Arg280Ter)	Stopgain	Pathogenic	M	12	Not stated
12641642	2003	LCA5	NM_181714.4	c.838C>T	p.(Arg280Ter)	Stopgain	Pathogenic	M	12	Not stated
14989624	2004	LCA5	NM_181714.4	c.887dup	p.(Asn296LysfsTer5)	Frameshift	Likely Pathogenic	M	38	Not stated
14989624	2004	LCA5	NM_181714.4	c.887dup	p.(Asn296LysfsTer5)	Frameshift	Likely Pathogenic	M	38	Not stated
15615067	2005	LCA5	NM_181714.4	c.1811del	p.(Leu604Ter)	Stopgain	Likely Pathogenic	F	41	Unknown
15615067	2005	LCA5	NM_181714.4	c.1811del	p.(Leu604Ter)	Stopgain	Likely Pathogenic	F	41	Unknown
15955666	2006	LCA5	NM_181714.4	c.795T>G	p.(Tyr265Ter)	Stopgain	Pathogenic	F	15	Not stated
15955666	2006	LCA5	NM_181714.4	c.795T>G	p.(Tyr265Ter)	Stopgain	Pathogenic	F	15	Not stated
9335157	2007	D1, MT-ND4, MT-ND6		m.11778G>A	Mitochondrial	Mitochondrial	Pathogenic	M	78	White - British
9905580	2008	D1, MT-ND4, MT-ND6		m.3635G>A	Mitochondrial	Mitochondrial	Pathogenic	M	51	Not stated
9539249	2009	D1, MT-ND4, MT-ND6		m.11778G>A	Mitochondrial	Mitochondrial	Pathogenic	M	46	Black - African
15358832	2010	D1, MT-ND4, MT-ND6		m.11778G>A	Mitochondrial	Mitochondrial	Pathogenic	M	63	Not stated
15358832	2010	D1, MT-ND4, MT-ND6		m.11778G>A	Mitochondrial	Mitochondrial	Pathogenic	M	63	Not stated
16987312	2011	D1, MT-ND4, MT-ND6		m.11778G>A	Mitochondrial	Mitochondrial	Pathogenic	M	52	White - British
16987312	2011	D1, MT-ND4, MT-ND6		m.11778G>A	Mitochondrial	Mitochondrial	Pathogenic	M	52	White - British
17970609	2012	D1, MT-ND4, MT-ND6		m.11778G>A	Mitochondrial	Mitochondrial	Pathogenic	M	45	Unknown
18061994	2013	D1, MT-ND4, MT-ND6		m.11778G>A	Mitochondrial	Mitochondrial	Pathogenic	F	58	Not stated
17903031	2014	D1, MT-ND4, MT-ND6		m.11778G>A	Mitochondrial	Mitochondrial	Pathogenic	F	38	Not stated
18481077	2015	D1, MT-ND4, MT-ND6		m.11778G>A	Mitochondrial	Mitochondrial	Pathogenic	F	60	Not stated
18728289	2016	D1, MT-ND4, MT-ND6		m.11778G>A	Mitochondrial	Mitochondrial	Pathogenic	M	25	White - Other
7639939	2017	LRAT	NM_004744.5	c.40_41delinsTT	p.(Glu14Leu)	Inframe indel	Variant of Uncertain Significance	F	67	White - British
7639939	2017	LRAT	NM_004744.5	c.40_41delinsTT	p.(Glu14Leu)	Inframe indel	Variant of Uncertain Significance	F	67	White - British
5635615	2018	LRAT	NM_004744.5	c.316G>A	p.(Ala106Thr)	Missense	Likely Pathogenic	F	42	Black - Caribbean
5635615	2018	LRAT	NM_004744.5	c.316G>A	p.(Ala106Thr)	Missense	Likely Pathogenic	F	42	Black - Caribbean
12911275	2019	LRP	NM_004525.3	c.4733G>C	p.(Arg1578Pro)	Missense	Likely Pathogenic	M	58	White - British
12911275	2019	LRP	NM_004525.3	c.9733G>T	p.(Asp3245Tyr)	Missense	Pathogenic	M	58	White - British
4359921	2020	LRP5	NM_002335.4	c.1733G>A	p.(Arg578Gln)	Missense	Likely Pathogenic	F	31	Not stated
4359921	2020	LRP5	NM_002335.4	c.4454_4465del	p.(Ser1485_Ser1488del)	Inframe indel	Variant of Uncertain Significance	F	31	Not stated
6391272	2021	LRP5	NM_002335.4	c.1797del	p.(Val600SerfsTer35)	Frameshift	Likely Pathogenic	F	30	White - British
1057097	2022	LRP5	NM_002335.4	c.3871G>T	p.(Asp1291Tyr)	Missense	Likely Pathogenic	F	48	Not stated
1057097	2022	LRP5	NM_002335.4	c.3871G>T	p.(Asp1291Tyr)	Missense	Likely Pathogenic	F	48	Not stated
1057104	2022	LRP5	NM_002335.4	c.3871G>T	p.(Asp1291Tyr)	Missense	Likely Pathogenic	M	50	Not stated
1057104	2022	LRP5	NM_002335.4	c.3871G>T	p.(Asp1291Tyr)	Missense	Likely Pathogenic	M	50	Not stated
10940369	2023	LRP5	NM_002335.4	c.1265C>T	p.(Ala422Val)	Missense	Likely Pathogenic	M	17	White - British
10940369	2023	LRP5	NM_002335.4	c.3242T>G	p.(Leu1081Arg)	Missense	Likely Pathogenic	M	17	White - British
11622799	2024	LRP5	NM_002335.4	c.2254C>T	p.(Arg752Trp)	Missense	Likely Pathogenic	F	13	White - British
11622799	2024	LRP5	NM_002335.4	c.3914G>A	p.(Cys1305Tyr)	Missense	Likely Pathogenic	F	13	White - British
13312564	2025	LRP5	NM_002335.4	c.4081T>G	p.(Cys1361Gly)	Missense	Pathogenic	M	12	White - Other
13312564	2025	LRP5	NM_002335.4	c.4081T>G	p.(Cys1361Gly)	Missense	Pathogenic	M	12	White - Other
4325747	2026	LRP5	NM_002335.4	c.1319T>C	p.(Ile440Thr)	Missense	Likely Pathogenic	M	38	White - British
4325747	2026	LRP5	NM_002335.4	c.4099G>A	p.(Glu1367Lys)	Missense	Likely Pathogenic	M	38	White - British
7290975	2027	LRP5	NM_002335.4	c.2366C>A	p.(Ala789Asp)	Missense	Variant of Uncertain Significance	F	48	White - British
7290975	2027	LRP5	NM_002335.4	c.2873G>A	p.(Arg958Gln)	Missense	Variant of Uncertain Significance	F	48	White - British
17893854	2028	LRP5	NM_002335.4	c.4488+2T>G	Splice	Splice	Pathogenic	F	48	Not stated
8893331	2029	MAK	NM_001242957.3	Exon 2 deletion	Deletion	Deletion	Likely Pathogenic	M	60	Not stated
8893331	2029	MAK	NM_001242957.3	Exon 2 deletion	Deletion	Deletion	Likely Pathogenic	M	60	Not stated
6011151	2030	MERTK	NM_006343.3	c.345C>G	p.(Cys115Trp)	Missense	Likely Pathogenic	F	62	Not stated
6011151	2030	MERTK	NM_006343.3	c.634A>C	p.(Thr212Pro)	Missense	Variant of Uncertain Significance	F	62	Not stated

6894131	2031	MERTK	NM_006343.3	c.1951C>T	p.(Arg651Ter)	Stopgain	Pathogenic	M	36	White - British
6894131	2031	MERTK	NM_006343.3	c.61+1G>A	Splice	Splice	Pathogenic	M	36	White - British
9488562	2032	MERTK	NM_006343.3	c.1470del	p.(Ser491LeufsTer24)	Frameshift	Likely Pathogenic	F	29	Asian - Bangladeshi
9488562	2032	MERTK	NM_006343.3	c.1470del	p.(Ser491LeufsTer24)	Frameshift	Likely Pathogenic	F	29	Asian - Bangladeshi
7912820	2033	MERTK	NM_006343.3	c.2530C>T	p.(Arg844Cys)	Missense	Pathogenic	F	39	Mixed - Other
7912820	2033	MERTK	NM_006343.3	c.2530C>T	p.(Arg844Cys)	Missense	Pathogenic	F	39	Mixed - Other
14888257	2033	MERTK	NM_006343.3	c.2530C>T	p.(Arg844Cys)	Missense	Pathogenic	M	33	Not stated
14888257	2033	MERTK	NM_006343.3	c.2530C>T	p.(Arg844Cys)	Missense	Pathogenic	M	33	Not stated
10878531	2034	MERTK	NM_006343.3	c.2214del	p.(Cys738TrpfsTer32)	Frameshift	Pathogenic	F	19	Unknown
10878531	2034	MERTK	NM_006343.3	c.2214del	p.(Cys738TrpfsTer32)	Frameshift	Pathogenic	F	19	Unknown
10721843	2035	MERTK	NM_006343.3	c.2486+6T>A	Splice	Splice	Variant of Uncertain Significance	M	30	Not stated
10721843	2035	MERTK	NM_006343.3	Exon 7 deletion	Deletion	Deletion	Likely Pathogenic	M	30	Not stated
11804771	2036	MERTK	NM_006343.3	c.2179C>G	p.(Arg727Gly)	Missense	Likely Pathogenic	F	39	White - British
11804771	2036	MERTK	NM_006343.3	c.2180G>A	p.(Arg727Gln)	Missense	Pathogenic	F	39	White - British
12738683	2037	MERTK	NM_006343.3	c.2163T>A	p.(His721Gln)	Missense	Likely Pathogenic	M	31	White - Other
12738683	2037	MERTK	NM_006343.3	c.2163T>A	p.(His721Gln)	Missense	Likely Pathogenic	M	31	White - Other
13310520	2038	MERTK	NM_006343.3	c.2164C>T	p.(Arg722Ter)	Stopgain	Pathogenic	M	21	White - British
13310520	2038	MERTK	NM_006343.3	Exon 1-7 deletion	Deletion	Deletion	Likely Pathogenic	M	21	White - British
13705901	2039	MERTK	NM_006343.3	c.263C>T	p.(Ser88Leu)	Missense	Variant of Uncertain Significance	M	22	Any other ethnic group
13705901	2039	MERTK	NM_006343.3	Exon 3-19 deletion	Deletion	Deletion	Likely Pathogenic	M	22	Any other ethnic group
14921255	2040	MERTK	NM_006343.3	c.2180G>A	p.(Arg727Gln)	Missense	Pathogenic	M	25	Unknown
14921255	2040	MERTK	NM_006343.3	c.933_935delinsTT	p.(Pro313ArgfsTer15)	Frameshift	Pathogenic	M	25	Unknown
12721295	2041	MERTK	NM_006343.3	c.1868T>A	p.(Leu623Ter)	Stopgain	Pathogenic	F	24	Black - African
12721295	2041	MERTK	NM_006343.3	c.2167G>A	p.(Asp723Asn)	Missense	Variant of Uncertain Significance	F	24	Black - African
13878955	2042	MERTK	NM_006343.3	c.1672C>T	p.(Arg558Ter)	Stopgain	Pathogenic	F	33	Asian - Other
13878955	2042	MERTK	NM_006343.3	c.2190-2A>G	Splice	Splice	Pathogenic	F	33	Asian - Other
10980584	2043	MERTK	NM_006343.3	Exon 8 deletion	Deletion	Deletion	Likely Pathogenic	F	30	Not stated
10980584	2043	MERTK	NM_006343.3	Exon 8 deletion	Deletion	Deletion	Likely Pathogenic	F	30	Not stated
16941875	2044	MERTK	NM_006343.3	c.2486+6T>A	Splice	Splice	Variant of Uncertain Significance	F	21	Not stated
16941875	2044	MERTK	NM_006343.3	c.2486+6T>A	Splice	Splice	Variant of Uncertain Significance	F	21	Not stated
17338978	2045	MERTK	NM_006343.3	c.1102T>C	p.(Ser368Pro)	Missense	Pathogenic	M	26	Not stated
17338978	2045	MERTK	NM_006343.3	c.245del	p.(Gln82ArgfsTer2)	Frameshift	Likely Pathogenic	M	26	Not stated
18212382	2046	MERTK	NM_006343.3	c.2163T>A	p.(His721Gln)	Missense	Likely Pathogenic	F	18	Not stated
18212382	2046	MERTK	NM_006343.3	c.2163T>A	p.(His721Gln)	Missense	Likely Pathogenic	F	18	Not stated
18461918	2047	MERTK	NM_006343.3	c.2194C>T	p.(Arg732Ter)	Stopgain	Likely Pathogenic	M	29	Not stated
18461918	2047	MERTK	NM_006343.3	c.2194C>T	p.(Arg732Ter)	Stopgain	Likely Pathogenic	M	29	Not stated
4484059	2048	MFRP	NM_031433.4	c.498del	p.(Asn167ThrfsTer25)	Frameshift	Pathogenic	M	57	White - Other
4484059	2048	MFRP	NM_031433.4	c.498del	p.(Asn167ThrfsTer25)	Frameshift	Pathogenic	M	57	White - Other
13346486	2049	MFRP	NM_031433.4	c.1150del	p.(His384ThrfsTer94)	Frameshift	Likely Pathogenic	F	12	Asian - Pakistani
13346486	2049	MFRP	NM_031433.4	c.1150del	p.(His384ThrfsTer94)	Frameshift	Likely Pathogenic	F	12	Asian - Pakistani
6611408	2050	MFRP	NM_031433.4	c.1615C>T	p.(Arg539Cys)	Missense	Likely Pathogenic	M	52	Not stated
6611408	2050	MFRP	NM_031433.4	c.1615C>T	p.(Arg539Cys)	Missense	Likely Pathogenic	M	52	Not stated
16844617	2051	MFRP	NM_031433.4	c.642-2A>G	Splice	Splice	Pathogenic	M	33	Not stated
16844617	2051	MFRP	NM_031433.4	c.760_761insTA	p.(Ala254ValfsTer53)	Frameshift	Likely Pathogenic	M	33	Not stated
2808483	2052	MFSD8	NM_152778.3	c.1361T>C	p.(Met454Thr)	Missense	Pathogenic	M	77	Asian - Indian
2808483	2052	MFSD8	NM_152778.3	c.1361T>C	p.(Met454Thr)	Missense	Pathogenic	M	77	Asian - Indian
3301262	2053	MFSD8	NM_152778.3	c.1361T>C	p.(Met454Thr)	Missense	Pathogenic	F	68	Not stated
3301262	2053	MFSD8	NM_152778.3	c.1361T>C	p.(Met454Thr)	Missense	Pathogenic	F	68	Not stated
5581785	2054	MFSD8	NM_152778.3	c.1361T>C	p.(Met454Thr)	Missense	Pathogenic	F	74	Not stated
5581785	2054	MFSD8	NM_152778.3	c.1361T>C	p.(Met454Thr)	Missense	Pathogenic	F	74	Not stated
9018106	2054	MFSD8	NM_152778.3	c.1361T>C	p.(Met454Thr)	Missense	Pathogenic	F	52	Asian - Indian

9018106	2054	MFSD8	NM_152778.3	c.1361T>C	p.(Met454Thr)	Missense	Pathogenic	F	52	Asian - Indian
16432779	2054	MFSD8	NM_152778.3	c.1361T>C	p.(Met454Thr)	Missense	Pathogenic	M	76	Any other ethnic group
16432779	2054	MFSD8	NM_152778.3	c.1361T>C	p.(Met454Thr)	Missense	Pathogenic	M	76	Any other ethnic group
5629014	2055	MFSD8	NM_152778.3	c.1361T>C	p.(Met454Thr)	Missense	Pathogenic	F	47	Asian - Indian
5629014	2055	MFSD8	NM_152778.3	c.1361T>C	p.(Met454Thr)	Missense	Pathogenic	F	47	Asian - Indian
3181261	2056	MFSD8	NM_152778.3	c.1361T>C	p.(Met454Thr)	Missense	Pathogenic	M	79	Asian - Indian
3181261	2056	MFSD8	NM_152778.3	c.1361T>C	p.(Met454Thr)	Missense	Pathogenic	M	79	Asian - Indian
11107263	2057	MFSD8	NM_152778.3	c.416G>T	p.(Arg139Leu)	Missense	Pathogenic	M	36	Not stated
11107263	2057	MFSD8	NM_152778.3	c.509C>T	p.(Ala170Val)	Missense	Variant of Uncertain Significance	M	36	Not stated
12253856	2058	MFSD8	NM_152778.3	c.1361T>C	p.(Met454Thr)	Missense	Pathogenic	M	46	Unknown
12253856	2058	MFSD8	NM_152778.3	c.1361T>C	p.(Met454Thr)	Missense	Pathogenic	M	46	Unknown
13453957	2059	MFSD8	NM_152778.3	c.1361T>C	p.(Met454Thr)	Missense	Pathogenic	F	42	Any other ethnic group
13453957	2059	MFSD8	NM_152778.3	c.1361T>C	p.(Met454Thr)	Missense	Pathogenic	F	42	Any other ethnic group
10940068	2060	MFSD8	NM_152778.3	c.1361T>C	p.(Met454Thr)	Missense	Pathogenic	M	46	Asian - Indian
10940068	2060	MFSD8	NM_152778.3	c.1361T>C	p.(Met454Thr)	Missense	Pathogenic	M	46	Asian - Indian
8665677	2061	MFSD8	NM_152778.3	c.1361T>C	p.(Met454Thr)	Missense	Pathogenic	M	71	Asian - Indian
8665677	2061	MFSD8	NM_152778.3	c.1361T>C	p.(Met454Thr)	Missense	Pathogenic	M	71	Asian - Indian
16112718	2062	MFSD8	NM_152778.3	c.1006G>C	p.(Glu336Gln)	Missense	Likely Pathogenic	M	52	Not stated
16112718	2062	MFSD8	NM_152778.3	c.1254del	p.(Gln418HisfsTer8)	Frameshift	Likely Pathogenic	M	52	Not stated
7507520	2063	MKKS	NM_018848.3	c.896C>T	p.(Pro299Leu)	Missense	Variant of Uncertain Significance	M	25	Asian - Indian
7507520	2063	MKKS	NM_018848.3	c.896C>T	p.(Pro299Leu)	Missense	Variant of Uncertain Significance	M	25	Asian - Indian
7666259	2064	MT-ATP6		m.8672T>C	Mitochondrial	Mitochondrial	Variant of Uncertain Significance	M	59	White - British
10274340	2065	MT-ATP6		m.8993T>G	Mitochondrial	Mitochondrial	Pathogenic	M	39	Not stated
7381275	2066	MT-TL1		m.3243A>G	Mitochondrial	Mitochondrial	Likely Pathogenic	F	57	Not stated
7040326	2067	MT-TL1		m.3243A>G	Mitochondrial	Mitochondrial	Likely Pathogenic	M	75	Asian - Indian
12086612	2068	MT-TL1		m.3243A>G	Mitochondrial	Mitochondrial	Likely Pathogenic	F	58	Asian - Indian
12535256	2069	MT-TL1		m.3243A>G	Mitochondrial	Mitochondrial	Likely Pathogenic	F	80	White - British
12994239	2070	MT-TL1		m.3243A>G	Mitochondrial	Mitochondrial	Likely Pathogenic	F	35	White - British
13111097	2071	MT-TL1		m.3243A>G	Mitochondrial	Mitochondrial	Likely Pathogenic	M	61	Unknown
15959691	2072	MT-TL1		m.3243A>G	Mitochondrial	Mitochondrial	Likely Pathogenic	F	72	Not stated
15759820	2073	MT-TL1		m.3243A>G	Mitochondrial	Mitochondrial	Likely Pathogenic	M	61	Not stated
16398787	2074	MT-TL1		m.3243A>G	Mitochondrial	Mitochondrial	Likely Pathogenic	F	52	White - British
16859877	2075	MT-TL1		m.3243A>G	Mitochondrial	Mitochondrial	Likely Pathogenic	F	41	Not stated
16195199	2076	MT-TL1		m.3243A>G	Mitochondrial	Mitochondrial	Likely Pathogenic	F	41	Black - Caribbean
17401894	2077	MT-TL1		m.3243A>G	Mitochondrial	Mitochondrial	Likely Pathogenic	M	75	Not stated
16166310	2078	MT-TL1		m.3243A>G	Mitochondrial	Mitochondrial	Likely Pathogenic	F	54	White - Other
508220	2079	MYO7A	NM_000260.4	c.2187+1G>A	Splice	Splice	Pathogenic	M	85	White - British
508220	2079	MYO7A	NM_000260.4	c.2187+1G>A	Splice	Splice	Pathogenic	M	85	White - British
791643	2080	MYO7A	NM_000260.4	c.223G>C	p.(Asp75His)	Missense	Variant of Uncertain Significance	F	53	White - British
791643	2080	MYO7A	NM_000260.4	c.6070C>T	p.(Arg2024Ter)	Stopgain	Pathogenic	F	53	White - British
8629039	2081	MYO7A	NM_000260.4	c.635G>A	p.(Arg212His)	Missense	Pathogenic	M	47	White - British
8629039	2081	MYO7A	NM_000260.4	c.640G>A	p.(Gly214Arg)	Missense	Pathogenic	M	47	White - British
1500799	2082	MYO7A	NM_000260.4	c.3504-1G>C	Splice	Splice	Pathogenic	M	56	White - British
1500799	2082	MYO7A	NM_000260.4	c.5573T>C	p.(Leu1858Pro)	Missense	Pathogenic	M	56	White - British
1428482	2083	MYO7A	NM_000260.4	c.3718C>T	p.(Arg1240Trp)	Missense	Pathogenic	F	63	Not stated
1428482	2083	MYO7A	NM_000260.4	c.6377del	p.(Pro2126LeufsTer5)	Frameshift	Pathogenic	F	63	Not stated
1052757	2084	MYO7A	NM_000260.4	c.5824G>T	p.(Gly1942Ter)	Stopgain	Pathogenic	F	47	White - British
1052757	2084	MYO7A	NM_000260.4	c.631A>G	p.(Ser211Gly)	Missense	Pathogenic	F	47	White - British
2593072	2085	MYO7A	NM_000260.4	c.3764del	p.(Lys1255ArgfsTer8)	Frameshift	Pathogenic	M	45	White - British
2593072	2085	MYO7A	NM_000260.4	c.4951G>A	p.(Gly1651Ser)	Missense	Likely Pathogenic	M	45	White - British
3230275	2086	MYO7A	NM_000260.4	c.6572del	p.(Asp2191ValfsTer4)	Frameshift	Variant of Uncertain Significance	M	58	Unknown

3230275	2086	MYO7A	NM_000260.4	c.6572del	p.(Asp2191ValfsTer4)	Frameshift	Variant of Uncertain Significance	M	58	Unknown
3336843	2087	MYO7A	NM_000260.4	c.3764del	p.(Lys1255ArgfsTer8)	Frameshift	Pathogenic	M	38	Not stated
3336843	2087	MYO7A	NM_000260.4	c.77C>A	p.(Ala26Glu)	Missense	Likely Pathogenic	M	38	Not stated
3280045	2088	MYO7A	NM_000260.4	c.2905-1G>A	Splice	Splice	Likely Pathogenic	M	53	Asian - Bangladeshi
3280045	2088	MYO7A	NM_000260.4	c.2905-1G>A	Splice	Splice	Likely Pathogenic	M	53	Asian - Bangladeshi
678432	2089	MYO7A	NM_000260.4	c.133-2A>G	Splice	Splice	Pathogenic	M	50	White - British
678432	2089	MYO7A	NM_000260.4	c.3719G>A	p.(Arg1240Gln)	Missense	Pathogenic	M	50	White - British
3890795	2090	MYO7A	NM_000260.4	c.-48A>G	Regulatory	Regulatory	Variant of Uncertain Significance	M	64	White - British
3890795	2090	MYO7A	NM_000260.4	c.6025del	p.(Ala2009ProfsTer32)	Frameshift	Pathogenic	M	64	White - British
4665744	2091	MYO7A	NM_000260.4	c.1623dup	p.(Lys542GlnfsTer5)	Frameshift	Pathogenic	F	62	White - British
4665744	2091	MYO7A	NM_000260.4	c.2005C>T	p.(Arg669Ter)	Stopgain	Pathogenic	F	62	White - British
3705631	2092	MYO7A	NM_000260.4	c.5944G>A	p.(Gly1982Arg)	Missense	Pathogenic	M	59	Not stated
3705631	2092	MYO7A	NM_000260.4	c.722G>C	p.(Arg241Pro)	Missense	Likely Pathogenic	M	59	Not stated
5125301	2093	MYO7A	NM_000260.4	c.338_348dup	p.(Glu117SerfsTer33)	Frameshift	Pathogenic	F	60	White - British
5125301	2093	MYO7A	NM_000260.4	c.592+1G>T	Splice	Splice	Likely Pathogenic	F	60	White - British
1175327	2094	MYO7A	NM_000260.4	c.3719G>A	p.(Arg1240Gln)	Missense	Pathogenic	M	61	White - British
1175327	2094	MYO7A	NM_000260.4	c.3764del	p.(Lys1255ArgfsTer8)	Frameshift	Pathogenic	M	61	White - British
5918163	2095	MYO7A	NM_000260.4	c.1623dup	p.(Lys542GlnfsTer5)	Frameshift	Pathogenic	M	47	Not stated
5918163	2095	MYO7A	NM_000260.4	c.3764del	p.(Lys1255ArgfsTer8)	Frameshift	Pathogenic	M	47	Not stated
5697369	2096	MYO7A	NM_000260.4	c.2914C>T	p.(Arg972Ter)	Stopgain	Pathogenic	M	41	Not stated
5697369	2096	MYO7A	NM_000260.4	c.2914C>T	p.(Arg972Ter)	Stopgain	Pathogenic	M	41	Not stated
7117823	2097	MYO7A	NM_000260.4	c.3108+1G>A	Splice	Splice	Pathogenic	M	26	Not stated
7117823	2097	MYO7A	NM_000260.4	c.3108+1G>A	Splice	Splice	Pathogenic	M	26	Not stated
6620928	2098	MYO7A	NM_000260.4	c.2005C>T	p.(Arg669Ter)	Stopgain	Pathogenic	F	40	Not stated
6620928	2098	MYO7A	NM_000260.4	c.5944G>A	p.(Gly1982Arg)	Missense	Pathogenic	F	40	Not stated
7365203	2099	MYO7A	NM_000260.4	c.133-2A>G	Splice	Splice	Pathogenic	F	59	White - British
7365203	2099	MYO7A	NM_000260.4	c.4919del	p.(Gly1640AlafsTer5)	Frameshift	Pathogenic	F	59	White - British
7212785	2100	MYO7A	NM_000260.4	c.324C>A	p.(Tyr108Ter)	Stopgain	Pathogenic	F	48	White - British
7212785	2100	MYO7A	NM_000260.4	c.324C>A	p.(Tyr108Ter)	Stopgain	Pathogenic	F	48	White - British
9997546	2101	MYO7A	NM_000260.4	c.1977del	p.(Gly660GlufsTer2)	Frameshift	Pathogenic	F	26	White - British
9997546	2101	MYO7A	NM_000260.4	c.2005C>T	p.(Arg669Ter)	Stopgain	Pathogenic	F	26	White - British
10178468	2102	MYO7A	NM_000260.4	c.1385A>G	p.(Gln462Arg)	Missense	Likely Pathogenic	F	38	White - British
10178468	2102	MYO7A	NM_000260.4	c.6439-2A>G	Splice	Splice	Pathogenic	F	38	White - British
9001110	2103	MYO7A	NM_000260.4	c.3546C>A	p.(Asn1182Lys)	Missense	Pathogenic	M	32	White - British
9001110	2103	MYO7A	NM_000260.4	c.721C>T	p.(Arg241Cys)	Missense	Pathogenic	M	32	White - British
10467638	2104	MYO7A	NM_000260.4	c.397dup	p.(His133ProfsTer7)	Frameshift	Pathogenic	F	22	White - British
10467638	2104	MYO7A	NM_000260.4	c.4919del	p.(Gly1640AlafsTer5)	Frameshift	Pathogenic	F	22	White - British
10586316	2104	MYO7A	NM_000260.4	c.397dup	p.(His133ProfsTer7)	Frameshift	Pathogenic	M	21	White - British
10586316	2104	MYO7A	NM_000260.4	c.4919del	p.(Gly1640AlafsTer5)	Frameshift	Pathogenic	M	21	White - British
11300708	2105	MYO7A	NM_000260.4	c.5886_5888del	p.(Phe1963del)	Inframe indel	Pathogenic	M	43	Not stated
11300708	2105	MYO7A	NM_000260.4	c.5968C>T	p.(Gln1990Ter)	Stopgain	Pathogenic	M	43	Not stated
6255087	2106	MYO7A	NM_000260.4	c.1849T>C	p.(Ser617Pro)	Missense	Likely Pathogenic	F	45	Asian - Other
6255087	2106	MYO7A	NM_000260.4	c.4838del	p.(Asp1613ValfsTer32)	Frameshift	Pathogenic	F	45	Asian - Other
6545923	2107	MYO7A	NM_000260.4	c.147_148del	p.(Pro50AlafsTer89)	Frameshift	Likely Pathogenic	M	35	Any other ethnic group
6545923	2107	MYO7A	NM_000260.4	c.147_148del	p.(Pro50AlafsTer89)	Frameshift	Likely Pathogenic	M	35	Any other ethnic group
7732752	2108	MYO7A	NM_000260.4	c.22dup	p.(Asp8GlyfsTer34)	Frameshift	Likely Pathogenic	M	64	White - British
7732752	2108	MYO7A	NM_000260.4	c.6551C>T	p.(Thr2184Met)	Missense	Pathogenic	M	64	White - British
10327218	2109	MYO7A	NM_000260.4	c.2005C>T	p.(Arg669Ter)	Stopgain	Pathogenic	M	59	Not stated
10327218	2109	MYO7A	NM_000260.4	c.3728C>T	p.(Pro1243Leu)	Missense	Pathogenic	M	59	Not stated
9169292	2110	MYO7A	NM_000260.4	c.1798-3C>G	Splice	Splice	Variant of Uncertain Significance	M	52	Not stated
9169292	2110	MYO7A	NM_000260.4	c.635G>A	p.(Arg212His)	Missense	Pathogenic	M	52	Not stated

8842749	2111	MYO7A	NM_000260.4	c.5648G>A	p.(Arg1883Gln)	Missense	Pathogenic	M	22	Not stated
8842749	2111	MYO7A	NM_000260.4	c.93C>A	p.(Cys31Ter)	Stopgain	Pathogenic	M	22	Not stated
9220616	2112	MYO7A	NM_000260.4	c.5886_5888del	p.(Phe1963del)	Inframe indel	Pathogenic	F	20	Asian - Other
9220616	2112	MYO7A	NM_000260.4	c.5886_5888del	p.(Phe1963del)	Inframe indel	Pathogenic	F	20	Asian - Other
8954469	2113	MYO7A	NM_000260.4	c.1563del	p.(Asp521GlufsTer8)	Frameshift	Pathogenic	M	45	White - British
8954469	2113	MYO7A	NM_000260.4	c.3764del	p.(Lys1255ArgfsTer8)	Frameshift	Pathogenic	M	45	White - British
9220553	2114	MYO7A	NM_000260.4	c.1138G>A	p.(Glu380Lys)	Missense	Variant of Uncertain Significance	M	26	White - British
9220553	2114	MYO7A	NM_000260.4	c.722G>C	p.(Arg241Pro)	Missense	Likely Pathogenic	M	26	White - British
1934169	2115	MYO7A	NM_000260.4	c.1258A>T	p.(Lys420Ter)	Stopgain	Pathogenic	M	46	Asian - Pakistani
1934169	2115	MYO7A	NM_000260.4	c.4838del	p.(Asp1613ValfsTer32)	Frameshift	Pathogenic	M	46	Asian - Pakistani
8657907	2116	MYO7A	NM_000260.4	c.5824G>T	p.(Gly1942Ter)	Stopgain	Pathogenic	F	36	Not stated
8657907	2116	MYO7A	NM_000260.4	c.73G>A	p.(Gly25Arg)	Missense	Pathogenic	F	36	Not stated
3530771	2117	MYO7A	NM_000260.4	c.2904G>T	p.(Glu968Asp)	Missense	Likely Pathogenic	M	36	Not stated
3530771	2117	MYO7A	NM_000260.4	c.4131dup	p.(Gly1378TrpfsTer6)	Frameshift	Likely Pathogenic	M	36	Not stated
7252853	2118	MYO7A	NM_000260.4	c.2476G>A	p.(Ala826Thr)	Missense	Benign	M	59	White - British
7252853	2118	MYO7A	NM_000260.4	c.4293G>A	p.(Trp1431Ter)	Stopgain	Pathogenic	M	59	White - British
12162618	2119	MYO7A	NM_000260.4	c.3014C>T	p.(Ala1005Val)	Missense	Variant of Uncertain Significance	M	46	Any other ethnic group
12162618	2119	MYO7A	NM_000260.4	c.3014C>T	p.(Ala1005Val)	Missense	Variant of Uncertain Significance	M	46	Any other ethnic group
12805953	2120	MYO7A	NM_000260.4	c.3476G>T	p.(Gly1159Val)	Missense	Pathogenic	F	67	Any other ethnic group
12805953	2120	MYO7A	NM_000260.4	c.3728C>T	p.(Pro1243Leu)	Missense	Pathogenic	F	67	Any other ethnic group
9460016	2121	MYO7A	NM_000260.4	c.3764del	p.(Lys1255ArgfsTer8)	Frameshift	Pathogenic	M	31	White - British
9460016	2121	MYO7A	NM_000260.4	c.397C>A	p.(His133Asn)	Missense	Likely Pathogenic	M	31	White - British
4290670	2122	MYO7A	NM_000260.4	c.4838del	p.(Asp1613ValfsTer32)	Frameshift	Pathogenic	F	57	Asian - Indian
4290670	2122	MYO7A	NM_000260.4	c.4838del	p.(Asp1613ValfsTer32)	Frameshift	Pathogenic	F	57	Asian - Indian
14903188	2123	MYO7A	NM_000260.4	c.324C>A	p.(Tyr108Ter)	Stopgain	Pathogenic	F	46	Not stated
14903188	2123	MYO7A	NM_000260.4	c.324C>A	p.(Tyr108Ter)	Stopgain	Pathogenic	F	46	Not stated
14947001	2124	MYO7A	NM_000260.4	c.535_536del	p.(Ser179TrpfsTer7)	Frameshift	Likely Pathogenic	F	22	Any other ethnic group
14947001	2124	MYO7A	NM_000260.4	c.535_536del	p.(Ser179TrpfsTer7)	Frameshift	Likely Pathogenic	F	22	Any other ethnic group
13629006	2125	MYO7A	NM_000260.4	c.133-2A>G	Splice	Splice	Pathogenic	M	49	White - British
13629006	2125	MYO7A	NM_000260.4	c.5886_5888del	p.(Phe1963del)	Inframe indel	Pathogenic	M	49	White - British
3930513	2126	MYO7A	NM_000260.4	c.3508G>A	p.(Glu1170Lys)	Missense	Pathogenic	M	61	Not stated
3930513	2126	MYO7A	NM_000260.4	c.3508G>A	p.(Glu1170Lys)	Missense	Pathogenic	M	61	Not stated
10677848	2127	MYO7A	NM_000260.4	c.722G>C	p.(Arg241Pro)	Missense	Likely Pathogenic	F	28	Not stated
10677848	2127	MYO7A	NM_000260.4	c.722G>C	p.(Arg241Pro)	Missense	Likely Pathogenic	F	28	Not stated
16218082	2128	MYO7A	NM_000260.4	c.397C>G	p.(His133Asp)	Missense	Likely Pathogenic	M	13	Not stated
16218082	2128	MYO7A	NM_000260.4	c.401T>A	p.(Ile134Asn)	Missense	Pathogenic	M	13	Not stated
16059714	2129	MYO7A	NM_000260.4	c.5886_5888del	p.(Phe1963del)	Inframe indel	Pathogenic	F	20	Not stated
16059714	2129	MYO7A	NM_000260.4	c.5886_5888del	p.(Phe1963del)	Inframe indel	Pathogenic	F	20	Not stated
11543559	2130	MYO7A	NM_000260.4	c.5101C>T	p.(Arg1701Ter)	Stopgain	Pathogenic	F	31	Black - African
11543559	2130	MYO7A	NM_000260.4	c.5260C>T	p.(Gln1754Ter)	Stopgain	Likely Pathogenic	F	31	Black - African
17602633	2131	MYO7A	NM_000260.4	c.401T>A	p.(Ile134Asn)	Missense	Pathogenic	M	28	Not stated
17602633	2131	MYO7A	NM_000260.4	c.5730T>A	p.(Asp1910Glu)	Missense	Variant of Uncertain Significance	M	28	Not stated
17632446	2131	MYO7A	NM_000260.4	c.401T>A	p.(Ile134Asn)	Missense	Pathogenic	F	28	Not stated
17632446	2131	MYO7A	NM_000260.4	c.5730T>A	p.(Asp1910Glu)	Missense	Variant of Uncertain Significance	F	28	Not stated
17693997	2132	MYO7A	NM_000260.4	c.1690G>A	p.(Gly564Ser)	Missense	Variant of Uncertain Significance	F	47	Not stated
17693997	2132	MYO7A	NM_000260.4	c.1690G>A	p.(Gly564Ser)	Missense	Variant of Uncertain Significance	F	47	Not stated
17195786	2133	MYO7A	NM_000260.4	c.640G>A	p.(Gly214Arg)	Missense	Pathogenic	F	28	Not stated
17195786	2133	MYO7A	NM_000260.4	c.640G>A	p.(Gly214Arg)	Missense	Pathogenic	F	28	Not stated
17986632	2134	MYO7A	NM_000260.4	c.5229del	p.(Leu1744CysfsTer61)	Frameshift	Pathogenic	F	78	Not stated
17986632	2134	MYO7A	NM_000260.4	c.5886_5888del	p.(Phe1963del)	Inframe indel	Pathogenic	F	78	Not stated
17647601	2135	MYO7A	NM_000260.4	c.1258A>T	p.(Lys420Ter)	Stopgain	Pathogenic	M	12	Any other ethnic group

17647601	2135	MYO7A	NM_000260.4	c.1258A>T	p.(Lys420Ter)	Stopgain	Pathogenic	M	12	Any other ethnic group
347017	2136	MYO7A	NM_000260.4	c.3719G>A	p.(Arg1240Gln)	Missense	Pathogenic	F	65	Unknown
347017	2136	MYO7A	NM_000260.4	c.6577C>T	p.(Leu2193Phe)	Missense	Variant of Uncertain Significance	F	65	Unknown
8707621	2137	MYO7A	NM_000260.4	c.1200+2T>C	Splice	Splice	Likely Pathogenic	F	50	Not stated
8707621	2137	MYO7A	NM_000260.4	c.5944G>A	p.(Gly1982Arg)	Missense	Pathogenic	F	50	Not stated
18523553	2138	MYO7A	NM_000260.4	c.6070C>T	p.(Arg2024Ter)	Stopgain	Pathogenic	F	5	Not stated
18523553	2138	MYO7A	NM_000260.4	c.999T>G	p.(Tyr333Ter)	Stopgain	Pathogenic	F	5	Not stated
18552288	2139	MYO7A	NM_000260.4	c.5743-2A>G	Splice	Splice	Pathogenic	M	27	Asian - Pakistani
18552288	2139	MYO7A	NM_000260.4	c.5743-2A>G	Splice	Splice	Pathogenic	M	27	Asian - Pakistani
3754939	2140	NDP	NM_000266.4	Exon 3 deletion	Deletion	Deletion	Likely Pathogenic	M	30	Not stated
10507860	2141	NDP	NM_000266.4	c.203_225dup	p.(Glu76ThrfsTer36)	Frameshift	Likely Pathogenic	M	15	White - British
12449996	2142	NDP	NM_000266.4	c.109C>T	p.(Arg37Ter)	Stopgain	Likely Pathogenic	M	11	White - British
5785359	2143	NDP	NM_000266.4	c.112C>T	p.(Arg38Cys)	Missense	Pathogenic	M	39	White - British
5704761	2144	NMNAT1	NM_022787.4	c.661dup	p.(Ile221AsnfsTer29)	Frameshift	Likely Pathogenic	M	32	White - British
5704761	2144	NMNAT1	NM_022787.4	c.769G>A	p.(Glu257Lys)	Missense	Pathogenic	M	32	White - British
5064135	2145	NMNAT1	NM_022787.4	c.53A>G	p.(Asn18Ser)	Missense	Pathogenic	F	34	Not stated
5064135	2145	NMNAT1	NM_022787.4	c.769G>A	p.(Glu257Lys)	Missense	Pathogenic	F	34	Not stated
5064149	2145	NMNAT1	NM_022787.4	c.53A>G	p.(Asn18Ser)	Missense	Pathogenic	M	40	Not stated
5064149	2145	NMNAT1	NM_022787.4	c.769G>A	p.(Glu257Lys)	Missense	Pathogenic	M	40	Not stated
7128197	2146	NMNAT1	NM_022787.4	c.466G>C	p.(Gly156Arg)	Missense	Pathogenic	M	22	White - British
7128197	2146	NMNAT1	NM_022787.4	c.769G>A	p.(Glu257Lys)	Missense	Pathogenic	M	22	White - British
8604784	2147	NMNAT1	NM_022787.4	c.364del	p.(Arg122GlyfsTer20)	Frameshift	Pathogenic	M	19	Any other ethnic group
8604784	2147	NMNAT1	NM_022787.4	c.634G>A	p.(Val212Met)	Missense	Likely Pathogenic	M	19	Any other ethnic group
9203669	2148	NMNAT1	NM_022787.4	c.769G>A	p.(Glu257Lys)	Missense	Pathogenic	M	18	Not stated
9203669	2148	NMNAT1	NM_022787.4	c.769G>A	p.(Glu257Lys)	Missense	Pathogenic	M	18	Not stated
9459414	2149	NMNAT1	NM_022787.4	c.293T>G	p.(Val98Gly)	Missense	Pathogenic	F	27	Not stated
9459414	2149	NMNAT1	NM_022787.4	c.37G>A	p.(Ala13Thr)	Missense	Likely Pathogenic	F	27	Not stated
9106796	2150	NMNAT1	NM_022787.4	c.53A>G	p.(Asn18Ser)	Missense	Pathogenic	M	17	Asian - Bangladeshi
9106796	2150	NMNAT1	NM_022787.4	c.53A>G	p.(Asn18Ser)	Missense	Pathogenic	M	17	Asian - Bangladeshi
9559129	2151	NMNAT1	NM_022787.4	c.293T>G	p.(Val98Gly)	Missense	Pathogenic	F	17	Not stated
9559129	2151	NMNAT1	NM_022787.4	c.161C>T	p.(Ala54Val)	Missense	Likely Pathogenic	F	17	Not stated
10682377	2152	NMNAT1	NM_022787.4	c.205A>G	p.(Met69Val)	Missense	Pathogenic	F	18	White - British
10682377	2152	NMNAT1	NM_022787.4	c.769G>A	p.(Glu257Lys)	Missense	Pathogenic	F	18	White - British
11757626	2153	NMNAT1	NM_022787.4	c.59T>A	p.(Ile20Asn)	Missense	Likely Pathogenic	F	14	Unknown
11757626	2153	NMNAT1	NM_022787.4	c.769G>A	p.(Glu257Lys)	Missense	Pathogenic	F	14	Unknown
10855837	2154	NMNAT1	NM_022787.4	c.552A>G	p.(Ile184Met)	Missense	Likely Pathogenic	M	53	Not stated
10855837	2154	NMNAT1	NM_022787.4	c.769G>A	p.(Glu257Lys)	Missense	Pathogenic	M	53	Not stated
11692631	2155	NMNAT1	NM_022787.4	c.155G>T	p.(Gly52Val)	Missense	Likely Pathogenic	F	14	Not stated
11692631	2155	NMNAT1	NM_022787.4	c.736G>C	p.(Glu246Gln)	Missense	Likely Pathogenic	F	14	Not stated
12413946	2156	NMNAT1	NM_022787.4	c.769G>A	p.(Glu257Lys)	Missense	Pathogenic	M	11	White - British
12413946	2156	NMNAT1	NM_022787.4	Exon 4 duplication	Duplication	Duplication	Variant of Uncertain Significance	M	11	White - British
12661340	2157	NMNAT1	NM_022787.4	c.500A>G	p.(Asn167Ser)	Missense	Pathogenic	F	15	Unknown
12661340	2157	NMNAT1	NM_022787.4	c.500A>G	p.(Asn167Ser)	Missense	Pathogenic	F	15	Unknown
12778002	2158	NMNAT1	NM_022787.4	c.485C>A	p.(Ser162Tyr)	Missense	Likely Pathogenic	F	47	Unknown
12778002	2158	NMNAT1	NM_022787.4	c.769G>A	p.(Glu257Lys)	Missense	Pathogenic	F	47	Unknown
11921573	2159	NMNAT1	NM_022787.4	c.25G>A	p.(Val9Met)	Missense	Likely Pathogenic	F	58	Mixed - Other
11921573	2159	NMNAT1	NM_022787.4	c.25G>A	p.(Val9Met)	Missense	Likely Pathogenic	F	58	Mixed - Other
14987839	2160	NMNAT1	NM_022787.4	c.155G>A	p.(Gly52Asp)	Missense	Likely Pathogenic	M	9	Any other ethnic group
14987839	2160	NMNAT1	NM_022787.4	c.155G>A	p.(Gly52Asp)	Missense	Likely Pathogenic	M	9	Any other ethnic group
16065265	2161	NMNAT1	NM_022787.4	c.25G>A	p.(Val9Met)	Missense	Likely Pathogenic	F	17	Not stated
16065265	2161	NMNAT1	NM_022787.4	c.25G>A	p.(Val9Met)	Missense	Likely Pathogenic	F	17	Not stated

16117219	2162	NMNAT1	NM_022787.4	c.53A>G	p.(Asn18Ser)	Missense	Pathogenic	M	13	Not stated
16117219	2162	NMNAT1	NM_022787.4	c.709C>T	p.(Arg237Cys)	Missense	Pathogenic	M	13	Not stated
619975	2163	NR2E3	NM_014249.4	c.119-2A>C	Splice	Splice	Pathogenic	M	59	Not stated
619975	2163	NR2E3	NM_014249.4	c.932G>A	p.(Arg311Gln)	Missense	Pathogenic	M	59	Not stated
4431447	2164	NR2E3	NM_014249.4	c.119-2A>C	Splice	Splice	Pathogenic	M	52	White - British
4431447	2164	NR2E3	NM_014249.4	c.767C>A	p.(Ala256Glu)	Missense	Pathogenic	M	52	White - British
3880890	2165	NR2E3	NM_014249.4	c.119-2A>C	Splice	Splice	Pathogenic	F	69	Not stated
3880890	2165	NR2E3	NM_014249.4	c.119-2A>C	Splice	Splice	Pathogenic	F	69	Not stated
4620692	2166	NR2E3	NM_014249.4	c.1025T>C	p.(Val342Ala)	Missense	Variant of Uncertain Significance	M	55	Not stated
4620692	2166	NR2E3	NM_014249.4	c.119-2A>C	Splice	Splice	Pathogenic	M	55	Not stated
4879923	2167	NR2E3	NM_014249.4	c.119-2A>C	Splice	Splice	Pathogenic	M	57	White - British
4879923	2167	NR2E3	NM_014249.4	c.119-2A>C	Splice	Splice	Pathogenic	M	57	White - British
5634852	2168	NR2E3	NM_014249.4	c.1027G>A	p.(Glu343Lys)	Missense	Variant of Uncertain Significance	F	50	White - British
5634852	2168	NR2E3	NM_014249.4	c.119-2A>C	Splice	Splice	Pathogenic	F	50	White - British
8382842	2169	NR2E3	NM_014249.4	c.767C>A	p.(Ala256Glu)	Missense	Pathogenic	F	22	Not stated
8382842	2169	NR2E3	NM_014249.4	c.994G>A	p.(Glu332Lys)	Missense	Likely Pathogenic	F	22	Not stated
10385150	2170	NR2E3	NM_014249.4	c.1112T>G	p.(Leu371Trp)	Missense	Variant of Uncertain Significance	F	42	Not stated
10385150	2170	NR2E3	NM_014249.4	c.932G>A	p.(Arg311Gln)	Missense	Pathogenic	F	42	Not stated
11050682	2171	NR2E3	NM_014249.4	c.119-2A>C	Splice	Splice	Pathogenic	M	60	White - British
11050682	2171	NR2E3	NM_014249.4	c.908T>C	p.(Leu303Pro)	Missense	Likely Pathogenic	M	60	White - British
11057906	2172	NR2E3	NM_014249.4	c.166G>A	p.(Gly56Arg)	Missense	Pathogenic	F	48	Asian - Other
10116042	2173	NR2E3	NM_014249.4	c.166G>A	p.(Gly56Arg)	Missense	Pathogenic	F	31	Not stated
12526723	2174	NR2E3	NM_014249.4	c.211T>C	p.(Phe71Leu)	Missense	Variant of Uncertain Significance	F	55	White - Other
12526723	2174	NR2E3	NM_014249.4	c.932G>A	p.(Arg311Gln)	Missense	Pathogenic	F	55	White - Other
14972418	2175	NR2E3	NM_014249.4	c.119-2A>C	Splice	Splice	Pathogenic	M	59	Asian - Other
14972418	2175	NR2E3	NM_014249.4	c.119-2A>C	Splice	Splice	Pathogenic	M	59	Asian - Other
14732500	2176	NR2E3	NM_014249.4	c.925C>T	p.(Arg309Trp)	Missense	Likely Pathogenic	F	14	Any other ethnic group
14732500	2176	NR2E3	NM_014249.4	c.925C>T	p.(Arg309Trp)	Missense	Likely Pathogenic	F	14	Any other ethnic group
15610006	2177	NR2E3	NM_014249.4	c.310C>T	p.(Arg104Trp)	Missense	Likely Pathogenic	F	13	Unknown
15610006	2177	NR2E3	NM_014249.4	c.310C>T	p.(Arg104Trp)	Missense	Likely Pathogenic	F	13	Unknown
16445085	2178	NR2E3	NM_014249.4	c.310C>T	p.(Arg104Trp)	Missense	Likely Pathogenic	F	25	Not stated
16445085	2178	NR2E3	NM_014249.4	c.310C>T	p.(Arg104Trp)	Missense	Likely Pathogenic	F	25	Not stated
16900148	2179	NR2E3	NM_014249.4	c.119-2A>C	Splice	Splice	Pathogenic	F	55	White - Other
16900148	2179	NR2E3	NM_014249.4	c.119-2A>C	Splice	Splice	Pathogenic	F	55	White - Other
16706451	2180	NR2E3	NM_014249.4	c.196G>A	p.(Gly66Ser)	Missense	Likely Pathogenic	M	41	White - British
16706451	2180	NR2E3	NM_014249.4	c.932G>A	p.(Arg311Gln)	Missense	Pathogenic	M	41	White - British
17280164	2181	NR2E3	NM_014249.4	c.119-2A>C	Splice	Splice	Pathogenic	M	35	Not stated
17280164	2181	NR2E3	NM_014249.4	c.119-2A>C	Splice	Splice	Pathogenic	M	35	Not stated
17595920	2182	NR2E3	NM_014249.4	c.119-2A>C	Splice	Splice	Pathogenic	F	38	Not stated
17595920	2182	NR2E3	NM_014249.4	c.119-2A>C	Splice	Splice	Pathogenic	F	38	Not stated
18088216	2183	NR2E3	NM_014249.4	c.119-2A>C	Splice	Splice	Pathogenic	F	41	White - Other
18088216	2183	NR2E3	NM_014249.4	c.194_202del	p.(Asn65_Cys67del)	Inframe indel	Likely Pathogenic	F	41	White - Other
18398841	2184	NR2E3	NM_014249.4	c.119-2A>C	Splice	Splice	Pathogenic	F	26	Not stated
18398841	2184	NR2E3	NM_014249.4	c.932G>A	p.(Arg311Gln)	Missense	Pathogenic	F	26	Not stated
18578545	2185	NR2E3	NM_014249.4	c.166G>A	p.(Gly56Arg)	Missense	Pathogenic	F	38	Unknown
18627643	2186	NR2E3	NM_014249.4	c.119-2A>C	Splice	Splice	Pathogenic	F	16	Not stated
18627643	2186	NR2E3	NM_014249.4	c.119-2A>C	Splice	Splice	Pathogenic	F	16	Not stated
13497329	2187	NR2F1	NM_005654.6	c.1115T>C	p.(Leu372Pro)	Missense	Pathogenic	F	30	Not stated
10223835	2188	NR2F1	NM_005654.6	c.1118_1123del	p.(Arg373_Leu374del)	Inframe indel	Variant of Uncertain Significance	F	23	Any other ethnic group
227212	2189	NRL	NM_006177.5	c.148T>A	p.(Ser50Thr)	Missense	Likely Pathogenic	F	49	White - British
2948672	2189	NRL	NM_006177.5	c.148T>A	p.(Ser50Thr)	Missense	Likely Pathogenic	F	47	White - British

1038981	2189	NRL	NM_006177.5	c.148T>A	p.(Ser50Thr)	Missense	Likely Pathogenic	M	69	Not stated
18182653	2189	NRL	NM_006177.5	c.148T>A	p.(Ser50Thr)	Missense	Likely Pathogenic	F	21	Not stated
3249567	2190	NRL	NM_006177.5	c.148T>A	p.(Ser50Thr)	Missense	Likely Pathogenic	M	80	Not stated
15332582	2191	NRL	NM_006177.5	c.149C>T	p.(Ser50Leu)	Missense	Pathogenic	F	40	Not stated
1270009	2192	NYX	NM_022567.2	c.339-353del	p.(Glu114_Ala118del)	Inframe indel	Variant of Uncertain Significance	M	56	White - British
839229	2193	NYX	NM_022567.2	c.339-353del	p.(Glu114_Ala118del)	Inframe indel	Variant of Uncertain Significance	M	87	Black - Caribbean
12702206	2194	NYX	NM_022567.2	c.1308dup	p.(Leu437AlafsTer56)	Frameshift	Likely Pathogenic	M	26	Not stated
11838028	2195	NYX	NM_022567.2	c.936C>G	p.(Asn312Lys)	Missense	Likely Pathogenic	M	13	White - British
12259911	2196	NYX	NM_022567.2	c.523C>T	p.(Pro175Ser)	Missense	Pathogenic	M	17	Not stated
15665614	2197	NYX	NM_022567.2	c.647A>G	p.(Asn216Ser)	Missense	Likely Pathogenic	M	42	White - British
15703799	2198	NYX	NM_022567.2	c.1109G>T	p.(Gly370Val)	Missense	Variant of Uncertain Significance	M	11	Any other ethnic group
16046981	2199	NYX	NM_022567.2	c.647A>C	p.(Asn216Thr)	Missense	Likely Pathogenic	M	10	Not stated
16669071	2200	NYX	NM_022567.2	c.797T>A	p.(Leu266Gln)	Missense	Variant of Uncertain Significance	M	15	Asian - Indian
16901555	2201	NYX	NM_022567.2	c.137T>G	p.(Val46Gly)	Missense	Likely Pathogenic	M	14	Not stated
16441389	2202	NYX	NM_022567.2	Exon 2 deletion	Deletion	Deletion	Likely Pathogenic	M	47	Not stated
4231982	2203	NYX	NM_022567.2	c.293T>C	p.(Leu98Pro)	Missense	Variant of Uncertain Significance	M	30	Mixed - Other
15178064	2204	NYX	NM_022567.2	c.854T>C	p.(Leu285Pro)	Missense	Variant of Uncertain Significance	M	10	Not stated
17542426	2205	NYX	NM_022567.2	c.411_419dup	p.(Arg138_Leu140dup)	Inframe indel	Variant of Uncertain Significance	M	24	Not stated
18195701	2206	NYX	NM_022567.2	c.281del	p.(Arg94ProfsTer47)	Frameshift	Likely Pathogenic	M	15	Not stated
371601	2207	GPR143	NM_000273.3	c.779A>G	p.(Asn260Ser)	Missense	Variant of Uncertain Significance	M	47	White - British
1122645	2208	GPR143	NM_000273.3	c.870_872del	p.(Thr291del)	Inframe indel	Variant of Uncertain Significance	M	50	Not stated
136954	2209	GPR143	NM_000273.3	c.939del	p.(Trp313Ter)	Stopgain	Likely Pathogenic	M	63	Not stated
4876038	2210	GPR143	NM_000273.3	c.3G>T	p.(Met1?)	Start codon	Likely Pathogenic	M	45	White - British
10388230	2211	GPR143	NM_000273.3	c.499del	p.(Leu167CysfsTer51)	Frameshift	Likely Pathogenic	M	15	Asian - Indian
8006431	2212	GPR143	NM_000273.3	c.874T>G	p.(Trp292Gly)	Missense	Likely Pathogenic	M	27	White - British
5664322	2213	GPR143	NM_000273.3	c.874T>G	p.(Trp292Gly)	Missense	Likely Pathogenic	F	28	Not stated
18323346	2213	GPR143	NM_000273.3	c.874T>G	p.(Trp292Gly)	Missense	Likely Pathogenic	M	5	Not stated
7951012	2214	GPR143	NM_000273.3	c.703G>A	p.(Glu235Lys)	Missense	Likely Pathogenic	M	24	Any other ethnic group
7254743	2215	GPR143	NM_000273.3	c.733C>T	p.(Arg245Ter)	Stopgain	Pathogenic	F	25	White - British
10416461	2216	GPR143	NM_000273.3	c.149C>G	p.(Pro50Arg)	Missense	Variant of Uncertain Significance	F	44	Black - African
16908576	2217	GPR143	NM_000273.3	c.691T>C	p.(Tyr231His)	Missense	Variant of Uncertain Significance	F	24	Black - African
16874759	2218	GPR143	NM_000273.3	c.11C>G	p.(Pro4Arg)	Missense	Likely Pathogenic	M	16	Black - Caribbean
18298272	2219	GPR143	NM_000273.3	c.874T>G	p.(Trp292Gly)	Missense	Likely Pathogenic	M	69	Unknown
10909478	2220	OAT	NM_000274.4	c.272G>A	p.(Gly91Glu)	Missense	Likely Pathogenic	F	22	Not stated
10909478	2220	OAT	NM_000274.4	c.272G>A	p.(Gly91Glu)	Missense	Likely Pathogenic	F	22	Not stated
10966150	2221	OAT	NM_000274.4	c.362G>A	p.(Gly121Asp)	Missense	Likely Pathogenic	F	50	Not stated
10966150	2221	OAT	NM_000274.4	c.897C>G	p.(Tyr299Ter)	Stopgain	Pathogenic	F	50	Not stated
8446640	2222	OAT	NM_000274.4	c.1250C>T	p.(Pro417Leu)	Missense	Pathogenic	M	43	Not stated
8446640	2222	OAT	NM_000274.4	c.596C>A	p.(Pro199Gln)	Missense	Pathogenic	M	43	Not stated
5464038	2223	OAT	NM_000274.4	c.152G>A	p.(Gly51Asp)	Missense	Likely Pathogenic	F	27	Asian - Pakistani
5464038	2223	OAT	NM_000274.4	c.152G>A	p.(Gly51Asp)	Missense	Likely Pathogenic	F	27	Asian - Pakistani
13870233	2224	OAT	NM_000274.4	c.533_537del	p.(Trp178Ter)	Stopgain	Pathogenic	F	60	White - British
13870233	2224	OAT	NM_000274.4	c.734A>G	p.(Tyr245Cys)	Missense	Likely Pathogenic	F	60	White - British
55208	2225	TYR	NM_000372.5	c.1118C>A	p.(Thr373Lys)	Missense	Pathogenic	F	68	White - British
55208	2225	TYR	NM_000372.5	c.1217C>T	p.(Pro406Leu)	Missense	Pathogenic	F	68	White - British
14792728	2226	TYR	NM_000372.5	c.1037-7T>A	Splice	Splice	Pathogenic	F	38	White - Other
14792728	2226	TYR	NM_000372.5	c.140G>A	p.(Gly47Asp)	Missense	Pathogenic	F	38	White - Other
3003769	2227	TYR	NM_000372.5	c.1118C>A	p.(Thr373Lys)	Missense	Pathogenic	M	47	White - British
3003769	2227	TYR	NM_000372.5	c.1A>G	p.(Met1?)	Start codon	Likely Pathogenic	M	47	White - British
7225574	2228	TYR	NM_000372.5	c.1205G>A	p.(Arg402Gln)	Missense	Variant of Uncertain Significance	M	28	White - British
7225574	2228	TYR	NM_000372.5	c.575C>A	p.(Ser192Tyr)	Missense	Variant of Uncertain Significance	M	28	White - British

7225574	2228	TYR	NM_000372.5	c.823G>T	p.(Val275Phe)	Missense	Pathogenic	M	28	White - British
7225609	2228	TYR	NM_000372.5	c.1205G>A	p.(Arg402Gln)	Missense	Variant of Uncertain Significance	M	35	White - British
7225609	2228	TYR	NM_000372.5	c.575C>A	p.(Ser192Tyr)	Missense	Variant of Uncertain Significance	M	35	White - British
7225609	2228	TYR	NM_000372.5	c.823G>T	p.(Val275Phe)	Missense	Pathogenic	M	35	White - British
2896354	2229	TYR	NM_000372.5	c.1205G>A	p.(Arg402Gln)	Missense	Variant of Uncertain Significance	F	34	White - British
2896354	2229	TYR	NM_000372.5	c.575C>A	p.(Ser192Tyr)	Missense	Variant of Uncertain Significance	F	34	White - British
2896354	2229	TYR	NM_000372.5	c.1336G>A	p.(Gly446Ser)	Missense	Pathogenic	F	34	White - British
11652143	2230	TYR	NM_000372.5	c.1205G>A	p.(Arg402Gln)	Missense	Variant of Uncertain Significance	F	34	White - British
11652143	2230	TYR	NM_000372.5	c.575C>A	p.(Ser192Tyr)	Missense	Variant of Uncertain Significance	F	34	White - British
8922304	2231	TYR	NM_000372.5	c.1037-1G>A	Splice	Splice	Pathogenic	M	22	Not stated
8922304	2231	TYR	NM_000372.5	c.2T>C	p.(Met1?)	Start codon	Pathogenic	M	22	Not stated
14787247	2232	TYR	NM_000372.5	c.1336G>A	p.(Gly446Ser)	Missense	Pathogenic	M	40	White - British
14787247	2232	TYR	NM_000372.5	c.242C>T	p.(Pro81Leu)	Missense	Pathogenic	M	40	White - British
9519250	2233	TYR	NM_000372.5	c.1118C>A	p.(Thr373Lys)	Missense	Pathogenic	F	20	Not stated
9519250	2233	TYR	NM_000372.5	c.823G>T	p.(Val275Phe)	Missense	Pathogenic	F	20	Not stated
11132015	2233	TYR	NM_000372.5	c.1118C>A	p.(Thr373Lys)	Missense	Pathogenic	M	17	White - Irish
11132015	2233	TYR	NM_000372.5	c.823G>T	p.(Val275Phe)	Missense	Pathogenic	M	17	White - Irish
12620369	2234	TYR	NM_000372.5	c.1037-7T>A	Splice	Splice	Pathogenic	F	11	Not stated
12620369	2234	TYR	NM_000372.5	c.1037-7T>A	Splice	Splice	Pathogenic	F	11	Not stated
17063024	2234	TYR	NM_000372.5	c.1037-7T>A	Splice	Splice	Pathogenic	M	6	Any other ethnic group
17063024	2234	TYR	NM_000372.5	c.1037-7T>A	Splice	Splice	Pathogenic	M	6	Any other ethnic group
17730719	2234	TYR	NM_000372.5	c.1037-7T>A	Splice	Splice	Pathogenic	F	5	White - Other
17730719	2234	TYR	NM_000372.5	c.1037-7T>A	Splice	Splice	Pathogenic	F	5	White - Other
3850027	2235	TYR	NM_000372.5	c.1118C>A	p.(Thr373Lys)	Missense	Pathogenic	M	59	White - Irish
3850027	2235	TYR	NM_000372.5	c.1336G>A	p.(Gly446Ser)	Missense	Pathogenic	M	59	White - Irish
10834207	2236	TYR	NM_000372.5	c.1205G>A	p.(Arg402Gln)	Missense	Variant of Uncertain Significance	M	19	White - British
10834207	2236	TYR	NM_000372.5	c.575C>A	p.(Ser192Tyr)	Missense	Variant of Uncertain Significance	M	19	White - British
10834207	2236	TYR	NM_000372.5	c.896G>A	p.(Arg299His)	Missense	Pathogenic	M	19	White - British
8616985	2236	TYR	NM_000372.5	c.1205G>A	p.(Arg402Gln)	Missense	Variant of Uncertain Significance	F	26	White - British
8616985	2236	TYR	NM_000372.5	c.575C>A	p.(Ser192Tyr)	Missense	Variant of Uncertain Significance	F	26	White - British
8616985	2236	TYR	NM_000372.5	c.896G>A	p.(Arg299His)	Missense	Pathogenic	F	26	White - British
16341359	2237	TYR	NM_000372.5	c.1205G>A	p.(Arg402Gln)	Missense	Variant of Uncertain Significance	F	33	White - Other
16341359	2237	TYR	NM_000372.5	c.242C>T	p.(Pro81Leu)	Missense	Pathogenic	F	33	White - Other
16341359	2237	TYR	NM_000372.5	c.575C>A	p.(Ser192Tyr)	Missense	Variant of Uncertain Significance	F	33	White - Other
16341359	2237	TYR	NM_000372.5	c.575C>A	p.(Ser192Tyr)	Missense	Variant of Uncertain Significance	F	33	White - Other
6640514	2238	TYR	NM_000372.5	c.1099C>T	p.(His367Tyr)	Missense	Pathogenic	F	29	White - Other
6640514	2238	TYR	NM_000372.5	c.1357C>T	p.(Gln453Ter)	Stopgain	Likely Pathogenic	F	29	White - Other
196468	2239	TYR	NM_000372.5	c.1037-1G>A	Splice	Splice	Pathogenic	M	76	White - British
196468	2239	TYR	NM_000372.5	c.1118C>A	p.(Thr373Lys)	Missense	Pathogenic	M	76	White - British
4475232	2240	TYR	NM_000372.5	c.1205G>A	p.(Arg402Gln)	Missense	Variant of Uncertain Significance	M	29	White - British
4475232	2240	TYR	NM_000372.5	c.229C>T	p.(Arg77Trp)	Missense	Likely Pathogenic	M	29	White - British
17296880	2241	TYR	NM_000372.5	c.1205G>A	p.(Arg402Gln)	Missense	Variant of Uncertain Significance	M	6	Any other ethnic group
17296880	2241	TYR	NM_000372.5	c.575C>A	p.(Ser192Tyr)	Missense	Variant of Uncertain Significance	M	6	Any other ethnic group
17296880	2241	TYR	NM_000372.5	c.1036+2T>G	Splice	Splice	Pathogenic	M	6	Any other ethnic group
17464971	2242	TYR	NM_000372.5	c.1037-7T>A	Splice	Splice	Pathogenic	F	16	Asian - Other
17464971	2242	TYR	NM_000372.5	c.996G>A	p.(Met332Ile)	Missense	Pathogenic	F	16	Asian - Other
784881	2243	TYR	NM_000372.5	c.1118C>A	p.(Thr373Lys)	Missense	Pathogenic	F	61	Any other ethnic group
784881	2243	TYR	NM_000372.5	c.823G>T	p.(Val275Phe)	Missense	Pathogenic	F	61	Any other ethnic group
17829293	2244	TYR	NM_000372.5	c.1205G>A	p.(Arg402Gln)	Missense	Variant of Uncertain Significance	M	11	Not stated
17829293	2244	TYR	NM_000372.5	c.575C>A	p.(Ser192Tyr)	Missense	Variant of Uncertain Significance	M	11	Not stated
17829293	2244	TYR	NM_000372.5	c.242C>T	p.(Pro81Leu)	Missense	Pathogenic	M	11	Not stated

11517862	2245	TYR	NM_000372.5	c.1255G>A	p.(Gly419Arg)	Missense	Pathogenic	F	40	Asian - Indian
11517862	2245	TYR	NM_000372.5	c.419T>G	p.(Leu140Ter)	Stopgain	Likely Pathogenic	F	40	Asian - Indian
17760518	2246	TYR	NM_000372.5	c.832C>T	p.(Arg278Ter)	Stopgain	Pathogenic	F	39	Not stated
17760518	2246	TYR	NM_000372.5	c.832C>T	p.(Arg278Ter)	Stopgain	Pathogenic	F	39	Not stated
17291672	2247	TYR	NM_000372.5	c.1205G>A	p.(Arg402Gln)	Missense	Variant of Uncertain Significance	F	34	Not stated
17291672	2247	TYR	NM_000372.5	c.575C>A	p.(Ser192Tyr)	Missense	Variant of Uncertain Significance	F	34	Not stated
17291672	2247	TYR	NM_000372.5	c.823G>T	p.(Val275Phe)	Missense	Pathogenic	F	34	Not stated
18625025	2248	TYR	NM_000372.5	c.1118C>A	p.(Thr373Lys)	Missense	Pathogenic	F	39	Not stated
18625025	2248	TYR	NM_000372.5	c.1118C>A	p.(Thr373Lys)	Missense	Pathogenic	F	39	Not stated
949843	2249	OCA2	NM_000275.3	c.1660T>C	p.(Trp554Arg)	Missense	Likely Pathogenic	F	65	Black - Caribbean
949843	2249	OCA2	NM_000275.3	c.2339G>A	p.(Gly780Asp)	Missense	Pathogenic	F	65	Black - Caribbean
949843	2249	OCA2	NM_000275.3	c.407G>A	p.(Arg136Gln)	Missense	Variant of Uncertain Significance	F	65	Black - Caribbean
845809	2250	OCA2	NM_000275.3	c.1320G>C	p.(Leu440Phe)	Missense	Likely Pathogenic	F	45	White - British
845809	2250	OCA2	NM_000275.3	c.1320G>C	p.(Leu440Phe)	Missense	Likely Pathogenic	F	45	White - British
13977235	2251	OCA2	NM_000275.3	Exon 7 deletion	Deletion	Deletion	Likely Pathogenic	M	38	Not stated
13977235	2251	OCA2	NM_000275.3	Exon 10-15 deletion	Deletion	Deletion	Likely Pathogenic	M	38	Not stated
12166874	2252	OCA2	NM_000275.3	c.1182+1G>A	Splice	Splice	Pathogenic	M	49	Not stated
12166874	2252	OCA2	NM_000275.3	c.2079G>A	p.(Glu693Glu)	Synonymous	Likely Pathogenic	M	49	Not stated
16068835	2253	OCA2	NM_000275.3	c.619_636del	p.(Leu207_Leu212del)	Inframe indel	Pathogenic	F	28	Any other ethnic group
16068835	2253	OCA2	NM_000275.3	c.619_636del	p.(Leu207_Leu212del)	Inframe indel	Pathogenic	F	28	Any other ethnic group
13986055	2254	OCA2	NM_000275.3	c.1320G>C	p.(Leu440Phe)	Missense	Likely Pathogenic	M	9	White - British
13986055	2254	OCA2	NM_000275.3	c.1327G>A	p.(Val443Ile)	Missense	Pathogenic	M	9	White - British
16487344	2255	OCA2	NM_000275.3	c.1286T>C	p.(Leu429Pro)	Missense	Likely Pathogenic	F	7	White - Other
16487344	2255	OCA2	NM_000275.3	c.1327G>A	p.(Val443Ile)	Missense	Pathogenic	F	7	White - Other
17569124	2255	OCA2	NM_000275.3	c.1286T>C	p.(Leu429Pro)	Missense	Likely Pathogenic	M	10	Unknown
17569124	2255	OCA2	NM_000275.3	c.1327G>A	p.(Val443Ile)	Missense	Pathogenic	M	10	Unknown
16071999	2256	OCA2	NM_000275.3	c.1142A>G	p.(Glu381Gly)	Missense	Variant of Uncertain Significance	M	13	Asian - Pakistani
16071999	2256	OCA2	NM_000275.3	c.1142A>G	p.(Glu381Gly)	Missense	Variant of Uncertain Significance	M	13	Asian - Pakistani
16071985	2256	OCA2	NM_000275.3	c.1142A>G	p.(Glu381Gly)	Missense	Variant of Uncertain Significance	M	15	Asian - Pakistani
16071985	2256	OCA2	NM_000275.3	c.1142A>G	p.(Glu381Gly)	Missense	Variant of Uncertain Significance	M	15	Asian - Pakistani
16739127	2257	OCA2	NM_000275.3	c.1327G>A	p.(Val443Ile)	Missense	Pathogenic	F	6	White - British
16739127	2257	OCA2	NM_000275.3	c.619_636del	p.(Leu207_Leu212del)	Inframe indel	Pathogenic	F	6	White - British
3145624	2258	OCA2	NM_000275.3	c.1103C>T	p.(Ala368Val)	Missense	Likely Pathogenic	F	47	Black - Caribbean
3145624	2258	OCA2	NM_000275.3	c.619_636del	p.(Leu207_Leu212del)	Inframe indel	Pathogenic	F	47	Black - Caribbean
3145911	2258	OCA2	NM_000275.3	c.1103C>T	p.(Ala368Val)	Missense	Likely Pathogenic	F	41	Black - Caribbean
3145911	2258	OCA2	NM_000275.3	c.619_636del	p.(Leu207_Leu212del)	Inframe indel	Pathogenic	F	41	Black - Caribbean
16843966	2259	OCA2	NM_000275.3	c.216_222del	p.(Thr73GlyfsTer27)	Frameshift	Likely Pathogenic	F	7	White - British
16843966	2259	OCA2	NM_000275.3	c.593C>T	p.(Pro198Leu)	Missense	Pathogenic	F	7	White - British
16921820	2259	OCA2	NM_000275.3	c.216_222del	p.(Thr73GlyfsTer27)	Frameshift	Likely Pathogenic	F	9	White - British
16921820	2259	OCA2	NM_000275.3	c.593C>T	p.(Pro198Leu)	Missense	Pathogenic	F	9	White - British
746129	2260	OCA2	NM_000275.3	c.1465A>G	p.(Asn489Asp)	Missense	Pathogenic	M	83	White - British
746129	2260	OCA2	NM_000275.3	c.1503+5G>A	Splice	Splice	Pathogenic	M	83	White - British
18217611	2261	OCA2	NM_000275.3	c.1327G>A	p.(Val443Ile)	Missense	Pathogenic	M	28	Unknown
18217611	2261	OCA2	NM_000275.3	c.1465A>G	p.(Asn489Asp)	Missense	Pathogenic	M	28	Unknown
4800158	2262	OPA1	NM_130837.3	c.2661+2T>C	Splice	Splice	Likely Pathogenic	F	33	White - British
4980387	2262	OPA1	NM_130837.3	c.2661+2T>C	Splice	Splice	Likely Pathogenic	M	38	Not stated
4947116	2262	OPA1	NM_130837.3	c.2661+2T>C	Splice	Splice	Likely Pathogenic	M	36	White - British
213268	2263	OPA1	NM_130837.3	c.2873_2876del	p.(Val958GlyfsTer3)	Frameshift	Pathogenic	M	51	White - British
6202335	2264	OPA1	NM_130837.3	Exon 28-29 deletion	Deletion	Deletion	Variant of Uncertain Significance	F	61	White - British
2943548	2265	OPA1	NM_130837.3	c.1943T>C	p.(Leu648Pro)	Missense	Likely Pathogenic	M	34	Not stated
3606546	2265	OPA1	NM_130837.3	c.1943T>C	p.(Leu648Pro)	Missense	Likely Pathogenic	F	64	Not stated

264830	2266	OPA1	NM_130837.3	c.1681+1G>T	Splice	Splice	Pathogenic	M	59	White - British
65036	2267	OPA1	NM_130837.3	c.1673C>A	p.(Thr558Lys)	Missense	Variant of Uncertain Significance	F	52	White - British
4811519	2268	OPA1	NM_130837.3	c.2873_2876del	p.(Val958GlyfsTer3)	Frameshift	Pathogenic	F	28	White - British
4615029	2268	OPA1	NM_130837.3	c.2873_2876del	p.(Val958GlyfsTer3)	Frameshift	Pathogenic	F	64	White - British
12029380	2269	OPA1	NM_130837.3	c.1461_1463del	p.(Ile488del)	Inframe indel	Variant of Uncertain Significance	M	61	Not stated
8132333	2270	OPA1	NM_130837.3	c.949-2A>G	Splice	Splice	Likely Pathogenic	F	35	Not stated
8075367	2271	OPA1	NM_130837.3	c.1188T>G	p.(Phe396Leu)	Missense	Variant of Uncertain Significance	M	25	Not stated
9064166	2272	OPA1	NM_130837.3	c.1035+5G>A	Splice	Splice	Pathogenic	F	41	White - British
3316620	2273	OPA1	NM_130837.3	c.678+2T>G	Splice	Splice	Pathogenic	F	59	Not stated
10217773	2274	OPA1	NM_130837.3	c.1035+5G>A	Splice	Splice	Pathogenic	M	25	Not stated
10217787	2274	OPA1	NM_130837.3	c.1035+5G>A	Splice	Splice	Pathogenic	M	29	Not stated
11029892	2275	OPA1	NM_130837.3	c.1377+1G>A	Splice	Splice	Likely Pathogenic	F	33	Any other ethnic group
12377273	2276	OPA1	NM_130837.3	c.2873_2876del	p.(Val997GlufsTer25)	Frameshift	Pathogenic	M	21	Unknown
12833743	2277	OPA1	NM_130837.3	c.2890_2891insTTA	p.(Lys963_Asn964insIle)	Inframe indel	Variant of Uncertain Significance	F	53	White - British
500625	2278	OPA1	NM_130837.3	c.1499G>A	p.(Arg500His)	Missense	Pathogenic	M	54	Any other ethnic group
12948564	2279	OPA1	NM_130837.3	c.1574A>G	p.(Asp525Gly)	Missense	Variant of Uncertain Significance	F	66	White - British
13251783	2279	OPA1	NM_130837.3	c.1574A>G	p.(Asp525Gly)	Missense	Variant of Uncertain Significance	M	38	Not stated
12942733	2280	OPA1	NM_130837.3	c.2873_2876del	p.(Val958GlyfsTer3)	Frameshift	Pathogenic	F	44	White - British
7588965	2281	OPA1	NM_130837.3	c.1377+1G>A	Splice	Splice	Likely Pathogenic	M	33	White - British
10446778	2282	OPA1	NM_130837.3	c.2650G>A	p.(Asp884Asn)	Missense	Variant of Uncertain Significance	F	46	Any other ethnic group
13215166	2282	OPA1	NM_130837.3	c.2650G>A	p.(Asp884Asn)	Missense	Variant of Uncertain Significance	F	74	Not stated
12379534	2283	OPA1	NM_130837.3	c.2873_2876del	p.(Val958GlyfsTer3)	Frameshift	Pathogenic	M	44	Unknown
12705930	2284	OPA1	NM_130837.3	c.2873_2876del	p.(Val958GlyfsTer3)	Frameshift	Pathogenic	F	34	Not stated
13772492	2284	OPA1	NM_130837.3	c.2873_2876del	p.(Val958GlyfsTer3)	Frameshift	Pathogenic	F	31	Unknown
13311227	2285	OPA1	NM_130837.3	c.1377+2dup	Splice	Splice	Likely Pathogenic	M	16	White - British
12998110	2286	OPA1	NM_130837.3	c.1728_1734del	p.(Glu576AspfsTer7)	Frameshift	Pathogenic	M	25	White - British
6790727	2287	OPA1	NM_130837.3	c.1035+5G>A	Splice	Splice	Pathogenic	M	31	White - British
7081493	2288	OPA1	NM_130837.3	c.1377+5G>A	Splice	Splice	Likely Pathogenic	F	27	Any other ethnic group
13651644	2289	OPA1	NM_130837.3	c.2873_2876del	p.(Val958GlyfsTer3)	Frameshift	Pathogenic	F	67	Unknown
6655522	2290	OPA1	NM_130837.3	c.1352T>G	p.(Leu451Arg)	Missense	Pathogenic	F	51	Not stated
8382954	2291	OPA1	NM_130837.3	c.2873_2876del	p.(Val958GlyfsTer3)	Frameshift	Pathogenic	M	55	White - British
13386526	2292	OPA1	NM_130837.3	c.1036-1G>T	Splice	Splice	Pathogenic	F	43	Asian - Indian
13676480	2293	OPA1	NM_130837.3	Exon 20-23 duplication	Duplication	Duplication	Likely Pathogenic	F	36	Not stated
14778826	2294	OPA1	NM_130837.3	c.799A>T	p.(Lys267Ter)	Stopgain	Likely Pathogenic	F	29	Unknown
14901431	2295	OPA1	NM_130837.3	c.2267_2268del	p.(Lys756ArgfsTer4)	Frameshift	Likely Pathogenic	F	36	Unknown
14873389	2296	OPA1	NM_130837.3	c.2983+5G>A	Splice	Splice	Variant of Uncertain Significance	M	56	Not stated
8420978	2297	OPA1	NM_130837.3	c.2521-1G>T	Splice	Splice	Likely Pathogenic	F	70	Not stated
14934807	2298	OPA1	NM_130837.3	c.1378-2A>G	Splice	Splice	Likely Pathogenic	F	21	Not stated
14778707	2299	OPA1	NM_130837.3	c.2873_2876del	p.(Val958GlyfsTer3)	Frameshift	Pathogenic	M	27	Unknown
15116380	2300	OPA1	NM_130837.3	c.1230+2T>C	Splice	Splice	Likely Pathogenic	F	15	White - British
7921486	2301	OPA1	NM_130837.3	c.2873_2876del	p.(Val958GlyfsTer3)	Frameshift	Pathogenic	M	27	White - British
8402421	2301	OPA1	NM_130837.3	c.2873_2876del	p.(Val958GlyfsTer3)	Frameshift	Pathogenic	M	25	Not stated
14853607	2302	OPA1	NM_130837.3	c.2749dup	p.(Tyr917LeufsTer10)	Frameshift	Likely Pathogenic	F	26	Not stated
14813042	2303	OPA1	NM_130837.3	c.2873_2876del	p.(Val958GlyfsTer3)	Frameshift	Pathogenic	F	78	Unknown
15678368	2304	OPA1	NM_130837.3	c.1144del	p.(Val382LeufsTer3)	Frameshift	Likely Pathogenic	F	63	Any other ethnic group
15744868	2305	OPA1	NM_130837.3	c.1064G>A	p.(Gly355Glu)	Missense	Likely Pathogenic	M	46	Any other ethnic group
9320583	2306	OPA1	NM_130837.3	c.2041G>T	p.(Glu681Ter)	Stopgain	Likely Pathogenic	M	32	White - British
13310233	2307	OPA1	NM_130837.3	c.304dup	p.(Tyr102LeufsTer19)	Frameshift	Likely Pathogenic	F	79	White - Other
16447080	2307	OPA1	NM_130837.3	c.304dup	p.(Tyr102LeufsTer19)	Frameshift	Likely Pathogenic	F	47	Not stated
11639683	2308	OPA1	NM_130837.3	c.1363C>G	p.(Pro455Ala)	Missense	Likely Pathogenic	M	15	Asian - Bangladeshi
318030	2309	OPA1	NM_130837.3	c.2873_2876del	p.(Val958GlyfsTer3)	Frameshift	Pathogenic	F	72	Not stated

15103388	2310	OPA1	NM_130837.3	c.2873_2876del	p.(Val958GlyfsTer3)	Frameshift	Pathogenic	M	44	White - Irish
12105596	2311	OPA1	NM_130837.3	c.1183del	p.(Leu395TyrfsTer20)	Frameshift	Likely Pathogenic	M	75	White - British
11866924	2312	OPA1	NM_130837.3	c.2483dup	p.(Arg829GlufsTer17)	Frameshift	Likely Pathogenic	M	27	White - British
16900106	2313	OPA1	NM_130837.3	c.2873_2876del	p.(Val958GlyfsTer3)	Frameshift	Pathogenic	M	59	Not stated
12404195	2314	OPA1	NM_130837.3	c.670_672delinsA	p.(Phe224LysfsTer7)	Frameshift	Likely Pathogenic	M	17	Any other ethnic group
17306484	2315	OPA1	NM_130837.3	c.2760del	p.(Asp920GlufsTer4)	Frameshift	Likely Pathogenic	M	68	White - British
14746717	2316	OPA1	NM_130837.3	c.2179-7A>G	Splice	Splice	Variant of Uncertain Significance	M	12	Not stated
17220804	2316	OPA1	NM_130837.3	c.2179-7A>G	Splice	Splice	Variant of Uncertain Significance	F	43	Not stated
10100089	2317	OPA1	NM_130837.3	c.628del	p.(Ala210ArgfsTer73)	Frameshift	Likely Pathogenic	M	34	Asian - Indian
12488678	2318	OPA1	NM_130837.3	c.2778+1G>A	Splice	Splice	Likely Pathogenic	M	59	Not stated
16213308	2319	OPA1	NM_130837.3	c.1681+1G>T	Splice	Splice	Pathogenic	F	56	White - British
11089987	2320	OPA1	NM_130837.3	c.2908G>T	p.(Glu970Ter)	Stopgain	Likely Pathogenic	M	27	Any other ethnic group
11089987	2320	OPA1	NM_130837.3	c.2959C>A	p.(Arg987Ser)	Missense	Variant of Uncertain Significance	M	27	Any other ethnic group
10967606	2321	OPA1	NM_130837.3	c.800_801del	p.(Lys267ArgfsTer4)	Frameshift	Pathogenic	F	19	Any other ethnic group
13691964	2322	OPA1	NM_130837.3	c.112C>T	p.(Arg38Ter)	Stopgain	Pathogenic	M	14	Not stated
17706345	2323	OPA1	NM_130837.3	c.357del	p.(Phe119LeufsTer7)	Frameshift	Pathogenic	M	23	Not stated
11258988	2324	OPA1	NM_130837.3	c.1377+1G>T	Splice	Splice	Pathogenic	M	20	Unknown
17904396	2325	OPA1	NM_130837.3	c.2990_2993del	p.(Val997GlufsTer25)	Frameshift	Pathogenic	M	11	Not stated
16689595	2326	OPA1	NM_130837.3	c.1036-1G>C	Splice	Splice	Likely Pathogenic	M	48	Any other ethnic group
2275090	2327	OPA1	NM_130837.3	c.2661+1G>A	Splice	Splice	Pathogenic	M	52	Not stated
17963812	2328	OPA1	NM_130837.3	c.2873_2876del	p.(Val958GlyfsTer3)	Frameshift	Pathogenic	M	51	Not stated
17540494	2329	OPA1	NM_130837.3	c.1377+1G>A	Splice	Splice	Likely Pathogenic	M	47	Unknown
17387607	2330	OPA1	NM_130837.3	c.1608+1G>A	Splice	Splice	Likely Pathogenic	M	20	Any other ethnic group
16163279	2331	OPA1	NM_130837.3	c.2873_2876del	p.(Val958GlyfsTer3)	Frameshift	Pathogenic	F	38	Not stated
18102293	2332	OPA1	NM_130837.3	c.2209del	p.(Ser737ProfsTer4)	Frameshift	Likely Pathogenic	M	39	Not stated
18352011	2333	OPA1	NM_130837.3	c.901_902del	p.(Leu301SerfsTer6)	Frameshift	Likely Pathogenic	F	32	Not stated
17702215	2334	OPA1	NM_130837.3	c.1681+1G>T	Splice	Splice	Pathogenic	F	52	Unknown
11905816	2335	OPN1LW	NM_020061.6	c.22del	p.(Gln8LysfsTer53)	Frameshift	Likely Pathogenic	M	57	Asian - Indian
16708943	2336	OPN1LW	NM_020061.6	c.602C>T	p.(Thr201Ile)	Missense	Variant of Uncertain Significance	M	32	White - British
16263792	2337	PAX2	NM_003987.5	c.343C>T	p.(Arg115Ter)	Stopgain	Pathogenic	F	34	Not stated
18572077	2338	PAX2	NM_003987.5	c.805dup	p.(Glu269GlyfsTer7)	Frameshift	Likely Pathogenic	F	46	Not stated
6059843	2339	PAX6	NM_000280.5	c.917-1G>A	Splice	Splice	Pathogenic	F	25	Asian - Pakistani
12295310	2340	PAX6	NM_000280.5	c.775dup	p.(Ser259PhefsTer2)	Frameshift	Pathogenic	F	12	Unknown
1585954	2340	PAX6	NM_000280.5	c.775dup	p.(Ser259PhefsTer2)	Frameshift	Pathogenic	M	47	White - British
1185260	2340	PAX6	NM_000280.5	c.775dup	p.(Ser259PhefsTer2)	Frameshift	Pathogenic	F	61	Unknown
98853	2341	PAX6	NM_000280.5	c.191G>T	p.(Gly64Val)	Missense	Pathogenic	F	50	White - British
8643235	2342	PAX6	NM_000280.5	c.214G>A	p.(Gly72Ser)	Missense	Pathogenic	F	22	White - British
1755753	2342	PAX6	NM_000280.5	c.214G>A	p.(Gly72Ser)	Missense	Pathogenic	F	45	White - British
2758118	2343	PAX6	NM_000280.5	c.115_116dup	p.(Cys40ArgfsTer15)	Frameshift	Pathogenic	F	53	White - British
2861529	2344	PAX6	NM_000280.5	c.106G>A	p.(Gly36Arg)	Missense	Likely Pathogenic	F	35	White - British
434055	2345	PAX6	NM_000280.5	c.1035_1048del	p.(Pro346AspfsTer20)	Frameshift	Likely Pathogenic	F	74	Any other ethnic group
1810962	2345	PAX6	NM_000280.5	c.1035_1048del	p.(Pro346AspfsTer20)	Frameshift	Likely Pathogenic	M	39	Not stated
828372	2346	PAX6	NM_000280.5	c.1268A>T	p.(Ter423Leu)	Stopgain	Pathogenic	M	43	Not stated
3024951	2347	PAX6	NM_000280.5	Whole gene deletion	Deletion	Deletion	Likely Pathogenic	F	34	White - British
5192249	2348	PAX6	NM_000280.5	c.683-6T>A	Splice	Splice	Variant of Uncertain Significance	M	26	White - British
3428144	2348	PAX6	NM_000280.5	c.683-6T>A	Splice	Splice	Variant of Uncertain Significance	F	32	Not stated
6360115	2349	PAX6	NM_000280.5	c.164A>G	p.(Lys55Arg)	Missense	Likely Pathogenic	M	36	White - British
2434865	2350	PAX6	NM_000280.5	c.718C>T	p.(Arg240Ter)	Stopgain	Pathogenic	F	55	White - British
7100680	2350	PAX6	NM_000280.5	c.718C>T	p.(Arg240Ter)	Stopgain	Pathogenic	F	26	White - British
7100715	2350	PAX6	NM_000280.5	c.718C>T	p.(Arg240Ter)	Stopgain	Pathogenic	M	30	White - British
7984633	2351	PAX6	NM_000280.5	c.1061_1070del	p.(Tyr354CysfsTer8)	Frameshift	Pathogenic	M	37	White - British

6192773	2352	PAX6	NM_000280.5	c.197T>A	p.(Ile66Asn)	Missense	Likely Pathogenic	M	34	White - British
13188335	2352	PAX6	NM_000280.5	c.197T>A	p.(Ile66Asn)	Missense	Likely Pathogenic	M	10	Not stated
8921002	2353	PAX6	NM_000280.5	c.345_351dup	p.(Pro118Ter)	Stopgain	Pathogenic	F	18	White - British
8644537	2354	PAX6	NM_000280.5	c.718C>T	p.(Arg240Ter)	Stopgain	Pathogenic	M	52	White - British
12717116	2354	PAX6	NM_000280.5	c.718C>T	p.(Arg240Ter)	Stopgain	Pathogenic	F	76	Unknown
8142931	2354	PAX6	NM_000280.5	c.718C>T	p.(Arg240Ter)	Stopgain	Pathogenic	M	48	White - British
1194500	2355	PAX6	NM_000280.5	c.718C>T	p.(Arg240Ter)	Stopgain	Pathogenic	M	44	Not stated
11682418	2356	PAX6	NM_000280.5	c.607C>T	p.(Arg203Ter)	Stopgain	Pathogenic	F	13	White - British
9074561	2357	PAX6	NM_000280.5	c.1252_1266delinsACAGTAAA	p.(Trp418ThrfsTer105)	Frameshift	Pathogenic	M	40	White - British
17686906	2358	PAX6	NM_000280.5	c.107G>A	p.(Gly36Glu)	Missense	Pathogenic	M	5	Not stated
13887621	2359	PAX6	NM_000280.5	c.114dup	p.(Pro39AlafsTer17)	Frameshift	Pathogenic	M	12	White - British
13061215	2360	PAX6	NM_000280.5	c.214G>T	p.(Gly72Cys)	Missense	Pathogenic	F	22	White - British
4043164	2361	PAX6	NM_000280.5	c.115_116dup	p.(Cys40ArgfsTer15)	Frameshift	Pathogenic	M	30	White - British
18199019	2361	PAX6	NM_000280.5	c.115_116dup	p.(Cys40ArgfsTer15)	Frameshift	Pathogenic	M	4	Not stated
12482539	2362	PAX6	NM_000280.5	c.1184-1G>C	Splice	Splice	Pathogenic	M	35	Any other ethnic group
16109694	2363	PAX6	NM_000280.5	c.112del	p.(Arg38GlyfsTer16)	Frameshift	Pathogenic	M	29	Mixed - White and Asian
6662949	2364	PAX6	NM_000280.5	c.1253_1262del	p.(Trp418TyrfsTer104)	Frameshift	Pathogenic	M	23	Not stated
16699731	2364	PAX6	NM_000280.5	c.1253_1262del	p.(Trp418TyrfsTer104)	Frameshift	Pathogenic	F	65	Not stated
108317	2365	PAX6	NM_000280.5	c.151G>A	p.(Gly51Arg)	Missense	Pathogenic	F	71	White - Other
12833498	2366	PAX6	NM_000280.5	c.357+1G>A	Splice	Splice	Pathogenic	M	17	Unknown
11857390	2366	PAX6	NM_000280.5	c.357+1G>A	Splice	Splice	Pathogenic	F	75	Not stated
17875850	2366	PAX6	NM_000280.5	c.357+1G>A	Splice	Splice	Pathogenic	F	51	Not stated
17618873	2367	PAX6	NM_000280.5	c.925_926del	p.(Phe309HisfsTer31)	Frameshift	Pathogenic	M	7	Any other ethnic group
16924830	2368	PAX6	NM_000280.5	c.163A>T	p.(Lys55Ter)	Stopgain	Pathogenic	M	41	Not stated
13785498	2369	PAX6	NM_000280.5	c.112C>G	p.(Arg38Gly)	Missense	Pathogenic	M	25	Black - African
17204466	2369	PAX6	NM_000280.5	c.112C>G	p.(Arg38Gly)	Missense	Pathogenic	F	23	Not stated
8338994	2369	PAX6	NM_000280.5	c.112C>G	p.(Arg38Gly)	Missense	Pathogenic	F	19	Not stated
9544877	2370	PAX6	NM_000280.5	c.959_960del	p.(Thr320SerfsTer20)	Frameshift	Likely Pathogenic	M	49	White - British
13386554	2371	PAX6	NM_000280.5	c.1268A>T	p.(Ter423Leu)	Stopgain	Pathogenic	F	24	Asian - Other
8385747	2372	PAX6	NM_000280.5	c.115_116dup	p.(Cys40ArgfsTer15)	Frameshift	Pathogenic	M	19	Not stated
5040727	2373	PAX6	NM_000280.5	c.580G>T	p.(Gly194Ter)	Stopgain	Likely Pathogenic	F	27	White - British
796116	2374	PAX6	NM_000280.5	c.331dup	p.(Val111GlyfsTer6)	Frameshift	Pathogenic	F	67	Any other ethnic group
14781850	2374	PAX6	NM_000280.5	c.331dup	p.(Val111GlyfsTer6)	Frameshift	Pathogenic	F	26	Any other ethnic group
14306809	2375	PAX6	NM_000280.5	c.1268A>T	p.(Ter423Leu)	Stopgain	Pathogenic	F	51	Black - Other
950172	2376	PCDH15	NM_033056.4	c.3501+2T>C	Splice	Splice	Pathogenic	M	57	Not stated
950172	2376	PCDH15	NM_033056.4	c.3501+2T>C	Splice	Splice	Pathogenic	M	57	Not stated
711052	2377	PCDH15	NM_033056.4	Exon 10 deletion	Deletion	Deletion	Likely Pathogenic	F	53	White - British
711052	2377	PCDH15	NM_033056.4	Exon 10 deletion	Deletion	Deletion	Likely Pathogenic	F	53	White - British
711059	2377	PCDH15	NM_033056.4	Exon 10 deletion	Deletion	Deletion	Likely Pathogenic	F	55	Not stated
711059	2377	PCDH15	NM_033056.4	Exon 10 deletion	Deletion	Deletion	Likely Pathogenic	F	55	Not stated
6969367	2378	PCDH15	NM_033056.4	c.3717+1G>A	Splice	Splice	Pathogenic	M	35	Any other ethnic group
6969367	2378	PCDH15	NM_033056.4	Multiexon deletion	Deletion	Deletion	Likely Pathogenic	M	35	Any other ethnic group
1900898	2379	PCDH15	NM_033056.4	c.4202+5G>A	Splice	Splice	Variant of Uncertain Significance	M	52	White - British
1900898	2379	PCDH15	NM_033056.4	c.876+2T>A	Splice	Splice	Likely Pathogenic	M	52	White - British
8465267	2380	PCDH15	NM_033056.4	c.2825del	p.(Gly942ValfsTer22)	Frameshift	Pathogenic	F	25	Not stated
8465267	2380	PCDH15	NM_033056.4	c.2825del	p.(Gly942ValfsTer22)	Frameshift	Pathogenic	F	25	Not stated
13100009	2381	PCDH15	NM_033056.4	c.2825del	p.(Gly942ValfsTer22)	Frameshift	Pathogenic	F	27	Unknown
13100009	2381	PCDH15	NM_033056.4	Exon 1 deletion	Deletion	Deletion	Likely Pathogenic	F	27	Unknown
4856690	2382	PCDH15	NM_033056.4	c.1806T>G	p.(Tyr602Ter)	Stopgain	Pathogenic	F	70	Not stated
4856690	2382	PCDH15	NM_033056.4	c.3501+2T>C	Splice	Splice	Pathogenic	F	70	Not stated
14781591	2383	PCDH15	NM_033056.4	c.2624C>T	p.(Ser875Leu)	Missense	Variant of Uncertain Significance	F	48	White - British

14781591	2383	PCDH15	NM_033056.4	Exon 2 deletion	Deletion	Deletion	Likely Pathogenic	F	48	White - British
18555473	2384	PCDH15	NM_033056.4	c.1997+1G>A	Splice	Splice	Pathogenic	F	72	Not stated
18555473	2384	PCDH15	NM_033056.4	c.2687dup	p.(Glu897ArgfsTer4)	Frameshift	Likely Pathogenic	F	72	Not stated
18523707	2385	PCDH15	NM_033056.4	c.3358C>T	p.(Arg1120Ter)	Stopgain	Pathogenic	M	20	White - British
18523707	2385	PCDH15	NM_033056.4	Exon 2-9 deletion	Deletion	Deletion	Likely Pathogenic	M	20	White - British
6805910	2386	PDE6A	NM_000440.3	c.1703G>A	p.(Gly568Glu)	Missense	Variant of Uncertain Significance	M	77	White - British
6805910	2386	PDE6A	NM_000440.3	c.1705C>A	p.(Gln569Lys)	Missense	Pathogenic	M	77	White - British
140447	2386	PDE6A	NM_000440.3	c.1703G>A	p.(Gly568Glu)	Missense	Variant of Uncertain Significance	F	84	Not stated
140447	2386	PDE6A	NM_000440.3	c.1705C>A	p.(Gln569Lys)	Missense	Pathogenic	F	84	Not stated
3218067	2387	PDE6A	NM_000440.3	c.1630C>T	p.(Arg544Trp)	Missense	Likely Pathogenic	M	72	Asian - Indian
3218067	2387	PDE6A	NM_000440.3	c.1630C>T	p.(Arg544Trp)	Missense	Likely Pathogenic	M	72	Asian - Indian
12611829	2388	PDE6A	NM_000440.3	c.1957C>T	p.(Arg653Ter)	Stopgain	Pathogenic	F	23	Not stated
12611829	2388	PDE6A	NM_000440.3	c.1957C>T	p.(Arg653Ter)	Stopgain	Pathogenic	F	23	Not stated
12177514	2389	PDE6A	NM_000440.3	c.1630C>T	p.(Arg544Trp)	Missense	Likely Pathogenic	F	36	Asian - Indian
12177514	2389	PDE6A	NM_000440.3	c.769C>T	p.(Arg257Ter)	Stopgain	Pathogenic	F	36	Asian - Indian
13548464	2390	PDE6A	NM_000440.3	c.1957C>T	p.(Arg653Ter)	Stopgain	Pathogenic	M	37	Any other ethnic group
13548464	2390	PDE6A	NM_000440.3	c.1957C>T	p.(Arg653Ter)	Stopgain	Pathogenic	M	37	Any other ethnic group
13498582	2391	PDE6A	NM_000440.3	c.650_651dup	p.(Ala218LeufsTer4)	Frameshift	Likely Pathogenic	F	27	Asian - Pakistani
13498582	2391	PDE6A	NM_000440.3	c.650_651dup	p.(Ala218LeufsTer4)	Frameshift	Likely Pathogenic	F	27	Asian - Pakistani
14930236	2392	PDE6A	NM_000440.3	c.2327del	p.(Phe776SerfsTer45)	Frameshift	Likely Pathogenic	F	43	White - Other
14930236	2392	PDE6A	NM_000440.3	c.2327del	p.(Phe776SerfsTer45)	Frameshift	Likely Pathogenic	F	43	White - Other
14942010	2393	PDE6A	NM_000440.3	c.1620G>A	p.(Glu540Glu)	Synonymous	Pathogenic	M	31	Not stated
14942010	2393	PDE6A	NM_000440.3	c.2274+2_2274+3del	Splice	Splice	Variant of Uncertain Significance	M	31	Not stated
15356662	2394	PDE6A	NM_000440.3	c.1966G>T	p.(Glu656Ter)	Stopgain	Pathogenic	F	38	White - Other
15356662	2394	PDE6A	NM_000440.3	c.2027+5G>T	Splice	Splice	Variant of Uncertain Significance	F	38	White - Other
16233272	2394	PDE6A	NM_000440.3	c.1966G>T	p.(Glu656Ter)	Stopgain	Pathogenic	F	38	Any other ethnic group
16233272	2394	PDE6A	NM_000440.3	c.2027+5G>T	Splice	Splice	Variant of Uncertain Significance	F	38	Any other ethnic group
15745029	2395	PDE6A	NM_000440.3	c.1630C>T	p.(Arg544Trp)	Missense	Likely Pathogenic	F	32	Any other ethnic group
15745029	2395	PDE6A	NM_000440.3	c.769C>T	p.(Arg257Ter)	Stopgain	Pathogenic	F	32	Any other ethnic group
16407117	2396	PDE6A	NM_000440.3	c.304C>A	p.(Arg102Ser)	Missense	Pathogenic	M	23	White - British
16407117	2396	PDE6A	NM_000440.3	c.823_824del	p.(Tyr275LeufsTer15)	Frameshift	Likely Pathogenic	M	23	White - British
2657395	2397	PDE6B	NM_000283.4	c.1923_1969delinsTCTGGG	p.(Asn643GlyfsTer29)	Frameshift	Pathogenic	M	58	White - British
2657395	2397	PDE6B	NM_000283.4	c.1923_1969delinsTCTGGG	p.(Asn643GlyfsTer29)	Frameshift	Pathogenic	M	58	White - British
612695	2398	PDE6B	NM_000283.4	c.1243G>A	p.(Glu415Lys)	Missense	Variant of Uncertain Significance	F	57	Not stated
612695	2398	PDE6B	NM_000283.4	c.1859A>G	p.(His620Arg)	Missense	Variant of Uncertain Significance	F	57	Not stated
5812295	2399	PDE6B	NM_000283.4	c.1580T>C	p.(Leu527Pro)	Missense	Pathogenic	M	66	White - British
5812295	2399	PDE6B	NM_000283.4	c.1678C>T	p.(Arg560Cys)	Missense	Pathogenic	M	66	White - British
2614723	2400	PDE6B	NM_000283.4	c.1485dup	p.(Pro496AlafsTer5)	Frameshift	Pathogenic	M	69	Any other ethnic group
2614723	2400	PDE6B	NM_000283.4	c.1485dup	p.(Pro496AlafsTer5)	Frameshift	Pathogenic	M	69	Any other ethnic group
5898297	2401	PDE6B	NM_000283.4	c.1860del	p.(His620GlnfsTer23)	Frameshift	Pathogenic	F	55	Mixed - White and Asian
5898297	2401	PDE6B	NM_000283.4	c.1860del	p.(His620GlnfsTer23)	Frameshift	Pathogenic	F	55	Mixed - White and Asian
5060068	2402	PDE6B	NM_000283.4	c.2401C>T	p.(Gln801Ter)	Stopgain	Pathogenic	F	36	Asian - Indian
5060068	2402	PDE6B	NM_000283.4	c.2401C>T	p.(Gln801Ter)	Stopgain	Pathogenic	F	36	Asian - Indian
8089857	2403	PDE6B	NM_000283.4	c.1580T>C	p.(Leu527Pro)	Missense	Pathogenic	F	49	Any other ethnic group
8089857	2403	PDE6B	NM_000283.4	c.1678C>T	p.(Arg560Cys)	Missense	Pathogenic	F	49	Any other ethnic group
6212310	2404	PDE6B	NM_000283.4	c.1257+1G>A	Splice	Splice	Likely Pathogenic	M	58	Any other ethnic group
6212310	2404	PDE6B	NM_000283.4	c.1943T>A	p.(Leu648Gln)	Missense	Likely Pathogenic	M	58	Any other ethnic group
11058781	2405	PDE6B	NM_000283.4	c.1547T>C	p.(Leu516Pro)	Missense	Likely Pathogenic	M	43	Not stated
11058781	2405	PDE6B	NM_000283.4	c.739T>A	p.(Phe247Ile)	Missense	Likely Pathogenic	M	43	Not stated
10638641	2406	PDE6B	NM_000283.4	c.1923_1969delinsTCTGGG	p.(Asn643GlyfsTer29)	Frameshift	Pathogenic	F	43	Unknown
10638641	2406	PDE6B	NM_000283.4	c.892C>T	p.(Gln298Ter)	Stopgain	Pathogenic	F	43	Unknown

10935917	2407	PDE6B	NM_000283.4	c.1697C>T	p.(Ala566Val)	Missense	Variant of Uncertain Significance	F	48	Asian - Bangladeshi
10935917	2407	PDE6B	NM_000283.4	c.1697C>T	p.(Ala566Val)	Missense	Variant of Uncertain Significance	F	48	Asian - Bangladeshi
12230413	2408	PDE6B	NM_000283.4	c.1107+3A>G	Splice	Splice	Pathogenic	F	33	Not stated
12230413	2408	PDE6B	NM_000283.4	c.2193+1G>A	Splice	Splice	Pathogenic	F	33	Not stated
12579811	2409	PDE6B	NM_000283.4	c.1A>G	p.(Met1?)	Start codon	Likely Pathogenic	M	39	Unknown
12579811	2409	PDE6B	NM_000283.4	c.1A>G	p.(Met1?)	Start codon	Likely Pathogenic	M	39	Unknown
12563620	2410	PDE6B	NM_000283.4	c.1160C>T	p.(Pro387Leu)	Missense	Pathogenic	M	18	Asian - Pakistani
12563620	2410	PDE6B	NM_000283.4	c.1160C>T	p.(Pro387Leu)	Missense	Pathogenic	M	18	Asian - Pakistani
7646813	2411	PDE6B	NM_000283.4	c.1655G>A	p.(Arg552Gln)	Missense	Pathogenic	F	57	Not stated
7646813	2411	PDE6B	NM_000283.4	c.1655G>A	p.(Arg552Gln)	Missense	Pathogenic	F	57	Not stated
14839341	2412	PDE6B	NM_000283.4	c.1059+2_1059+3del	Splice	Splice	Likely Pathogenic	F	21	Mixed - Other
14839341	2412	PDE6B	NM_000283.4	c.1669C>T	p.(His557Tyr)	Missense	Pathogenic	F	21	Mixed - Other
14919106	2413	PDE6B	NM_000283.4	c.1860del	p.(His620GlnfsTer23)	Frameshift	Pathogenic	F	15	Not stated
14919106	2413	PDE6B	NM_000283.4	c.810C>A	p.(Cys270Ter)	Stopgain	Pathogenic	F	15	Not stated
15718163	2414	PDE6B	NM_000283.4	c.1107+3A>G	Splice	Splice	Pathogenic	F	11	Not stated
15718163	2414	PDE6B	NM_000283.4	c.1107+3A>G	Splice	Splice	Pathogenic	F	11	Not stated
16797836	2415	PDE6B	NM_000283.4	c.1107+3A>G	Splice	Splice	Pathogenic	M	46	White - British
16797836	2415	PDE6B	NM_000283.4	c.892C>T	p.(Gln298Ter)	Stopgain	Pathogenic	M	46	White - British
14054417	2416	PDE6B	NM_000283.4	c.1580T>C	p.(Leu527Pro)	Missense	Pathogenic	F	78	White - Other
14054417	2416	PDE6B	NM_000283.4	c.1655G>A	p.(Arg552Gln)	Missense	Pathogenic	F	78	White - Other
18155227	2417	PDE6B	NM_000283.4	c.756del	p.(Asp252GlufsTer29)	Frameshift	Pathogenic	M	28	Unknown
18155227	2417	PDE6B	NM_000283.4	c.811G>A	p.(Glu271Lys)	Missense	Likely Pathogenic	M	28	Unknown
18170963	2418	PDE6B	NM_000283.4	c.1107+3A>G	Splice	Splice	Pathogenic	M	43	Not stated
18170963	2418	PDE6B	NM_000283.4	c.1891G>T	p.(Glu631Ter)	Stopgain	Likely Pathogenic	M	43	Not stated
14026760	2419	PDE6B	NM_000283.4	c.1679G>A	p.(Arg560His)	Missense	Likely Pathogenic	F	37	Black - African
14026760	2419	PDE6B	NM_000283.4	c.1679G>A	p.(Arg560His)	Missense	Likely Pathogenic	F	37	Black - African
4481749	2420	PDE6B	NM_000283.4	c.1373G>A	p.(Cys458Tyr)	Missense	Likely Pathogenic	M	86	Black - Caribbean
4481749	2420	PDE6B	NM_000283.4	c.1373G>A	p.(Cys458Tyr)	Missense	Likely Pathogenic	M	86	Black - Caribbean
16964436	2421	PDE6B	NM_000283.4	c.1591C>T	p.(Arg531Ter)	Stopgain	Pathogenic	F	26	White - British
16964436	2421	PDE6B	NM_000283.4	c.1954C>T	p.(Gln652Ter)	Stopgain	Pathogenic	F	26	White - British
18383931	2422	PDE6B	NM_000283.4	c.2338A>C	p.(Thr780Pro)	Missense	Variant of Uncertain Significance	F	49	Asian - Bangladeshi
18383931	2422	PDE6B	NM_000283.4	c.2338A>C	p.(Thr780Pro)	Missense	Variant of Uncertain Significance	F	49	Asian - Bangladeshi
18291909	2423	PDE6B	NM_000283.4	c.1936C>T	p.(Glu646Ter)	Stopgain	Likely Pathogenic	F	30	White - Other
18291909	2423	PDE6B	NM_000283.4	c.2138T>A	p.(Met713Lys)	Missense	Likely Pathogenic	F	30	White - Other
7848693	2424	PDE6C	NM_006204.4	c.1646T>C	p.(Met549Thr)	Missense	Variant of Uncertain Significance	F	46	Asian - Pakistani
7848693	2424	PDE6C	NM_006204.4	c.1646T>C	p.(Met549Thr)	Missense	Variant of Uncertain Significance	F	46	Asian - Pakistani
7848693	2424	PDE6C	NM_006204.4	c.1853C>T	p.(Thr618Met)	Missense	Variant of Uncertain Significance	F	46	Asian - Pakistani
7848693	2424	PDE6C	NM_006204.4	c.1853C>T	p.(Thr618Met)	Missense	Variant of Uncertain Significance	F	46	Asian - Pakistani
10025448	2425	PDE6C	NM_006204.4	c.864+1G>A	Splice	Splice	Pathogenic	F	35	Not stated
10025448	2425	PDE6C	NM_006204.4	c.864+1G>A	Splice	Splice	Pathogenic	F	35	Not stated
10550840	2426	PDE6C	NM_006204.4	c.631G>T	p.(Glu211Ter)	Stopgain	Pathogenic	F	41	Not stated
10550840	2426	PDE6C	NM_006204.4	c.631G>T	p.(Glu211Ter)	Stopgain	Pathogenic	F	41	Not stated
5637365	2427	PDE6C	NM_006204.4	c.304C>T	p.(Arg102Trp)	Missense	Likely Pathogenic	M	55	Asian - Pakistani
5637365	2427	PDE6C	NM_006204.4	c.304C>T	p.(Arg102Trp)	Missense	Likely Pathogenic	M	55	Asian - Pakistani
4829691	2428	PDE6C	NM_006204.4	c.2503G>T	p.(Gly835Ter)	Stopgain	Variant of Uncertain Significance	F	48	Not stated
4829691	2428	PDE6C	NM_006204.4	c.2503G>T	p.(Gly835Ter)	Stopgain	Variant of Uncertain Significance	F	48	Not stated
1036279	2429	PDE6C	NM_006204.4	c.1847+3_1847+6del	Splice	Splice	Pathogenic	F	58	White - British
1036279	2429	PDE6C	NM_006204.4	c.595A>G	p.(Lys199Glu)	Missense	Likely Pathogenic	F	58	White - British
15801316	2430	PDE6C	NM_006204.4	c.326A>T	p.(Glu109Val)	Missense	Variant of Uncertain Significance	F	52	White - British
15801316	2430	PDE6C	NM_006204.4	c.893T>A	p.(Leu298His)	Missense	Variant of Uncertain Significance	F	52	White - British
15986375	2431	PDE6C	NM_006204.4	c.311G>A	p.(Arg104Gln)	Missense	Variant of Uncertain Significance	F	33	Not stated

15986375	2431	PDE6C	NM_006204.4	c.864+1G>A	Splice	Splice	Pathogenic	F	33	Not stated
16395938	2432	PDE6C	NM_006204.4	c.1708G>A	p.(Val570Met)	Missense	Variant of Uncertain Significance	F	10	Asian - Pakistani
16395938	2432	PDE6C	NM_006204.4	c.1708G>A	p.(Val570Met)	Missense	Variant of Uncertain Significance	F	10	Asian - Pakistani
16647532	2433	PDE6C	NM_006204.4	Exon 17-18 deletion	Deletion	Deletion	Variant of Uncertain Significance	M	25	Any other ethnic group
16647532	2433	PDE6C	NM_006204.4	Exon 17-18 deletion	Deletion	Deletion	Variant of Uncertain Significance	M	25	Any other ethnic group
4072116	2434	PDE6G	NM_002602.4	c.109C>T	p.(Gln37Ter)	Stopgain	Likely Pathogenic	F	69	Asian - Indian
4072116	2434	PDE6G	NM_002602.4	c.109C>T	p.(Gln37Ter)	Stopgain	Likely Pathogenic	F	69	Asian - Indian
2614065	2434	PDE6G	NM_002602.4	c.109C>T	p.(Gln37Ter)	Stopgain	Likely Pathogenic	M	86	Unknown
2614065	2434	PDE6G	NM_002602.4	c.109C>T	p.(Gln37Ter)	Stopgain	Likely Pathogenic	M	86	Unknown
11903905	2435	PEX1	NM_000466.3	c.2528G>A	p.(Gly843Asp)	Missense	Pathogenic	F	20	Not stated
11903905	2435	PEX1	NM_000466.3	c.2T>C	p.(Met1?)	Start codon	Pathogenic	F	20	Not stated
2760379	2436	PHYH	NM_00624.4	c.135-2A>G	Splice	Splice	Pathogenic	M	42	White - British
2760379	2436	PHYH	NM_00624.4	c.135-2A>G	Splice	Splice	Pathogenic	M	42	White - British
2793447	2436	PHYH	NM_00624.4	c.135-2A>G	Splice	Splice	Pathogenic	M	44	White - British
2793447	2436	PHYH	NM_00624.4	c.135-2A>G	Splice	Splice	Pathogenic	M	44	White - British
13478751	2437	PHYH	NM_00624.4	c.678+5G>T	Splice	Splice	Variant of Uncertain Significance	M	37	White - British
13478751	2437	PHYH	NM_00624.4	c.823C>T	p.(Arg275Trp)	Missense	Pathogenic	M	37	White - British
8991247	2438	PNPLA6	NM_001166114.2	c.1946+2T>G	Splice	Splice	Likely Pathogenic	M	36	Asian - Indian
8991247	2438	PNPLA6	NM_001166114.2	c.3614T>C	p.(Val1205Ala)	Missense	Likely Pathogenic	M	36	Asian - Indian
14815744	2439	PNPLA6	NM_001166114.2	c.3304G>A	p.(Ala1102Thr)	Missense	Likely Pathogenic	F	19	White - Other
14815744	2439	PNPLA6	NM_001166114.2	c.3517C>T	p.(Arg1173Trp)	Missense	Likely Pathogenic	F	19	White - Other
16089065	2440	PNPLA6	NM_001166114.2	c.187_191del	p.(Thr63ArgfsTer98)	Frameshift	Likely Pathogenic	M	19	Not stated
16089065	2440	PNPLA6	NM_001166114.2	c.3614T>C	p.(Val1205Ala)	Missense	Likely Pathogenic	M	19	Not stated
16647826	2441	PNPLA6	NM_001166114.2	c.3122T>C	p.(Leu1041Ser)	Missense	Variant of Uncertain Significance	F	37	xed - White and Black African
16647826	2441	PNPLA6	NM_001166114.2	c.3137C>T	p.(Pro1046Leu)	Missense	Variant of Uncertain Significance	F	37	xed - White and Black African
16977596	2442	PNPLA6	NM_001166114.2	c.1459_1462del	p.(Tyr487ArgfsTer19)	Frameshift	Likely Pathogenic	M	14	Asian - Indian
16977596	2442	PNPLA6	NM_001166114.2	c.2969T>C	p.(Leu990Ser)	Missense	Variant of Uncertain Significance	M	14	Asian - Indian
6556311	2443	POC1B	NM_172240.3	c.188T>A	p.(Val63Glu)	Missense	Variant of Uncertain Significance	F	33	White - British
6556311	2443	POC1B	NM_172240.3	c.565_566delinsTT	p.(Ala189Leu)	Inframe indel	Variant of Uncertain Significance	F	33	White - British
13524965	2444	POC1B	NM_172240.3	c.681del	p.(His227GlnfsTer32)	Frameshift	Likely Pathogenic	M	58	Unknown
13524965	2444	POC1B	NM_172240.3	c.681del	p.(His227GlnfsTer32)	Frameshift	Likely Pathogenic	M	58	Unknown
17617172	2445	POC1B	NM_172240.3	c.116A>T	p.(Asp39Val)	Missense	Variant of Uncertain Significance	F	31	Asian - Other
17617172	2445	POC1B	NM_172240.3	c.116A>T	p.(Asp39Val)	Missense	Variant of Uncertain Significance	F	31	Asian - Other
17059573	2446	PROM1	NM_006017.3	c.1117C>T	p.(Arg373Cys)	Missense	Pathogenic	F	33	Not stated
3821397	2447	PROM1	NM_006017.3	c.1117C>T	p.(Arg373Cys)	Missense	Pathogenic	M	86	White - Other
1147733	2448	PROM1	NM_006017.3	c.1117C>T	p.(Arg373Cys)	Missense	Pathogenic	M	51	Not stated
2699528	2449	PROM1	NM_006017.3	c.302A>G	p.(Lys101Arg)	Missense	Variant of Uncertain Significance	F	81	White - British
3740197	2449	PROM1	NM_006017.3	c.302A>G	p.(Lys101Arg)	Missense	Variant of Uncertain Significance	F	45	White - British
4786459	2450	PROM1	NM_006017.3	c.1354dup	p.(Tyr452LeufsTer13)	Frameshift	Pathogenic	M	60	White - British
4786459	2450	PROM1	NM_006017.3	c.1354dup	p.(Tyr452LeufsTer13)	Frameshift	Pathogenic	M	60	White - British
12758521	2451	PROM1	NM_006017.3	c.1117C>T	p.(Arg373Cys)	Missense	Pathogenic	F	46	Unknown
14781759	2451	PROM1	NM_006017.3	c.1117C>T	p.(Arg373Cys)	Missense	Pathogenic	F	55	Any other ethnic group
5729737	2452	PROM1	NM_006017.3	c.1117C>T	p.(Arg373Cys)	Missense	Pathogenic	F	59	Not stated
5914565	2452	PROM1	NM_006017.3	c.1117C>T	p.(Arg373Cys)	Missense	Pathogenic	F	69	Not stated
14709694	2453	PROM1	NM_006017.3	c.1117C>T	p.(Arg373Cys)	Missense	Pathogenic	M	52	Black - African
6550207	2454	PROM1	NM_006017.3	c.1710C>A	p.(Tyr570Ter)	Stopgain	Likely Pathogenic	F	65	Not stated
6550207	2454	PROM1	NM_006017.3	c.1726C>T	p.(Gln576Ter)	Stopgain	Pathogenic	F	65	Not stated
1406229	2455	PROM1	NM_006017.3	c.1117C>T	p.(Arg373Cys)	Missense	Pathogenic	M	47	White - British
736203	2455	PROM1	NM_006017.3	c.1117C>T	p.(Arg373Cys)	Missense	Pathogenic	F	73	White - British
6840392	2455	PROM1	NM_006017.3	c.1117C>T	p.(Arg373Cys)	Missense	Pathogenic	M	51	White - British
7355067	2456	PROM1	NM_006017.3	c.2346del	p.(Leu783CysfsTer9)	Frameshift	Likely Pathogenic	M	53	Any other ethnic group

7355067	2456	PROM1	NM_006017.3	c.2346del	p.(Leu783CysfsTer9)	Frameshift	Likely Pathogenic	M	53	Any other ethnic group
9190082	2457	PROM1	NM_006017.3	c.1117C>T	p.(Arg373Cys)	Missense	Pathogenic	F	81	White - British
9111724	2457	PROM1	NM_006017.3	c.1117C>T	p.(Arg373Cys)	Missense	Pathogenic	F	47	White - British
11243399	2458	PROM1	NM_006017.3	c.1117C>T	p.(Arg373Cys)	Missense	Pathogenic	F	47	Black - African
8305772	2458	PROM1	NM_006017.3	c.1117C>T	p.(Arg373Cys)	Missense	Pathogenic	M	58	Black - African
9461843	2459	PROM1	NM_006017.3	c.1354dup	p.(Tyr452LeufsTer13)	Frameshift	Pathogenic	M	23	White - British
9461843	2459	PROM1	NM_006017.3	c.1354dup	p.(Tyr452LeufsTer13)	Frameshift	Pathogenic	M	23	White - British
1203033	2460	PROM1	NM_006017.3	c.1117C>T	p.(Arg373Cys)	Missense	Pathogenic	M	56	White - British
11259457	2461	PROM1	NM_006017.3	c.1389T>A	p.(Tyr463Ter)	Stopgain	Likely Pathogenic	F	48	Asian - Pakistani
11259457	2461	PROM1	NM_006017.3	c.1389T>A	p.(Tyr463Ter)	Stopgain	Likely Pathogenic	F	48	Asian - Pakistani
6116564	2462	PROM1	NM_006017.3	c.1455-1G>A	Splice	Splice	Pathogenic	F	45	Asian - Bangladeshi
6116564	2462	PROM1	NM_006017.3	c.1946C>T	p.(Ser649Leu)	Missense	Variant of Uncertain Significance	F	45	Asian - Bangladeshi
4961004	2463	PROM1	NM_006017.3	c.1141+1G>A	Splice	Splice	Likely Pathogenic	F	44	Asian - Pakistani
4961004	2463	PROM1	NM_006017.3	c.1141+1G>A	Splice	Splice	Likely Pathogenic	F	44	Asian - Pakistani
6592410	2464	PROM1	NM_006017.3	c.436C>T	p.(Arg146Ter)	Stopgain	Pathogenic	F	48	Not stated
6592410	2464	PROM1	NM_006017.3	c.436C>T	p.(Arg146Ter)	Stopgain	Pathogenic	F	48	Not stated
11423558	2465	PROM1	NM_006017.3	c.1117C>T	p.(Arg373Cys)	Missense	Pathogenic	M	42	Asian - Indian
12209931	2466	PROM1	NM_006017.3	c.1002G>A	p.(Gln334Gln)	Synonymous	Variant of Uncertain Significance	M	19	Asian - Other
12209931	2466	PROM1	NM_006017.3	c.1002G>A	p.(Gln334Gln)	Synonymous	Variant of Uncertain Significance	M	19	Asian - Other
10819521	2467	PROM1	NM_006017.3	c.1117C>T	p.(Arg373Cys)	Missense	Pathogenic	M	42	Any other ethnic group
10340035	2468	PROM1	NM_006017.3	c.1632G>T	p.(Gly544Gly)	Synonymous	Variant of Uncertain Significance	F	47	Asian - Other
10340035	2468	PROM1	NM_006017.3	c.1946C>T	p.(Ser649Leu)	Missense	Variant of Uncertain Significance	F	47	Asian - Other
12426518	2469	PROM1	NM_006017.3	c.1117C>T	p.(Arg373Cys)	Missense	Pathogenic	M	51	Not stated
13016639	2470	PROM1	NM_006017.3	c.1002+5G>A	Splice	Splice	Likely Pathogenic	F	44	Any other ethnic group
13016639	2470	PROM1	NM_006017.3	c.1557C>A	p.(Tyr519Ter)	Stopgain	Pathogenic	F	44	Any other ethnic group
12366255	2471	PROM1	NM_006017.3	c.2110C>T	p.(Arg704Cys)	Missense	Likely Pathogenic	F	54	Any other ethnic group
10968439	2472	PROM1	NM_006017.3	c.1354dup	p.(Tyr452LeufsTer13)	Frameshift	Pathogenic	F	58	Unknown
10968439	2472	PROM1	NM_006017.3	c.1579-1G>C	Splice	Splice	Pathogenic	F	58	Unknown
12709367	2473	PROM1	NM_006017.3	c.1579-1G>C	Splice	Splice	Pathogenic	M	36	Not stated
12709367	2473	PROM1	NM_006017.3	c.1579-1G>C	Splice	Splice	Pathogenic	M	36	Not stated
15787519	2473	PROM1	NM_006017.3	c.1579-1G>C	Splice	Splice	Pathogenic	M	24	White - British
15787519	2473	PROM1	NM_006017.3	c.1579-1G>C	Splice	Splice	Pathogenic	M	24	White - British
12654193	2474	PROM1	NM_006017.3	c.1354dup	p.(Tyr452LeufsTer13)	Frameshift	Pathogenic	M	36	Not stated
12654193	2474	PROM1	NM_006017.3	[illegible]30_c.630+8delinsAACTTGAATGA[illegible]	Splice	Splice	Likely Pathogenic	M	36	Not stated
13227122	2475	PROM1	NM_006017.3	c.1117C>T	p.(Arg373Cys)	Missense	Pathogenic	M	53	Not stated
13354186	2476	PROM1	NM_006017.3	c.1117C>T	p.(Arg373Cys)	Missense	Pathogenic	M	47	Unknown
13305872	2477	PROM1	NM_006017.3	c.1117C>T	p.(Arg373Cys)	Missense	Pathogenic	M	53	Not stated
13999334	2478	PROM1	NM_006017.3	c.1177_1178del	p.(Ile393ArgfsTer21)	Frameshift	Pathogenic	F	19	White - British
13999334	2478	PROM1	NM_006017.3	c.730C>T	p.(Arg244Ter)	Stopgain	Pathogenic	F	19	White - British
14898610	2479	PROM1	NM_006017.3	c.1117C>T	p.(Arg373Cys)	Missense	Pathogenic	M	56	Unknown
13387289	2480	PROM1	NM_006017.3	c.2110C>T	p.(Arg704Cys)	Missense	Likely Pathogenic	M	20	Not stated
13215796	2481	PROM1	NM_006017.3	Multiexon deletion	Deletion	Deletion	Likely Pathogenic	F	30	Asian - Other
13215796	2481	PROM1	NM_006017.3	Multiexon deletion	Deletion	Deletion	Likely Pathogenic	F	30	Asian - Other
15134909	2482	PROM1	NM_006017.3	c.1117C>T	p.(Arg373Cys)	Missense	Pathogenic	F	43	Not stated
11504877	2483	PROM1	NM_006017.3	c.1142-1G>A	Splice	Splice	Pathogenic	F	24	Any other ethnic group
11504877	2483	PROM1	NM_006017.3	c.1142-1G>A	Splice	Splice	Pathogenic	F	24	Any other ethnic group
14833825	2484	PROM1	NM_006017.3	c.1354dup	p.(Tyr452LeufsTer13)	Frameshift	Pathogenic	M	25	Unknown
14833825	2484	PROM1	NM_006017.3	c.1579-1G>C	Splice	Splice	Pathogenic	M	25	Unknown
15161362	2485	PROM1	NM_006017.3	c.1726C>T	p.(Gln576Ter)	Stopgain	Pathogenic	F	25	Asian - Pakistani
15161362	2485	PROM1	NM_006017.3	c.1726C>T	p.(Gln576Ter)	Stopgain	Pathogenic	F	25	Asian - Pakistani
15329579	2486	PROM1	NM_006017.3	c.1579del	p.(Val527PhefsTer8)	Frameshift	Likely Pathogenic	F	35	Any other ethnic group

15329579	2486	PROM1	NM_006017.3	c.1579del	p.(Val527PhefsTer8)	Frameshift	Likely Pathogenic	F	35	Any other ethnic group
16652943	2487	PROM1	NM_006017.3	c.1887_1890del	p.(Met629IlefsTer6)	Frameshift	Pathogenic	F	13	White - British
16652943	2487	PROM1	NM_006017.3	Exon 4 deletion	Deletion	Deletion	Variant of Uncertain Significance	F	13	White - British
17344410	2488	PROM1	NM_006017.3	c.1117C>T	p.(Arg373Cys)	Missense	Pathogenic	F	71	White - British
17101167	2489	PROM1	NM_006017.3	c.1117C>T	p.(Arg373Cys)	Missense	Pathogenic	M	22	Not stated
16720990	2490	PROM1	NM_006017.3	c.1632G>T	p.(Gly544Gly)	Synonymous	Variant of Uncertain Significance	F	25	Any other ethnic group
16720990	2490	PROM1	NM_006017.3	c.730C>T	p.(Arg244Ter)	Stopgain	Pathogenic	F	25	Any other ethnic group
299130	2491	PROM1	NM_006017.3	c.1354dup	p.(Tyr452LeufsTer13)	Frameshift	Pathogenic	F	67	Not stated
299130	2491	PROM1	NM_006017.3	c.622del	p.(Thr208LeufsTer23)	Frameshift	Pathogenic	F	67	Not stated
716449	2492	PROM1	NM_006017.3	c.139del	p.(His47IlefsTer12)	Frameshift	Pathogenic	F	77	White - British
716449	2492	PROM1	NM_006017.3	c.2458C>G	p.(Arg820Gly)	Missense	Variant of Uncertain Significance	F	77	White - British
3316361	2493	PRPF31	NM_015629.4	c.770dup	p.(Thr258AspfsTer21)	Frameshift	Pathogenic	F	69	Unknown
319780	2494	PRPF31	NM_015629.4	c.527+3A>G	Splice	Splice	Likely Pathogenic	F	53	White - British
99714	2494	PRPF31	NM_015629.4	c.1113_1123del	p.(Arg372GlnfsTer99)	Frameshift	Pathogenic	F	72	Not stated
5035862	2495	PRPF31	NM_015629.4	c.527+3A>G	Splice	Splice	Likely Pathogenic	F	76	Not stated
8607752	2495	PRPF31	NM_015629.4	c.527+3A>G	Splice	Splice	Likely Pathogenic	M	52	White - British
748691	2496	PRPF31	NM_015629.4	c.877del	p.(Arg293GlyfsTer28)	Frameshift	Likely Pathogenic	M	69	White - British
3271533	2497	PRPF31	NM_015629.4	c.527+3A>G	Splice	Splice	Likely Pathogenic	F	60	Not stated
1840621	2498	PRPF31	NM_015629.4	c.646G>C	p.(Ala216Pro)	Missense	Likely Pathogenic	F	43	White - British
8031526	2499	PRPF31	NM_015629.4	c.528-39_531del	p.(Gln177SerfsTer20)	Frameshift	Likely Pathogenic	F	43	White - British
7634556	2499	PRPF31	NM_015629.4	c.528-39_531del	p.(Gln177SerfsTer20)	Frameshift	Likely Pathogenic	F	45	White - British
11637779	2500	PRPF31	NM_015629.4	c.1120C>T	p.(Gln374Ter)	Stopgain	Pathogenic	F	24	Not stated
11822432	2501	PRPF31	NM_015629.4	c.1374+569C>G	Splice	Splice	Variant of Uncertain Significance	M	39	Any other ethnic group
18272253	2501	PRPF31	NM_015629.4	c.1374+569C>G	Splice	Splice	Variant of Uncertain Significance	F	34	Unknown
17083513	2502	PRPF31	NM_015629.4	c.239-1G>A	Splice	Splice	Pathogenic	F	44	Unknown
13574847	2503	PRPF31	NM_015629.4	c.1073+5G>A	Splice	Splice	Variant of Uncertain Significance	F	39	Unknown
10744264	2504	PRPF31	NM_015629.4	c.341T>A	p.(Ile114Asn)	Missense	Variant of Uncertain Significance	F	47	Not stated
5147281	2505	PRPF31	NM_015629.4	c.584T>C	p.(Leu195Pro)	Missense	Variant of Uncertain Significance	F	57	Not stated
697262	2505	PRPF31	NM_015629.4	c.584T>C	p.(Leu195Pro)	Missense	Variant of Uncertain Significance	F	99	Not stated
16225978	2505	PRPF31	NM_015629.4	c.584T>C	p.(Leu195Pro)	Missense	Variant of Uncertain Significance	M	75	Not stated
12981450	2506	PRPF31	NM_015629.4	c.527+3A>G	Splice	Splice	Likely Pathogenic	M	39	Not stated
605716	2507	PRPF31	NM_015629.4	Exon 3-12 deletion	Deletion	Deletion	Likely Pathogenic	M	62	White - British
3007437	2508	PRPF31	NM_015629.4	c.527+3A>G	Splice	Splice	Likely Pathogenic	F	69	White - British
943347	2509	PRPF31	NM_015629.4	c.79G>T	p.(Glu27Ter)	Stopgain	Likely Pathogenic	F	61	Not stated
484035	2510	PRPF31	NM_015629.4	c.413C>A	p.(Thr138Lys)	Missense	Variant of Uncertain Significance	F	69	Not stated
3307569	2511	PRPF31	NM_015629.4	c.527+1G>T	Splice	Splice	Pathogenic	F	70	Not stated
17917318	2512	PRPF31	NM_015629.4	c.527+3A>G	Splice	Splice	Likely Pathogenic	F	13	Not stated
6432593	2513	PRPF31	NM_015629.4	c.529C>T	p.(Gln177Ter)	Stopgain	Pathogenic	M	61	White - British
3846044	2514	PRPF31	NM_015629.4	c.413C>A	p.(Thr138Lys)	Missense	Variant of Uncertain Significance	M	70	Not stated
4541949	2515	PRPF31	NM_015629.4	c.523C>T	p.(Gln175Ter)	Stopgain	Pathogenic	F	74	White - British
5479389	2516	PRPF31	NM_015629.4	c.322+4_322+7del	Splice	Splice	Pathogenic	M	55	White - British
9301277	2517	PRPF31	NM_015629.4	c.527+1G>T	Splice	Splice	Pathogenic	M	55	Not stated
5586188	2518	PRPF31	NM_015629.4	c.527+3A>G	Splice	Splice	Likely Pathogenic	F	73	Not stated
5268073	2519	PRPF31	NM_015629.4	c.356C>G	p.(Ser119Ter)	Stopgain	Likely Pathogenic	M	70	White - British
62229	2520	PRPF31	NM_015629.4	c.1129del	p.(Arg377ValfsTer2)	Frameshift	Pathogenic	M	42	Unknown
6781774	2521	PRPF31	NM_015629.4	c.527+3A>G	Splice	Splice	Likely Pathogenic	M	55	Any other ethnic group
691172	2522	PRPF31	NM_015629.4	c.527+3A>G	Splice	Splice	Likely Pathogenic	F	77	White - British
7214290	2523	PRPF31	NM_015629.4	c.757G>A	p.(Gly253Arg)	Missense	Pathogenic	F	53	Not stated
10602836	2524	PRPF31	NM_015629.4	c.1146+2T>C	Splice	Splice	Likely Pathogenic	F	61	White - British
13512386	2524	PRPF31	NM_015629.4	c.1146+2T>C	Splice	Splice	Likely Pathogenic	M	32	Unknown
7629369	2524	PRPF31	NM_015629.4	c.1146+2T>C	Splice	Splice	Likely Pathogenic	F	38	Not stated

7824004	2525	PRPF31	NM_015629.4	c.527+3A>G	Splice	Splice	Likely Pathogenic	M	54	White - British
8069179	2526	PRPF31	NM_015629.4	c.202G>T	p.(Glu68Ter)	Stopgain	Likely Pathogenic	M	40	White - British
1814980	2527	PRPF31	NM_015629.4	c.528-1G>A	Splice	Splice	Pathogenic	M	67	White - British
9019394	2528	PRPF31	NM_015629.4	c.335del	p.(Lys112SerfsTer86)	Frameshift	Pathogenic	M	55	White - British
9812186	2529	PRPF31	NM_015629.4	c.527+3A>G	Splice	Splice	Likely Pathogenic	F	61	White - British
9943849	2530	PRPF31	NM_015629.4	c.973G>T	p.(Glu325Ter)	Stopgain	Pathogenic	F	57	White - British
892093	2531	PRPF31	NM_015629.4	c.319C>G	p.(Leu107Val)	Missense	Variant of Uncertain Significance	F	58	White - British
10253655	2532	PRPF31	NM_015629.4	Exon 1-14 deletion	Deletion	Deletion	Likely Pathogenic	M	55	Not stated
1384095	1108	PRPF31	NM_015629.4	c.527+3A>G	Splice	Splice	Likely Pathogenic	M	72	White - British
11681466	2533	PRPF31	NM_015629.4	c.1087A>T	p.(Lys363Ter)	Stopgain	Likely Pathogenic	F	27	White - Other
18425420	2534	PRPF31	NM_015629.4	c.1275+1del	Splice	Splice	Likely Pathogenic	M	37	Not stated
6541583	2535	PRPF31	NM_015629.4	c.838_841dup	p.(Gln281ArgfsTer44)	Frameshift	Pathogenic	M	45	Not stated
11082014	2536	PRPF31	NM_015629.4	c.238+2T>C	Splice	Splice	Likely Pathogenic	F	45	Not stated
15705129	2537	PRPF31	NM_015629.4	c.459_460del	p.(Asn154ProfsTer124)	Frameshift	Likely Pathogenic	M	30	Not stated
3301101	2537	PRPF31	NM_015629.4	c.459_460del	p.(Asn154ProfsTer124)	Frameshift	Likely Pathogenic	F	35	Not stated
10427465	2538	PRPF31	NM_015629.4	c.808del	p.(His270ThrfsTer51)	Frameshift	Likely Pathogenic	M	20	Not stated
11477206	2538	PRPF31	NM_015629.4	c.808del	p.(His270ThrfsTer51)	Frameshift	Likely Pathogenic	F	44	Unknown
15513070	2539	PRPF31	NM_015629.4	Exon 1-14 rearrangement	Complex rearrangement	Complex rearrangemen	Likely Pathogenic	F	38	Any other ethnic group
154139	2540	PRPF31	NM_015629.4	c.319C>G	p.(Leu107Val)	Missense	Variant of Uncertain Significance	F	67	White - Other
15562980	2541	PRPF31	NM_015629.4	c.1074-2A>G	Splice	Splice	Pathogenic	F	55	Any other ethnic group
13779394	2542	PRPF31	NM_015629.4	c.527+3A>G	Splice	Splice	Likely Pathogenic	F	59	White - British
12546197	2543	PRPF31	NM_015629.4	c.527+1G>T	Splice	Splice	Pathogenic	F	57	Not stated
15561048	2544	PRPF31	NM_015629.4	c.527+3A>G	Splice	Splice	Likely Pathogenic	F	56	Not stated
15431261	2545	PRPF31	NM_015629.4	c.527+3A>G	Splice	Splice	Likely Pathogenic	M	51	Not stated
14883861	2546	PRPF31	NM_015629.4	c.946-3C>G	Splice	Splice	Variant of Uncertain Significance	M	71	Unknown
16177622	2547	PRPF31	NM_015629.4	Exon 4-5 deletion	Deletion	Deletion	Variant of Uncertain Significance	F	16	Asian - Indian
16470075	2548	PRPF31	NM_015629.4	c.908C>A	p.(Ala303Asp)	Missense	Variant of Uncertain Significance	F	20	Not stated
6394730	2549	PRPF31	NM_015629.4	Exon 1 deletion	Deletion	Deletion	Likely Pathogenic	F	41	Not stated
16553907	2550	PRPF31	NM_015629.4	c.547G>T	p.(Glu183Ter)	Stopgain	Likely Pathogenic	F	26	White - Other
17125737	2551	PRPF31	NM_015629.4	c.527+3A>G	Splice	Splice	Likely Pathogenic	F	61	Not stated
16880240	2552	PRPF31	NM_015629.4	c.527+3A>G	Splice	Splice	Likely Pathogenic	F	70	Not stated
17229057	2553	PRPF31	NM_015629.4	c.527+3A>G	Splice	Splice	Likely Pathogenic	F	25	Not stated
17287920	2554	PRPF31	NM_015629.4	c.527+3A>G	Splice	Splice	Likely Pathogenic	F	38	Not stated
11433162	2555	PRPF31	NM_015629.4	c.1073+3_1073+9del	Splice	Splice	Variant of Uncertain Significance	M	29	Not stated
18068798	2556	PRPF31	NM_015629.4	Exon 10-11 deletion	Deletion	Deletion	Likely Pathogenic	F	30	White - British
16260299	2557	PRPF31	NM_015629.4	c.528-1G>A	Splice	Splice	Pathogenic	F	31	Not stated
18279589	2558	PRPF31	NM_015629.4	Exon 9-14 deletion	Deletion	Deletion	Likely Pathogenic	F	35	Not stated
18367040	2559	PRPF31	NM_015629.4	c.760dup	p.(Ala254GlyfsTer25)	Frameshift	Likely Pathogenic	F	48	Not stated
18250553	2560	PRPF31	NM_015629.4	c.527+1G>T	Splice	Splice	Pathogenic	F	40	Not stated
11062323	2561	PRPF31	NM_015629.4	Exon 1-14 deletion	Deletion	Deletion	Likely Pathogenic	F	19	White - British
18355952	2562	PRPF31	NM_015629.4	Exon 1-14 deletion	Deletion	Deletion	Likely Pathogenic	F	19	Not stated
15921891	2563	PRPF6	NM_012469.4	c.514C>T	p.(Arg172Trp)	Missense	Likely Pathogenic	F	59	Any other ethnic group
8906743	2564	PRPF6	NM_012469.4	c.514C>T	p.(Arg172Trp)	Missense	Likely Pathogenic	F	57	White - British
16075086	2565	PRPF6	NM_012469.4	c.514C>T	p.(Arg172Trp)	Missense	Likely Pathogenic	M	55	White - British
18387340	2566	PRPF6	NM_012469.4	c.514C>T	p.(Arg172Trp)	Missense	Likely Pathogenic	F	57	Not stated
12471416	2567	PRPF8	NM_006445.4	c.6353C>T	p.(Ser2118Phe)	Missense	Variant of Uncertain Significance	F	39	Not stated
17909268	2567	PRPF8	NM_006445.4	c.6353C>T	p.(Ser2118Phe)	Missense	Variant of Uncertain Significance	F	52	Unknown
4582563	2568	PRPF8	NM_006445.4	c.6901C>T	p.(Pro2301Ser)	Missense	Pathogenic	F	71	Any other ethnic group
12765682	2568	PRPF8	NM_006445.4	c.6901C>T	p.(Pro2301Ser)	Missense	Pathogenic	F	43	Unknown
3658290	2569	PRPF8	NM_006445.4	c.6942C>A	p.(Phe2341Leu)	Missense	Pathogenic	F	64	White - British
5035015	2570	PRPF8	NM_006445.4	c.6992A>G	p.(Glu2331Gly)	Missense	Likely Pathogenic	F	75	Not stated

18653564	2570	PRPF8	NM_006445.4	c.6992A>G	p.(Glu2331Gly)	Missense	Likely Pathogenic	F	29	Not stated
9560116	2571	PRPF8	NM_006445.4	c.6926A>C	p.(His2309Pro)	Missense	Pathogenic	F	53	White - British
2698394	2572	PRPF8	NM_006445.4	c.5804G>A	p.(Arg1935His)	Missense	Pathogenic	F	53	White - British
14812615	2572	PRPF8	NM_006445.4	c.5804G>A	p.(Arg1935His)	Missense	Pathogenic	M	31	Unknown
14812650	2572	PRPF8	NM_006445.4	c.5804G>A	p.(Arg1935His)	Missense	Pathogenic	M	29	Any other ethnic group
1178421	2573	PRPF8	NM_006445.4	c.6970dup	p.(Glu2324GlyfsTer61)	Frameshift	Pathogenic	F	47	White - Irish
8504670	2574	PRPF8	NM_006445.4	c.6840C>A	p.(Asn2280Lys)	Missense	Variant of Uncertain Significance	M	26	Not stated
6805700	2574	PRPF8	NM_006445.4	c.6840C>A	p.(Asn2280Lys)	Missense	Variant of Uncertain Significance	F	58	White - British
5580329	2575	PRPF8	NM_006445.4	c.6928A>G	p.(Arg2310Gly)	Missense	Pathogenic	F	66	White - British
8010519	2575	PRPF8	NM_006445.4	c.6928A>G	p.(Arg2310Gly)	Missense	Pathogenic	F	61	Not stated
8916858	2576	PRPF8	NM_006445.4	c.7000T>A	p.(Tyr2334Asn)	Missense	Pathogenic	M	32	White - Other
4170452	2576	PRPF8	NM_006445.4	c.7000T>A	p.(Tyr2334Asn)	Missense	Pathogenic	M	66	Not stated
9595417	2576	PRPF8	NM_006445.4	c.7000T>A	p.(Tyr2334Asn)	Missense	Pathogenic	M	83	Not stated
15537808	2577	PRPF8	NM_006445.4	c.7007G>C	p.(Ter2336SerextTer41)	Stoploss	Likely Pathogenic	F	30	White - British
16606610	2578	PRPF8	NM_006445.4	c.6929G>A	p.(Arg2310Lys)	Missense	Pathogenic	F	35	White - Other
18772053	2578	PRPF8	NM_006445.4	c.6929G>A	p.(Arg2310Lys)	Missense	Pathogenic	F	31	Not stated
4490702	2579	PRPF8	NM_006445.4	c.6928A>G	p.(Arg2310Gly)	Missense	Pathogenic	M	56	Any other ethnic group
12671966	2580	PRPH2	NM_000322.5	c.646C>T	p.(Pro216Ser)	Missense	Pathogenic	M	31	Unknown
8987	2581	PRPH2	NM_000322.5	c.356_358del	p.(Cys119del)	Inframe indel	Variant of Uncertain Significance	F	53	White - British
15805124	2581	PRPH2	NM_000322.5	c.356_358del	p.(Cys119del)	Inframe indel	Variant of Uncertain Significance	M	46	White - British
15260433	2582	PRPH2	NM_000322.5	c.634A>G	p.(Ser212Gly)	Missense	Pathogenic	M	57	White - British
7464729	2583	PRPH2	NM_000322.5	c.514C>T	p.(Arg172Trp)	Missense	Likely Pathogenic	F	73	White - British
9195073	2584	PRPH2	NM_000322.5	c.394del	p.(Gln132LysfsTer7)	Frameshift	Pathogenic	F	57	White - British
6767053	2585	PRPH2	NM_000322.5	c.514C>T	p.(Arg172Trp)	Missense	Likely Pathogenic	M	66	White - British
1343600	2586	PRPH2	NM_000322.5	c.647C>T	p.(Pro216Leu)	Missense	Pathogenic	M	72	White - Irish
3334729	2587	PRPH2	NM_000322.5	c.551A>C	p.(Tyr184Ser)	Missense	Likely Pathogenic	F	66	Unknown
1988629	2588	PRPH2	NM_000322.5	c.514C>T	p.(Arg172Trp)	Missense	Likely Pathogenic	F	62	White - British
13599011	2588	PRPH2	NM_000322.5	c.514C>T	p.(Arg172Trp)	Missense	Likely Pathogenic	F	26	Unknown
12495804	2589	PRPH2	NM_000322.5	c.514C>T	p.(Arg172Trp)	Missense	Likely Pathogenic	F	48	Any other ethnic group
3592434	2590	PRPH2	NM_000322.5	c.514C>T	p.(Arg172Trp)	Missense	Likely Pathogenic	F	37	Not stated
172850	2591	PRPH2	NM_000322.5	c.418_421dup	p.(Tyr141LeufsTer37)	Frameshift	Pathogenic	F	76	Any other ethnic group
9378480	2592	PRPH2	NM_000322.5	c.514C>T	p.(Arg172Trp)	Missense	Likely Pathogenic	F	44	Not stated
9818514	2593	PRPH2	NM_000322.5	c.514C>T	p.(Arg172Trp)	Missense	Likely Pathogenic	M	51	White - British
4380319	2594	PRPH2	NM_000322.5	c.514C>T	p.(Arg172Trp)	Missense	Likely Pathogenic	M	62	Not stated
7505532	2595	PRPH2	NM_000322.5	c.514C>T	p.(Arg172Trp)	Missense	Likely Pathogenic	M	60	Not stated
1260167	2596	PRPH2	NM_000322.5	c.514C>T	p.(Arg172Trp)	Missense	Likely Pathogenic	F	70	White - British
8711884	2597	PRPH2	NM_000322.5	c.514C>T	p.(Arg172Trp)	Missense	Likely Pathogenic	F	64	Not stated
4001605	2598	PRPH2	NM_000322.5	c.136C>T	p.(Arg46Ter)	Stopgain	Pathogenic	F	82	White - Other
169994	2598	PRPH2	NM_000322.5	c.136C>T	p.(Arg46Ter)	Stopgain	Pathogenic	F	85	White - Other
8294152	2599	PRPH2	NM_000322.5	c.514C>T	p.(Arg172Trp)	Missense	Likely Pathogenic	M	75	White - British
5079108	2600	PRPH2	NM_000322.5	c.514C>T	p.(Arg172Trp)	Missense	Likely Pathogenic	M	56	Not stated
13824586	2600	PRPH2	NM_000322.5	c.514C>T	p.(Arg172Trp)	Missense	Likely Pathogenic	F	25	Unknown
13502145	2601	PRPH2	NM_000322.5	c.259_266del	p.(Asp87GlnfsTer87)	Frameshift	Pathogenic	F	59	Not stated
5308498	2602	PRPH2	NM_000322.5	c.643A>C	p.(Asn215His)	Missense	Pathogenic	M	63	White - British
3879189	2603	PRPH2	NM_000322.5	c.637T>C	p.(Cys213Arg)	Missense	Pathogenic	F	76	White - Other
15662814	2604	PRPH2	NM_000322.5	c.394del	p.(Gln132LysfsTer7)	Frameshift	Pathogenic	F	53	Not stated
9229233	2605	PRPH2	NM_000322.5	c.514C>T	p.(Arg172Trp)	Missense	Likely Pathogenic	M	44	White - British
10093803	2606	PRPH2	NM_000322.5	c.603_620del	p.(Arg203_Gly208del)	Inframe indel	Pathogenic	M	60	Not stated
2590335	2607	PRPH2	NM_000322.5	c.514C>T	p.(Arg172Trp)	Missense	Likely Pathogenic	M	67	Mixed - White and Asian
5020581	2608	PRPH2	NM_000322.5	c.499G>A	p.(Gly167Ser)	Missense	Pathogenic	F	76	Asian - Indian
6121919	2609	PRPH2	NM_000322.5	c.514C>T	p.(Arg172Trp)	Missense	Likely Pathogenic	M	79	White - British

6468965	2610	PRPH2	NM_000322.5	c.514C>T	p.(Arg172Trp)	Missense	Likely Pathogenic	F	67	Not stated
6501137	2611	PRPH2	NM_000322.5	c.514C>T	p.(Arg172Trp)	Missense	Likely Pathogenic	F	50	Unknown
7117004	2612	PRPH2	NM_000322.5	c.584G>T	p.(Arg195Leu)	Missense	Pathogenic	F	53	Not stated
11241775	2613	PRPH2	NM_000322.5	c.514C>T	p.(Arg172Trp)	Missense	Likely Pathogenic	F	50	White - British
429288	2614	PRPH2	NM_000322.5	c.514C>T	p.(Arg172Trp)	Missense	Likely Pathogenic	M	90	White - British
1960384	2614	PRPH2	NM_000322.5	c.514C>T	p.(Arg172Trp)	Missense	Likely Pathogenic	M	63	White - British
7033263	2615	PRPH2	NM_000322.5	c.424C>T	p.(Arg142Trp)	Missense	Pathogenic	F	40	White - British
7128092	2616	PRPH2	NM_000322.5	c.514C>T	p.(Arg172Trp)	Missense	Likely Pathogenic	M	84	White - British
7225105	2617	PRPH2	NM_000322.5	c.394del	p.(Gln132LysfsTer7)	Frameshift	Pathogenic	M	51	White - British
7631084	2618	PRPH2	NM_000322.5	c.514C>T	p.(Arg172Trp)	Missense	Likely Pathogenic	F	67	White - British
6723261	2618	PRPH2	NM_000322.5	c.514C>T	p.(Arg172Trp)	Missense	Likely Pathogenic	M	64	White - British
7601824	2619	PRPH2	NM_000322.5	c.469G>A	p.(Asp157Asn)	Missense	Pathogenic	M	58	White - Other
7934401	2620	PRPH2	NM_000322.5	c.62G>A	p.(Trp21Ter)	Stopgain	Pathogenic	F	74	Not stated
15265480	2620	PRPH2	NM_000322.5	c.62G>A	p.(Trp21Ter)	Stopgain	Pathogenic	F	52	Unknown
7641381	2620	PRPH2	NM_000322.5	c.62G>A	p.(Trp21Ter)	Stopgain	Pathogenic	F	47	White - British
5915664	2621	PRPH2	NM_000322.5	c.634A>G	p.(Ser212Gly)	Missense	Pathogenic	M	73	Not stated
8661456	2622	PRPH2	NM_000322.5	c.259_266del	p.(Asp87GlnfsTer87)	Frameshift	Pathogenic	M	62	Not stated
9057992	2623	PRPH2	NM_000322.5	c.163del	p.(Ser55LeufsTer10)	Frameshift	Pathogenic	F	63	White - British
11889023	2624	PRPH2	NM_000322.5	c.515G>A	p.(Arg172Gln)	Missense	Pathogenic	M	32	White - British
8966957	2624	PRPH2	NM_000322.5	c.515G>A	p.(Arg172Gln)	Missense	Pathogenic	M	69	Not stated
9366986	2625	PRPH2	NM_000322.5	c.656C>G	p.(Pro219Arg)	Missense	Variant of Uncertain Significance	F	65	White - British
9443321	2626	PRPH2	NM_000322.5	c.259_266del	p.(Asp87GlnfsTer87)	Frameshift	Pathogenic	F	77	Not stated
9475465	2627	PRPH2	NM_000322.5	c.394del	p.(Gln132LysfsTer7)	Frameshift	Pathogenic	M	80	Not stated
9823386	2628	PRPH2	NM_000322.5	c.515G>A	p.(Arg172Gln)	Missense	Pathogenic	M	52	Black - African
7159676	2629	PRPH2	NM_000322.5	c.656C>G	p.(Pro219Arg)	Missense	Variant of Uncertain Significance	F	72	White - British
9802505	2630	PRPH2	NM_000322.5	c.605G>A	p.(Gly202Glu)	Missense	Likely Pathogenic	F	61	Unknown
10667866	2630	PRPH2	NM_000322.5	c.605G>A	p.(Gly202Glu)	Missense	Likely Pathogenic	F	89	Unknown
10517513	2630	PRPH2	NM_000322.5	c.605G>A	p.(Gly202Glu)	Missense	Likely Pathogenic	F	57	Unknown
7183644	2631	PRPH2	NM_000322.5	c.656C>G	p.(Pro219Arg)	Missense	Variant of Uncertain Significance	F	56	White - British
11377232	2631	PRPH2	NM_000322.5	c.656C>G	p.(Pro219Arg)	Missense	Variant of Uncertain Significance	M	83	Any other ethnic group
1864449	2632	PRPH2	NM_000322.5	c.394del	p.(Gln132LysfsTer7)	Frameshift	Pathogenic	M	68	White - British
9610306	2633	PRPH2	NM_000322.5	c.514C>T	p.(Arg172Trp)	Missense	Likely Pathogenic	F	60	Not stated
10107089	2634	PRPH2	NM_000322.5	c.514C>T	p.(Arg172Trp)	Missense	Likely Pathogenic	M	68	Unknown
12166916	2635	PRPH2	NM_000322.5	c.582-2A>T	Splice	Splice	Pathogenic	M	89	Unknown
10470305	2635	PRPH2	NM_000322.5	c.582-2A>T	Splice	Splice	Pathogenic	F	58	Not stated
10466182	2636	PRPH2	NM_000322.5	c.514C>T	p.(Arg172Trp)	Missense	Likely Pathogenic	F	85	Unknown
6364154	2636	PRPH2	NM_000322.5	c.514C>T	p.(Arg172Trp)	Missense	Likely Pathogenic	F	54	Not stated
10303929	2637	PRPH2	NM_000322.5	c.259_266del	p.(Asp87GlnfsTer87)	Frameshift	Pathogenic	F	60	White - British
10428515	2638	PRPH2	NM_000322.5	c.514C>T	p.(Arg172Trp)	Missense	Likely Pathogenic	M	53	White - British
10226355	2639	PRPH2	NM_000322.5	c.259_266del	p.(Asp87GlnfsTer87)	Frameshift	Pathogenic	M	60	White - British
10243057	2640	PRPH2	NM_000322.5	c.638G>A	p.(Cys213Tyr)	Missense	Likely Pathogenic	M	72	Not stated
10724363	2641	PRPH2	NM_000322.5	c.422A>G	p.(Tyr141Cys)	Missense	Pathogenic	F	77	White - British
10617487	2642	PRPH2	NM_000322.5	c.618_626del	p.(Asp207_Val209del)	Inframe indel	Likely Pathogenic	F	48	Unknown
10726512	2643	PRPH2	NM_000322.5	c.554T>C	p.(Lys185Pro)	Missense	Pathogenic	M	83	White - Other
8036510	2644	PRPH2	NM_000322.5	c.514C>T	p.(Arg172Trp)	Missense	Likely Pathogenic	F	52	Not stated
18332271	2644	PRPH2	NM_000322.5	c.514C>T	p.(Arg172Trp)	Missense	Likely Pathogenic	F	27	Not stated
10966206	2645	PRPH2	NM_000322.5	c.625G>T	p.(Val209Phe)	Missense	Likely Pathogenic	M	71	White - British
9900603	2646	PRPH2	NM_000322.5	c.653C>T	p.(Ser218Leu)	Missense	Pathogenic	F	47	Black - African
8247140	2647	PRPH2	NM_000322.5	c.664T>C	p.(Cys222Arg)	Missense	Likely Pathogenic	M	77	Not stated
11597291	2648	PRPH2	NM_000322.5	c.514C>T	p.(Arg172Trp)	Missense	Likely Pathogenic	M	49	Unknown
11642308	2649	PRPH2	NM_000322.5	Exon 1 deletion	Deletion	Deletion	Likely Pathogenic	M	59	Not stated

11897108	2650	PRPH2	NM_000322.5	c.612C>G	p.(Tyr204Ter)	Stopgain	Pathogenic	F	53	White - British
4106591	2650	PRPH2	NM_000322.5	c.612C>G	p.(Tyr204Ter)	Stopgain	Pathogenic	F	76	Not stated
11284510	2650	PRPH2	NM_000322.5	c.612C>G	p.(Tyr204Ter)	Stopgain	Pathogenic	M	52	Unknown
11612621	2651	PRPH2	NM_000322.5	c.514C>T	p.(Arg172Trp)	Missense	Likely Pathogenic	M	52	White - British
11898529	2652	PRPH2	NM_000322.5	c.634A>G	p.(Ser212Gly)	Missense	Pathogenic	M	49	White - British
11816671	2653	PRPH2	NM_000322.5	c.640T>A	p.(Cys214Ser)	Missense	Likely Pathogenic	M	64	Any other ethnic group
12606348	2653	PRPH2	NM_000322.5	c.640T>A	p.(Cys214Ser)	Missense	Likely Pathogenic	M	61	Any other ethnic group
11623233	2654	PRPH2	NM_000322.5	c.514C>T	p.(Arg172Trp)	Missense	Likely Pathogenic	M	60	Unknown
7687973	2655	PRPH2	NM_000322.5	c.583C>G	p.(Arg195Gly)	Missense	Likely Pathogenic	M	66	Not stated
11193083	2656	PRPH2	NM_000322.5	c.394del	p.(Gln132LysfsTer7)	Frameshift	Pathogenic	M	53	Not stated
6667954	865	PRPH2	NM_000322.5	c.136C>T	p.(Arg46Ter)	Stopgain	Pathogenic	M	55	White - British
12471899	2657	PRPH2	NM_000322.5	c.227C>A	p.(Ser76Ter)	Stopgain	Pathogenic	F	75	Unknown
12248697	2658	PRPH2	NM_000322.5	c.640T>A	p.(Cys214Ser)	Missense	Likely Pathogenic	F	72	White - British
10821390	2659	PRPH2	NM_000322.5	c.612C>G	p.(Tyr204Ter)	Stopgain	Pathogenic	F	64	White - Other
5970565	2660	PRPH2	NM_000322.5	c.80C>T	p.(Ser27Phe)	Missense	Likely Pathogenic	F	71	Not stated
5970579	2660	PRPH2	NM_000322.5	c.80C>T	p.(Ser27Phe)	Missense	Likely Pathogenic	M	40	Not stated
12511281	2661	PRPH2	NM_000322.5	c.259_266del	p.(Asp87GlnfsTer87)	Frameshift	Pathogenic	F	64	Any other ethnic group
13099386	2661	PRPH2	NM_000322.5	c.259_266del	p.(Asp87GlnfsTer87)	Frameshift	Pathogenic	F	45	Any other ethnic group
12662523	2662	PRPH2	NM_000322.5	c.634A>G	p.(Ser212Gly)	Missense	Pathogenic	F	36	White - Irish
8009189	2663	PRPH2	NM_000322.5	c.259_266del	p.(Asp87GlnfsTer87)	Frameshift	Pathogenic	M	70	White - British
12789629	2664	PRPH2	NM_000322.5	c.514C>T	p.(Arg172Trp)	Missense	Likely Pathogenic	F	48	White - British
10230695	2665	PRPH2	NM_000322.5	c.715C>T	p.(Gln239Ter)	Stopgain	Pathogenic	F	69	White - Other
17443138	2665	PRPH2	NM_000322.5	c.715C>T	p.(Gln239Ter)	Stopgain	Pathogenic	F	46	White - Other
12979735	2666	PRPH2	NM_000322.5	c.623G>A	p.(Gly208Asp)	Missense	Pathogenic	F	49	Unknown
12932338	2667	PRPH2	NM_000322.5	c.394del	p.(Gln132LysfsTer7)	Frameshift	Pathogenic	M	69	Not stated
12951805	2668	PRPH2	NM_000322.5	c.774del	p.(Tyr258Ter)	Stopgain	Likely Pathogenic	F	74	Not stated
12951819	2668	PRPH2	NM_000322.5	c.774del	p.(Tyr258Ter)	Stopgain	Likely Pathogenic	M	45	White - British
12343050	2669	PRPH2	NM_000322.5	c.394del	p.(Gln132LysfsTer7)	Frameshift	Pathogenic	F	45	Unknown
12851761	2670	PRPH2	NM_000322.5	c.499G>A	p.(Gly167Ser)	Missense	Pathogenic	F	52	White - British
13105959	2671	PRPH2	NM_000322.5	c.515G>A	p.(Arg172Gln)	Missense	Pathogenic	M	64	Unknown
12764429	2672	PRPH2	NM_000322.5	c.499G>A	p.(Gly167Ser)	Missense	Pathogenic	F	67	Black - Caribbean
11388663	2673	PRPH2	NM_000322.5	c.658C>T	p.(Arg220Trp)	Missense	Likely Pathogenic	M	64	White - British
13155708	2674	PRPH2	NM_000322.5	c.316del	p.(Val106PhefsTer33)	Frameshift	Likely Pathogenic	M	59	Not stated
13483161	2675	PRPH2	NM_000322.5	c.700dup	p.(Tyr234LeufsTer67)	Frameshift	Pathogenic	F	65	Unknown
8744882	2676	PRPH2	NM_000322.5	c.515G>A	p.(Arg172Gln)	Missense	Pathogenic	F	61	Not stated
6883624	2677	PRPH2	NM_000322.5	c.634A>G	p.(Ser212Gly)	Missense	Pathogenic	M	62	White - British
11022780	2678	PRPH2	NM_000322.5	c.380A>G	p.(Glu127Gly)	Missense	Variant of Uncertain Significance	M	65	Not stated
13576170	2679	PRPH2	NM_000322.5	c.612C>G	p.(Tyr204Ter)	Stopgain	Pathogenic	M	64	White - Other
12771135	2680	PRPH2	NM_000322.5	c.394del	p.(Gln132LysfsTer7)	Frameshift	Pathogenic	F	48	Unknown
5220655	2681	PRPH2	NM_000322.5	c.665G>C	p.(Cys222Ser)	Missense	Pathogenic	M	54	Any other ethnic group
13986230	2682	PRPH2	NM_000322.5	c.638G>A	p.(Cys213Tyr)	Missense	Likely Pathogenic	M	55	Not stated
9539109	2683	PRPH2	NM_000322.5	c.634A>G	p.(Ser212Gly)	Missense	Pathogenic	F	76	White - British
14750182	2684	PRPH2	NM_000322.5	c.136C>T	p.(Arg46Ter)	Stopgain	Pathogenic	M	51	Not stated
14826860	2685	PRPH2	NM_000322.5	c.620_627delinsTA	p.(Asp207_Gly208del)	Inframe indel	Likely Pathogenic	F	10	Not stated
14826860	2685	PRPH2	NM_000322.5	c.620_627delinsTA	p.(Asp207_Gly208del)	Inframe indel	Likely Pathogenic	F	10	Not stated
14762915	2686	PRPH2	NM_000322.5	c.638G>A	p.(Cys213Tyr)	Missense	Likely Pathogenic	F	59	Unknown
13143360	2687	PRPH2	NM_000322.5	c.749G>T	p.(Cys250Phe)	Missense	Pathogenic	F	55	White - British
15264927	2688	PRPH2	NM_000322.5	c.647C>T	p.(Pro216Leu)	Missense	Pathogenic	F	59	Not stated
15011884	2689	PRPH2	NM_000322.5	c.638G>A	p.(Cys213Tyr)	Missense	Likely Pathogenic	F	58	Any other ethnic group
11255327	2690	PRPH2	NM_000322.5	c.469G>A	p.(Asp157Asn)	Missense	Pathogenic	F	66	White - British
14913072	2691	PRPH2	NM_000322.5	c.514C>T	p.(Arg172Trp)	Missense	Likely Pathogenic	F	56	White - British

15280124	2692	PRPH2	NM_000322.5	c.658C>T	p.(Arg220Trp)	Missense	Likely Pathogenic	F	59	White - British
15486519	2693	PRPH2	NM_000322.5	c.623G>A	p.(Gly208Asp)	Missense	Pathogenic	M	55	White - British
14832936	2694	PRPH2	NM_000322.5	c.394del	p.(Gln132LysfsTer7)	Frameshift	Pathogenic	M	50	White - British
15499224	2695	PRPH2	NM_000322.5	c.77del	p.(Phe26SerfsTer12)	Frameshift	Likely Pathogenic	F	63	Unknown
15549680	2696	PRPH2	NM_000322.5	c.80C>T	p.(Ser27Phe)	Missense	Likely Pathogenic	M	71	White - British
15473471	2697	PRPH2	NM_000322.5	c.643A>T	p.(Asn215Tyr)	Missense	Pathogenic	F	50	Not stated
15633806	2698	PRPH2	NM_000322.5	c.77del	p.(Phe26SerfsTer12)	Frameshift	Likely Pathogenic	F	45	White - British
15571310	2699	PRPH2	NM_000322.5	c.498C>G	p.(Cys166Trp)	Missense	Likely Pathogenic	F	66	Any other ethnic group
15751301	2700	PRPH2	NM_000322.5	c.136C>T	p.(Arg46Ter)	Stopgain	Pathogenic	F	64	Not stated
13900543	2701	PRPH2	NM_000322.5	c.401T>C	p.(Leu134Pro)	Missense	Variant of Uncertain Significance	M	56	White - British
15817003	2702	PRPH2	NM_000322.5	c.774del	p.(Tyr258Ter)	Stopgain	Likely Pathogenic	M	65	White - British
15768878	2703	PRPH2	NM_000322.5	c.940dup	p.(Glu314GlyfsTer78)	Frameshift	Likely Pathogenic	M	41	Asian - Indian
15966250	2704	PRPH2	NM_000322.5	c.537G>T	p.(Trp179Cys)	Missense	Pathogenic	M	56	Not stated
13980938	2705	PRPH2	NM_000322.5	c.476T>C	p.(Leu159Pro)	Missense	Likely Pathogenic	M	34	Asian - Pakistani
18466370	2705	PRPH2	NM_000322.5	c.476T>C	p.(Leu159Pro)	Missense	Likely Pathogenic	M	67	Unknown
16000158	2706	PRPH2	NM_000322.5	c.695C>T	p.(Ala232Val)	Missense	Likely Pathogenic	M	70	Not stated
16167934	2707	PRPH2	NM_000322.5	c.499G>A	p.(Gly167Ser)	Missense	Pathogenic	M	43	Not stated
8771825	2708	PRPH2	NM_000322.5	c.589A>T	p.(Lys197Ter)	Stopgain	Likely Pathogenic	M	56	White - British
7840552	2709	PRPH2	NM_000322.5	c.514C>T	p.(Arg172Trp)	Missense	Likely Pathogenic	M	39	Black - Other
15810640	2710	PRPH2	NM_000322.5	c.584G>A	p.(Arg195Gln)	Missense	Pathogenic	M	69	Any other ethnic group
15661078	2711	PRPH2	NM_000322.5	c.596A>G	p.(Asn199Ser)	Missense	Variant of Uncertain Significance	M	51	Not stated
16591364	2712	PRPH2	NM_000322.5	c.828+3A>G	Splice	Splice	Variant of Uncertain Significance	M	71	White - Other
16379572	2713	PRPH2	NM_000322.5	c.500G>A	p.(Gly167Asp)	Missense	Likely Pathogenic	F	73	White - British
16834180	2714	PRPH2	NM_000322.5	c.424C>T	p.(Arg142Trp)	Missense	Pathogenic	F	36	White - British
10377184	2715	PRPH2	NM_000322.5	c.77del	p.(Phe26SerfsTer12)	Frameshift	Likely Pathogenic	F	75	Not stated
17156082	2715	PRPH2	NM_000322.5	c.77del	p.(Phe26SerfsTer12)	Frameshift	Likely Pathogenic	F	57	Not stated
16809386	2716	PRPH2	NM_000322.5	c.136C>T	p.(Arg46Ter)	Stopgain	Pathogenic	F	55	Not stated
10101370	2717	PRPH2	NM_000322.5	c.598G>A	p.(Val200Met)	Missense	Likely Pathogenic	M	61	Not stated
16622304	2718	PRPH2	NM_000322.5	c.648del	p.(Ser217AlafsTer39)	Frameshift	Likely Pathogenic	M	35	Not stated
17061876	2719	PRPH2	NM_000322.5	c.133del	p.(Leu45SerfsTer7)	Frameshift	Likely Pathogenic	M	62	Not stated
16888794	2720	PRPH2	NM_000322.5	c.126_127del	p.(Lys42AsnfsTer2)	Frameshift	Likely Pathogenic	F	56	Unknown
16404478	2721	PRPH2	NM_000322.5	c.394del	p.(Gln132LysfsTer7)	Frameshift	Pathogenic	F	52	White - British
17279317	2722	PRPH2	NM_000322.5	c.469G>A	p.(Asp157Asn)	Missense	Pathogenic	M	53	Not stated
17441626	2723	PRPH2	NM_000322.5	c.603_620del	p.(Arg203_Gly208del)	Inframe indel	Pathogenic	F	49	Not stated
17499698	2724	PRPH2	NM_000322.5	c.828+3A>T	Splice	Splice	Pathogenic	M	38	Not stated
17510695	2725	PRPH2	NM_000322.5	c.598G>A	p.(Val200Met)	Missense	Likely Pathogenic	M	51	Not stated
8478140	2726	PRPH2	NM_000322.5	c.394del	p.(Gln132LysfsTer7)	Frameshift	Pathogenic	M	65	Not stated
17842712	2727	PRPH2	NM_000322.5	c.424C>T	p.(Arg142Trp)	Missense	Pathogenic	F	52	Not stated
5450857	2728	PRPH2	NM_000322.5	c.623G>A	p.(Gly208Asp)	Missense	Pathogenic	M	33	Not stated
11457361	2729	PRPH2	NM_000322.5	c.715del	p.(Gln239ArgfsTer17)	Frameshift	Likely Pathogenic	M	61	Asian - Indian
6203385	2730	PRPH2	NM_000322.5	c.394del	p.(Gln132LysfsTer7)	Frameshift	Pathogenic	F	68	Not stated
16636185	2731	PRPH2	NM_000322.5	c.515G>A	p.(Arg172Gln)	Missense	Pathogenic	M	38	Not stated
18155682	2732	PRPH2	NM_000322.5	c.514C>T	p.(Arg172Trp)	Missense	Likely Pathogenic	F	33	Not stated
17968285	2733	PRPH2	NM_000322.5	c.612C>G	p.(Tyr204Ter)	Stopgain	Pathogenic	M	51	Not stated
17738951	2734	PRPH2	NM_000322.5	c.655_656insAATGC	p.(Pro219GlnfsTer39)	Frameshift	Likely Pathogenic	F	53	Not stated
18137454	2735	PRPH2	NM_000322.5	c.515G>A	p.(Arg172Gln)	Missense	Pathogenic	M	62	Unknown
18451705	2736	PRPH2	NM_000322.5	c.665G>C	p.(Cys222Ser)	Missense	Pathogenic	F	53	Not stated
18453945	2737	PRPH2	NM_000322.5	c.80C>T	p.(Ser27Phe)	Missense	Likely Pathogenic	M	33	Not stated
18628826	2737	PRPH2	NM_000322.5	c.80C>T	p.(Ser27Phe)	Missense	Likely Pathogenic	F	59	Not stated
18523693	2738	PRPH2	NM_000322.5	c.676C>T	p.(Gln226Ter)	Stopgain	Pathogenic	M	14	Not stated
18523693	2738	PRPH2	NM_000322.5	c.676C>T	p.(Gln226Ter)	Stopgain	Pathogenic	M	14	Not stated

18523896	2739	PRPH2	NM_000322.5	c.394del	p.(Gln132LysfsTer7)	Frameshift	Pathogenic	M	60	Not stated
18579014	2740	PRPH2	NM_000322.5	c.428A>T	p.(Asp143Val)	Missense	Likely Pathogenic	M	54	White - British
16247398	2741	PRPH2	NM_000322.5	c.394del	p.(Gln132LysfsTer7)	Frameshift	Pathogenic	M	34	Not stated
18409264	2742	PRPH2	NM_000322.5	c.259_266del	p.(Asp87GlnfsTer87)	Frameshift	Pathogenic	F	52	Not stated
17444965	2743	RAB28	NM_004249.4	c.58dup	p.(Asp20GlyfsTer62)	Frameshift	Likely Pathogenic	M	13	Not stated
17444965	2743	RAB28	NM_004249.4	c.58dup	p.(Asp20GlyfsTer62)	Frameshift	Likely Pathogenic	M	13	Not stated
15009644	2744	RAX2	NM_032753.4	c.247C>T	p.(Arg83Cys)	Missense	Pathogenic	F	46	Asian - Other
15009644	2744	RAX2	NM_032753.4	c.247C>T	p.(Arg83Cys)	Missense	Pathogenic	F	46	Asian - Other
13008204	2745	RAX2	NM_032753.4	c.247C>T	p.(Arg83Cys)	Missense	Pathogenic	M	50	Any other ethnic group
13008204	2745	RAX2	NM_032753.4	c.92G>A	p.(Arg31His)	Missense	Likely Pathogenic	M	50	Any other ethnic group
3922050	975	RAX2	NM_032753.4	c.247C>T	p.(Arg83Cys)	Missense	Pathogenic	M	70	Any other ethnic group
3922050	975	RAX2	NM_032753.4	c.247C>T	p.(Arg83Cys)	Missense	Pathogenic	M	70	Any other ethnic group
10179126	2746	RBP3	NM_002900.3	c.832_834del	p.(Phe278del)	Inframe indel	Likely Pathogenic	M	33	Not stated
10179126	2746	RBP3	NM_002900.3	c.832_834del	p.(Phe278del)	Inframe indel	Likely Pathogenic	M	33	Not stated
11837706	2747	RBP3	NM_002900.3	c.160C>T	p.(Gln54Ter)	Stopgain	Pathogenic	F	26	Asian - Pakistani
11837706	2747	RBP3	NM_002900.3	c.160C>T	p.(Gln54Ter)	Stopgain	Pathogenic	F	26	Asian - Pakistani
13076335	2748	RBP4	NM_006744.4	c.248+1G>A	Splice	Splice	Pathogenic	F	33	Not stated
13076335	2748	RBP4	NM_006744.4	c.248+1G>A	Splice	Splice	Pathogenic	F	33	Not stated
2103870	2749	RDH12	NM_152443.3	c.454T>A	p.(Phe152Ile)	Missense	Likely Pathogenic	M	56	Any other ethnic group
2103870	2749	RDH12	NM_152443.3	c.454T>A	p.(Phe152Ile)	Missense	Likely Pathogenic	M	56	Any other ethnic group
2628891	2750	RDH12	NM_152443.3	c.715C>T	p.(Arg239Trp)	Missense	Pathogenic	F	41	White - British
2628891	2750	RDH12	NM_152443.3	c.806_810del	p.(Ala269GlyfsTer2)	Frameshift	Pathogenic	F	41	White - British
6949564	2750	RDH12	NM_152443.3	c.715C>T	p.(Arg239Trp)	Missense	Pathogenic	M	28	White - British
6949564	2750	RDH12	NM_152443.3	c.806_810del	p.(Ala269GlyfsTer2)	Frameshift	Pathogenic	M	28	White - British
2720360	2751	RDH12	NM_152443.3	c.619A>G	p.(Asn207Asp)	Missense	Likely Pathogenic	F	55	Not stated
2720360	2751	RDH12	NM_152443.3	c.619A>G	p.(Asn207Asp)	Missense	Likely Pathogenic	F	55	Not stated
2790948	2752	RDH12	NM_152443.3	c.601T>C	p.(Cys201Arg)	Missense	Likely Pathogenic	F	41	Asian - Indian
2790948	2752	RDH12	NM_152443.3	c.601T>C	p.(Cys201Arg)	Missense	Likely Pathogenic	F	41	Asian - Indian
1397913	2753	RDH12	NM_152443.3	c.295C>A	p.(Leu99Ile)	Missense	Pathogenic	F	57	White - Other
1397913	2753	RDH12	NM_152443.3	c.883C>T	p.(Arg295Ter)	Stopgain	Pathogenic	F	57	White - Other
5853035	2754	RDH12	NM_152443.3	c.506G>A	p.(Arg169Gln)	Missense	Pathogenic	M	34	Not stated
5853035	2754	RDH12	NM_152443.3	c.506G>A	p.(Arg169Gln)	Missense	Pathogenic	M	34	Not stated
4922350	2755	RDH12	NM_152443.3	c.601T>C	p.(Cys201Arg)	Missense	Likely Pathogenic	F	34	Not stated
4922350	2755	RDH12	NM_152443.3	c.601T>C	p.(Cys201Arg)	Missense	Likely Pathogenic	F	34	Not stated
3477018	2756	RDH12	NM_152443.3	c.481C>T	p.(Arg161Trp)	Missense	Likely Pathogenic	M	54	White - Other
3477018	2756	RDH12	NM_152443.3	c.715dup	p.(Arg239ProfsTer34)	Frameshift	Pathogenic	M	54	White - Other
2146416	2757	RDH12	NM_152443.3	c.609C>A	p.(Ser203Arg)	Missense	Pathogenic	M	49	Not stated
2146416	2757	RDH12	NM_152443.3	c.609C>A	p.(Ser203Arg)	Missense	Pathogenic	M	49	Not stated
5682970	2758	RDH12	NM_152443.3	c.778del	p.(Glu260ArgfsTer18)	Frameshift	Pathogenic	M	38	Not stated
5767894	2759	RDH12	NM_152443.3	c.481C>T	p.(Arg161Trp)	Missense	Likely Pathogenic	M	47	White - British
5767894	2759	RDH12	NM_152443.3	c.806_810del	p.(Ala269GlyfsTer2)	Frameshift	Pathogenic	M	47	White - British
5745249	2760	RDH12	NM_152443.3	c.609C>A	p.(Ser203Arg)	Missense	Pathogenic	F	41	Asian - Other
5745249	2760	RDH12	NM_152443.3	c.609C>A	p.(Ser203Arg)	Missense	Pathogenic	F	41	Asian - Other
136513	2761	RDH12	NM_152443.3	c.146C>A	p.(Thr49Lys)	Missense	Pathogenic	M	49	Not stated
136513	2761	RDH12	NM_152443.3	c.146C>A	p.(Thr49Lys)	Missense	Pathogenic	M	49	Not stated
8976673	2762	RDH12	NM_152443.3	c.505C>T	p.(Arg169Trp)	Missense	Pathogenic	M	22	White - British
8976673	2762	RDH12	NM_152443.3	c.524C>T	p.(Ser175Leu)	Missense	Pathogenic	M	22	White - British
5219381	2763	RDH12	NM_152443.3	c.601T>C	p.(Cys201Arg)	Missense	Likely Pathogenic	F	48	Asian - Indian
5219381	2763	RDH12	NM_152443.3	c.601T>C	p.(Cys201Arg)	Missense	Likely Pathogenic	F	48	Asian - Indian
5506150	2764	RDH12	NM_152443.3	c.193C>T	p.(Arg65Ter)	Stopgain	Pathogenic	F	40	Asian - Bangladeshi
5506150	2764	RDH12	NM_152443.3	c.193C>T	p.(Arg65Ter)	Stopgain	Pathogenic	F	40	Asian - Bangladeshi

9329634	2765	RDH12	NM_152443.3	c.316C>T	p.(Arg106Ter)	Stopgain	Likely Pathogenic	F	50	White - British
9329634	2765	RDH12	NM_152443.3	c.806_810del	p.(Ala269GlyfsTer2)	Frameshift	Pathogenic	F	50	White - British
15837086	2766	RDH12	NM_152443.3	c.451C>G	p.(His151Asp)	Missense	Pathogenic	F	22	White - British
15837086	2766	RDH12	NM_152443.3	c.806_810del	p.(Ala269GlyfsTer2)	Frameshift	Pathogenic	F	22	White - British
8166227	2767	RDH12	NM_152443.3	c.506G>A	p.(Arg169Gln)	Missense	Pathogenic	M	26	Asian - Pakistani
8166227	2767	RDH12	NM_152443.3	c.506G>A	p.(Arg169Gln)	Missense	Pathogenic	M	26	Asian - Pakistani
9562636	2768	RDH12	NM_152443.3	c.184C>T	p.(Arg62Ter)	Stopgain	Pathogenic	F	28	White - British
9562636	2768	RDH12	NM_152443.3	c.806_810del	p.(Ala269GlyfsTer2)	Frameshift	Pathogenic	F	28	White - British
7041418	2769	RDH12	NM_152443.3	c.599A>G	p.(Tyr200Cys)	Missense	Pathogenic	F	36	Asian - Other
7041418	2769	RDH12	NM_152443.3	c.599A>G	p.(Tyr200Cys)	Missense	Pathogenic	F	36	Asian - Other
10138442	2770	RDH12	NM_152443.3	c.619A>G	p.(Asn207Asp)	Missense	Likely Pathogenic	F	35	Asian - Pakistani
10138442	2770	RDH12	NM_152443.3	c.619A>G	p.(Asn207Asp)	Missense	Likely Pathogenic	F	35	Asian - Pakistani
10224094	2771	RDH12	NM_152443.3	c.379G>T	p.(Gly127Ter)	Stopgain	Pathogenic	M	35	White - Other
10224094	2771	RDH12	NM_152443.3	c.379G>T	p.(Gly127Ter)	Stopgain	Pathogenic	M	35	White - Other
9350165	2772	RDH12	NM_152443.3	c.609C>A	p.(Ser203Arg)	Missense	Pathogenic	M	43	Not stated
9350165	2772	RDH12	NM_152443.3	c.609C>A	p.(Ser203Arg)	Missense	Pathogenic	M	43	Not stated
4367810	2773	RDH12	NM_152443.3	c.601T>C	p.(Cys201Arg)	Missense	Likely Pathogenic	F	53	Not stated
4367810	2773	RDH12	NM_152443.3	c.601T>C	p.(Cys201Arg)	Missense	Likely Pathogenic	F	53	Not stated
1027305	2773	RDH12	NM_152443.3	c.601T>C	p.(Cys201Arg)	Missense	Likely Pathogenic	M	52	Asian - Indian
1027305	2773	RDH12	NM_152443.3	c.601T>C	p.(Cys201Arg)	Missense	Likely Pathogenic	M	52	Asian - Indian
10382105	2774	RDH12	NM_152443.3	c.209G>A	p.(Cys70Tyr)	Missense	Pathogenic	M	20	Any other ethnic group
10382105	2774	RDH12	NM_152443.3	c.806_810del	p.(Ala269GlyfsTer2)	Frameshift	Pathogenic	M	20	Any other ethnic group
9613841	2775	RDH12	NM_152443.3	c.448+1G>A	Splice	Splice	Pathogenic	M	21	ed - White and Black Caribbean
9613841	2775	RDH12	NM_152443.3	c.697_698insGT	p.(Arg234SerfsTer45)	Frameshift	Likely Pathogenic	M	21	ed - White and Black Caribbean
10678219	2776	RDH12	NM_152443.3	c.146C>T	p.(Thr49Met)	Missense	Pathogenic	F	67	Asian - Indian
10678219	2776	RDH12	NM_152443.3	c.146C>T	p.(Thr49Met)	Missense	Pathogenic	F	67	Asian - Indian
11447932	2777	RDH12	NM_152443.3	c.506G>A	p.(Arg169Gln)	Missense	Pathogenic	F	45	Unknown
11447932	2777	RDH12	NM_152443.3	c.57_59del	p.(Pro20del)	Inframe indel	Variant of Uncertain Significance	F	45	Unknown
11532114	2778	RDH12	NM_152443.3	c.142A>G	p.(Asn48Asp)	Missense	Likely Pathogenic	M	31	White - British
11532114	2778	RDH12	NM_152443.3	c.316C>T	p.(Arg106Ter)	Stopgain	Likely Pathogenic	M	31	White - British
12643098	2779	RDH12	NM_152443.3	c.383T>G	p.(Val128Gly)	Missense	Variant of Uncertain Significance	F	13	Unknown
12643098	2779	RDH12	NM_152443.3	c.910T>C	p.(Trp304Arg)	Missense	Variant of Uncertain Significance	F	13	Unknown
11578230	2780	RDH12	NM_152443.3	c.763del	p.(Val255SerfsTer23)	Frameshift	Likely Pathogenic	F	47	Not stated
13607999	2780	RDH12	NM_152443.3	c.763del	p.(Val255SerfsTer23)	Frameshift	Likely Pathogenic	F	37	Not stated
13613347	2781	RDH12	NM_152443.3	c.806_810del	p.(Ala269GlyfsTer2)	Frameshift	Pathogenic	F	37	Not stated
13613347	2781	RDH12	NM_152443.3	Exon 3-8 deletion	Deletion	Deletion	Likely Pathogenic	F	37	Not stated
15866059	2782	RDH12	NM_152443.3	c.325G>C	p.(Ala109Pro)	Missense	Likely Pathogenic	F	9	Any other ethnic group
15866059	2782	RDH12	NM_152443.3	c.677A>G	p.(Tyr226Cys)	Missense	Pathogenic	F	9	Any other ethnic group
16192427	2783	RDH12	NM_152443.3	c.701G>A	p.(Arg234His)	Missense	Likely Pathogenic	M	15	White - British
16192427	2783	RDH12	NM_152443.3	c.735_743del	p.(Cys245_Leu247del)	Inframe indel	Variant of Uncertain Significance	M	15	White - British
17082666	2784	RDH12	NM_152443.3	c.601T>C	p.(Cys201Arg)	Missense	Likely Pathogenic	M	41	Not stated
17082666	2784	RDH12	NM_152443.3	c.601T>C	p.(Cys201Arg)	Missense	Likely Pathogenic	M	41	Not stated
15878463	2785	RDH12	NM_152443.3	c.464C>T	p.(Thr155Ile)	Missense	Pathogenic	M	11	Any other ethnic group
15878463	2785	RDH12	NM_152443.3	c.464C>T	p.(Thr155Ile)	Missense	Pathogenic	M	11	Any other ethnic group
16974313	2786	RDH12	NM_152443.3	c.448+1G>A	Splice	Splice	Pathogenic	F	35	Any other ethnic group
16974313	2786	RDH12	NM_152443.3	c.701G>A	p.(Arg234His)	Missense	Likely Pathogenic	F	35	Any other ethnic group
17630773	2787	RDH12	NM_152443.3	c.759del	p.(Phe254LeufsTer24)	Frameshift	Pathogenic	M	41	Not stated
18521523	2788	RDH12	NM_152443.3	c.601T>C	p.(Cys201Arg)	Missense	Likely Pathogenic	M	16	Not stated
18521523	2788	RDH12	NM_152443.3	c.601T>C	p.(Cys201Arg)	Missense	Likely Pathogenic	M	16	Not stated
2265549	2789	RDH5	NM_002905.5	c.-33+2dup	Splice	Splice	Variant of Uncertain Significance	M	71	Asian - Indian
2265549	2789	RDH5	NM_002905.5	c.758T>G	p.(Met253Arg)	Missense	Variant of Uncertain Significance	M	71	Asian - Indian

4508818	2790	RDH5	NM_002905.5	c.470G>A	p.(Arg157Gln)	Missense	Likely Pathogenic	M	51	Asian - Indian
4508818	2790	RDH5	NM_002905.5	c.470G>A	p.(Arg157Gln)	Missense	Likely Pathogenic	M	51	Asian - Indian
3977434	2791	RDH5	NM_002905.5	c.-33+2dup	Splice	Splice	Variant of Uncertain Significance	M	79	Asian - Indian
3977434	2791	RDH5	NM_002905.5	c.758T>G	p.(Met253Arg)	Missense	Variant of Uncertain Significance	M	79	Asian - Indian
3535426	2792	RDH5	NM_002905.5	c.824_825del	p.(Arg275ProfsTer60)	Frameshift	Likely Pathogenic	F	42	Unknown
3535426	2792	RDH5	NM_002905.5	c.824_825del	p.(Arg275ProfsTer60)	Frameshift	Likely Pathogenic	F	42	Unknown
6882434	2793	RDH5	NM_002905.5	c.712G>T	p.(Gly238Trp)	Missense	Pathogenic	M	58	White - British
6882434	2793	RDH5	NM_002905.5	c.98T>C	p.(Ile33Thr)	Missense	Likely Pathogenic	M	58	White - British
9805102	2794	RDH5	NM_002905.5	c.416G>T	p.(Gly139Val)	Missense	Pathogenic	M	34	Unknown
9805102	2794	RDH5	NM_002905.5	c.955T>C	p.(Ter319ArgextTer32)	Stoploss	Pathogenic	M	34	Unknown
10292512	2795	RDH5	NM_002905.5	c.712G>T	p.(Gly238Trp)	Missense	Pathogenic	F	22	Asian - Pakistani
10292512	2795	RDH5	NM_002905.5	c.712G>T	p.(Gly238Trp)	Missense	Pathogenic	F	22	Asian - Pakistani
10526410	2796	RDH5	NM_002905.5	c.310+1G>A	Splice	Splice	Likely Pathogenic	M	63	Unknown
10526410	2796	RDH5	NM_002905.5	c.712G>T	p.(Gly238Trp)	Missense	Pathogenic	M	63	Unknown
10446995	2797	RDH5	NM_002905.5	c.346G>C	p.(Gly116Arg)	Missense	Variant of Uncertain Significance	M	69	Not stated
10446995	2797	RDH5	NM_002905.5	c.710A>C	p.(Tyr237Ser)	Missense	Variant of Uncertain Significance	M	69	Not stated
11021345	2798	RDH5	NM_002905.5	c.602C>T	p.(Ser201Phe)	Missense	Variant of Uncertain Significance	M	49	Asian - Other
11021345	2798	RDH5	NM_002905.5	c.602C>T	p.(Ser201Phe)	Missense	Variant of Uncertain Significance	M	49	Asian - Other
17060777	2799	RDH5	NM_002905.5	c.602C>T	p.(Ser201Phe)	Missense	Variant of Uncertain Significance	M	65	Not stated
17060777	2799	RDH5	NM_002905.5	c.602C>T	p.(Ser201Phe)	Missense	Variant of Uncertain Significance	M	65	Not stated
17916954	2800	RDH5	NM_002905.5	c.371T>C	p.(Leu124Pro)	Missense	Variant of Uncertain Significance	M	27	Unknown
17916954	2800	RDH5	NM_002905.5	c.392G>A	p.(Arg131Gln)	Missense	Variant of Uncertain Significance	M	27	Unknown
17916954	2800	RDH5	NM_002905.5	c.592del	p.(Ile198TyrfsTer15)	Frameshift	Pathogenic	M	27	Unknown
18108789	2801	RDH5	NM_002905.5	c.310+1_310+5del	Splice	Splice	Likely Pathogenic	F	46	Not stated
18108789	2801	RDH5	NM_002905.5	c.310+1_310+5del	Splice	Splice	Likely Pathogenic	F	46	Not stated
18513851	2802	RDH5	NM_002905.5	c.500G>A	p.(Arg167His)	Missense	Likely Pathogenic	F	53	Asian - Bangladeshi
18513851	2802	RDH5	NM_002905.5	c.678G>A	p.(Trp226Ter)	Stopgain	Pathogenic	F	53	Asian - Bangladeshi
10776184	2803	REEP6	NM_001329556.3	c.337_338insC	p.(Tyr113SerfsTer68)	Frameshift	Likely Pathogenic	M	62	Not stated
10776184	2803	REEP6	NM_001329556.3	c.337_338insC	p.(Tyr113SerfsTer68)	Frameshift	Likely Pathogenic	M	62	Not stated
12782629	2804	REEP6	NM_001329556.3	c.383C>T	p.(Pro128Leu)	Missense	Variant of Uncertain Significance	M	40	Any other ethnic group
12782629	2804	REEP6	NM_001329556.3	c.383C>T	p.(Pro128Leu)	Missense	Variant of Uncertain Significance	M	40	Any other ethnic group
15325715	2805	REEP6	NM_001329556.3	c.279_280del	p.(Leu94ValfsTer86)	Frameshift	Likely Pathogenic	F	45	Asian - Pakistani
15325715	2805	REEP6	NM_001329556.3	c.279_280del	p.(Leu94ValfsTer86)	Frameshift	Likely Pathogenic	F	45	Asian - Pakistani
3756528	2806	RGR	NM_002921.3	c.836dup	p.(Ile280AsnfsTer78)	Frameshift	Pathogenic	F	84	Unknown
1673916	2807	RHO	NM_000539.3	c.1040C>T	p.(Pro347Leu)	Missense	Pathogenic	M	63	White - British
189713	2807	RHO	NM_000539.3	c.1040C>T	p.(Pro347Leu)	Missense	Pathogenic	M	91	White - British
4582640	2807	RHO	NM_000539.3	c.1040C>T	p.(Pro347Leu)	Missense	Pathogenic	F	33	Not stated
10738615	2808	RHO	NM_000539.3	c.116T>G	p.(Met39Arg)	Missense	Likely Pathogenic	F	60	White - British
1283253	2808	RHO	NM_000539.3	c.116T>G	p.(Met39Arg)	Missense	Likely Pathogenic	M	95	Not stated
16858337	2808	RHO	NM_000539.3	c.116T>G	p.(Met39Arg)	Missense	Likely Pathogenic	M	74	Not stated
229900	2809	RHO	NM_000539.3	c.1040C>T	p.(Pro347Leu)	Missense	Pathogenic	F	58	White - British
1067107	2810	RHO	NM_000539.3	c.1040C>T	p.(Pro347Leu)	Missense	Pathogenic	M	76	White - British
121372	2810	RHO	NM_000539.3	c.1040C>T	p.(Pro347Leu)	Missense	Pathogenic	F	49	White - British
530361	2811	RHO	NM_000539.3	c.937-24_943delins150	Splice	Splice	Likely Pathogenic	F	51	Not stated
65232	2812	RHO	NM_000539.3	c.1040C>T	p.(Pro347Leu)	Missense	Pathogenic	F	72	White - British
2750845	2813	RHO	NM_000539.3	c.1040C>T	p.(Pro347Leu)	Missense	Pathogenic	M	62	Not stated
6913500	2814	RHO	NM_000539.3	c.647T>A	p.(Met216Lys)	Missense	Likely Pathogenic	M	67	White - British
6826350	2814	RHO	NM_000539.3	c.647T>A	p.(Met216Lys)	Missense	Likely Pathogenic	M	35	White - British
9419556	2815	RHO	NM_000539.3	c.116T>G	p.(Met39Arg)	Missense	Likely Pathogenic	F	57	Not stated
17745391	2815	RHO	NM_000539.3	c.116T>G	p.(Met39Arg)	Missense	Likely Pathogenic	M	30	Not stated
8871372	2816	RHO	NM_000539.3	c.1040C>T	p.(Pro347Leu)	Missense	Pathogenic	F	59	Not stated

12782489	2817	RHO	NM_000539.3	c.173C>G	p.(Thr58Arg)	Missense	Pathogenic	M	55	Any other ethnic group
11354958	2817	RHO	NM_000539.3	c.173C>G	p.(Thr58Arg)	Missense	Pathogenic	F	79	Unknown
14921878	2817	RHO	NM_000539.3	c.173C>G	p.(Thr58Arg)	Missense	Pathogenic	F	51	Any other ethnic group
16351558	2818	RHO	NM_000539.3	c.553T>C	p.(Cys185Arg)	Missense	Pathogenic	F	58	Not stated
13613389	2819	RHO	NM_000539.3	c.560G>A	p.(Cys187Tyr)	Missense	Pathogenic	F	44	Not stated
10243015	2820	RHO	NM_000539.3	c.1033G>A	p.(Val345Met)	Missense	Pathogenic	F	50	Not stated
783579	2821	RHO	NM_000539.3	c.316G>A	p.(Gly106Arg)	Missense	Likely Pathogenic	F	62	Not stated
862413	2822	RHO	NM_000539.3	c.937-1G>A	Splice	Splice	Likely Pathogenic	M	72	White - British
538432	2823	RHO	NM_000539.3	c.173C>G	p.(Thr58Arg)	Missense	Pathogenic	F	53	Not stated
3580807	2824	RHO	NM_000539.3	c.533A>G	p.(Tyr178Cys)	Missense	Pathogenic	F	84	White - British
3341253	2825	RHO	NM_000539.3	c.1040C>T	p.(Pro347Leu)	Missense	Pathogenic	M	71	Not stated
9495933	2826	RHO	NM_000539.3	c.533A>G	p.(Tyr178Cys)	Missense	Pathogenic	M	84	Not stated
7338561	2827	RHO	NM_000539.3	c.541G>A	p.(Glu181Lys)	Missense	Pathogenic	F	71	White - British
7373099	2827	RHO	NM_000539.3	c.541G>A	p.(Glu181Lys)	Missense	Pathogenic	M	27	Not stated
6012810	2828	RHO	NM_000539.3	c.512C>T	p.(Pro171Leu)	Missense	Pathogenic	M	60	Not stated
9386670	2828	RHO	NM_000539.3	c.512C>T	p.(Pro171Leu)	Missense	Pathogenic	F	49	Not stated
1558395	2829	RHO	NM_000539.3	c.937-1G>T	Splice	Splice	Likely Pathogenic	M	72	White - Other
2992835	2830	RHO	NM_000539.3	c.568G>A	p.(Asp190Asn)	Missense	Pathogenic	F	69	White - British
14871660	2831	RHO	NM_000539.3	c.1033G>A	p.(Val345Met)	Missense	Pathogenic	F	52	Unknown
14930467	2831	RHO	NM_000539.3	c.1033G>A	p.(Val345Met)	Missense	Pathogenic	M	55	Unknown
17122468	2831	RHO	NM_000539.3	c.1033G>A	p.(Val345Met)	Missense	Pathogenic	F	34	White - British
11819072	2832	RHO	NM_000539.3	c.403C>T	p.(Arg135Trp)	Missense	Pathogenic	M	51	White - Other
11820031	2833	RHO	NM_000539.3	c.1033G>A	p.(Val345Met)	Missense	Pathogenic	F	29	Not stated
4812163	2833	RHO	NM_000539.3	c.1033G>A	p.(Val345Met)	Missense	Pathogenic	F	59	Not stated
90390	2834	RHO	NM_000539.3	c.644C>T	p.(Pro215Leu)	Missense	Pathogenic	M	59	Any other ethnic group
12219381	2835	RHO	NM_000539.3	c.173C>G	p.(Thr58Arg)	Missense	Pathogenic	M	49	White - British
3124813	2836	RHO	NM_000539.3	c.509C>G	p.(Pro170Arg)	Missense	Pathogenic	F	54	Unknown
330371	2837	RHO	NM_000539.3	c.568G>A	p.(Asp190Asn)	Missense	Pathogenic	M	75	White - British
6767011	2837	RHO	NM_000539.3	c.568G>A	p.(Asp190Asn)	Missense	Pathogenic	F	51	White - British
4151321	2838	RHO	NM_000539.3	c.403C>T	p.(Arg135Trp)	Missense	Pathogenic	M	39	White - British
3046756	2839	RHO	NM_000539.3	c.568G>A	p.(Asp190Asn)	Missense	Pathogenic	M	68	Any other ethnic group
2291162	2840	RHO	NM_000539.3	c.553T>C	p.(Cys185Arg)	Missense	Pathogenic	M	95	White - British
5125518	2841	RHO	NM_000539.3	c.541G>A	p.(Glu181Lys)	Missense	Pathogenic	F	59	Not stated
5151950	2842	RHO	NM_000539.3	c.204_215del	p.(Arg69_Leu72del)	Inframe indel	Pathogenic	M	81	Not stated
1929493	2843	RHO	NM_000539.3	c.83A>G	p.(Gln28Arg)	Missense	Pathogenic	M	86	White - British
6561568	2844	RHO	NM_000539.3	c.116T>G	p.(Met39Arg)	Missense	Likely Pathogenic	M	60	White - British
5634999	2844	RHO	NM_000539.3	c.116T>G	p.(Met39Arg)	Missense	Likely Pathogenic	F	80	White - British
5837789	2845	RHO	NM_000539.3	c.1040C>T	p.(Pro347Leu)	Missense	Pathogenic	F	76	White - British
12126141	2846	RHO	NM_000539.3	c.173C>G	p.(Thr58Arg)	Missense	Pathogenic	M	50	White - British
6994952	2847	RHO	NM_000539.3	c.512C>T	p.(Pro171Leu)	Missense	Pathogenic	F	70	Any other ethnic group
7216152	2848	RHO	NM_000539.3	c.316G>A	p.(Gly106Arg)	Missense	Likely Pathogenic	M	54	White - British
16968958	2848	RHO	NM_000539.3	c.316G>A	p.(Gly106Arg)	Missense	Likely Pathogenic	M	22	Not stated
7338722	2849	RHO	NM_000539.3	c.511C>A	p.(Pro171Thr)	Missense	Likely Pathogenic	F	70	Asian - Indian
7706663	2850	RHO	NM_000539.3	c.1039C>T	p.(Pro347Ser)	Missense	Likely Pathogenic	M	59	Not stated
7555155	2851	RHO	NM_000539.3	c.509C>G	p.(Pro170Arg)	Missense	Pathogenic	M	54	Mixed - Other
8094449	2852	RHO	NM_000539.3	c.165C>A	p.(Asn55Lys)	Missense	Pathogenic	F	52	White - British
13259231	2853	RHO	NM_000539.3	c.173C>G	p.(Thr58Arg)	Missense	Pathogenic	F	44	Any other ethnic group
13645211	2853	RHO	NM_000539.3	c.173C>G	p.(Thr58Arg)	Missense	Pathogenic	F	13	Not stated
17651087	2854	RHO	NM_000539.3	c.403C>T	p.(Arg135Trp)	Missense	Pathogenic	F	10	Not stated
9095981	2855	RHO	NM_000539.3	c.266G>A	p.(Gly89Asp)	Missense	Pathogenic	M	48	Not stated
10025098	2856	RHO	NM_000539.3	c.541G>A	p.(Glu181Lys)	Missense	Pathogenic	F	61	Not stated

43812	2857	RHO	NM_000539.3	c.533A>G	p.(Tyr178Cys)	Missense	Pathogenic	F	73	White - Irish
10333525	2858	RHO	NM_000539.3	c.316G>A	p.(Gly106Arg)	Missense	Likely Pathogenic	F	47	Not stated
11715178	2859	RHO	NM_000539.3	c.266G>A	p.(Gly89Asp)	Missense	Pathogenic	F	23	White - Other
10378829	2859	RHO	NM_000539.3	c.266G>A	p.(Gly89Asp)	Missense	Pathogenic	F	63	White - Other
10756332	2860	RHO	NM_000539.3	c.165C>A	p.(Asn55Lys)	Missense	Pathogenic	M	23	White - British
10756290	2860	RHO	NM_000539.3	c.165C>A	p.(Asn55Lys)	Missense	Pathogenic	M	27	White - British
10920230	2860	RHO	NM_000539.3	c.165C>A	p.(Asn55Lys)	Missense	Pathogenic	F	55	White - British
6933877	2861	RHO	NM_000539.3	c.886A>G	p.(Lys296Glu)	Missense	Pathogenic	M	67	Any other ethnic group
10803106	2861	RHO	NM_000539.3	c.886A>G	p.(Lys296Glu)	Missense	Pathogenic	F	23	White - British
11351038	2862	RHO	NM_000539.3	c.158C>G	p.(Pro53Arg)	Missense	Pathogenic	F	60	White - British
11278322	2863	RHO	NM_000539.3	c.541G>A	p.(Glu181Lys)	Missense	Pathogenic	F	31	White - British
11557244	2864	RHO	NM_000539.3	c.116T>G	p.(Met39Arg)	Missense	Likely Pathogenic	M	42	Not stated
7346905	2864	RHO	NM_000539.3	c.116T>G	p.(Met39Arg)	Missense	Likely Pathogenic	M	72	Not stated
11642644	2864	RHO	NM_000539.3	c.116T>G	p.(Met39Arg)	Missense	Likely Pathogenic	M	68	White - British
11681746	2865	RHO	NM_000539.3	c.512C>G	p.(Pro171Arg)	Missense	Pathogenic	M	48	Not stated
9035879	2866	RHO	NM_000539.3	c.541G>A	p.(Glu181Lys)	Missense	Pathogenic	F	44	White - Other
11181673	2867	RHO	NM_000539.3	c.1040C>T	p.(Pro347Leu)	Missense	Pathogenic	F	28	Asian - Pakistani
11798156	2868	RHO	NM_000539.3	c.886A>G	p.(Lys296Glu)	Missense	Pathogenic	F	41	White - British
11793816	2869	RHO	NM_000539.3	c.50C>T	p.(Thr17Met)	Missense	Pathogenic	M	56	Unknown
11751249	2870	RHO	NM_000539.3	c.1040C>T	p.(Pro347Leu)	Missense	Pathogenic	M	73	Unknown
11806563	2871	RHO	NM_000539.3	c.553T>C	p.(Cys185Arg)	Missense	Pathogenic	F	52	Unknown
10647398	2872	RHO	NM_000539.3	c.44A>G	p.(Asn15Ser)	Missense	Pathogenic	M	58	Not stated
8672719	2873	RHO	NM_000539.3	c.541G>A	p.(Glu181Lys)	Missense	Pathogenic	M	44	Not stated
11670007	2874	RHO	NM_000539.3	c.1040C>T	p.(Pro347Leu)	Missense	Pathogenic	M	35	White - British
12528116	2875	RHO	NM_000539.3	c.568G>A	p.(Asp190Asn)	Missense	Pathogenic	M	60	Not stated
12684426	2876	RHO	NM_000539.3	c.568G>T	p.(Asp190Tyr)	Missense	Pathogenic	F	36	Not stated
12544363	2877	RHO	NM_000539.3	c.1039C>T	p.(Pro347Ser)	Missense	Likely Pathogenic	F	42	Asian - Indian
13075551	2878	RHO	NM_000539.3	c.1040C>T	p.(Pro347Leu)	Missense	Pathogenic	M	21	Unknown
11068266	2879	RHO	NM_000539.3	c.1040C>T	p.(Pro347Leu)	Missense	Pathogenic	F	39	Asian - Indian
13177394	2880	RHO	NM_000539.3	c.886A>G	p.(Lys296Glu)	Missense	Pathogenic	F	46	Any other ethnic group
14719536	2880	RHO	NM_000539.3	c.886A>G	p.(Lys296Glu)	Missense	Pathogenic	F	22	Not stated
11538134	2881	RHO	NM_000539.3	c.512C>T	p.(Pro171Leu)	Missense	Pathogenic	F	39	Any other ethnic group
13486689	2882	RHO	NM_000539.3	c.953_955del	p.(Leu318_Thr319delinsPro)	Inframe indel	Likely Pathogenic	F	52	Any other ethnic group
13199444	2883	RHO	NM_000539.3	c.1040C>T	p.(Pro347Leu)	Missense	Pathogenic	M	63	Any other ethnic group
14756874	2884	RHO	NM_000539.3	c.266G>A	p.(Gly89Asp)	Missense	Pathogenic	F	62	Not stated
14931265	2885	RHO	NM_000539.3	c.545_546delinsAA	p.(Gly182Glu)	Missense	Pathogenic	M	21	Not stated
13135408	2886	RHO	NM_000539.3	c.568G>A	p.(Asp190Asn)	Missense	Pathogenic	M	42	Any other ethnic group
13209195	2887	RHO	NM_000539.3	c.328T>C	p.(Cys110Arg)	Missense	Pathogenic	M	76	White - British
15418388	2888	RHO	NM_000539.3	c.810C>A	p.(Ser270Arg)	Missense	Pathogenic	M	64	White - British
14996981	2889	RHO	NM_000539.3	c.647T>A	p.(Met216Lys)	Missense	Likely Pathogenic	F	73	White - British
15607122	2890	RHO	NM_000539.3	c.50C>T	p.(Thr17Met)	Missense	Pathogenic	F	40	White - British
17360825	2890	RHO	NM_000539.3	c.50C>T	p.(Thr17Met)	Missense	Pathogenic	M	80	Not stated
15638300	2891	RHO	NM_000539.3	c.68C>A	p.(Pro23His)	Missense	Pathogenic	M	19	White - British
12789265	2892	RHO	NM_000539.3	c.1040C>T	p.(Pro347Leu)	Missense	Pathogenic	F	40	Any other ethnic group
11343331	2893	RHO	NM_000539.3	c.328T>C	p.(Cys110Arg)	Missense	Pathogenic	M	67	Unknown
15911965	2894	RHO	NM_000539.3	c.173C>G	p.(Thr58Arg)	Missense	Pathogenic	F	34	Not stated
16167675	2895	RHO	NM_000539.3	c.1040C>T	p.(Pro347Leu)	Missense	Pathogenic	F	35	White - Other
16529379	2896	RHO	NM_000539.3	c.165C>A	p.(Asn55Lys)	Missense	Pathogenic	F	32	White - British
16705254	2897	RHO	NM_000539.3	c.541G>A	p.(Glu181Lys)	Missense	Pathogenic	F	33	White - British
8014712	2898	RHO	NM_000539.3	c.316G>A	p.(Gly106Arg)	Missense	Likely Pathogenic	F	49	Not stated
17093936	2899	RHO	NM_000539.3	c.1021G>A	p.(Glu341Lys)	Missense	Likely Pathogenic	M	37	White - Other

16056277	2900	RHO	NM_000539.3	c.448G>A	p.(Glu150Lys)	Missense	Pathogenic	F	47	White - British
17158077	2901	RHO	NM_000539.3	c.173C>G	p.(Thr58Arg)	Missense	Pathogenic	F	32	Not stated
17701109	2901	RHO	NM_000539.3	c.173C>G	p.(Thr58Arg)	Missense	Pathogenic	F	34	Unknown
16449670	2902	RHO	NM_000539.3	c.563G>A	p.(Gly188Glu)	Missense	Pathogenic	M	36	Not stated
16684198	2903	RHO	NM_000539.3	c.647T>A	p.(Met216Lys)	Missense	Likely Pathogenic	F	46	Not stated
13774809	2904	RHO	NM_000539.3	c.1040C>T	p.(Pro347Leu)	Missense	Pathogenic	F	12	Not stated
17107152	2905	RHO	NM_000539.3	c.403C>T	p.(Arg135Trp)	Missense	Pathogenic	F	47	Not stated
17119962	2906	RHO	NM_000539.3	c.1021G>A	p.(Glu341Lys)	Missense	Likely Pathogenic	F	54	Not stated
17367027	2907	RHO	NM_000539.3	c.173C>G	p.(Thr58Arg)	Missense	Pathogenic	F	27	White - Other
6971957	2908	RHO	NM_000539.3	c.562G>A	p.(Gly188Arg)	Missense	Pathogenic	F	50	Not stated
17546353	2909	RHO	NM_000539.3	c.1033G>A	p.(Val345Met)	Missense	Pathogenic	M	35	White - British
17402755	2910	RHO	NM_000539.3	c.173C>G	p.(Thr58Arg)	Missense	Pathogenic	F	44	White - British
16720080	2911	RHO	NM_000539.3	c.190C>T	p.(Gln64Ter)	Stopgain	Pathogenic	M	61	Not stated
15104543	2912	RHO	NM_000539.3	c.50C>T	p.(Thr17Met)	Missense	Pathogenic	F	39	Not stated
18176276	2913	RHO	NM_000539.3	c.532T>C	p.(Tyr178His)	Missense	Pathogenic	M	56	Not stated
10188569	2914	RHO	NM_000539.3	c.1030C>T	p.(Gln344Ter)	Stopgain	Pathogenic	M	50	Not stated
13926289	2915	RHO	NM_000539.3	c.766_777del	p.(Ile256_Ile259del)	Inframe indel	Variant of Uncertain Significance	F	52	Asian - Indian
18043465	2916	RHO	NM_000539.3	c.403C>T	p.(Arg135Trp)	Missense	Pathogenic	F	21	Not stated
18605922	2917	RHO	NM_000539.3	c.541G>A	p.(Glu181Lys)	Missense	Pathogenic	M	33	Not stated
16930122	2918	RHO	NM_000539.3	c.1021G>A	p.(Glu341Lys)	Missense	Likely Pathogenic	F	41	White - British
18653879	2919	RHO	NM_000539.3	c.553T>C	p.(Cys185Arg)	Missense	Pathogenic	M	66	Not stated
18677616	2920	RHO	NM_000539.3	c.1030C>G	p.(Gln344Glu)	Missense	Likely Pathogenic	F	54	Not stated
1374939	2921	RLBP1	NM_000326.5	c.12+2del	Splice	Splice	Pathogenic	F	83	White - British
1374939	2921	RLBP1	NM_000326.5	c.141+2T>C	Splice	Splice	Pathogenic	F	83	White - British
7128155	2922	RLBP1	NM_000326.5	c.286_297del	p.(Phe96_Phe99del)	Inframe indel	Likely Pathogenic	M	39	White - British
7128155	2922	RLBP1	NM_000326.5	c.286_297del	p.(Phe96_Phe99del)	Inframe indel	Likely Pathogenic	M	39	White - British
8199470	2923	RLBP1	NM_000326.5	c.32T>C	p.(Val11Ala)	Missense	Variant of Uncertain Significance	F	54	Any other ethnic group
8199470	2923	RLBP1	NM_000326.5	c.32T>C	p.(Val11Ala)	Missense	Variant of Uncertain Significance	F	54	Any other ethnic group
13553546	2924	RLBP1	NM_000326.5	c.701G>A	p.(Arg234Gln)	Missense	Likely Pathogenic	F	15	Not stated
13553546	2924	RLBP1	NM_000326.5	c.701G>A	p.(Arg234Gln)	Missense	Likely Pathogenic	F	15	Not stated
14797768	2925	RLBP1	NM_000326.5	c.466C>T	p.(Arg156Ter)	Stopgain	Pathogenic	M	35	White - British
14797768	2925	RLBP1	NM_000326.5	c.832del	p.(Gln278ArgfsTer51)	Frameshift	Likely Pathogenic	M	35	White - British
16793461	2926	RLBP1	NM_000326.5	c.141+2T>C	Splice	Splice	Pathogenic	M	58	White - British
16793461	2926	RLBP1	NM_000326.5	c.141+2T>C	Splice	Splice	Pathogenic	M	58	White - British
5191143	2927	RLBP1	NM_000326.5	c.466C>T	p.(Arg156Ter)	Stopgain	Pathogenic	M	55	Asian - Other
5191143	2927	RLBP1	NM_000326.5	c.466C>T	p.(Arg156Ter)	Stopgain	Pathogenic	M	55	Asian - Other
6936670	2928	RP1	NM_006269.2	c.2029C>T	p.(Arg677Ter)	Stopgain	Pathogenic	M	77	White - British
4547829	2929	RP1	NM_006269.2	c.2098G>T	p.(Glu700Ter)	Stopgain	Likely Pathogenic	F	57	Not stated
10577398	2930	RP1	NM_006269.2	c.2206dup	p.(Thr736AsnfsTer4)	Frameshift	Pathogenic	M	41	Not stated
12419105	2931	RP1	NM_006269.2	c.2232T>A	p.(Cys744Ter)	Stopgain	Pathogenic	M	40	Unknown
441468	2931	RP1	NM_006269.2	c.2232T>A	p.(Cys744Ter)	Stopgain	Pathogenic	F	72	Not stated
13926520	2931	RP1	NM_006269.2	c.2232T>A	p.(Cys744Ter)	Stopgain	Pathogenic	M	70	Unknown
16515106	2931	RP1	NM_006269.2	c.2232T>A	p.(Cys744Ter)	Stopgain	Pathogenic	F	69	Not stated
3173064	2932	RP1	NM_006269.2	c.2172_2185del	p.(Ile725ArgfsTer6)	Frameshift	Pathogenic	M	71	Unknown
7706831	2933	RP1	NM_006269.2	c.2172_2185del	p.(Ile725ArgfsTer6)	Frameshift	Pathogenic	M	86	White - British
18401606	2934	RP1	NM_006269.2	c.2029C>T	p.(Arg677Ter)	Stopgain	Pathogenic	M	27	White - British
13076370	2935	RP1	NM_006269.2	c.2613dup	p.(Arg872ThrfsTer2)	Frameshift	Pathogenic	M	68	White - British
10769492	2936	RP1	NM_006269.2	c.2172_2185del	p.(Ile725ArgfsTer6)	Frameshift	Pathogenic	M	44	Unknown
15691493	2936	RP1	NM_006269.2	c.2172_2185del	p.(Ile725ArgfsTer6)	Frameshift	Pathogenic	M	52	Not stated
17969055	2936	RP1	NM_006269.2	c.2172_2185del	p.(Ile725ArgfsTer6)	Frameshift	Pathogenic	M	33	Not stated
10851728	2937	RP1	NM_006269.2	c.2596_2597del	p.(Leu866LysfsTer7)	Frameshift	Pathogenic	M	77	White - British

11314575	2937	RP1	NM_006269.2	c.2596_2597del	p.(Leu866LysfsTer7)	Frameshift	Pathogenic	M	65	Unknown
9848012	2937	RP1	NM_006269.2	c.2596_2597del	p.(Leu866LysfsTer7)	Frameshift	Pathogenic	M	66	Unknown
5729345	2937	RP1	NM_006269.2	c.2596_2597del	p.(Leu866LysfsTer7)	Frameshift	Pathogenic	F	76	Not stated
11314540	2937	RP1	NM_006269.2	c.2596_2597del	p.(Leu866LysfsTer7)	Frameshift	Pathogenic	M	75	Unknown
9401699	2938	RP1	NM_006269.2	c.2018del	p.(Lys673ArgfsTer9)	Frameshift	Pathogenic	F	76	White - British
376529	2938	RP1	NM_006269.2	c.2018del	p.(Lys673ArgfsTer9)	Frameshift	Pathogenic	F	80	White - British
9944248	2939	RP1	NM_006269.2	c.2172_2185del	p.(Ile725ArgfsTer6)	Frameshift	Pathogenic	M	47	Not stated
1120377	2939	RP1	NM_006269.2	c.2172_2185del	p.(Ile725ArgfsTer6)	Frameshift	Pathogenic	M	73	White - British
377635	2940	RP1	NM_006269.2	c.2029C>T	p.(Arg677Ter)	Stopgain	Pathogenic	F	72	White - British
554756	2941	RP1	NM_006269.2	c.2098G>T	p.(Glu700Ter)	Stopgain	Likely Pathogenic	F	84	Not stated
3614988	2941	RP1	NM_006269.2	c.2098G>T	p.(Glu700Ter)	Stopgain	Likely Pathogenic	F	87	White - British
15119124	2941	RP1	NM_006269.2	c.2098G>T	p.(Glu700Ter)	Stopgain	Likely Pathogenic	F	58	Not stated
2685633	2942	RP1	NM_006269.2	c.2029C>T	p.(Arg677Ter)	Stopgain	Pathogenic	F	83	Unknown
5179180	2943	RP1	NM_006269.2	c.2613dup	p.(Arg872ThrfsTer2)	Frameshift	Pathogenic	F	80	White - British
17149054	2944	RP1	NM_006269.2	c.2172_2185del	p.(Ile725ArgfsTer6)	Frameshift	Pathogenic	F	79	Unknown
3939522	2944	RP1	NM_006269.2	c.2172_2185del	p.(Ile725ArgfsTer6)	Frameshift	Pathogenic	M	51	Not stated
1928996	2945	RP1	NM_006269.2	c.2029C>T	p.(Arg677Ter)	Stopgain	Pathogenic	M	89	Unknown
12365506	2946	RP1	NM_006269.2	c.2029C>T	p.(Arg677Ter)	Stopgain	Pathogenic	F	47	Not stated
4981605	2946	RP1	NM_006269.2	c.2029C>T	p.(Arg677Ter)	Stopgain	Pathogenic	F	101	Not stated
3171181	2947	RP1	NM_006269.2	c.2029C>T	p.(Arg677Ter)	Stopgain	Pathogenic	F	68	Not stated
18631136	2947	RP1	NM_006269.2	c.2029C>T	p.(Arg677Ter)	Stopgain	Pathogenic	M	76	Not stated
16701124	2948	RP1	NM_006269.2	c.2232T>A	p.(Cys744Ter)	Stopgain	Pathogenic	F	56	White - British
5686694	2949	RP1	NM_006269.2	c.2172_2185del	p.(Ile725ArgfsTer6)	Frameshift	Pathogenic	M	53	White - British
6612906	2950	RP1	NM_006269.2	c.148G>C	p.(Gly50Arg)	Missense	Variant of Uncertain Significance	M	38	White - British
3343276	2951	RP1	NM_006269.2	c.1498_1499del	p.(Met500ValfsTer7)	Frameshift	Pathogenic	M	54	Not stated
3343276	2951	RP1	NM_006269.2	c.515T>G	p.(Leu172Arg)	Missense	Likely Pathogenic	M	54	Not stated
4367992	2952	RP1	NM_006269.2	c.2172_2185del	p.(Ile725ArgfsTer6)	Frameshift	Pathogenic	F	85	White - British
10245031	2953	RP1	NM_006269.2	c.4242_4243del	p.(His1414GlnfsTer5)	Frameshift	Likely Pathogenic	M	54	Black - African
10245031	2953	RP1	NM_006269.2	c.4242_4243del	p.(His1414GlnfsTer5)	Frameshift	Likely Pathogenic	M	54	Black - African
2347309	2954	RP1	NM_006269.2	c.2167G>T	p.(Gly723Ter)	Stopgain	Pathogenic	M	92	Unknown
10075386	2955	RP1	NM_006269.2	c.2029C>T	p.(Arg677Ter)	Stopgain	Pathogenic	F	75	White - British
8117787	2956	RP1	NM_006269.2	c.2035C>T	p.(Gln679Ter)	Stopgain	Pathogenic	M	45	Not stated
5052438	2957	RP1	NM_006269.2	c.2172_2185del	p.(Ile725ArgfsTer6)	Frameshift	Pathogenic	M	87	Not stated
2687775	2958	RP1	NM_006269.2	c.2029C>T	p.(Arg677Ter)	Stopgain	Pathogenic	M	87	Not stated
3754813	2959	RP1	NM_006269.2	c.2285_2289delTAAAT	p.(Leu762TyrfsTer17)	Frameshift	Pathogenic	F	74	Not stated
17601282	2959	RP1	NM_006269.2	c.2285_2289delTAAAT	p.(Leu762TyrfsTer17)	Frameshift	Pathogenic	F	62	Not stated
7321523	2959	RP1	NM_006269.2	c.2285_2289del	p.(Leu762TyrfsTer17)	Frameshift	Pathogenic	M	54	Not stated
3954159	2960	RP1	NM_006269.2	c.2206dup	p.(Thr736AsnfsTer4)	Frameshift	Pathogenic	M	62	White - British
4170417	2961	RP1	NM_006269.2	c.2172_2185del	p.(Ile725ArgfsTer6)	Frameshift	Pathogenic	M	69	Not stated
4111043	2962	RP1	NM_006269.2	c.2029C>T	p.(Arg677Ter)	Stopgain	Pathogenic	F	86	Not stated
4210835	2963	RP1	NM_006269.2	c.2613dup	p.(Arg872ThrfsTer2)	Frameshift	Pathogenic	F	58	White - British
4160477	2964	RP1	NM_006269.2	c.2596_2597del	p.(Leu866LysfsTer7)	Frameshift	Pathogenic	F	72	White - British
12860070	2965	RP1	NM_006269.2	c.2596_2597del	p.(Leu866LysfsTer7)	Frameshift	Pathogenic	F	59	White - Irish
4813626	2965	RP1	NM_006269.2	c.2596_2597del	p.(Leu866LysfsTer7)	Frameshift	Pathogenic	M	63	White - British
13757456	2965	RP1	NM_006269.2	c.2596_2597del	p.(Leu866LysfsTer7)	Frameshift	Pathogenic	M	65	Unknown
8132914	2966	RP1	NM_006269.2	c.2596_2597del	p.(Leu866LysfsTer7)	Frameshift	Pathogenic	M	81	White - British
5064100	2967	RP1	NM_006269.2	c.2613dup	p.(Arg872ThrfsTer2)	Frameshift	Pathogenic	F	78	Not stated
10020800	2968	RP1	NM_006269.2	c.2172_2185del	p.(Ile725ArgfsTer6)	Frameshift	Pathogenic	F	43	White - British
9571638	2968	RP1	NM_006269.2	c.2172_2185del	p.(Ile725ArgfsTer6)	Frameshift	Pathogenic	M	68	Not stated
10317523	2969	RP1	NM_006269.2	c.2596_2597del	p.(Leu866LysfsTer7)	Frameshift	Pathogenic	F	52	Not stated
9361253	2969	RP1	NM_006269.2	c.2596_2597del	p.(Leu866LysfsTer7)	Frameshift	Pathogenic	M	82	Not stated

8233511	2969	RP1	NM_006269.2	c.2596_2597del	p.(Leu866LysfsTer7)	Frameshift	Pathogenic	M	46	White - British
5519443	2970	RP1	NM_006269.2	c.2321del	p.(Leu774Ter)	Stopgain	Likely Pathogenic	M	86	White - British
1714320	2971	RP1	NM_006269.2	c.2591_2600del	p.(Ile864LysfsTer11)	Frameshift	Likely Pathogenic	M	88	Not stated
5785954	2972	RP1	NM_006269.2	c.2083del	p.(Ile695PhefsTer8)	Frameshift	Likely Pathogenic	F	55	White - British
6248465	2973	RP1	NM_006269.2	c.2656C>T	p.(Gln886Ter)	Stopgain	Pathogenic	M	69	White - British
6369530	2974	RP1	NM_006269.2	c.2596_2597del	p.(Leu866LysfsTer7)	Frameshift	Pathogenic	M	105	Not stated
6047327	2975	RP1	NM_006269.2	c.312_315del	p.(Leu105ValfsTer10)	Frameshift	Pathogenic	F	29	Not stated
6047327	2975	RP1	NM_006269.2	c.3560_4860del	p.(Val1187GlufsTer4)	Frameshift	Likely Pathogenic	F	29	Not stated
6844249	2976	RP1	NM_006269.2	c.2596_2597del	p.(Leu866LysfsTer7)	Frameshift	Pathogenic	F	77	Any other ethnic group
6691621	2977	RP1	NM_006269.2	c.2596_2597del	p.(Leu866LysfsTer7)	Frameshift	Pathogenic	F	80	White - British
6699762	2978	RP1	NM_006269.2	c.2029C>T	p.(Arg677Ter)	Stopgain	Pathogenic	M	71	Any other ethnic group
6883610	2979	RP1	NM_006269.2	c.2596_2597del	p.(Leu866LysfsTer7)	Frameshift	Pathogenic	F	68	White - British
6266252	2980	RP1	NM_006269.2	c.2055T>A	p.(Tyr685Ter)	Stopgain	Likely Pathogenic	F	78	White - British
7177344	2981	RP1	NM_006269.2	c.2035C>T	p.(Gln679Ter)	Stopgain	Pathogenic	F	53	White - British
12743562	2982	RP1	NM_006269.2	c.2029C>T	p.(Arg677Ter)	Stopgain	Pathogenic	F	62	White - British
7294972	2983	RP1	NM_006269.2	c.2029C>T	p.(Arg677Ter)	Stopgain	Pathogenic	M	46	White - British
7338400	2984	RP1	NM_006269.2	c.2285_2289del	p.(Leu762TyrfsTer17)	Frameshift	Pathogenic	F	68	White - Other
7752828	2985	RP1	NM_006269.2	c.2029C>T	p.(Arg677Ter)	Stopgain	Pathogenic	M	61	White - British
6649047	2986	RP1	NM_006269.2	c.2613dup	p.(Arg872ThrfsTer2)	Frameshift	Pathogenic	M	65	Any other ethnic group
4996095	2987	RP1	NM_006269.2	c.4242_4243del	p.(His1414GlnfsTer5)	Frameshift	Likely Pathogenic	F	53	Black - African
4996095	2987	RP1	NM_006269.2	c.4242_4243del	p.(His1414GlnfsTer5)	Frameshift	Likely Pathogenic	F	53	Black - African
3986261	2988	RP1	NM_006269.2	c.2055T>A	p.(Tyr685Ter)	Stopgain	Likely Pathogenic	F	59	White - British
17421739	2988	RP1	NM_006269.2	c.2055T>A	p.(Tyr685Ter)	Stopgain	Likely Pathogenic	F	61	Not stated
9126102	2989	RP1	NM_006269.2	c.2029C>T	p.(Arg677Ter)	Stopgain	Pathogenic	M	51	White - British
9202948	2990	RP1	NM_006269.2	c.1625C>G	p.(Ser542Ter)	Stopgain	Pathogenic	F	37	Not stated
9202948	2990	RP1	NM_006269.2	c.1625C>G	p.(Ser542Ter)	Stopgain	Pathogenic	F	37	Not stated
9166415	2991	RP1	NM_006269.2	c.2172_2185del	p.(Ile725ArgfsTer6)	Frameshift	Pathogenic	F	62	Unknown
8858401	2992	RP1	NM_006269.2	c.2172_2185del	p.(Ile725ArgfsTer6)	Frameshift	Pathogenic	F	68	White - British
10073741	2993	RP1	NM_006269.2	c.2613dup	p.(Arg872ThrfsTer2)	Frameshift	Pathogenic	M	63	White - British
9945704	2994	RP1	NM_006269.2	c.2172_2185del	p.(Ile725ArgfsTer6)	Frameshift	Pathogenic	M	49	Unknown
8530094	2995	RP1	NM_006269.2	c.3843del	p.(Pro1282LeufsTer12)	Frameshift	Pathogenic	F	36	White - Other
8530094	2995	RP1	NM_006269.2	c.3843del	p.(Pro1282LeufsTer12)	Frameshift	Pathogenic	F	36	White - Other
10349688	2996	RP1	NM_006269.2	c.2029C>T	p.(Arg677Ter)	Stopgain	Pathogenic	M	57	Not stated
10326287	2997	RP1	NM_006269.2	c.4576G>T	p.(Glu1526Ter)	Stopgain	Likely Pathogenic	M	55	Not stated
10326287	2997	RP1	NM_006269.2	c.4576G>T	p.(Glu1526Ter)	Stopgain	Likely Pathogenic	M	55	Not stated
10524226	2998	RP1	NM_006269.2	c.2035C>T	p.(Gln679Ter)	Stopgain	Pathogenic	F	59	Unknown
10506950	2999	RP1	NM_006269.2	c.2115del	p.(Gly706ValfsTer7)	Frameshift	Likely Pathogenic	M	72	Any other ethnic group
5032866	3000	RP1	NM_006269.2	c.2029C>T	p.(Arg677Ter)	Stopgain	Pathogenic	M	54	Not stated
6550333	3001	RP1	NM_006269.2	c.2143C>T	p.(Gln715Ter)	Stopgain	Likely Pathogenic	F	90	White - British
9910984	3002	RP1	NM_006269.2	c.2032C>T	p.(Gln678Ter)	Stopgain	Pathogenic	M	47	White - British
10996551	3003	RP1	NM_006269.2	c.2206dup	p.(Thr736AsnfsTer4)	Frameshift	Pathogenic	F	54	White - British
16873681	3003	RP1	NM_006269.2	c.2613dup	p.(Arg872ThrfsTer2)	Frameshift	Pathogenic	F	57	White - British
11164194	3004	RP1	NM_006269.2	c.2029C>T	p.(Arg677Ter)	Stopgain	Pathogenic	M	72	White - British
11460819	3005	RP1	NM_006269.2	c.2029C>T	p.(Arg677Ter)	Stopgain	Pathogenic	M	54	Not stated
11251694	3006	RP1	NM_006269.2	c.2029C>T	p.(Arg677Ter)	Stopgain	Pathogenic	F	55	Not stated
11972498	3007	RP1	NM_006269.2	c.2029C>T	p.(Arg677Ter)	Stopgain	Pathogenic	M	50	White - Other
11778759	3008	RP1	NM_006269.2	c.2348dup	p.(Asn783LysfsTer2)	Frameshift	Pathogenic	F	38	White - British
3248251	3009	RP1	NM_006269.2	c.3843del	p.(Pro1282LeufsTer12)	Frameshift	Pathogenic	F	60	White - Other
3248251	3009	RP1	NM_006269.2	c.5883del	p.(Thr1962GlnfsTer15)	Frameshift	Likely Pathogenic	F	60	White - Other
9577952	3010	RP1	NM_006269.2	c.2281A>T	p.(Lys761Ter)	Stopgain	Likely Pathogenic	M	60	Not stated
12665736	3011	RP1	NM_006269.2	c.2732C>A	p.(Ser911Ter)	Stopgain	Likely Pathogenic	F	61	White - British

5704208	3012	RP1	NM_006269.2	c.5883del	p.(Thr1962GlnfsTer15)	Frameshift	Likely Pathogenic	M	29	Not stated
5704208	3012	RP1	NM_006269.2	c.5883del	p.(Thr1962GlnfsTer15)	Frameshift	Likely Pathogenic	M	29	Not stated
10460953	3013	RP1	NM_006269.2	c.2167G>T	p.(Gly723Ter)	Stopgain	Pathogenic	F	55	White - British
11061623	3014	RP1	NM_006269.2	c.2098G>T	p.(Glu700Ter)	Stopgain	Likely Pathogenic	F	66	Unknown
13016625	3015	RP1	NM_006269.2	c.2749C>T	p.(Gln917Ter)	Stopgain	Pathogenic	F	55	Unknown
13205247	3016	RP1	NM_006269.2	c.2098G>T	p.(Glu700Ter)	Stopgain	Likely Pathogenic	M	69	White - British
6784315	3017	RP1	NM_006269.2	c.2219C>G	p.(Ser740Ter)	Stopgain	Pathogenic	M	53	Not stated
13561225	3018	RP1	NM_006269.2	c.2029C>T	p.(Arg667Ter)	Stopgain	Pathogenic	F	35	Not stated
13484218	3019	RP1	NM_006269.2	c.2056C>T	p.(Gln686Ter)	Stopgain	Pathogenic	M	78	Unknown
13770973	3020	RP1	NM_006269.2	c.2596_2597del	p.(Leu866LysfsTer7)	Frameshift	Pathogenic	F	61	Unknown
10516190	3021	RP1	NM_006269.2	c.2172_2185del	p.(Ile725ArgfsTer6)	Frameshift	Pathogenic	M	43	White - British
13514584	3022	RP1	NM_006269.2	c.2029C>T	p.(Arg677Ter)	Stopgain	Pathogenic	M	40	White - British
13906423	3023	RP1	NM_006269.2	c.2029C>T	p.(Arg677Ter)	Stopgain	Pathogenic	F	41	Any other ethnic group
9121538	3023	RP1	NM_006269.2	c.2029C>T	p.(Arg677Ter)	Stopgain	Pathogenic	F	43	Not stated
13386603	3024	RP1	NM_006269.2	c.2596_2597del	p.(Leu866LysfsTer7)	Frameshift	Pathogenic	M	67	White - British
1771811	3025	RP1	NM_006269.2	c.2285_2289del	p.(Leu762TyrfsTer17)	Frameshift	Pathogenic	F	66	White - British
14947169	3026	RP1	NM_006269.2	c.2591_2600del	p.(Ile864LysfsTer11)	Frameshift	Likely Pathogenic	M	46	Not stated
14880263	3027	RP1	NM_006269.2	c.2596_2597del	p.(Leu866LysfsTer7)	Frameshift	Pathogenic	F	76	Not stated
14934891	3028	RP1	NM_006269.2	c.4147_4151del	p.(Gly138Ter)	Stopgain	Likely Pathogenic	M	19	Asian - Pakistani
14934891	3028	RP1	NM_006269.2	c.4147_4151del	p.(Gly138Ter)	Stopgain	Likely Pathogenic	M	19	Asian - Pakistani
10302781	3028	RP1	NM_006269.2	c.4147_4151del	p.(Gly138Ter)	Stopgain	Likely Pathogenic	F	21	Asian - Indian
10302781	3028	RP1	NM_006269.2	c.4147_4151del	p.(Gly138Ter)	Stopgain	Likely Pathogenic	F	21	Asian - Indian
14984584	3029	RP1	NM_006269.2	c.2648T>G	p.(Leu883Ter)	Stopgain	Likely Pathogenic	M	68	White - British
3822398	3030	RP1	NM_006269.2	c.2029C>T	p.(Arg677Ter)	Stopgain	Pathogenic	F	82	Unknown
1955253	3031	RP1	NM_006269.2	c.2585C>G	p.(Ser862Ter)	Stopgain	Likely Pathogenic	F	86	Asian - Indian
15218139	3032	RP1	NM_006269.2	c.2029C>T	p.(Arg677Ter)	Stopgain	Pathogenic	M	48	Not stated
10850370	3033	RP1	NM_006269.2	c.1793dup	p.(Tyr598Ter)	Stopgain	Likely Pathogenic	M	37	White - British
14823955	3034	RP1	NM_006269.2	c.2029C>T	p.(Arg677Ter)	Stopgain	Pathogenic	F	56	Asian - Other
15341409	3035	RP1	NM_006269.2	c.2780del	p.(Pro927GlnfsTer3)	Frameshift	Pathogenic	F	51	White - British
15500281	3036	RP1	NM_006269.2	c.606C>A	p.(Asp202Glu)	Missense	Variant of Uncertain Significance	F	57	Any other ethnic group
15500281	3036	RP1	NM_006269.2	c.606C>A	p.(Asp202Glu)	Missense	Variant of Uncertain Significance	F	57	Any other ethnic group
15773876	3037	RP1	NM_006269.2	c.2172_2185del	p.(Ile725ArgfsTer6)	Frameshift	Pathogenic	M	69	Any other ethnic group
16026730	3038	RP1	NM_006269.2	c.668del	p.(Gly223GlufsTer41)	Frameshift	Pathogenic	M	42	White - British
16026730	3038	RP1	NM_006269.2	c.668del	p.(Gly223GlufsTer41)	Frameshift	Pathogenic	M	42	White - British
18060062	3038	RP1	NM_006269.2	c.668del	p.(Gly223GlufsTer41)	Frameshift	Pathogenic	M	39	Not stated
18060062	3038	RP1	NM_006269.2	c.668del	p.(Gly223GlufsTer41)	Frameshift	Pathogenic	M	39	Not stated
15042768	3039	RP1	NM_006269.2	c.2596_2597del	p.(Leu866LysfsTer7)	Frameshift	Pathogenic	M	46	White - British
12501397	3039	RP1	NM_006269.2	c.2596_2597del	p.(Leu866LysfsTer7)	Frameshift	Pathogenic	M	71	White - British
10485768	3040	RP1	NM_006269.2	c.2613dup	p.(Arg872ThrfsTer2)	Frameshift	Pathogenic	M	55	White - British
16539172	3041	RP1	NM_006269.2	c.2014_2015dup	p.(Lys673ArgfsTer10)	Frameshift	Likely Pathogenic	M	44	White - British
8156042	3042	RP1	NM_006269.2	c.2801C>G	p.(Ser934Ter)	Stopgain	Likely Pathogenic	M	60	Not stated
14826881	3043	RP1	NM_006269.2	c.2585C>G	p.(Ser862Ter)	Stopgain	Likely Pathogenic	F	57	Black - Caribbean
16939495	3044	RP1	NM_006269.2	c.2029C>T	p.(Arg677Ter)	Stopgain	Pathogenic	M	50	White - British
15846340	3045	RP1	NM_006269.2	c.2029C>T	p.(Arg677Ter)	Stopgain	Pathogenic	F	60	White - British
17720660	3046	RP1	NM_006269.2	c.2596_2597del	p.(Leu866LysfsTer7)	Frameshift	Pathogenic	M	67	Not stated
17002264	3047	RP1	NM_006269.2	c.2780del	p.(Pro927GlnfsTer3)	Frameshift	Pathogenic	F	56	Not stated
17836573	3048	RP1	NM_006269.2	c.2564dup	p.(Leu855PhefsTer7)	Frameshift	Likely Pathogenic	F	71	White - British
17991861	3049	RP1	NM_006269.2	c.2348dup	p.(Asn783LysfsTer2)	Frameshift	Pathogenic	F	55	Not stated
18090400	3050	RP1	NM_006269.2	c.2029C>T	p.(Arg677Ter)	Stopgain	Pathogenic	F	31	Not stated
18425238	3051	RP1	NM_006269.2	c.2025_2026dup	p.(Ser676TyrfsTer7)	Frameshift	Likely Pathogenic	F	47	Not stated
6361123	3052	RP1L1	NM_178857.6	c.3596C>T	p.(Ser1199Phe)	Missense	Variant of Uncertain Significance	M	48	Any other ethnic group

12643042	3052	RP1L1	NM_178857.6	c.3596C>T	p.(Ser1199Phe)	Missense	Variant of Uncertain Significance	M	13	Not stated
10559331	3053	RP1L1	NM_178857.6	c.133C>T	p.(Arg45Trp)	Missense	Likely Pathogenic	M	42	White - British
10559331	3053	RP1L1	NM_178857.6	c.449C>T	p.(Thr150Ile)	Missense	Variant of Uncertain Significance	M	42	White - British
10667978	3054	RP1L1	NM_178857.6	c.3773T>C	p.(Phe1258Ser)	Missense	Variant of Uncertain Significance	F	64	Not stated
8606450	3055	RP1L1	NM_178857.6	c.3599G>C	p.(Gly1200Ala)	Missense	Variant of Uncertain Significance	F	63	Not stated
5374473	3056	RP1L1	NM_178857.6	c.2849G>A	p.(Arg950His)	Missense	Variant of Uncertain Significance	M	71	Not stated
10245696	3057	RP1L1	NM_178857.6	c.329C>T	p.(Pro110Leu)	Missense	Variant of Uncertain Significance	F	89	Any other ethnic group
4742765	3058	RP1L1	NM_178857.6	c.2873G>A	p.(Arg958His)	Missense	Variant of Uncertain Significance	M	58	Not stated
7371811	3059	RP1L1	NM_178857.6	c.603del	p.(Lys203ArgfsTer28)	Frameshift	Pathogenic	M	84	Asian - Pakistani
7371811	3059	RP1L1	NM_178857.6	c.603del	p.(Lys203ArgfsTer28)	Frameshift	Pathogenic	M	84	Asian - Pakistani
10580359	3060	RP1L1	NM_178857.6	c.133C>T	p.(Arg45Trp)	Missense	Likely Pathogenic	M	85	White - British
4978105	3061	RP1L1	NM_178857.6	c.133C>T	p.(Arg45Trp)	Missense	Likely Pathogenic	F	42	Not stated
10280976	3062	RP1L1	NM_178857.6	c.133C>T	p.(Arg45Trp)	Missense	Likely Pathogenic	M	46	Not stated
11701941	3063	RP1L1	NM_178857.6	c.3599G>C	p.(Gly1200Ala)	Missense	Variant of Uncertain Significance	F	42	Not stated
11668334	3064	RP1L1	NM_178857.6	c.1215T>G	p.(Tyr405Ter)	Stopgain	Likely Pathogenic	F	60	White - British
11668334	3064	RP1L1	NM_178857.6	c.1215T>G	p.(Tyr405Ter)	Stopgain	Likely Pathogenic	F	60	White - British
15795912	3065	RP1L1	NM_178857.6	c.133C>T	p.(Arg45Trp)	Missense	Likely Pathogenic	F	26	Black - Caribbean
15934652	3066	RP1L1	NM_178857.6	c.3629C>T	p.(Ser1210Leu)	Missense	Variant of Uncertain Significance	M	81	Not stated
16489514	3067	RP1L1	NM_178857.6	c.133C>T	p.(Arg45Trp)	Missense	Likely Pathogenic	F	29	White - British
4813269	3068	RP2	NM_006915.3	c.460G>T	p.(Glu154Ter)	Stopgain	Likely Pathogenic	M	41	White - British
5330639	3068	RP2	NM_006915.3	c.460G>T	p.(Glu154Ter)	Stopgain	Likely Pathogenic	M	32	Not stated
13675472	3069	RP2	NM_006915.3	c.568_569delinsG	p.(Pro190GlufsTer48)	Frameshift	Likely Pathogenic	M	33	Not stated
1161173	3069	RP2	NM_006915.3	c.568_569delinsG	p.(Pro190GlufsTer48)	Frameshift	Likely Pathogenic	M	61	Black - Caribbean
782711	3070	RP2	NM_006915.3	c.884-1G>T	Splice	Splice	Likely Pathogenic	M	57	White - British
18042478	3071	RP2	NM_006915.3	c.258T>A	p.(Cys86Ter)	Stopgain	Likely Pathogenic	M	66	Not stated
11706274	3072	RP2	NM_006915.3	c.929dup	p.(Cys311MetfsTer18)	Frameshift	Likely Pathogenic	M	80	Unknown
4853316	3073	RP2	NM_006915.3	Exon 5 deletion	Deletion	Deletion	Variant of Uncertain Significance	M	39	Asian - Other
5441393	3074	RP2	NM_006915.3	c.235del	p.(Ala79LeufsTer12)	Frameshift	Likely Pathogenic	M	36	White - British
6060620	3075	RP2	NM_006915.3	c.798_801del	p.(Thr267ArgfsTer5)	Frameshift	Likely Pathogenic	M	67	White - British
6549885	3076	RP2	NM_006915.3	c.685_691del	p.(Gln229AlafsTer7)	Frameshift	Likely Pathogenic	M	34	Black - Caribbean
5387801	3076	RP2	NM_006915.3	c.685_691del	p.(Gln229AlafsTer7)	Frameshift	Likely Pathogenic	M	53	Black - Other
5133323	3077	RP2	NM_006915.3	c.43del	p.(Ser15ArgfsTer31)	Frameshift	Pathogenic	M	29	Not stated
5133323	3077	RP2	NM_006915.3	c.43del	p.(Ser15ArgfsTer31)	Frameshift	Pathogenic	M	29	Not stated
6917077	3078	RP2	NM_006915.3	c.341G>A	p.(Cys114Tyr)	Missense	Likely Pathogenic	F	55	White - British
7264480	3079	RP2	NM_006915.3	c.969+3A>T	Splice	Splice	Variant of Uncertain Significance	M	29	White - British
9547558	3079	RP2	NM_006915.3	c.969+3A>T	Splice	Splice	Variant of Uncertain Significance	M	28	Not stated
9207701	3080	RP2	NM_006915.3	c.19A>T	p.(Lys7Ter)	Stopgain	Likely Pathogenic	M	30	White - British
9510283	3081	RP2	NM_006915.3	c.768+1G>A	Splice	Splice	Likely Pathogenic	M	31	White - British
9079314	3082	RP2	NM_006915.3	c.358C>T	p.(Arg120Ter)	Stopgain	Pathogenic	M	27	White - British
10355442	3083	RP2	NM_006915.3	c.460G>T	p.(Glu154Ter)	Stopgain	Likely Pathogenic	M	30	Not stated
5793703	3084	RP2	NM_006915.3	c.352C>T	p.(Arg118Cys)	Missense	Pathogenic	M	33	White - British
10428844	3085	RP2	NM_006915.3	c.128_140del	p.(Ser43MetfsTer4)	Frameshift	Likely Pathogenic	M	53	Any other ethnic group
11218871	3086	RP2	NM_006915.3	Exon 2 deletion	Deletion	Deletion	Variant of Uncertain Significance	M	32	Unknown
3665227	3087	RP2	NM_006915.3	c.14_16del	p.(Phe5del)	Inframe indel	Pathogenic	M	59	White - British
16353028	3087	RP2	NM_006915.3	c.14_16del	p.(Phe5del)	Inframe indel	Pathogenic	M	26	Not stated
13413812	3088	RP2	NM_006915.3	c.352C>T	p.(Arg118Cys)	Missense	Pathogenic	M	42	Unknown
13871535	3089	RP2	NM_006915.3	Exon 1-5 deletion	Deletion	Deletion	Likely Pathogenic	M	15	Not stated
15284555	3089	RP2	NM_006915.3	Exon 1-5 deletion	Deletion	Deletion	Likely Pathogenic	M	13	Not stated
15123548	3090	RP2	NM_006915.3	c.852del	p.(Ala285HisfsTer8)	Frameshift	Likely Pathogenic	F	47	Not stated
9042648	3091	RP2	NM_006915.3	Exon 5 deletion	Deletion	Deletion	Variant of Uncertain Significance	M	32	Not stated
17066762	3092	RP2	NM_006915.3	c.358C>T	p.(Arg120Ter)	Stopgain	Pathogenic	M	16	Not stated

17227090	3093	RP2	NM_006915.3	c.159_160insAA	p.(Pro54AsnfsTer5)	Frameshift	Likely Pathogenic	M	25	Not stated
17756472	3094	RP2	NM_006915.3	c.257G>A	p.(Cys86Tyr)	Missense	Likely Pathogenic	M	18	Not stated
17424168	3095	RP2	NM_006915.3	c.181C>T	p.(Gln61Ter)	Stopgain	Likely Pathogenic	M	12	Not stated
18214482	3096	RP2	NM_006915.3	c.235del	p.(Ala79LeufsTer12)	Frameshift	Likely Pathogenic	M	19	Not stated
18201035	3097	RP2	NM_006915.3	c.450G>A	p.(Trp150Ter)	Stopgain	Pathogenic	M	33	Not stated
2114573	3098	RP9	NM_203288.2	c.410A>T	p.(His137Leu)	Missense	Variant of Uncertain Significance	F	58	White - British
9328353	3098	RP9	NM_203288.2	c.410A>T	p.(His137Leu)	Missense	Variant of Uncertain Significance	F	33	Any other ethnic group
11302073	3098	RP9	NM_203288.2	c.410A>T	p.(His137Leu)	Missense	Variant of Uncertain Significance	M	35	Not stated
164464	3098	RP9	NM_203288.2	c.410A>T	p.(His137Leu)	Missense	Variant of Uncertain Significance	M	57	White - British
13478800	3098	RP9	NM_203288.2	c.410A>T	p.(His137Leu)	Missense	Variant of Uncertain Significance	F	32	Not stated
13907739	3098	RP9	NM_203288.2	c.410A>T	p.(His137Leu)	Missense	Variant of Uncertain Significance	M	51	Unknown
9992541	3099	RP9	NM_203288.2	c.410A>T	p.(His137Leu)	Missense	Variant of Uncertain Significance	M	63	Not stated
8593052	3100	RP9	NM_203288.2	c.410A>T	p.(His137Leu)	Missense	Variant of Uncertain Significance	M	74	White - British
6767067	3101	RP9	NM_203288.2	c.410A>T	p.(His137Leu)	Missense	Variant of Uncertain Significance	M	77	White - British
7258761	3101	RP9	NM_203288.2	c.410A>T	p.(His137Leu)	Missense	Variant of Uncertain Significance	M	54	White - British
12484492	3102	RP9	NM_203288.2	c.410A>T	p.(His137Leu)	Missense	Variant of Uncertain Significance	M	51	Not stated
13765576	3102	RP9	NM_203288.2	c.410A>T	p.(His137Leu)	Missense	Variant of Uncertain Significance	F	74	Unknown
15929430	3103	RP9	NM_203288.2	c.410A>T	p.(His137Leu)	Missense	Variant of Uncertain Significance	M	33	White - Other
18268578	3104	RP9	NM_203288.2	c.410A>T	p.(His137Leu)	Missense	Variant of Uncertain Significance	F	21	Unknown
18439378	3105	RP9	NM_203288.2	c.410A>T	p.(His137Leu)	Missense	Variant of Uncertain Significance	F	32	Unknown
605485	3106	RPE65	NM_000329.3	c.11+5G>A	Splice	Splice	Pathogenic	M	52	White - British
605485	3106	RPE65	NM_000329.3	c.1543C>T	p.(Arg515Trp)	Missense	Pathogenic	M	52	White - British
3701998	3107	RPE65	NM_000329.3	c.118G>A	p.(Gly40Ser)	Missense	Pathogenic	F	32	Not stated
3701998	3107	RPE65	NM_000329.3	c.118G>A	p.(Gly40Ser)	Missense	Pathogenic	F	32	Not stated
6470050	3108	RPE65	NM_000329.3	c.1543C>T	p.(Arg515Trp)	Missense	Pathogenic	F	55	Any other ethnic group
6470050	3108	RPE65	NM_000329.3	c.1079C>T	p.(Ala360Val)	Missense	Likely Pathogenic	F	55	Any other ethnic group
12384637	3109	RPE65	NM_000329.3	c.1430A>G	p.(Asp477Gly)	Missense	Pathogenic	M	57	White - British
637580	3109	RPE65	NM_000329.3	c.1430A>G	p.(Asp477Gly)	Missense	Pathogenic	M	82	Not stated
9631992	3110	RPE65	NM_000329.3	c.179T>C	p.(Leu60Pro)	Missense	Likely Pathogenic	F	48	Asian - Pakistani
9631992	3110	RPE65	NM_000329.3	c.179T>C	p.(Leu60Pro)	Missense	Likely Pathogenic	F	48	Asian - Pakistani
850296	3110	RPE65	NM_000329.3	c.179T>C	p.(Leu60Pro)	Missense	Likely Pathogenic	M	41	Asian - Pakistani
850296	3110	RPE65	NM_000329.3	c.179T>C	p.(Leu60Pro)	Missense	Likely Pathogenic	M	41	Asian - Pakistani
5569584	3110	RPE65	NM_000329.3	c.179T>C	p.(Leu60Pro)	Missense	Likely Pathogenic	M	47	Asian - Indian
5569584	3110	RPE65	NM_000329.3	c.179T>C	p.(Leu60Pro)	Missense	Likely Pathogenic	M	47	Asian - Indian
4687990	3110	RPE65	NM_000329.3	c.179T>C	p.(Leu60Pro)	Missense	Likely Pathogenic	M	49	White - British
4687990	3110	RPE65	NM_000329.3	c.179T>C	p.(Leu60Pro)	Missense	Likely Pathogenic	M	49	White - British
6997682	3111	RPE65	NM_000329.3	c.118G>A	p.(Gly40Ser)	Missense	Pathogenic	F	34	White - British
6997682	3111	RPE65	NM_000329.3	c.11+5G>A	Splice	Splice	Pathogenic	F	34	White - British
6092981	3112	RPE65	NM_000329.3	c.1067dup	p.(Asn356LysfsTer9)	Frameshift	Pathogenic	M	32	White - British
6092981	3112	RPE65	NM_000329.3	c.1543C>T	p.(Arg515Trp)	Missense	Pathogenic	M	32	White - British
7127924	3113	RPE65	NM_000329.3	c.271C>T	p.(Arg91Trp)	Missense	Pathogenic	M	24	Black - African
7127924	3113	RPE65	NM_000329.3	c.271C>T	p.(Arg91Trp)	Missense	Pathogenic	M	24	Black - African
858381	3114	RPE65	NM_000329.3	c.179T>C	p.(Leu60Pro)	Missense	Likely Pathogenic	M	63	Asian - Other
858381	3114	RPE65	NM_000329.3	c.179T>C	p.(Leu60Pro)	Missense	Likely Pathogenic	M	63	Asian - Other
4847016	3115	RPE65	NM_000329.3	c.886dup	p.(Arg296LysfsTer7)	Frameshift	Pathogenic	F	37	Not stated
4847016	3115	RPE65	NM_000329.3	c.433G>A	p.(Ala145Thr)	Missense	Likely Pathogenic	F	37	Not stated
6245147	3116	RPE65	NM_000329.3	c.1430A>G	p.(Asp477Gly)	Missense	Pathogenic	M	57	White - British
7784650	3117	RPE65	NM_000329.3	c.746A>G	p.(Tyr249Cys)	Missense	Pathogenic	M	32	Asian - Other
7784650	3117	RPE65	NM_000329.3	c.746A>G	p.(Tyr249Cys)	Missense	Pathogenic	M	32	Asian - Other
8852269	3118	RPE65	NM_000329.3	c.1102T>C	p.(Tyr368His)	Missense	Pathogenic	M	20	Not stated
8852269	3118	RPE65	NM_000329.3	c.271C>T	p.(Arg91Trp)	Missense	Pathogenic	M	20	Not stated

10034828	3119	RPE65	NM_000329.3	c.11+5G>A	Splice	Splice	Pathogenic	M	27	Not stated
10034828	3119	RPE65	NM_000329.3	c.1102T>C	p.(Tyr368His)	Missense	Pathogenic	M	27	Not stated
8993837	3120	RPE65	NM_000329.3	c.1067dup	p.(Asn356LysfsTer9)	Frameshift	Pathogenic	M	18	Asian - Bangladeshi
8993837	3120	RPE65	NM_000329.3	c.1067dup	p.(Asn356LysfsTer9)	Frameshift	Pathogenic	M	18	Asian - Bangladeshi
8753604	3121	RPE65	NM_000329.3	c.852del	p.(Met285TrpfsTer40)	Frameshift	Likely Pathogenic	M	23	Not stated
8753604	3121	RPE65	NM_000329.3	c.952T>A	p.(Tyr318Asn)	Missense	Likely Pathogenic	M	23	Not stated
9981663	3122	RPE65	NM_000329.3	c.1306G>A	p.(Gly436Arg)	Missense	Pathogenic	F	24	Black - African
9981663	3122	RPE65	NM_000329.3	c.272G>A	p.(Arg91Gln)	Missense	Likely Pathogenic	F	24	Black - African
10177152	3123	RPE65	NM_000329.3	c.1102T>C	p.(Tyr368His)	Missense	Pathogenic	M	20	White - British
10177152	3123	RPE65	NM_000329.3	c.130C>T	p.(Arg44Ter)	Stopgain	Pathogenic	M	20	White - British
9637375	3124	RPE65	NM_000329.3	c.1102T>C	p.(Tyr368His)	Missense	Pathogenic	F	18	Not stated
9637375	3124	RPE65	NM_000329.3	c.461del	p.(Lys154ArgfsTer54)	Frameshift	Likely Pathogenic	F	18	Not stated
10102476	3125	RPE65	NM_000329.3	c.353G>A	p.(Arg118Lys)	Missense	Variant of Uncertain Significance	F	19	Asian - Other
10102476	3125	RPE65	NM_000329.3	c.353G>A	p.(Arg118Lys)	Missense	Variant of Uncertain Significance	F	19	Asian - Other
10885951	3126	RPE65	NM_000329.3	c.1087C>A	p.(Pro363Thr)	Missense	Pathogenic	M	18	Unknown
10885951	3126	RPE65	NM_000329.3	c.1418T>A	p.(Val473Asp)	Missense	Pathogenic	M	18	Unknown
11500124	3127	RPE65	NM_000329.3	c.1024T>C	p.(Tyr342His)	Missense	Variant of Uncertain Significance	F	15	Not stated
11500124	3127	RPE65	NM_000329.3	c.131G>A	p.(Arg44Gln)	Missense	Pathogenic	F	15	Not stated
11872293	3128	RPE65	NM_000329.3	c.11+5G>A	Splice	Splice	Pathogenic	M	24	White - British
11872293	3128	RPE65	NM_000329.3	c.245G>A	p.(Arg82Lys)	Missense	Variant of Uncertain Significance	M	24	White - British
12041630	3129	RPE65	NM_000329.3	c.118G>A	p.(Gly40Ser)	Missense	Pathogenic	F	29	Not stated
12041630	3129	RPE65	NM_000329.3	c.955G>A	p.(Glu319Lys)	Missense	Likely Pathogenic	F	29	Not stated
12704579	3130	RPE65	NM_000329.3	c.304G>T	p.(Glu102Ter)	Stopgain	Pathogenic	M	61	Unknown
12704579	3130	RPE65	NM_000329.3	c.272G>A	p.(Arg91Gln)	Missense	Likely Pathogenic	M	61	Unknown
12892095	3131	RPE65	NM_000329.3	c.11+5G>A	Splice	Splice	Pathogenic	F	17	Not stated
12892095	3131	RPE65	NM_000329.3	c.74C>T	p.(Pro25Leu)	Missense	Pathogenic	F	17	Not stated
13041839	3132	RPE65	NM_000329.3	c.1451G>A	p.(Gly484Asp)	Missense	Pathogenic	M	27	Not stated
13041839	3132	RPE65	NM_000329.3	c.1451G>A	p.(Gly484Asp)	Missense	Pathogenic	M	27	Not stated
13721175	3133	RPE65	NM_000329.3	c.304G>T	p.(Glu102Ter)	Stopgain	Pathogenic	F	11	Not stated
13721175	3133	RPE65	NM_000329.3	c.304G>T	p.(Glu102Ter)	Stopgain	Pathogenic	F	11	Not stated
14763034	3134	RPE65	NM_000329.3	c.1443_1445del	p.(Glu481del)	Inframe indel	Variant of Uncertain Significance	F	28	Any other ethnic group
14763034	3134	RPE65	NM_000329.3	c.989G>A	p.(Cys330Tyr)	Missense	Likely Pathogenic	F	28	Any other ethnic group
15777600	3135	RPE65	NM_000329.3	c.11+5G>A	Splice	Splice	Pathogenic	F	27	Not stated
15777600	3135	RPE65	NM_000329.3	c.1341_1342dup	p.(Cys448SerfsTer4)	Frameshift	Likely Pathogenic	F	27	Not stated
15824430	3136	RPE65	NM_000329.3	c.859G>T	p.(Val287Phe)	Missense	Likely Pathogenic	M	25	Not stated
15824430	3136	RPE65	NM_000329.3	c.859G>T	p.(Val287Phe)	Missense	Likely Pathogenic	M	25	Not stated
15779938	3137	RPE65	NM_000329.3	c.1398C>G	p.(Tyr466Ter)	Stopgain	Likely Pathogenic	M	27	Not stated
15779938	3137	RPE65	NM_000329.3	c.1464T>A	p.(Ser488Arg)	Missense	Variant of Uncertain Significance	M	27	Not stated
16168116	666	RPE65	NM_000329.3	c.304G>T	p.(Glu102Ter)	Stopgain	Pathogenic	M	10	Not stated
16168116	666	RPE65	NM_000329.3	c.304G>T	p.(Glu102Ter)	Stopgain	Pathogenic	M	10	Not stated
16146626	3138	RPE65	NM_000329.3	c.130C>T	p.(Arg44Ter)	Stopgain	Pathogenic	M	28	Not stated
16146626	3138	RPE65	NM_000329.3	c.1543C>T	p.(Arg515Trp)	Missense	Pathogenic	M	28	Not stated
16146640	3138	RPE65	NM_000329.3	c.130C>T	p.(Arg44Ter)	Stopgain	Pathogenic	M	30	Not stated
16146640	3138	RPE65	NM_000329.3	c.1543C>T	p.(Arg515Trp)	Missense	Pathogenic	M	30	Not stated
15938929	3139	RPE65	NM_000329.3	c.370C>T	p.(Arg124Ter)	Stopgain	Pathogenic	M	24	Not stated
15938929	3139	RPE65	NM_000329.3	c.952T>A	p.(Tyr318Asn)	Missense	Likely Pathogenic	M	24	Not stated
16091606	3140	RPE65	NM_000329.3	c.825C>A	p.(Tyr275Ter)	Stopgain	Likely Pathogenic	M	13	Not stated
16091606	3140	RPE65	NM_000329.3	c.982C>T	p.(Leu328Phe)	Missense	Likely Pathogenic	M	13	Not stated
16925635	3141	RPE65	NM_000329.3	c.271C>T	p.(Arg91Trp)	Missense	Pathogenic	F	10	Not stated
16925635	3141	RPE65	NM_000329.3	c.271C>T	p.(Arg91Trp)	Missense	Pathogenic	F	10	Not stated
16925656	3141	RPE65	NM_000329.3	c.271C>T	p.(Arg91Trp)	Missense	Pathogenic	F	9	Not stated

16925656	3141	RPE65	NM_000329.3	c.271C>T	p.(Arg91Trp)	Missense	Pathogenic	F	9	Not stated
16910921	3142	RPE65	NM_000329.3	c.1292A>G	p.(Tyr431Cys)	Missense	Pathogenic	F	17	Not stated
16910921	3142	RPE65	NM_000329.3	c.47T>C	p.(Phe16Ser)	Missense	Variant of Uncertain Significance	F	17	Not stated
16672970	3143	RPE65	NM_000329.3	c.433G>A	p.(Ala145Thr)	Missense	Likely Pathogenic	M	45	Not stated
16672970	3143	RPE65	NM_000329.3	c.433G>A	p.(Ala145Thr)	Missense	Likely Pathogenic	M	45	Not stated
17026834	3144	RPE65	NM_000329.3	c.1451G>A	p.(Gly484Asp)	Missense	Pathogenic	M	11	Not stated
17026834	3144	RPE65	NM_000329.3	c.331C>T	p.(Pro111Ser)	Missense	Likely Pathogenic	M	11	Not stated
3909366	3145	RPE65	NM_000329.3	c.1334A>G	p.(Asp445Gly)	Missense	Likely Pathogenic	M	54	White - British
3909366	3145	RPE65	NM_000329.3	c.499G>T	p.(Asp167Tyr)	Missense	Likely Pathogenic	M	54	White - British
17056605	3146	RPE65	NM_000329.3	c.370C>T	p.(Arg124Ter)	Stopgain	Pathogenic	F	16	Not stated
17056605	3146	RPE65	NM_000329.3	Exon 1-14 deletion	Deletion	Deletion	Likely Pathogenic	F	16	Not stated
17143384	3147	RPE65	NM_000329.3	c.311G>T	p.(Gly104Val)	Missense	Pathogenic	F	14	Not stated
17143384	3147	RPE65	NM_000329.3	c.95-2A>T	Splice	Splice	Pathogenic	F	14	Not stated
15831122	3148	RPE65	NM_000329.3	c.859G>T	p.(Val287Phe)	Missense	Likely Pathogenic	M	11	Asian - Other
15831122	3148	RPE65	NM_000329.3	c.859G>T	p.(Val287Phe)	Missense	Likely Pathogenic	M	11	Asian - Other
17941076	3149	RPE65	NM_000329.3	c.1543C>T	p.(Arg515Trp)	Missense	Pathogenic	M	6	Not stated
17941076	3149	RPE65	NM_000329.3	c.644-5T>G	Splice	Splice	Variant of Uncertain Significance	M	6	Not stated
18094313	3150	RPE65	NM_000329.3	c.726-2A>C	Splice	Splice	Likely Pathogenic	M	10	Not stated
18094313	3150	RPE65	NM_000329.3	c.726-2A>C	Splice	Splice	Likely Pathogenic	M	10	Not stated
17993758	3151	RPE65	NM_000329.3	c.499G>T	p.(Asp167Tyr)	Missense	Likely Pathogenic	F	24	Not stated
17993758	3151	RPE65	NM_000329.3	c.499G>T	p.(Asp167Tyr)	Missense	Likely Pathogenic	F	24	Not stated
9095939	3152	RPE65	NM_000329.3	c.1430A>G	p.(Asp477Gly)	Missense	Pathogenic	F	47	White - British
7283156	3153	RPGR	NM_001034853.2	c.2426_2427del	p.(Glu809GlyfsTer25)	Frameshift	Pathogenic	F	42	White - British
17024650	3153	RPGR	NM_001034853.2	c.2426_2427del	p.(Glu809GlyfsTer25)	Frameshift	Pathogenic	M	28	Not stated
596574	3154	RPGR	NM_001034853.2	c.2405_2406del	p.(Glu802GlyfsTer32)	Frameshift	Pathogenic	M	42	White - British
5593545	3154	RPGR	NM_001034853.2	c.2405_2406del	p.(Glu802GlyfsTer32)	Frameshift	Pathogenic	F	54	White - British
9097038	3154	RPGR	NM_001034853.2	c.2405_2406del	p.(Glu802GlyfsTer32)	Frameshift	Pathogenic	M	21	White - British
7967448	3154	RPGR	NM_001034853.2	c.2405_2406del	p.(Glu802GlyfsTer32)	Frameshift	Pathogenic	M	23	Not stated
5116306	3154	RPGR	NM_001034853.2	c.2405_2406del	p.(Glu802GlyfsTer32)	Frameshift	Pathogenic	F	47	Not stated
4301541	3154	RPGR	NM_001034853.2	c.2405_2406del	p.(Glu802GlyfsTer32)	Frameshift	Pathogenic	M	36	Not stated
10876165	3155	RPGR	NM_001034853.2	c.2624_2643del	p.(Glu875GlyfsTer197)	Frameshift	Likely Pathogenic	F	85	Not stated
13142415	3155	RPGR	NM_001034853.2	c.2624_2643del	p.(Glu875GlyfsTer197)	Frameshift	Likely Pathogenic	M	25	Any other ethnic group
3989558	3156	RPGR	NM_001034853.2	c.891_892del	p.(Ile297MetfsTer48)	Frameshift	Likely Pathogenic	M	33	White - British
1181788	3157	RPGR	NM_001034853.2	c.295A>G	p.(Thr99Ala)	Missense	Variant of Uncertain Significance	M	77	Unknown
4854877	3158	RPGR	NM_001034853.2	c.2045_2046dup	p.(Arg683ValfsTer15)	Frameshift	Likely Pathogenic	M	35	White - British
12762616	3159	RPGR	NM_001034853.2	c.2384del	p.(Glu795GlyfsTer20)	Frameshift	Pathogenic	F	57	White - British
430898	3160	RPGR	NM_001034853.2	c.2426_2427del	p.(Glu809GlyfsTer25)	Frameshift	Pathogenic	M	70	Not stated
4064444	3161	RPGR	NM_001034853.2	c.470-73_619+1581del	Deletion	Deletion	Variant of Uncertain Significance	M	72	Not stated
6284830	3162	RPGR	NM_001034853.2	c.799G>A	p.(Gly267Arg)	Missense	Pathogenic	M	34	Not stated
7686580	3163	RPGR	NM_001034853.2	c.2426_2427del	p.(Glu809GlyfsTer25)	Frameshift	Pathogenic	M	25	Not stated
3121670	3164	RPGR	NM_001034853.2	c.581G>A	p.(Trp194Ter)	Stopgain	Pathogenic	M	34	White - British
6857367	3164	RPGR	NM_001034853.2	c.581G>A	p.(Trp194Ter)	Stopgain	Pathogenic	M	38	Not stated
878156	3164	RPGR	NM_001034853.2	c.581G>A	p.(Trp194Ter)	Stopgain	Pathogenic	M	54	White - British
4473461	3165	RPGR	NM_001034853.2	c.2245G>T	p.(Glu749Ter)	Stopgain	Likely Pathogenic	F	59	Not stated
6462644	3165	RPGR	NM_001034853.2	c.2245G>T	p.(Glu749Ter)	Stopgain	Likely Pathogenic	M	29	White - British
2431624	3165	RPGR	NM_001034853.2	c.2245G>T	p.(Glu749Ter)	Stopgain	Likely Pathogenic	F	81	White - British
8819530	3165	RPGR	NM_001034853.2	c.2245G>T	p.(Glu749Ter)	Stopgain	Likely Pathogenic	M	37	Not stated
10632607	3166	RPGR	NM_001034853.2	c.2557G>T	p.(Glu853Ter)	Stopgain	Likely Pathogenic	M	62	White - British
16178189	3167	RPGR	NM_001034853.2	c.442_469+8del	Splice	Splice	Likely Pathogenic	M	22	Not stated
3328891	3168	RPGR	NM_001034853.2	c.2384del	p.(Glu795GlyfsTer20)	Frameshift	Pathogenic	M	52	White - British
11661894	3169	RPGR	NM_001034853.2	Exon 7 deletion	Deletion	Deletion	Variant of Uncertain Significance	M	32	White - British

3413794	3170	RPGR	NM_001034853.2	c.2541_2542del	p.(Glu848GlyfsTer230)	Frameshift	Pathogenic	M	59	White - British
768739	3171	RPGR	NM_001034853.2	c.2405_2406del	p.(Glu802GlyfsTer32)	Frameshift	Pathogenic	M	66	Asian - Indian
10528860	3172	RPGR	NM_001034853.2	c.2236_2237del	p.(Glu746ArgfsTer23)	Frameshift	Pathogenic	M	83	White - British
3630262	3173	RPGR	NM_001034853.2	c.1243_1244dup	p.(Glu416GlyfsTer10)	Frameshift	Likely Pathogenic	M	59	White - British
3492866	3174	RPGR	NM_001034853.2	c.154G>A	p.(Gly52Arg)	Missense	Likely Pathogenic	M	67	Not stated
6344988	3175	RPGR	NM_001034853.2	c.2476_2477del	p.(Arg826GlyfsTer8)	Frameshift	Pathogenic	M	27	White - British
1428321	3176	RPGR	NM_001034853.2	c.2105_2213dup	p.(Glu742fsTer)	Frameshift	Variant of Uncertain Significance	M	42	Not stated
16116820	3177	RPGR	NM_001034853.2	c.2144_2216dup	p.(Glu740GlyfsTer54)	Frameshift	Variant of Uncertain Significance	F	29	Not stated
403570	3178	RPGR	NM_001034853.2	c.1765G>T	p.(Glu589Ter)	Stopgain	Likely Pathogenic	M	73	White - British
18477220	3179	RPGR	NM_001034853.2	c.2521del	p.(Glu841ArgfsTer248)	Frameshift	Likely Pathogenic	M	37	Unknown
3830252	3180	RPGR	NM_001034853.2	c.2992_2996del	p.(Glu998GlyfsTer79)	Frameshift	Likely Pathogenic	M	35	White - British
11813829	3181	RPGR	NM_001034853.2	c.2659_2662del	p.(Gly887LysfsTer201)	Frameshift	Likely Pathogenic	F	53	Unknown
531	3181	RPGR	NM_001034853.2	c.2659_2662del	p.(Gly887LysfsTer201)	Frameshift	Likely Pathogenic	M	55	Not stated
851143	3182	RPGR	NM_001034853.2	c.2426_2427del	p.(Glu809GlyfsTer25)	Frameshift	Pathogenic	M	71	White - British
6796838	3183	RPGR	NM_001034853.2	c.2557G>T	p.(Glu853Ter)	Stopgain	Likely Pathogenic	F	49	Mixed - White and Asian
4978189	3184	RPGR	NM_001034853.2	c.2426_2427del	p.(Glu809GlyfsTer25)	Frameshift	Pathogenic	M	65	White - British
1600759	3184	RPGR	NM_001034853.2	c.2426_2427del	p.(Glu809GlyfsTer25)	Frameshift	Pathogenic	M	52	Not stated
2493658	3185	RPGR	NM_001034853.2	c.2899del	p.(Glu967LysfsTer122)	Frameshift	Likely Pathogenic	M	40	White - British
1155391	3186	RPGR	NM_001034853.2	c.1345C>T	p.(Arg449Ter)	Stopgain	Pathogenic	M	60	White - British
4876059	3187	RPGR	NM_001034853.2	c.415G>T	p.(Glu139Ter)	Stopgain	Likely Pathogenic	M	32	Not stated
18208777	3188	RPGR	NM_001034853.2	c.1216_1217del	p.(Leu406IlefsTer46)	Frameshift	Pathogenic	M	9	Not stated
1136456	3189	RPGR	NM_001034853.2	c.1951_1954del	p.(Pro651LysfsTer45)	Frameshift	Likely Pathogenic	M	60	Not stated
1240322	3190	RPGR	NM_001034853.2	c.779-3C>A	Splice	Splice	Likely Pathogenic	M	68	Not stated
1310637	3191	RPGR	NM_001034853.2	c.2416del	p.(Glu806ArgfsTer9)	Frameshift	Likely Pathogenic	M	66	Unknown
1562028	3192	RPGR	NM_001034853.2	c.1926dup	p.(Ser643IlefsTer10)	Frameshift	Pathogenic	M	62	Not stated
1571576	3193	RPGR	NM_001034853.2	c.295A>G	p.(Thr99Ala)	Missense	Variant of Uncertain Significance	M	48	Not stated
15564527	3193	RPGR	NM_001034853.2	c.295A>G	p.(Thr99Ala)	Missense	Variant of Uncertain Significance	M	33	Not stated
11066138	3194	RPGR	NM_001034853.2	c.2405_2406del	p.(Glu802GlyfsTer32)	Frameshift	Pathogenic	M	42	Unknown
13473606	3194	RPGR	NM_001034853.2	c.2405_2406del	p.(Glu802GlyfsTer32)	Frameshift	Pathogenic	M	35	Not stated
13486017	3194	RPGR	NM_001034853.2	c.2405_2406del	p.(Glu802GlyfsTer32)	Frameshift	Pathogenic	M	40	Not stated
6094402	3195	RPGR	NM_001034853.2	c.2236_2237del	p.(Glu746ArgfsTer23)	Frameshift	Pathogenic	M	60	White - British
2751790	3196	RPGR	NM_001034853.2	c.778+5G>T	Splice	Splice	Variant of Uncertain Significance	M	58	Not stated
5205787	3197	RPGR	NM_001034853.2	c.2625dup	p.(Gly876ArgfsTer203)	Frameshift	Pathogenic	M	29	White - British
1663171	3198	RPGR	NM_001034853.2	c.2929G>T	p.(Gly977Ter)	Stopgain	Likely Pathogenic	M	63	Not stated
7221073	3199	RPGR	NM_001034853.2	c.294C>A	p.(His98Gln)	Missense	Variant of Uncertain Significance	M	63	White - British
6185017	3200	RPGR	NM_001034853.2	c.1571del	p.(Lys524ArgfsTer9)	Frameshift	Likely Pathogenic	M	47	Not stated
4952765	3201	RPGR	NM_001034853.2	c.2405_2406del	p.(Glu802GlyfsTer32)	Frameshift	Pathogenic	M	66	White - British
2741087	3202	RPGR	NM_001034853.2	c.194G>T	p.(Gly65Val)	Missense	Likely Pathogenic	M	54	White - British
3134753	3203	RPGR	NM_001034853.2	c.2054_2133dup	p.(Gln712LysfsTer12)	Frameshift	Variant of Uncertain Significance	M	47	Not stated
3111492	3203	RPGR	NM_001034853.2	c.2054_2133dup	p.(Gln712LysfsTer12)	Frameshift	Variant of Uncertain Significance	M	54	Not stated
3134760	3203	RPGR	NM_001034853.2	c.2054_2133dup	p.(Gln712LysfsTer12)	Frameshift	Variant of Uncertain Significance	F	48	White - British
4473426	3204	RPGR	NM_001034853.2	Exon 14-15 deletion	Deletion	Deletion	Likely Pathogenic	M	48	White - British
12332662	3205	RPGR	NM_001034853.2	c.2628_2629del	p.(Glu877GlyfsTer201)	Frameshift	Pathogenic	M	47	Asian - Indian
3671121	3206	RPGR	NM_001034853.2	c.1234C>T	p.(Arg412Ter)	Stopgain	Pathogenic	M	42	Not stated
9062549	3207	RPGR	NM_001034853.2	c.2601_2602del	p.(Glu868GlyfsTer210)	Frameshift	Pathogenic	M	37	Any other ethnic group
7376186	3208	RPGR	NM_001034853.2	c.1234C>T	p.(Arg412Ter)	Stopgain	Pathogenic	M	27	Black - African
4401641	3208	RPGR	NM_001034853.2	c.1234C>T	p.(Arg412Ter)	Stopgain	Pathogenic	M	64	Black - Caribbean
7820770	3209	RPGR	NM_001034853.2	c.2907_2910del	p.(Gly970LysfsTer118)	Frameshift	Likely Pathogenic	M	30	Not stated
4767076	3210	RPGR	NM_001034853.2	c.2426_2427del	p.(Glu809GlyfsTer25)	Frameshift	Pathogenic	M	46	Not stated
18334756	3210	RPGR	NM_001034853.2	c.2426_2427del	p.(Glu809GlyfsTer25)	Frameshift	Pathogenic	M	24	Not stated
16439702	3211	RPGR	NM_001034853.2	c.2257_2260del	p.(Gly753LysfsTer61)	Frameshift	Pathogenic	M	43	Not stated

4973548	3212	RPGR	NM_001034853.2	c.2625dup	p.(Gly876ArgfsTer203)	Frameshift	Pathogenic	M	55	Not stated
8778391	3213	RPGR	NM_001034853.2	c.2236_2237del	p.(Glu746ArgfsTer23)	Frameshift	Pathogenic	M	44	Asian - Indian
4995339	3213	RPGR	NM_001034853.2	c.2236_2237del	p.(Glu746ArgfsTer23)	Frameshift	Pathogenic	M	53	Asian - Indian
5413071	3214	RPGR	NM_001034853.2	c.2236_2237del	p.(Glu746ArgfsTer23)	Frameshift	Pathogenic	M	55	Not stated
7184862	3215	RPGR	NM_001034853.2	c.2650G>T	p.(Glu884Ter)	Stopgain	Pathogenic	M	53	White - British
4783302	3216	RPGR	NM_001034853.2	c.3092del	p.(Glu1031GlyfsTer58)	Frameshift	Pathogenic	M	69	White - British
5761965	3217	RPGR	NM_001034853.2	c.2426_2427del	p.(Glu809GlyfsTer25)	Frameshift	Pathogenic	M	57	White - British
6219660	3218	RPGR	NM_001034853.2	c.1572+1G>A	Splice	Splice	Likely Pathogenic	M	34	Not stated
6296429	3219	RPGR	NM_001034853.2	c.2212G>T	p.(Gly738Ter)	Stopgain	Likely Pathogenic	M	57	Black - Caribbean
6477295	3220	RPGR	NM_001034853.2	c.2238del	p.(Glu747ArgfsTer68)	Frameshift	Likely Pathogenic	M	30	Not stated
3218753	3221	RPGR	NM_001034853.2	c.2442_2445del	p.(Gly817LysfsTer2)	Frameshift	Pathogenic	M	74	White - British
9895129	3222	RPGR	NM_001034853.2	c.3092del	p.(Glu1031GlyfsTer58)	Frameshift	Pathogenic	M	38	White - British
6191121	3222	RPGR	NM_001034853.2	c.3092del	p.(Glu1031GlyfsTer58)	Frameshift	Pathogenic	M	59	Not stated
3969496	3223	RPGR	NM_001034853.2	c.836_934+1276del	Deletion	Deletion	Likely Pathogenic	M	36	White - British
6927794	3223	RPGR	NM_001034853.2	c.836_934+1276del	Deletion	Deletion	Likely Pathogenic	M	27	White - British
13007385	3223	RPGR	NM_001034853.2	c.836_934+1276del	Deletion	Deletion	Likely Pathogenic	F	38	Not stated
12019216	3224	RPGR	NM_001034853.2	c.2384del	p.(Glu795GlyfsTer20)	Frameshift	Pathogenic	M	17	White - British
6796607	3225	RPGR	NM_001034853.2	c.914dup	p.(Asn305LysfsTer41)	Frameshift	Pathogenic	M	35	Any other ethnic group
6975891	3226	RPGR	NM_001034853.2	Exon 6-11 deletion	Deletion	Deletion	Likely Pathogenic	M	49	Asian - Indian
3417616	3227	RPGR	NM_001034853.2	c.2426_2427del	p.(Glu809GlyfsTer25)	Frameshift	Pathogenic	M	56	Asian - Bangladeshi
5952834	3228	RPGR	NM_001034853.2	c.2997_2998del	p.(Glu1000GlyfsTer78)	Frameshift	Pathogenic	M	51	White - British
7248569	3229	RPGR	NM_001034853.2	c.2650G>T	p.(Glu884Ter)	Stopgain	Pathogenic	M	82	White - British
10101503	3230	RPGR	NM_001034853.2	c.2848G>T	p.(Glu950Ter)	Stopgain	Pathogenic	M	33	Not stated
3327365	3231	RPGR	NM_001034853.2	c.126T>G	p.(Cys42Trp)	Missense	Variant of Uncertain Significance	M	62	Asian - Pakistani
12129837	3231	RPGR	NM_001034853.2	c.126T>G	p.(Cys42Trp)	Missense	Variant of Uncertain Significance	M	60	White - British
8863385	3232	RPGR	NM_001034853.2	c.1516dup	p.(Met506AsnfsTer7)	Frameshift	Likely Pathogenic	M	26	Not stated
9202850	3233	RPGR	NM_001034853.2	c.2405_2406del	p.(Glu802GlyfsTer32)	Frameshift	Pathogenic	M	50	Not stated
1604399	3234	RPGR	NM_001034853.2	c.2426_2427del	p.(Glu809GlyfsTer25)	Frameshift	Pathogenic	F	62	White - British
8464623	3235	RPGR	NM_001034853.2	c.2426_2427del	p.(Glu809GlyfsTer25)	Frameshift	Pathogenic	M	52	Not stated
9397394	3236	RPGR	NM_001034853.2	c.1894_1897del	p.(Asp632LysfsTer13)	Frameshift	Likely Pathogenic	F	39	Not stated
16068982	3236	RPGR	NM_001034853.2	c.1894_1897del	p.(Asp632LysfsTer13)	Frameshift	Likely Pathogenic	F	12	White - British
9545108	3237	RPGR	NM_001034853.2	c.1243_1244del	p.(Arg415GlyfsTer37)	Frameshift	Pathogenic	M	73	Not stated
9564190	3238	RPGR	NM_001034853.2	c.3092del	p.(Glu1031GlyfsTer58)	Frameshift	Pathogenic	M	45	Not stated
9080315	3239	RPGR	NM_001034853.2	c.1500A>C	p.(Leu500Phe)	Missense	Variant of Uncertain Significance	M	60	Not stated
9080315	3239	RPGR	NM_001034853.2	c.177dup	p.(Gly60TrpfsTer3)	Frameshift	Likely Pathogenic	M	60	Not stated
9836371	3240	RPGR	NM_001034853.2	c.2763_2764del	p.(Glu922GlyfsTer156)	Frameshift	Pathogenic	M	40	Not stated
1186863	3241	RPGR	NM_001034853.2	c.2426_2427del	p.(Glu809GlyfsTer25)	Frameshift	Pathogenic	M	70	Not stated
8904230	3242	RPGR	NM_001034853.2	c.296C>T	p.(Thr99Ile)	Missense	Variant of Uncertain Significance	F	23	Asian - Bangladeshi
9552087	3242	RPGR	NM_001034853.2	c.296C>T	p.(Thr99Ile)	Missense	Variant of Uncertain Significance	M	20	Not stated
10182192	3243	RPGR	NM_001034853.2	c.2860G>T	p.(Glu954Ter)	Stopgain	Likely Pathogenic	M	57	Not stated
9535007	3244	RPGR	NM_001034853.2	c.2405_2406del	p.(Glu802GlyfsTer32)	Frameshift	Pathogenic	M	27	Not stated
9556700	3245	RPGR	NM_001034853.2	c.3178_3179del	p.(Glu1060ArgfsTer18)	Frameshift	Pathogenic	M	54	Not stated
13155190	3245	RPGR	NM_001034853.2	c.3178_3179del	p.(Glu1060ArgfsTer18)	Frameshift	Pathogenic	F	75	Unknown
2801476	3246	RPGR	NM_001034853.2	c.2405_2406del	p.(Glu802GlyfsTer32)	Frameshift	Pathogenic	M	51	Not stated
10357038	3247	RPGR	NM_001034853.2	c.1234C>T	p.(Arg412Ter)	Stopgain	Pathogenic	M	24	White - British
10440590	3248	RPGR	NM_001034853.2	c.2045_2046dup	p.(Arg683ValfsTer15)	Frameshift	Likely Pathogenic	M	29	Not stated
9571708	3249	RPGR	NM_001034853.2	c.3178_3179del	p.(Glu1060ArgfsTer18)	Frameshift	Pathogenic	M	61	Not stated
6116858	3249	RPGR	NM_001034853.2	c.3178_3179del	p.(Glu1060ArgfsTer18)	Frameshift	Pathogenic	M	57	Not stated
10741450	3250	RPGR	NM_001034853.2	c.2426_2427del	p.(Glu809GlyfsTer25)	Frameshift	Pathogenic	M	42	White - Other
10769975	3251	RPGR	NM_001034853.2	c.2236_2237del	p.(Glu746ArgfsTer23)	Frameshift	Pathogenic	M	51	Not stated
10277469	3252	RPGR	NM_001034853.2	c.494G>T	p.(Gly165Val)	Missense	Pathogenic	M	56	Not stated

10884362	3253	RPGR	NM_001034853.2	c.29-1_29delinsAT	Splice	Splice	Likely Pathogenic	M	65	White - British
10854752	3254	RPGR	NM_001034853.2	c.2763_2764del	p.(Glu922GlyfsTer156)	Frameshift	Pathogenic	M	60	White - British
18152147	3254	RPGR	NM_001034853.2	c.2763_2764del	p.(Glu922GlyfsTer156)	Frameshift	Pathogenic	M	7	Not stated
6399518	3255	RPGR	NM_001034853.2	c.2384del	p.(Glu795GlyfsTer20)	Frameshift	Pathogenic	M	52	Not stated
10993359	3255	RPGR	NM_001034853.2	c.2384del	p.(Glu795GlyfsTer20)	Frameshift	Pathogenic	M	50	Any other ethnic group
10470480	3256	RPGR	NM_001034853.2	c.2601_2602delGG	p.(Glu868GlyfsTer210)	Frameshift	Pathogenic	M	39	Not stated
10562866	3257	RPGR	NM_001034853.2	c.2236_2237del	p.(Glu746ArgfsTer23)	Frameshift	Pathogenic	M	38	White - British
10353902	3258	RPGR	NM_001034853.2	c.2426_2427del	p.(Glu809GlyfsTer25)	Frameshift	Pathogenic	F	41	White - Other
4700870	3258	RPGR	NM_001034853.2	c.2426_2427del	p.(Glu809GlyfsTer25)	Frameshift	Pathogenic	F	66	White - Other
11089238	3259	RPGR	NM_001034853.2	c.2997_2998del	p.(Glu1000GlyfsTer78)	Frameshift	Pathogenic	F	54	Unknown
11062253	3260	RPGR	NM_001034853.2	c.880del	p.(Asp294IlefsTer4)	Frameshift	Likely Pathogenic	M	27	White - British
11278287	3261	RPGR	NM_001034853.2	c.2521G>T	p.(Glu841Ter)	Stopgain	Likely Pathogenic	M	32	White - Other
11233816	3262	RPGR	NM_001034853.2	c.2586_2587del	p.(Glu863ArgfsTer215)	Frameshift	Likely Pathogenic	M	47	Unknown
12302436	3263	RPGR	NM_001034853.2	c.2027_2039del	p.(Lys676ThrfsTer17)	Frameshift	Likely Pathogenic	M	16	Not stated
10639782	3264	RPGR	NM_001034853.2	c.3317dup	p.(Ser1107ValfsTer4)	Frameshift	Pathogenic	M	59	White - British
11700247	3265	RPGR	NM_001034853.2	c.2323_2324del	p.(Arg775GlufsTer59)	Frameshift	Pathogenic	M	55	White - British
11185215	3266	RPGR	NM_001034853.2	c.1429G>T	p.(Glu477Ter)	Stopgain	Pathogenic	M	56	White - Other
3595759	3267	RPGR	NM_001034853.2	c.2929G>T	p.(Gly977Ter)	Stopgain	Likely Pathogenic	M	57	Any other ethnic group
3717741	3268	RPGR	NM_001034853.2	c.3096_3097del	p.(Glu1033ArgfsTer45)	Frameshift	Pathogenic	M	60	Not stated
11114165	3269	RPGR	NM_001034853.2	c.958G>T	p.(Gly320Ter)	Stopgain	Likely Pathogenic	M	46	Black - Caribbean
7896216	3270	RPGR	NM_001034853.2	c.2236_2237del	p.(Glu746ArgfsTer23)	Frameshift	Pathogenic	M	50	Any other ethnic group
11757766	3271	RPGR	NM_001034853.2	c.3050_3051del	p.(Glu1017GlyfsTer61)	Frameshift	Pathogenic	M	37	Any other ethnic group
12122459	3272	RPGR	NM_001034853.2	c.3039_3040del	p.(Glu1014GlyfsTer64)	Frameshift	Pathogenic	M	41	Asian - Indian
12138937	3273	RPGR	NM_001034853.2	c.2405_2406del	p.(Glu802GlyfsTer32)	Frameshift	Pathogenic	F	30	Not stated
1073099	3274	RPGR	NM_001034853.2	c.2986_3014del	p.(Glu996ArgfsTer73)	Frameshift	Likely Pathogenic	F	62	White - British
12339207	3275	RPGR	NM_001034853.2	c.3092del	p.(Glu1031GlyfsTer58)	Frameshift	Pathogenic	M	46	Unknown
11800389	3276	RPGR	NM_001034853.2	c.1572G>A	p.(Lys524Lys)	Synonymous	Variant of Uncertain Significance	M	16	White - Other
4089574	3277	RPGR	NM_001034853.2	c.3013_3014del	p.(Gly1005ArgfsTer73)	Frameshift	Likely Pathogenic	M	65	Not stated
12495692	3278	RPGR	NM_001034853.2	c.350G>A	p.(Gly117Glu)	Missense	Variant of Uncertain Significance	M	54	White - British
9980487	3279	RPGR	NM_001034853.2	c.2993_2997del	p.(Glu998GlyfsTer79)	Frameshift	Likely Pathogenic	M	31	Not stated
4843054	3280	RPGR	NM_001034853.2	c.2506G>T	p.(Glu836Ter)	Stopgain	Likely Pathogenic	M	84	Asian - Indian
10123350	3281	RPGR	NM_001034853.2	c.2252_2255del	p.(Lys751ArgfsTer63)	Frameshift	Pathogenic	M	46	White - British
3838155	3282	RPGR	NM_001034853.2	c.1237A>T	p.(Arg413Ter)	Stopgain	Pathogenic	M	34	Asian - Bangladeshi
12790343	3283	RPGR	NM_001034853.2	c.2091dup	p.(Glu698ArgfsTer72)	Frameshift	Likely Pathogenic	M	40	Any other ethnic group
12602316	3284	RPGR	NM_001034853.2	c.2405_2406del	p.(Glu802GlyfsTer32)	Frameshift	Pathogenic	M	59	Any other ethnic group
12862289	3285	RPGR	NM_001034853.2	c.1243_1244del	p.(Arg415GlyfsTer37)	Frameshift	Pathogenic	M	30	Not stated
18116944	3285	RPGR	NM_001034853.2	c.1243_1244del	p.(Arg415GlyfsTer37)	Frameshift	Pathogenic	M	21	Unknown
12850396	3286	RPGR	NM_001034853.2	c.2250_2251del	p.(Lys751GlyfsTer18)	Frameshift	Likely Pathogenic	M	34	Not stated
8053674	429	RPGR	NM_001034853.2	c.2405_2406del	p.(Glu802GlyfsTer32)	Frameshift	Pathogenic	F	45	White - British
11153351	3287	RPGR	NM_001034853.2	c.1234C>T	p.(Arg412Ter)	Stopgain	Pathogenic	F	48	Not stated
13300937	3287	RPGR	NM_001034853.2	c.1234C>T	p.(Arg412Ter)	Stopgain	Pathogenic	M	27	Any other ethnic group
309364	3288	RPGR	NM_001034853.2	c.1393del	p.(Leu465SerfsTer11)	Frameshift	Pathogenic	M	66	Not stated
13033901	3289	RPGR	NM_001034853.2	c.3320_3323del	p.(Ser1107LeufsTer23)	Frameshift	Likely Pathogenic	M	45	Not stated
10120690	3290	RPGR	NM_001034853.2	c.2236_2237del	p.(Glu746ArgfsTer23)	Frameshift	Pathogenic	M	56	Any other ethnic group
10791948	3291	RPGR	NM_001034853.2	c.1387C>T	p.(Gln463Ter)	Stopgain	Pathogenic	M	27	White - British
1095100	3292	RPGR	NM_001034853.2	c.3139G>T	p.(Glu1047Ter)	Stopgain	Likely Pathogenic	M	64	Not stated
13449827	3293	RPGR	NM_001034853.2	c.2405_2406del	p.(Glu802GlyfsTer32)	Frameshift	Pathogenic	M	14	Unknown
17369820	3293	RPGR	NM_001034853.2	c.2405_2406del	p.(Glu802GlyfsTer32)	Frameshift	Pathogenic	M	37	Not stated
13537838	3294	RPGR	NM_001034853.2	c.2140del	p.(Glu714ArgfsTer101)	Frameshift	Likely Pathogenic	F	61	Unknown
13716128	3295	RPGR	NM_001034853.2	c.3453G>T	p.(Leu1151Phe)	Missense	Variant of Uncertain Significance	M	46	Not stated
13839195	3296	RPGR	NM_001034853.2	c.2899del	p.(Glu967LysfsTer122)	Frameshift	Likely Pathogenic	M	24	Unknown

18397770	3296	RPGR	NM_001034853.2	c.2899del	p.(Glu967LysfsTer122)	Frameshift	Likely Pathogenic	F	56	White - British
13808570	3297	RPGR	NM_001034853.2	c.2405_2406del	p.(Glu802GlyfsTer32)	Frameshift	Pathogenic	M	27	Not stated
13833966	3298	RPGR	NM_001034853.2	c.2628_2629del	p.(Glu877GlyfsTer201)	Frameshift	Pathogenic	F	48	Not stated
14800470	3299	RPGR	NM_001034853.2	c.2586_2587del	p.(Glu877GlyfsTer201)	Frameshift	Likely Pathogenic	M	15	Not stated
11133660	3300	RPGR	NM_001034853.2	c.2384del	p.(Glu795GlyfsTer20)	Frameshift	Pathogenic	M	19	Asian - Other
6266140	3301	RPGR	NM_001034853.2	c.2650G>T	p.(Glu884Ter)	Stopgain	Pathogenic	M	33	Not stated
13274505	3302	RPGR	NM_001034853.2	c.2628_2629del	p.(Glu877GlyfsTer201)	Frameshift	Pathogenic	M	74	Asian - Indian
13978089	3303	RPGR	NM_001034853.2	c.2854G>T	p.(Glu952Ter)	Stopgain	Likely Pathogenic	F	52	White - British
15104228	3304	RPGR	NM_001034853.2	c.1307G>A	p.(Gly436Asp)	Missense	Variant of Uncertain Significance	M	49	White - British
13803089	3305	RPGR	NM_001034853.2	c.2426_2427del	p.(Glu809GlyfsTer25)	Frameshift	Pathogenic	M	32	White - British
15635010	3306	RPGR	NM_001034853.2	c.1754-3C>G	Splice	Splice	Variant of Uncertain Significance	M	60	White - British
15701125	3307	RPGR	NM_001034853.2	c.2006G>A	p.(Trp669Ter)	Stopgain	Likely Pathogenic	M	34	Any other ethnic group
16954559	1990	RPGR	NM_001034853.2	c.639_640del	p.(Phe214TrpfsTer4)	Frameshift	Likely Pathogenic	M	11	Not stated
15219077	3308	RPGR	NM_001034853.2	c.1377_1378del	p.(Leu460IlefsTer2)	Frameshift	Pathogenic	M	21	White - British
10370051	3309	RPGR	NM_001034853.2	c.3136_3137del	p.(Arg1046GlyfsTer32)	Frameshift	Likely Pathogenic	M	63	Asian - Indian
16127047	3310	RPGR	NM_001034853.2	c.2792del	p.(Glu931GlyfsTer158)	Frameshift	Likely Pathogenic	M	34	White - Other
8494128	3311	RPGR	NM_001034853.2	c.2426_2427del	p.(Glu809GlyfsTer25)	Frameshift	Pathogenic	M	22	White - British
4445755	3312	RPGR	NM_001034853.2	c.2405_2406del	p.(Glu802GlyfsTer32)	Frameshift	Pathogenic	M	50	Not stated
16645096	3313	RPGR	NM_001034853.2	c.2405_2406del	p.(Glu802GlyfsTer32)	Frameshift	Pathogenic	F	54	Not stated
16999303	3314	RPGR	NM_001034853.2	c.2248G>T	p.(Glu750Ter)	Stopgain	Likely Pathogenic	M	25	White - British
11128550	3315	RPGR	NM_001034853.2	Whole gene deletion	Deletion	Deletion	Likely Pathogenic	F	25	White - British
17122888	3316	RPGR	NM_001034853.2	c.2257G>T	p.(Gly753Ter)	Stopgain	Pathogenic	M	29	White - British
16963757	3317	RPGR	NM_001034853.2	c.2442_2445del	p.(Gly817LysfsTer2)	Frameshift	Pathogenic	M	16	Not stated
17227601	3318	RPGR	NM_001034853.2	c.1572+1G>T	Splice	Splice	Likely Pathogenic	F	41	Not stated
17245367	3319	RPGR	NM_001034853.2	c.2840del	p.(Glu947GlyfsTer142)	Frameshift	Likely Pathogenic	M	33	Not stated
17378871	3320	RPGR	NM_001034853.2	c.2118_2190dup	p.(Gly731TrpfsTer63)	Frameshift	Variant of Uncertain Significance	M	20	Not stated
17500188	3321	RPGR	NM_001034853.2	c.2442_2445del	p.(Gly817LysfsTer2)	Frameshift	Pathogenic	F	66	Not stated
17483717	3322	RPGR	NM_001034853.2	c.3096_3097del	p.(Glu1033ArgfsTer45)	Frameshift	Pathogenic	M	37	Not stated
12486144	3323	RPGR	NM_001034853.2	c.2236_2237del	p.(Glu746ArgfsTer23)	Frameshift	Pathogenic	M	45	Not stated
17107859	3324	RPGR	NM_001034853.2	c.3178_3179del	p.(Glu1060ArgfsTer18)	Frameshift	Pathogenic	M	60	Unknown
17650002	3325	RPGR	NM_001034853.2	c.1414+2T>A	Splice	Splice	Likely Pathogenic	M	11	Not stated
17700731	3326	RPGR	NM_001034853.2	c.2426_2427del	p.(Glu809GlyfsTer25)	Frameshift	Pathogenic	M	7	Not stated
17700724	3326	RPGR	NM_001034853.2	c.2426_2427del	p.(Glu809GlyfsTer25)	Frameshift	Pathogenic	M	12	Not stated
17645844	3327	RPGR	NM_001034853.2	c.1234C>T	p.(Arg412Ter)	Stopgain	Pathogenic	M	58	Not stated
17747981	3328	RPGR	NM_001034853.2	c.393dup	p.(Val132CysfsTer3)	Frameshift	Likely Pathogenic	F	45	Not stated
13831551	3329	RPGR	NM_001034853.2	c.2405_2406del	p.(Glu802GlyfsTer32)	Frameshift	Pathogenic	M	19	Not stated
17630283	3330	RPGR	NM_001034853.2	c.2257_2260del	p.(Gly753LysfsTer61)	Frameshift	Pathogenic	M	26	Not stated
16399564	3331	RPGR	NM_001034853.2	c.2236_2237del	p.(Glu746ArgfsTer23)	Frameshift	Pathogenic	F	19	Not stated
17926866	3332	RPGR	NM_001034853.2	c.470-2A>G	Splice	Splice	Likely Pathogenic	M	12	White - British
17764809	3333	RPGR	NM_001034853.2	c.1912G>T	p.(Glu638Ter)	Stopgain	Likely Pathogenic	M	48	White - British
17406885	3334	RPGR	NM_001034853.2	c.2236_2237del	p.(Glu746ArgfsTer23)	Frameshift	Pathogenic	F	49	Not stated
18350660	3335	RPGR	NM_001034853.2	c.2509G>T	p.(Glu837Ter)	Stopgain	Likely Pathogenic	M	24	Not stated
805755	3336	RPGR	NM_001034853.2	c.2323_2324del	p.(Arg775GlufsTer59)	Frameshift	Pathogenic	M	52	White - British
18454890	3337	RPGR	NM_001034853.2	c.814G>A	p.(Gly272Ser)	Missense	Likely Pathogenic	M	32	Unknown
18640796	3338	RPGR	NM_001034853.2	c.2997_2998del	p.(Glu1000GlyfsTer78)	Frameshift	Pathogenic	M	21	Unknown
18392793	3339	RPGR	NM_001034853.2	c.1345C>T	p.(Arg449Ter)	Stopgain	Pathogenic	M	35	Not stated
18361440	3340	RPGR	NM_001034853.2	c.1234C>T	p.(Arg412Ter)	Stopgain	Pathogenic	M	26	Not stated
2824121	3341	RPGRIP1	NM_020366.4	c.1447C>T	p.(Gln483Ter)	Stopgain	Pathogenic	M	58	White - British
2824121	3341	RPGRIP1	NM_020366.4	c.2108T>C	p.(Ile703Thr)	Missense	Variant of Uncertain Significance	M	58	White - British
2824121	3341	RPGRIP1	NM_020366.4	c.2471C>T	p.(Ala824Val)	Missense	Variant of Uncertain Significance	M	58	White - British
7561224	3342	RPGRIP1	NM_020366.4	c.2941C>T	p.(Arg981Ter)	Stopgain	Pathogenic	M	23	White - Other

7561224	3342	RPGRIP1	NM_020366.4	c.3120G>A	p.(Trp1040Ter)	Stopgain	Pathogenic	M	23	White - Other
9184622	3343	RPGRIP1	NM_020366.4	c.1107del	p.(Glu370AsnfsTer5)	Frameshift	Pathogenic	M	29	Asian - Pakistani
9184622	3343	RPGRIP1	NM_020366.4	c.1107del	p.(Glu370AsnfsTer5)	Frameshift	Pathogenic	M	29	Asian - Pakistani
9397779	3343	RPGRIP1	NM_020366.4	c.1107del	p.(Glu370AsnfsTer5)	Frameshift	Pathogenic	M	25	Asian - Other
9397779	3343	RPGRIP1	NM_020366.4	c.1107del	p.(Glu370AsnfsTer5)	Frameshift	Pathogenic	M	25	Asian - Other
10303901	3344	RPGRIP1	NM_020366.4	c.1116del	p.(Lys372AsnfsTer3)	Frameshift	Pathogenic	F	18	White - British
10303901	3344	RPGRIP1	NM_020366.4	c.3263C>T	p.(Ser1088Phe)	Missense	Variant of Uncertain Significance	F	18	White - British
9399445	3345	RPGRIP1	NM_020366.4	c.2890del	p.(Ser964ProfsTer37)	Frameshift	Pathogenic	F	28	Any other ethnic group
9399445	3345	RPGRIP1	NM_020366.4	c.2890del	p.(Ser964ProfsTer37)	Frameshift	Pathogenic	F	28	Any other ethnic group
11866469	3346	RPGRIP1	NM_020366.4	c.1303A>T	p.(Lys435Ter)	Stopgain	Pathogenic	F	14	Unknown
11866469	3346	RPGRIP1	NM_020366.4	c.1447C>T	p.(Gln483Ter)	Stopgain	Pathogenic	F	14	Unknown
11276068	3347	RPGRIP1	NM_020366.4	c.2398G>A	p.(Glu800Lys)	Missense	Pathogenic	F	20	Not stated
11276068	3347	RPGRIP1	NM_020366.4	c.2398G>A	p.(Glu800Lys)	Missense	Pathogenic	F	20	Not stated
12870808	3348	RPGRIP1	NM_020366.4	c.2941C>T	p.(Arg981Ter)	Stopgain	Pathogenic	M	30	Any other ethnic group
12870808	3348	RPGRIP1	NM_020366.4	c.2941C>T	p.(Arg981Ter)	Stopgain	Pathogenic	M	30	Any other ethnic group
16646629	3349	RPGRIP1	NM_020366.4	c.3340-3C>G	Splice	Splice	Variant of Uncertain Significance	F	7	Not stated
16646629	3349	RPGRIP1	NM_020366.4	c.973C>T	p.(Gln325Ter)	Stopgain	Likely Pathogenic	F	7	Not stated
17443572	3350	RS1	NM_000330.4	c.216G>C	p.(Glu72Asp)	Missense	Likely Pathogenic	M	10	Not stated
4700352	3351	RS1	NM_000330.4	c.206T>C	p.(Leu69Pro)	Missense	Likely Pathogenic	M	69	Any other ethnic group
4916757	3352	RS1	NM_000330.4	c.579dup	p.(Ile194HisfsTer70)	Frameshift	Pathogenic	M	57	White - British
5526604	3353	RS1	NM_000330.4	c.325G>C	p.(Gly109Arg)	Missense	Likely Pathogenic	M	32	Not stated
6559601	3354	RS1	NM_000330.4	c.637C>T	p.(Arg213Trp)	Missense	Pathogenic	M	43	Not stated
1005710	3355	RS1	NM_000330.4	c.214G>A	p.(Glu72Lys)	Missense	Pathogenic	M	70	Not stated
1078727	3355	RS1	NM_000330.4	c.214G>A	p.(Glu72Lys)	Missense	Pathogenic	M	72	Not stated
763524	3356	RS1	NM_000330.4	c.304C>T	p.(Arg102Trp)	Missense	Likely Pathogenic	M	60	Any other ethnic group
6585795	3357	RS1	NM_000330.4	c.305G>A	p.(Arg102Gln)	Missense	Pathogenic	M	59	White - British
3108951	3357	RS1	NM_000330.4	c.305G>A	p.(Arg102Gln)	Missense	Pathogenic	M	39	Not stated
12708030	3357	RS1	NM_000330.4	c.305G>A	p.(Arg102Gln)	Missense	Pathogenic	M	13	White - British
13281799	3357	RS1	NM_000330.4	c.305G>A	p.(Arg102Gln)	Missense	Pathogenic	M	41	White - British
9550778	3357	RS1	NM_000330.4	c.305G>A	p.(Arg102Gln)	Missense	Pathogenic	M	46	White - British
3624725	3358	RS1	NM_000330.4	c.304C>T	p.(Arg102Trp)	Missense	Likely Pathogenic	M	39	Not stated
1504166	3359	RS1	NM_000330.4	Exon 2-3 deletion	Deletion	Deletion	Likely Pathogenic	M	44	Not stated
15557422	3360	RS1	NM_000330.4	c.304C>T	p.(Arg102Trp)	Missense	Likely Pathogenic	M	15	Not stated
537340	3361	RS1	NM_000330.4	c.214G>C	p.(Glu72Gln)	Missense	Pathogenic	M	58	White - British
2786370	3362	RS1	NM_000330.4	Exon 1 deletion	Deletion	Deletion	Likely Pathogenic	M	51	White - British
9268377	3363	RS1	NM_000330.4	Exon 1 deletion	Deletion	Deletion	Likely Pathogenic	M	60	Not stated
2128685	3364	RS1	NM_000330.4	c.421C>T	p.(Arg141Cys)	Missense	Pathogenic	M	43	Not stated
2804164	3365	RS1	NM_000330.4	Exon 1 deletion	Deletion	Deletion	Likely Pathogenic	M	41	Any other ethnic group
4653403	3366	RS1	NM_000330.4	c.574C>T	p.(Pro192Ser)	Missense	Pathogenic	M	66	Not stated
1176622	3367	RS1	NM_000330.4	c.304C>T	p.(Arg102Trp)	Missense	Likely Pathogenic	M	70	White - British
3645690	3368	RS1	NM_000330.4	Exon 1 deletion	Deletion	Deletion	Likely Pathogenic	M	78	White - British
5127373	3369	RS1	NM_000330.4	c.574C>T	p.(Pro192Ser)	Missense	Pathogenic	M	37	Not stated
1043209	3370	RS1	NM_000330.4	c.305G>A	p.(Arg102Gln)	Missense	Pathogenic	M	66	White - British
5255774	3371	RS1	NM_000330.4	c.78G>C	p.(Glu26Asp)	Missense	Variant of Uncertain Significance	M	64	Not stated
1115946	3372	RS1	NM_000330.4	c.574C>T	p.(Pro192Ser)	Missense	Pathogenic	M	57	Not stated
2661973	3373	RS1	NM_000330.4	c.78+2T>C	Splice	Splice	Pathogenic	M	63	White - British
5697145	3374	RS1	NM_000330.4	c.496_498del	p.(Tyr166del)	Inframe indel	Variant of Uncertain Significance	M	46	Not stated
5065815	3375	RS1	NM_000330.4	c.35T>A	p.(Leu12His)	Missense	Likely Pathogenic	M	33	Not stated
5065815	3375	RS1	NM_000330.4	c.52+5G>C	Splice	Splice	Variant of Uncertain Significance	M	33	Not stated
5486011	3376	RS1	NM_000330.4	c.598C>T	p.(Arg200Cys)	Missense	Pathogenic	M	52	White - British
6777371	3377	RS1	NM_000330.4	Exon 4-5 deletion	Deletion	Deletion	Likely Pathogenic	M	40	Any other ethnic group

14719641	3377	RS1	NM_000330.4	Exon 4-5 deletion	Deletion	Deletion	Likely Pathogenic	M	12	Not stated
1936416	3378	RS1	NM_000330.4	c.608C>T	p.(Pro203Leu)	Missense	Pathogenic	M	88	Not stated
6744009	3379	RS1	NM_000330.4	c.325G>T	p.(Gly109Trp)	Missense	Likely Pathogenic	M	75	White - British
6057841	3380	RS1	NM_000330.4	Exon 1 deletion	Deletion	Deletion	Likely Pathogenic	M	71	Not stated
1171918	3381	RS1	NM_000330.4	c.599G>A	p.(Arg200His)	Missense	Pathogenic	M	62	White - British
7024016	3382	RS1	NM_000330.4	c.574C>T	p.(Pro192Ser)	Missense	Pathogenic	M	69	White - British
3133430	3383	RS1	NM_000330.4	c.421C>T	p.(Arg141Cys)	Missense	Pathogenic	M	53	White - British
3243687	3383	RS1	NM_000330.4	c.421C>T	p.(Arg141Cys)	Missense	Pathogenic	M	48	Unknown
6684852	3384	RS1	NM_000330.4	c.214G>C	p.(Glu72Gln)	Missense	Pathogenic	M	29	White - British
1954441	3385	RS1	NM_000330.4	c.574C>T	p.(Pro192Ser)	Missense	Pathogenic	M	44	Any other ethnic group
7628354	3386	RS1	NM_000330.4	c.574C>T	p.(Pro192Ser)	Missense	Pathogenic	M	48	Mixed - White and Asian
8480569	3387	RS1	NM_000330.4	c.304C>T	p.(Arg102Trp)	Missense	Likely Pathogenic	M	29	Asian - Pakistani
8763236	3388	RS1	NM_000330.4	c.239A>C	p.(Gln80Pro)	Missense	Likely Pathogenic	M	38	White - Irish
7876469	3389	RS1	NM_000330.4	c.304C>T	p.(Arg102Trp)	Missense	Likely Pathogenic	M	25	Not stated
8852332	3390	RS1	NM_000330.4	c.103C>T	p.(Gln35Ter)	Stopgain	Pathogenic	M	31	Asian - Pakistani
9245424	3391	RS1	NM_000330.4	c.598C>T	p.(Arg200Cys)	Missense	Pathogenic	M	19	Any other ethnic group
9295698	3392	RS1	NM_000330.4	c.589C>T	p.(Arg197Cys)	Missense	Pathogenic	M	32	White - British
10464075	3393	RS1	NM_000330.4	c.554C>A	p.(Thr185Lys)	Missense	Pathogenic	M	23	White - British
3232137	3393	RS1	NM_000330.4	c.554C>A	p.(Thr185Lys)	Missense	Pathogenic	M	46	Not stated
10008949	3394	RS1	NM_000330.4	c.35T>A	p.(Leu12His)	Missense	Likely Pathogenic	M	28	Not stated
10008949	3394	RS1	NM_000330.4	c.52+5G>C	Splice	Splice	Variant of Uncertain Significance	M	28	Not stated
9491075	3395	RS1	NM_000330.4	c.598C>T	p.(Arg200Cys)	Missense	Pathogenic	M	59	Not stated
9145877	3396	RS1	NM_000330.4	c.329G>A	p.(Cys110Tyr)	Missense	Pathogenic	M	30	Asian - Other
7113672	3397	RS1	NM_000330.4	c.590G>A	p.(Arg197His)	Missense	Pathogenic	M	55	White - British
9953796	3398	RS1	NM_000330.4	c.589C>T	p.(Arg197Cys)	Missense	Pathogenic	M	16	White - British
9560606	3399	RS1	NM_000330.4	c.647T>C	p.(Leu216Pro)	Missense	Pathogenic	M	25	Not stated
9941728	3400	RS1	NM_000330.4	c.35T>A	p.(Leu12His)	Missense	Likely Pathogenic	M	26	White - British
9941728	3400	RS1	NM_000330.4	c.52+5G>C	Splice	Splice	Variant of Uncertain Significance	M	26	White - British
9429748	3401	RS1	NM_000330.4	c.78G>C	p.(Glu26Asp)	Missense	Variant of Uncertain Significance	M	52	Any other ethnic group
9922793	3402	RS1	NM_000330.4	c.579dup	p.(Ile194HisfsTer70)	Frameshift	Pathogenic	M	21	White - British
1486484	3403	RS1	NM_000330.4	c.317A>C	p.(Gln106Pro)	Missense	Likely Pathogenic	M	39	Unknown
10169039	3404	RS1	NM_000330.4	c.554C>A	p.(Thr185Lys)	Missense	Pathogenic	M	49	Unknown
3334631	3405	RS1	NM_000330.4	c.637C>T	p.(Arg213Trp)	Missense	Pathogenic	M	66	Unknown
1594914	3406	RS1	NM_000330.4	c.599G>A	p.(Arg200His)	Missense	Pathogenic	M	64	White - British
10575081	3407	RS1	NM_000330.4	c.337C>T	p.(Leu113Phe)	Missense	Likely Pathogenic	M	49	Unknown
10429558	3408	RS1	NM_000330.4	c.304C>T	p.(Arg102Trp)	Missense	Likely Pathogenic	M	43	White - British
10573177	3409	RS1	NM_000330.4	c.184+2T>G	Splice	Splice	Pathogenic	M	39	Asian - Indian
10028927	3410	RS1	NM_000330.4	c.496T>C	p.(Tyr166His)	Missense	Likely Pathogenic	M	21	White - Other
10729312	3411	RS1	NM_000330.4	c.574C>T	p.(Pro192Ser)	Missense	Pathogenic	M	42	Not stated
10774952	3412	RS1	NM_000330.4	c.435dup	p.(Glu146Ter)	Stopgain	Likely Pathogenic	M	66	White - British
11181946	3413	RS1	NM_000330.4	Exon 1 deletion	Deletion	Deletion	Likely Pathogenic	M	26	White - Other
8140341	3414	RS1	NM_000330.4	c.304C>T	p.(Arg102Trp)	Missense	Likely Pathogenic	M	56	Not stated
10919894	3415	RS1	NM_000330.4	c.598C>T	p.(Arg200Cys)	Missense	Pathogenic	M	18	Any other ethnic group
10918235	3416	RS1	NM_000330.4	c.598C>T	p.(Arg200Cys)	Missense	Pathogenic	M	29	Unknown
10679990	3417	RS1	NM_000330.4	Exon 2-3 deletion	Deletion	Deletion	Likely Pathogenic	M	42	White - British
1748221	3418	RS1	NM_000330.4	c.575C>T	p.(Pro192Leu)	Missense	Pathogenic	M	77	White - British
8055620	3419	RS1	NM_000330.4	c.214G>A	p.(Glu72Lys)	Missense	Pathogenic	M	22	Any other ethnic group
1110360	3420	RS1	NM_000330.4	c.214G>C	p.(Glu72Gln)	Missense	Pathogenic	M	49	Any other ethnic group
11710327	3421	RS1	NM_000330.4	c.421C>T	p.(Arg141Cys)	Missense	Pathogenic	M	18	White - British
10973080	3422	RS1	NM_000330.4	c.438G>C	p.(Glu146Asp)	Missense	Likely Pathogenic	M	31	Unknown
12068510	3423	RS1	NM_000330.4	c.508A>C	p.(Thr170Pro)	Missense	Variant of Uncertain Significance	M	20	White - British

12235138	3424	RS1	NM_000330.4	c.596T>C	p.(Ile199Thr)	Missense	Pathogenic	M	46	Unknown
6538559	3425	RS1	NM_000330.4	c.120C>A	p.(Cys40Ter)	Stopgain	Likely Pathogenic	M	49	White - British
7813154	3426	RS1	NM_000330.4	c.185-1G>A	Splice	Splice	Likely Pathogenic	M	26	Black - Other
11872412	3427	RS1	NM_000330.4	c.577C>T	p.(Pro193Ser)	Missense	Pathogenic	M	19	Unknown
11720498	3428	RS1	NM_000330.4	c.305G>A	p.(Arg102Gln)	Missense	Pathogenic	M	41	White - British
11641405	3429	RS1	NM_000330.4	c.378del	p.(Leu127Ter)	Stopgain	Likely Pathogenic	M	18	Unknown
12587259	3430	RS1	NM_000330.4	c.35T>A	p.(Leu12His)	Missense	Likely Pathogenic	M	18	Not stated
12881791	3431	RS1	NM_000330.4	c.242T>A	p.(Ile81Asn)	Missense	Likely Pathogenic	M	35	Unknown
13316281	3432	RS1	NM_000330.4	c.326G>C	p.(Gly109Ala)	Missense	Pathogenic	M	13	Black - African
12386807	3433	RS1	NM_000330.4	c.579dup	p.(Ile194HisfsTer70)	Frameshift	Pathogenic	M	47	Unknown
13346283	3434	RS1	NM_000330.4	c.78G>C	p.(Glu26Asp)	Missense	Variant of Uncertain Significance	M	20	Unknown
11975459	3435	RS1	NM_000330.4	c.574_580delinsACCCCCCT	p.(Pro192ThrfsTer72)	Frameshift	Likely Pathogenic	M	18	Any other ethnic group
13308840	3436	RS1	NM_000330.4	c.638G>A	p.(Arg213Gln)	Missense	Likely Pathogenic	M	17	Not stated
1217369	3437	RS1	NM_000330.4	c.421C>T	p.(Arg141Cys)	Missense	Pathogenic	M	49	Not stated
13511035	3438	RS1	NM_000330.4	c.637C>T	p.(Arg213Trp)	Missense	Pathogenic	M	46	Unknown
13687365	3439	RS1	NM_000330.4	c.598C>T	p.(Arg200Cys)	Missense	Pathogenic	M	17	White - British
13385182	3440	RS1	NM_000330.4	c.329G>A	p.(Cys110Tyr)	Missense	Pathogenic	M	23	Not stated
3172392	3441	RS1	NM_000330.4	c.276G>C	p.(Trp92Cys)	Missense	Pathogenic	M	46	Black - Caribbean
13671874	3442	RS1	NM_000330.4	c.214G>A	p.(Glu72Lys)	Missense	Pathogenic	M	14	Any other ethnic group
10872826	3443	RS1	NM_000330.4	c.288G>C	p.(Trp96Cys)	Missense	Pathogenic	M	32	Unknown
14828323	3444	RS1	NM_000330.4	c.214G>A	p.(Glu72Lys)	Missense	Pathogenic	M	45	White - British
14807673	3445	RS1	NM_000330.4	c.544C>T	p.(Arg182Cys)	Missense	Pathogenic	M	12	White - Other
13277648	3446	RS1	NM_000330.4	c.308T>G	p.(Leu103Arg)	Missense	Likely Pathogenic	M	14	White - British
15150288	3447	RS1	NM_000330.4	c.304C>T	p.(Arg102Trp)	Missense	Likely Pathogenic	M	16	Not stated
15382401	3448	RS1	NM_000330.4	c.579dup	p.(Ile194HisfsTer70)	Frameshift	Pathogenic	M	42	Not stated
15359294	3449	RS1	NM_000330.4	c.216G>C	p.(Glu72Asp)	Missense	Likely Pathogenic	M	18	Not stated
15250591	3450	RS1	NM_000330.4	c.589C>T	p.(Arg197Cys)	Missense	Pathogenic	M	26	Not stated
3111275	3451	RS1	NM_000330.4	c.574C>T	p.(Pro192Ser)	Missense	Pathogenic	M	44	Not stated
15440074	3452	RS1	NM_000330.4	c.20del	p.(Gly7AlafsTer119)	Frameshift	Likely Pathogenic	M	42	Any other ethnic group
15782738	3453	RS1	NM_000330.4	c.422G>T	p.(Arg141Leu)	Missense	Likely Pathogenic	M	16	Black - African
15628507	3454	RS1	NM_000330.4	c.421C>T	p.(Arg141Cys)	Missense	Pathogenic	M	31	Any other ethnic group
11799157	3455	RS1	NM_000330.4	c.378del	p.(Leu127Ter)	Stopgain	Likely Pathogenic	M	22	White - British
14778910	3456	RS1	NM_000330.4	c.53-34A>G	Splice	Splice	Variant of Uncertain Significance	M	12	White - British
15989791	3457	RS1	NM_000330.4	c.304C>T	p.(Arg102Trp)	Missense	Likely Pathogenic	M	12	Not stated
2745882	3458	RS1	NM_000330.4	c.304C>T	p.(Arg102Trp)	Missense	Likely Pathogenic	M	68	Not stated
12850641	3459	RS1	NM_000330.4	c.214G>A	p.(Glu72Lys)	Missense	Pathogenic	M	22	Not stated
15600388	3460	RS1	NM_000330.4	c.336_337delinsTT	p.(Trp112_Leu113delinsCysPhe)	Inframe indel	Variant of Uncertain Significance	M	14	Any other ethnic group
16377619	3461	RS1	NM_000330.4	c.214G>A	p.(Glu72Lys)	Missense	Pathogenic	M	25	Not stated
15431898	3462	RS1	NM_000330.4	c.596T>A	p.(Ile199Asn)	Missense	Likely Pathogenic	M	17	Not stated
14724401	3463	RS1	NM_000330.4	c.625C>T	p.(Arg209Cys)	Missense	Pathogenic	M	10	Not stated
16945396	3464	RS1	NM_000330.4	c.52+1G>T	Splice	Splice	Likely Pathogenic	M	10	White - British
12909770	3465	RS1	NM_000330.4	c.78+2T>C	Splice	Splice	Pathogenic	M	34	Unknown
10295039	3466	RS1	NM_000330.4	c.547A>C	p.(Thr183Pro)	Missense	Variant of Uncertain Significance	M	20	Asian - Other
15485896	3467	RS1	NM_000330.4	c.209G>A	p.(Gly70Asp)	Missense	Pathogenic	M	39	Asian - Pakistani
13975023	3468	RS1	NM_000330.4	c.422G>A	p.(Arg141His)	Missense	Pathogenic	M	22	Mixed - Other
8178141	3469	RS1	NM_000330.4	c.598C>T	p.(Arg200Cys)	Missense	Pathogenic	M	65	White - British
16936926	3470	RS1	NM_000330.4	c.214G>A	p.(Glu72Lys)	Missense	Pathogenic	M	55	Not stated
17236519	3471	RS1	NM_000330.4	c.498C>G	p.(Tyr166Ter)	Stopgain	Likely Pathogenic	M	31	White - British
17251597	3472	RS1	NM_000330.4	c.35T>A	p.(Leu12His)	Missense	Likely Pathogenic	M	22	Not stated
17089610	3473	RS1	NM_000330.4	c.35T>A	p.(Leu12His)	Missense	Likely Pathogenic	M	45	White - Other
17089610	3473	RS1	NM_000330.4	c.52+5G>C	Splice	Splice	Variant of Uncertain Significance	M	45	White - Other

17409237	3474	RS1	NM_000330.4	c.349C>T	p.(Gln117Ter)	Stopgain	Pathogenic	M	21	Not stated
17394537	3475	RS1	NM_000330.4	c.608C>T	p.(Pro203Leu)	Missense	Pathogenic	M	49	Not stated
17414417	3476	RS1	NM_000330.4	c.305G>A	p.(Arg102Gln)	Missense	Pathogenic	M	58	Not stated
17615156	3477	RS1	NM_000330.4	c.554C>A	p.(Thr185Lys)	Missense	Pathogenic	M	15	Not stated
17615156	3477	RS1	NM_000330.4	c.598C>A	p.(Arg200Ser)	Missense	Likely Pathogenic	M	15	Not stated
17615163	3477	RS1	NM_000330.4	c.554C>A	p.(Thr185Lys)	Missense	Pathogenic	M	17	Not stated
17615163	3477	RS1	NM_000330.4	c.598C>A	p.(Arg200Ser)	Missense	Likely Pathogenic	M	17	Not stated
8893219	3478	RS1	NM_000330.4	c.515del	p.(Asn172ThrfsTer65)	Frameshift	Likely Pathogenic	M	46	Not stated
17740372	3479	RS1	NM_000330.4	c.78G>C	p.(Glu26Asp)	Missense	Variant of Uncertain Significance	M	12	Not stated
17786173	3480	RS1	NM_000330.4	c.598C>T	p.(Arg200Cys)	Missense	Pathogenic	M	11	White - British
9822168	3481	RS1	NM_000330.4	c.120C>A	p.(Cys40Ter)	Stopgain	Likely Pathogenic	M	24	White - British
8529954	3482	RS1	NM_000330.4	c.574C>T	p.(Pro192Ser)	Missense	Pathogenic	M	30	Not stated
17873862	3483	RS1	NM_000330.4	c.305G>A	p.(Arg102Gln)	Missense	Pathogenic	M	8	Not stated
17903976	3484	RS1	NM_000330.4	c.35T>A	p.(Leu12His)	Missense	Likely Pathogenic	M	26	Not stated
17903976	3484	RS1	NM_000330.4	c.52+5G>C	Splice	Splice	Variant of Uncertain Significance	M	26	Not stated
17872126	3485	RS1	NM_000330.4	c.187T>A	p.(Cys63Ser)	Missense	Variant of Uncertain Significance	M	67	Not stated
17472580	3486	RS1	NM_000330.4	c.421C>T	p.(Arg141Cys)	Missense	Pathogenic	M	11	Not stated
17790086	3487	RS1	NM_000330.4	c.423dup	p.(Cys142LeufsTer2)	Frameshift	Likely Pathogenic	M	51	Not stated
18136131	3488	RS1	NM_000330.4	c.378_382del	p.(Asp126GlufsTer16)	Frameshift	Likely Pathogenic	M	18	Not stated
18179356	3489	RS1	NM_000330.4	c.637C>T	p.(Arg213Trp)	Missense	Pathogenic	M	6	Unknown
17993849	3490	RS1	NM_000330.4	c.53-34A>G	Splice	Splice	Variant of Uncertain Significance	M	5	Not stated
18367019	3491	RS1	NM_000330.4	c.52+5G>C	Splice	Splice	Variant of Uncertain Significance	M	40	Not stated
18367019	3491	RS1	NM_000330.4	c.35T>A	p.(Leu12His)	Missense	Likely Pathogenic	M	40	Not stated
18561521	3492	RS1	NM_000330.4	c.329G>A	p.(Cys110Tyr)	Missense	Pathogenic	M	8	White - British
18598684	3493	RS1	NM_000330.4	c.79-2A>G	Splice	Splice	Likely Pathogenic	M	34	Not stated
18582038	3494	RS1	NM_000330.4	c.574C>G	p.(Pro192Ala)	Missense	Pathogenic	M	20	Not stated
9580815	3495	SAG	NM_000541.5	c.804G>T	p.(Ala268Ala)	Synonymous	Likely Benign	M	48	White - British
9580815	3495	SAG	NM_000541.5	c.966del	p.(Thr323ProfsTer13)	Frameshift	Likely Pathogenic	M	48	White - British
15848384	3496	SAG	NM_000541.5	c.182-2A>G	Splice	Splice	Pathogenic	M	54	Not stated
15848384	3496	SAG	NM_000541.5	c.182-2A>G	Splice	Splice	Pathogenic	M	54	Not stated
15521582	3497	SAG	NM_000541.5	c.182-2A>G	Splice	Splice	Pathogenic	F	16	Black - Other
15521582	3497	SAG	NM_000541.5	c.182-2A>G	Splice	Splice	Pathogenic	F	16	Black - Other
8369787	3498	ATXN7	NM_001377405.1	Expansion	Expansion	Expansion	NA	M	77	White - British
10395594	3499	SDCCAG8	NM_00664.2	c.1357-9A>G	Splice	Splice	Variant of Uncertain Significance	F	30	Not stated
10395594	3499	SDCCAG8	NM_00664.2	c.1420del	p.(Glu474SerfsTer20)	Frameshift	Pathogenic	F	30	Not stated
16306499	3500	SLC24A1	NM_004727.3	c.754_755del	p.(Met252ValfsTer2)	Frameshift	Pathogenic	M	29	White - British
16306499	3500	SLC24A1	NM_004727.3	c.754_755del	p.(Met252ValfsTer2)	Frameshift	Pathogenic	M	29	White - British
10026505	3501	SLC24A5	NM_205850.3	c.568_572del	p.(Ile190Ter)	Stopgain	Likely Pathogenic	M	18	Asian - Pakistani
10026505	3501	SLC24A5	NM_205850.3	c.568_572del	p.(Ile190Ter)	Stopgain	Likely Pathogenic	M	18	Asian - Pakistani
9441865	3501	SLC24A5	NM_205850.3	c.568_572del	p.(Ile190Ter)	Stopgain	Likely Pathogenic	F	21	Asian - Pakistani
9441865	3501	SLC24A5	NM_205850.3	c.568_572del	p.(Ile190Ter)	Stopgain	Likely Pathogenic	F	21	Asian - Pakistani
17711707	3502	SLC24A5	NM_205850.3	c.1361T>A	p.(Leu454Ter)	Stopgain	Likely Pathogenic	F	29	White - British
17711707	3502	SLC24A5	NM_205850.3	c.989G>A	p.(Trp330Ter)	Stopgain	Likely Pathogenic	F	29	White - British
17976769	3503	SLC24A5	NM_205850.3	c.1315del	p.(Thr439LeufsTer3)	Frameshift	Likely Pathogenic	M	22	Not stated
17976769	3503	SLC24A5	NM_205850.3	c.514_515del	p.(Leu172IlefsTer10)	Frameshift	Likely Pathogenic	M	22	Not stated
2595242	3504	SLC25A46	NM_138773.4	c.371G>A	p.(Arg124His)	Missense	Variant of Uncertain Significance	M	65	White - British
2595242	3504	SLC25A46	NM_138773.4	c.746G>A	p.(Gly249Asp)	Missense	Likely Pathogenic	M	65	White - British
8178302	3505	SLC38A8	NM_001080442.3	c.264C>G	p.(Tyr88Ter)	Stopgain	Likely Pathogenic	M	19	Asian - Indian
8178302	3505	SLC38A8	NM_001080442.3	c.264C>G	p.(Tyr88Ter)	Stopgain	Likely Pathogenic	M	19	Asian - Indian
2715369	3505	SLC38A8	NM_001080442.3	c.264C>G	p.(Tyr88Ter)	Stopgain	Likely Pathogenic	F	35	Asian - Indian
2715369	3505	SLC38A8	NM_001080442.3	c.264C>G	p.(Tyr88Ter)	Stopgain	Likely Pathogenic	F	35	Asian - Indian

15970849	3506	SLC38A8	NM_001080442.3	c.435G>A	p.(Trp145Ter)	Stopgain	Pathogenic	F	8	White - British
15970849	3506	SLC38A8	NM_001080442.3	c.632+1G>A	Splice	Splice	Pathogenic	F	8	White - British
17844322	3506	SLC38A8	NM_001080442.3	c.435G>A	p.(Trp145Ter)	Stopgain	Pathogenic	M	5	White - British
17844322	3506	SLC38A8	NM_001080442.3	c.632+1G>A	Splice	Splice	Pathogenic	M	5	White - British
17624599	3507	SLC38A8	NM_001080442.3	c.264C>G	p.(Tyr88Ter)	Stopgain	Likely Pathogenic	M	5	Not stated
17624599	3507	SLC38A8	NM_001080442.3	c.264C>G	p.(Tyr88Ter)	Stopgain	Likely Pathogenic	M	5	Not stated
2665690	3508	SNRNP200	NM_014014.5	c.3260C>T	p.(Ser1087Leu)	Missense	Pathogenic	M	60	White - British
18459020	3508	SNRNP200	NM_014014.5	c.3260C>T	p.(Ser1087Leu)	Missense	Pathogenic	M	30	Any other ethnic group
7092945	3509	SNRNP200	NM_014014.5	c.2042G>A	p.(Arg681His)	Missense	Likely Pathogenic	M	43	Mixed - Other
11366361	3509	SNRNP200	NM_014014.5	c.2042G>A	p.(Arg681His)	Missense	Likely Pathogenic	M	78	Any other ethnic group
4165342	3510	SNRNP200	NM_014014.5	c.3260C>T	p.(Ser1087Leu)	Missense	Pathogenic	F	56	White - British
1179261	3511	SNRNP200	NM_014014.5	c.2047G>T	p.(Val683Leu)	Missense	Likely Pathogenic	F	76	Not stated
10880813	3512	SNRNP200	NM_014014.5	c.3260C>T	p.(Ser1087Leu)	Missense	Pathogenic	F	52	White - British
11464865	3513	SNRNP200	NM_014014.5	c.3260C>T	p.(Ser1087Leu)	Missense	Pathogenic	F	64	Black - Caribbean
12406617	3514	SNRNP200	NM_014014.5	c.2042G>A	p.(Arg681His)	Missense	Likely Pathogenic	F	50	Unknown
13403725	3515	SNRNP200	NM_014014.5	c.2042G>A	p.(Arg681His)	Missense	Likely Pathogenic	F	37	White - British
11495112	3516	SNRNP200	NM_014014.5	c.3260C>T	p.(Ser1087Leu)	Missense	Pathogenic	F	45	Any other ethnic group
13831033	3517	SNRNP200	NM_014014.5	c.2359G>A	p.(Ala787Thr)	Missense	Pathogenic	M	43	Any other ethnic group
15834447	3518	SNRNP200	NM_014014.5	c.1519G>A	p.(Ala507Thr)	Missense	Variant of Uncertain Significance	F	33	Not stated
16916353	3519	SNRNP200	NM_014014.5	c.3260C>T	p.(Ser1087Leu)	Missense	Pathogenic	M	42	White - British
4940970	3520	SRD5A3	NM_02459.5	c.57G>A	p.(Trp19Ter)	Stopgain	Pathogenic	M	31	Asian - Indian
4940970	3520	SRD5A3	NM_02459.5	c.57G>A	p.(Trp19Ter)	Stopgain	Pathogenic	M	31	Asian - Indian
562869	3521	SSBP1	NM_001256510.1	c.320G>A	p.(Arg107Gln)	Missense	Likely Pathogenic	F	61	White - British
5904653	3522	TIMP3	NM_000362.5	c.610A>T	p.(Ser204Cys)	Missense	Likely Pathogenic	F	73	White - British
5472214	3523	TIMP3	NM_000362.5	c.610A>T	p.(Ser204Cys)	Missense	Likely Pathogenic	M	66	Not stated
4746601	3524	TIMP3	NM_000362.5	c.610A>T	p.(Ser204Cys)	Missense	Likely Pathogenic	F	73	White - British
3059265	3524	TIMP3	NM_000362.5	c.610A>T	p.(Ser204Cys)	Missense	Likely Pathogenic	F	53	White - British
2344691	3524	TIMP3	NM_000362.5	c.610A>T	p.(Ser204Cys)	Missense	Likely Pathogenic	F	65	White - British
1427845	3524	TIMP3	NM_000362.5	c.610A>T	p.(Ser204Cys)	Missense	Likely Pathogenic	M	54	White - British
4830069	3525	TIMP3	NM_000362.5	c.610A>T	p.(Ser204Cys)	Missense	Likely Pathogenic	F	50	Not stated
3490185	3525	TIMP3	NM_000362.5	c.610A>T	p.(Ser204Cys)	Missense	Likely Pathogenic	M	78	White - British
16963071	3526	TIMP3	NM_000362.5	c.610A>T	p.(Ser204Cys)	Missense	Likely Pathogenic	F	41	Not stated
15085363	3527	TIMP3	NM_000362.5	c.610A>T	p.(Ser204Cys)	Missense	Likely Pathogenic	F	59	White - British
11726056	3528	TIMP3	NM_000362.5	c.610A>T	p.(Ser204Cys)	Missense	Likely Pathogenic	F	56	Unknown
2983322	3528	TIMP3	NM_000362.5	c.610A>T	p.(Ser204Cys)	Missense	Likely Pathogenic	F	80	Not stated
13980175	3528	TIMP3	NM_000362.5	c.610A>T	p.(Ser204Cys)	Missense	Likely Pathogenic	F	57	Not stated
5534542	3529	TIMP3	NM_000362.5	c.610A>T	p.(Ser204Cys)	Missense	Likely Pathogenic	F	46	White - British
5583647	3530	TIMP3	NM_000362.5	c.610A>T	p.(Ser204Cys)	Missense	Likely Pathogenic	M	64	White - British
13514199	3531	TIMP3	NM_000362.5	c.610A>T	p.(Ser204Cys)	Missense	Likely Pathogenic	F	59	Any other ethnic group
9487456	3531	TIMP3	NM_000362.5	c.610A>T	p.(Ser204Cys)	Missense	Likely Pathogenic	M	43	Not stated
6134099	3531	TIMP3	NM_000362.5	c.610A>T	p.(Ser204Cys)	Missense	Likely Pathogenic	M	72	Not stated
3881380	3532	TIMP3	NM_000362.5	c.610A>T	p.(Ser204Cys)	Missense	Likely Pathogenic	M	67	White - British
9425835	3533	TIMP3	NM_000362.5	c.610A>T	p.(Ser204Cys)	Missense	Likely Pathogenic	M	66	White - British
6831026	3533	TIMP3	NM_000362.5	c.610A>T	p.(Ser204Cys)	Missense	Likely Pathogenic	F	58	White - British
6912240	3534	TIMP3	NM_000362.5	c.610A>T	p.(Ser204Cys)	Missense	Likely Pathogenic	M	51	White - British
12379247	3535	TIMP3	NM_000362.5	c.610A>T	p.(Ser204Cys)	Missense	Likely Pathogenic	M	66	Not stated
3824554	3535	TIMP3	NM_000362.5	c.610A>T	p.(Ser204Cys)	Missense	Likely Pathogenic	M	58	White - British
3679199	3535	TIMP3	NM_000362.5	c.610A>T	p.(Ser204Cys)	Missense	Likely Pathogenic	M	53	White - British
18115747	3535	TIMP3	NM_000362.5	c.610A>T	p.(Ser204Cys)	Missense	Likely Pathogenic	M	27	Unknown
9194443	3536	TIMP3	NM_000362.5	c.610A>T	p.(Ser204Cys)	Missense	Likely Pathogenic	F	65	White - British
9328360	3537	TIMP3	NM_000362.5	c.610A>T	p.(Ser204Cys)	Missense	Likely Pathogenic	M	65	Not stated

10483920	3538	TIMP3	NM_000362.5	c.610A>T	p.(Ser204Cys)	Missense	Likely Pathogenic	F	47	White - British
14741474	3538	TIMP3	NM_000362.5	c.610A>T	p.(Ser204Cys)	Missense	Likely Pathogenic	F	45	White - British
414826	3539	TIMP3	NM_000362.5	c.610A>T	p.(Ser204Cys)	Missense	Likely Pathogenic	F	76	Not stated
10767616	3539	TIMP3	NM_000362.5	c.610A>T	p.(Ser204Cys)	Missense	Likely Pathogenic	F	52	White - British
6535017	3540	TIMP3	NM_000362.5	c.34G>C	p.(Gly12Arg)	Missense	Variant of Uncertain Significance	M	72	Not stated
11979589	3540	TIMP3	NM_000362.5	c.34G>C	p.(Gly12Arg)	Missense	Variant of Uncertain Significance	M	39	Unknown
12814430	3541	TIMP3	NM_000362.5	c.489T>A	p.(Cys163Ter)	Stopgain	Variant of Uncertain Significance	F	54	Unknown
12868890	3542	TIMP3	NM_000362.5	c.610A>T	p.(Ser204cys)	Missense	Likely Pathogenic	F	55	White - British
18642609	3542	TIMP3	NM_000362.5	c.610A>T	p.(Ser204Cys)	Missense	Likely Pathogenic	F	29	Unknown
6704102	3543	TIMP3	NM_000362.5	c.610A>T	p.(Ser204Cys)	Missense	Likely Pathogenic	M	63	Not stated
13813841	3544	TIMP3	NM_000362.5	c.610A>T	p.(Ser204Cys)	Missense	Likely Pathogenic	F	50	White - British
13593439	3545	TIMP3	NM_000362.5	c.545A>G	p.(Tyr182Cys)	Missense	Variant of Uncertain Significance	F	56	Any other ethnic group
15386734	3546	TIMP3	NM_000362.5	c.610A>T	p.(Ser204Cys)	Missense	Likely Pathogenic	F	50	Not stated
17859386	3547	TIMP3	NM_000362.5	c.610A>T	p.(Ser204Cys)	Missense	Likely Pathogenic	F	50	Not stated
18199152	3548	TIMP3	NM_000362.5	c.610A>T	p.(Ser204Cys)	Missense	Likely Pathogenic	F	41	Not stated
17295802	3549	TIMP3	NM_000362.5	c.594G>C	p.(Trp198Cys)	Missense	Variant of Uncertain Significance	F	61	Any other ethnic group
3343962	3550	TOPORS	NM_005802.5	c.2489C>A	p.(Ser830Ter)	Stopgain	Likely Pathogenic	M	51	Not stated
5485031	3551	TOPORS	NM_005802.5	c.2581A>T	p.(Lys861Ter)	Stopgain	Likely Pathogenic	F	60	White - British
7534470	3552	TOPORS	NM_005802.5	c.2556_2557del	p.(Glu852AspfsTer20)	Frameshift	Pathogenic	F	55	Not stated
15876783	3552	TOPORS	NM_005802.5	c.2556_2557del	p.(Glu852AspfsTer20)	Frameshift	Pathogenic	F	21	White - British
12417936	3553	TOPORS	NM_005802.5	c.2569del	p.(Arg857GlyfsTer9)	Frameshift	Pathogenic	F	38	White - Other
18291335	3554	TOPORS	NM_005802.5	c.2551dup	p.(Arg851LysfsTer22)	Frameshift	Likely Pathogenic	F	69	White - British
18089959	3555	TOPORS	NM_005802.5	c.2554_2557del	p.(Glu852GlnfsTer13)	Frameshift	Pathogenic	F	34	Any other ethnic group
13929978	3556	TRNT1	NM_182916.3	c.193G>C	p.(Ala65Pro)	Missense	Variant of Uncertain Significance	F	41	Not stated
13929978	3556	TRNT1	NM_182916.3	c.488A>T	p.(Asp163Val)	Missense	Likely Pathogenic	F	41	Not stated
603602	3557	TRPM1	NM_002420.6	c.3105T>A	p.(Tyr1035Ter)	Stopgain	Pathogenic	F	51	Black - Caribbean
603602	3557	TRPM1	NM_002420.6	c.416del	p.(Gly139ValfsTer10)	Frameshift	Pathogenic	F	51	Black - Caribbean
11750598	3558	TRPM1	NM_002420.6	c.296T>C	p.(Leu99Pro)	Missense	Likely Pathogenic	M	15	White - British
11750598	3558	TRPM1	NM_002420.6	c.296T>C	p.(Leu99Pro)	Missense	Likely Pathogenic	M	15	White - British
11751725	3559	TRPM1	NM_002420.6	c.3089G>A	p.(Cys1030Tyr)	Missense	Likely Pathogenic	F	12	Asian - Bangladeshi
11751725	3559	TRPM1	NM_002420.6	c.3089G>A	p.(Cys1030Tyr)	Missense	Likely Pathogenic	F	12	Asian - Bangladeshi
5712426	3560	TRPM1	NM_002420.6	c.552+3_552+6del	Splice	Splice	Likely Pathogenic	M	27	Asian - Indian
5712426	3560	TRPM1	NM_002420.6	c.552+3_552+6del	Splice	Splice	Likely Pathogenic	M	27	Asian - Indian
16449082	3561	TRPM1	NM_002420.6	c.1600G>A	p.(Gly534Arg)	Missense	Variant of Uncertain Significance	F	9	Not stated
16449082	3561	TRPM1	NM_002420.6	c.3061+1G>A	Splice	Splice	Pathogenic	F	9	Not stated
13055909	3562	TRPM1	NM_002420.6	c.2795G>A	p.(Cys932Tyr)	Missense	Variant of Uncertain Significance	M	23	Any other ethnic group
13055909	3562	TRPM1	NM_002420.6	c.2822G>A	p.(Arg941His)	Missense	Likely Pathogenic	M	23	Any other ethnic group
12471164	3563	TSPAN12	NM_012338.4	c.225_227del	p.(Ile76del)	Inframe indel	Likely Pathogenic	F	24	Any other ethnic group
16004925	3564	TSPAN12	NM_012338.4	c.68T>G	p.(Leu23Ter)	Stopgain	Likely Pathogenic	F	38	White - British
10857300	3565	TTLL5	NM_015072.5	c.1586_1589del	p.(Glu529ValfsTer2)	Frameshift	Pathogenic	M	50	Not stated
10857300	3565	TTLL5	NM_015072.5	c.1586_1589del	p.(Glu529ValfsTer2)	Frameshift	Pathogenic	M	50	Not stated
10306799	3566	TTLL5	NM_015072.5	c.1920G>A	p.(Trp640Ter)	Stopgain	Pathogenic	M	44	Not stated
10306799	3566	TTLL5	NM_015072.5	c.2457del	p.(Thr820LeufsTer25)	Frameshift	Likely Pathogenic	M	44	Not stated
10890760	3567	TTLL5	NM_015072.5	c.1627G>A	p.(Glu543Lys)	Missense	Likely Pathogenic	M	66	Asian - Indian
10890760	3567	TTLL5	NM_015072.5	c.1627G>A	p.(Glu543Lys)	Missense	Likely Pathogenic	M	66	Asian - Indian
11088314	3568	TTLL5	NM_015072.5	c.3354G>A	p.(Trp1118Ter)	Stopgain	Pathogenic	M	58	Asian - Other
11088314	3568	TTLL5	NM_015072.5	c.401del	p.(Leu134ArgfsTer45)	Frameshift	Pathogenic	M	58	Asian - Other
16081757	3569	TTLL5	NM_015072.5	c.1994T>C	p.(Leu665Pro)	Missense	Variant of Uncertain Significance	M	45	Not stated
16081757	3569	TTLL5	NM_015072.5	c.1994T>C	p.(Leu665Pro)	Missense	Variant of Uncertain Significance	M	45	Not stated
16081757	3569	TTLL5	NM_015072.5	c.2000del	p.(Asp667ValfsTer12)	Frameshift	Likely Pathogenic	M	45	Not stated
16081757	3569	TTLL5	NM_015072.5	c.2000del	p.(Asp667ValfsTer12)	Frameshift	Likely Pathogenic	M	45	Not stated

16594941	3570	TTLL5	NM_015072.5	c.1462A>G	p.(Thr488Ala)	Missense	Variant of Uncertain Significance	M	45	Asian - Bangladeshi
16594941	3570	TTLL5	NM_015072.5	c.1627G>A	p.(Glu543Lys)	Missense	Likely Pathogenic	M	45	Asian - Bangladeshi
12084127	3571	TTLL5	NM_015072.5	c.1627G>A	p.(Glu543Lys)	Missense	Likely Pathogenic	M	70	Not stated
12084127	3571	TTLL5	NM_015072.5	c.1627G>A	p.(Glu543Lys)	Missense	Likely Pathogenic	M	70	Not stated
11939185	3572	TTLL5	NM_015072.5	c.1474T>G	p.(Trp492Gly)	Missense	Variant of Uncertain Significance	F	38	Black - African
11939185	3572	TTLL5	NM_015072.5	c.1474T>G	p.(Trp492Gly)	Missense	Variant of Uncertain Significance	F	38	Black - African
599808	3573	TULP1	NM_003322.6	c.1389_1390delinsGC	p.(Asn463_Lys464delinsLysGln)	Inframe indel	Variant of Uncertain Significance	M	57	White - British
599808	3573	TULP1	NM_003322.6	c.932G>A	p.(Arg311Gln)	Missense	Pathogenic	M	57	White - British
3653117	3574	TULP1	NM_003322.6	c.1313G>C	p.(Arg438Pro)	Missense	Likely Pathogenic	F	55	Asian - Pakistani
3653117	3574	TULP1	NM_003322.6	c.1313G>C	p.(Arg438Pro)	Missense	Likely Pathogenic	F	55	Asian - Pakistani
9212349	3575	TULP1	NM_003322.6	c.1066C>A	p.(Pro356Thr)	Missense	Variant of Uncertain Significance	F	32	White - British
9212349	3575	TULP1	NM_003322.6	c.823_826del	p.(Lys275ArgfsTer34)	Frameshift	Pathogenic	F	32	White - British
9451504	3576	TULP1	NM_003322.6	c.751G>T	p.(Glu251Ter)	Stopgain	Likely Pathogenic	F	37	White - British
9451504	3576	TULP1	NM_003322.6	c.751G>T	p.(Glu251Ter)	Stopgain	Likely Pathogenic	F	37	White - British
9305253	3577	TULP1	NM_003322.6	c.1035C>G	p.(Ser345Arg)	Missense	Variant of Uncertain Significance	F	35	Not stated
9305253	3577	TULP1	NM_003322.6	c.1035C>G	p.(Ser345Arg)	Missense	Variant of Uncertain Significance	F	35	Not stated
10516974	3578	TULP1	NM_003322.6	c.1047T>G	p.(Asn349Lys)	Missense	Likely Pathogenic	M	24	White - British
10516974	3578	TULP1	NM_003322.6	c.1047T>G	p.(Asn349Lys)	Missense	Likely Pathogenic	M	24	White - British
16058083	3579	TULP1	NM_003322.6	c.1394C>G	p.(Pro465Arg)	Missense	Variant of Uncertain Significance	F	36	Any other ethnic group
16058083	3579	TULP1	NM_003322.6	c.1394C>G	p.(Pro465Arg)	Missense	Variant of Uncertain Significance	F	36	Any other ethnic group
13187677	3580	TULP1	NM_003322.6	c.1466A>G	p.(Lys489Arg)	Missense	Pathogenic	M	23	Unknown
13187677	3580	TULP1	NM_003322.6	c.1466A>G	p.(Lys489Arg)	Missense	Pathogenic	M	23	Unknown
3189297	3581	TULP1	NM_003322.6	c.1496-6C>A	Splice	Splice	Pathogenic	M	69	White - British
3189297	3581	TULP1	NM_003322.6	c.1496-6C>A	Splice	Splice	Pathogenic	M	69	White - British
15026269	3582	TULP1	NM_003322.6	c.1453C>T	p.(Gln485Ter)	Stopgain	Likely Pathogenic	F	39	White - British
15026269	3582	TULP1	NM_003322.6	c.1609G>A	p.(Gly537Arg)	Missense	Variant of Uncertain Significance	F	39	White - British
15400958	3583	TULP1	NM_003322.6	c.901del	p.(Gln301ArgfsTer9)	Frameshift	Likely Pathogenic	F	8	Not stated
15400958	3583	TULP1	NM_003322.6	Exon 11-13 duplication	Duplication	Duplication	Likely Pathogenic	F	8	Not stated
16673124	3584	TULP1	NM_003322.6	c.629C>G	p.(Ser210Ter)	Stopgain	Pathogenic	F	24	Asian - Indian
16673124	3584	TULP1	NM_003322.6	c.629C>G	p.(Ser210Ter)	Stopgain	Pathogenic	F	24	Asian - Indian
17806249	3585	TULP1	NM_003322.6	c.1047T>G	p.(Asn349Lys)	Missense	Likely Pathogenic	M	45	Asian - Pakistani
17806249	3585	TULP1	NM_003322.6	c.1047T>G	p.(Asn349Lys)	Missense	Likely Pathogenic	M	45	Asian - Pakistani
9203382	3586	TULP1	NM_003322.6	c.1047T>G	p.(Asn349Lys)	Missense	Likely Pathogenic	F	33	Asian - Other
9203382	3586	TULP1	NM_003322.6	c.1047T>G	p.(Asn349Lys)	Missense	Likely Pathogenic	F	33	Asian - Other
2142510	3587	USH1C	NM_005709.4	c.238dup	p.(Arg80ProfsTer69)	Frameshift	Pathogenic	M	62	Not stated
2142510	3587	USH1C	NM_005709.4	c.238dup	p.(Arg80ProfsTer69)	Frameshift	Pathogenic	M	62	Not stated
4237960	3588	USH1C	NM_005709.4	c.319G>T	p.(Glu107Ter)	Stopgain	Likely Pathogenic	F	43	Not stated
4237960	3588	USH1C	NM_005709.4	c.319G>T	p.(Glu107Ter)	Stopgain	Likely Pathogenic	F	43	Not stated
2854200	3589	USH1C	NM_005709.4	c.446_448del	p.(Glu149del)	Inframe indel	Likely Pathogenic	F	58	Not stated
2854200	3589	USH1C	NM_005709.4	c.446_448del	p.(Glu149del)	Inframe indel	Likely Pathogenic	F	58	Not stated
6204680	3590	USH1C	NM_005709.4	c.238dup	p.(Arg80ProfsTer69)	Frameshift	Pathogenic	F	44	White - British
6204680	3590	USH1C	NM_005709.4	c.496+1G>A	Splice	Splice	Pathogenic	F	44	White - British
1093665	3591	USH1C	NM_005709.4	c.238dup	p.(Arg80ProfsTer69)	Frameshift	Pathogenic	F	41	Not stated
1093665	3591	USH1C	NM_005709.4	c.238dup	p.(Arg80ProfsTer69)	Frameshift	Pathogenic	F	41	Not stated
8586738	3592	USH1C	NM_005709.4	c.1327-1G>T	Splice	Splice	Pathogenic	M	58	Not stated
8586738	3592	USH1C	NM_005709.4	c.308G>A	p.(Arg103His)	Missense	Likely Pathogenic	M	58	Not stated
8997379	3592	USH1C	NM_005709.4	c.1327-1G>T	Splice	Splice	Pathogenic	F	60	Not stated
8997379	3592	USH1C	NM_005709.4	c.308G>A	p.(Arg103His)	Missense	Likely Pathogenic	F	60	Not stated
11353187	3593	USH1C	NM_005709.4	c.238dup	p.(Arg80ProfsTer69)	Frameshift	Pathogenic	M	39	Unknown
11353187	3593	USH1C	NM_005709.4	c.238dup	p.(Arg80ProfsTer69)	Frameshift	Pathogenic	M	39	Unknown
11193090	3593	USH1C	NM_005709.4	c.238dup	p.(Arg80ProfsTer69)	Frameshift	Pathogenic	M	32	Any other ethnic group

11193090	3593	USH1C	NM_005709.4	c.238dup	p.(Arg80ProfsTer69)	Frameshift	Pathogenic	M	32	Any other ethnic group
9088778	3594	USH1C	NM_005709.4	c.496+1G>A	Splice	Splice	Pathogenic	F	54	White - British
9088778	3594	USH1C	NM_005709.4	c.496+1G>A	Splice	Splice	Pathogenic	F	54	White - British
9169306	3595	USH1C	NM_005709.4	c.496+1G>A	Splice	Splice	Pathogenic	M	53	Not stated
9169306	3595	USH1C	NM_005709.4	c.496+1G>A	Splice	Splice	Pathogenic	M	53	Not stated
9220609	3596	USH1C	NM_005709.4	c.496+1G>A	Splice	Splice	Pathogenic	F	32	White - British
9220609	3596	USH1C	NM_005709.4	c.496+1G>A	Splice	Splice	Pathogenic	F	32	White - British
9235694	3597	USH1C	NM_005709.4	c.496+1G>A	Splice	Splice	Pathogenic	M	36	White - British
9235694	3597	USH1C	NM_005709.4	c.496+1G>A	Splice	Splice	Pathogenic	M	36	White - British
9882473	3598	USH1C	NM_005709.4	c.238dup	p.(Arg80ProfsTer69)	Frameshift	Pathogenic	F	38	White - British
9882473	3598	USH1C	NM_005709.4	c.1501G>T	p.(Glu501Ter)	Stopgain	Pathogenic	F	38	White - British
12942040	3599	USH1C	NM_005709.4	c.238dup	p.(Arg80ProfsTer69)	Frameshift	Pathogenic	M	29	Not stated
12942040	3599	USH1C	NM_005709.4	c.238dup	p.(Arg80ProfsTer69)	Frameshift	Pathogenic	M	29	Not stated
6561036	3600	USH1C	NM_005709.4	c.748_759+5del	Deletion	Deletion	Pathogenic	M	81	White - British
6561036	3600	USH1C	NM_005709.4	c.748_759+5del	Deletion	Deletion	Pathogenic	M	81	White - British
6573692	3601	USH1C	NM_005709.4	c.748_759+5del	Deletion	Deletion	Pathogenic	M	35	White - British
6573692	3601	USH1C	NM_005709.4	c.748_759+5del	Deletion	Deletion	Pathogenic	M	35	White - British
17690273	3602	USH1C	NM_005709.4	c.248+1G>A	Splice	Splice	Pathogenic	F	35	Not stated
17690273	3602	USH1C	NM_005709.4	c.248+1G>A	Splice	Splice	Pathogenic	F	35	Not stated
18526290	3603	USH1C	NM_005709.4	c.238dup	p.(Arg80ProfsTer69)	Frameshift	Pathogenic	M	42	White - Other
18526290	3603	USH1C	NM_005709.4	c.238dup	p.(Arg80ProfsTer69)	Frameshift	Pathogenic	M	42	White - Other
16143126	3604	USH1C	NM_005709.4	c.248+1G>A	Splice	Splice	Pathogenic	M	23	Asian - Pakistani
16143126	3604	USH1C	NM_005709.4	c.248+1G>A	Splice	Splice	Pathogenic	M	23	Asian - Pakistani
6992943	3605	USH1G	NM_173477.5	c.203C>A	p.(Pro68His)	Missense	Variant of Uncertain Significance	M	32	Any other ethnic group
6992943	3605	USH1G	NM_173477.5	c.387dup	p.(Lys130GlnfsTer5)	Frameshift	Pathogenic	M	32	Any other ethnic group
8615669	3606	USH1G	NM_173477.5	c.1240G>T	p.(Asp414Tyr)	Missense	Variant of Uncertain Significance	F	46	Not stated
8615669	3606	USH1G	NM_173477.5	c.1240G>T	p.(Asp414Tyr)	Missense	Variant of Uncertain Significance	F	46	Not stated
8605435	3607	USH2A	NM_206933.4	c.10561T>C	p.(Trp3521Arg)	Missense	Pathogenic	M	74	White - British
8605435	3607	USH2A	NM_206933.4	c.9459C>A	p.(Cys3153Ter)	Stopgain	Pathogenic	M	74	White - British
742447	3608	USH2A	NM_206933.4	c.1055C>T	p.(Thr352Ile)	Missense	Likely Pathogenic	F	70	Not stated
742447	3608	USH2A	NM_206933.4	c.12819T>A	p.(Tyr4273Ter)	Stopgain	Pathogenic	F	70	Not stated
1075157	3609	USH2A	NM_206933.4	c.920_923dup	p.(His308GlnfsTer16)	Frameshift	Pathogenic	M	72	White - British
1075157	3609	USH2A	NM_206933.4	c.9371+1G>C	Splice	Splice	Pathogenic	M	72	White - British
13582400	3610	USH2A	NM_206933.4	c.10342G>A	p.(Glu3448Lys)	Missense	Likely Pathogenic	M	79	Unknown
13582400	3610	USH2A	NM_206933.4	c.2299del	p.(Glu767SerfsTer21)	Frameshift	Pathogenic	M	79	Unknown
4598740	3611	USH2A	NM_206933.4	c.2299del	p.(Glu767SerfsTer21)	Frameshift	Pathogenic	M	65	White - British
4598740	3611	USH2A	NM_206933.4	c.2299del	p.(Glu767SerfsTer21)	Frameshift	Pathogenic	M	65	White - British
7629278	3612	USH2A	NM_206933.4	c.12295-3T>A	Splice	Splice	Pathogenic	F	76	Not stated
7629278	3612	USH2A	NM_206933.4	c.2299del	p.(Glu767SerfsTer21)	Frameshift	Pathogenic	F	76	Not stated
2110429	3613	USH2A	NM_206933.4	c.12093C>A	p.(Tyr4031Ter)	Stopgain	Pathogenic	M	66	Not stated
2110429	3613	USH2A	NM_206933.4	c.12295-3T>A	Splice	Splice	Pathogenic	M	66	Not stated
2111549	3614	USH2A	NM_206933.4	c.1256G>T	p.(Cys419Phe)	Missense	Likely Pathogenic	M	60	White - British
2111549	3614	USH2A	NM_206933.4	c.4510dup	p.(Arg1504LysfsTer26)	Frameshift	Pathogenic	M	60	White - British
1418451	3615	USH2A	NM_206933.4	c.1019A>T	p.(His340Leu)	Missense	Variant of Uncertain Significance	F	84	White - British
1418451	3615	USH2A	NM_206933.4	c.1679del	p.(Pro560LeufsTer31)	Frameshift	Pathogenic	F	84	White - British
7274714	3616	USH2A	NM_206933.4	c.236_239dup	p.(Gln81TyrfsTer28)	Frameshift	Pathogenic	M	66	Mixed - Other
7274714	3616	USH2A	NM_206933.4	c.236_239dup	p.(Gln81TyrfsTer28)	Frameshift	Pathogenic	M	66	Mixed - Other
10802721	3617	USH2A	NM_206933.4	c.10073G>A	p.(Cys3358Tyr)	Missense	Pathogenic	M	73	White - British
10802721	3617	USH2A	NM_206933.4	c.7789A>T	p.(Lys2597Ter)	Stopgain	Pathogenic	M	73	White - British
7128617	3618	USH2A	NM_206933.4	c.10561T>C	p.(Trp3521Arg)	Missense	Pathogenic	F	75	White - British
7128617	3618	USH2A	NM_206933.4	c.2299del	p.(Glu767SerfsTer21)	Frameshift	Pathogenic	F	75	White - British

392664	3619	USH2A	NM_206933.4	c.10561T>C	p.(Trp3521Arg)	Missense	Pathogenic	F	75	Not stated
392664	3619	USH2A	NM_206933.4	c.9882C>G	p.(Cys3294Trp)	Missense	Likely Pathogenic	F	75	Not stated
3987668	3620	USH2A	NM_206933.4	c.4251+1G>T	Splice	Splice	Pathogenic	F	70	Asian - Indian
3987668	3620	USH2A	NM_206933.4	c.5012G>A	p.(Gly1671Asp)	Missense	Likely Pathogenic	F	70	Asian - Indian
8375457	3621	USH2A	NM_206933.4	c.4510dup	p.(Arg1504LysfsTer26)	Frameshift	Pathogenic	F	60	Not stated
8375457	3621	USH2A	NM_206933.4	c.8231G>A	p.(Trp2744Ter)	Stopgain	Pathogenic	F	60	Not stated
701728	3622	USH2A	NM_206933.4	c.2299del	p.(Glu767SerfsTer21)	Frameshift	Pathogenic	F	75	White - British
701728	3622	USH2A	NM_206933.4	c.6928A>C	p.(Thr2310Pro)	Missense	Variant of Uncertain Significance	F	75	White - British
6250040	3623	USH2A	NM_206933.4	c.14426C>T	p.(Thr4809Ile)	Missense	Pathogenic	F	64	Not stated
6250040	3623	USH2A	NM_206933.4	c.2299del	p.(Glu767SerfsTer21)	Frameshift	Pathogenic	F	64	Not stated
840237	3624	USH2A	NM_206933.4	c.12295-3T>A	Splice	Splice	Pathogenic	F	63	Not stated
840237	3624	USH2A	NM_206933.4	c.2299del	p.(Glu767SerfsTer21)	Frameshift	Pathogenic	F	63	Not stated
8956079	3625	USH2A	NM_206933.4	c.10585G>A	p.(Gly3529Ser)	Missense	Likely Pathogenic	M	66	Not stated
8956079	3625	USH2A	NM_206933.4	c.2299del	p.(Glu767SerfsTer21)	Frameshift	Pathogenic	M	66	Not stated
1620296	3626	USH2A	NM_206933.4	c.2299del	p.(Glu767SerfsTer21)	Frameshift	Pathogenic	M	83	White - British
1620296	3626	USH2A	NM_206933.4	c.8261del	p.(Ile2754AsnfsTer15)	Frameshift	Likely Pathogenic	M	83	White - British
1380385	3627	USH2A	NM_206933.4	c.2236C>G	p.(Pro746Ala)	Missense	Variant of Uncertain Significance	M	60	White - British
1380385	3627	USH2A	NM_206933.4	c.7595-3C>G	Splice	Splice	Pathogenic	M	60	White - British
12991005	3628	USH2A	NM_206933.4	c.2299del	p.(Glu767SerfsTer21)	Frameshift	Pathogenic	M	41	Not stated
12991005	3628	USH2A	NM_206933.4	c.6104G>A	p.(Cys2035Tyr)	Missense	Variant of Uncertain Significance	M	41	Not stated
8168103	3629	USH2A	NM_206933.4	c.2299del	p.(Glu767SerfsTer21)	Frameshift	Pathogenic	F	52	White - British
8168103	3629	USH2A	NM_206933.4	c.3518C>A	p.(Ser1173Ter)	Stopgain	Likely Pathogenic	F	52	White - British
491602	3630	USH2A	NM_206933.4	c.13374del	p.(Glu4458AspfsTer3)	Frameshift	Pathogenic	M	65	Any other ethnic group
491602	3630	USH2A	NM_206933.4	c.7595-3C>G	Splice	Splice	Pathogenic	M	65	Any other ethnic group
1022720	3631	USH2A	NM_206933.4	c.2299del	p.(Glu767SerfsTer21)	Frameshift	Pathogenic	F	53	Any other ethnic group
1022720	3631	USH2A	NM_206933.4	c.820C>G	p.(Arg274Gly)	Missense	Likely Pathogenic	F	53	Any other ethnic group
3577447	3632	USH2A	NM_206933.4	c.11390-1G>C	Splice	Splice	Likely Pathogenic	M	63	White - British
3577447	3632	USH2A	NM_206933.4	c.2299del	p.(Glu767SerfsTer21)	Frameshift	Pathogenic	M	63	White - British
4437488	3633	USH2A	NM_206933.4	c.1036A>C	p.(Asn346His)	Missense	Pathogenic	M	73	Not stated
4437488	3633	USH2A	NM_206933.4	c.10561T>C	p.(Trp3521Arg)	Missense	Pathogenic	M	73	Not stated
1535393	3634	USH2A	NM_206933.4	c.10073G>A	p.(Cys3358Tyr)	Missense	Pathogenic	F	70	White - British
1535393	3634	USH2A	NM_206933.4	c.2276G>T	p.(Cys759Phe)	Missense	Pathogenic	F	70	White - British
2798977	3635	USH2A	NM_206933.4	c.12234_12235del	p.(Asn4079TrpfsTer19)	Frameshift	Pathogenic	F	52	Unknown
2798977	3635	USH2A	NM_206933.4	c.12739G>A	p.(Gly4247Arg)	Missense	Variant of Uncertain Significance	F	52	Unknown
3236120	3636	USH2A	NM_206933.4	c.11156G>A	p.(Arg3719His)	Missense	Pathogenic	F	59	White - British
3236120	3636	USH2A	NM_206933.4	c.920_923dup	p.(His308GlnfsTer16)	Frameshift	Pathogenic	F	59	White - British
9991141	3637	USH2A	NM_206933.4	c.12457G>A	p.(Ala4153Thr)	Missense	Variant of Uncertain Significance	F	71	White - British
9991141	3637	USH2A	NM_206933.4	c.2299del	p.(Glu767SerfsTer21)	Frameshift	Pathogenic	F	71	White - British
8760002	3638	USH2A	NM_206933.4	c.3840_3841delinsCT	p.(Met1280_1281delinsIleTer)	Stopgain	Pathogenic	F	71	Not stated
8760002	3638	USH2A	NM_206933.4	c.908G>A	p.(Arg303His)	Missense	Pathogenic	F	71	Not stated
877393	3639	USH2A	NM_206933.4	c.13621C>T	p.(Gln4541Ter)	Stopgain	Pathogenic	M	64	White - British
877393	3639	USH2A	NM_206933.4	c.2299del	p.(Glu767SerfsTer21)	Frameshift	Pathogenic	M	64	White - British
1953209	3640	USH2A	NM_206933.4	c.10073G>A	p.(Cys3358Tyr)	Missense	Pathogenic	F	64	White - British
1953209	3640	USH2A	NM_206933.4	c.6131C>A	p.(Ser2044Ter)	Stopgain	Pathogenic	F	64	White - British
8979452	3641	USH2A	NM_206933.4	c.11875_11876del	p.(Gln3959AsnfsTer53)	Frameshift	Pathogenic	F	76	White - British
8979452	3641	USH2A	NM_206933.4	c.2299del	p.(Glu767SerfsTer21)	Frameshift	Pathogenic	F	76	White - British
6805476	3642	USH2A	NM_206933.4	c.1679del	p.(Pro560LeufsTer31)	Frameshift	Pathogenic	M	53	White - British
6805476	3642	USH2A	NM_206933.4	c.6795_6797del	p.(Glu2265_Tyr2266delinsAsp)	Inframe indel	Likely Pathogenic	M	53	White - British
3651199	3643	USH2A	NM_206933.4	Exon 47 deletion	Deletion	Deletion	Likely Pathogenic	M	82	Unknown
3651199	3643	USH2A	NM_206933.4	Exon 47 deletion	Deletion	Deletion	Likely Pathogenic	M	82	Unknown
7770762	3644	USH2A	NM_206933.4	c.2299del	p.(Glu767SerfsTer21)	Frameshift	Pathogenic	F	63	White - British

7770762	3644	USH2A	NM_206933.4	c.4133T>C	p.(Leu1378Pro)	Missense	Likely Pathogenic	F	63	White - British
3648056	3645	USH2A	NM_206933.4	c.5012G>A	p.(Gly1671Asp)	Missense	Likely Pathogenic	F	70	Asian - Indian
3648056	3645	USH2A	NM_206933.4	c.5012G>A	p.(Gly1671Asp)	Missense	Likely Pathogenic	F	70	Asian - Indian
3646166	3646	USH2A	NM_206933.4	c.2299del	p.(Glu767SerfsTer21)	Frameshift	Pathogenic	F	66	White - British
3646166	3646	USH2A	NM_206933.4	c.2299del	p.(Glu767SerfsTer21)	Frameshift	Pathogenic	F	66	White - British
3894498	3647	USH2A	NM_206933.4	c.2299del	p.(Glu767SerfsTer21)	Frameshift	Pathogenic	F	47	Not stated
3894498	3647	USH2A	NM_206933.4	c.7595-2144A>G	Splice	Splice	Pathogenic	F	47	Not stated
3889906	3648	USH2A	NM_206933.4	c.13316C>T	p.(Thr4439Ile)	Missense	Pathogenic	M	43	Not stated
3889906	3648	USH2A	NM_206933.4	c.9799T>C	p.(Cys3267Arg)	Missense	Pathogenic	M	43	Not stated
4062183	3649	USH2A	NM_206933.4	c.2299del	p.(Glu767SerfsTer21)	Frameshift	Pathogenic	M	77	Not stated
4062183	3649	USH2A	NM_206933.4	c.2994_3007del	p.(Cys999LeufsTer9)	Frameshift	Likely Pathogenic	M	77	Not stated
4099101	3650	USH2A	NM_206933.4	c.11389+2dup	Splice	Splice	Likely Pathogenic	M	46	White - British
4099101	3650	USH2A	NM_206933.4	c.14426C>T	p.(Thr4809Ile)	Missense	Pathogenic	M	46	White - British
4346047	3650	USH2A	NM_206933.4	c.11389+2dup	Splice	Splice	Likely Pathogenic	F	47	White - British
4346047	3650	USH2A	NM_206933.4	c.14426C>T	p.(Thr4809Ile)	Missense	Pathogenic	F	47	White - British
4094348	3651	USH2A	NM_206933.4	c.13316C>T	p.(Thr4439Ile)	Missense	Pathogenic	M	62	Not stated
4094348	3651	USH2A	NM_206933.4	c.2299del	p.(Glu767SerfsTer21)	Frameshift	Pathogenic	M	62	Not stated
4192446	3652	USH2A	NM_206933.4	c.1859G>T	p.(Cys620Phe)	Missense	Pathogenic	F	61	White - British
4192446	3652	USH2A	NM_206933.4	c.4821G>A	p.(Trp1607Ter)	Stopgain	Pathogenic	F	61	White - British
4271959	3653	USH2A	NM_206933.4	c.9815C>T	p.(Pro3272Leu)	Missense	Likely Pathogenic	F	53	Asian - Indian
4271959	3653	USH2A	NM_206933.4	c.9815C>T	p.(Pro3272Leu)	Missense	Likely Pathogenic	F	53	Asian - Indian
4403902	3654	USH2A	NM_206933.4	c.14446A>T	p.(Lys4816Ter)	Stopgain	Likely Pathogenic	M	52	Not stated
4403902	3654	USH2A	NM_206933.4	c.2299del	p.(Glu767SerfsTer21)	Frameshift	Pathogenic	M	52	Not stated
4547976	3655	USH2A	NM_206933.4	c.2023C>T	p.(Gly675Ter)	Stopgain	Pathogenic	F	58	White - British
4547976	3655	USH2A	NM_206933.4	c.6159del	p.(Glu2054LysfsTer10)	Frameshift	Pathogenic	F	58	White - British
4871670	3656	USH2A	NM_206933.4	c.2299del	p.(Glu767SerfsTer21)	Frameshift	Pathogenic	M	46	Not stated
4871670	3656	USH2A	NM_206933.4	c.2299del	p.(Glu767SerfsTer21)	Frameshift	Pathogenic	M	46	Not stated
4922196	3657	USH2A	NM_206933.4	c.13374del	p.(Glu4458AspfsTer3)	Frameshift	Pathogenic	F	77	Not stated
4922196	3657	USH2A	NM_206933.4	c.2299del	p.(Glu767SerfsTer21)	Frameshift	Pathogenic	F	77	Not stated
4892446	3658	USH2A	NM_206933.4	c.12574C>T	p.(Arg4192Cys)	Missense	Likely Pathogenic	M	67	Not stated
4892446	3658	USH2A	NM_206933.4	c.13316C>T	p.(Thr4439Ile)	Missense	Pathogenic	M	67	Not stated
5861680	3659	USH2A	NM_206933.4	c.2299del	p.(Glu767SerfsTer21)	Frameshift	Pathogenic	M	44	Not stated
5861680	3659	USH2A	NM_206933.4	c.820C>G	p.(Arg274Gly)	Missense	Likely Pathogenic	M	44	Not stated
5060075	3660	USH2A	NM_206933.4	c.1036A>C	p.(Asn346His)	Missense	Pathogenic	M	58	White - British
5060075	3660	USH2A	NM_206933.4	c.13316C>T	p.(Thr4439Ile)	Missense	Pathogenic	M	58	White - British
5220746	3661	USH2A	NM_206933.4	c.10561T>C	p.(Trp3521Arg)	Missense	Pathogenic	M	57	Not stated
5220746	3661	USH2A	NM_206933.4	c.11065C>T	p.(Arg3689Ter)	Stopgain	Pathogenic	M	57	Not stated
7402408	3662	USH2A	NM_206933.4	c.6862G>T	p.(Glu2288Ter)	Stopgain	Pathogenic	F	58	Not stated
7402408	3662	USH2A	NM_206933.4	c.802G>A	p.(Gly268Arg)	Missense	Likely Pathogenic	F	58	Not stated
5460146	3663	USH2A	NM_206933.4	c.2299del	p.(Glu767SerfsTer21)	Frameshift	Pathogenic	M	48	Not stated
5460146	3663	USH2A	NM_206933.4	c.6862G>T	p.(Glu2288Ter)	Stopgain	Pathogenic	M	48	Not stated
7274994	3664	USH2A	NM_206933.4	c.1841-2A>G	Splice	Splice	Pathogenic	F	62	White - British
7274994	3664	USH2A	NM_206933.4	c.1841-2A>G	Splice	Splice	Pathogenic	F	62	White - British
5479102	3665	USH2A	NM_206933.4	c.13010C>T	p.(Thr4337Met)	Missense	Pathogenic	M	54	Not stated
5479102	3665	USH2A	NM_206933.4	c.2276G>T	p.(Cys759Phe)	Missense	Pathogenic	M	54	Not stated
5545721	3666	USH2A	NM_206933.4	c.10073G>A	p.(Cys3358Tyr)	Missense	Pathogenic	M	65	White - British
5545721	3666	USH2A	NM_206933.4	c.11156G>A	p.(Arg3719His)	Missense	Pathogenic	M	65	White - British
1548028	3667	USH2A	NM_206933.4	c.2299del	p.(Glu767SerfsTer21)	Frameshift	Pathogenic	F	49	White - British
1548028	3667	USH2A	NM_206933.4	c.2299del	p.(Glu767SerfsTer21)	Frameshift	Pathogenic	F	49	White - British
5632500	3668	USH2A	NM_206933.4	c.7595-2144A>G	Splice	Splice	Pathogenic	F	53	Asian - Other
5632500	3668	USH2A	NM_206933.4	c.7595-2144A>G	Splice	Splice	Pathogenic	F	53	Asian - Other

6134036	3669	USH2A	NM_206933.4	c.4354dup	p.(Cys1452LeufsTer25)	Frameshift	Likely Pathogenic	M	55	Asian - Pakistani
6134036	3669	USH2A	NM_206933.4	c.4354dup	p.(Cys1452LeufsTer25)	Frameshift	Likely Pathogenic	M	55	Asian - Pakistani
5918030	3670	USH2A	NM_206933.4	c.2299del	p.(Glu767SerfsTer21)	Frameshift	Pathogenic	M	56	Not stated
5918030	3670	USH2A	NM_206933.4	c.2299del	p.(Glu767SerfsTer21)	Frameshift	Pathogenic	M	56	Not stated
5935369	3671	USH2A	NM_206933.4	c.12575G>A	p.(Arg4192His)	Missense	Pathogenic	M	56	White - British
5935369	3671	USH2A	NM_206933.4	c.8079G>A	p.(Trp2693Ter)	Stopgain	Pathogenic	M	56	White - British
5948984	3672	USH2A	NM_206933.4	c.2299del	p.(Glu767SerfsTer21)	Frameshift	Pathogenic	M	42	Not stated
5948984	3672	USH2A	NM_206933.4	c.5614_5620del	p.(Ala1872LeufsTer64)	Frameshift	Likely Pathogenic	M	42	Not stated
5948991	3672	USH2A	NM_206933.4	c.2299del	p.(Glu767SerfsTer21)	Frameshift	Pathogenic	M	43	White - British
5948991	3672	USH2A	NM_206933.4	c.5614_5620del	p.(Ala1872LeufsTer64)	Frameshift	Likely Pathogenic	M	43	White - British
5952995	3673	USH2A	NM_206933.4	c.11709C>G	p.(Tyr3903Ter)	Stopgain	Pathogenic	M	47	Asian - Pakistani
5952995	3673	USH2A	NM_206933.4	c.11709C>G	p.(Tyr3903Ter)	Stopgain	Pathogenic	M	47	Asian - Pakistani
5492304	3674	USH2A	NM_206933.4	c.5012G>A	p.(Gly1671Asp)	Missense	Likely Pathogenic	F	66	Asian - Indian
5492304	3674	USH2A	NM_206933.4	c.5012G>A	p.(Gly1671Asp)	Missense	Likely Pathogenic	F	66	Asian - Indian
3053259	3675	USH2A	NM_206933.4	c.187C>T	p.(Arg63Ter)	Stopgain	Pathogenic	F	64	Black - Caribbean
3053259	3675	USH2A	NM_206933.4	c.2081G>A	p.(Cys694Tyr)	Missense	Pathogenic	F	64	Black - Caribbean
7338652	3675	USH2A	NM_206933.4	c.187C>T	p.(Arg63Ter)	Stopgain	Pathogenic	M	55	Not stated
7338652	3675	USH2A	NM_206933.4	c.2081G>A	p.(Cys694Tyr)	Missense	Pathogenic	M	55	Not stated
5837411	3676	USH2A	NM_206933.4	c.3724C>T	p.(Pro1242Ser)	Missense	Variant of Uncertain Significance	F	53	Not stated
5837411	3676	USH2A	NM_206933.4	c.3724C>T	p.(Pro1242Ser)	Missense	Variant of Uncertain Significance	F	53	Not stated
8310791	3677	USH2A	NM_206933.4	c.14426C>T	p.(Thr4809Ile)	Missense	Pathogenic	F	61	Not stated
8310791	3677	USH2A	NM_206933.4	c.2276G>T	p.(Cys759Phe)	Missense	Pathogenic	F	61	Not stated
6394688	3677	USH2A	NM_206933.4	c.14426C>T	p.(Thr4809Ile)	Missense	Pathogenic	F	66	Not stated
6394688	3677	USH2A	NM_206933.4	c.2276G>T	p.(Cys759Phe)	Missense	Pathogenic	F	66	Not stated
7861685	3678	USH2A	NM_206933.4	c.10073G>A	p.(Cys3358Tyr)	Missense	Pathogenic	M	74	White - British
7861685	3678	USH2A	NM_206933.4	c.2276G>T	p.(Cys759Phe)	Missense	Pathogenic	M	74	White - British
6306285	3679	USH2A	NM_206933.4	c.5012G>A	p.(Gly1671Asp)	Missense	Likely Pathogenic	M	49	Asian - Indian
6306285	3679	USH2A	NM_206933.4	c.5012G>A	p.(Gly1671Asp)	Missense	Likely Pathogenic	M	49	Asian - Indian
6294021	3679	USH2A	NM_206933.4	c.5012G>A	p.(Gly1671Asp)	Missense	Likely Pathogenic	F	48	Asian - Bangladeshi
6294021	3679	USH2A	NM_206933.4	c.5012G>A	p.(Gly1671Asp)	Missense	Likely Pathogenic	F	48	Asian - Bangladeshi
5753033	3679	USH2A	NM_206933.4	c.5012G>A	p.(Gly1671Asp)	Missense	Likely Pathogenic	M	51	Asian - Indian
5753033	3679	USH2A	NM_206933.4	c.5012G>A	p.(Gly1671Asp)	Missense	Likely Pathogenic	M	51	Asian - Indian
7570268	3680	USH2A	NM_206933.4	c.12954C>A	p.(Tyr4318Ter)	Stopgain	Pathogenic	M	52	White - British
7570268	3680	USH2A	NM_206933.4	c.920_923dup	p.(His308GlnfsTer16)	Frameshift	Pathogenic	M	52	White - British
6315224	3680	USH2A	NM_206933.4	c.12954C>A	p.(Tyr4318Ter)	Stopgain	Pathogenic	M	56	White - British
6315224	3680	USH2A	NM_206933.4	c.920_923dup	p.(His308GlnfsTer16)	Frameshift	Pathogenic	M	56	White - British
6315224	3680	USH2A	NM_206933.4	c.13870T>G	p.(Phe4624Val)	Missense	Variant of Uncertain Significance	M	56	White - British
1860256	3681	USH2A	NM_206933.4	c.100C>T	p.(Arg34Ter)	Stopgain	Pathogenic	M	61	Not stated
1860256	3681	USH2A	NM_206933.4	c.926C>T	p.(Pro309Leu)	Missense	Likely Pathogenic	M	61	Not stated
6248724	3682	USH2A	NM_206933.4	c.2276G>T	p.(Cys759Phe)	Missense	Pathogenic	F	74	White - British
6248724	3682	USH2A	NM_206933.4	c.2299del	p.(Glu767SerfsTer21)	Frameshift	Pathogenic	F	74	White - British
5854085	3683	USH2A	NM_206933.4	c.5012G>A	p.(Gly1671Asp)	Missense	Likely Pathogenic	M	65	Asian - Indian
5854085	3683	USH2A	NM_206933.4	c.5012G>A	p.(Gly1671Asp)	Missense	Likely Pathogenic	M	65	Asian - Indian
3589774	3684	USH2A	NM_206933.4	c.14911C>T	p.(Arg4971Ter)	Stopgain	Pathogenic	M	44	White - British
3589774	3684	USH2A	NM_206933.4	c.2299del	p.(Glu767SerfsTer21)	Frameshift	Pathogenic	M	44	White - British
3630395	3685	USH2A	NM_206933.4	c.14980C>T	p.(Gln4994Ter)	Stopgain	Likely Pathogenic	M	63	Black - Caribbean
3630395	3685	USH2A	NM_206933.4	c.7120+1G>A	Splice	Splice	Pathogenic	M	63	Black - Caribbean
1477937	3685	USH2A	NM_206933.4	c.14980C>T	p.(Gln4994Ter)	Stopgain	Likely Pathogenic	M	66	Black - Caribbean
1477937	3685	USH2A	NM_206933.4	c.7120+1G>A	Splice	Splice	Pathogenic	M	66	Black - Caribbean
6684922	3686	USH2A	NM_206933.4	c.14219C>A	p.(Ala4740Asp)	Missense	Variant of Uncertain Significance	F	57	White - British
6684922	3686	USH2A	NM_206933.4	c.1606T>C	p.(Cys536Arg)	Missense	Pathogenic	F	57	White - British

5581848	3687	USH2A	NM_206933.4	c.13126T>G	p.(Trp4376Gly)	Missense	Likely Pathogenic	M	53	Not stated
5581848	3687	USH2A	NM_206933.4	c.13331C>T	p.(Pro4444Leu)	Missense	Pathogenic	M	53	Not stated
6012838	3688	USH2A	NM_206933.4	c.10073G>A	p.(Cys3358Tyr)	Missense	Pathogenic	F	67	White - British
6012838	3688	USH2A	NM_206933.4	c.12575G>A	p.(Arg4192His)	Missense	Pathogenic	F	67	White - British
6778127	3688	USH2A	NM_206933.4	c.10073G>A	p.(Cys3358Tyr)	Missense	Pathogenic	M	70	White - British
6778127	3688	USH2A	NM_206933.4	c.12575G>A	p.(Arg4192His)	Missense	Pathogenic	M	70	White - British
6766990	3689	USH2A	NM_206933.4	c.2299del	p.(Glu767SerfsTer21)	Frameshift	Pathogenic	M	63	White - British
6766990	3689	USH2A	NM_206933.4	c.651+1G>A	Splice	Splice	Pathogenic	M	63	White - British
6840497	3690	USH2A	NM_206933.4	c.2299del	p.(Glu767SerfsTer21)	Frameshift	Pathogenic	M	33	White - Other
6840497	3690	USH2A	NM_206933.4	c.3187_3188del	p.(Gln1063SerfsTer15)	Frameshift	Pathogenic	M	33	White - Other
6860461	3691	USH2A	NM_206933.4	c.2299del	p.(Glu767SerfsTer21)	Frameshift	Pathogenic	M	38	White - British
6860461	3691	USH2A	NM_206933.4	Exon 4 deletion	Deletion	Deletion	Likely Pathogenic	M	38	White - British
6599123	3692	USH2A	NM_206933.4	c.12095G>T	p.(Gly4032Val)	Missense	Variant of Uncertain Significance	F	77	White - British
6599123	3692	USH2A	NM_206933.4	c.6470del	p.(Arg2157LysfsTer13)	Frameshift	Pathogenic	F	77	White - British
6894418	3693	USH2A	NM_206933.4	c.1876C>T	p.(Arg626Ter)	Stopgain	Pathogenic	M	43	White - British
6894418	3693	USH2A	NM_206933.4	c.2299del	p.(Glu767SerfsTer21)	Frameshift	Pathogenic	M	43	White - British
6264075	3694	USH2A	NM_206933.4	c.1606T>A	p.(Cys536Ser)	Missense	Likely Pathogenic	F	47	White - British
6264075	3694	USH2A	NM_206933.4	c.2299del	p.(Glu767SerfsTer21)	Frameshift	Pathogenic	F	47	White - British
6994868	3695	USH2A	NM_206933.4	c.13010C>T	p.(Thr4337Met)	Missense	Pathogenic	M	64	White - British
6994868	3695	USH2A	NM_206933.4	c.13895C>A	p.(Pro4632His)	Missense	Variant of Uncertain Significance	M	64	White - British
6995092	3696	USH2A	NM_206933.4	c.13316C>T	p.(Thr4439Ile)	Missense	Pathogenic	M	54	Any other ethnic group
6995092	3696	USH2A	NM_206933.4	c.2276G>T	p.(Cys759Phe)	Missense	Pathogenic	M	54	Any other ethnic group
6792897	3697	USH2A	NM_206933.4	c.2610C>A	p.(Cys870Ter)	Stopgain	Pathogenic	F	38	Any other ethnic group
6792897	3697	USH2A	NM_206933.4	c.2610C>A	p.(Cys870Ter)	Stopgain	Pathogenic	F	38	Any other ethnic group
7139586	3697	USH2A	NM_206933.4	c.2610C>A	p.(Cys870Ter)	Stopgain	Pathogenic	M	35	White - British
7139586	3697	USH2A	NM_206933.4	c.2610C>A	p.(Cys870Ter)	Stopgain	Pathogenic	M	35	White - British
6810348	3698	USH2A	NM_206933.4	c.2299del	p.(Glu767SerfsTer21)	Frameshift	Pathogenic	M	47	White - British
6810348	3698	USH2A	NM_206933.4	c.4510dup	p.(Arg1504LysfsTer26)	Frameshift	Pathogenic	M	47	White - British
7035930	3699	USH2A	NM_206933.4	c.4354dup	p.(Cys1452LeufsTer25)	Frameshift	Likely Pathogenic	F	69	Black - Caribbean
7035930	3699	USH2A	NM_206933.4	Exon 6 deletion	Deletion	Deletion	Variant of Uncertain Significance	F	69	Black - Caribbean
7067731	3700	USH2A	NM_206933.4	c.14403C>G	p.(Tyr4801Ter)	Stopgain	Likely Pathogenic	F	53	White - British
7067731	3700	USH2A	NM_206933.4	c.2299del	p.(Glu767SerfsTer21)	Frameshift	Pathogenic	F	53	White - British
7214255	3701	USH2A	NM_206933.4	c.13207_13208del	p.(Gly4403ProfsTer15)	Frameshift	Pathogenic	M	37	Mixed - White and Asian
7214255	3701	USH2A	NM_206933.4	c.4761del	p.(Ser1588HisfsTer5)	Frameshift	Likely Pathogenic	M	37	Mixed - White and Asian
7590470	3702	USH2A	NM_206933.4	c.2299del	p.(Glu767SerfsTer21)	Frameshift	Pathogenic	M	55	White - British
7590470	3702	USH2A	NM_206933.4	c.920_923dup	p.(His308GlnfsTer16)	Frameshift	Pathogenic	M	55	White - British
7379462	3703	USH2A	NM_206933.4	c.7595-3C>G	Splice	Splice	Pathogenic	M	63	Not stated
7379462	3703	USH2A	NM_206933.4	c.8546G>T	p.(Gly2849Val)	Missense	Variant of Uncertain Significance	M	63	Not stated
3411197	3704	USH2A	NM_206933.4	c.6862G>T	p.(Glu2288Ter)	Stopgain	Pathogenic	F	47	Not stated
3411197	3704	USH2A	NM_206933.4	Exon 40 deletion	Deletion	Deletion	Likely Pathogenic	F	47	Not stated
7230474	3705	USH2A	NM_206933.4	c.842C>A	p.(Thr281Lys)	Missense	Variant of Uncertain Significance	F	55	White - Other
7230474	3705	USH2A	NM_206933.4	c.842C>A	p.(Thr281Lys)	Missense	Variant of Uncertain Significance	F	55	White - Other
2751825	3706	USH2A	NM_206933.4	c.5329C>T	p.(Arg1777Trp)	Missense	Likely Pathogenic	M	70	Asian - Indian
2751825	3706	USH2A	NM_206933.4	c.6854A>G	p.(Asn2285Ser)	Missense	Variant of Uncertain Significance	M	70	Asian - Indian
7505574	3707	USH2A	NM_206933.4	c.12575G>A	p.(Arg4192His)	Missense	Pathogenic	F	64	Not stated
7505574	3707	USH2A	NM_206933.4	c.12575G>A	p.(Arg4192His)	Missense	Pathogenic	F	64	Not stated
7505574	3707	USH2A	NM_206933.4	c.15322del	p.(Arg5108GlyfsTer6)	Frameshift	Pathogenic	F	64	Not stated
7505574	3707	USH2A	NM_206933.4	c.15322del	p.(Arg5108GlyfsTer6)	Frameshift	Pathogenic	F	64	Not stated
7666973	3708	USH2A	NM_206933.4	c.2299del	p.(Glu767SerfsTer21)	Frameshift	Pathogenic	M	49	Not stated
7666973	3708	USH2A	NM_206933.4	Exon 27 deletion	Deletion	Deletion	Likely Pathogenic	M	49	Not stated
7818880	3709	USH2A	NM_206933.4	c.2299del	p.(Glu767SerfsTer21)	Frameshift	Pathogenic	F	52	Not stated

7818880	3709	USH2A	NM_206933.4	c.5836C>T	p.(Arg1946Ter)	Stopgain	Pathogenic	F	52	Not stated
7906807	3710	USH2A	NM_206933.4	c.12739G>A	p.(Gly4247Arg)	Missense	Variant of Uncertain Significance	M	65	White - British
7906807	3710	USH2A	NM_206933.4	c.13207_13208del	p.(Gly4403ProfsTer15)	Frameshift	Pathogenic	M	65	White - British
7203342	3711	USH2A	NM_206933.4	c.10073G>A	p.(Cys3358Tyr)	Missense	Pathogenic	F	44	White - British
7203342	3711	USH2A	NM_206933.4	c.920_923dup	p.(His308GlnfsTer16)	Frameshift	Pathogenic	F	44	White - British
8011485	3711	USH2A	NM_206933.4	c.10073G>A	p.(Cys3358Tyr)	Missense	Pathogenic	F	45	White - British
8011485	3711	USH2A	NM_206933.4	c.920_923dup	p.(His308GlnfsTer16)	Frameshift	Pathogenic	F	45	White - British
8025982	3712	USH2A	NM_206933.4	c.10073G>A	p.(Cys3358Tyr)	Missense	Pathogenic	F	68	Not stated
8025982	3712	USH2A	NM_206933.4	c.2276G>T	p.(Cys759Phe)	Missense	Pathogenic	F	68	Not stated
8117815	3713	USH2A	NM_206933.4	c.2299del	p.(Glu767SerfsTer21)	Frameshift	Pathogenic	M	61	White - British
8117815	3713	USH2A	NM_206933.4	c.8834G>A	p.(Trp2945Ter)	Stopgain	Pathogenic	M	61	White - British
5997746	3714	USH2A	NM_206933.4	c.10342G>A	p.(Glu3448Lys)	Missense	Likely Pathogenic	F	54	White - Other
5997746	3714	USH2A	NM_206933.4	c.2081G>A	p.(Cys694Tyr)	Missense	Pathogenic	F	54	White - Other
5997746	3714	USH2A	NM_206933.4	c.6670G>T	p.(Gly2224Cys)	Missense	Variant of Uncertain Significance	F	54	White - Other
8892701	3715	USH2A	NM_206933.4	c.2299del	p.(Glu767SerfsTer21)	Frameshift	Pathogenic	M	32	White - British
8892701	3715	USH2A	NM_206933.4	c.3407G>A	p.(Ser1136Asn)	Missense	Pathogenic	M	32	White - British
9299324	3716	USH2A	NM_206933.4	c.10073G>A	p.(Cys3358Tyr)	Missense	Pathogenic	M	41	Not stated
9299324	3716	USH2A	NM_206933.4	c.14191G>A	p.(Gly4731Arg)	Missense	Variant of Uncertain Significance	M	41	Not stated
9299324	3716	USH2A	NM_206933.4	c.920_923dup	p.(His308GlnfsTer16)	Frameshift	Pathogenic	M	41	Not stated
503117	3717	USH2A	NM_206933.4	c.1859G>T	p.(Cys620Phe)	Missense	Pathogenic	F	73	Not stated
503117	3717	USH2A	NM_206933.4	c.667dup	p.(Ile223AsnfsTer100)	Frameshift	Pathogenic	F	73	Not stated
9432968	3718	USH2A	NM_206933.4	c.4027A>C	p.(Asn1343His)	Missense	Likely Pathogenic	F	65	Not stated
9432968	3718	USH2A	NM_206933.4	c.4027A>C	p.(Asn1343His)	Missense	Likely Pathogenic	F	65	Not stated
9321738	3719	USH2A	NM_206933.4	c.10626del	p.(Glu3542AspfsTer8)	Frameshift	Pathogenic	F	58	White - British
9321738	3719	USH2A	NM_206933.4	c.4106C>T	p.(Ser1369Leu)	Missense	Likely Pathogenic	F	58	White - British
4610290	3720	USH2A	NM_206933.4	c.2299del	p.(Glu767SerfsTer21)	Frameshift	Pathogenic	M	47	Any other ethnic group
4610290	3720	USH2A	NM_206933.4	c.2299del	p.(Glu767SerfsTer21)	Frameshift	Pathogenic	M	47	Any other ethnic group
1106174	3721	USH2A	NM_206933.4	Exon 52 deletion	Deletion	Deletion	Likely Pathogenic	F	53	White - British
1106174	3721	USH2A	NM_206933.4	c.920_923dup	p.(His308GlnfsTer16)	Frameshift	Pathogenic	F	53	White - British
9598952	3722	USH2A	NM_206933.4	c.10073G>A	p.(Cys3358Tyr)	Missense	Pathogenic	M	53	Not stated
9598952	3722	USH2A	NM_206933.4	c.10342G>A	p.(Glu3448Lys)	Missense	Likely Pathogenic	M	53	Not stated
9598952	3722	USH2A	NM_206933.4	c.6670G>T	p.(Gly2224Cys)	Missense	Variant of Uncertain Significance	M	53	Not stated
9862740	3723	USH2A	NM_206933.4	c.12695C>A	p.(Pro4232Gln)	Missense	Variant of Uncertain Significance	M	56	Not stated
9862740	3723	USH2A	NM_206933.4	c.8851C>G	p.(Gln2951Glu)	Missense	Variant of Uncertain Significance	M	56	Not stated
9862740	3723	USH2A	NM_206933.4	c.8884C>A	p.(Leu2962Ile)	Missense	Variant of Uncertain Significance	M	56	Not stated
9906154	3724	USH2A	NM_206933.4	c.10699del	p.(Leu3567Ter)	Stopgain	Pathogenic	F	64	White - British
9906154	3724	USH2A	NM_206933.4	c.10699del	p.(Leu3567Ter)	Stopgain	Pathogenic	F	64	White - British
9580871	3725	USH2A	NM_206933.4	c.1679del	p.(Pro560LeufsTer31)	Frameshift	Pathogenic	M	46	White - British
9580871	3725	USH2A	NM_206933.4	c.2276G>T	p.(Cys759Phe)	Missense	Pathogenic	M	46	White - British
9001642	3726	USH2A	NM_206933.4	c.2299del	p.(Glu767SerfsTer21)	Frameshift	Pathogenic	F	28	White - British
9001642	3726	USH2A	NM_206933.4	c.1256G>T	p.(Cys419Phe)	Missense	Likely Pathogenic	F	28	White - British
7766961	3727	USH2A	NM_206933.4	c.2276G>T	p.(Cys759Phe)	Missense	Pathogenic	M	60	Not stated
7766961	3727	USH2A	NM_206933.4	c.8740C>T	p.(Arg2914Ter)	Stopgain	Pathogenic	M	60	Not stated
7274231	3728	USH2A	NM_206933.4	c.5012G>A	p.(Gly1671Asp)	Missense	Likely Pathogenic	M	70	Asian - Pakistani
7274231	3728	USH2A	NM_206933.4	c.5012G>A	p.(Gly1671Asp)	Missense	Likely Pathogenic	M	70	Asian - Pakistani
8097046	3729	USH2A	NM_206933.4	c.13335_13347delinsCTTG	.(Glu4445_Ser4449delinsAspLeu	Inframe indel	Pathogenic	M	65	White - Other
8097046	3729	USH2A	NM_206933.4	c.7524del	p.(Arg2509GlyfsTer19)	Frameshift	Pathogenic	M	65	White - Other
6949340	3730	USH2A	NM_206933.4	c.1036A>C	p.(Asn346His)	Missense	Pathogenic	F	72	White - British
6949340	3730	USH2A	NM_206933.4	c.12575G>A	p.(Arg4192His)	Missense	Pathogenic	F	72	White - British
8869489	3731	USH2A	NM_206933.4	c.13010C>T	p.(Thr4337Met)	Missense	Pathogenic	M	32	Not stated
8869489	3731	USH2A	NM_206933.4	Exon 47 deletion	Deletion	Deletion	Likely Pathogenic	M	32	Not stated

9072986	3732	USH2A	NM_206933.4	Exon 50-55 deletion	Deletion	Deletion	Likely Pathogenic	M	36	Asian - Pakistani
9072986	3732	USH2A	NM_206933.4	Exon 50-55 deletion	Deletion	Deletion	Likely Pathogenic	M	36	Asian - Pakistani
10466077	3733	USH2A	NM_206933.4	c.4645C>T	p.(Arg1549Ter)	Stopgain	Pathogenic	M	53	Not stated
10466077	3733	USH2A	NM_206933.4	c.4645C>T	p.(Arg1549Ter)	Stopgain	Pathogenic	M	53	Not stated
9541944	3734	USH2A	NM_206933.4	c.2276G>T	p.(Cys759Phe)	Missense	Pathogenic	M	57	White - British
9541944	3734	USH2A	NM_206933.4	c.10561T>C	p.(Trp3521Arg)	Missense	Pathogenic	M	57	White - British
10283475	3735	USH2A	NM_206933.4	c.12575G>A	p.(Arg4192His)	Missense	Pathogenic	M	53	Not stated
10283475	3735	USH2A	NM_206933.4	c.2276G>T	p.(Cys759Phe)	Missense	Pathogenic	M	53	Not stated
10697063	3736	USH2A	NM_206933.4	c.11065C>T	p.(Arg3689Ter)	Stopgain	Pathogenic	M	28	White - British
10697063	3736	USH2A	NM_206933.4	c.7645_7661del	p.(Met2549IafsTer3)	Frameshift	Likely Pathogenic	M	28	White - British
5989290	3737	USH2A	NM_206933.4	c.2802T>G	p.(Cys934Trp)	Missense	Pathogenic	F	68	Any other ethnic group
5989290	3737	USH2A	NM_206933.4	c.8284C>G	p.(Pro2762Ala)	Missense	Likely Pathogenic	F	68	Any other ethnic group
5989290	3737	USH2A	NM_206933.4	c.9958G>T	p.(Gly3320Cys)	Missense	Likely Pathogenic	F	68	Any other ethnic group
5055735	3738	USH2A	NM_206933.4	c.10073G>A	p.(Cys3358Tyr)	Missense	Pathogenic	F	67	White - British
5055735	3738	USH2A	NM_206933.4	c.2299del	p.(Glu767SerfsTer21)	Frameshift	Pathogenic	F	67	White - British
8139564	3739	USH2A	NM_206933.4	c.2139C>T	p.(Gly713Gly)	Synonymous	Likely Pathogenic	M	52	White - British
8139564	3739	USH2A	NM_206933.4	c.4133T>C	p.(Leu1378Pro)	Missense	Likely Pathogenic	M	52	White - British
3619853	3740	USH2A	NM_206933.4	c.2705G>A	p.(Cys902Tyr)	Missense	Variant of Uncertain Significance	F	90	Any other ethnic group
3619853	3740	USH2A	NM_206933.4	c.5858C>G	p.(Ala1953Gly)	Missense	Variant of Uncertain Significance	F	90	Any other ethnic group
3619853	3740	USH2A	NM_206933.4	c.8320G>A	p.(Ala2774Thr)	Missense	Likely Benign	F	90	Any other ethnic group
7918728	3741	USH2A	NM_206933.4	c.12575G>A	p.(Arg4192His)	Missense	Pathogenic	M	75	White - British
7918728	3741	USH2A	NM_206933.4	c.3310C>A	p.(Pro1104Thr)	Missense	Variant of Uncertain Significance	M	75	White - British
10439484	3742	USH2A	NM_206933.4	c.9815C>T	p.(Pro3272Leu)	Missense	Likely Pathogenic	F	61	Asian - Indian
10439484	3742	USH2A	NM_206933.4	c.9815C>T	p.(Pro3272Leu)	Missense	Likely Pathogenic	F	61	Asian - Indian
3798584	3742	USH2A	NM_206933.4	c.9815C>T	p.(Pro3272Leu)	Missense	Likely Pathogenic	M	69	Asian - Indian
3798584	3742	USH2A	NM_206933.4	c.9815C>T	p.(Pro3272Leu)	Missense	Likely Pathogenic	M	69	Asian - Indian
11129033	3743	USH2A	NM_206933.4	c.2299del	p.(Glu767SerfsTer21)	Frameshift	Pathogenic	F	48	Not stated
11129033	3743	USH2A	NM_206933.4	c.4732C>T	p.(Arg1578Cys)	Missense	Pathogenic	F	48	Not stated
10890886	3744	USH2A	NM_206933.4	c.2299del	p.(Glu767SerfsTer21)	Frameshift	Pathogenic	F	55	White - British
10890886	3744	USH2A	NM_206933.4	c.7595-3C>G	Splice	Splice	Pathogenic	F	55	White - British
9604853	3745	USH2A	NM_206933.4	c.12309del	p.(Phe4103LeufsTer11)	Frameshift	Pathogenic	F	45	White - British
9604853	3745	USH2A	NM_206933.4	c.12309del	p.(Phe4103LeufsTer11)	Frameshift	Pathogenic	F	45	White - British
11024264	3746	USH2A	NM_206933.4	c.14285A>G	p.(Asn4762Ser)	Missense	Pathogenic	F	55	Asian - Indian
11024264	3746	USH2A	NM_206933.4	c.11156G>T	p.(Arg3719Leu)	Missense	Variant of Uncertain Significance	F	55	Asian - Indian
10726267	3747	USH2A	NM_206933.4	c.4106C>T	p.(Ser1369Leu)	Missense	Likely Pathogenic	F	68	White - British
10726267	3747	USH2A	NM_206933.4	c.4510dup	p.(Arg1504LysfsTer26)	Frameshift	Pathogenic	F	68	White - British
11086340	3748	USH2A	NM_206933.4	c.10073G>A	p.(Cys3358Tyr)	Missense	Pathogenic	F	54	White - British
11086340	3748	USH2A	NM_206933.4	c.11549-1G>A	Splice	Splice	Pathogenic	F	54	White - British
11086340	3748	USH2A	NM_206933.4	c.11927C>T	p.(Thr3976Met)	Missense	Variant of Uncertain Significance	F	54	White - British
10884201	3749	USH2A	NM_206933.4	c.2994A>T	p.(Arg998Ser)	Missense	Variant of Uncertain Significance	M	33	Black - Other
10884201	3749	USH2A	NM_206933.4	c.895del	p.(Gln299AsnfsTer37)	Frameshift	Pathogenic	M	33	Black - Other
16340036	3750	USH2A	NM_206933.4	c.11389+2dup	Splice	Splice	Likely Pathogenic	M	32	Black - African
16340036	3750	USH2A	NM_206933.4	c.11389+2dup	Splice	Splice	Likely Pathogenic	M	32	Black - African
11581695	3751	USH2A	NM_206933.4	c.10435_10436del	p.(Trp3479GlufsTer35)	Frameshift	Likely Pathogenic	M	62	White - British
11581695	3751	USH2A	NM_206933.4	c.2299del	p.(Glu767SerfsTer21)	Frameshift	Pathogenic	M	62	White - British
11114088	3752	USH2A	NM_206933.4	c.2081G>A	p.(Cys694Tyr)	Missense	Pathogenic	M	40	White - British
11114088	3752	USH2A	NM_206933.4	c.2299del	p.(Glu767SerfsTer21)	Frameshift	Pathogenic	M	40	White - British
11173742	3753	USH2A	NM_206933.4	c.10073G>A	p.(Cys3358Tyr)	Missense	Pathogenic	M	48	White - British
11173742	3753	USH2A	NM_206933.4	c.10342G>A	p.(Glu3448Lys)	Missense	Likely Pathogenic	M	48	White - British
11773509	3754	USH2A	NM_206933.4	c.1036A>C	p.(Asn346His)	Missense	Pathogenic	F	57	White - British
11773509	3754	USH2A	NM_206933.4	c.6795_6797del	p.(Glu2265_Tyr2266delinsAsp)	Inframe indel	Likely Pathogenic	F	57	White - British

6502166	3755	USH2A	NM_206933.4	c.1606T>C	p.(Cys536Arg)	Missense	Pathogenic	F	62	White - British
6502166	3755	USH2A	NM_206933.4	c.7412T>A	p.(Leu2471His)	Missense	Variant of Uncertain Significance	F	62	White - British
11486040	3756	USH2A	NM_206933.4	c.10073G>A	p.(Cys3358Tyr)	Missense	Pathogenic	M	52	Any other ethnic group
11486040	3756	USH2A	NM_206933.4	c.12954C>A	p.(Tyr4318Ter)	Stopgain	Pathogenic	M	52	Any other ethnic group
6675549	3757	USH2A	NM_206933.4	c.11700C>A	p.(Tyr3900Ter)	Stopgain	Pathogenic	M	52	White - Other
6675549	3757	USH2A	NM_206933.4	c.4618G>A	p.(Asp1540Asn)	Missense	Variant of Uncertain Significance	M	52	White - Other
11260892	3758	USH2A	NM_206933.4	c.13335_13347delinsCTTG	.(Glu4445_ser4449delinsAspLeu	Inframe indel	Pathogenic	F	47	Not stated
11260892	3758	USH2A	NM_206933.4	Exon 22-24 deletion	Deletion	Deletion	Likely Pathogenic	F	47	Not stated
1561167	3759	USH2A	NM_206933.4	c.2139C>T	p.(Gly713Gly)	Synonymous	Likely Pathogenic	F	80	White - Other
1561167	3759	USH2A	NM_206933.4	c.7595-2144A>G	Splice	Splice	Pathogenic	F	80	White - Other
11350681	3760	USH2A	NM_206933.4	c.10073G>A	p.(Cys3358Tyr)	Missense	Pathogenic	F	69	Any other ethnic group
11350681	3760	USH2A	NM_206933.4	c.2299del	p.(Glu767SerfsTer21)	Frameshift	Pathogenic	F	69	Any other ethnic group
8994446	3761	USH2A	NM_206933.4	c.2299del	p.(Glu767SerfsTer21)	Frameshift	Pathogenic	M	37	Not stated
8994446	3761	USH2A	NM_206933.4	c.5603T>G	p.(Phe1868Cys)	Missense	Likely Pathogenic	M	37	Not stated
12387283	3762	USH2A	NM_206933.4	c.13621C>T	p.(Gln4541Ter)	Stopgain	Pathogenic	M	86	Not stated
12387283	3762	USH2A	NM_206933.4	c.2023C>T	p.(Gly675Ter)	Stopgain	Pathogenic	M	86	Not stated
9178028	3763	USH2A	NM_206933.4	c.3187_3188del	p.(Gln1063SerfsTer15)	Frameshift	Pathogenic	F	54	Not stated
9178028	3763	USH2A	NM_206933.4	Exon 22-23 deletion	Deletion	Deletion	Likely Pathogenic	F	54	Not stated
7777146	3764	USH2A	NM_206933.4	c.2299del	p.(Glu767SerfsTer21)	Frameshift	Pathogenic	M	41	Not stated
7777146	3764	USH2A	NM_206933.4	c.2299del	p.(Glu767SerfsTer21)	Frameshift	Pathogenic	M	41	Not stated
8522002	3765	USH2A	NM_206933.4	c.1256G>T	p.(Cys419Phe)	Missense	Likely Pathogenic	M	35	Not stated
8522002	3765	USH2A	NM_206933.4	c.2299del	p.(Glu767SerfsTer21)	Frameshift	Pathogenic	M	35	Not stated
8697765	3766	USH2A	NM_206933.4	c.11047+1G>A	Splice	Splice	Pathogenic	M	59	Not stated
8697765	3766	USH2A	NM_206933.4	c.4474G>T	p.(Glu1492Ter)	Stopgain	Pathogenic	M	59	Not stated
8715524	3767	USH2A	NM_206933.4	c.11875_11876del	p.(Gln3959AsnfsTer53)	Frameshift	Pathogenic	F	57	White - British
8715524	3767	USH2A	NM_206933.4	c.14285A>G	p.(Asn4762Ser)	Missense	Pathogenic	F	57	White - British
11608246	3768	USH2A	NM_206933.4	c.6653T>C	p.(Leu2218Pro)	Missense	Variant of Uncertain Significance	M	47	Not stated
11608246	3768	USH2A	NM_206933.4	c.6854A>G	p.(Asn2285Ser)	Missense	Variant of Uncertain Significance	M	47	Not stated
11608246	3768	USH2A	NM_206933.4	c.6904_6920dup	p.(Gln2307HisfsTer25)	Frameshift	Pathogenic	M	47	Not stated
9222023	3769	USH2A	NM_206933.4	c.2299del	p.(Glu767SerfsTer21)	Frameshift	Pathogenic	M	78	White - British
9222023	3769	USH2A	NM_206933.4	c.7595-2144A>G	Splice	Splice	Pathogenic	M	78	White - British
8643578	3770	USH2A	NM_206933.4	c.1036A>C	p.(Asn346His)	Missense	Pathogenic	M	72	Not stated
8643578	3770	USH2A	NM_206933.4	c.9912dup	p.(Glu3305ArgfsTer41)	Frameshift	Pathogenic	M	72	Not stated
9152268	3771	USH2A	NM_206933.4	c.1036A>C	p.(Asn346His)	Missense	Pathogenic	M	68	White - British
9152268	3771	USH2A	NM_206933.4	c.1256G>T	p.(Cys419Phe)	Missense	Likely Pathogenic	M	68	White - British
7666735	3772	USH2A	NM_206933.4	c.100C>T	p.(Arg34Ter)	Stopgain	Pathogenic	F	73	Not stated
7666735	3772	USH2A	NM_206933.4	c.2299del	p.(Glu767SerfsTer21)	Frameshift	Pathogenic	F	73	Not stated
6374906	3773	USH2A	NM_206933.4	c.1256G>T	p.(Cys419Phe)	Missense	Likely Pathogenic	M	54	Not stated
6374906	3773	USH2A	NM_206933.4	c.2299del	p.(Glu767SerfsTer21)	Frameshift	Pathogenic	M	54	Not stated
10082610	3774	USH2A	NM_206933.4	c.1256G>T	p.(Cys419Phe)	Missense	Likely Pathogenic	F	43	Not stated
10082610	3774	USH2A	NM_206933.4	c.13130C>A	p.(Ser4377Ter)	Stopgain	Pathogenic	F	43	Not stated
8810171	3775	USH2A	NM_206933.4	c.13374del	p.(Glu4458AspfsTer3)	Frameshift	Pathogenic	F	84	Not stated
8810171	3775	USH2A	NM_206933.4	c.7595-3C>G	Splice	Splice	Pathogenic	F	84	Not stated
9173933	3776	USH2A	NM_206933.4	c.2299del	p.(Glu767SerfsTer21)	Frameshift	Pathogenic	M	45	White - British
9173933	3776	USH2A	NM_206933.4	c.852_853del	p.(Glu284AspfsTer38)	Frameshift	Pathogenic	M	45	White - British
8842798	3777	USH2A	NM_206933.4	c.187C>T	p.(Arg63Ter)	Stopgain	Pathogenic	M	35	Not stated
8842798	3777	USH2A	NM_206933.4	c.4645C>T	p.(Arg1549Ter)	Stopgain	Pathogenic	M	35	Not stated
8287852	3778	USH2A	NM_206933.4	c.2299del	p.(Glu767SerfsTer21)	Frameshift	Pathogenic	M	42	Not stated
8287852	3778	USH2A	NM_206933.4	Exon 4-13 duplication	Duplication	Duplication	Likely Pathogenic	M	42	Not stated
9008082	3779	USH2A	NM_206933.4	c.4732C>T	p.(Arg1578Cys)	Missense	Pathogenic	M	55	White - British
9008082	3779	USH2A	NM_206933.4	c.5899_5900del	p.(Asn1967TrpfsTer5)	Frameshift	Likely Pathogenic	M	55	White - British

7088983	3780	USH2A	NM_206933.4	c.14139G>A	p.(Trp4713Ter)	Stopgain	Likely Pathogenic	M	61	White - British
7088983	3780	USH2A	NM_206933.4	c.2299del	p.(Glu767SerfsTer21)	Frameshift	Pathogenic	M	61	White - British
6192563	3781	USH2A	NM_206933.4	c.187C>T	p.(Arg63Ter)	Stopgain	Pathogenic	M	75	White - British
6192563	3781	USH2A	NM_206933.4	Exon 70 deletion	Deletion	Deletion	Variant of Uncertain Significance	M	75	White - British
8328613	3782	USH2A	NM_206933.4	c.2023C>T	p.(Gly675Ter)	Stopgain	Pathogenic	F	41	Not stated
8328613	3782	USH2A	NM_206933.4	c.2299del	p.(Glu767SerfsTer21)	Frameshift	Pathogenic	F	41	Not stated
8395015	3783	USH2A	NM_206933.4	c.2299del	p.(Glu767SerfsTer21)	Frameshift	Pathogenic	F	38	Not stated
8395015	3783	USH2A	NM_206933.4	c.2299del	p.(Glu767SerfsTer21)	Frameshift	Pathogenic	F	38	Not stated
8771328	3784	USH2A	NM_206933.4	c.1256G>T	p.(Cys419Phe)	Missense	Likely Pathogenic	F	75	Not stated
8771328	3784	USH2A	NM_206933.4	c.1256G>T	p.(Cys419Phe)	Missense	Likely Pathogenic	F	75	Not stated
10297664	3785	USH2A	NM_206933.4	c.2802T>G	p.(Cys934Trp)	Missense	Pathogenic	M	54	Black - African
10297664	3785	USH2A	NM_206933.4	c.2802T>G	p.(Cys934Trp)	Missense	Pathogenic	M	54	Black - African
11899012	3786	USH2A	NM_206933.4	c.10561T>C	p.(Trp3521Arg)	Missense	Pathogenic	F	33	White - British
11899012	3786	USH2A	NM_206933.4	c.7595-2144A>G	Splice	Splice	Pathogenic	F	33	White - British
10872441	3787	USH2A	NM_206933.4	c.2299del	p.(Glu767SerfsTer21)	Frameshift	Pathogenic	F	37	Not stated
10872441	3787	USH2A	NM_206933.4	c.2299del	p.(Glu767SerfsTer21)	Frameshift	Pathogenic	F	37	Not stated
10002271	3788	USH2A	NM_206933.4	c.2023C>T	p.(Gln675Ter)	Stopgain	Pathogenic	M	63	Not stated
10002271	3788	USH2A	NM_206933.4	c.2299del	p.(Glu767SerfsTer21)	Frameshift	Pathogenic	M	63	Not stated
11520865	3789	USH2A	NM_206933.4	c.11694del	p.(Asn3899ThrfsTer34)	Frameshift	Pathogenic	F	40	White - British
11520865	3789	USH2A	NM_206933.4	c.3158-6A>G	Splice	Splice	Pathogenic	F	40	White - British
11929098	3790	USH2A	NM_206933.4	c.2276G>T	p.(Cys759Phe)	Missense	Pathogenic	M	47	White - Irish
11929098	3790	USH2A	NM_206933.4	c.2299del	p.(Glu767SerfsTer21)	Frameshift	Pathogenic	M	47	White - Irish
12133575	3791	USH2A	NM_206933.4	c.12954C>A	p.(Tyr4318Ter)	Stopgain	Pathogenic	M	61	White - British
12133575	3791	USH2A	NM_206933.4	c.5603T>G	p.(Phe1868Cys)	Missense	Likely Pathogenic	M	61	White - British
10614379	3792	USH2A	NM_206933.4	c.11875_11876del	p.(Gln3959AsnfsTer53)	Frameshift	Pathogenic	M	48	Any other ethnic group
10614379	3792	USH2A	NM_206933.4	c.13274C>T	p.(Thr4425Met)	Missense	Pathogenic	M	48	Any other ethnic group
12301974	3793	USH2A	NM_206933.4	c.2299del	p.(Glu767SerfsTer21)	Frameshift	Pathogenic	F	35	White - British
12301974	3793	USH2A	NM_206933.4	c.6118T>G	p.(Cys2040Gly)	Missense	Variant of Uncertain Significance	F	35	White - British
12301974	3793	USH2A	NM_206933.4	c.7475C>T	p.(Ser2492Leu)	Missense	Likely Pathogenic	F	35	White - British
12329841	3794	USH2A	NM_206933.4	c.12574C>T	p.(Arg4192Cys)	Missense	Likely Pathogenic	M	46	White - British
12329841	3794	USH2A	NM_206933.4	c.2276G>T	p.(Cys759Phe)	Missense	Pathogenic	M	46	White - British
12349679	3795	USH2A	NM_206933.4	c.11927C>T	p.(Thr3976Met)	Missense	Variant of Uncertain Significance	F	28	Not stated
12349679	3795	USH2A	NM_206933.4	c.2299del	p.(Glu767SerfsTer21)	Frameshift	Pathogenic	F	28	Not stated
11519955	3796	USH2A	NM_206933.4	c.11864G>A	p.(Trp3955Ter)	Stopgain	Pathogenic	M	34	Not stated
11519955	3796	USH2A	NM_206933.4	c.8618T>G	p.(Leu2873Ter)	Stopgain	Pathogenic	M	34	Not stated
12345395	3797	USH2A	NM_206933.4	c.12574C>T	p.(Arg4192Cys)	Missense	Likely Pathogenic	M	48	Not stated
12345395	3797	USH2A	NM_206933.4	c.4510dup	p.(Arg1504LysfsTer26)	Frameshift	Pathogenic	M	48	Not stated
12510301	3798	USH2A	NM_206933.4	c.2299del	p.(Glu767SerfsTer21)	Frameshift	Pathogenic	M	24	White - British
12510301	3798	USH2A	NM_206933.4	c.2299del	p.(Glu767SerfsTer21)	Frameshift	Pathogenic	M	24	White - British
12252568	3799	USH2A	NM_206933.4	c.10488_10490del	p.(Glu3496del)	Inframe indel	Variant of Uncertain Significance	M	32	Not stated
12252568	3799	USH2A	NM_206933.4	c.12954C>A	p.(Tyr4318Ter)	Stopgain	Pathogenic	M	32	Not stated
9858995	3800	USH2A	NM_206933.4	c.10073G>A	p.(Cys3358Tyr)	Missense	Pathogenic	F	37	Not stated
9858995	3800	USH2A	NM_206933.4	c.12574C>T	p.(Arg4192Cys)	Missense	Likely Pathogenic	F	37	Not stated
12313111	3801	USH2A	NM_206933.4	c.12505A>G	p.(Thr4169Ala)	Missense	Variant of Uncertain Significance	F	44	Not stated
12313111	3801	USH2A	NM_206933.4	c.12505A>G	p.(Thr4169Ala)	Missense	Variant of Uncertain Significance	F	44	Not stated
12555087	3802	USH2A	NM_206933.4	c.1859G>T	p.(Cys620Phe)	Missense	Pathogenic	M	69	Not stated
12555087	3802	USH2A	NM_206933.4	c.2276G>T	p.(Cys759Phe)	Missense	Pathogenic	M	69	Not stated
6733936	3803	USH2A	NM_206933.4	c.9860_9873del	p.(His3287ProfsTer54)	Frameshift	Pathogenic	F	38	Asian - Indian
6733936	3803	USH2A	NM_206933.4	c.9860_9873del	p.(His3287ProfsTer54)	Frameshift	Pathogenic	F	38	Asian - Indian
11792017	3804	USH2A	NM_206933.4	c.3902G>T	p.(Gly1301Val)	Missense	Benign	F	38	Not stated
11792017	3804	USH2A	NM_206933.4	c.7334C>T	p.(Ser2445Phe)	Missense	Benign	F	38	Not stated

12293077	3805	USH2A	NM_206933.4	c.3831_3834delinsG	p.(Leu1278del)	Inframe indel	Variant of Uncertain Significance	M	36	White - British
12293077	3805	USH2A	NM_206933.4	c.920_923dup	p.(His308GlnfsTer16)	Frameshift	Pathogenic	M	36	White - British
12776245	3806	USH2A	NM_206933.4	c.13274C>T	p.(Thr4425Met)	Missense	Pathogenic	F	60	Not stated
12776245	3806	USH2A	NM_206933.4	c.8981G>A	p.(Trp2994Ter)	Stopgain	Pathogenic	F	60	Not stated
10948811	3807	USH2A	NM_206933.4	c.13374del	p.(Glu4458AspfsTer3)	Frameshift	Pathogenic	F	51	Any other ethnic group
10948811	3807	USH2A	NM_206933.4	c.2276G>T	p.(Cys759Phe)	Missense	Pathogenic	F	51	Any other ethnic group
12735610	3808	USH2A	NM_206933.4	c.653T>A	p.(Val218Glu)	Missense	Pathogenic	F	43	Unknown
12735610	3808	USH2A	NM_206933.4	c.9882C>G	p.(Cys3294Trp)	Missense	Likely Pathogenic	F	43	Unknown
11217401	3809	USH2A	NM_206933.4	c.2276G>T	p.(Cys759Phe)	Missense	Pathogenic	F	69	Not stated
11217401	3809	USH2A	NM_206933.4	c.6446C>A	p.(Pro2149Gln)	Missense	Pathogenic	F	69	Not stated
8222864	3810	USH2A	NM_206933.4	c.2299del	p.(Glu767SerfsTer21)	Frameshift	Pathogenic	M	67	White - British
8222864	3810	USH2A	NM_206933.4	c.6050-1G>A	Splice	Splice	Pathogenic	M	67	White - British
12872376	3811	USH2A	NM_206933.4	c.13576C>T	p.(Arg4526Ter)	Stopgain	Pathogenic	M	28	Any other ethnic group
12872376	3811	USH2A	NM_206933.4	c.4222C>T	p.(Gln1408Ter)	Stopgain	Likely Pathogenic	M	28	Any other ethnic group
12869086	3812	USH2A	NM_206933.4	c.2299del	p.(Glu767SerfsTer21)	Frameshift	Pathogenic	F	33	White - British
12869086	3812	USH2A	NM_206933.4	c.920_923dup	p.(His308GlnfsTer16)	Frameshift	Pathogenic	F	33	White - British
12327804	3813	USH2A	NM_206933.4	c.10559A>G	p.(Asn3520Ser)	Missense	Variant of Uncertain Significance	F	41	White - British
12327804	3813	USH2A	NM_206933.4	c.1256G>T	p.(Cys419Phe)	Missense	Likely Pathogenic	F	41	White - British
12327804	3813	USH2A	NM_206933.4	c.13750dup	p.(Thr4584AsnfsTer4)	Frameshift	Likely Pathogenic	F	41	White - British
12327804	3813	USH2A	NM_206933.4	c.6590C>T	p.(Thr2197Ile)	Missense	Variant of Uncertain Significance	F	41	White - British
12868687	3814	USH2A	NM_206933.4	c.13396C>T	p.(Pro4466Ser)	Missense	Likely Pathogenic	M	39	Unknown
12868687	3814	USH2A	NM_206933.4	c.13396C>T	p.(Pro4466Ser)	Missense	Likely Pathogenic	M	39	Unknown
12858530	3815	USH2A	NM_206933.4	c.2276G>T	p.(Cys759Phe)	Missense	Pathogenic	F	33	White - British
12858530	3815	USH2A	NM_206933.4	c.2299del	p.(Glu767SerfsTer21)	Frameshift	Pathogenic	F	33	White - British
9910515	3816	USH2A	NM_206933.4	c.9571-2A>G	Splice	Splice	Pathogenic	M	65	Not stated
9910515	3816	USH2A	NM_206933.4	c.2299del	p.(Glu767SerfsTer21)	Frameshift	Pathogenic	M	65	Not stated
13091546	3817	USH2A	NM_206933.4	c.3589del	p.(Ser1197ProfsTer40)	Frameshift	Pathogenic	M	39	Not stated
13091546	3817	USH2A	NM_206933.4	c.8981G>A	p.(Trp2994Ter)	Stopgain	Pathogenic	M	39	Not stated
11729101	3818	USH2A	NM_206933.4	c.2276G>T	p.(Cys759Phe)	Missense	Pathogenic	M	83	White - British
11729101	3818	USH2A	NM_206933.4	c.4510dup	p.(Arg1504LysfsTer26)	Frameshift	Pathogenic	M	83	White - British
13033845	3819	USH2A	NM_206933.4	c.13331C>T	p.(Pro4444Leu)	Missense	Pathogenic	F	46	Asian - Pakistani
13033845	3819	USH2A	NM_206933.4	c.8223+1G>C	Splice	Splice	Pathogenic	F	46	Asian - Pakistani
13033845	3819	USH2A	NM_206933.4	c.5555A>G	p.(His1852Arg)	Missense	Variant of Uncertain Significance	F	46	Asian - Pakistani
13100156	3820	USH2A	NM_206933.4	c.11507C>T	p.(Pro3836Leu)	Missense	Likely Pathogenic	F	65	Unknown
13100156	3820	USH2A	NM_206933.4	c.9571-2A>G	Splice	Splice	Pathogenic	F	65	Unknown
13096978	3821	USH2A	NM_206933.4	c.12525G>T	p.(Trp4175Cys)	Missense	Pathogenic	F	82	Not stated
13096978	3821	USH2A	NM_206933.4	c.13316C>T	p.(Thr4439Ile)	Missense	Pathogenic	F	82	Not stated
13138684	3822	USH2A	NM_206933.4	c.11694del	p.(Asn3899ThrfsTer34)	Frameshift	Pathogenic	M	49	Not stated
13138684	3822	USH2A	NM_206933.4	c.802G>A	p.(Gly268Arg)	Missense	Likely Pathogenic	M	49	Not stated
13237972	3823	USH2A	NM_206933.4	c.10073G>A	p.(Cys3358Tyr)	Missense	Pathogenic	M	73	White - British
13237972	3823	USH2A	NM_206933.4	c.2276G>T	p.(Cys759Phe)	Missense	Pathogenic	M	73	White - British
9320912	3824	USH2A	NM_206933.4	c.3518C>A	p.(Ser1173Ter)	Stopgain	Likely Pathogenic	F	25	White - British
9320912	3824	USH2A	NM_206933.4	c.920_923dup	p.(His308GlnfsTer16)	Frameshift	Pathogenic	F	25	White - British
12837824	3825	USH2A	NM_206933.4	c.10996T>G	p.(Cys3666Gly)	Missense	Likely Pathogenic	M	58	Not stated
12837824	3825	USH2A	NM_206933.4	c.5603T>G	p.(Phe1868Cys)	Missense	Likely Pathogenic	M	48	Not stated
13299845	3825	USH2A	NM_206933.4	c.10996T>G	p.(Cys3666Gly)	Missense	Likely Pathogenic	M	57	White - British
13299845	3825	USH2A	NM_206933.4	c.5603T>G	p.(Phe1868Cys)	Missense	Likely Pathogenic	M	57	White - British
13324562	3826	USH2A	NM_206933.4	c.163C>T	p.(Gln55Ter)	Stopgain	Pathogenic	M	27	White - British
13324562	3826	USH2A	NM_206933.4	c.2299del	p.(Glu767SerfsTer21)	Frameshift	Pathogenic	M	27	White - British
13326725	3826	USH2A	NM_206933.4	c.163C>T	p.(Gln55Ter)	Stopgain	Pathogenic	M	24	White - British
13326725	3826	USH2A	NM_206933.4	c.2299del	p.(Glu767SerfsTer21)	Frameshift	Pathogenic	M	24	White - British

12690789	3827	USH2A	NM_206933.4	Exon 47 deletion	Deletion	Deletion	Likely Pathogenic	F	45	Not stated
12690789	3827	USH2A	NM_206933.4	c.2802T>C	p.(Cys934Trp)	Missense	Variant of Uncertain Significance	F	45	Not stated
9429419	3828	USH2A	NM_206933.4	c.2299del	p.(Glu767SerfsTer21)	Frameshift	Pathogenic	F	29	White - British
9429419	3828	USH2A	NM_206933.4	c.3187_3188del	p.(Gln1063SerfsTer15)	Frameshift	Pathogenic	F	29	White - British
13384419	3829	USH2A	NM_206933.4	c.2299del	p.(Glu767SerfsTer21)	Frameshift	Pathogenic	M	46	Not stated
13384419	3829	USH2A	NM_206933.4	c.7595-2144A>G	Splice	Splice	Pathogenic	M	46	Not stated
13439684	3830	USH2A	NM_206933.4	c.2276G>T	p.(Cys759Phe)	Missense	Pathogenic	F	35	Any other ethnic group
13439684	3830	USH2A	NM_206933.4	c.4474G>T	p.(Glu1492Ter)	Stopgain	Pathogenic	F	35	Any other ethnic group
13349825	3831	USH2A	NM_206933.4	c.2299del	p.(Glu767SerfsTer21)	Frameshift	Pathogenic	F	38	Not stated
13349825	3831	USH2A	NM_206933.4	c.4251+1G>A	Splice	Splice	Likely Pathogenic	F	38	Not stated
1802128	3832	USH2A	NM_206933.4	c.3420_3423del	p.(Tyr1141ArgfsTer11)	Frameshift	Likely Pathogenic	F	50	Black - Caribbean
1802128	3832	USH2A	NM_206933.4	c.5528C>T	p.(Pro1843Leu)	Missense	Likely Pathogenic	F	50	Black - Caribbean
13378707	3833	USH2A	NM_206933.4	c.3187_3188del	p.(Gln1063SerfsTer15)	Frameshift	Pathogenic	M	35	Not stated
13378707	3833	USH2A	NM_206933.4	c.6486-1G>A	Splice	Splice	Pathogenic	M	35	Not stated
11663189	3834	USH2A	NM_206933.4	c.10073G>A	p.(Cys3358Tyr)	Missense	Pathogenic	F	66	Not stated
11663189	3834	USH2A	NM_206933.4	c.2299del	p.(Glu767SerfsTer21)	Frameshift	Pathogenic	F	66	Not stated
13744604	3835	USH2A	NM_206933.4	c.14180G>A	p.(Trp4727Ter)	Stopgain	Pathogenic	M	38	Not stated
13744604	3835	USH2A	NM_206933.4	c.2299del	p.(Glu767SerfsTer21)	Frameshift	Pathogenic	M	38	Not stated
12849353	3836	USH2A	NM_206933.4	c.12145G>A	p.(Ala4049Thr)	Missense	Likely Pathogenic	F	47	Not stated
12849353	3836	USH2A	NM_206933.4	c.9785G>T	p.(Gly3262Val)	Missense	Variant of Uncertain Significance	F	47	Not stated
8151639	3837	USH2A	NM_206933.4	c.14426C>T	p.(Thr4809Ile)	Missense	Pathogenic	M	36	Not stated
8151639	3837	USH2A	NM_206933.4	c.4133_4134del	p.(Leu1378GlnfsTer10)	Frameshift	Likely Pathogenic	M	36	Not stated
8095177	3838	USH2A	NM_206933.4	c.12356T>C	p.(Phe4119Ser)	Missense	Variant of Uncertain Significance	F	63	White - British
8095177	3838	USH2A	NM_206933.4	c.2299del	p.(Glu767SerfsTer21)	Frameshift	Pathogenic	F	63	White - British
13733481	3839	USH2A	NM_206933.4	c.10010G>T	p.(Cys3337Phe)	Missense	Variant of Uncertain Significance	M	35	White - British
13733481	3839	USH2A	NM_206933.4	c.11516A>G	p.(Gln3839Arg)	Missense	Variant of Uncertain Significance	M	35	White - British
13746130	3840	USH2A	NM_206933.4	c.1256G>T	p.(Cys419Phe)	Missense	Likely Pathogenic	M	55	Not stated
13746130	3840	USH2A	NM_206933.4	c.2299del	p.(Glu767SerfsTer21)	Frameshift	Pathogenic	M	55	Not stated
13120036	3841	USH2A	NM_206933.4	c.6967C>T	p.(Arg2323Ter)	Stopgain	Pathogenic	M	46	Not stated
13120036	3841	USH2A	NM_206933.4	c.7883dup	p.(Ser2629LysfsTer7)	Frameshift	Likely Pathogenic	M	46	Not stated
13441735	3842	USH2A	NM_206933.4	c.2299del	p.(Glu767SerfsTer21)	Frameshift	Pathogenic	F	25	Not stated
13441735	3842	USH2A	NM_206933.4	c.949C>A	p.(Arg317Arg)	Synonymous	Likely Pathogenic	F	25	Not stated
13869323	3843	USH2A	NM_206933.4	c.2299del	p.(Glu767SerfsTer21)	Frameshift	Pathogenic	M	45	Not stated
13869323	3843	USH2A	NM_206933.4	c.802G>A	p.(Gly268Arg)	Missense	Likely Pathogenic	M	45	Not stated
13056623	3844	USH2A	NM_206933.4	c.8431C>A	p.(Pro2811Thr)	Missense	Variant of Uncertain Significance	F	40	Asian - Indian
13056623	3844	USH2A	NM_206933.4	c.9860_9873del	p.(His3287ProfsTer54)	Frameshift	Pathogenic	F	40	Asian - Indian
11355770	3845	USH2A	NM_206933.4	c.10999A>C	p.(Thr3667Pro)	Missense	Variant of Uncertain Significance	F	60	Not stated
11355770	3845	USH2A	NM_206933.4	c.9976C>T	p.(Gln3326Ter)	Stopgain	Pathogenic	F	60	Not stated
12629182	3846	USH2A	NM_206933.4	c.13335_13347delinsCTTG	.(Glu4445_Ser4449delinsAspLeu	Inframe indel	Pathogenic	M	53	White - Other
12629182	3846	USH2A	NM_206933.4	c.14301_14302del	p.(Tyr4768GlnfsTer11)	Frameshift	Likely Pathogenic	M	53	White - Other
6332724	3847	USH2A	NM_206933.4	c.12874A>G	p.(Asn4292Asp)	Missense	Likely Pathogenic	M	55	Asian - Pakistani
6332724	3847	USH2A	NM_206933.4	c.13126T>G	p.(Trp4376Gly)	Missense	Likely Pathogenic	M	55	Asian - Pakistani
10089526	3848	USH2A	NM_206933.4	c.12819T>A	p.(Tyr4273Ter)	Stopgain	Pathogenic	M	39	Not stated
10089526	3848	USH2A	NM_206933.4	c.2299del	p.(Glu767SerfsTer21)	Frameshift	Pathogenic	M	39	Not stated
14925210	3849	USH2A	NM_206933.4	c.1256G>T	p.(Cys419Phe)	Missense	Likely Pathogenic	F	31	Not stated
14925210	3849	USH2A	NM_206933.4	c.12574C>T	p.(Arg4192Cys)	Missense	Likely Pathogenic	F	31	Not stated
13470820	3850	USH2A	NM_206933.4	c.2276G>T	p.(Cys759Phe)	Missense	Pathogenic	M	33	Not stated
13470820	3850	USH2A	NM_206933.4	c.6470del	p.(Arg2157LysfsTer13)	Frameshift	Pathogenic	M	33	Not stated
13777294	3851	USH2A	NM_206933.4	c.1876C>T	p.(Arg626Ter)	Stopgain	Pathogenic	F	27	White - British
13777294	3851	USH2A	NM_206933.4	c.9328C>T	p.(Pro3110Ser)	Missense	Variant of Uncertain Significance	F	27	White - British
14826041	3852	USH2A	NM_206933.4	c.13335_13347delinsCTTG	p.(Glu4445_4449delinsAspLeu)	Inframe indel	Pathogenic	F	32	Any other ethnic group

14826041	3852	USH2A	NM_206933.4	c.9459C>A	p.(Cys3153Ter)	Stopgain	Pathogenic	F	32	Any other ethnic group
12381123	3853	USH2A	NM_206933.4	c.11156G>A	p.(Arg3719His)	Missense	Pathogenic	F	39	White - British
12381123	3853	USH2A	NM_206933.4	c.2299del	p.(Glu767SerfsTer21)	Frameshift	Pathogenic	F	39	White - British
6473914	3854	USH2A	NM_206933.4	c.2276G>T	p.(Cys759Phe)	Missense	Pathogenic	F	30	White - British
6473914	3854	USH2A	NM_206933.4	c.2299del	p.(Glu767SerfsTer21)	Frameshift	Pathogenic	F	30	White - British
11861086	3855	USH2A	NM_206933.4	c.2299del	p.(Glu767SerfsTer21)	Frameshift	Pathogenic	M	31	Not stated
11861086	3855	USH2A	NM_206933.4	c.4086del	p.(Val1363TyrfsTer3)	Frameshift	Pathogenic	M	31	Not stated
13994105	3856	USH2A	NM_206933.4	c.2296T>C	p.(Cys766Arg)	Missense	Pathogenic	M	31	White - Other
13994105	3856	USH2A	NM_206933.4	c.908G>A	p.(Arg303His)	Missense	Pathogenic	M	31	White - Other
15409743	3857	USH2A	NM_206933.4	c.10073G>A	p.(Cys3358Tyr)	Missense	Pathogenic	M	76	Unknown
15409743	3857	USH2A	NM_206933.4	c.2299del	p.(Glu767SerfsTer21)	Frameshift	Pathogenic	M	76	Unknown
13791413	3858	USH2A	NM_206933.4	c.2299del	p.(Glu767SerfsTer21)	Frameshift	Pathogenic	M	55	White - British
13791413	3858	USH2A	NM_206933.4	c.2299del	p.(Glu767SerfsTer21)	Frameshift	Pathogenic	M	55	White - British
8538494	3859	USH2A	NM_206933.4	c.11156G>A	p.(Arg3719His)	Missense	Pathogenic	M	50	Asian - Other
8538494	3859	USH2A	NM_206933.4	c.3902G>T	p.(Gly1301Val)	Missense	Variant of Uncertain Significance	M	50	Asian - Other
12316254	3860	USH2A	NM_206933.4	c.10342G>A	p.(Glu3448Lys)	Missense	Likely Pathogenic	M	63	Any other ethnic group
12316254	3860	USH2A	NM_206933.4	c.10342G>A	p.(Glu3448Lys)	Missense	Likely Pathogenic	M	63	Any other ethnic group
11502833	3861	USH2A	NM_206933.4	c.9860_9873del	p.(His3287ProfsTer54)	Frameshift	Pathogenic	F	45	Unknown
11502833	3861	USH2A	NM_206933.4	c.9860_9873del	p.(His3287ProfsTer54)	Frameshift	Pathogenic	F	45	Unknown
6998697	3862	USH2A	NM_206933.4	c.5776+1G>A	Splice	Splice	Pathogenic	F	38	Not stated
6998697	3862	USH2A	NM_206933.4	c.5776+1G>A	Splice	Splice	Pathogenic	F	38	Not stated
14962716	3863	USH2A	NM_206933.4	c.10073G>A	p.(Cys3358Tyr)	Missense	Pathogenic	M	42	Not stated
14962716	3863	USH2A	NM_206933.4	c.2299del	p.(Glu767SerfsTer21)	Frameshift	Pathogenic	M	42	Not stated
15611889	3864	USH2A	NM_206933.4	c.14512_14525del	p.(Gly4838LysfsTer21)	Frameshift	Likely Pathogenic	F	39	Any other ethnic group
15611889	3864	USH2A	NM_206933.4	c.2299del	p.(Glu767SerfsTer21)	Frameshift	Pathogenic	F	39	Any other ethnic group
15715146	3865	USH2A	NM_206933.4	c.2299del	p.(Glu767SerfsTer21)	Frameshift	Pathogenic	M	46	White - British
15715146	3865	USH2A	NM_206933.4	c.920_923dup	p.(His308GlnfsTer16)	Frameshift	Pathogenic	M	46	White - British
15296224	3866	USH2A	NM_206933.4	c.1256G>A	p.(Cys419Tyr)	Missense	Variant of Uncertain Significance	M	45	Not stated
15296224	3866	USH2A	NM_206933.4	c.13018G>C	p.(Gly4340Arg)	Missense	Pathogenic	M	45	Not stated
14755061	3867	USH2A	NM_206933.4	c.2276G>T	p.(Cys759Phe)	Missense	Pathogenic	F	73	Any other ethnic group
14755061	3867	USH2A	NM_206933.4	c.264C>G	p.(Cys88Trp)	Missense	Likely Pathogenic	F	73	Any other ethnic group
15883125	3868	USH2A	NM_206933.4	c.13649T>G	p.(Val4550Gly)	Missense	Variant of Uncertain Significance	F	53	Not stated
15883125	3868	USH2A	NM_206933.4	c.13812-1G>A	Splice	Splice	Pathogenic	F	53	Not stated
14972894	3869	USH2A	NM_206933.4	c.10073G>A	p.(Cys3358Tyr)	Missense	Pathogenic	F	38	Not stated
14972894	3869	USH2A	NM_206933.4	c.11864G>A	p.(Trp3955Ter)	Stopgain	Pathogenic	F	38	Not stated
10443439	3870	USH2A	NM_206933.4	c.11206G>A	p.(Asp3736Asn)	Missense	Variant of Uncertain Significance	F	53	Black - African
10443439	3870	USH2A	NM_206933.4	Exon 46-48 deletion	Deletion	Deletion	Likely Pathogenic	F	53	Black - African
8269890	3871	USH2A	NM_206933.4	c.2299del	p.(Glu767SerfsTer21)	Frameshift	Pathogenic	M	36	White - British
8269890	3871	USH2A	NM_206933.4	c.920_923dup	p.(His308GlnfsTer16)	Frameshift	Pathogenic	M	36	White - British
7914045	3872	USH2A	NM_206933.4	c.6967C>T	p.(Arg2323Ter)	Stopgain	Pathogenic	M	34	Asian - Indian
7914045	3872	USH2A	NM_206933.4	c.770G>A	p.(Gly257Glu)	Missense	Variant of Uncertain Significance	M	34	Asian - Indian
15163056	3873	USH2A	NM_206933.4	c.10342G>A	p.(Glu3448Lys)	Missense	Likely Pathogenic	M	49	Unknown
15163056	3873	USH2A	NM_206933.4	c.11389+2dup	Splice	Splice	Likely Pathogenic	M	49	Unknown
11686114	3874	USH2A	NM_206933.4	c.9860_9873del	p.(His3287ProfsTer54)	Frameshift	Pathogenic	F	33	Asian - Indian
11686114	3874	USH2A	NM_206933.4	c.9860_9873del	p.(His3287ProfsTer54)	Frameshift	Pathogenic	F	33	Asian - Indian
15987649	3875	USH2A	NM_206933.4	c.11065C>T	p.(Arg3689Ter)	Stopgain	Pathogenic	F	72	White - British
15987649	3875	USH2A	NM_206933.4	c.2299del	p.(Glu767SerfsTer21)	Frameshift	Pathogenic	F	72	White - British
16077333	3876	USH2A	NM_206933.4	c.13466dup	p.(Glu4491GlyfsTer6)	Frameshift	Likely Pathogenic	M	35	White - Other
16077333	3876	USH2A	NM_206933.4	Exon 10-11 deletion	Deletion	Deletion	Likely Pathogenic	M	35	White - Other
15144023	3877	USH2A	NM_206933.4	c.2299del	p.(Glu767SerfsTer21)	Frameshift	Pathogenic	F	69	Not stated
15144023	3877	USH2A	NM_206933.4	c.2299del	p.(Glu767SerfsTer21)	Frameshift	Pathogenic	F	69	Not stated

13273518	3878	USH2A	NM_206933.4	c.2299del	p.(Glu767SerfsTer21)	Frameshift	Pathogenic	F	48	Any other ethnic group
13273518	3878	USH2A	NM_206933.4	c.2299del	p.(Glu767SerfsTer21)	Frameshift	Pathogenic	F	48	Any other ethnic group
12575506	3879	USH2A	NM_206933.4	c.13576C>T	p.(Arg4526Ter)	Stopgain	Pathogenic	M	73	Asian - Indian
12575506	3879	USH2A	NM_206933.4	c.4222C>T	p.(Gln1408Ter)	Stopgain	Likely Pathogenic	M	73	Asian - Indian
7819734	3880	USH2A	NM_206933.4	c.1256G>T	p.(Cys419Phe)	Missense	Likely Pathogenic	F	56	White - British
7819734	3880	USH2A	NM_206933.4	c.2276G>T	p.(Cys759Phe)	Missense	Pathogenic	F	56	White - British
15780001	3881	USH2A	NM_206933.4	c.1226G>A	p.(Trp409Ter)	Stopgain	Pathogenic	M	29	Asian - Bangladeshi
15780001	3881	USH2A	NM_206933.4	c.1546G>A	p.(Gly516Arg)	Missense	Variant of Uncertain Significance	M	29	Asian - Bangladeshi
16199714	3882	USH2A	NM_206933.4	c.2299del	p.(Glu767SerfsTer21)	Frameshift	Pathogenic	M	33	White - British
16199714	3882	USH2A	NM_206933.4	c.2299del	p.(Glu767SerfsTer21)	Frameshift	Pathogenic	M	33	White - British
15140432	3883	USH2A	NM_206933.4	c.2299del	p.(Glu767SerfsTer21)	Frameshift	Pathogenic	F	60	White - British
15140432	3883	USH2A	NM_206933.4	c.2299del	p.(Glu767SerfsTer21)	Frameshift	Pathogenic	F	60	White - British
16422034	3884	USH2A	NM_206933.4	c.3407G>A	p.(Ser1136Asn)	Missense	Pathogenic	F	59	Not stated
16422034	3884	USH2A	NM_206933.4	c.8546G>T	p.(Gly2849Val)	Missense	Variant of Uncertain Significance	F	59	Not stated
17024867	3884	USH2A	NM_206933.4	c.3407G>A	p.(Ser1136Asn)	Missense	Pathogenic	F	56	Not stated
17024867	3884	USH2A	NM_206933.4	c.8546G>T	p.(Gly2849Val)	Missense	Variant of Uncertain Significance	F	56	Not stated
8303791	3885	USH2A	NM_206933.4	c.4222C>T	p.(Gln1408Ter)	Stopgain	Likely Pathogenic	F	50	Asian - Bangladeshi
8303791	3885	USH2A	NM_206933.4	c.4222C>T	p.(Gln1408Ter)	Stopgain	Likely Pathogenic	F	50	Asian - Bangladeshi
14874124	3886	USH2A	NM_206933.4	c.12874A>G	p.(Asn4292Asp)	Missense	Likely Pathogenic	F	35	Not stated
14874124	3886	USH2A	NM_206933.4	c.12874A>G	p.(Asn4292Asp)	Missense	Likely Pathogenic	F	35	Not stated
15793210	3887	USH2A	NM_206933.4	c.3737dup	p.(Ser1247LysfsTer4)	Frameshift	Pathogenic	M	39	Any other ethnic group
15793210	3887	USH2A	NM_206933.4	c.486-14G>A	Splice	Splice	Likely Pathogenic	M	39	Any other ethnic group
16752588	3888	USH2A	NM_206933.4	c.2299del	p.(Glu767SerfsTer21)	Frameshift	Pathogenic	M	71	Not stated
16752588	3888	USH2A	NM_206933.4	c.9799T>C	p.(Cys3267Arg)	Missense	Pathogenic	M	71	Not stated
7070482	3889	USH2A	NM_206933.4	c.2299del	p.(Glu767SerfsTer21)	Frameshift	Pathogenic	F	24	White - British
7070482	3889	USH2A	NM_206933.4	c.7814C>G	p.(Ser2605Ter)	Stopgain	Likely Pathogenic	F	24	White - British
15598309	3890	USH2A	NM_206933.4	c.12268C>A	p.(Pro4090Thr)	Missense	Pathogenic	M	37	Any other ethnic group
15598309	3890	USH2A	NM_206933.4	c.14791+2T>A	Splice	Splice	Pathogenic	M	37	Any other ethnic group
15560901	3891	USH2A	NM_206933.4	c.2276G>T	p.(Cys759Phe)	Missense	Pathogenic	F	85	White - British
15560901	3891	USH2A	NM_206933.4	c.2299del	p.(Glu767SerfsTer21)	Frameshift	Pathogenic	F	85	White - British
16938872	3892	USH2A	NM_206933.4	c.15017C>T	p.(Thr5006Met)	Missense	Likely Pathogenic	M	34	White - British
16938872	3892	USH2A	NM_206933.4	c.2299del	p.(Glu767SerfsTer21)	Frameshift	Pathogenic	M	34	White - British
16938872	3892	USH2A	NM_206933.4	c.802G>A	p.(Gly268Arg)	Missense	Likely Pathogenic	M	34	White - British
16013542	3893	USH2A	NM_206933.4	c.6795_6797del	p.(Glu2265_Tyr2266delinsAsp)	Inframe indel	Likely Pathogenic	F	27	Not stated
16013542	3893	USH2A	NM_206933.4	c.781_784+1375del	p.(Asn261ValfsTer2)	Frameshift	Likely Pathogenic	F	27	Not stated
13081095	3894	USH2A	NM_206933.4	c.13576C>T	p.(Arg4526Ter)	Stopgain	Pathogenic	F	51	Any other ethnic group
13081095	3894	USH2A	NM_206933.4	c.4222C>T	p.(Gln1408Ter)	Stopgain	Likely Pathogenic	F	51	Any other ethnic group
16531836	3895	USH2A	NM_206933.4	c.11864G>A	p.(Trp3955Ter)	Stopgain	Pathogenic	M	35	Any other ethnic group
16531836	3895	USH2A	NM_206933.4	c.8682-9A>G	Splice	Splice	Pathogenic	M	35	Any other ethnic group
11151104	3896	USH2A	NM_206933.4	c.1111_1112del	p.(Ile371PhefsTer3)	Frameshift	Pathogenic	M	75	Not stated
11151104	3896	USH2A	NM_206933.4	c.2276G>T	p.(Cys759Phe)	Missense	Pathogenic	M	75	Not stated
17147220	3897	USH2A	NM_206933.4	c.12295-3T>A	Splice	Splice	Pathogenic	M	33	White - British
17147220	3897	USH2A	NM_206933.4	c.2299del	p.(Glu767SerfsTer21)	Frameshift	Pathogenic	M	33	White - British
17176095	3898	USH2A	NM_206933.4	c.14792-2A>G	Splice	Splice	Pathogenic	M	35	Not stated
17176095	3898	USH2A	NM_206933.4	c.2276G>T	p.(Cys759Phe)	Missense	Pathogenic	M	35	Not stated
17264932	3899	USH2A	NM_206933.4	c.2299del	p.(Glu767SerfsTer21)	Frameshift	Pathogenic	M	64	White - British
17264932	3899	USH2A	NM_206933.4	c.4714del	p.(Leu1572PhefsTer3)	Frameshift	Likely Pathogenic	M	64	White - British
17349562	3900	USH2A	NM_206933.4	c.10073G>A	p.(Cys3358Tyr)	Missense	Pathogenic	F	77	Not stated
17349562	3900	USH2A	NM_206933.4	c.13316C>T	p.(Thr4439Ile)	Missense	Pathogenic	F	77	Not stated
17395384	3901	USH2A	NM_206933.4	c.8981G>A	p.(Trp2994Ter)	Stopgain	Pathogenic	F	30	Not stated
17395384	3901	USH2A	NM_206933.4	c.9882C>G	p.(Cys3294Trp)	Missense	Likely Pathogenic	F	30	Not stated

17335191	3902	USH2A	NM_206933.4	c.2276G>T	p.(Cys759Phe)	Missense	Pathogenic	M	32	White - British
17335191	3902	USH2A	NM_206933.4	c.5217_5218del	p.(Glu1739AspfsTer4)	Frameshift	Likely Pathogenic	M	32	White - British
17443439	3903	USH2A	NM_206933.4	c.10073G>A	p.(Cys3358Tyr)	Missense	Pathogenic	F	54	Not stated
17443439	3903	USH2A	NM_206933.4	c.1111_1112del	p.(Ile371PhefsTer3)	Frameshift	Pathogenic	F	54	Not stated
16599533	3904	USH2A	NM_206933.4	c.13335_13347delinsCTTG	.(Glu4445_Ser4449delinsAspLeu	Inframe indel	Pathogenic	F	63	Not stated
16599533	3904	USH2A	NM_206933.4	c.2299del	p.(Glu767SerfsTer21)	Frameshift	Pathogenic	F	63	Not stated
16714571	3905	USH2A	NM_206933.4	c.4474G>T	p.(Glu1492Ter)	Stopgain	Pathogenic	F	52	Not stated
16714571	3905	USH2A	NM_206933.4	c.5614_5620del	p.(Ala1872LeufsTer64)	Frameshift	Likely Pathogenic	F	52	Not stated
17577909	3906	USH2A	NM_206933.4	c.10342G>A	p.(Glu3448Lys)	Missense	Likely Pathogenic	M	35	Not stated
17577909	3906	USH2A	NM_206933.4	c.852_853del	p.(Glu284AspfsTer38)	Frameshift	Pathogenic	M	35	Not stated
15899526	3907	USH2A	NM_206933.4	Exon 50-58 deletion	Deletion	Deletion	Likely Pathogenic	M	14	Not stated
15899526	3907	USH2A	NM_206933.4	Exon 50-58 deletion	Deletion	Deletion	Likely Pathogenic	M	14	Not stated
13493416	3908	USH2A	NM_206933.4	c.13621C>T	p.(Gln4541Ter)	Stopgain	Pathogenic	M	30	White - British
13493416	3908	USH2A	NM_206933.4	c.3296_3297del	p.(Thr1099ArgfsTer27)	Frameshift	Pathogenic	M	30	White - British
16441809	3909	USH2A	NM_206933.4	c.13621C>T	p.(Gln4541Ter)	Stopgain	Pathogenic	M	49	Not stated
16441809	3909	USH2A	NM_206933.4	c.2276G>T	p.(Cys759Phe)	Missense	Pathogenic	M	49	Not stated
17713947	3910	USH2A	NM_206933.4	c.13290_13291delinsT	p.(Ala4431LeufsTer30)	Frameshift	Likely Pathogenic	F	57	Not stated
17713947	3910	USH2A	NM_206933.4	c.926C>T	p.(Pro309Leu)	Missense	Likely Pathogenic	F	57	Not stated
17752370	3911	USH2A	NM_206933.4	c.14426C>T	p.(Thr4809Ile)	Missense	Pathogenic	M	47	Unknown
17752370	3911	USH2A	NM_206933.4	c.9449G>A	p.(Trp3150Ter)	Stopgain	Likely Pathogenic	M	47	Unknown
16917221	3912	USH2A	NM_206933.4	c.2276G>T	p.(Cys759Phe)	Missense	Pathogenic	F	45	Not stated
16917221	3912	USH2A	NM_206933.4	Exon 22-24 deletion	Deletion	Deletion	Likely Pathogenic	F	45	Not stated
17739210	3913	USH2A	NM_206933.4	c.2299del	p.(Glu767SerfsTer21)	Frameshift	Pathogenic	F	26	Not stated
17739210	3913	USH2A	NM_206933.4	c.2299del	p.(Glu767SerfsTer21)	Frameshift	Pathogenic	F	26	Not stated
17755765	3914	USH2A	NM_206933.4	c.2299del	p.(Glu767SerfsTer21)	Frameshift	Pathogenic	M	21	Not stated
17755765	3914	USH2A	NM_206933.4	c.926C>T	p.(Pro309Leu)	Missense	Likely Pathogenic	M	21	Not stated
16486196	3915	USH2A	NM_206933.4	c.1644+1G>A	Splice	Splice	Pathogenic	F	37	Not stated
16486196	3915	USH2A	NM_206933.4	c.2276G>T	p.(Cys759Phe)	Missense	Pathogenic	F	37	Not stated
17677428	3916	USH2A	NM_206933.4	c.12446G>A	p.(Trp4149Ter)	Stopgain	Likely Pathogenic	M	45	Not stated
17677428	3916	USH2A	NM_206933.4	c.1481A>G	p.(Tyr494Cys)	Missense	Variant of Uncertain Significance	M	45	Not stated
17850769	3917	USH2A	NM_206933.4	c.11194C>T	p.(Gln3732Ter)	Stopgain	Likely Pathogenic	M	41	Not stated
17850769	3917	USH2A	NM_206933.4	c.5776+1G>A	Splice	Splice	Pathogenic	M	41	Not stated
17917332	3918	USH2A	NM_206933.4	12294+1_12294+4delinsATCTACCA	Splice	Splice	Likely Pathogenic	F	19	Black - African
17917332	3918	USH2A	NM_206933.4	c.12574C>T	p.(Arg4192Cys)	Missense	Likely Pathogenic	F	19	Black - African
3968908	3919	USH2A	NM_206933.4	c.2299del	p.(Glu767SerfsTer21)	Frameshift	Pathogenic	F	80	Any other ethnic group
3968908	3919	USH2A	NM_206933.4	c.7945del	p.(His2649ThrfsTer25)	Frameshift	Likely Pathogenic	F	80	Any other ethnic group
18035667	3920	USH2A	NM_206933.4	c.10342G>A	p.(Glu3448Lys)	Missense	Likely Pathogenic	M	22	Not stated
18035667	3920	USH2A	NM_206933.4	c.7595-2144A>G	Splice	Splice	Pathogenic	M	22	Not stated
18093879	3921	USH2A	NM_206933.4	c.10450C>T	p.(Arg3484Ter)	Stopgain	Pathogenic	F	43	Not stated
18093879	3921	USH2A	NM_206933.4	c.10450C>T	p.(Arg3484Ter)	Stopgain	Pathogenic	F	43	Not stated
18105275	3922	USH2A	NM_206933.4	c.11712-2A>G	Splice	Splice	Pathogenic	M	36	Not stated
18105275	3922	USH2A	NM_206933.4	c.11712-2A>G	Splice	Splice	Pathogenic	M	36	Not stated
4849522	3923	USH2A	NM_206933.4	c.4732C>T	p.(Arg1578Cys)	Missense	Pathogenic	F	59	Any other ethnic group
4849522	3923	USH2A	NM_206933.4	c.7595-2144A>G	Splice	Splice	Pathogenic	F	59	Any other ethnic group
18126324	3924	USH2A	NM_206933.4	c.5588dup	p.(Met1863IlefsTer5)	Frameshift	Likely Pathogenic	M	54	Not stated
18126324	3924	USH2A	NM_206933.4	c.13649T>G	p.(Val4550Gly)	Missense	Variant of Uncertain Significance	M	54	Not stated
18171474	3925	USH2A	NM_206933.4	c.11189del	p.(Glu3730GlyfsTer20)	Frameshift	Pathogenic	M	20	Not stated
18171474	3925	USH2A	NM_206933.4	c.1859G>T	p.(Cys620Phe)	Missense	Pathogenic	M	20	Not stated
18182716	3925	USH2A	NM_206933.4	c.11189del	p.(Glu3730GlyfsTer20)	Frameshift	Pathogenic	M	20	Not stated
18182716	3925	USH2A	NM_206933.4	c.1859G>T	p.(Cys620Phe)	Missense	Pathogenic	M	20	Not stated
18136796	3926	USH2A	NM_206933.4	c.1001G>A	p.(Arg334Gln)	Missense	Likely Pathogenic	M	31	Not stated

18136796	3926	USH2A	NM_206933.4	c.3661C>T	p.(Gln1221Ter)	Stopgain	Pathogenic	M	31	Not stated
16102029	3927	USH2A	NM_206933.4	c.1391G>A	p.(Arg464His)	Missense	Pathogenic	F	69	Any other ethnic group
16102029	3927	USH2A	NM_206933.4	c.486-14G>A	Splice	Splice	Likely Pathogenic	F	69	Any other ethnic group
16185756	3928	USH2A	NM_206933.4	c.1618C>T	p.(Gln540Ter)	Stopgain	Likely Pathogenic	M	15	Not stated
16185756	3928	USH2A	NM_206933.4	c.2299del	p.(Glu767SerfsTer21)	Frameshift	Pathogenic	M	15	Not stated
16163188	3928	USH2A	NM_206933.4	c.1618C>T	p.(Gln540Ter)	Stopgain	Likely Pathogenic	M	15	Not stated
16163188	3928	USH2A	NM_206933.4	c.2299del	p.(Glu767SerfsTer21)	Frameshift	Pathogenic	M	15	Not stated
17937352	3929	USH2A	NM_206933.4	c.2299del	p.(Glu767SerfsTer21)	Frameshift	Pathogenic	F	29	Not stated
17937352	3929	USH2A	NM_206933.4	c.908G>A	p.(Arg303His)	Missense	Pathogenic	F	29	Not stated
18238317	3930	USH2A	NM_206933.4	c.1606T>C	p.(Cys536Arg)	Missense	Pathogenic	F	14	Not stated
18238317	3930	USH2A	NM_206933.4	c.7595-2144A>G	Splice	Splice	Pathogenic	F	14	Not stated
5828612	3931	USH2A	NM_206933.4	c.920_923dup	p.(His308GlnfsTer16)	Frameshift	Pathogenic	F	64	Not stated
5828612	3931	USH2A	NM_206933.4	c.920_923dup	p.(His308GlnfsTer16)	Frameshift	Pathogenic	F	64	Not stated
12317738	3932	USH2A	NM_206933.4	c.2276G>T	p.(Cys759Phe)	Missense	Pathogenic	F	75	Unknown
12317738	3932	USH2A	NM_206933.4	c.2299del	p.(Glu767SerfsTer21)	Frameshift	Pathogenic	F	75	Unknown
18440435	3933	USH2A	NM_206933.4	c.2276G>T	p.(Cys759Phe)	Missense	Pathogenic	F	40	Not stated
18440435	3933	USH2A	NM_206933.4	c.2276G>T	p.(Cys759Phe)	Missense	Pathogenic	F	40	Not stated
17716810	3934	USH2A	NM_206933.4	c.10073G>A	p.(Cys3358Tyr)	Missense	Pathogenic	M	53	Not stated
17716810	3934	USH2A	NM_206933.4	c.2276G>T	p.(Cys759Phe)	Missense	Pathogenic	M	53	Not stated
18132939	3935	USH2A	NM_206933.4	c.3187_3188del	p.(Gln1063SerfsTer15)	Frameshift	Pathogenic	F	33	Not stated
18132939	3935	USH2A	NM_206933.4	c.5203G>A	p.(Gly1735Arg)	Missense	Variant of Uncertain Significance	F	33	Not stated
18418448	3936	USH2A	NM_206933.4	c.1036A>C	p.(Asn346His)	Missense	Pathogenic	M	33	Unknown
18418448	3936	USH2A	NM_206933.4	c.264C>G	p.(Cys88Trp)	Missense	Likely Pathogenic	M	33	Unknown
18111932	3937	USH2A	NM_206933.4	c.1679del	p.(Pro560LeufsTer31)	Frameshift	Pathogenic	F	60	White - British
18111932	3937	USH2A	NM_206933.4	c.5037_5038delinsTT	ɔ.(Met1679_Lys1680delinsIleTer	Stopgain	Likely Pathogenic	F	60	White - British
18613048	3938	USH2A	NM_206933.4	c.10073G>A	p.(Cys3358Tyr)	Missense	Pathogenic	F	36	Not stated
18613048	3938	USH2A	NM_206933.4	c.1256G>T	p.(Cys419Phe)	Missense	Likely Pathogenic	F	36	Not stated
3029536	3939	USH2A	NM_206933.4	c.10073G>A	p.(Cys3358Tyr)	Missense	Pathogenic	M	99	Not stated
3029536	3939	USH2A	NM_206933.4	c.8834G>A	p.(Trp2945Ter)	Stopgain	Pathogenic	M	99	Not stated
18664708	3940	USH2A	NM_206933.4	c.10450C>T	p.(Arg3484Ter)	Stopgain	Pathogenic	F	25	Unknown
18664708	3940	USH2A	NM_206933.4	c.10450C>T	p.(Arg3484Ter)	Stopgain	Pathogenic	F	25	Unknown
18359963	3941	USH2A	NM_206933.4	c.2276G>T	p.(Cys759Phe)	Missense	Pathogenic	F	60	Not stated
18359963	3941	USH2A	NM_206933.4	c.2299del	p.(Glu767SerfsTer21)	Frameshift	Pathogenic	F	60	Not stated
18001535	3942	USH2A	NM_206933.4	c.4732C>T	p.(Arg1578Cys)	Missense	Pathogenic	F	31	Any other ethnic group
18001535	3942	USH2A	NM_206933.4	c.6326-1G>A	Splice	Splice	Likely Pathogenic	F	31	Any other ethnic group
18001535	3942	USH2A	NM_206933.4	c.8981G>A	p.(Trp2994Ter)	Stopgain	Pathogenic	F	31	Any other ethnic group
2185014	3943	USH2A	NM_206933.4	c.5012G>A	p.(Gly1671Asp)	Missense	Likely Pathogenic	F	72	Asian - Indian
2185014	3943	USH2A	NM_206933.4	c.5012G>A	p.(Gly1671Asp)	Missense	Likely Pathogenic	F	72	Asian - Indian
3660649	3943	USH2A	NM_206933.4	c.5012G>A	p.(Gly1671Asp)	Missense	Likely Pathogenic	F	39	Asian - Indian
3660649	3943	USH2A	NM_206933.4	c.5012G>A	p.(Gly1671Asp)	Missense	Likely Pathogenic	F	39	Asian - Indian
12519331	3944	VCAN	NM_004385.5	c.9265+1G>A	Splice	Splice	Pathogenic	F	47	White - British
16774505	3945	WDR19	NM_025132.4	c.1776A>G	p.(Gln592Gln)	Synonymous	Variant of Uncertain Significance	M	19	Not stated
16774505	3945	WDR19	NM_025132.4	c.1776A>G	p.(Gln592Gln)	Synonymous	Variant of Uncertain Significance	M	19	Not stated
6425208	3946	WFS1	NM_006005.3	c.2390A>T	p.(Asp797Val)	Missense	Likely Pathogenic	F	77	Not stated
8637901	3947	WFS1	NM_006005.3	c.968A>G	p.(His323Arg)	Missense	Likely Pathogenic	F	55	White - British
8419627	3947	WFS1	NM_006005.3	c.968A>G	p.(His323Arg)	Missense	Likely Pathogenic	F	19	White - British
9477768	3948	WFS1	NM_006005.3	c.2213C>A	p.(Ala738Asp)	Missense	Variant of Uncertain Significance	M	33	Not stated
9477768	3948	WFS1	NM_006005.3	c.2648_2651del	p.(Phe883SerfsTer68)	Frameshift	Pathogenic	M	33	Not stated
9538507	3948	WFS1	NM_006005.3	c.2213C>A	p.(Ala738Asp)	Missense	Variant of Uncertain Significance	F	35	Not stated
9538507	3948	WFS1	NM_006005.3	c.2648_2651del	p.(Phe883SerfsTer68)	Frameshift	Pathogenic	F	35	Not stated
9803415	3949	WFS1	NM_006005.3	c.505G>A	p.(Glu169Lys)	Missense	Likely Pathogenic	M	56	White - British

9803415	3949	WFS1	NM_006005.3	c.874C>A	p.(Pro292Thr)	Missense	Variant of Uncertain Significance	M	56	White - British
10180379	3950	WFS1	NM_006005.3	c.937C>T	p.(His313Tyr)	Missense	Likely Pathogenic	M	21	Not stated
16331055	3951	WFS1	NM_006005.3	c.2051C>T	p.(Ala684Val)	Missense	Pathogenic	M	24	White - British
3086642	3952	WFS1	NM_006005.3	c.346G>A	p.(Asp116Asn)	Missense	Variant of Uncertain Significance	M	53	Asian - Bangladeshi
17107999	3952	WFS1	NM_006005.3	c.346G>A	p.(Asp116Asn)	Missense	Variant of Uncertain Significance	M	6	Asian - Bangladeshi
17098815	3953	WFS1	NM_006005.3	c.2648_2651del	p.(Phe883SerfsTer68)	Frameshift	Pathogenic	F	19	Not stated
17098815	3953	WFS1	NM_006005.3	c.505G>A	p.(Glu169Lys)	Missense	Likely Pathogenic	F	19	Not stated
